# Alcohols as Alkylating Agents in the Cation‐Induced Formation of Nitrogen Heterocycles

**DOI:** 10.1002/anie.202206800

**Published:** 2022-07-13

**Authors:** Lydia Cox, Yuxiang Zhu, Philip J. Smith, Kirsten E. Christensen, Mireia Sidera Portela, Timothy J. Donohoe

**Affiliations:** ^1^ Department of Chemistry University of Oxford Chemistry Research Laboratory Mansfield Road Oxford OX1 3TA UK; ^2^ Vertex Pharmaceuticals 86–88 Jubilee Ave, Milton Abingdon OX14 4RW UK

**Keywords:** Azacycles, Carboamination, Carbocations, Hexafluoroisopropanol, Lewis Acid Catalysis

## Abstract

A Ti(O*i*‐Pr)_4_ promoted 5‐ or 6‐*endo*‐trig cyclisation to make nitrogen heterocycles is presented. The utilisation of HFIP as a key solvent enables the stereoselective preparation of di‐ & tri‐substituted pyrrolidines and piperidines while forming a new C−C bond at the same time. The process is triggered by a cationic intermediate generated from an allylic or benzylic alcohol and leads to the simultaneous generation of both a C−C and a C−N bond in a single step. Notably, either 2,3‐*trans‐* or 2,3‐*cis*‐substituted heterocycles can be obtained by using a nucleophilic amine bearing different substituents. Lastly, the stereoselective synthesis of enantiopure products was achieved by using readily available enantiopure acyclic starting materials.

## Graphical abstract

The use of HFIP as a solvent enables the stereoselective preparation of substituted pyrrolidines and piperidines from an acyclic precursor. The process is triggered by a cation generated from an alcohol in situ and leads to the simultaneous generation of both a C−C and a C−N bond. Note that either 2,3‐*trans‐* or 2,3‐*cis*‐substituted heterocycles can be obtained at will by using a nucleophilic amine bearing different substituents.

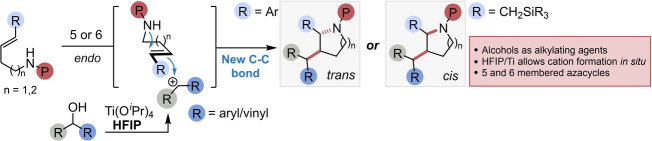

The prevalence of nitrogen‐containing saturated heterocycles in natural products and biologically active molecules has led to considerable efforts towards their synthesis.^[1]^ Although several methods exist to access important structures such as Atrasentan or Dienomycin‐C, the *hydroa*mination of alkenes has emerged as a popular approach to make these heterocycles.^[2]^ Activation of the amine or alkene partners using a catalytic transition metal or a Lewis or Brønsted acid is well established, offering complementary routes to prepare 2‐substituted *N*‐heterocycles.[^3^, ^4^, ^5^, ^6^] Conversely the more powerful *carbo*amination approach is much less well explored, despite the additional advantage of being able to simultaneously generate a C−C and C−N bond along with two new stereogenic centers in a single step (Scheme 1). Examples in the literature are limited to transition metal catalysed processes, with the alkene reacting in an *exocyclic* fashion, generating one stereocentre in the ring and a second in an exocyclic position.[^7^, ^8^] Thus, methods to generate rings with greater substitution and multiple stereocentres within the heterocyclic ring remain desirable.

**Scheme 1:**
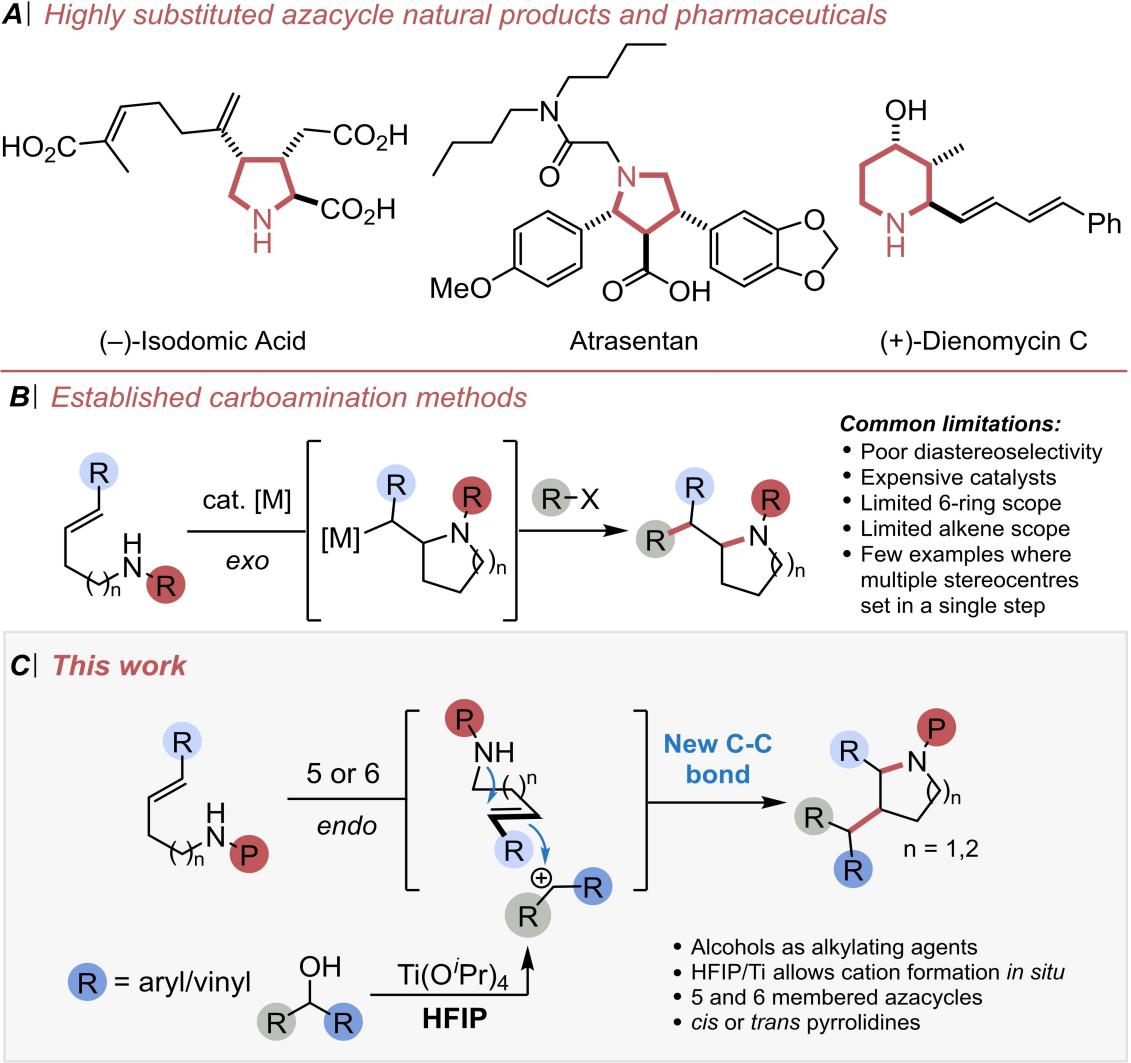
A) Desirable highly substituted azacycles. B) Established carboamination methods. C) Proposed synthesis of azacycles through a cation triggered annulation.

Herein, we report an alternative approach to preparing such molecules, whereby the activation of an external alcohol electrophile triggers the formation of a C−C bond in addition to concomitant cyclisation and formation of a C−N bond.[^9^, ^10^] In this route the alkene reacts in an *endo* fashion to access 2,3‐disubstituted *N*‐heterocycles with the additional advantage of being able to set a third exocyclic stereocenter if desired. Following our recent work using hexafluoroisopropanol (HFIP) as a solvent in the hetero‐cyclisation of homoallylic alcohols to afford tetrahydrofurans,^[11]^ we have now been able to expand this methodology to access both pyrrolidine and piperidine products. HFIP plays a crucial role in the transformation, enabling readily available alcohols to act as alkylating agents via the formation of a carbocation in situ, triggering the cyclisation and generating water as a stoichiometric byproduct.^[12]^ This synthetic approach enables the rapid construction of key azacycles and facilitates the preparation of a number of medicinally relevant pyrrolidines and piperidines.

Our attention was initially focused on the synthesis of pyrrolidines from homoallylic amines **1** and alcohols, and as a starting point we took advantage of the reaction conditions used in the synthesis of tetrahydrofurans (i.e. 30 mol% Ti(O*i*‐Pr)_4_ in 0.1 M HFIP), which were found to be optimal for this transformation (for details of screening studies, see Supporting Information). Benzhydrol **2** **a** was chosen as a suitable cation precursor, allowing us to evaluate the reactivity of a range of nitrogen protecting groups. Thus, a series of sulfonamides were succesfully employed in the synthesis of **3**, with tosyl **1** **a** and mesyl **1** **b** groups giving moderate yields, 64 % and 43 % respectively, but more importantly as single *trans* diastereomers, as confirmed by NOE experiments (Table 1). Switching to a nitro‐sulfonamide group was beneficial, with *ortho*‐nosyl **3** **c** out‐performing *para*‐nosyl **3** **d** by 10 %. Pleasingly, a Cbz carbamate **1** **e** was also tolerated in the cyclisation, albeit giving the product **3** **e** in a reduced yield of 77 %. However, the use of a benzyl protecting group gave no conversion to **3** **f** which was attributed to the enhanced basicity of the nitrogen atom, leading to deactivation under the reaction conditions.

**Table 1:**
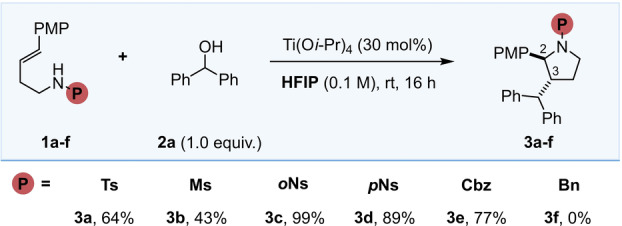
Protecting group screen using benzhydrol **2** **a** as a model electrophile.

^†^ *d.r. (C2–C3) >20 : 1*.

We then undertook a scope of the electrophilic (alcohol) component using the optimal *ortho‐*nosyl protected amine **1** **c**. Knowing that benzhydrol performed well in the reaction (Scheme 2) we were pleased to find that *one equivalent* of other simple benzylic and allylic alcohols were compatible with the cyclisation with **1** **c** (Scheme 2, **3** **g**–**3** **j**). Pleasingly, the reaction performed well upon scale‐up, producing **3** **c** in 95 % yield when run on a 2.50 mmol scale (1.25 g of **3** **c**). Tertiary alcohols were also screened as electrophiles, however they were found to perform poorly when compared to primary and secondary benzylic alcohols, giving diminished yields of 0–33 % (see Supporting Information).

**Scheme 2:**
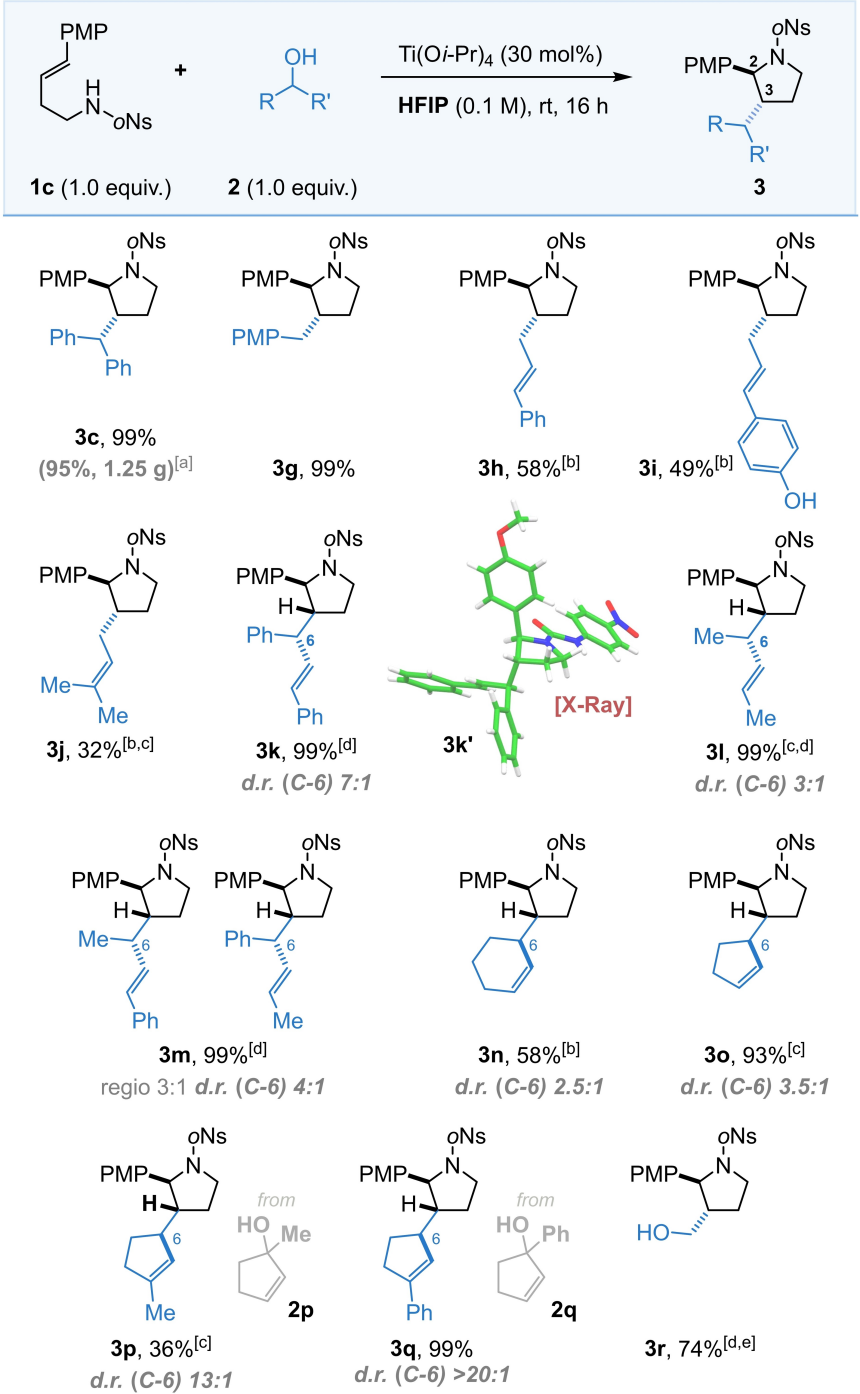
Substrate scope of the electrophilic partner. ^[a]^ Performed on a 2.50 mmol scale. ^[b]^ Performed at 70 °C. ^[c]^ Using 2.0 equiv. of electrophilic partner. ^[d]^ Performed at 0 °C for 48 h. ^[e]^ Using 20 equiv. of electrophilic partner. ^†^ *d.r. (C2*–*C3) >20 : 1*.

In addition to being able to set the 2,3‐ring (*trans*) stereochemistry, a range of acyclic α‐substituted allylic alcohols were then used to set an additional exocyclic stereogenic centre, affording products with three contiguous stereocenters (Scheme 2, **3** **k**–**3** **m**). While each reaction afforded exclusively the 2,3‐*trans* stereochemistry with notably high yield, we also observed good diastereoselectivity at the *exo*cyclic stereocenter (the levels depended on the steric bulk of substitution, compare **3** **k** with **3** **l**). When an allylic alcohol bearing two different terminal substituents was used, partial regiocontrol was also observed (Scheme 2, **3** **m**) stemming from preferential attack at the least hindered end of an unsymmetrical allyl cation intermediate. Whilst the ring stereochemistry could be confirmed through NOESY data, the stereochemistry of the exocyclic centres was elucidated through single crystal X‐ray diffraction on a derivative of **3** **k**, (**3** **k**′,^[13]^ with compounds **3** **l**–**3** **m** assigned by analogy).

Next, a diverse range of cyclic allylic alcohols were utilised in order to increase the scope of the method. Pleasingly, both cyclopentenyl and cyclohexenyl alcohols gave moderate to good diastereocontrol at the exocyclic stereocentre (Scheme 2, **3** **n** and **3** **o**). Moreover, the use of methyl or phenyl substituted tertiary cyclopentene derived allyl alcohols **2** **p** and **2** **q** led to pyrrolidines **3** **p** and **3** **q** with high to complete diastereocontrol at the exocyclic centre coupled with complete regioselectivity. When cyclic allylic alcohols were used as electrophilles it was not possible to unambiguously confirm the relative stereochemistry at the exocyclic centres; here assignments have been made by analogy to the previously synthesised tetrahydrofuran products through careful comparison of the NMR data with the corresponding THF products.[^11^, ^14^] Finally, other non‐alcohol electrophiles were also screened to enhance the scope of the electrophile and we were delighted to discover that paraformaldehyde could be utilised to access pyrrolidine **3** **r** in 74 % yield (Scheme 2).

Having demonstrated the scope of the electrophile, attention moved to explore the versatility of the nucleophilic alkene partner **1**. Both electron‐neutral (Scheme 3, **3** **s** and **3** **t**) and ‐deficient aryl rings (Scheme 3, **3** **u**) were tolerated with satisfying results being obtained. To our delight, employing tri‐substituted aliphatic alkenes as the nucleophile allowed access to pyrrolidines bearing a fully substituted center including a spirocycle (Scheme 3, **3** **v** and **3** **w**).

**Scheme 3:**
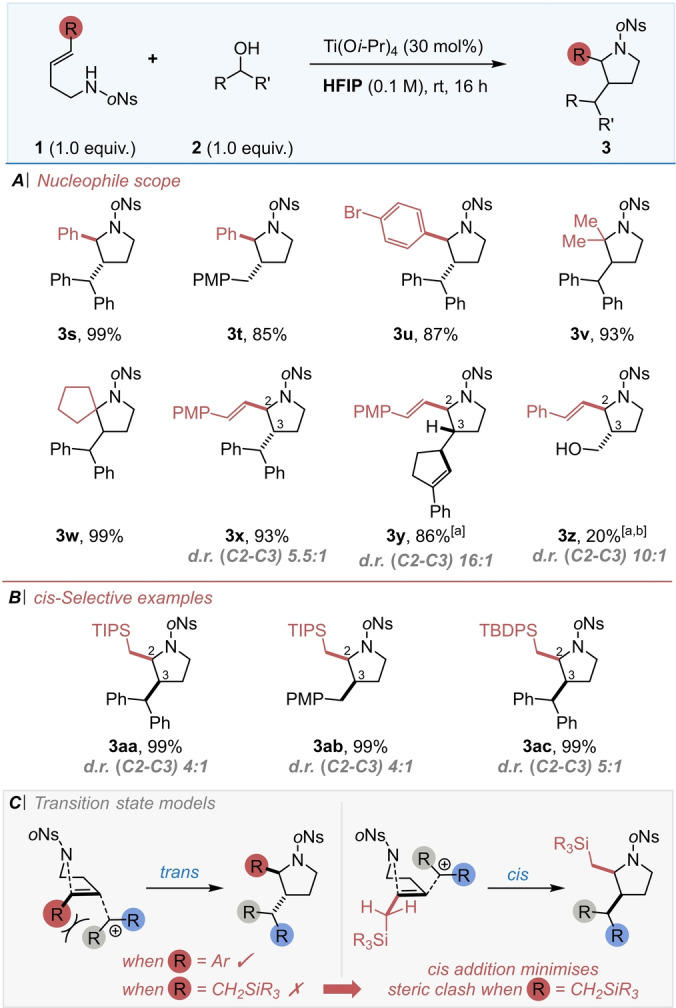
(A) Substrate scope of the nucleophilic partner. (B) c*is*‐Selective substrate scope. (C) Suggested transition state models to rationalise the reversal in stereoselectivity. ^[a]^ Performed at 0 °C for 48 h. ^[b]^ Using 20 equiv. of paraformaldehyde as the electrophilic partner. ^†^ *d.r. (C2–C3) >20 : 1* unless otherwise stated.

Of particular interest was the inclusion of a reactive functionality at the pyrrolidine C2‐position which could be easily derivatised to access a wider range of functional groups. To this end, two distinct tactics were established, firstly through the introduction of an *exo*‐cyclic vinyl group (rather than an aryl group). In this case a conjugated diene was used to access pyrrolidines (Scheme 3, **3** **x**–**3** **z)** with complete regioselectivity and satisfying (*trans*) diastereocontrol depending on the electrophile employed. Our second approach utilised nucleophilic allyl silanes, which cyclised well and, to our delight, gave access to the 2,3‐*cis* products (see Scheme 3B). Both TIPS‐ and TBDPS‐substituted alkenes gave pyrrolidines in moderate to excellent yield, and the *cis* diastereoselectivity largely depended on the nature of the electrophile **2** employed (Scheme 3, **3** **aa**–**3** **ac**). This modification marks a significant advance in our method, with a judicious choice of substrate now allowing us to preferentially prepare either *trans* or *cis* ring substituted products.

Studies on the formation of *cis* and *trans* **3** from *E*‐ and *Z*‐isomers of starting material showed that the *E*‐alkene reacted stereoselectively to give the *trans* product while the *Z*‐alkene displayed variable levels of stereoselectivity (see Supporting Information). This means that the use of the allyl silanes remains the best and most stereoselective route to these nitrogen heterocycles.

In order to rationalise the reversal in stereochemistry observed with allyl silanes compared to styrene derivatives, a set of transition states are proposed (Scheme 3C; the addition is portrayed as concerted) which suggest the steric bulk of the silicon group plays in a key role dictating in the selectivity. For the parent aryl system (e.g. **1** **c**) a *trans* addition of the nitrogen nucleophile and cation electrophile across the alkene leads to the C2,3‐*trans* diastereoisomer. However, an analogous transition state for the allyl silane (e.g. **1** **aa**) would lead to a steric clash between the silyl group and the incoming electrophile (assuming that the silicon lies *syn* periplanar to the p‐orbitals of the alkene bond in order to maximise hyperconjugation). Consequently, addition of the cation from the face of the alkene anti to the silyl group would minimise steric interactions and maxmise stereoelectronic effects while leading to the *cis* heterocyclic product, as observed.

Finally, the inclusion of substituents on the alkene backbone allowed for the formation of more complex heterocyclic scaffolds, with up to four contiguous stereocentres, accessible in a single step. When a phenyl group was introduced in the 4‐position, moderate to high C3–C4 *trans* diastereoselectivity was coupled with complete C2–C3 *trans* selectivity and excellent acyclic stereoselectivity (Scheme 4, **3** **ad**–**3** **af**). Additionally, this route allowed us the opportunity to use enantiopure starting material to produce enantiopure products with complete enantioretention.

**Scheme 4:**
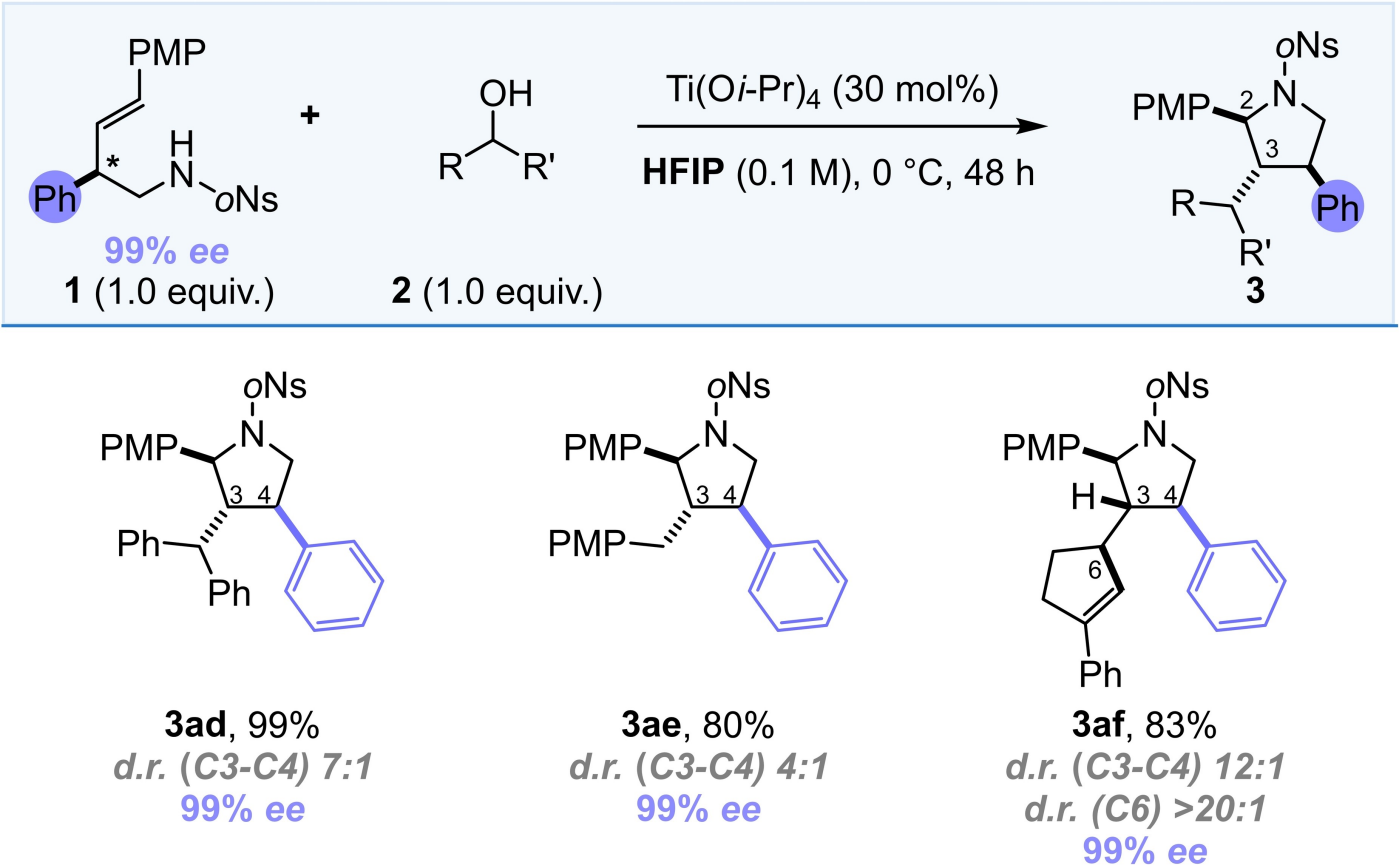
Substrate scope of enantiopure products. ^†^ *d.r. (C2–C3) >20 : 1*.

Despite several literature reports of carboamination reactions to form pyrrolidines, comparatively few examples which prepare piperidines have been published. With the newly developed methodology in hand, extension to the synthesis of piperidines proved to be challenging. Our efforts to improve reaction efficiency using the previous conditions for making pyrrolidines were unsuccessful and cyclisation of the homologue of **1** **c** was low yielding. Ultimately, we found that re‐optimisation of the *N*‐protecting group was required to enable this more demanding cyclisation to proceed with comparable yield and diastereoselectivity to the pyrrolidine system. A screen of various *N*‐protecting groups, again utilising benzhydrol as the electrophile, revealed that carbamates were superior to sulfonamides in this case, with a methyl carbamate group giving the best yield of the six membered heterocycle (for details, see Supporting Information). Consequently, a series of methyl carbamate protected bishomoallylic amines were then subjected to a full electrophile scope (Scheme 5).

**Scheme 5:**
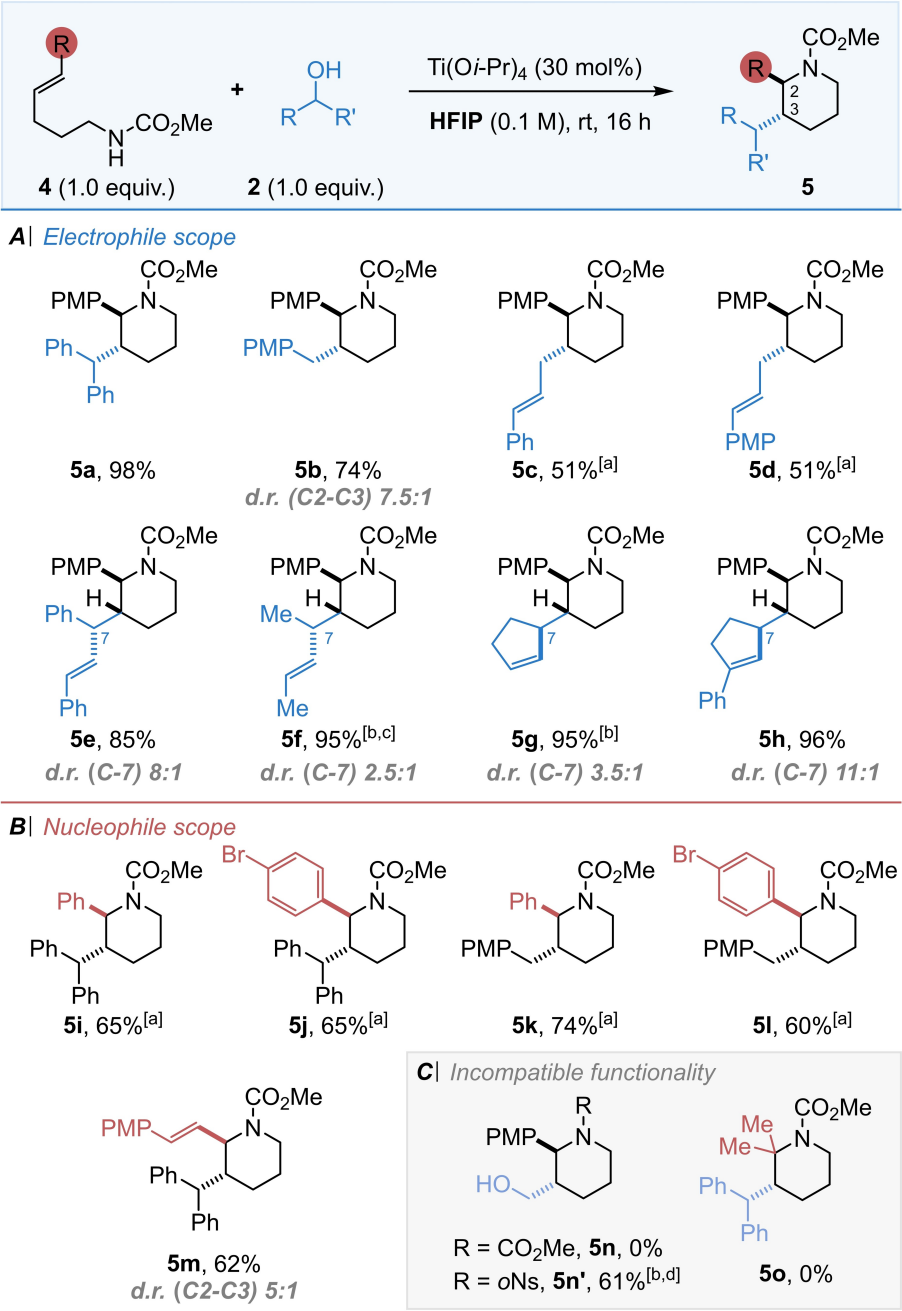
(A) Substrate scope of the electrophilic partner with piperidines. (B) Substrate scope of the nucleophilic partner with piperidines. ^[a]^ Performed at 70 °C. ^[b]^ Using 2.0 equiv. of electrophilic partner. ^[c]^ Performed at 0 °C for 48 h. ^[d]^ Using 20 equiv. of electrophilic partner. ^†^ *d.r. (C2–C3) >20 : 1* unless otherwise stated.

Pleasingly, this work gave comparable results to the pyrrolidine system; only in one case was exclusive 2,3‐*trans* selectivity not observed, when the benzylic alcohol **2** **b** afforded **5** **b** with a good 7.5 : 1 diastereomeric ratio. Note that assignment of the piperidine relative stereochemistry was challenging by NMR spectroscopy, and the stereochemistry was best confirmed through single crystal X‐ray diffraction data. A crystalline derivative of an N−Ns piperidine made via this methodology was prepared (**5iNs**,^[13]^ see Supporting Information), which confirmed the *trans* stereochemistry, and also showed the 2,3‐subsitutents adopting a diaxial conformation, presumably to minimise interaction with the *N*‐protecting group. All relative stereochemistry in this series was made by analogy with this example, in addition to a single‐crystal X‐ray diffraction structure of a derivative of **5** **c** (see below). While we found that paraformaldehyde electrophile was incompatible with the carbamate protecting group, surprisingly, the original *ortho‐*nosyl group retained high diastereoselectivity and moderate yield in this case only (Scheme 5, **5** **n**′). In this six‐membered ring series, changes to the nucleophile partner were more detrimental to the reaction efficiency, again showing the more challenging nature of the cyclisation. While variation in the aryl group was possible (Scheme 5, **5** **i**–**5** **l**), attempts to replace the alkene aryl substituent with alkyl groups was unsuccessful and no reaction occurred (Scheme 5, **5** **o**). However, the introduction of a diene precursor was successful once more, forming vinyl‐substituted piperidine **5** **m** with reasonable yield and diastereoselectivity.

With an extensive reaction scope established we were also keen to further derivatise the heterocyclic products in order to highlight their rich potential as intermediates in the preparation of medicinally relevant azacycles. Reaction conditions were established for the removal of both the *ortho*‐nosyl and methyl carbamate protecting groups, affording the corresponding free amine (Scheme 6A) or amine salt (Scheme 6B) in high yields.[^15^, ^16^] A single crystal X‐ray diffraction structure of derivative **7** **c** confirmed the relative stereochemistry for the six ring series.^[13]^ Derivatisation at the C‐2 position was also carried out; the PMP group could be selectively converted to a useful ester **8** via Ru‐catalysed oxidation (followed by ester formation), even in the presence of other aromatic groups (Scheme 6C).^[17]^ Moreover, both vinyl or silyl groups at C2 could be transformed into alcohol functionality via ozonolysis (followed by reductive quench) or Fleming‐Tamao oxidation respectively (Scheme 6C).^[18]^ It is particularly noteworthy that these transformations mean that the two diastereoisomers of alcohol products *trans*‐**9** or *cis*‐**9** can be produced at will by using either the vinyl or silyl substituted alkenes as starting materials for the cyclisation.

**Scheme 6:**
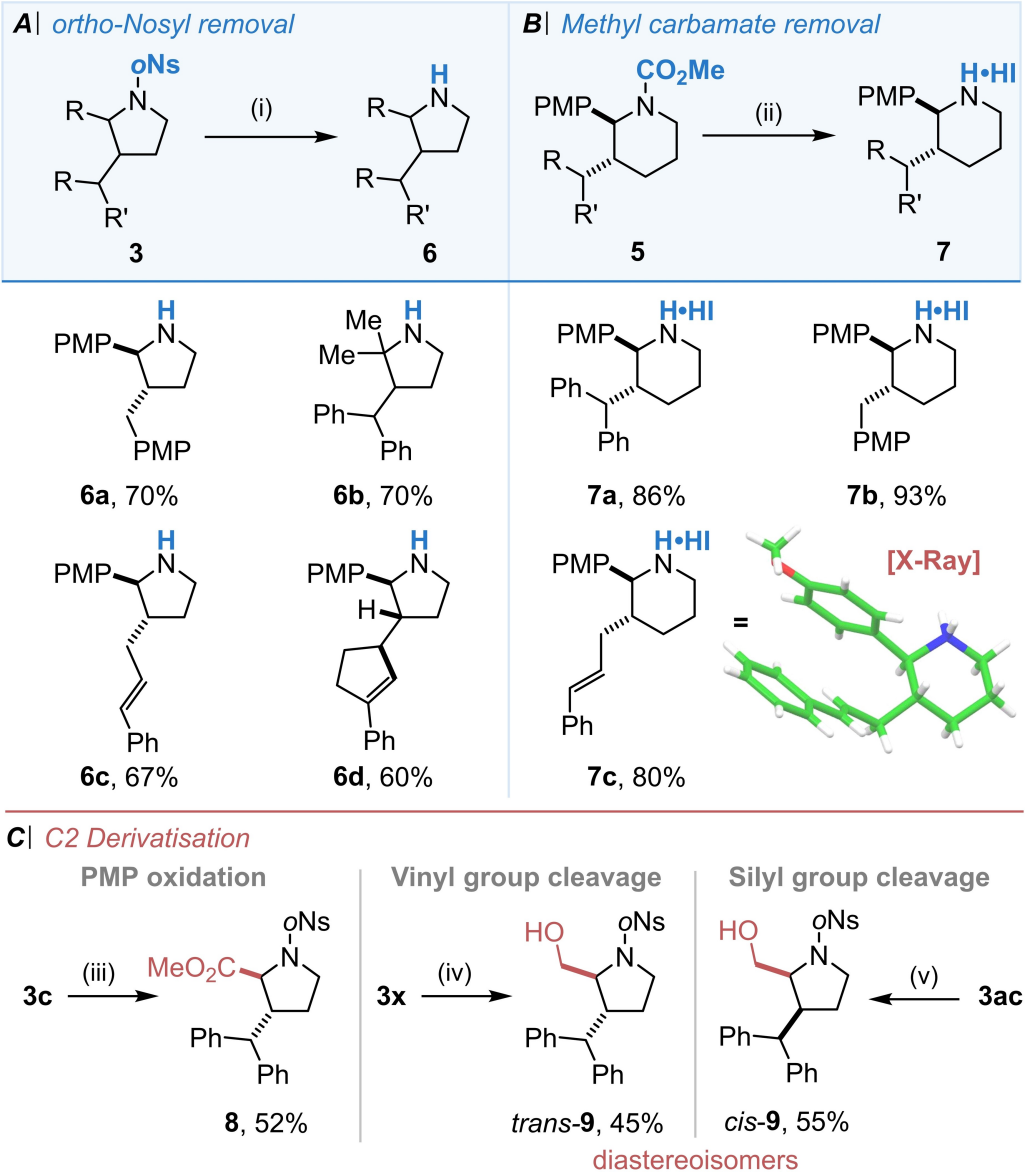
Product derivatisation. A) *ortho*‐Nosyl removal. B) Methyl carbamate removal. C) C2 group derivatisation. i) Thioglycolic acid (2.0 equiv), LiOH (4.0 equiv), DMF, rt; ii) TMS‐I (3.0 equiv), CHCl_3_, 55 °C; iii) a) RuCl_3_ (5 mol %), NaIO_4_, Na_2_HPO_4_:MeCN:CCl_4_; b) TMS‐CHN_2_, MeOH:PhMe; iv) O_3_, CH_2_Cl_2_/MeOH, −78 °C, NaBH_4_, −78 °C; v) BF_3_ ⋅ 2AcOH, CH_2_Cl_2_, 40 °C; KF (3.0 equiv), H_2_O_2_, NaHCO_3_, THF/MeOH, 70 °C.

In summary, we have outlined a useful and efficient Ti(O*i*‐Pr)_4_ promoted stereoselective synthesis of a diverse range of pyrrolidines and piperidines from readily available amine and alcohol components. Using HFIP as a solvent to encourage the formation of a reactive electrophile, not only alcohols but also paraformaldehyde can be utilised as alkylating agents to trigger a 5‐ or 6‐*endo*‐trig cyclisation. Moreover, a key breakthrough is the selective synthesis of either 2,3‐*trans* or 2,3‐*cis* pyrrolidine diastereoisomers by varying the substituent on the nucleophilic alkene partner. Finally, easy access to enantiopure products via use of enantiopure alkene starting materials and also a diverse derivatisation of the products reveal the rich potential of this methodology in synthetic organic chemistry.

## Conflict of interest

The authors declare no conflict of interest.

## Supporting information

As a service to our authors and readers, this journal provides supporting information supplied by the authors. Such materials are peer reviewed and may be re‐organized for online delivery, but are not copy‐edited or typeset. Technical support issues arising from supporting information (other than missing files) should be addressed to the authors.

Supporting Information

## Data Availability

The data that support the findings of this study are available in the supplementary material of this article.
