# Supplementary material for: Alcohols as Alkylating Agents in the Cation‐Induced Formation of Nitrogen Heterocycles
Source: Angew Chem Int Ed Engl. 2022 Jul 13;61(34):e202206800. doi: 10.1002/anie.202206800 (PMC9546487; doi:10.1002/anie.202206800)
Supplement: Supplementary file 1 — Supporting Information [file ANIE-61-0-s001.pdf]

Angewandte Chemie
Eine Zeitschrift der Gesellschaft Deutscher Chemiker

Supporting Information

Alcohols as Alkylating Agents in the Cation-Induced Formation of Nitrogen Heterocycles

*L. Cox, Y. Zhu, P. J. Smith, K. E. Christensen, M. Sidera Portela, T. J. Donohoe**

Table of Contents

General Experimental Details 3

Experimental Procedures and Data 5

General Procedures 5

Extended Optimisation Tables 9

Experimental Procedures 11

Preparation of Nucleophiles 11

Preparation of Electrophiles 39

Cyclisations 43

Product Derivatisations 87

Use of Tertiary Alcohols 95

Unreactive Nucleophiles 96

Supporting document for mechanistic hypothesis 97

Supporting document for structural assignment of piperidines 98

Crystallography 99

Copies of ^{1}H & ^{13}C NMR spectra for novel compounds 102

References 200

General Experimental Details

Reagents, solvents and techniques: All reactions were performed under an inert atmosphere with constant magnetic stirring, unless otherwise stated, using clean, oven dried glassware. Reagents and solvents were obtained from commercial supplies and were used without further purification. All inert gases were sourced from the University of Oxford's internal supplies and dried through $CaCl_2$ drying columns. Anhydrous solvents were obtained from the University of Oxford internal solvent system and dried by passing through alumina columns, manufactured by Innovative Technology Inc. PS-400-7. Temperatures of −78 °C and 0 °C were achieved with a CO_2(s)/acetone bath and ice/water bath respectively. Where a reaction was required to stay at 0 °C for 48 h a Thermo Scientific EK90 Immersion Cooler with Flex Probe and isopropanol bath was used.

Chromatography: Reactions were monitored by TLC using aluminium backed silica gel plates (Merck Kieselgel 60 PF254) with the appropriate solvent system and were visualised using UV fluorescence (254 nm) and developed with potassium permanganate, phosphomolybdic acid or vanillin. Flash column chromatography was performed on silica gel (60 Å, 230-400 mesh) under a positive pressure of nitrogen and the required eluent.

NMR Spectroscopy: ^{1}H and ^{13}C NMR samples were run on a Bruker AVIIIHD 400 MHz spectrometer, where required COSY and HSQC spectra were used to assign NMR spectra. In addition, Bruker AVIIIHD 500 MHz and Bruker NEO 600 MHz spectrometers were used to obtain NOESY and HMBC data where required. All NMR spectra were recorded at ambient temperature. Chemical shifts (δ) are recorded in parts per million (ppm). The residual protic solvent signal(s) acted as the internal reference for ^{1}H NMR spectra [$CDCl_3$ (δ 7.26) or MeOH-d4 (δ 3.31)], and the deuterated solvent signal acted as the internal reference for ^{13}C NMR spectra [$CDCl_3$ (δ 77.16), MeOH-d4 (δ 49.0)]. All diastereomeric ratio values were based on the crude NMR unless otherwise stated.

Mass Spectroscopy: Mass spectra were obtained through the University of Oxford mass spectrometry service by electrospray ionisation (ESI) using a Bruker Daltonics microTOF spectrometer. The m/z values were all recorded in Daltons to four decimal places, the mass found was compared to the mass calculated from the monoisotopic molecular formula.

Infrared Spectroscopy: Infrared spectra were obtained using a Bruker Tensor 27 FT-IR spectrometer equipped with a diamond ATR module. Absorption maxima (ν_{max}) are quoted in wavenumbers (cm^{-1}).

Melting Points: Melting points were obtained using Griffin melting point apparatus.

Optical Rotations: Optical rotations were recorded on a Schmidt Haensch Unipol L2000 polarimeter in a cell with a path length of 1 dm (using the sodium D line, 589 nm). Concentrations are reported in g/100 mL. Temperatures are reported in °C.

Chiral normal phase HPLC: Chiral normal phase HPLC was performed on an Agilent 1260 Series HPLC unit equipped with UV-vis diode-array detector, fitted with the appropriate Daicel Chiralpak column (dimensions: 0.46 cm ø × 25 cm) along with the corresponding guard column (0.4 cm ø × 1 cm). Wavelengths (λ) are reported in nm, retention times (tR) are reported in minutes and solvent flow rates are reported in mL min^{-1}.

Experimental Procedures and Data

General Procedures

General Procedure A: Wittig

According to the procedure of *Maryanoff.*[1] To a solution of (3-carboxypropyl)triphenyl-phosphonium bromide (1.2 equiv.) in anhydrous THF (2.0 mL/mmol) was added NaHMDS (2.0 M in THF, 2.4 equiv.) dropwise at 0 °C and left to stir for 30 min. The solution was cooled to –78 °C before addition of aldehyde (1.0 equiv.) dropwise. The reaction mixture was left to warm to rt over 16 h. The solution was diluted with EtOAc and water and the layers separated. The aqueous layer was acidified to pH 1 with 1.0 M HCl before extracting with EtOAc. The combined organic layers were washed with 1.0 M HCl and dried over $MgSO_4$ before concentrating *in vacuo* and purifying by FCC.

General Procedure B: Curtius rearrangement and amine protection sequence

To a solution of acid (1.0 equiv.) in PhMe (4.0 mL/mmol) was added Et_3N (1.5 equiv.) followed by dropwise addition of diphenylphosphoryl azide (DPPA) (1.5 equiv.) before heating to 85 °C for 2 h. The solution was cooled to rt before the addition of THF (4.0 mL/mmol) and aq. NaOH (2.0 M, 2 mL/mmol) and the reaction was left to stir vigorously for 16 h. The solution was diluted with EtOAc and water and the layers separated. The aqueous layer was extracted with EtOAc and the combined organic layers were washed with 1.0 M NaOH and dried over $MgSO_4$ before concentrating *in vacuo*. The crude amine was re-dissolved in CH_2Cl_2 (5.0 mL/mmol) and Et_3N added (1.25 equiv.). The solution was cooled to 0 °C before addition of the appropriate sulfonamide (as a solution) or chloroformate (1.25 equiv.). The reaction mixture was left to warm up to rt and stirred for 16 h. The reaction mixture was diluted with CH_2Cl_2 and water. The aqueous later was extracted with CH_2Cl_2 and the combined organic layers were washed with brine and dried over $MgSO_4$ before concentrating *in vacuo* and purifying by FCC.

General Procedure C: Ru-catalysed metathesis

To a solution of the appropriate Ru catalyst (1.5 mol%) in anhydrous solvent (4 mL/mmol, previously degassed by bubbling through Ar for 30 min.) was added the alkene components in solution (1.0 mL/mmol) and the mixture heated at reflux. The reaction was monitored by TLC until completion, before the solvent was removed *in vacuo* and the crude material purified by FCC.

General Procedure D: Mitsunobu reaction

To a solution of alcohol (1.0 equiv.), Ph_3P (1.5 equiv.) and nucleophile (1.2 equiv.) in THF (3.0 mL/mmol) was added diisopropyl azodicarboxylate (1.3 equiv.) dropwise at 0 °C before allowing to warm to rt. After 16 h the solvent was removed *in vacuo* before purifying by FCC.

General Procedure E: Boc deprotection

To a solution of carbamate (1.0 equiv.) in CH_2Cl_2 (3.0 mL/mmol) was added trifluoroacetic acid (5.0 equiv.) dropwise at 0 °C. The reaction was monitored by TLC until completion before addition of aq. $NaHCO_3$. The aqueous later was extracted with CH_2Cl_2 and the combined organic layers were washed with brine and dried over $MgSO_4$ before concentrating *in vacuo* and using crude in the following reaction.

General Procedure F: Carboxylic acid reduction

To a suspension of $LiAlH_4$ (1.2 equiv.) in anhydrous THF (1.0 mL/mmol) was added a solution of acid (1.0 equiv.) in THF dropwise at 0 °C and left to stir for 3 h. The solution was quenched using a Fieser workup before concentrating *in vacuo* and purifying by FCC.

General Procedure G: Mesylate formation

To a solution of alcohol (1.0 equiv.) in anhydrous CH_2Cl_2 (5.0 mL/mmol) was added triethylamine (1.5 equiv.) before cooling to 0 °C. Methanesulfonic anhydride (1.2 equiv.) was added portionwise before warming to rt. The reaction mixture was left to stir for 3 h before addition of 0.5 M HCl (4.5 mL/mmol). The aqueous later was extracted with CH_2Cl_2 and the combined organic layers were washed with brine, dried over $MgSO_4$ before concentrating *in vacuo* and purifying by FCC.

General Procedure H: S_N2

A suspension of sulfonamide (1.5 equiv.) and potassium hydroxide (1.5 equiv.) in DMF (10 mL/mmol) was stirred at 100 °C for 30 min before dropwise addition of mesylate (1.0 equiv.) in DMF. The reaction mixture was left to stir at 100 °C for 1 h before cooling to rt and diluting with water. The aqueous layer was extracted with Et_2O and the organic layer washed with water and brine before drying over Na_2SO_4, concentrating *in vacuo* and purifying by FCC.

General Procedure I: Amide formation

To a solution of carboxylic acid (1.0 equiv.) in anhydrous CH_2Cl_2 (2 mL/mmol) was added oxalyl chloride (1.2 equiv.) dropwise at 0 °C followed by DMF (2 drops) and left to warm up to rt. After 45 min, 80% of the solvent was removed in vacuo before re-dissolving in CH_2Cl_2 (2 mL/mmol). Aq. ammonia (28% in H_2O, 4.0 mL/mmol) was added portionwise at 0 °C and left to warm up to rt and stirred for 16 h. The aqueous later was extracted with CH_2Cl_2 and the combined organic layers were washed with brine and dried over $MgSO_4$ before concentrating *in vacuo*. The crude amide was recrystallised from hot EtOAc.

General Procedure J: Amide reduction and protection sequence

To a solution of amide (1.0 equiv.) in anhydrous THF (3.0 mL/mmol) was added $LiAlH_4$ (1.0 M in THF, 1.5 equiv.) dropwise at 0 °C and left to warm up to rt and stirred for 16 h. The solution was diluted with Et_2O and cooled to 0 °C before subsequent addition of H_2O (1.0 mL/g of $LiAlH_4$), aq. NaOH (15 wt%, 1.0 mL/g of $LiAlH_4$) and H_2O (3.0 mL/g of $LiAlH_4$). The solution was warmed to rt and stirred for 15 min before addition of anhydrous $MgSO_4$, after a further 15 min the solid was removed by filtration before concentrating *in vacuo*. The crude amine was used directly in the following protection reaction. To a solution of amine (1.0 equiv.) in anhydrous CH_2Cl_2 (5.0 mL/mmol) was added triethylamine (1.25 equiv.). The solution was cooled to 0 °C before addition of the appropriate sulfonamide (as a solution) or chloroformate (1.25 equiv.). The reaction mixture was left to warm up to rt and stirred for 16 h. The reaction mixture was diluted with CH_2Cl_2 and water. The aqueous later was extracted with CH_2Cl_2 and the combined organic layers were washed with brine and dried over $MgSO_4$ before concentrating *in vacuo* and purifying by FCC.

Modification for low molecular weight amines: Following the Fieser workup, before concentrating in vacuo, the solution was cooled to 0 °C and HCl (2.0 M in Et_2O, 1.25 equiv.) was added dropwise and stirred for 15 min. The hydrochloride salt of the amine was then used directly in the following protection reaction. To a solution of hydrochloride salt of the amine (1.0 equiv.) in anhydrous CH_2Cl_2 (5.0 mL/mmol) at 0 °C was added triethylamine (3.0 equiv.) dropwise and left to stir for 30 min before addition of methylchloroformate (1.25 equiv.) dropwise. The normal procedure was then followed.

General Procedure K: Cyclisation

To a microwave vial charged with a stirrer bar was added the appropriately protected amine (1.0 equiv.), alcohol (1.0 equiv.) and HFIP (10 mL/mmol) under argon. A solution of $Ti(O^iPr)_4$ in HFIP (30 mol%) was added and the reaction stirred at the given temperature for the given period of time. The reaction mixture was concentrated *in vacuo* and purified by FCC.

General Procedure L: *ortho*-Ns removal

To a solution of pyrrolidine (1.0 equiv.) in anhydrous DMF (1.0 mL/mmol) under Ar was added LiOH (4.0 equiv.) and mercaptoacetic acid (2.0 equiv.) before stirring at rt for 16 h. The solution was diluted with EtOAc and water and the layers separated. The aqueous layer was extracted with EtOAc and the combined organic layers were washed with brine and dried over Na_2SO_4 before concentrating *in vacuo* and purifying by FCC.

General Procedure M: Methyl carbamate removal

To a solution of cyclised product (1.0 equiv.) in $CHCl_3$ (25 mL/mmol) was added trimethylsilyl iodide (3.0 equiv.) before heating to 55 °C for 2 h. The reaction mixture was concentrated *in vacuo* and purified by FCC.

Modification for piperidines containing alkenes: Isoprene (10.0 equiv.) was added before heating to 55 °C.

Extended Optimisation Tables

Additional condition screening

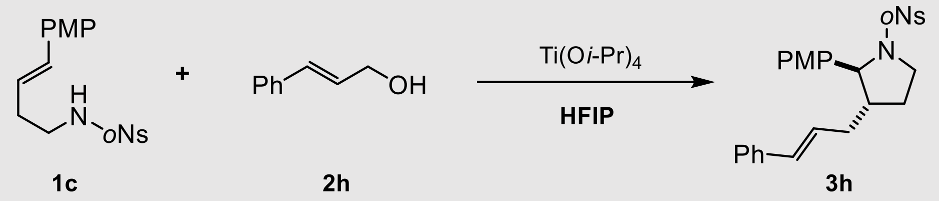

Entry	**1c**:**2h**	Ti(O^{i}Pr)$_4$ / mol%	Temp / ˚C	Time / h	Concentration/ M	Yield **3h** / %	Recovered / % **1c**	Recovered / % **2h**
1	1:1	30	70	16	0.1	58	42	0
2	1:1	20	70	16	0.1	43	-	-
3	1:1	10	70	16	0.1	38	-	-
4	1:2	30	70	16	0.1	40	37	37
5	1:1	30	40	16	0.1	31	68	35
6	1:1	30	70	16	0.05	54	46	0
7	1:1	30	40	36	0.1	40	60	5
8	1:2	30	70	36	0.1	43	57	33
9	1:2	30	95	16	0.1	52	48	33
10	1:2	50	70	16	0.1	58	41	0
11[†]	1:2	50	70	16	0.1	43	57	5

[†]1 equiv. of **2h** was added after 6 h.

HFIP source

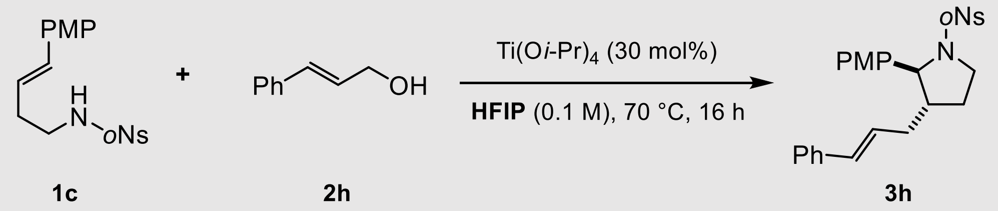

Entry	**HFIP** source	Yield **3h** / %	Recovered **1c** / %	Recovered **2h** / %
1	Commercial bottle	58	42	0
2	Commercial bottle with additional 20 equiv. H_2O	58	42	0
3	Distilled	57	42	0
4	Degassed	58	42	0
5	Distilled and degassed	58	42	0

Piperidine protecting group screen

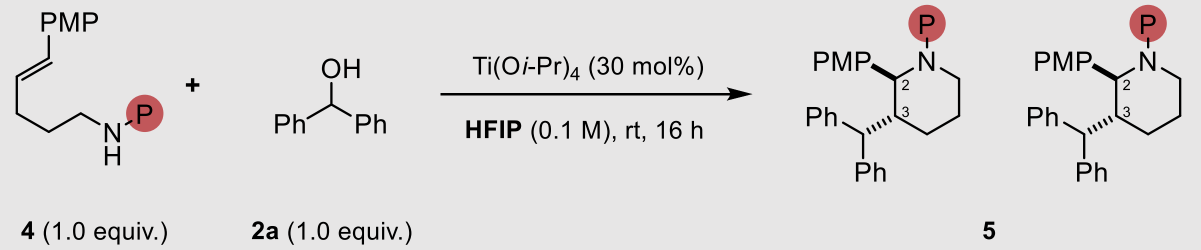

P =	Ts	Ms	*o*Ns	*p*Ns	Cbz	CO_2Me
Yield **5** / %	95	64	84	88	86	98
d.r (C2-C3)	5:1	2:1	1:2	3:1	>20:1	>20:1

Experimental Procedures

Preparation of Nucleophiles

(S1): (*E*)-5-(4-Methoxyphenyl)pent-4-enoic acid

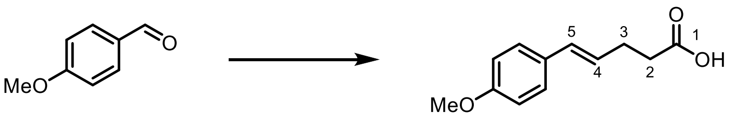

S1

Prepared by **General Procedure A** using 4-methoxybenzaldehyde (1.22 mL, 10.0 mmol), (3-carboxypropyl)triphenylphosphonium bromide (5.15 g, 12.0 mmol), NaHMDS (2.0 M in THF, 12.0 mL, 24.0 mmol). Flash column chromatography (50% Et_2O – pentane + 1% acetic acid) afforded **S1** as a crystalline white solid (1.65 g, 80%).

Data for **S1**: **R_f** 0.54 (20% EtOAc – pentane). **^{1}H NMR (400 MHz, $CDCl_3$)** 7.27 (2H, d, *J* = 8.5 Hz, Ar), 6.84 (2H, d, *J* = 8.8 Hz, Ar), 6.39 (1H, d, *J* = 15.8 Hz, C5-H), 6.07 (1H, dt, *J* = 15.8, 7.0 Hz, C4-H), 3.80 (3H, s, OMe), 2.69 – 2.45 (4H, m, 2 × C2-H_2 + 2 × C3-H_2). **^{13}C NMR (101 MHz, $CDCl_3$) δ** 159.2 (C Ar), 130.7 (C5), 130.3 (C Ar), 127.4 (C4), 126.0 (2 × C Ar), 114.1 (2 × C Ar), 55.5 (OMe), 33.9 (C2), 28.1 (C3). *The spectroscopic properties were consistent with the data available in the literature.*[1]

(1a): (3*E*)-*N*-[4-(4-Methoxyphenyl)but-3-en-1-yl]-4-methylbenzenesulfonamide

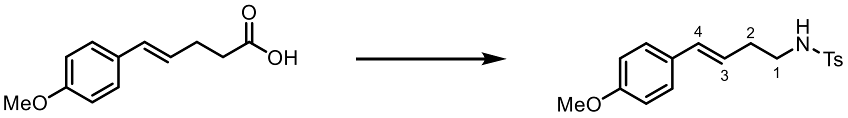

S1 **1a**

Prepared by **General Procedure B** using **S1** (1.00 g, 4.85 mmol), Et_3N (1.00 mL, 7.28 mmol) and DPPA (1.50 mL, 7.28 mmol). Crude amine was then subjected to Et_3N (0.84 mL, 6.08 mmol) and 4-toluenesulfonyl chloride (1.16 g, 6.08 mmol). Flash column chromatography (25% EtOAc – pentane) afforded **1a** as a white solid (1.08 g, 67%).

Data for **1a**: **R_f** 0.50 (70% Et_2O – pentane). **^{1}H NMR (400 MHz, $CDCl_3$) δ** 7.74 (2H, d, *J* = 8.7 Hz, Ar), 7.29 (2H, d, *J* = 8.7 Hz, Ar), 7.21 (2H, d, *J* = 8.7 Hz, Ar), 6.83 (2H, d, *J* = 8.7 Hz, Ar), 6.30 (1H, d, *J* = 15.8 Hz, C4-H), 5.82 (1H, dt, *J* = 15.8, 7.1 Hz, C3-H), 4.41 (1H, t, *J* = 6.5 Hz, *N*H), 3.81 (3H, s, OMe), 3.09 (2H, q, *J* = 6.5 Hz, C1-H_2), 2.43 (3H, s, TsMe), 2.34 (2H, qd, *J* = 6.5, 1.4 Hz, C2-H_2). **^{13}C NMR (100 MHz, $CDCl_3$) δ** 159.3 (2 × C Ar), 143.6 (2 × C Ar), 132.9 (C4), 129.9 (2 × C Ar), 127.4 (2 × C Ar), 127.3 (2 × C Ar), 123.3 (C3), 114.1 (2 × C Ar), 55.5 (OMe), 42.8 (C1), 33.1 (C-2), 21.7 (TsMe). *The spectroscopic properties were consistent with the data available in the literature.*[2]

(1b): (*E*)-*N*-(4-(4-Methoxyphenyl)but-3-en-1-yl)methanesulfonamide

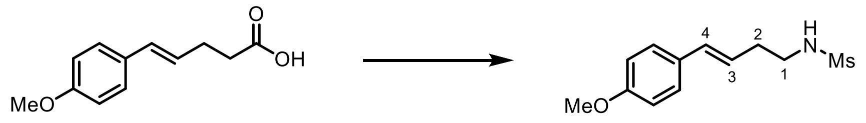

S1 **1b**

Prepared by **General Procedure B** using **S1** (500 mg, 2.43 mmol), Et_3N (0.50 mL, 3.6 mmol) and DPPA (0.78 mL, 3.6 mmol). Crude amine was then subjected to Et_3N (0.420 mL, 3.04 mmol) and methanesulfonyl chloride (0.24 mL, 3.04 mmol). Flash column chromatography (25% EtOAc – pentane) afforded **1b** as a white solid (382 mg, 56 %).

Data for **1b**: $\mathbf{R_f}$ 0.50 (60% EtOAc – pentane). **^{1}H NMR (400 MHz, $CDCl_3$) δ** 7.28 (2H, d, *J* = 8.7 Hz, Ar), 6.85 (2H, d, *J* = 8.7 Hz, Ar), 6.43 (1H, d, *J* = 15.8 Hz, C4-H), 5.97 (1H, dt, *J* = 15.8, 7.1 Hz, C3-H), 4.50 (1H, t, *J* = 6.5 Hz, *N*H), 3.80 (3H, s, OMe), 3.09 (2H, q, *J* = 6.5 Hz, C1-H_2), 2.94 (3H, s, SO_2Me), 2.46 (2H, qd, *J* = 6.5, 1.4 Hz, C2-H_2). **^{13}C NMR (100 MHz, $CDCl_3$) δ** 159.3 (C Ar), 132.9 (C4), 129.7 (C Ar), 127.4 (2 × C Ar), 123.3 (C3), 114.1 (2 × C Ar), 55.4 (OMe), 42.9 (C1), 40.5 (SO_2Me), 33.7 (C2). **HRMS** (ESI): calculated for $C_{12}H_{17}NO_3SNa$ $[M+Na]^+$ requires *m/z* 278.0827, found *m/z* 278.0821. **IR** (film) ν_{max} : 3283, 2980, 2360, 1576, 1511, 1313 cm^{-1}. **mp** (97 – 99 °C, EtOAc).

(1c): (3*E*)-*N*-[4-(4-Methoxyphenyl)but-3-en-1-yl]-2-nitrobenzenesulfonamide

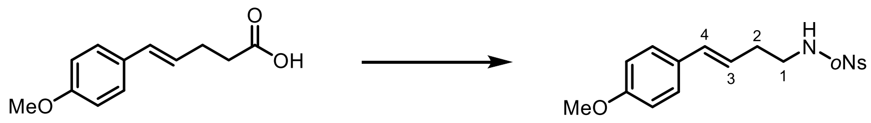

S1 **1c**

Prepared by **General Procedure B** using **S1** (1.00 g, 4.85 mmol), Et_3N (1.00 mL, 7.28 mmol) and DPPA (1.50 mL, 7.28 mmol). Crude amine was then subjected to Et_3N (0.84 mL, 6.08 mmol) and 2-nitrobenzenesulfonyl chloride (1.35 g, 6.08 mmol). Flash column chromatography (25% EtOAc – pentane) afforded **1c** as a white solid (1.30 mg, 68%).

Data for **1c**: $\mathbf{R_f}$ 0.22 (30% EtOAc – pentane). **^{1}H NMR (400 MHz, $CDCl_3$) δ** 8.15 – 8.11 (1H, m, Ar), 7.79 – 7.63 (3H, m, Ar), 7.17 (2H, d, *J* = 8.6 Hz, Ar), 6.81 (2H, d, *J* = 8.8 Hz, Ar), 6.29 (1H, dt, *J* = 15.8, 1.5 Hz, C4-H), 5.82 (1H, dt, *J* = 15.8, 7.1 Hz, C3-H), 5.39 (1H, t, *J* = 5.9 Hz, *N*H), 3.80 (3H, s, OMe), 3.27 (2H, td, *J* = 6.7, 5.9 Hz, C1-H_2), 2.40 (2H, qd, *J* = 6.7, 1.4 Hz, C2-H_2). **^{13}C NMR (101 MHz, $CDCl_3$) δ** 159.3 (C Ar), 134.0 (C Ar), 133.5 (C Ar), 133.0 (C Ar), 132.9 (C4), 131.1 (C Ar), 129.6 (C Ar), 127.4 (2 × C Ar), 125.5 (C Ar), 122.9 (C3), 114.1 (2 × C Ar), 55.4 (OMe), 43.7 (C1), 33.3 (C2). **HRMS** (ESI): calculated for $C_{17}H_{18}N_2O_5SNa$ $[M+Na]^+$ requires *m/z* 385.0829, found *m/z* 385.0826. **IR** (film) ν_{max} : 2981, 2360, 1521, 1441, 1342, 1126, 969 cm^{-1}. **mp** (111 – 113 °C, EtOAc).

(1d): (3*E*)-*N*-[4-(4-Methoxyphenyl)but-3-en-1-yl]-4-nitrobenzenesulfonamide

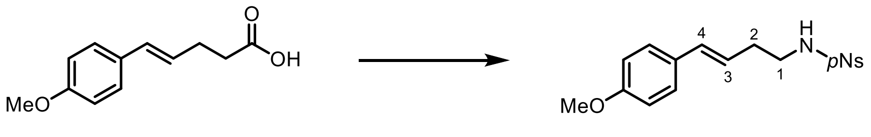

S1 **1d**

Prepared by **General Procedure B** using **S1** (1.00 g, 4.85 mmol), Et_3N (1.00 mL, 7.28 mmol) and DPPA (1.50 mL, 7.28 mmol). Crude amine was then subjected to Et_3N (0.84 mL, 6.08 mmol) and 4-nitrobenzenesulfonyl chloride (1.35 g, 6.08 mmol). Flash column chromatography (25% EtOAc – pentane) afforded **1d** as a white solid (1.35 mg, 71%).

Data for **1d**: **R_f** 0.23 (30% EtOAc – pentane). **^{1}H NMR (400 MHz, $CDCl_3$) δ** 8.28 (2H, d, *J* = 8.8 Hz, Ar), 8.01 (2H, d, *J* = 8.8 Hz, Ar), 7.17 (2H, d, *J* = 8.8 Hz, Ar), 6.82 (2H, d, *J* = 8.8 Hz, Ar), 6.30 (1H, d, *J* = 15.8 Hz, C4-H), 5.76 (1H, dt, *J* = 15.8, 7.2 Hz, C3-H), 4.70 (1H, t, *J* = 6.0 Hz, *N*H), 3.18 (2H, q, *J* = 6.3 Hz, C1-H_2), 2.37 (tdd, *J* = 7.6, 6.5, 1.4 Hz, C2-H_2). **^{13}C NMR (101 MHz, $CDCl_3$) δ** 159.5 (C Ar), 150.1 (C Ar), 146.1 (C Ar), 133.3 (C4), 129.4 (C Ar), 128.4 (2 × C Ar), 127.4 (2 × C Ar), 124.5 (2 × C Ar), 122.7 (C3), 114.2 (2 × C Ar), 55.5 (OMe), 43.0 (C1), 33.3 (C2). **HRMS** (ESI): calculated for $C_{17}H_{18}N_2O_5SNa$ $[M+Na]^+$ requires *m/z* 385.0829, found *m/z* 385.0826. **IR** (film) ν_{max} : 3294, 1667, 1529, 1521, 1348, 1248, 1092, 918 cm^{-1}. **mp** (104 – 106 °C, EtOAc).

(1e): Benzyl (*E*)-(4-(4-methoxyphenyl)but-3-en-1-yl)carbamate

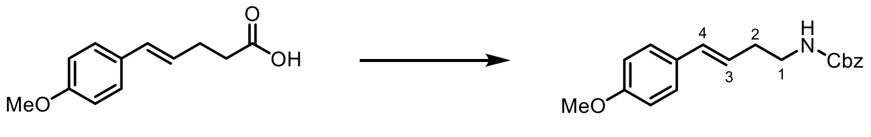

S1 **1e**

Prepared by **General Procedure B** using **S1** (500 mg, 2.43 mmol), Et_3N (0.50 mL, 3.6 mmol) and DPPA (0.78 mL, 3.6 mmol). Crude amine was then subjected to Et_3N (0.420 mL, 3.04 mmol) and benzyl chloroformate (0.43 mL, 3.6 mmol). Flash column chromatography (20% EtOAc – pentane) afforded **1e** as a white solid (614 mg, 75%).

Data for **1e**: **R_f** 0.28 (30% EtOAc – pentane). **^{1}H NMR (400 MHz, $CDCl_3$) δ** 7.38 – 7.30 (5H, m, Ar), 7.28 – 7.24 (2H, m, Ar), 6.84 (2H, d, *J* = 8.8 Hz, Ar), 6.39 (1H, d, *J* = 16.0 Hz, C4-H), 5.99 (1H, dt, *J* = 15.8, 7.1 Hz, C3-H), 5.10 (2H, s, CH_2Ph), 4.83 (1H, *br*.s, *N*H), 3.80 (3H, s, OMe), 3.34 (2H, q, *J* = 6.5 Hz, C1-H_2), 2.40 (2H, q, *J* = 6.8 Hz, C2-H_2). **^{13}C NMR (101 MHz, $CDCl_3$) δ** 159.1 (C Ar), 156.5 (CO), 136.7 (C Ar), 132.0 (C Ar), 130.1 (C Ar), 128.7 (2 × C Ar), 128.3 (C4), 128.2 (2 × C Ar), 127.4 (2 × C Ar), 124.5 (C3), 114.1 (2 × C Ar), 66.8 (CH_2Ph), 55.4 (OMe), 40.8 (C1), 33.4 (C2). **HRMS** (ESI): calculated for $C_{19}H_{21}NO_3Na$ $[M+Na]^+$ requires *m/z* 334.1414, found *m/z* 334.1411. **IR** (film) ν_{max} : 2981, 2360, 1713, 1511, 1247, 907, 730, 650 cm^{-1}. **mp** (117 – 119 °C, EtOAc).

(S2): *N*-Benzylbut-3-en-1-amine

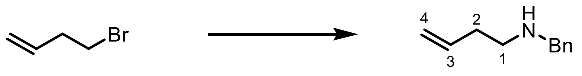

S2

To a solution of benzylamine (4.00 mL, 37.1 mmol, 5.00 equiv.) and 4-bromo-1-butene (0.75 mL, 7.4 mmol, 1.0 equiv.) in EtOH (5 mL) was added NaI (110 mg, 0.74 mmol, 0.10 equiv.), before heating to reflux for 4 h. The reaction mixture was cooled to rt and the solvent removed *in vacuo*. The residue was dissolved in CH_2Cl_2 and 1.0 M aq. KOH added. The aqueous later was extracted with CH_2Cl_2 and the combined organic layers were washed with brine and dried over $MgSO_4$ before concentrating *in vacuo*. Flash column chromatography (gradient elution 20% → 100% EtOAc – pentane) afforded **S2** as a colourless oil (780 mg, 65%).

Data for S2: **R_f** 0.20 (60% EtOAc – pentane). **^{1}H NMR (400 MHz, $CDCl_3$) δ** 7.29 – 7.34 (4H, m, Ar), 7.23 – 7.27 (1H, m, Ar), 5.79 (1H, ddt, *J* = 17.1, 10.2, 6.9 Hz, C3-H), 4.99 – 5.13 (2H, m, C4-H_2), 3.80 (2H, s, CH_2Ph), 2.71 (2H, t, *J* = 6.9 Hz, C1-H_2), 2.29 (2H, qt, J = 6.9, 1.4 Hz, C2-H_2). **^{13}C NMR (100 MHz, $CDCl_3$) δ** 140.5 (C Ar), 136.6 (C3), 128.5 (2 × C Ar), 128.2 (2 × C Ar), 127.0 (C Ar), 116.5 (C4), 54.1 (CH_2Ph), 48.4 (C1), 34.4 (C2). *The spectroscopic properties were consistent with the data available in the literature.*[3]

(1f): (3*E*)-*N*-Benzyl-4-(4-methoxyphenyl)but-3-en-1-amine

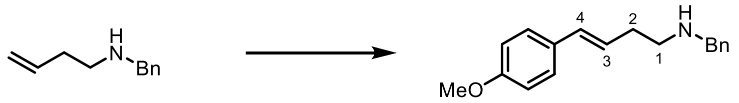

S2 **1f**

To a solution of **S2** (254 mg, 1.56 mmol, 1.00 equiv.) in Et_2O (1.70 mL) was added trifluoroacetic acid (0.24 mL, 1.7 mmol, 1.1 equiv.) dropwise at 0 ˚C. After stirring for 2 h at rt water was added and the layers separated. The aqueous later was extracted with Et_2O and the combined organic layers were washed with brine and dried over Na_2SO_4 before concentrating *in vacuo*. The crude material was then subjected to **General Procedure C** using 4-methoxystyrene (290 mg, 2.16 mmol) and Grubbs II (14 mg, 0.016 mmol) in PhMe at 100 ˚C. The solvent was removed *in vacuo* before being re-dissolved in MeOH (4 mL) and water (0.5 mL). KOH (123 mg, 2.16 mmol) was added and the reaction mixture heated to reflux for 2 h. The reaction mixture was diluted with EtOAc and water and the layers separated. The aqueous later was extracted with EtOAc and the combined organic layers were washed with brine and dried over Na_2SO_4 before concentrating *in vacuo*. Flash column chromatography (gradient elution 20% → 50% EtOAc – pentane) afforded **1f** as a colourless oil (130 mg, 43%).

Data for **1f**: **R_f** 0.50 (40% EtOAc – pentane). **^{1}H NMR (400 MHz, $CDCl_3$) δ** 7.22 – 7.37 (7H, m, Ar), 6.83 (2H, d, *J* = 8.7 Hz, Ar), 6.39 (1H, d, *J* = 15.8 Hz, C4-H), 6.02 (1H, dt, *J* = 15.8, 7.1 Hz, C3-H), 3.84 (2H, s, CH_2Ph), 3.80 (3H, s, OMe), 2.78 (2H, t, *J* = 6.9 Hz, C1-H_2), 2.45 (2H, qd, *J* = 7.0, 1.4 Hz, C2-H_2). **^{13}C NMR (100 MHz, $CDCl_3$) δ** 158.9 (C Ar), 139.2 (C Ar), 131.2 (C4), 130.3 (C Ar), 128.5 (2 × C Ar), 128.4 (2 × C Ar), 127.2 (C Ar), 127.1 (2 × C Ar), 125.5 (C3), 113.9 (2 × C Ar), 55.3 (OMe), 53.6 (CH_2Ph), 48.5 (C1), 33.1 (C2). **HRMS (ESI):** calculated for $C_{18}H_{22}NO$ $[M+H]^+$ requires m/z 268.1701, found m/z 268.1695. **IR** (film) ν_{max}: 2980, 1606, 1576, 1509, 1453, 1245 cm^{-1}.

(S3): (3*E*)-4-Phenylbut-3-en-1-yl acetate

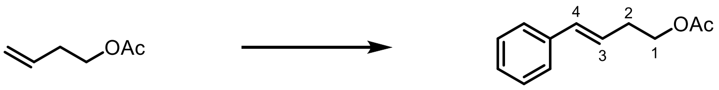

S3

Prepared according to **General Procedure C** using but-3-en-1-yl acetate (1.00 g, 8.76 mmol), styrene (456 mg, 4.38 mmol) and Grubbs II (56 mg, 0.066 mmol) at reflux in CH_2Cl_2. Flash column chromatography (5% → 8% Et_2O – pentane) afforded **S3** as a white solid (494 mg, 59%).

Data for **S3**: **R_f** 0.50 (20% Et_2O – pentane). **^{1}H NMR (400 MHz, $CDCl_3$) δ** 7.28 – 7.38 (4H, m, Ar), 7.19 – 7.25 (1H, m, Ar), 6.48 (1H, d, *J* = 15.9 Hz, C4-H), 6.18 (1H, dt, *J* = 15.9, 7.0 Hz, C3-H), 4.20 (2H, t, *J* = 6.7 Hz, C1-H_2), 2.55 (2H, qd, *J* = 6.8, 1.5 Hz, C2-H_2), 2.06 (3H, s, C(O)Me). **^{13}C NMR (100 MHz, $CDCl_3$) δ** 171.1 (CO), 137.3 (C Ar), 132.4 (C4), 128.6 (2 × C Ar), 127.3 (C Ar), 126.1 (2 × C Ar), 125.6 (C3), 63.8 (C1), 32.4 (C2), 21.0 (C(O)Me). *The spectroscopic properties were consistent with the data available in the literature.*[4]

(S4): (3*E*)-4-Phenylbut-3-en-1-ol

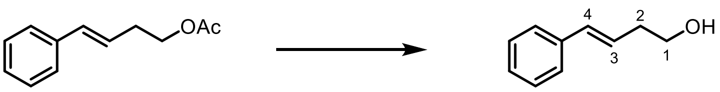

S3 **S4**

To a solution of **S3** (541 mg, 2.85 mmol) in MeOH (30 mL) was added K_2CO_3 (1.97 g, 14.3 mmol, 5.0 equiv.) at 0 °C. The reaction was monitored by TLC until completion and the solid removed by filtration before concentrating *in vacuo*. Flash column chromatography (20% → 50% Et_2O – pentane) afforded **S4** as a colourless oil (369 mg, 87%).

Data for **S4**: **R_f** 0.40 (75% Et_2O – pentane). **^{1}H NMR (400 MHz, $CDCl_3$) δ** 7.28 – 7.39 (4H, m, Ar), 7.19 – 7.25 (1H, m, Ar), 6.51 (1H, d, *J* = 15.8 Hz, C4-H), 6.21 (1H, dt, *J* = 15.9, 7.1 Hz, C3-H), 3.73 – 3.81 (2H, m, C1-H_2), 2.47 – 2.53 (2H, m, C2-H_2), 1.47 (1H, *br.* s, *O*H). **^{13}C NMR (100 MHz, $CDCl_3$)**

δ 137.2 (C Ar), 132.9 (C4), 128.6 (2 × C Ar), 127.3 (C Ar), 126.3 (C3), 126.1 (2 × C Ar), 62.0 (C1), 36.4 (C2). *The spectroscopic properties were consistent with the data available in the literature.*[5]

(S5): *tert*-Butyl [(2-nitrophenyl)sulfonyl]carbamate

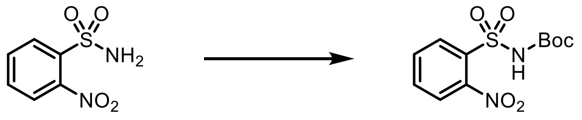

S5

To a solution of 2-nitrobenzene sulfonamide (1.00 g, 4.95 mmol, 1.00 equiv.), DMAP (640 mg, 0.50 mmol, 0.1 equiv.), in CH_2Cl_2 (10 mL) was added Et3N (0.83 mL, 5.95 mmol, 1.20 equiv.) followed by di-tert-butyl decarbonate (1.10 g, 4.95 mmol, 1.00 equiv.) at 0 °C. The mixture was stirred at rt for 8 h before concentrating *in vacuo* and re-dissolving in EtOAc. The solution was washed with 1.0 M HCl before drying over Na_2SO_4 and concentrating *in vacuo*. Recrystallisation from hot hexane afforded **S5** as a crystalline white solid (1.34 g, 80%).

Data for **S5**: **^{1}H NMR (400 MHz, $CDCl_3$) δ** 8.31 – 8.36 (1H, m, Ar), 7.83 – 7.88 (1H, m, Ar), 7.74 – 7.82 (2H, m, Ar), 1.43 (9H, s, C(C**H**$_3$)$_3$). **^{13}C NMR (100 MHz, $CDCl_3$) δ** 148.8 (C(O)), 148.2 (C Ar), 134.8 (C Ar), 133.4 (C Ar), 132.6 (C Ar), 132.2 (C Ar), 125.2 (C Ar), 84.9 (**C**(CH$_3$)$_3$), 28.0 (C(**C**H$_3$)$_3$). *The spectroscopic properties were consistent with the data available in the literature.*[6]

(S6): *tert*-Butyl (3*E*)-[(2-nitrophenyl)sulfonyl](4-phenylbut-3-en-1-yl)carbamate

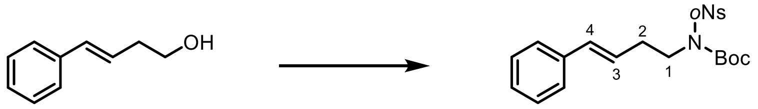

S4 **S6**

Prepared by **General Procedure D** using alcohol **S4** (201 mg, 1.36 mmol), **S5** (442 mg, 1.63 mmol), triphenylphosphine (535 mg, 2.04 mmol) and DIAD (359 mg, 1.77 mmol). Flash column chromatography (15% → 25% Et_2O – pentane) afforded **S6** as a colourless oil (474 mg, 80%).

Data for **S6**: **R_f** 0.50 (60% Et_2O – pentane). **^{1}H NMR (400 MHz, $CDCl_3$) δ** 8.27 – 8.33 (1H, m, Ar), 7.68 – 7.76 (3H, m, Ar), 7.33 – 7.39 (2H, m, Ar), 7.26 – 7.32 (2H, m, Ar), 7.17 – 7.23 (1H, m, Ar), 6.52 (1H, d, J = 15.8 Hz, C4-H), 6.21 (1H, dt, J = 15.8, 7.3 Hz, C3-H), 3.88 – 3.96 (2H, m, C1-H$_2$), 2.66 (2H, qd, J = 7.3, 1.4 Hz, C2-H$_2$), 1.33 (9H, s, C(C**H$_3$**)$_3$). **^{13}C NMR (100 MHz, $CDCl_3$) δ** 150.5 (CO), 147.8 (C Ar), 137.5 (C Ar), 134.2 (C Ar), 133.8 (C Ar), 133.4 (C Ar), 133.0 (C4), 131.8 (C Ar), 128.6 (2 × C Ar), 127.3 (C Ar), 126.4 (2 × C Ar), 126.0 (C3), 124.5 (C Ar), 85.1 (**C**(CH$_3$)$_3$), 47.7 (C1), 34.1 (C2), 28.0 (C(**C**H$_3$)$_3$). **HRMS** (ESI): calculated for $C_{21}H_{25}N_2O_6SN$ $[M+H]^+$ requires m/z 433.1729, found m/z 433.1734. **IR** (film) ν_{max}: 2980, 2888, 1731, 1542, 1369, 1152cm^{-1}.

(1g): (3*E*)-2-Nitro-*N*-(4-phenylbut-3-en-1-yl)benzenesulfonamide

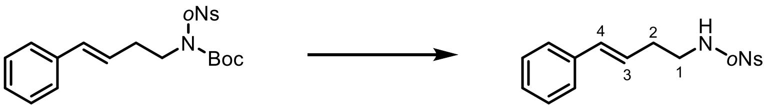

S6 **1g**

Prepared by **General Procedure E** using **S6** (454 mg, 1.05 mmol) and TFA (0.40 mL, 5.26 mmol). Flash column chromatography (10% → 40% Et_2O – pentane) afforded **1g** as a colourless oil (312 mg, 85%).

Data for **1g**: $\mathbf{R_f}$ 0.40 (40% EtOAc – pentane). **^{1}H NMR (400 MHz, $CDCl_3$) δ** 8.13 (1H, dd, *J* = 7.6, 1.6 Hz, Ar), 7.75 (1H, dd, *J* = 7.7, 1.6 Hz, Ar), 7.62 – 7.72 (2H, m, Ar), 7.18 – 7.30 (5H, m, Ar), 6.36 (1H, d, *J* = 15.8 Hz, C4-H), 5.99 (1H, dt, *J* = 15.8, 7.1 Hz, C3-H), 5.43 (1H, t, *J* = 6.5 Hz, *N*H), 3.29 (2H, q, *J* = 6.5 Hz, C1-H_2), 2.43 (qd, *J* = 7.1, 1.4 Hz, C2-H_2). **^{13}C NMR (100 MHz, $CDCl_3$) δ** 147.9 (C Ar), 136.8 (C Ar), 133.8 (C Ar), 133.5 (C4), 133.4 (C Ar), 132.8 (C Ar), 130.9 (C Ar), 128.6 (2 × C Ar), 127.6 (C Ar), 126.2 (2 × CH Ar), 125.4 (C Ar), 125.3 (C3). *The spectroscopic properties were consistent with the data available in the literature.*[7]

(S7): *tert*-Butyl but-3-en-1-yl[(2-nitrophenyl)sulfonyl]carbamate

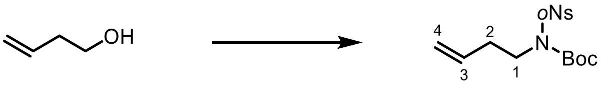

S7

Prepared by **General Procedure D** using but-3-en-1-ol (150 mg, 2.08 mmol), **S5** (755 mg, 2.50 mmol), triphenylphosphine (709 mg, 2.70 mmol) and DIAD (630 mg, 3.12 mmol). Flash column chromatography (15% → 25% EtOAc – pentane) afforded **S7** as a colourless oil (740 mg, 99%).

Data for **S7**: $\mathbf{R_f}$ 0.50 (60% EtOAc – pentane). **^{1}H NMR (400 MHz, $CDCl_3$) δ** 8.27 – 8.32 (1H, m, Ar), 7.69 – 7.76 (3H, m, Ar), 5.82 (1H, ddt, *J* = 17.2, 10.2, 7.0 Hz, C3-H), 5.07 – 5.19 (2H, m, C4-H_2), 3.79 – 3.86 (2H, m, C1-H_2), 2.50 (2H, qd, *J* = 7.1, 1.3 Hz, C2-H_2), 1.36 (9H, s, C(C$\mathbf{H_3}$)$_3$). **^{13}C NMR (100 MHz, $CDCl_3$) δ** 150.5 (CO), 147.8 (C Ar), 134.3 (C3), 134.2 (C Ar), 133.7 (C Ar), 133.5 (C Ar), 131.8 (C Ar), 124.5 (C Ar), 117.9 (C4), 85.1 (**C**(CH_3)$_3$), 47.5 (C1), 34.8 (C2), 28.0 (C(**C**H_3)$_3$). **HRMS**: stable ion was not found in ESI, EI and CI. **IR** (film) ν_{max}: 2980, 1721, 1607, 1510, 1348, 1246 cm^{-1}.

(S8): *N*-(but-3-en-1-yl)-2-nitrobenzenesulfonamide

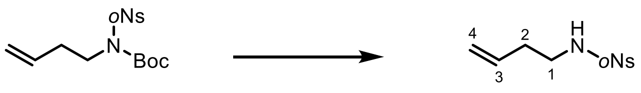

S7 **S8**

Prepared by **General Procedure E** using **S7** (720 mg, 2.02 mmol) and TFA (0.77 mL, 10 mmol). Flash column chromatography (10% → 40% Et_2O – pentane) afforded **S8** as a colourless oil (535 mg, 85%).

Data for **S8**: **R$_f$** 0.40 (70% Et_2O – pentane). **^{1}H NMR (400 MHz, CDCl$_3$) δ** 8.11 – 8.16 (1H, m, Ar), 7.84 – 7.89 (1H, m, Ar), 7.71 – 7.77 (2H, m, Ar), 5.66 (1H, ddt, *J* = 17.1, 10.3, 6.9 Hz, C3-H), 5.34 (1H, t, *J* = 6.5 Hz, *N*H), 5.00 – 5.10 (2H, m, C4-H_2), 3.18 (2H, q, *J* = 6.5 Hz, C1-H_2), 2.28 (qt, *J* = 6.5, 1.3 Hz, C2-H_2). **^{13}C NMR (100 MHz, CDCl3) δ** 134.0 (C Ar), 133.9 (C3), 133.7 (2 × C Ar), 132.9 (C Ar), 131.2 (C Ar), 125.5 (C Ar), 118.5 (C4), 43.0 (C1), 33.8 (C2). *The spectroscopic properties were consistent with the data available in the literature.*[8]

(1h): (3*E*)-*N*-[4-(4-Bromophenyl)but-3-en-1-yl]-2-nitrobenzenesulfonamide

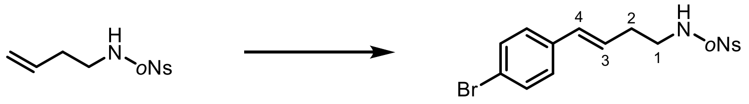

S8 **1h**

Prepared by **General Procedure C** using **S8** (104 mg, 0.407 mmol), 1-bromo-4-vinylbenzene (0.11 mL, 0.814 mmol) and Grubbs II (5 mg, 0.06 mmol) in PhMe at 100 ˚C. Flash column chromatography (15% → 25% Et_2O – pentane) afforded **1h** as a colourless oil (53 mg, 31%).

Data for **1h**: **R$_f$** 0.45 (40% EtOAc – pentane). **^{1}H NMR (400 MHz, CDCl$_3$) δ** 8.14 (1H, dd, *J* = 7.6, 1.7 Hz, Ar), 7.79 (1H, dd, *J* = 7.6, 1.6 Hz, Ar), 7.65 – 7.74 (2H, m, Ar), 7.40 (2H, d, *J* = 8.5 Hz, Ar), 7.11 (2H, d, *J* = 8.5 Hz, Ar), 6.31 (1H, d, *J* = 15.9 Hz, C4-H), 6.00 (1H, dt, *J* = 15.8, 7.1 Hz, C3-H), 5.37 (1H, t, *J* = 6.5 Hz, *N*H), 3.28 (2H, q, *J* = 6.5 Hz, C1-H_2), 2.43 (2H, qd, *J* = 6.5, 1.4 Hz, C2-H_2). **^{13}C NMR (100 MHz, CDCl$_3$) δ** 135.8 (C Ar), 134.0 (2 × C Ar), 133.6 (C Ar), 132.9 (C Ar), 132.5 (C4), 131.8 (2 × C Ar), 131.1 (C Ar), 127.8 (2 × C Ar), 126.2 (C3), 125.6 (C Ar), 121.4 (C Ar), 43.4 (C1), 33.3 (C2). **HRMS** (ESI): calculated for $C_{16}H_{15}N_2O_4SBrNa$ $[M+Na]^+$ requires *m/z* 432.9834, found *m/z* 432.9828. **IR** (film) ν_{max}: 3334, 2980, 2888, 2360, 1536, 1382 cm^{-1}.

(1i): *N*-(4-Methylpent-3-en-1-yl)-2-nitrobenzenesulfonamide

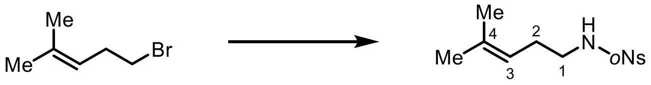

1i

A solution of 5-bromo-2-methylpent-2-ene (500 mg, 3.00 mmol, 1.10 equiv.), 2-nitrobenzenesulfonamide (553 mg, 2.74 mg, 1.00 equiv.), K_2CO_3 (755 mg, 5.47 mmol, 2.00 equiv.) in acetone (3 mL) was heated to 60 °C for 16 h. The reaction mixture was filtered through a plug of Celite, washed with EtOAc and concentrated *in vacuo*. Flash column chromatography (10% → 15% Et_2O – pentane) afforded **1i** as a colourless oil (107 mg, 14%).

Data for **1i**: **R_f** 0.40 (30% EtOAc – pentane). **^{1}H NMR (400 MHz, $CDCl_3$) δ** 8.11 – 8.15 (1H, m, Ar), 7.84 – 7.89 (1H, m, Ar), 7.70 – 7.77 (2H, m, Ar), 5.28 (1H, t, *J* = 6.6 Hz, *N*H), 4.94 (1H, tp, *J* = 7.4, 1.5 Hz, C3-H), 3.10 (2H, q, *J* = 6.6 Hz, C1-H_2), 2.22 (2H, q, *J* = 7.0 Hz, C2-H_2), 1.66 (3H, s, Me), 1.56 (3H, s, Me). **^{13}C NMR (100 MHz, $CDCl_3$) δ** 148.2 (C Ar), 136.2 (C4), 134.0 (C Ar), 133.6 (C Ar), 132.9 (C Ar), 131.2 (C Ar), 125.5 (C Ar), 119.5 (C3), 43.8 (C1), 28.2 (C2), 25.9 (Me), 18.0 (Me). **HRMS** (ESI): calculated for $C_{12}H_{16}N_2O_4SNa$ $[M+Na]^+$ requires *m/z* 307.0728, found *m/z* 307.0722. **IR** (film) ν_{max}: 3341, 2980, 1538, 1408, 1338, 1162 cm^{-1}.

(1j): *N*-(3-Cyclopentylidenepropyl)-2-nitrobenzenesulfonamide

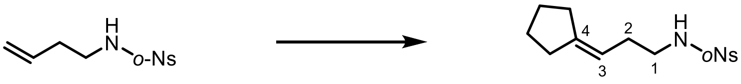

S8 **1j**

Prepared by **General Procedure C** using **S8** (625 mg, 2.44 mmol), methylenecyclopentane (1.00 g, 12.2 mmol) and Grubbs II (31 mg, 0.036 mmol) in CH_2Cl_2 at rt for 72 h. Flash column chromatography (10% → 15% EtOAc – pentane) afforded **1j** as a colourless oil (134 mg, 18%).

Data for **1j**: **R_f** 0.40 (30% EtOAc – pentane). **^{1}H NMR (400 MHz, $CDCl_3$) δ** 8.10 – 8.15 (1H, m, Ar), 7.83 – 7.88 (1H, m, Ar), 7.70 – 7.76 (2H, m, Ar), 5.30 (1H, t, *J* = 6.4 Hz, *N*H), 5.05 (1H, tt, *J* = 7.3, 2.3 Hz, C3-H), 3.11 (2H, q, *J* = 6.4 Hz, C1-H_2), 2.06 – 2.24 (7H, m, C2-H_2 + C-H cyclopentyl + 2 × CH_2 cyclopentyl), 1.51 – 1.68 (5H, m, C-H cyclopentyl + 2 × C-H_2 cyclopentyl). **^{13}C NMR (100 MHz, $CDCl_3$) δ** 148.2 (C Ar), 134.0 (C Ar), 133.6 (C Ar), 132.9 (C Ar), 131.3 (C4), 131.2 (C Ar), 125.5 (C Ar), 114.9 (C3), 43.7 (C1), 33.8 (CH_2 cyclopentyl), 29.8 (CH_2 cyclopentyl), 29.0 (CH_2 cyclopentyl), 26.4 and 26.3 (CH_2 cyclopentyl + C2). **HRMS** (ESI): calculated for $C_{14}H_{18}N_2O_4SNa$ $[M+Na]^+$ requires *m/z* 333.09, found *m/z* 333.09. **IR** (film) ν_{max}: 3341, 2980, 2888, 1538, 1340, 1163 cm^{-1}.

(S9): (*E*)-1-(Buta-1,3-dien-1-yl)-4-methoxybenzene

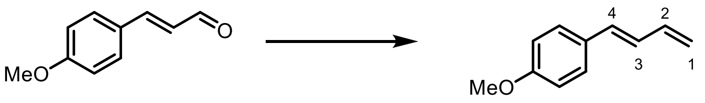

S9

Prepared according to the procedure reported by Doye et. al.[9] To suspension of methyltriphenylphosphonium bromide (8.57 g, 24.0 mmol) in THF (100 mL) was added *n*-butyllithium (2.5 M in hexanes, 9.60 mL, 24.0 mmol) dropwise at 0 °C. After stirring at 0 °C for 1.5 h 4-methoxy-cinnamaldehyde (3.24 g, 20.0 mmol) in THF (20 mL) was added slowly and allowed to warm up to rt. After 1 h, silica gel (10 g) and hexane (150 mL) was added. After stirring for 15 minutes the solid was removed by filtration and the filtrate concentrated *in vacuo*. Flash column chromatography (100% pentane) afforded **S9** as a crystalline white solid (2.34 g, 73%).

Data for **S9**: **R$_f$** 0.80 (5% Et_2O – pentane). **^{1}H NMR (400 MHz, $CDCl_3$) δ** 7.25 (2H, d, *J* = 8.7 Hz, Ar), 6.77 (2H, d, *J* = 8.8 Hz, Ar), 6.67 – 6.54 (1H, m, C4-H), 6.49 – 6.34 (2H, m, C2-H + C3-H), 5.21 (1H, ddd, *J* = 16.9, 1.8, 0.9 Hz, C1-H$_A$), 5.12 – 4.96 (1H, m, C1-H$_B$), 3.68 (s, 3H, OMe). **^{13}C NMR (101 MHz, $CDCl_3$) δ** 159.5 (C Ar), 137.6 (C2), 132.6 (C3), 130.1 (C Ar), 127.8 (C4), 127.8 (2 × C Ar), 116.5 (C1), 114.2 (2 × C Ar), 55.3 (OMe). *The spectroscopic properties were consistent with the data available in the literature.*[9]

(1k): *N*-[(3*E*,5*E*)-6-(4-Methoxyphenyl)hexa-3,5-dien-1-yl]-2-nitrobenzenesulfonamide and *N*-[(3*E*,5*Z*)-6-(4-Methoxyphenyl)hexa-3,5-dien-1-yl]-2-nitrobenzenesulfonamide

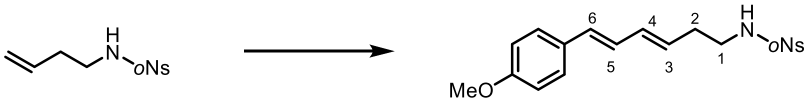

S8 **1k**

E,E : *E,Z*
6:1

Prepared by **General Procedure C** using **S8** (95 mg, 0.37 mmol), **S9** (118 mg, 0.738 mmol) and Grubbs I (15 mg, 0.019 mmol, 5 mol%) in PhMe at 40 °C for 16 h. Flash column chromatography (20% → 35% EtOAc – pentane) afforded **1k** as a partially separable 6:1 mixture of ***E,E*-1k** and ***E,Z*-1k**; ***E,E*-1k** was isolated as a yellow oil (15 mg, 15%) and a 4:1 mixture of ***E,Z*-1k** + ***E,E*-1k** was isolated as a yellow oil (39 mg, 36%).

Data for ***E,E*-1k**: **R$_f$** 0.50 (40% EtOAc – pentane). **^{1}H NMR (500 MHz, $CDCl_3$) δ** 8.14 (1H, dd, *J* = 7.6, 1.6 Hz, Ar), 7.83 (1H, dd, *J* = 7.6, 1.6 Hz, Ar), 7.67 – 7.76 (2H, m, Ar), 7.28 (2H, d, *J* = 8.7 Hz, Ar), 6.85 (2H, d, *J* = 8.7 Hz, Ar), 6.47 (1H, dd, *J* = 15.6, 9.9 Hz, C5-H), 6.36 (1H, d, *J* = 15.6 Hz, C6-H), 6.14 (1H, ddt, *J* = 14.8, 9.9, 1.3 Hz, C4-H), 5.52 (1H, dt, *J* = 14.8, 6.8 Hz, C3-H), 5.37 (1H, t, *J* = 6.8 Hz,

*N*H), 3.81 (3H, s, OMe), 3.23 (2H, q, *J* = 6.8 Hz, C1-H_2), 2.35 (2H, qd, *J* = 6.8, 1.3 Hz, C2-H_2). **^{13}C NMR (125 MHz, $CDCl_3$) δ** 159.4 (C Ar), 134.3 (C4), 134.2 (2 × C Ar), 133.6 (C Ar), 132.9 (C Ar), 131.6 (C6), 131.1 (C Ar), 130.1 (C Ar), 128.1 (C3), 127.7 (2 × C Ar), 126.4 (C5), 125.6 (C Ar), 114.2 (2 × C Ar), 55.4 (OMe), 43.6 (C1), 33.1 (C2). **HRMS** (EI): calculated for $C_{19}H_{20}N_2O_5SNa$ $[M+Na]^+$ requires *m/z* 411.0991, found *m/z* 411.0986. **IR** (film) ν_{max}: 3349, 2980, 2888, 1541, 1428, 1163 cm^{-1}.

Partial data for ***E*,*Z*-1k** (from the mixture): **^{1}H NMR (500 MHz, $CDCl_3$) δ** 8.14 (1H, dd, *J* = 7.6, 1.6 Hz, Ar), 7.83 (1H, dd, *J* = 7.6, 1.6 Hz, Ar), 7.60 – 7.79 (2H, m, Ar), 7.30 (2H, d, *J* = 8.7 Hz, Ar), 6.85 (2H, d, *J* = 8.7 Hz, Ar), 6.69 (1H, ddd, *J* = 15.4, 11.1, 1.2 Hz, C5-H), 6.13 (1H, d, *J* = 15.4 Hz, C6-H), 6.15 – 6.23 (1H, m, C4-H), 5.43 (1H, d, *J* = 6.4 Hz, *N*H), 5.28 (1H, dt, *J* = 10.7, 7.7 Hz, C3-H), 3.82 (3H, s, OMe), 3.19-3.26 (2H, m, C1-H_2), 2.50 (2H, qd, *J* = 6.9, 1.5 Hz, C2-H_2). **^{13}C NMR (125 MHz, $CDCl_3$) δ** 159.6 (C Ar), 148.1 (C Ar), 134.1 (C Ar), 133.71 (C Ar), 133.66 (C Ar), 132.5 (C4), 131.6 (C6), 131.1 (C Ar), 130.0 (C Ar), 127.9 (2 × C Ar), 125.6 (C Ar), 125.5 (C3), 121.4 (C5), 114.2 (2 × C Ar), 55.5 (OMe), 43.7 (C1), 28.4 (C2).

(S10): (3*E*)-Buta-1,3-dien-1-ylbenzene

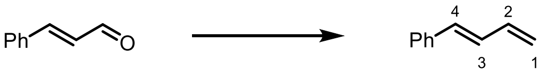

S10

To suspension of methyltriphenylphosphonium bromide (6.13 g, 17.5 mmol) in THF (55 mL) was added *n*-butyllithium (2.5 M in hexanes, 8.00 mL, 15.9 mmol) dropwise at 0 °C. After stirring at 0 °C for 1.5 h cinnamaldehyde (2.00 mL, 15.9 mmol) in THF (5 mL) was added slowly and allowed to warm up to rt. After 1 h, silica gel (10 g) and hexane (150 mL) was added. After stirring for 15 minutes the solid was removed by filtration and the filtrate concentrated *in vacuo*. Flash column chromatography (0% → 5% Et_2O – pentane) afforded **S10** as a crystalline white solid (817 mg, 41%).

Data for **S10**: **R_f** 0.50 (10% Et_2O – pentane). **^{1}H NMR (400 MHz, $CDCl_3$) δ** 7.38 – 7.44 (2H, m, Ar), 7.29 – 7.36 (2H, m, Ar), 7.20 – 7.27 (1H, m, Ar), 6.80 (1H, dd, *J* = 15.7, 10.5 Hz, C3-H), 6.58 (1H, d, *J* = 15.7 Hz, C4-H), 6.52 (1H, dt, *J* = 16.9, 10.5 Hz, C2-H), 5.35 (1H, d, *J* = 16.9 Hz, C1-H_A), 5.19 (1H, d, *J* = 10.5 Hz, C1-H_B). **^{13}C NMR (100 MHz, $CDCl_3$) δ** 137.3 (C2), 137.3 (C Ar), 133.0 (C4), 129.8 (C3), 128.7 (2 × C Ar), 127.8 (C Ar), 126.6 (2 × C Ar), 117.8 (C1). *The spectroscopic properties were consistent with the data available in the literature.*[10]

(1l): *N*-[(3*E*,5*E*)-6-Phenyl-hexa-3,5-dien-1-yl]-2-nitrobenzenesulfonamide and *N*-[(3*E*,5*Z*)-6-Phenyl-hexa-3,5-dien-1-yl]-2-nitrobenzenesulfonamide

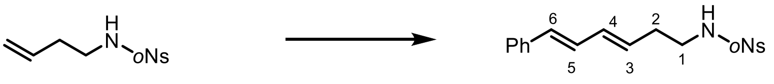

S8 **1l**

E,E : *E,Z*
5:1

Prepared by **General Procedure C** using sulfonamide **S8** (228 mg, 0.969 mmol), **S10** (259 mg, 1.94 mmol) and Grubbs I (40 mg, 0.048 mmol, 5 mol%) in PhMe at 40 °C for 16 h. Flash column chromatography (10% → 35% EtOAc – pentane) afforded **1l** as a partially separable 5:1 mixture of ***E,E*-1l** and ***E,Z*-1l**; ***E,E*-1l** was isolated as a yellow oil (45 mg, 13%) and a 3.5:1 mixture of ***E,Z*-1l** + ***E,E*-1l** was isolated as a yellow oil (147 mg, 43%).

Data for ***E,E*-1l**: $\mathbf{R_f}$ 0.40 (40% EtOAc – pentane). **^{1}H NMR (500 MHz, $CDCl_3$) δ** 8.15 (1H, dd, *J* = 7.6, 1.7 Hz, Ar), 7.84 (1H, dd, *J* = 7.7, 1.6 Hz, Ar), 7.67 – 7.82 (2H, m, Ar), 7.28 – 7.38 (4H, m, Ar), 7.19 – 7.24 (1H, m, Ar), 6.60 (1H, dd, *J* = 15.7, 10.4 Hz, C5-H), 6.42 (1H, d, *J* = 15.7 Hz, C6-H), 6.17 (1H, ddt, *J* = 15.2, 10.4, 1.4 Hz, C4-H), 5.59 (1H, dt, *J* = 15.2, 6.4 Hz, C3-H), 5.36 (1H, t, *J* = 6.4 Hz, *N*H), 3.24 (q, *J* = 6.4 Hz, C1-H_2), 2.37 (2H, qd, *J* = 6.4, 1.4 Hz, C2-H_2). **^{13}C NMR (125 MHz, $CDCl_3$) δ** 137.2 (C Ar), 134.1 (C Ar), 134.1 (C4), 133.6 (C Ar), 132.9 (C Ar), 132.1 (C6), 131.2 (C Ar), 129.4 (C3), 128.8 (2 × C Ar), 128.3 (C5), 127.7 (C Ar), 126.5 (2 × C Ar), 125.6 (C Ar), 43.5 (C1), 33.1 (C2). **HRMS** (ESI): calculated for $C_{18}H_{19}N_2O_4SNa$ $[M+H]^+$ requires *m/z* 359.1060, found *m/z* 359.1063. **IR** (film) ν_{max}: 3304, 2980, 2888, 1382, 1251, 1158 cm^{-1}.

Partial data for ***E,Z*-1l** (from the mixture): **^{1}H NMR (500 MHz, $CDCl_3$) δ** 8.15 (1H, dd, *J* = 7.6, 1.7 Hz, Ar), 7.68 – 7.78 (2H, m, Ar), 7.61 – 7.66 (1H, m, Ar), 7.28 – 7.38 (4H, m, Ar), 7.19 – 7.24 (1H, m, Ar), 6.82 (1H, ddd, *J* = 15.5, 11.1, 1.2 Hz, C5-H), 6.53 (1H, d, *J* = 15.5 Hz, C6-H), 6.20 – 6.26 (1H, m, C4-H), 5.36 (1H, dt, *J* = 10.8, 7.7 Hz, C3-H), 3.23 – 3.26 (2H, m, C1-H_2), 2.52 (2H, qd, *J* = 6.9, 1.5 Hz, C2-H_2). **^{13}C NMR (125 MHz, $CDCl_3$) δ** 137.2 (C Ar), 134.2 (C6), 134.2 (C Ar), 133.7 (C Ar), 132.9 (2 × C Ar), 132.3 (C4), 131.1 (C Ar), 129.4 (C Ar), 128.8 (2 × C Ar), 128.0 (C Ar), 126.7 (C3), 126.7 (2 × C Ar), 123.4 (C5), 43.7 (C1), 28.5 (C2).

(S11): Allyltriisopropylsilane

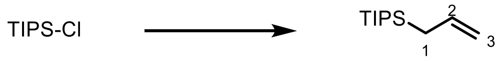

S11

To a solution of triisopropylsilyl chloride (1.10 mL, 5.21 mmol) in anhydrous THF (1.5 mL) was added allylmagnesium bromide (1.0 M in Et_2O, 7.3 mL, 7.3 mmol) at 0 °C. After warming to rt, the reaction

mixture was heated to reflux for 16 h. The reaction was cooled to 0 °C before addition of aq. NH_4Cl. The aqueous later was extracted with Et_2O and the combined organic layers were washed with brine and dried over $MgSO_4$ before concentrating *in vacuo*. Flash column chromatography (0% → 5% Et_2O – pentane) afforded **S11** as a colourless oil (1.02 g, 99%).

Data for **S11**: **R_f** 0.40 (2% Et_2O – pentane). **^{1}H NMR (400 MHz, $CDCl_3$) δ** 5.88 (1H, ddt, *J* = 16.9, 10.0, 8.1 Hz, C2-H), 4.76 – 4.97 (2H, m, C3-H_2), 1.64 (2H, d, *J* = 8.1 Hz, C1-H_2), 1.06 (18H, s, 6 × CH(C**H_3**)$_2$), 1.03 – 1.08 (3H, m, 3 × C**H**(CH_3)$_2$). **^{13}C NMR (100 MHz, $CDCl_3$) δ** 136.4 (C2), 112.9 (C3), 18.8 (6 × CH(**C**H_3)$_2$), 11.2 (3 × **C**H(CH_3)$_2$). *The spectroscopic properties were consistent with the data available in the literature.*[11]

(1m): (3*E*)-2-Nitro-*N*-[5-(triisopropylsilyl)pent-3-en-1-yl]benzenesulfonamide and (3*Z*)-2-Nitro-*N*-[5-(triisopropylsilyl)pent-3-en-1-yl]benzenesulfonamide

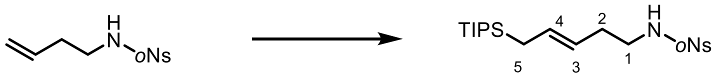

S8 **1m**
E:Z
4:1

Prepared by **General Procedure C** using **S8** (511 mg, 2.00 mmol), **S11** (790 mg, 4.00 mmol) and Grubbs II (25 mg, 0.030 mmol) in PhMe at 100 °C for 16 h. Flash column chromatography (20% → 35% EtOAc – pentane) afforded **1m** as an inseparable 4:1 mixture of ***E*-1m** and ***Z*-1m** as a yellow oil (770 mg, 90%).

Data for ***E*-1m** (from the mixture): **^{1}H NMR (400 MHz, $CDCl_3$) δ** 8.11 – 8.16 (1H, m, Ar), 7.83 – 7.88 (1H, m, Ar), 7.70 – 7.76 (2H, m, Ar), 5.51 (1H, dtt, *J* = 15.3, 8.1, 1.3 Hz, C4-H), 5.29 (1H, t, *J* = 5.9 Hz, *N*H), 5.13 (1H, dtt, *J* = 15.3, 6.9, 1.3 Hz, C3-H), 3.10 (2H, td, *J* = 6.9, 5.9 Hz, C1-H_2), 2.18 (2H, qd, *J* = 6.9, 1.2 Hz, C2-H_2), 1.52 (2H, dd, *J* = 8.1, 1.3 Hz, C5-H_2), 1.01 (18H, s, 6 × CH(C**H_3**)$_2$), 0.97 – 1.04 (3H, m, 3 × C**H**(CH_3)$_2$). **^{13}C NMR (100 MHz, $CDCl_3$) δ** 148.3 (C Ar), 134.1 (C Ar), 133.58 (C Ar), 132.8 (C Ar), 132.0 (C4), 131.2 (C Ar), 125.5 (C Ar), 123.3 (C3), 43.8 (C1), 33.0 (C2), 18.8 (6 × CH(**C**H_3)$_2$), 15.7 (C-5), 11.1 (3 × **C**H(CH_3)$_2$).

Partial data for ***Z*-1m** (from the mixture): **^{1}H NMR (400 MHz, $CDCl_3$) δ** 8.11 – 8.16 (1H, m, Ar), 7.83 – 7.88 (1H, m, Ar), 7.70 – 7.76 (2H, m, Ar), 5.50 – 5.58 (1H, m, C3-H), 5.63 (1H, dtt, *J* = 10.2, 8.6, 1.6 Hz, C4-H), 5.34 (1H, d, *J* = 5.9 Hz, *N*H), 5.00 – 5.08 (1H, m, C3-H), 3.14 (1H, td, *J* = 6.9, 5.9 Hz, C1-H_2), 2.29 (2H, qd, *J* = 6.9, 1.6 Hz, C2-H_2), 1.52 (2H, dd, *J* = 8.6, 1.3 Hz, C5-H_2), 1.01 (18H, s, 6 × CH(C**H_3**)$_2$), 0.97 – 1.04 (3H, m, 3 × C**H**(CH_3)$_2$). **^{13}C NMR (100 MHz, $CDCl_3$) δ** 148.3 (C Ar), 134.1 (C Ar), 133.6 (C Ar), 132.9 (C Ar), 131.3 (C Ar), 130.9 (C4), 125.51 (C Ar), 121.7 (C3), 43.8 (C1), 27.4 (C2), 18.8 (6 × CH(**C**H_3)$_2$), 11.2 (C5 + 3 × **C**H(CH_3)$_2$).

Data for both ***E*-1m** and ***Z*-1m**: **R$_f$** 0.30 (45% Et_2O – pentane). **HRMS**: stable ion was not found in ESI, EI and CI. **IR** (film) ν_{max}: 3347, 2980, 2888, 1540, 1382, 1164 cm^{-1}.

(S12): Allyl(*tert*-butyl)diphenylsilane

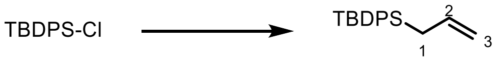

S12

To a solution of *tert*-butylchlorodiphenylsilane (1.00 mL, 3.91 mmol) in anhydrous THF (2.3 mL) was added allylmagnesium bromide (1.0 M in Et_2O, 4.3 mL, 4.3 mmol) at 0 °C. After warming to rt, the reaction mixture was left to stir for 16 h. The reaction was cooled to 0 °C before addition of aq. NH_4Cl. The aqueous later was extracted with Et_2O and the combined organic layers were washed with brine and dried over $MgSO_4$ before concentrating *in vacuo*. Flash column chromatography (0% → 5% Et_2O – pentane) afforded **S12** as a colourless oil (458 mg, 42%).

Data for **S12**: **R$_f$** 0.40 (2% Et_2O – pentane). **^{1}H NMR (400 MHz, CDCl$_3$) δ** 7.59 – 7.65 (4H, m, Ar), 7.32 – 7.43 (6H, m, Ar), 5.79 (1H, ddt, *J* = 17.0, 10.0, 7.9 Hz, C2-H), 4.92 (1H, dq, *J* = 17.0, 1.6 Hz, C3-H$_A$), 4.81 (1H, ddt, *J* = 10.0, 2.1, 1.2 Hz, C3-H$_B$), 2.20 (2H, dt, *J* = 7.9, 1.3 Hz, C1-H$_2$), 1.08 (9H, s, C(C**H$_3$**)$_3$). **^{13}C NMR (100 MHz, CDCl$_3$) δ** 136.2 (4 × C Ar), 134.8 (2 × C Ar), 134.6 (C2), 129.2 (2 × C Ar), 127.7 (4 × C Ar), 114.6 (C3), 28.0 (C1), 18.9 (C(**C**H$_3$)$_3$), 18.6 (**C**(CH$_3$)$_3$). *The spectroscopic properties were consistent with the data available in the literature.*[12]

(1n): (3*E*)-2-Nitro-*N*-[5-(*tert*-butyldiphenylsilyl)pent-3-en-1-yl]benzenesulfonamide and (3*Z*)-2-Nitro-*N*-[5-(*tert*-butyldiphenylsilyl)pent-3-en-1-yl]benzenesulfonamide

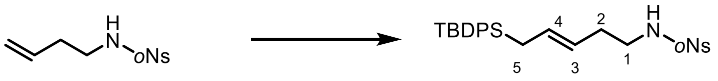

S8 **1n** *E:Z* 3:1

Prepared by **General Procedure C** using **S8** (177 mg, 0.691 mmol), **S12** (387 mg, 1.38 mmol) and Grubbs II (9.0 mg, 0.010 mmol) in PhMe at 100 °C for 16 h. Flash column chromatography (15% → 25% EtOAc – pentane) afforded **1n** as a partially separable 3:1 mixture of ***E*-1n** and ***Z*-1n**; a 16:1 mixture of ***E*-1n** + ***Z*-1n** was isolated as a yellow oil (18 mg, 1%) and a 3:1 mixture of ***E*-1n** + ***Z*-1n** was isolated as a yellow oil (285 mg, 85%).

Data for ***E*-1n**: **R$_f$** 0.50 (60% Et_2O – pentane). **^{1}H NMR (400 MHz, CDCl$_3$) δ** 8.03 – 8.08 (1H, m, Ar), 7.78 – 7.82 (1H, m, Ar), 7.65 – 7.71 (2H, m, Ar), 7.52 – 7.58 (4H, m, Ar), 7.30 – 7.41 (6H, m, Ar), 5.37 (1H, dt, *J* = 15.4, 7.8 Hz, C4-H), 5.10 (1H, t, *J* = 6.5 Hz, *N*H), 5.05 (1H, dt, *J* = 15.4, 7.0 Hz, C3-H), 2.93 (2H, q, *J* = 6.5 Hz, C1-H$_2$), 2.07 (2H, d, *J* = 7.8 Hz, C5-H$_2$), 2.01 (2H, q, *J* = 7.0 Hz, C2-H$_2$), 1.02

(9H, s, C(C**H**$_3$)$_3$). **^{13}C NMR (100 MHz, CDCl$_3$) δ** 136.1 (4 × C Ar), 134.4 (3 × C Ar), 133.9 (C Ar), 133.5 (C Ar), 132.8 (C Ar), 131.2 (C Ar), 130.3 (C4), 129.3 (2 × C Ar), 127.7 (4 × C Ar), 125.4 (C Ar), 125.2 (C3), 45.8 (C1), 32.9 (C2), 28.0 (C(**C**H$_3$)$_3$), 18.5 (**C**(CH$_3$)$_3$), 17.1 (C5). **HRMS**: stable ion was not found in ESI, EI and CI. **IR** (film) ν_{max}: 3347, 2980, 2888, 1539, 1426, 1163 cm^{-1}.

Partial data for ***Z*-1n** (from the mixture): **^{1}H NMR (400 MHz, CDCl$_3$) δ** 8.06 – 8.09 (1H, m, Ar), 7.82 – 7.86 (1H, m, Ar), 7.70 – 7.75 (2H, m, Ar), 7.52 – 7.58 (4H, m, Ar), 7.30 – 7.41 (6H, m, Ar), 5.51 – 5.61 (1H, m, C4-H), 5.13 (1H, t, *J* = 6.5 Hz, *N*H), 4.93 – 5.01 (1H, m, C3-H), 2.85 (2H, td, *J* = 7.0, 5.9 Hz, C1-H$_2$), 1.90-2.04 (4H, m, 1 × C2-H$_2$ + 1 × C5-H$_2$), 1.05 (9H, s, 3 × C(C**H**$_3$)$_3$). **^{13}C NMR (100 MHz, CDCl$_3$) δ** 136.2 (4 × C Ar), 134.4 (3 × C Ar), 133.9 (C Ar), 133.5 (C Ar), 132.8 (C Ar), 131.2 (C Ar), 129.4 (2 × Ar + C4), 127.7 (4 × C Ar), 125.4 (C Ar), 123.3 (C3), 43.5 (C1), 27.95 (C(**C**H$_3$)$_3$), 27.4 (C2), 18.5 (**C**(CH$_3$)$_3$), 12.9 (C5).

((+)-S13): (+)-(*S*)-2-Phenylbut-3-en-1-ol

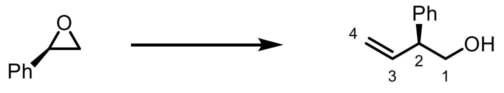

(+)-S13

To a solution of (*R*)-phenyloxirane (1.00 g, 8.33 mmol, 1.00 equiv.) and [Cu(COD)Cl]$_2$ (172 mg, 0.415 mmol) in THF (12 mL) at – 78 °C was added vinylmagnesium bromide (1.0 M in THF, 10.0 mL, 10.0 mmol). The reaction was allowed to warm to rt and stirred for 6 h before addition of aq. NH_4Cl and dilution with EtOAc. The aqueous later was extracted with EtOAc and the combined organic layers were washed with brine and dried over $MgSO_4$ before concentrating *in vacuo*. Flash column chromatography (15% → 20% Et_2O – pentane) afforded **(+)-S13** as a colourless oil (650 mg, 53%).

Data for **(+)-S13**: **R$_f$** 0.40 (40% Et_2O – pentane). **$[\alpha]_D^{25}$** = +62.1 (0.01 g/mL, $CHCl_3$). **^{1}H NMR (400 MHz, CDCl$_3$) δ** 7.20 – 7.37 (2H, m, Ar), 7.20 – 7.27 (3H, m, Ar), 6.00 (1H, ddd, *J* = 17.6, 10.4, 7.7 Hz, C3-H), 5.15 – 5.23 (2H, m, C4-H$_2$), 3.81 (2H, t, *J* = 6.0 Hz, C1-H$_2$), 3.52 (1H, q, *J* = 7.3 Hz, C2-H), 1.57 (1H, *br.* s, *O*H). **^{13}C NMR (100 MHz, CDCl$_3$) δ** 140.7 (C Ar), 138.3 (C3), 128.9 (2 × C Ar), 128.1 (2 × C Ar), 127.1 (C Ar), 117.2 (C4), 66.2 (C1), 52.6 (C2). *The spectroscopic properties were consistent with the data available in the literature.*[13]

Racemic **(±)-S13** was made *via* the same procedure without using [Cu(COD)Cl]$_2$ (55%).

((–)-S14): (–)-*tert*-Butyl (2*S*)-[(2-nitrophenyl)sulfonyl](2-phenylbut-3-en-1-yl)carbamate

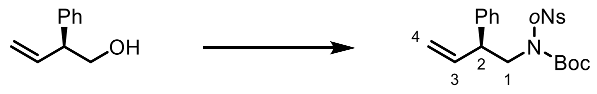

Prepared by **General Procedure D** using **(–)-S13** (652 mg, 4.41 mmol), **S5** (1.60 g, 5.29 mmol), triphenylphosphine (1.73 g, 6.61 mmol) and DIAD (1.15 g, 5.73 mmol). Flash column chromatography (5% → 25% Et_2O – pentane) afforded **(–)-S14** as a colourless oil (1.53 g, 81%).

Data for **(–)-S14**: $\mathbf{R_f}$ 0.30 (50% Et_2O – pentane). $[\alpha]_D^{25}$ = –0.164 (*c* = 10.0, $CHCl_3$). **^{1}H NMR (400 MHz, $CDCl_3$) δ** 8.24 – 8.28 (1H, m, Ar), 7.68 – 7.75 (3H, m, Ar), 7.29 – 7.37 (4H, m, Ar), 7.23 – 7.27 (1H, m, Ar), 6.11 (1H, ddd, *J* = 17.1, 10.2, 8.7 Hz, C3-H), 5.18 – 5.27 (4H, m, C4-H_2), 4.10 (1H, dd, *J* = 14.6, 9.2 Hz, C1-H_A), 4.01 (1H, dd, *J* = 14.6, 6.6 Hz, C1-H_B), 3.78 – 3.86 (1H, m, C2-H), 1.32 (9H, s, 3 × C(C**H**$_3$)$_3$). **^{13}C NMR (100 MHz, $CDCl_3$) δ** 150.4 (CO), 147.7 (C Ar), 140.9 (C Ar), 138.1 (C3), 134.2 (C Ar), 133.8 (C Ar), 133.7 (C Ar), 131.8 (C Ar), 128.8 (2 × C Ar), 128.2 (2 × C Ar), 127.2 (C Ar), 124.5 (C Ar), 117.7 (C4), 85.1 (**C**(CH_3)$_3$), 52.7 (C1), 50.8 (C2), 27.9 (C(**C**H$_3$)$_3$). **HRMS** (ESI): calculated for $C_{21}H_{24}N_2O_6SNa$ $[M+Na]^+$ requires *m/z* 455.1253, found *m/z* 455.1239. **IR** (film) ν_{max}: 2980, 2888, 2360, 1728, 1541, 1365 cm^{-1}.

Racemic (±**)-S14** was made *via* the same procedure using (±**)-S13** (55%).

((+)-S15): (+)-(2*S*)-2-Nitro-*N*-(2-phenylbut-3-en-1-yl)benzenesulfonamide

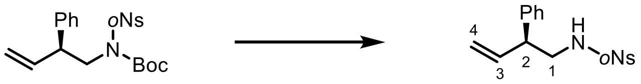

Prepared by **General Procedure E** using **(–)-S14** (320 mg, 0.744 mmol) and TFA (0.29 mL, 3.72 mmol). Flash column chromatography (15% → 50% EtOAc – pentane) afforded **(+)-S15** as a colourless oil (240 mg, 97%).

Data for **(+)-S15**: $\mathbf{R_f}$ 0.40 (70% Et_2O – pentane). $[\alpha]_D^{25}$ = +0.182 (*c* = 10.0, $CHCl_3$). **^{1}H NMR (400 MHz, $CDCl_3$) δ** 8.09 – 8.13 (1H, m, Ar), 7.81 – 7.86 (1H, m, Ar), 7.70 – 7.76 (2H, m, Ar), 7.19 – 7.28 (3H, m, Ar), 7.05 – 7.09 (2H, m, Ar), 5.89 (1H, ddd, *J* = 17.5, 10.3, 7.5 Hz, C3-H), 5.34 (1H, t, *J* = 6.5 Hz, *N*H), 5.06 – 5.20 (2H, m, C4-H_2), 3.49 (1H, q, *J* = 7.5 Hz, C2-H), 3.37 – 3.43 (2H, m, C1-H_2). **^{13}C NMR (100 MHz, CDCl3) δ** 148.1 (C Ar), 139.9 (C Ar), 137.7 (C3), 133.9 (C Ar), 133.7 (C Ar), 133.0 (C Ar), 131.1 (C Ar), 129.1 (2 × C Ar), 127.7 (2 × C Ar), 127.5 (C Ar), 125.6 (C Ar), 117.6 (C4), 49.5 (C1), 48.0 (C2). **HRMS** (ESI): calculated for $C_{16}H_{17}N_2O_4S$ $[M+H]^+$ requires *m/z* 333.0909, found *m/z* 333.0909. **IR** (film) ν_{max}: 3360, 2980, 2888, 2360, 1738, 1545 cm^{-1}.

Racemic (±**)-S15** was made *via* the same procedure using (±**)-S14** (81%).

((–)-1o): (–)-(2*S*,3*E*)-*N*-[4-(4-Methoxyphenyl)-2-phenylbut-3-en-1-yl]-2-nitrobenzenesulfonamide

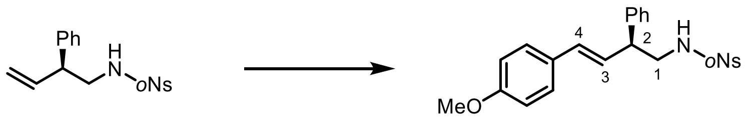

(+)-S15 **1o**

Prepared by **General Procedure C** using **(+)-S15** (73 mg, 0.23 mmol), 4-methoxystyrene (61 mg, 0.45 mmol) and Grubbs II (3 mg, 0.03 mmol) in PhMe at 100 ˚C. Flash column chromatography (15% → 50% Et_2O – pentane) afforded **(–)-1o** as a colourless oil (55 mg, 56%).

Data for **(–)-1o**: $\mathbf{R_f}$ 0.50 (75% Et_2O – pentane). $[\alpha]_D^{25}$ = –0.153 (*c* = 10.0, $CHCl_3$). **^{1}H NMR (400 MHz, $CDCl_3$) δ** 8.10 – 8.14 (1H, m, Ar), 7.65 – 7.79 (3H, m, Ar), 7.24 – 7.30 (2H, m, Ar), 7.17 – 7.24 (3H, m, Ar), 7.11 – 7.16 (2H, m, Ar), 6.81 (2H, d, *J* = 8.7 Hz, Ar), 6.34 (1H, d, *J* = 15.8 Hz, C4-H), 6.05 (1H, dd, *J* = 15.9, 7.9 Hz, C3-H), 5.40 (1H, t, *J* = 5.9 Hz, *N*H), 3.80 (OMe), 3.63 (1H, q, *J* = 7.6 Hz, C2-H), 3.47-3.52 (1H, m, C1-H_2). **^{13}C NMR (100 MHz, $CDCl_3$) δ** 159.5 (C Ar), 148.0 (C Ar), 140.4 (C Ar), 134.0 (C Ar), 133.5 (C Ar), 132.9 (C Ar), 132.1 (C4), 131.0 (C Ar), 129.4 (C Ar), 129.1 (2 × C Ar), 127.7 (2 × C Ar), 127.6 (2 × C Ar), 127.5 (C Ar), 126.8 (C3), 125.6 (C Ar), 114.1 (2 × C Ar), 55.4 (OMe), 49.0 (C2), 48.6 (C1)**. HRMS** (ESI): calculated for $C_{23}H_{22}N_2O_5SNa$ $[M+Na]^+$ requires *m/z* 461.1147, found *m/z* 461.1147. **IR** (film) ν_{max}: 3344, 2980, 2888, 1576, 1510, 1163 cm^{-1}.

Racemic (±**)-1o** was made *via* the same procedure using (±**)-S15** (56%).

(S16): (*E*)-5-(4-Methoxyphenyl)pent-4-en-1-ol

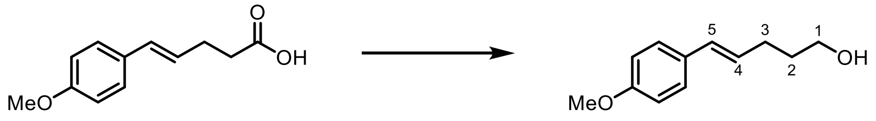

S1 **S16**

Prepared by **General Procedure F** using **S1** (1.00 g, 4.85 mmol) and $LiAlH_4$ (1.0 M in THF, 6.0 mL, 6.0 mmol). Flash column chromatography (50% Et_2O – pentane) afforded **S16** as a crystalline white solid (750 mg, 80%).

Data for **S16**: $\mathbf{R_f}$ 0.45 (50% Et_2O – pentane). **^{1}H NMR (400 MHz, $CDCl_3$) δ** 7.27 (2H, d, *J* = 8.6 Hz, Ar), 6.84 (2H, d, *J* = 8.8 Hz, Ar), 6.36 (1H, d, *J* = 15.7 Hz, C5-H), 6.09 (1H, dt, *J* = 15.8, 7.0 Hz, C4-H), 3.80 (3H, s, OMe), 3.70 (2H, t, *J* = 6.5 Hz, C1-H_2), 2.29 (2H, qd, *J* = 7.1, 1.5 Hz, C3-H_2), 1.82 – 1.65 (2H, m, C2-H_2), 1.58 – 1.56 (1H, m, *O*H). **^{13}C NMR (100 MHz, $CDCl_3$) δ** 158.9 (C Ar), 130.6 (C Ar), 129.9 (C5), 128.0 (C4), 127.2 (2 × C Ar), 114.1 (2 × C Ar), 62.6 (C1), 55.4 (OMe), 32.5 (C2), 29.4 (C3). *The spectroscopic properties were consistent with the data available in the literature.*[14]

(S17): (*E*)-5-(4-Methoxyphenyl)pent-4-en-1-yl methanesulfonate

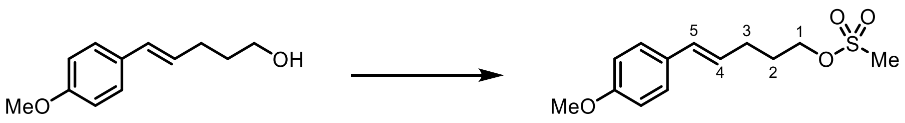

S16 **S17**

Prepared by **General Procedure G** using **S16** (711 mg, 3.70 mmol), triethylamine (0.77 mL, 5.6 mmol) and methanesulfonic anhydride (805 mg, 4.62 mmol). Flash column chromatography (30% Et_2O – pentane) afforded **S17** as a crystalline white solid (730 mg, 73%).

Data for **S17**: $\mathbf{R_f}$ 0.42 (50% Et_2O – pentane). **^{1}H NMR (400 MHz, $CDCl_3$) δ** 7.27 (2H, d, *J* = 8.9 Hz, Ar), 6.84 (2H, d, *J* = 8.8 Hz, Ar), 6.38 (1H, d, *J* = 15.8 Hz, C5-H), 6.02 (1H, dt, *J* = 15.8, 7.0 Hz, C4-H), 4.28 (2H, t, *J* = 6.4 Hz, C1-H_2), 3.80 (3H, s, OMe), 3.00 (3H, s, SO_2Me), 2.33 (2H, *app.* qd, *J* = 7.3, 1.5 Hz, C3-H_2), 1.93 (2H, *app.* dq, *J* = 7.8, 6.5 Hz, C2-H_2). **^{13}C NMR (100 MHz, $CDCl_3$) δ** 159.1 (C Ar), 131.0 (C5), 130.2 (C Ar), 127.3 (2 × C Ar), 126.2 (C4), 114.1 (2 × C Ar), 69.4 (C1), 55.4 (OMe), 37.5 (SO_2Me), 29.0 (C2), 28.9 (C3). *The spectroscopic properties were consistent with the data available in the literature.*[15]

(4-Ts): (*E*)-*N*-(5-(4-Methoxyphenyl)pent-4-en-1-yl)-4-methylbenzenesulfonamide

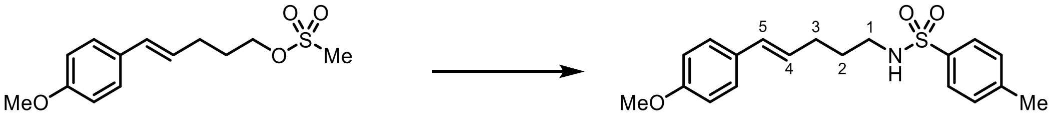

S17 **4-Ts**

Prepared by **General Procedure H** using **S17** (100 mg, 0.370 mmol), *p*-toluenesulfonamide (94 mg, 0.55 mmol) and potassium hydroxide (31 mg, 0.55 mmol). Flash column chromatography (25% EtOAc – pentane) afforded **4-Ts** as a white solid (82 mg, 64%).

Data for **4-Ts**: $\mathbf{R_f}$ 0.29 (20% EtOAc – pentane). **^{1}H NMR (400 MHz, $CDCl_3$)** 7.75 (2H, d, *J* = 8.3 Hz, Ar), 7.28 (2H, d, *J* = 7.9 Hz, Ar), 7.22 (2H, d, *J* = 8.6 Hz, Ar), 6.82 (2H, d, *J* = 8.7 Hz, Ar), 6.26 (1H, d, *J* = 15.8 Hz, C5-H), 5.93 (1H, dt, *J* = 15.8, 6.9 Hz, C4-H), 4.71 (1H, t, *J* = 6.2 Hz, *N*H), 3.80 (3H, s, OMe), 3.03 – 2.93 (2H, m, C1-H_2), 2.41 (3H, s, TsMe), 2.17 (2H, *app.* qd, *J* = 7.1, 1.5 Hz, C3-H_2), 1.63 (2H, p, *J* = 7.1 Hz, C2-H_2). **^{13}C NMR (100 MHz, $CDCl_3$) δ** 159.0 (C Ar), 143.5 (C Ar), 137.1 (C Ar), 130.4 (C5), 130.3 (C Ar), 129.8 (2 × C Ar), 127.2 (2 × C Ar), 127.2 (2 × C Ar), 126.8 (C4), 114.0 (2 × C Ar), 55.4 (OMe), 42.7 (C1), 29.9 (C3), 29.4 (C2), 21.6 (TsMe). *The spectroscopic properties were consistent with those reported in the literature.*[16]

(4-Ms): (*E*)-*N*-(5-(4-Methoxyphenyl)pent-4-en-1-yl)methanesulfonamide

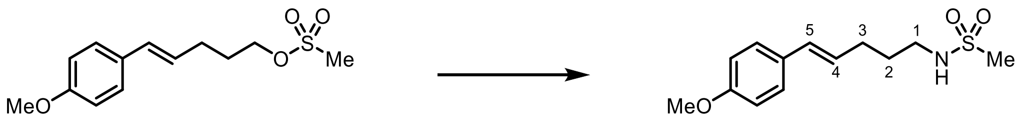

S17 **4-Ms**

Prepared by **General Procedure H** using **S17** (100 mg, 0.370 mmol), methanesulfonamide (53 mg, 0.55 mmol) and potassium hydroxide (31 mg, 0.55 mmol). Flash column chromatography (25% EtOAc – pentane) afforded **4-Ms** as a white solid (53 mg, 53%).

Data for **4-Ms**: $\mathbf{R_f}$ 0.31 (20% EtOAc – pentane). **^{1}H NMR (400 MHz, $CDCl_3$) δ** 7.28 (2H, d, *J* = 8.8 Hz, Ar), 6.85 (2H, d, *J* = 8.7 Hz, Ar), 6.37 (1H, d, *J* = 15.8 Hz, C5-H), 6.03 (1H, dt, *J* = 15.7, 7.0 Hz, C4-H), 4.40 (1H, t, *J* = 6.3 Hz, *N*H), 3.81 (3H, s, OMe), 3.19 (2H, q, *J* = 6.8 Hz, C1-H_2), 2.96 (s, 3H, SO_2Me), 2.34 – 2.23 (2H, m, C3-H_2), 1.76 (p, *J* = 7.2 Hz, C2-H_2). **^{13}C NMR (100 MHz, $CDCl_3$) δ** 158.9 (C Ar), 130.5 (C Ar), 130.2 (C5), 127.1 (2 × C Ar), 126.6 (C4), 114.0 (2 × C Ar), 55.3 (OMe), 42.8 (C1), 40.4 (SO_2Me), 29.9 (C3), 29.9 (C2). **HRMS** (ESI): calculated for $C_{13}H_{19}O_3NNaS$ $[M+Na]^+$ requires m/z 292.0978, found m/z 292.0978. **IR** (film) ν_{max}: 3239, 2940, 2360, 1511, 1302, 1247, 1161, 1140, 1063, 1024 cm^{-1}. **mp** (90 – 92 °C, EtOAc).

(4-*o*Ns): (*E*)-*N*-(5-(4-Methoxyphenyl)pent-4-en-1-yl)-2-nitrobenzenesulfonamide

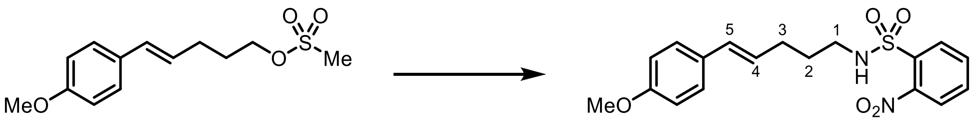

S17 **4-*o*Ns**

Prepared by **General Procedure H** using **S17** (200 mg, 0.740 mmol), 2-nitrobenzenesulfonamide (224 mg, 1.11 mmol) and potassium hydroxide (62 mg, 1.1 mmol). Flash column chromatography (25% EtOAc – pentane) afforded **4-*o*Ns** as a white solid (169 mg, 61%).

Data for **4-*o*Ns**: $\mathbf{R_f}$ 0.21 (20% EtOAc – pentane). **^{1}H NMR (400 MHz, $CDCl_3$) δ** 8.21 – 7.99 (1H, m, Ar), 7.88 – 7.79 (1H, m, Ar), 7.77 – 7.62 (2H, m, Ar), 7.22 (2H, d, *J* = 8.6 Hz, Ar), 6.83 (2H, d, *J* = 8.7 Hz, Ar), 6.29 (1H, d, *J* = 15.9 Hz, C5-H), 5.94 (1H, dt, *J* = 15.8, 7.0 Hz, C4-H), 5.30 (1H, t, *J* = 6.1 Hz, *N*H), 3.80 (3H, s, OMe), 3.15 (2H, td, *J* = 7.0, 6.1 Hz, C1-H_2), 2.23 (2H, *app.* qd, *J* = 7.1, 1.5 Hz, C3-H_2), 1.71 (2H, p, *J* = 7.1 Hz, C2-H_2). **^{13}C NMR (101 MHz, $CDCl_3$) δ** 159.0 (C Ar), 133.6 (C Ar), 132.9 (C Ar), 131.2 (C Ar), 130.7 (C5), 130.2 (C Ar), 127.2 (2 × C Ar), 126.5 (C4), 125.5 (C Ar), 114.1 (2 × C Ar), 55.4 (OMe), 43.3 (C1), 29.9 (C3), 29.4 (C2). **HRMS** (ESI): calculated for $C_{18}H_{21}O_5NS$ $[M+H]^+$ requires m/z 377.1166, found m/z 377.1170. **IR** (film) ν_{max}: 3265, 2981, 2361, 2341, 1670, 1606, 1539, 1510, 1464, 1440, 1363, 1336, 1298, 1247, 1163 cm^{-1}. **mp** (86 – 88 °C, EtOAc).

(4-*p*Ns): (*E*)-*N*-(5-(4-Methoxyphenyl)pent-4-en-1-yl)-4-nitrobenzenesulfonamide

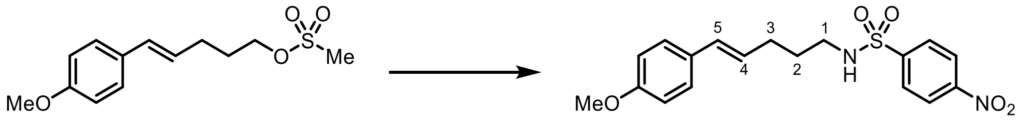

S17 **4-*p*Ns**

Prepared by **General Procedure H** using **S17** (250 mg, 0.920 mmol), 4-nitrobenzenesulfonamide (281 mg, 1.39 mmol) and potassium hydroxide (78 mg, 1.4 mmol). Flash column chromatography (25% EtOAc – pentane) afforded **4-*p*Ns** as a white solid (308 mg, 89%).

Data for **4-*p*Ns**: **R_f** 0.21 (20% EtOAc – pentane). **^{1}H NMR (400 MHz, $CDCl_3$) δ** 8.38 – 8.28 (2H, m, Ar), 8.08 – 7.99 (2H, m, Ar), 7.26 – 7.17 (2H, m, Ar), 6.90 – 6.79 (2H, m, Ar), 6.27 (1H, d, *J* = 15.7 Hz, C5-H), 5.92 (1H, dt, *J* = 15.8, 7.0 Hz, C4-H), 4.58 (1H, t, *J* = 6.1 Hz, *N*H), 3.81 (3H, s, OMe), 3.07 (2H, q, *J* = 6.7 Hz, C1-H_2), 2.21 (2H, *app.* qd, *J* = 7.1, 1.4 Hz, C3-H_2), 1.68 (2H, p, *J* = 7.1 Hz, C2-H_2). **^{13}C NMR (100 MHz, $CDCl_3$) δ** 159.2 (C Ar), 146.1 (C Ar), 130.9 (C5), 130.0 (C Ar), 128.4 (2 × C Ar), 127.2 (2 × C Ar), 126.2 (C4), 124.5 (2 × C Ar), 114.2 (2 × C Ar), 55.5 (OMe), 42.9 (C1), 29.9 (C3), 29.4 (C2). *The spectroscopic properties were consistent with the data available in the literature.*[17]

(S18): (*E*)-5-(4-Methoxyphenyl)pent-4-enamide

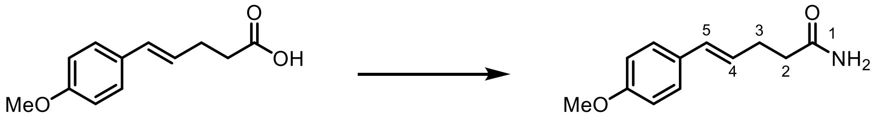

S1 **S18**

Prepared by **General Procedure I** using **S1** (1.67 g, 8.10 mmol) and oxalyl chloride (0.81 mL, 9.6 mmol). Recrystallisation from hot EtOAc afforded **S18** as a crystalline white solid (784 mg, 47%).

Data for **S18**: **R_f** 0.65 (5% MeOH – CH_2Cl_2). **^{1}H NMR (400 MHz, $CDCl_3$) δ** 7.27 (2H, d, *J* = 8.8 Hz, Ar), 6.83 (2H, d, *J* = 8.8 Hz, Ar), 6.40 (1H, d, *J* = 15.9 Hz, C5-H), 6.07 (1H, dt, *J* = 15.8, 6.9 Hz, C4-H), 5.50 (2H, *br.* s, NH_2), 3.80 (3H, s, OMe), 2.53 (2H, *app.* q, *J* = 6.6, C3-H_2), 2.45 – 2.32 (2H, m, C2-H_2). **^{13}C NMR (100 MHz, $CDCl_3$) δ** 174.7 (C1), 159.1 (C Ar), 130.7 (C5), 130.2 (C Ar), 127.3 (C4), 126.4 (2 × C Ar), 114.1 (2 × C Ar), 55.4 (OMe), 35.8 (C2), 28.8 (C3). **HRMS** (ESI): calculated for $C_{12}H_{16}ON$ $[M+H]^+$ requires m/z 206.1176, found m/z 206.1176. **IR** (film) ν_{max}: 3386, 3191, 1648, 1604, 1510, 1440, 1282, 1174, 1029, 968, 847, 807 cm^{-1}. **mp** (165 – 167 °C, EtOAc).

(4-Cbz): Benzyl (*E*)-(5-(4-methoxyphenyl)pent-4-en-1-yl)carbamate

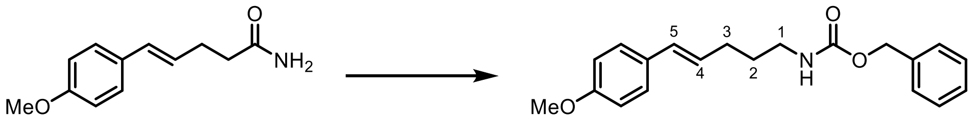

S18 **4-Cbz**

Prepared by **General Procedure J** using **S8** (120 mg, 0.600 mmol) and $LiAlH_4$ (33 mg, 0.90 mmol). Crude amine (80 mg, 0.42 mmol), triethylamine (0.07 mL, 0.5 mmol) and benzyl chloroformate (0.07 mL, 0.5 mmol). Flash column chromatography (25% EtOAc – pentane) afforded **4-Cbz** as a white solid (107 mg, 73%).

Data for **4-Cbz**: **R_f** 0.30 (20% EtOAc – pentane). **^{1}H NMR (400 MHz, $CDCl_3$) δ** 7.43 – 7.20 (5H, m, Ar), 7.28 – 7.19 (2H, m, Ar), 6.83 (2H, d, *J* = 8.8 Hz, Ar), 6.34 (1H, d, *J* = 15.8 Hz, C5-H), 6.04 (1H, dt, *J* = 15.5, 6.9 Hz, C4-H), 5.10 (2H, s, CH_2Ph), 4.77 (1H, *br.* s, *N*H), 3.80 (3H, s, OMe), 3.25 (2H, q, *J* = 6.7 Hz, C1-H_2), 2.31 – 2.15 (2H, m, C3-H_2), 1.68 (2H, p, *J* = 7.2 Hz, C2-H_2). **^{13}C NMR (101 MHz, $CDCl_3$) δ** 158.9 (C6), 136.8 (C Ar), 130.5 (C Ar), 130.1 (C5), 128.7 (2 × C Ar), 128.3 (C Ar), 128.3 (2 × C Ar), 127.5 (C Ar), 127.2 (2 × C Ar), 114.1 (2 × C Ar), 66.8 (CH_2Ph), 55.4 (OMe), 40.8 (C1), 30.3 (C2), 29.9 (C3). **HRMS** (ESI): calculated for $C_{20}H_{23}O_3NNa$ $[M+Na]^+$ requires m/z 348.1570, found m/z 348.1570. **IR** (film) ν_{max}: 3328, 2970, 2359, 1686, 1542, 1510, 1247, 1231, 1175, 1143 cm^{-1}. **mp** (68 – 70 °C, EtOAc).

(4a): Methyl (*E*)-(5-(4-methoxyphenyl)pent-4-en-1-yl)carbamate

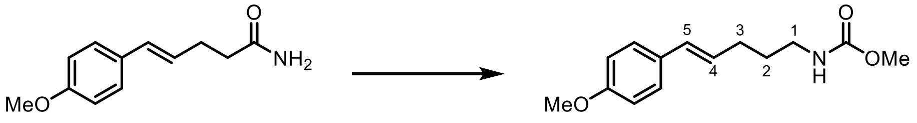

S18 **4a**

Prepared by **General Procedure J** using **S18** (726 mg, 3.54 mmol) and $LiAlH_4$ (201 mg, 3.51 mmol). Crude amine (411 mg, 2.15 mmol), triethylamine (0.37 mL, 2.7 mmol) and methyl chloroformate (0.21 mL, 2.7 mmol). Flash column chromatography (25% EtOAc – pentane) afforded **4a** as a white solid (418 mg, 78%).

Data for **4a**: **R_f** 0.16 (20% EtOAc – pentane). **^{1}H NMR (400 MHz, $CDCl_3$) δ** 7.26 (2H, d, *J* = 8.6 Hz, Ar), 6.83 (2H, d, *J* = 8.7 Hz, Ar), 6.34 (1H, d, *J* = 15.8 Hz, C5-H), 6.04 (1H, dt, *J* = 15.8, 6.9 Hz, C4-H), 4.69 (1H, *br.* s, *N*H), 3.80 (3H, s, OMe), 3.66 (3H, s, OMe), 3.23 (2H, q, *J* = 6.8 Hz, C1-H_2), 2.28 – 2.17 (2H, m, C3-H_2), 1.67 (2H, p, *J* = 7.2 Hz, C2-H_2). **^{13}C NMR (101 MHz, $CDCl_3$) δ** 158.6 (CO), 130.5 (C Ar), 130.1 (C5), 127.5 (C4), 127.2 (2 × C Ar), 114.1 (2 × C Ar), 55.4 (OMe), 52.2 (OMe), 40.8 (C1), 30.3 (C3), 29.9 (C2). **HRMS** (ESI): calculated for $C_{14}H_{19}O_3NNa$ $[M+Na]^+$ requires m/z 272.1257,

found m/z 272.1526. **IR** (film) ν_{max}: 3327, 2929, 1688, 1607, 2545, 1511, 1271, 1251, 1176, 1030 cm^{-1}. **mp** (74 – 76 °C, EtOAc).

(S19): (*E*)-5-Phenylpent-4-enoic acid

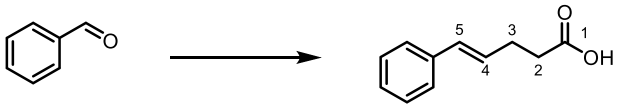

S19

Prepared by **General Procedure A** using benzaldehyde (1.38 mL, 13.6 mmol), (3-carboxypropyl)triphenylphosphonium bromide (7.00 g, 16.3 mmol), NaHMDS (2.0 M in THF, 16.3 mL, 32.6 mmol). Flash column chromatography (80% Et_2O – pentane + 1% acetic acid) followed by trituration with ice cold hexane (3 × 10 mL) afforded **S19** as a crystalline white solid (1.52 g, 63%).

Data for **S19**: **R_f** 0.54 (20% EtOAc – pentane). **^{1}H NMR (400 MHz, $CDCl_3$) δ** 7.39 – 7.28 (4H, m, Ar), 7.22 (1H, m, Ar), 6.46 (1H, d, *J* = 16.0 Hz, C5-H), 6.24 (1H, m, C4-H), 2.61-2.49 (4H, m, C2-H_2 + C3-H_2). **^{13}C NMR (101 MHz, $CDCl_3$) δ** 179.2 (C1) 137.3 (C Ar), 131.2 (C5), 128.5 (2 × C Ar), 128.0 (C4), 127.2 (2 × C Ar), 126.1 (C Ar), 33.8 (C2), 27.9 (C3). *The spectroscopic properties were consistent with the data available in the literature.*[18]

(S20): (*E*)-5-Phenylpent-4-enamide

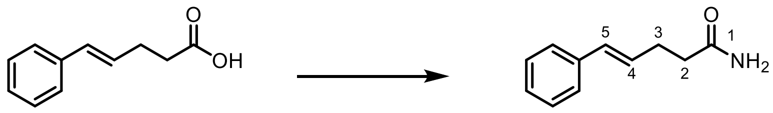

S19 **S20**

Prepared by **General Procedure I** using **S19** (500 mg, 2.84 mmol) and oxalyl chloride (0.29 mL, 3.4 mmol). Recrystallisation from hot EtOAc afforded **S20** as a crystalline white solid (350 mg, 70%).

Data for **S20**: **R_f** 0.59 (10% MeOH – CH_2Cl_2). **^{1}H NMR (400 MHz, $CDCl_3$)** δ 7.37 – 7.26 (4H, m, Ar), 7.24 – 7.17 (1H, m, Ar), 6.46 (1H, dt, *J* = 15.7, 1.5 Hz, C5-H), 6.22 (1H, dt, *J* = 15.8, 6.9 Hz, C4-H), 5.50 (2H, *br.* s, *N*H_2), 2.75 – 2.52 (2H, m, C3-H_2), 2.47 – 2.34 (2H, m, C2-H_2). **^{13}C NMR (100 MHz, $CDCl_3$) δ** 174.8 (C1), 137.4 (C Ar), 131.3 (C5), 128.7 (C4 + 2 × C Ar), 127.3 (C Ar), 126.2 (2 × C Ar), 35.6 (C2), 28.8 (C3). *The spectroscopic properties were consistent with the data available in the literature.*[16]

(4b): Methyl (*E*)-(5-phenylpent-4-en-1-yl)carbamate

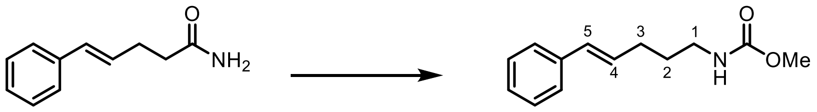

S20 **4b**

Prepared by **General Procedure J** using **S20** (300 mg, 1.71 mmol) and $LiAlH_4$ (97 mg, 2.6 mmol). Crude amine (270 mg, 1.67 mmol), triethylamine (0.29 mL, 2.1 mmol) and methyl chloroformate (0.16 mL, 2.1 mmol). Flash column chromatography (25% EtOAc – pentane) afforded **4b** as a yellow oil (112 mg, 30%).

Data for **4b**: $\mathbf{R_f}$ 0.43 (20% EtOAc – pentane). **^{1}H NMR (400 MHz, $CDCl_3$) δ** 7.37 – 7.25 (4H, m, Ar), 7.24 – 7.17 (1H, m, Ar), 6.41 (1H, d, *J* = 15.8, C5-H), 6.19 (1H, dt, *J* = 15.8, 6.9 Hz, C4-H), 4.85 (1H, *br*. s, *N*H), 3.66 (3H, s, C7-H_3), 3.23 (2H, q, *J* = 6.8 Hz, C1-H_2), 2.33 – 2.08 (2H, m, C3-H_2), 1.68 (2H, p, *J* = 7.3 Hz, C2-H_2). **^{13}C NMR (101 MHz, $CDCl_3$) δ** 157.2 (C6), 137.6 (C Ar), 130.6 (C5), 129.6 (C4), 128.6 (2 × C Ar), 127.1 (C Ar), 126.0 (2 × C Ar), 52.1 (C7), 40.7 (C1), 30.2 (C3), 29.7 (C2). **HRMS** (ESI): calculated for $C_{13}H_{18}O_2N$ $[M+H]^+$ requires m/z 220.1332, found m/z 220.1333. **IR** (film) ν_{max}: 3650, 3338, 3959, 3024, 2981, 2889, 2360, 2339, 1699, 1531, 1253, 966 cm^{-1}.

(S21): (*E*)-5-Phenylpent-4-en-1-ol

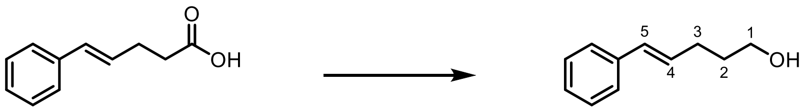

S19 **S21**

Prepared by **General Procedure F** using **S19** (1.00 g, 5.67 mmol) and $LiAlH_4$ (1.0 M $LiAlH_4$ in THF, 6.8 mL, 6.8 mmol). Flash column chromatography (50% Et_2O – pentane) afforded **S21** as a colourless liquid (799 mg, 87%).

Data for **S21**: $\mathbf{R_f}$ 0.45 (50% Et_2O – pentane). **^{1}H NMR (400 MHz, $CDCl_3$) δ** 7.40 – 7.28 (4H, m, Ar), 7.22 (1H, m, 1H, Ar), 6.44 (1H, d, *J* = 15.7 Hz, C5-H), 6.25 (1H, dt, *J* = 15.8, 7.0 Hz, C4-H), 3.71 (2H, t, *J* = 6.5 Hz, C1-H_2), 2.40 – 2.24 (2H, m, C3-H_2), 1.76 (2H, *app*. dq, *J* = 9.5, 6.5 Hz, C2-H_2). **^{13}C NMR (100 MHz, $CDCl_3$) δ** 137.6 (C Ar), 130.3 (C5), 130.0 (C4), 128.4 (2 × C Ar), 126.9 (2 × C Ar), 125.9 (Ar), 62.28 (C1), 32.2 (C2), 29.3 (C3). *The spectroscopic properties were consistent with the data available in the literature.*[19]

(S22): (*E*)-5-Phenylpent-4-en-1-yl methanesulfonate

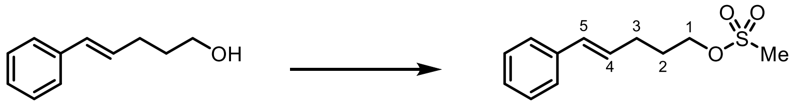

S21 **S22**

Prepared by **General Procedure G** using **S21** (500 mg, 3.08 mmol), triethylamine (0.64 mL, 4.6 mmol) and methanesulfonic anhydride (644 mg, 3.70 mmol). Flash column chromatography (30% Et_2O – pentane) afforded **S22** as a colourless liquid (600 mg, 81%).

Data for **S22**: **R_f** 0.42 (50% Et_2O – pentane). **^{1}H NMR (400 MHz, $CDCl_3$) δ** 7.40 – 7.28 (4H, m, Ar), 7.22 (1H, m, Ar), 6.45 (1H, d, *J* = 15.8 Hz, C5-H), 6.18 (1H, dt, *J* = 15.8, 7.0 Hz, C4-H), 4.28 (2H, t, *J* = 6.4 Hz, C1-H_2), 3.00 (3H, s, SO_2Me) 2.36 (2H, dt, *J* = 7.3, 7.0 Hz, C3-H_2), 1.99 – 1.89 (2H, m, C2-H_2). **^{13}C NMR (100 MHz, $CDCl_3$)** δ 137.2 (C Ar), 131.4 (C5), 128.5 (2 × C Ar), 128.2 (2 × C Ar), 127.2 (C4), 126.0 (C Ar), 69.2 (C1), 37.3 (SO_2Me), 28.7 (C2), 28.7 (C3). *The spectroscopic properties were consistent with the data available in the literature.*[15]

(4b-Ns): (*E*)-2-Nitro-*N*-(5-phenylpent-4-en-1-yl)benzenesulfonamide

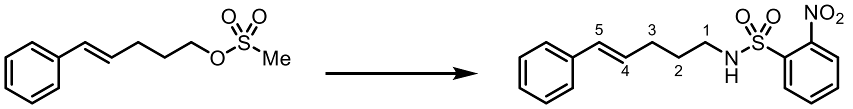

S22 **4b-Ns**

Prepared by **General Procedure H** using **S22** (1.20 g, 5.00 mmol), 2-nitrobenzenesulfonamide (1.52 g, 7.50 mmol) and potassium hydroxide (421 mg, 7.50 mmol). Flash column chromatography (25% EtOAc – pentane) afforded **4b-Ns** as a white solid (902 mg, 52%).

Data for **4b-Ns**: **R_f** 0.21 (20% EtOAc – pentane). **^{1}H NMR (400 MHz, $CDCl_3$) δ** 8.12 (1H, m, Ar), 7.82 (1H, m, Ar), 7.76 – 7.67 (2H, m, Ar), 7.31 – 7.27 (4H, m, Ar), 7.21 (1H, m, Ar), 6.35 (1H, d, *J* = 15.7 Hz , C5-H), 6.10 (1H, dt, *J* = 15.7, 7.0 Hz, C4-H), 5.32 (1H, t, *J* = 6.1 Hz, *N*H), 3.16 (2H, q, *J* = 6.1, 5.5 Hz, C1-H_2), 2.26 (2H, *app.* qd, *J* = 7.1, 1.5 Hz, C3-H_2), 1.73 (2H, p, *J* = 7.0 Hz, C2-H_2). **^{13}C NMR (100 MHz, $CDCl_3$) δ** 148.1 (C Ar), 137.3 (C Ar), 133.7 (C Ar), 133.5 (C Ar), 132.7 (C Ar), 131.2 (C5), 131.0 (C Ar), 128.6 (C4), 128.5 (2 × C Ar), 127.1 (C Ar), 126.0 (2 × C Ar), 125.3 (C Ar), 43.1 (C1), 29.7 (C3), 29.1 (C2). **HRMS** (ESI): calculated for $C_{17}H_{19}O_4N_2S$ $[M+H]^+$ requires m/z 347.1060, found m/z 347.1062. **IR** (film) ν_{max}: 3344, 3025, 2925, 1538, 1412, 1342, 1165, 740 cm^{-1}. **mp** (92 – 94 °C, EtOAc).

(S23): Pent-4-enamide

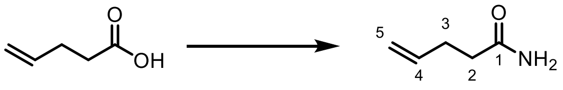

S23

Prepared by **General Procedure I** using pent-4-enoic acid (2.04 mL, 20.0 mmol) and oxalyl chloride (2.04 mL, 24.0 mmol). Recrystallisation from hot EtOAc afforded **S23** as a crystalline white solid (780 mg, 39%).

Data for **S23**: **R_f** 0.62 (10% MeOH – CH_2Cl_2). **^{1}H NMR (400 MHz, $CDCl_3$) δ** 6.13 – 5.42 (2H, *br.* s, *N*H_2), 5.83 (1H, *app.* dddd, *J* = 16.6, 12.4, 8.0, 5.1 Hz, C4-H), 5.17 – 4.90 (2H, m, C5-H_2), 2.43 – 2.26 (4H, m, 2 × C2-H_2 + 2 × C3-H_2).**^{13}C NMR (101 MHz, $CDCl_3$) δ** 175.3 (C1), 137.0 (C4), 115.8 (C5), 35.1 (C2), 29.4 (C3). *The spectroscopic properties were consistent with the data available in the literature.*[20]

(S24): Methyl pent-4-en-1-ylcarbamate

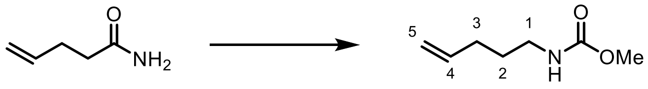

S23 **S24**

Prepared by **General Procedure J** *(with modification)* using **S23** (1.00 g, 10.0 mmol) and $LiAlH_4$ (574 mg, 15.0 mmol). Crude amine hydrochloride salt (787 mg, 6.47 mmol), triethylamine (2.71 mL, 19.4 mmol) and methyl chloroformate (0.63 mL, 8.1 mmol). Flash column chromatography (25% EtOAc – pentane) afforded **S24** as a colourless oil (450 mg, 31%).

Data for **S24**: **R_f** 0.50 (20% EtOAc – pentane). **^{1}H NMR (400 MHz, $CDCl_3$)** δ 5.76 (1H, ddt, *J* = 16.9, 10.2, 6.6 Hz, C4-H), 5.00 (1H, dq, *J* = 17.1, 1.7 Hz, C5-H_A), 4.95 (1H, dq, *J* = 10.2, 1.4 Hz, C5-H_B), 4.82 (1H, *br.* s, *N*H), 3.62 (3H, s, OMe), 3.15 (2H, q, *J* = 6.8 Hz, C1-H_2), 2.05 (2H, td, *J* = 8.0, 6.0 Hz, C3-H_2), 1.57 (2H, p, *J* = 7.3 Hz, C2-H_2).**^{13}C NMR (101 MHz, $CDCl_3$) δ** 157.2 (CO), 137.8 (C4), 115.2 (C5), 52.0 (OMe), 40.6 (C1), 31.0 (C3), 29.2 (C2). **HRMS** (ESI): calculated for $C_7H_{14}O_2N$ $[M+H]^+$ requires m/z 144.1019, found m/z 144.1018. **IR** (film) ν_{max}: 3649, 3331, 3078, 2980, 2359, 2342, 1697, 1536, 1449, 1256 cm^{-1}.

(4c): Methyl (*E*)-(5-(4-bromophenyl)pent-4-en-1-yl)carbamate

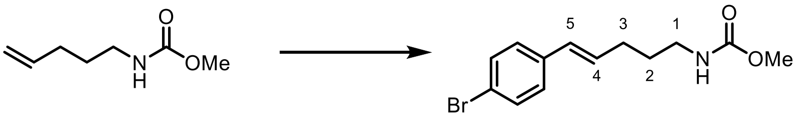

S24 **4c**

Prepared by **General Procedure C** using **S24** (200 mg, 1.40 mmol), 4-bromostyrene (0.37 mL, 2.80 mmol), Grubbs II (58 mg, 0.070 mmol) in PhMe (5 mL). Flash column chromatography (gradient elution 0% → 15% EtOAc – pentane) followed by recrystallisation from hot EtOAc afforded **4c** as a white solid (91 mg, 22%).

Data for **4c**: $\mathbf{R_f}$ 0.38 (20% EtOAc – pentane). **^{1}H NMR (400 MHz, $CDCl_3$) δ** 7.48 – 7.34 (2H, m, Ar), 7.25 – 7.06 (2H, m, Ar), 6.34 (1H, d, *J* = 15.8 Hz, C5-H), 6.18 (1H, dt, *J* = 15.9, 6.8 Hz, C4-H), 4.69 (1H, *br.* s, *N*H), 3.66 (3H, s, OMe), 3.23 (2H, q, *J* = 6.8 Hz, C1-H_2), 2.40 – 2.10 (2H, m, C3-H_2), 1.68 (2H, p, *J* = 7.3 Hz, C2-H_2). **^{13}C NMR (101 MHz, $CDCl_3$) δ** 157.2 (CO), 136.6 (C Ar), 131.7 (2 × C Ar), 130.6 (C5), 129.6 (C4), 127.6 (2 × C Ar), 120.8 (C Ar), 52.2 (OMe), 40.7 (C1), 30.3 (C3), 29.7 (C2). **HRMS** (ESI): calculated for $C_{13}H_{17}O_2N^{78}BrNa$ $[M+Na]^+$ requires m/z 336.0206, found m/z 336.0205. **IR** (film) ν_{max}: 3650, 3338, 2981, 2886, 2360, 2336, 1690, 1538, 1487, 1461 cm^{-1}. **mp** (78 – 80 °C, EtOAc).

(4d): Methyl ((4*E*,6*E*)-7-(4-methoxyphenyl)hepta-4,6-dien-1-yl)carbamate

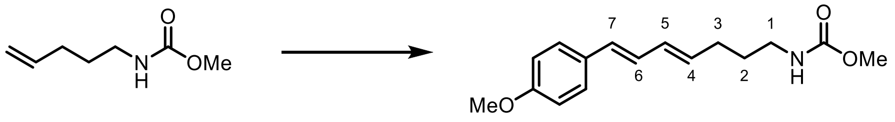

S24 **4d**

Prepared by **General Procedure C** using **S24** (400 mg, 2.86 mmol), **S9** (918 mg, 5.73 mmol), Grubbs I (115 mg, 0.140 mmol) in PhMe (12 mL) at 100 °C. Flash column chromatography (gradient elution 0% → 15% EtOAc – pentane) followed by recrystallisation from hot EtOAc afforded **4d** as a crystalline white solid (125 mg, 16%).

Data for **4d**: $\mathbf{R_f}$ 0.43 (20% EtOAc – pentane). **^{1}H NMR (400 MHz, $CDCl_3$) δ** 7.31 (2H, d, *J* = 8.7 Hz, Ar), 6.84 (2H, d, *J* = 8.8 Hz, Ar), 6.61 (1H, dd, *J* = 15.7, 10.3 Hz, C6-H), 6.40 (1H, d, *J* = 15.7 Hz, C7-H), 6.19 (1H, dd, *J* = 14.0, 10.3 Hz, C5-H), 5.73 (1H, dt, *J* = 14.7, 7.0 Hz, C4-H), 4.69 (1H, *br.* s, *N*H), 3.80 (3H, s, OMe), 3.66 (3H, s, OMe), 3.21 (2H, q, *J* = 7.0 Hz, C1-H_2), 2.17 (2H, td, *J* = 7.3, 1.5 Hz, C3-H_2), 1.63 (2H, p, *J* = 7.3 Hz, C2-H_2).**^{13}C NMR (101 MHz, $CDCl_3$) δ** 159.2 (CO), 133.0 (C4), 131.6 (C5), 130.5 (C Ar), 130.3 (C7), 127.5 (2 × C Ar), 127.2 (C6), 114.2 (2 × C Ar), 55.4 (OMe), 52.1 (OMe), 40.7 (C1), 30.1 (C3), 29.8 (C2). **HRMS** (ESI): calculated for $C_{16}H_{22}O_3N$ $[M+H]^+$ requires

m/z 276.1594, found m/z 276.1596. **IR** (film) ν_{max}: 3331, 3015, 2363, 1692, 1602, 1552, 1510, 1284, 1254, 1030, 985, 834 cm^{-1}. **mp** (105 – 107 °C, EtOAc).

(S25): 5-Methylhex-4-enoic acid

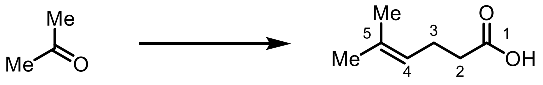

S25

Prepared by **General Procedure A** using acetone (0.73 mL, 10 mmol), (3-carboxypropyl)triphenylphosphonium bromide (5.15 g, 12.0 mmol), NaHMDS (2.0 M in THF, 12.0 mL, 24.0 mmol). Flash column chromatography (80% Et_2O/pentane + 1% acetic acid) afforded **S25** as a yellow oil (903 mg, 70%).

Data for **S25**: **R_f** 0.54 (20% EtOAc – pentane). **^{1}H NMR (400 MHz, $CDCl_3$) δ** 5.01 (1H, m, C4-H), 2.44 – 2.13 (4H, m, 2 × C2-H_2 + 2 × C3-H_2), 1.60 (3H, d, *J* = 1.3 Hz, Me), 1.53 (3H, d, *J* = 1.3 Hz, Me). **^{13}C NMR (101 MHz, $CDCl_3$) δ** 179.2 (C1), 133.6 (C5), 122.2 (C4), 34.3 (C2), 25.8 (C3), 23.5 (Me), 17.8 (Me). *The spectroscopic properties were consistent with the data available in the literature.*[21]

(S26): 5-Methylhex-4-enamide

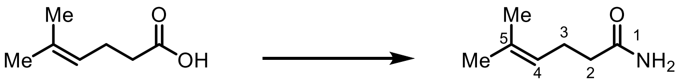

S25 **S26**

Prepared by **General Procedure I** using **S25** (500 mg, 3.90 mmol) and oxalyl chloride (0.40 mL, 4.70 mmol). Recrystallisation from hot EtOAc afforded **S26** as a crystalline white solid (418 mg, 84%).

Data for **S26**: **R_f** 0.54 (10% MeOH, CH_2Cl_2). **^{1}H NMR (400 MHz, $CDCl_3$) δ** 6.08 (1H, *br.* s, *N*H), 5.69 (1H, *br.* s, *N*H), 5.15 – 5.05 (1H, m, C4-H), 2.33 – 2.25 (2H, m, C3-H_2), 2.25 – 2.19 (2H, m, C2-H_2), 1.67 (3H, s, Me), 1.60 (3H, s, Me). **^{13}C NMR (100 MHz, $CDCl_3$)** 175.9 (C1), 133.5 (C5), 122.8 (C4), 36.1 (C2), 25.9 (Me), 24.3 (C3), 17.9 (Me). **HRMS** (ESI): calculated for $C_7H_{14}ON$ $[M+H]^+$ requires m/z 128.1070, found m/z 128.1070. **IR** (film) ν_{max}: 3366, 3186, 2969, 1678, 1657, 1452, 1411, 1318, 952, 817 cm^{-1}. **mp** (87 – 89 °C, EtOAc).

(4e): Methyl (5-methylhex-4-en-1-yl) carbamate

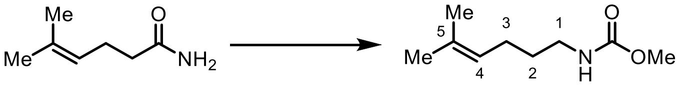

S26 **4e**

Prepared by **General Procedure J** *(with modification)* using **S26** (300 mg, 2.37 mmol) and $LiAlH_4$ (574 mg, 3.56 mmol). Crude amine hydrochloride salt (208 mg, 1.39 mmol), triethylamine (0.58 mL, 4.2 mmol) and methyl chloroformate (0.13 mL, 1.7 mmol). Flash column chromatography (25% EtOAc – pentane) afforded **4e** as a colourless oil (90 mg, 27%).

Data for **4e**: $\mathbf{R_f}$ 0.38 (20% EtOAc – pentane). **^{1}H NMR (400 MHz, $CDCl_3$)** δ 5.07 (1H, *app.* dddd, *J* = 7.2, 5.7, 2.9, 1.5 Hz, C4-H), 4.73 (1H, *br.* s, *N*H), 3.63 (3H, s, C8-H_3), 3.14 (2H, q, *J* = 6.7 Hz, C1-H_2), 1.99 (2H, q, *J* = 7.4 Hz, C3-H_2), 1.66 (3H, s, Me), 1.58 (3H, s, Me), 1.51 (2H, p, *J* = 7.4 Hz, C2-H_2). **^{13}C NMR (101 MHz, $CDCl_3$) δ** 157.2 (C7), 132.4 (C4), 123.6 (C5), 52.0 (C8), 40.8 (C1), 30.1 (C3), 25.8 (Me), 25.3 (C2), 17.8 (Me). **HRMS** (ESI): calculated for $C_9H_{17}O_2N$ $[M+H]^+$ requires m/z 172.1332, found m/z 172.1335. **IR** (film) ν_{max}: 3112, 1698, 1536, 1525, 1449, 1380, 1262, 1193, 1145, 779 cm^{-1}.

Preparation of Electrophiles

(2d): (2*E*)-3-(4-Methoxyphenyl)prop-2-en-1-ol

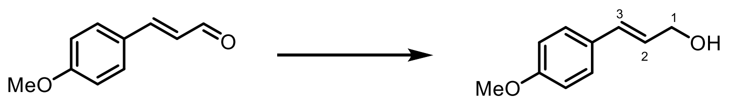

2d

To a solution of 4-methoxycinnamaldehyde (4.80 g, 29.6 mmol) in MeOH (56 mL) under Ar was added $NaBH_4$ (840 mg, 17.8 mmol) portionwise at 0 °C. The reaction mixture was warmed to rt after 30 min at 0 °C. After an additional 1 h at rt the reaction was quenched with aq. NH_4Cl. The aqueous later was extracted with Et_2O and the combined organic layers were washed with brine and dried over $MgSO_4$ before concentrating *in vacuo*. Recrystallisation from Et_2O afforded **2d** as a white solid (4.85 g, 98%).

Data for **2d**: **R_f** 0.50 (80% Et_2O – pentane). **^{1}H NMR (400 MHz, $CDCl_3$) δ** 7.32 (2H, d, *J* = 8.7 Hz, Ar), 6.86 (2H, d, *J* = 8.7 Hz, Ar), 6.56 (1H, d, *J* = 15.9 Hz, C3-H), 6.24 (1H, dt, *J* = 15.8, 6.0 Hz, C2-H), 4.29 (2H, dd, *J* = 5.9, 1.2 Hz, C1-H_2), 3.81 (3H, s, OMe), 1.53 (1H, *br.* s, *O*H). **^{13}C NMR (100 MHz, $CDCl_3$) δ** 159.5 (C Ar), 131.1 (C3), 129.6 (C Ar), 127.8 (2 × C Ar), 126.4 (C2), 114.2 (2 × C Ar), 64.1 (C1), 55.43 (OMe). *The spectroscopic properties were consistent with the data available in the literature.*[22]

(S27): Ethyl (*E*)-3-(4-hydroxyphenyl)acrylate

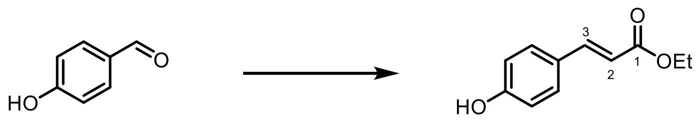

S27

To a solution of 4-hydroxybenzaldehyde (305 mg, 2.50 mmol) in CH_2Cl_2 (2.5 mL) was added ethyl(triphenylphosphoranylidene)acetate (1.34 g, 3.75 mmol) and stirred for 16 h. Consumption of aldehyde was monitored by TLC and upon completetion the solvent was removed *in vacuo*. Flash column chromatography (10% → 20% EtOAc – pentane) afforded **S27** as a white solid (364 mg, 76%).

Data for **S27**: **R_f** 0.50 (30% EtOAc – pentane). **^{1}H NMR (400 MHz, $CDCl_3$) δ** 7.63 (1H, d, *J* = 16.0 Hz, C3-H), 7.50 – 7.35 (2H, m, Ar), 6.94 – 6.78 (2H, m, Ar), 6.30 (1H, d, *J* = 15.9 Hz, C2-H), 4.26 (2H, q, *J* = 7.1 Hz, C**H_2**CH_3), 1.34 (3H, t, *J* = 7.1 Hz, CH_2C**H_3**). **^{13}C NMR (101 MHz, $CDCl_3$) δ** 166.8 (CO), 158.1 (C Ar), 144.9 (C3), 130.1 (2 × C Ar), 127.2 (C Ar), 116.0 (2 × C Ar), 115.5 (C2), 60.8 (**C**H_2CH_3), 14.5 (CH_2CH_3). *The spectroscopic properties were consistent with the data available in the literature.*[23]

(2i): (*E*)-4-(3-Hydroxyprop-1-en-1-yl)phenol

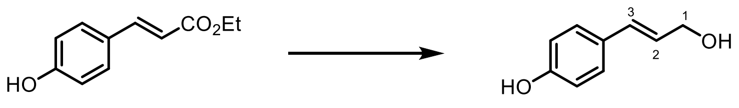

S27 **2i**

To a solution of **S27** (200 mg, 1.04 mmol) in CH_2Cl_2 (6.5 mL) was added DIBALH (1.0 M in CH_2Cl_2, 3.60 mL, 3.64 mmol) dropwise at -78 ˚C before warming to rt. After 1 h the solution was cooled to -78 ˚C and aq. sat. Rochelle salts (3.5 mL) were added before warming to rt. To solution was stirred vigorously for 2 h before diluted with EtOAc. The aqueous layer was extracted with EtOAc and the combined organic layers washed with brine and dried over $MgSO_4$ before concentrating *in vacuo*. Flash column chromatography (10% → 35% EtOAc – pentane) afforded **2i** as a white solid (135 mg, 90%).

Data for **2i**: $\mathbf{R_f}$ 0.27 (30% EtOAc – pentane). **^{1}H NMR (400 MHz, DMSO) δ** 9.44 (1H, s, *O*H), 7.22 (2H, d, *J* = 8.6 Hz, Ar), 6.70 (2H, d, *J* = 8.6 Hz, Ar), 6.42 (1H, dt, *J* = 16.0, 1.7 Hz, C3-H), 6.12 (1H, dt, *J* = 15.9, 5.4 Hz, C2-H), 4.06 (2H, td, *J* = 5.5, 1.6 Hz, C1-H_2). **^{13}C NMR (101 MHz, DMSO) δ** 156.8 (C Ar), 128.7 (C3), 127.9 (C Ar), 127.4 (2 × C Ar), 127.2 (C2), 115.4 (2 × C Ar), 61.73 (C1). *The spectroscopic properties were consistent with the data available in the literature.*[24]

(2m): (±)-(*E*)-4-Phenylbut-3-en-2-ol

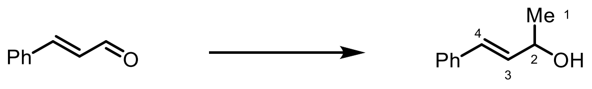

2m

To a solution of cinamaldehyde (1.26 mL, 10.0 mmol) in THF (15 mL) was added MeMgBr (3.0 M in Et2O, 3.7 mL, 11 mmol) dropwise at 0 °C. After 30 min at 0 °C the reaction was warmed to rt and quenched with sat. aq. NH_4Cl (10 mL). The aqueous later was extracted with Et_2O and the combined organic layers were washed with brine, dried over $MgSO_4$ before concentrating *in vacuo*. Flash column chromatography (50% Et_2O – pentane) afforded **2m** as a colourless oil (1.39 g, 94%).

Data for **2m**: $\mathbf{R_f}$ 0.30 (80% Et_2O – pentane). **^{1}H NMR (400 MHz, CDCl3) δ** 7.36 – 7.41 (2H, m, Ar), 7.29 – 7.35 (2H, m, Ar), 7.21 – 7.27 (1H, m, Ar), 6.57 (1H, d, *J* = 15.8 Hz, C4-H), 6.27 (1H, dd, *J* = 15.9, 6.4 Hz, C3-H), 4.44 – 4.55 (1H, m, C2-H), 1.66 (1H, *br.* s, *O*H), 1.38 (3H, d, *J* = 6.4 Hz, C1-H_3). **^{13}C NMR (100 MHz, $CDCl_3$) δ** 136.8 (C Ar), 133.7 (C3), 129.5 (C4), 128.7 (2 × C Ar), 127.8 (C Ar), 126.6 (2 × C Ar), 69.1 (C2), 23.5 (C1). *The spectroscopic properties were consistent with the data available in the literature.*[25]

(2o): (±)-Cyclopent-2-en-1-ol

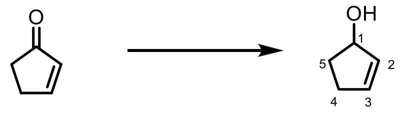

2o

To a solution of cerium trichloride (976 mg, 3.96 mmol) in MeOH (10 mL) was added cyclopent-2-enone (0.50 mL, 6.0 mmol). After 5 min, sodium borohydride (454 mg, 12.0 mmol) was added portionwise before stirring at rt for 15 min. H_2O was added until a clear solution was observed, the aqueous later was extracted with Et2O and the combined organic layers were washed with brine, dried over $MgSO_4$ before concentrating *in vacuo*. Flash column chromatography (20% Et_2O – pentane) afforded **2o** as a colourless oil (368 mg, 73%).

Data for **2o**: **R_f** 0.45 (50% Et_2O – pentane). **^{1}H NMR (400 MHz, $CDCl_3$) δ** 5.93 – 5.99 (1H, m, C3-H), 5.78 – 5.84 (1H, m, C2-H), 4.82-4.88 (1H, m, C1-H), 2.49 (1H, ddt, *J* = 16.5, 10.9, 5.4 Hz, C4-H_A), 2.17 – 2.31 (2H, m, 1 × C4-H_B + 1 × C5-H_A), 1.95 (1H, *br.* s, *O*H), 1.61 – 1.76 (1H, m, C5-H_B). **^{13}C NMR (100 MHz, $CDCl_3$) δ** 135.2 (C3), 133.4 (C2), 77.6 (C1), 33.3 (C5), 31.1 (C4). *The spectroscopic properties were consistent with the data available in the literature.*[25]

(2p): (±)-1-Methylcyclopent-2-en-1-ol

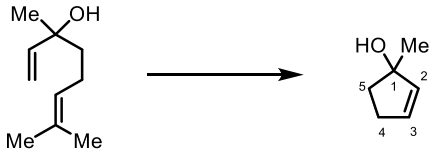

2p

Prepared according to **General Procedure C** using linalool (500 mg, 3.23 mmol) and Grubbs I (133 mg, 0.161 mmol) at rt in $CHCl_3$. Flash column chromatography (20% → 50% Et_2O – pentane) afforded **2p** as a yellow oil (250 mg, 79%).

Data for **2p**: **R_f** 0.40 (80% Et_2O – pentane). **^{1}H NMR (400 MHz, $CDCl_3$) δ** 5.81 (1H, dt, *J* = 5.4, 2.3 Hz, C3-H), 5.69 (1H, dt, *J* = 5.4, 2.3 Hz, C2-H), 2.48 (1H, m, C4-H_A), 2.30 (1H, m, C4-H_B), 1.96 (1H, ddd, *J* = 13.4, 8.1, 4.8 Hz, C5-H_A), 1.89 (1H, ddd, *J* = 13.4, 8.1, 4.8 Hz, C5-H_B), 1.37 (3H, s, Me). **^{13}C NMR (100 MHz, $CDCl_3$) δ** 138.0 (C2), 132.8 (C3), 83.6 (C1), 39.8 (C5), 31.2 (C4), 27.6 (Me). *The spectroscopic properties were consistent with the data available in the literature.*[25]

(2q): (±)-1-Phenylcyclopent-2-en-1-ol

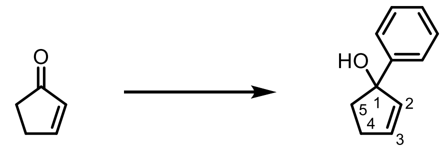

2q

To a solution of bromobenzene (0.63 mL, 6.0 mmol) in THF (30 mL) was added *n*-butyllithium (2.5 M in hexanes, 2.08 mL, 5.20 mmol) dropwise at -78 °C. After stirring at -78 °C for 30 min cyclopent-2-enone (0.34 mL, 4.0 mmol) in THF (5 mL) was added slowly. After 1 h stirring at -78 °C H_2O was added and allowed to warm to rt. The aqueous later was extracted with Et_2O and the combined organic layers were washed with brine, dried over $MgSO_4$ before concentrating *in vacuo*. Flash column chromatography (20% Et_2O – pentane) afforded **2q** as a white solid (0.564 g, 88%).

Data for **2q**: $\mathbf{R_f}$ 0.45 (30% Et_2O – pentane). **^{1}H NMR (400 MHz, $CDCl_3$) δ** 7.43 – 7.47 (2H, m, Ar), 7.32 – 7.38 (2H, m, Ar), 7.23 – 7.28 (1H, m, Ar), 6.12 (1H, dt, J = 5.6, 2.4 Hz, C3-H), 5.88 (1H, dt, J = 5.5, 2.2 Hz, C2-H), 2.60 – 2.70 (1H, m, C4-H_A), 2.43 – 2.52 (1H, m, C4-H_B), 2.24 – 2.29 (2H, m, C5-H_2), 1.98 (1H, *br.* s, *O*H). **^{13}C NMR (100 MHz, $CDCl_3$) δ** 147.0 (C Ar), 136.6 (C2), 134.9 (C3), 128.2 (2 × C Ar), 126.8 (C Ar), 124.9 (2 × C Ar), 87.0 (C1), 42.0 (C5), 31.5 (C4). *The spectroscopic properties were consistent with the data available in the literature.*[25]

Cyclisations

(3a): (±)-(2*S*,3*R*)-3-Benzhydryl-2-(4-methoxyphenyl)-1-tosylpyrrolidine

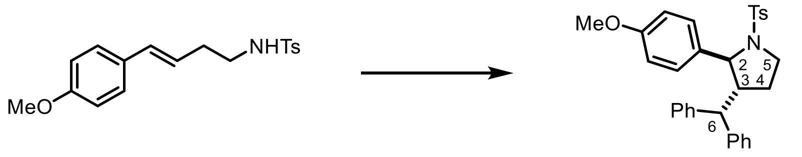

1a **3a**

Prepared according to **General Procedure K** using **1a** (27 mg, 0.082 mmol) and benzhydrol (15 mg, 0.082 mmol). Flash column chromatography (gradient elution 20% → 25% Et_2O – pentane) afforded **3a** as a colourless oil (26 mg, 64%).

Data for **3a**: **R_f** 0.50 (60% Et_2O – pentane). **^{1}H NMR (400 MHz, $CDCl_3$) δ** 7.67 (2H, d, *J* = 8.3 Hz, Ar), 7.35 (2H, d, *J* = 8.3 Hz, Ar), 7.23 – 7.27 (2H, m, Ar), 7.17 – 7.22 (3H, m, Ar), 7.07 – 7.14 (1H, m, Ar), 7.05 (2H, d, *J* = 8.4 Hz, Ar), 6.98 (2H, d, *J* = 8.4 Hz, Ar), 6.95 (2H, d, *J* = 8.7 Hz, Ar), 6.77 (2H, d, *J* = 8.7 Hz, Ar), 4.47 (1H, d, *J* = 2.0 Hz, C2-H), 3.78 (3H, s, OMe), 3.67 – 3.73 (1H, m, C5-H_A), 3.52 (1H, td, *J* = 10.0, 6.7 Hz, C5-H_B), 3.20 (1H, d, *J* = 12.1 Hz, C6-H), 2.93 (1H, ddt, *J* = 12.1, 6.6, 2.0 Hz, C3-H), 2.52 (3H, s, TsMe), 2.03 – 2.12 (1H, m, C4-H_A), 1.52 (1H, ddt, *J* = 13.2, 6.7, 2.3 Hz, C4-H_B). **^{13}C NMR (100 MHz, $CDCl_3$) δ** 158.6 (C Ar), 143.4 (C Ar), 143.0 (C Ar), 142.8 (C Ar), 135.7 (C Ar), 135.6 (C Ar), 129.8 (2 × C Ar), 128.82 (2 × C Ar), 128.75 (2 × C Ar), 128.5 (2 × C Ar), 127.8 (4 × C Ar), 127.1 (2 × C Ar), 126.9 (C Ar), 126.7 (C Ar), 113.8 (2 × C Ar), 66.4 (C2), 55.4 (OMe), 54.0 (C6), 52.9 (C3), 47.5 (C5), 26.9 (C4), 21.7 (TsMe). **NOESY- 2D (500 MHz, $CDCl_3$)**: between C2-H and C6-H. **HRMS** (ESI): calculated for $C_{31}H_{32}NO_3S$ $[M+H]^+$ requires *m/z* 498.2103, found *m/z* 498.2097. **IR** (film) ν_{max}: 3649, 2980, 2888, 1611, 1511, 1159 cm^{-1}.

(3b): (±)-(2*S*,3*R*)-3-Benzhydryl-2-(4-methoxyphenyl)-1-(methylsulfonyl)pyrrolidine

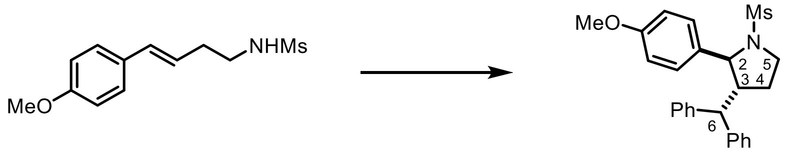

1b **3b**

Prepared according to **General Procedure K** using **1a** (22 mg, 0.086 mmol) and benzhydrol (16 mg, 0.086 mmol). Flash column chromatography (gradient elution 40% → 45% Et_2O – pentane) afforded **3b** as a colourless oil (19.5 mg, 43%).

Data for **3b**: **R_f** 0.30 (80% Et_2O – pentane). **^{1}H NMR (400 MHz, $CDCl_3$) δ** 7.15 – 7.32 (10H, m, Ar), 6.94 (2H, d, *J* = 8.7 Hz, Ar), 6.78 (2H, d, *J* = 8.7 Hz, Ar), 4.61 (1H, d, *J* = 2.4 Hz, C2-H), 3.87 (1H, d,

J = 11.9 Hz, C6-H), 3.74 – 3.81 (1H, m, C5-H_A), 3.78 (3H, s, OMe), 3.61 (1H, ddd, J = 9.6, 8.0, 3.6 Hz, C5-H_B), 3.06 (1H, ddt, J = 11.9, 6.5, 3.0 Hz, C3-H), 2.62 (3H, s, SO_2Me), 1.10 – 1.21 (1H, m, C4-H_A), 1.75 (1H, ddt, J = 13.5, 6.5, 3.5 Hz, C4-H_B). **^{13}C NMR (100 MHz, $CDCl_3$) δ** 158.8 (C Ar),143.1 (C Ar), 143.0 (C Ar), 135.0 (C Ar), 129.0 (2 × C Ar), 128.9 (2 × C Ar), 128.4 (2 × C Ar), 128.0 (2 × C Ar), 127.4 (2 × C Ar), 127.0 (C Ar), 126.8 (C Ar), 114.0 (2 × C Ar), 66.2 (C2), 55.4 (OMe), 54.7 (C6), 53.1 (C3), 47.3 (C5), 39.3 (SO_2Me), 27.9 (C4). **HRMS** (ESI): calculated for $C_{25}H_{27}NO_3SNa$ $[M+Na]^+$ requires *m/z* 444.1609, found *m/z* 444.1602. **IR** (film) ν_{max}: 2980, 1511, 1493, 1452, 1247, 1175 cm^{-1}.

(3c): (±)-(2*S*,3*R*)-3-Benzhydryl-2-(4-methoxyphenyl)-1-[(2-nitrophenyl)sulfonyl]pyrrolidine

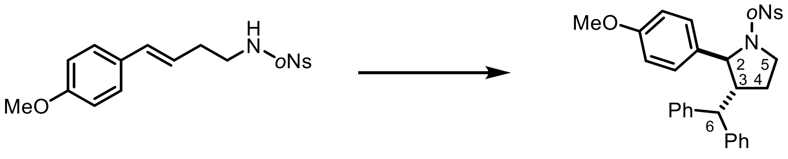

1c **3c**

Prepared according to **General Procedure K** using **1c** (40 mg, 0.11 mmol) and benzhydrol (20 mg, 0.11 mmol). Flash column chromatography (gradient elution 15% → 25% Et_2O – pentane) afforded **3c** as a colourless oil (58 mg, 99%).

Data for **3c**: **R_f** 0.30 (50% Et_2O – pentane). **^{1}H NMR (400 MHz, $CDCl_3$) δ** 7.47 – 7.55 (2H, m, Ar), 7.34 (1H, dd, J = 8.0, 1.3 Hz, Ar), 7.19 – 7.28 (6H, m, Ar), 7.06 – 7.18 (6H, m, Ar), 6.74 (2H, d, J = 8.7 Hz, Ar), 6.52 (2H, d, J = 8.7 Hz, Ar), 4.65 (1H, d, J = 3.6 Hz, C2-H), 3.97 (1H, dt, J = 10.2, 7.4 Hz, C5-H_A), 3.88 (1H, ddd, J = 10.2, 7.7, 5.0 Hz, C5-H_B), 3.79 (1H, d, J = 11.5 Hz, C6-H), 3.70 (3H, s, OMe), 3.05 (1H, m, C3-H), 3.01 – 3.09 (1H, m, C4-H_A), 1.72 (1h, ddt, J = 12.3, 7.2, 5.0 Hz, C4-H_B). **^{13}C NMR (100 MHz, $CDCl_3$) δ** 158.7 (C Ar), 147.7 (C Ar), 143.0 (C Ar), 142.9 (C Ar), 133.8 (C Ar), 133.6 (C Ar), 132.8 (C Ar), 131.2 (C Ar), 131.1 (C Ar), 128.9 (2 × C Ar),128.8 (2 × C Ar), 128.3 (2 × C Ar), 127.9 (2 × C Ar), 127.7 (2 × C Ar), 126.8 (C Ar), 126.8 (C Ar), 123.6 (C Ar), 113.6 (2 × C Ar), 67.0 (C2). 55.4 (OMe), 54.9 (C6), 53.6 (C3), 48.6 (C5), 28.3 (C4). **HRMS**: stable ion was not found in ESI, EI and CI. **IR** (film) ν_{max}: 2980, 1542, 1511, 1493, 1371, 1161 cm^{-1}.

<u>*Gram scale reaction:*</u>

To a solution of **1c** (906 mg, 2.50 mmol) in HFIP (25 mL) was added benzhydrol (461 mg, 2.50 mmol) before addition of $Ti(O^iPr)_4$ (0.22 mL, 30 mol%). Following stirring at rt for 16 h the solvent was removed *in vacuo.* Flash column chromatography (gradient elution 15% → 25% Et_2O – pentane) afforded **3c** as a colourless oil (1.25 g, 95%).

(3d): (±)-(2*S*,3*R*)-3-Benzhydryl-2-(4-methoxyphenyl)-1-[(4-nitrophenyl)sulfonyl]-pyrrolidine

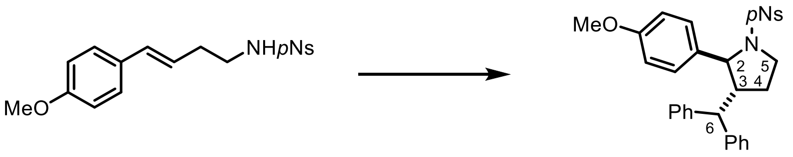

1d **3d**

Prepared according to **General Procedure K** using **1d** (36 mg, 0.10 mmol) and benzhydrol (18 mg, 0.10 mmol). Flash column chromatography (gradient elution 10% → 20% EtOAc – pentane) afforded **3d** as a colourless oil (47 mg, 89%).

Data for **3c**: $\mathbf{R_f}$ 0.30 (30% EtOAc – pentane). **^{1}H NMR (500 MHz, $CDCl_3$) δ** 8.21 (2H, d, *J* = 8.8 Hz, Ar), 7.71 (2H, d, *J* = 8.8 Hz, Ar), 7.23 (4H, m, Ar), 7.20 – 7.17 (1H, m, Ar), 7.17 – 7.11 (3H, m, Ar), 7.09 – 7.01 (2H, m, Ar), 6.76 (2H, d, *J* = 8.6 Hz, Ar), 6.65 (2H, d, *J* = 8.7 Hz, Ar), 4.57 (1H, d, *J* = 2.7 Hz, C2-H), 3.75 (3H, s, OMe), 3.74 – 3.69 (1H, m, C5-H_A), 3.66 (1H, ddd, *J* = 9.6, 8.0, 4.1 Hz, C5-H_B), 3.49 (1H, d, *J* = 11.8 Hz, C6-H), 3.07 – 2.94 (1H, m, C3-H), 2.14 (1H, dtd, *J* = 13.1, 8.3, 6.7 Hz, C4-H_A), 1.68 (ddt, *J* = 13.2, 7.3, 3.9 Hz, C4-H_B). **^{13}C NMR (126 MHz, $CDCl_3$) δ** 158.9 (C Ar), 149.8 (C Ar), 145.1 (C Ar), 142.7 (C Ar), 142.6 (C Ar), 134.2 (C Ar), 128.9 (2 × C Ar), 128.9 (2 × C Ar), 128.4 (2 × C Ar), 128.3 (2 × C Ar), 127.8 (2 × C Ar), 127.5 (2 × C Ar), 127.1 (C Ar), 126.9 (C Ar), 124.1 (2 × C Ar), 113.8 (2 × C Ar), 67.0 (C2), 55.4 (OMe), 54.7 (C6), 52.9 (C3), 47.8 (C5), 27.8 (C4). **HRMS** (ESI): calculated for $C_{30}H_{28}N_2O_5SNa$ $[M+Na]^+$ requires *m/z* 551.1611, found *m/z* 551.1611. **IR** (film) ν_{max}: 2913, 2360, 1530, 1513, 1350, 1249, 1164, 1104 cm^{-1}.

(3e): (±)-Benzyl (2*S*,3*R*)-3-benzhydryl-2-(4-methoxyphenyl)pyrrolidine-1-carboxylate

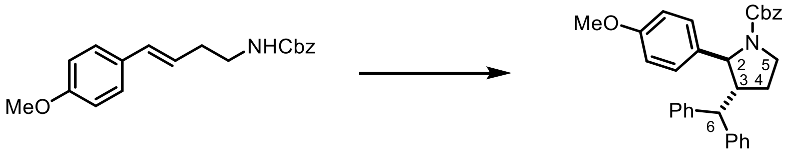

1e **3e**

Prepared according to **General Procedure K** using **1e** (31 mg, 0.10 mmol) and benzhydrol (18 mg, 0.10 mmol). Flash column chromatography (gradient elution 10% → 15% EtOAc – pentane) afforded **3e** as a colourless oil (37 mg, 77%).

Data for **3e,** 1.75:1 mixture of rotamers *A:B*: $\mathbf{R_f}$ 0.45 (30% EtOAc – pentane). **^{1}H NMR (600 MHz, $CDCl_3$) δ** 7.41 – 7.23 (10H, m, Ar), 7.22 – 7.12 (4H, m, Ar), 6.93 – 6.82 (3H, m, Ar), 6.80 – 6.74 (2H, m, Ar), 5.13 (1H, q, *J* = 12.4 Hz, CH_2Ph, *B*), 5.05 – 4.95 (1H, m, CH_2Ph, *A*), 4.73 (1H, *br.* s, C2-H, *B*), 4.68 (1H, *br.* s, C2-H, *A*), 3.78 (3H, s, OMe, *A*+*B*), 3.85 – 3.61 (3H, m, C5-H_2 + C6-H, *A*+*B*), 3.02 –

2.96 (1H, m, C3-H, *A*+*B*), 2.04 (1H, dq, *J* = 15.8, 9.3 Hz, C4-H_A, *A*+*B*), 1.67 – 1.59 (1H, m, C4-H_B). ^{13}C NMR (151 MHz, $CDCl_3$) δ 158.5, 155.4, 154.8, 143.7, 143.5, 143.5, 143.3, 137.1, 137.1, 136.1, 135.7, 129.0, 128.8, 128.8, 128.6, 128.6, 128.4, 128.3, 128.3, 128.0, 128.0, 127.6, 127.3, 126.8, 126.7, 126.6, 126.6, 113.9, 113.8, 66.9 (CH_2Ph, *B*), 66.5 (CH_2Ph, *A*), 64.8 (C2, *B*), 64.3 (C2, *A)*, 55.4 (OMe, *A*), 55.4 (OMe, *B*), 54.7 (C6, *B*), 54.5 (C6, *A*), 52.5 (C3, *A*), 51.7 (C3, *B*), 45.8 (C5, *A*), 45.6 (C5, *B*), 26.9 (C4, *B*), 25.7 (C4, *A*). **HRMS** (ESI): calculated for $C_{32}H_{32}NO_3$ $[M+H]^+$ requires *m/z* 478.2377, found *m/z* 478.2376. **IR** (film) ν_{max}: 2953, 2361, 1698, 1512, 1451, 1411, 1351, 1247 cm^{-1}.

(3g): (±)-(2*S*,3*R*)-2-(4-Methoxyphenyl)-1-[(2-nitrophenyl)sulfonyl)-3-[(1'*R*)-3-phenylcyclopent-2-en-1-yl]pyrrolidine

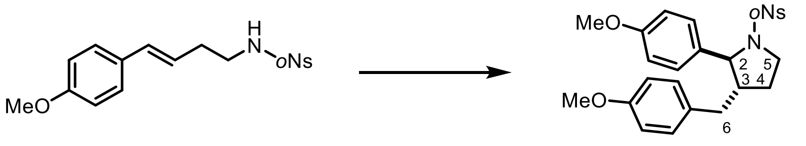

1c **3g**

Prepared according to **General Procedure K** using **1c** (40 mg, 0.11 mmol) and 4-methoxybenzyl alcohol (15 mg, 0.11 mmol). Flash column chromatography (gradient elution 15% → 25% EtOAc – pentane) afforded **3g** as a colourless oil (53 mg, 99%).

Data for **3g: R_f** 0.45 (40% EtOAc – pentane). **^{1}H NMR (500 MHz, $CDCl_3$) δ** 7.45 – 7.53 (2H, m, Ar), 7.24 – 7.27 (1H, m, Ar), 7.18 – 7.23 (1H, m, Ar), 7.00 (2H, d, *J* = 8.6 Hz, Ar), 6.95 (2H, d, *J* = 8.6 Hz, Ar), 6.78 (2H, d, *J* = 8.6 Hz, Ar), 6.60 (2H, d, *J* = 8.6 Hz, Ar), 4.48 (1H, d, *J* = 7.4 Hz, C2-H), 4.05 (1H, ddd, *J* = 11.9, 8.9, 3.5 Hz, C5-H_A), 3.77 (3H, s, OMe), 3.71 – 3.76 (1H, m, C5-H_B), 3.73 (3H, s, OMe), 2.69 (1H, dd, *J* = 13.4, 5.2 Hz, C6-H_A), 2.45 (1H, dd, *J* = 13.5, 9.2 Hz, C6-H_B), 2.32-2.41 (1H, m, C3-H), 2.00 (1H, dtd, *J* = 12.2, 6.1, 3.6 Hz, C4-H_A), 1.71 (1H, dq, *J* = 12.3, 8.9 Hz, C4-H_B). **^{13}C NMR (125 MHz, $CDCl_3$) δ** 159.2 (C Ar), 158.2 (C Ar), 147.5 (C Ar), 134.1 (C Ar), 132.6 (CH Ar), 132.1 (C Ar), 131.5 (C Ar), 131.0 (C Ar), 130.9 (C Ar), 129.9 (2 × C Ar), 128.7 (2 × C Ar), 123.6 (C Ar), 114.0 (2 × C Ar), 113.7 (2 × C Ar), 68.5 (C2), 55.40 (OMe), 55.37 (OMe), 52.3 (C3), 49.4 (C5), 37.0 (C6), 30.3 (C4). **NOESY- 2D (500 MHz, $CDCl_3$)**: between C2-H and C6-H_A, between C2-H and C6-H_B. **HRMS** (ESI): calculated for $C_{25}H_{27}N_2O_5S$ $[M+H]^+$ requires *m/z* 483.1590, found *m/z* 483.1585. **IR** (film) ν_{max}: 2980, 2890, 1514, 1510, 1244 cm^{-1}.

(3h): (±)-(2*S*,3*S*)-3-Cinnamyl-2-(4-methoxyphenyl)-1-[(2-nitrophenyl)sulfonyl]pyrrolidine

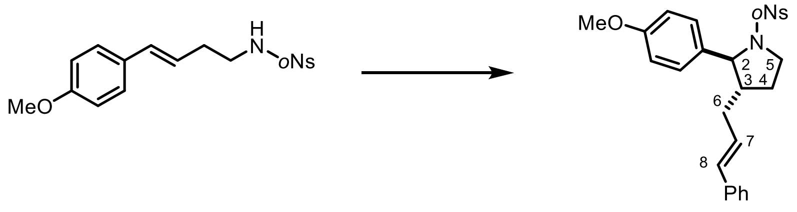

1c **3h**

Prepared according to **General Procedure K** using **1c** (30 mg, 0.083 mmol) and cinnamyl alcohol (11 mg, 0.083 mmol) at 70 ˚C. Flash column chromatography (gradient elution 10% → 25% EtOAc – pentane) afforded **3h** as a colourless oil (23 mg, 58%).

Data for **3h**: $\mathbf{R_f}$ 0.30 (30% EtOAc – pentane). ^{1}**H NMR (400 MHz, CDCl$_3$) δ** 7.51 (1H, dd, *J* = 7.9, 1.4 Hz, Ar), 7.43 – 7.48 (1H, m, Ar), 7.23 – 7.29 (5H, m, Ar), 7.16 – 7.22 (2H, m, Ar), 7.05 (2H, d, *J* = 8.7 Hz, Ar), 6.60 (2H, d, *J* = 8.7 Hz, Ar), 6.35 (1H, d, *J* = 15.8 Hz, C8-H), 6.03 (1H, dt, *J* = 15.8, 7.1 Hz, C7-H), 4.49 (1H, d, *J* = 7.5 Hz, C2-H), 4.08 (1H, ddd, *J* = 10.9, 7.9, 3.1 Hz, C5-H_A), 3.75 – 3.82 (1H, m, C5-H_B), 3.72 (3H, s, OMe), 2.32 – 2.39 (1H, m, C6-H_A), 2.26 – 2.32 (1H, m, C3-H), 2.14 – 2.23 (2H, m, C4-H_A + C6-H_B), 1.75 (1H, dtd, *J* = 12.3, 9.5, 7.9 Hz, C4-H_B). ^{13}**C NMR (100 MHz, CDCl$_3$) δ** 159.2 (C Ar), 147.5 (C Ar), 137.3 (C Ar), 134.1 (C Ar), 132.6 (C Ar), 132.3 (C Ar + C8), 132.1 (C Ar), 131.1 (C Ar), 130.9 (C Ar), 128.9 (2 × C Ar), 128.6 (2 × C Ar), 127.4 (C7), 126.2 (2 × C Ar), 123.6 (C Ar), 113.8 (2 × C Ar), 68.7 (C2), 55.4 (OMe), 50.4 (C3), 49.6 (C5), 35.3 (C4), 30.6 (C6). **NOESY- 2D (500 MHz, CDCl$_3$)**: between C2-H and C6-H_B, between C2-H and C7-H. **HRMS** (ESI): calculated for $C_{26}H_{27}N_2O_5S$ [M+H]$^+$ requires *m/z* 479.1641, found *m/z* 479.1636. **IR** (film) ν_{max}: 2980, 1541, 1511, 1371, 1289, 1159 cm^{-1}.

(3i): (±)-4-((*E*)-3-((2*S*,3*S*)-2-(4-Methoxyphenyl)-1-((2-nitrophenyl)sulfonyl)pyrrolidin-3-yl)prop-1-en-1-yl)phenol

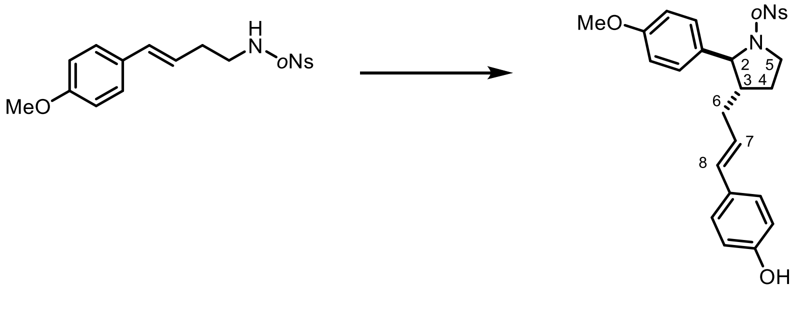

1c **3i**

Prepared according to **General Procedure K** using **1c** (18 mg, 0.050 mmol) and **2i** (7.5 mg, 0.050 mmol) at 70 ˚C. Flash column chromatography (gradient elution 10% → 30% EtOAc – pentane) afforded **3i** as a colourless oil (12 mg, 49%).

Data for **3i**: **R_f** 0.42 (40% EtOAc – pentane). **^{1}H NMR (600 MHz, $CDCl_3$) δ** 7.51 (1H, dd, *J* = 7.9, 1.3 Hz, Ar), 7.46 (1H, ddd, *J* = 7.9, 7.3, 1.4 Hz, Ar), 7.25 (1H, d, *J* = 8.9 Hz, Ar), 7.19 (1H, ddd, *J* = 8.0, 7.4, 1.4 Hz, Ar), 7.14 (2H, d, *J* = 8.6 Hz, Ar), 7.05 (2H, d, *J* = 8.7 Hz, Ar), 6.74 (2H, d, *J* = 8.6 Hz, Ar), 6.60 (2H, d, *J* = 8.7 Hz, Ar), 6.27 (1H, dd, *J* = 15.8, 1.6 Hz, C8-H), 5.87 (1H, dt, *J* = 15.7, 7.1 Hz, C7-H), 4.78 (1H, *br.* s, *O*H), 4.48 (1H, d, *J* = 7.5 Hz, C2-H), 4.07 (1H, ddd, *J* = 10.8, 8.0, 3.2 Hz, C5-H_A), 3.78 (1H, ddd, *J* = 10.4, 9.5, 6.3 Hz, C5-H_B), 3.72 (3H, s, OMe), 2.36 – 2.24 (2H, m, C3-H + C6-H_A), 2.20 – 2.12 (2H, m, C5-H_A + C6-H_B), 1.74 (1H, dtd, *J* = 12.4, 9.5, 8.0 Hz, C5-H_B). **^{13}C NMR (151 MHz, $CDCl_3$) δ** 159.2 (C Ar), 155.0 (C Ar), 147.5 (C Ar), 134.2 (C Ar), 132.6 (C Ar), 132.2 (C Ar), 131.6 (C8), 131.1 (C Ar), 131.0 (C Ar), 130.4 (C Ar), 128.9 (2 × C Ar), 127.5 (2 × C Ar), 125.2 (C7), 123.6 (C Ar), 115.5 (2 × C Ar), 113.8 (2 × C Ar), 68.7 (C2), 55.4 (OMe), 50.5 (C3), 49.6 (C5), 35.3 (C6), 30.6 (C4). **NOESY- 2D (600 MHz, $CDCl_3$)**: between C2-H and C6-H_B, between C2-H and C7-H. **HRMS** (ESI): calculated for $C_{26}H_{27}N_2O_6S$ $[M+H]^+$ requires *m/z* 495.1584, found *m/z* 495.1585. **IR** (film) ν_{max}: 2923, 2852, 1611, 1542, 1513, 1371, 1247, 1160 cm^{-1}.

(3j): (±)-(2*S*,3*R*)-2-(4-Methoxyphenyl)-1-[(2-nitrophenyl)sulfonyl]-3-[(1'*S*,2'*E*)-pent-3-en-2-yl]-pyrrolidine

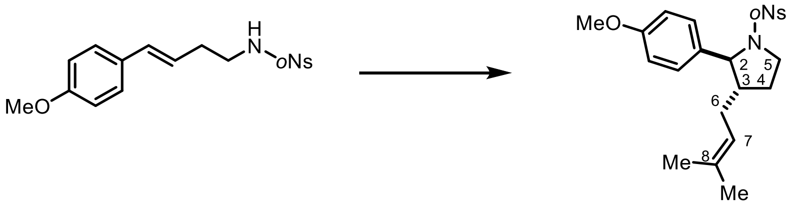

1c **3j**

Prepared according to **General Procedure K** using **1c** (40 mg, 0.11 mmol) and 3-methyl-2-buten-1-ol (19 mg, 0.22 mmol, 2.0 equiv.) at 70 ˚C. Flash column chromatography (gradient elution 10% → 15% EtOAc – pentane) afforded **3j** as a colourless oil (15 mg, 32%).

Data for **3j: R_f** 0.50 (35% EtOAc – pentane). **^{1}H NMR (500 MHz, $CDCl_3$) δ** 7.50 (1H, dd, *J* = 8.0, 1.5 Hz, Ar), 7.43 – 7.48 (1H, m, Ar), 7.24 (1H, dd, *J* = 8.0, 1.5 Hz, Ar), 7.16 – 7.20 (1H, m, Ar), 7.03 (2H, d, *J* = 8.7 Hz, Ar), 6.59 (2H, d, *J* = 8.7 Hz, Ar), 5.00 (1H, t, *J* = 7.2 Hz, C7-H), 4.42 (1H, d, *J* = 7.6 Hz, C2-H), 4.04 (1H, ddd, *J* = 10.6, 7.9, 3.1 Hz, C5-H_A), 3.72 – 3.80 (1H, m, C5-H_B), 3.72 (3H, s, OMe), 2.13 – 2.19 (1H, m, C3-H), 2.05 – 2.13 (2H, m, C4-H_A + C6-H_A), 1.95 (1H, dt, *J* = 15.0, 7.9 Hz, C6-H_B), 1.65 – 1.72 (1H, m, C4-H_B), 1.64 (3H, s, Me), 1.51 (3H, s, Me). **^{13}C NMR (125 MHz, $CDCl_3$) δ** 159.1 (C Ar), 147.5 (C Ar), 134.2 (C Ar), 133.8 (C Ar), 132.5 (C Ar), 132.4 (C8), 131.0 (C Ar), 130.9 (C Ar), 128.8 (2 × C Ar), 123.6 (C Ar), 131.3 (C7), 113.6 (2 × C Ar), 68.8 (C2), 55.4 (OMe), 50.8 (C3), 49.6 (C5), 30.4 (C4), 29.9 (C6), 25.9 (Me), 18.0 (Me). **NOESY- 2D (500 MHz, $CDCl_3$)**: between C2-H and C6-H_B, between C2-H and C7-H. **HRMS** (ESI): calculated for $C_{22}H_{27}N_2O_5S$ $[M+H]^+$ requires *m/z* 431.1641, found *m/z* 431.1634. **IR** (film) ν_{max}: 2980, 2888, 2360, 1541, 1462, 1159, 1127 cm^{-1}.

(3k): (±)-(2*S*,3*R*)-2-(4-Methoxyphenyl)-1-[(2-nitrophenyl)sulfonyl]-3-[(1'*S*,2'*E*)-4-phenylbut-3-en-2-yl]pyrrolidine and (±)-(2*S*,3*R*)-2-(4-Methoxyphenyl)-1-[(2-nitrophenyl)sulfonyl]-3-[(1'*R*,2'*E*)-4-phenylbut-3-en-2-yl]pyrrolidine

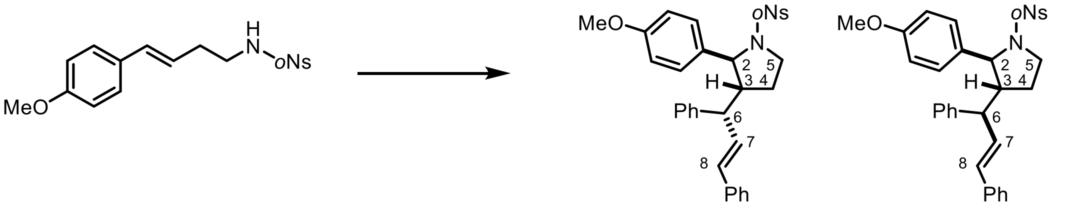

1c **A** **3k** **B**

d.r. (C-6) 7:1

Prepared according to **General Procedure K** using **1c** (40 mg, 0.11 mmol) and *trans*-1,3-diphenyl-2-propen-1-ol (23 mg, 0.11 mmol) at 0 ˚C for 48 h. Flash column chromatography (gradient elution 25% → 30% EtOAc – pentane) afforded **3j** as an inseparable 7:1 mixture of diastereomers as a colourless oil (61 mg, 99%).

Data for **3k-A** (from the mixture): **^{1}H NMR (500 MHz, CDCl$_3$) δ** 7.43 – 7.52 (2H, m, Ar), 7.13 – 7.33 (12H, m, Ar), 7.02 (2H, d, *J* = 8.7 Hz, Ar), 6.55 (2H, d, *J* = 8.7 Hz, Ar), 6.37 (1H, d, *J* = 15.8 Hz, C8-H), 6.04 (1H, dd, *J* = 15.8, 9.3 Hz, C7-H), 4.84 (1H, d, *J* = 5.7 Hz, C2-H), 3.97 (1H, ddd, *J* = 10.3, 7.5, 5.2 Hz, C5-H$_A$), 3.81 (1H, dt, *J* = 10.3, 7.5 Hz, C5-H$_B$), 3.70 (3H, s, OMe), 3.39 (1H, t, *J* = 9.8 Hz, C6-H), 2.64 – 2.71 (1H, m, C3-H), 1.89 – 1.97 (1H, m, C4-H$_A$), 1.67 (1H, dq, *J* = 12.7, 7.5 Hz, C4-H$_B$). **^{13}C NMR (125 MHz, CDCl$_3$) δ** 158.9 (C Ar), 147.5 (C Ar), 142.4 (C Ar), 137.0 (C Ar), 133.9 (C Ar), 133.2 (C Ar), 132.6 (C Ar), 132.2 (C7), 131.06 (C8), 130.97 (2 × C Ar), 129.1 (2 × C Ar), 129.0 (2 × C Ar), 128.5 (2 × C Ar), 127.7 (2 × C Ar), 127.5 (C Ar), 126.94 (C Ar), 126.42 (2 × C Ar), 123.5 (C Ar), 113.7 (2 × C Ar), 67.2 (C2), 55.4 (OMe), 55.0 (C3), 53.5 (C6), 49.2 (C5), 29.1 (C4). **NOESY-2D (500 MHz, CDCl$_3$)**: between C2-H and C6-H, between C2-H and C7-H, between C2-H and C8-H.

Partial data for **3k-B** (from the mixture): **^{1}H NMR (500 MHz, CDCl$_3$) δ** 7.43 – 7.52 (2H, m, Ar), 7.13 – 7.33 (10H, m, Ar), 7.07 (2H, d, *J* = 6.9 Hz, Ar), 6.76 (2H, d, *J* = 8.7 Hz, Ar), 6.53 (2H, d, *J* = 8.7 Hz, Ar), 6.45 (1H, d, *J* = 15.7 Hz, C8-H), 6.29 (1H, dd, *J* = 15.7, 9.3 Hz, C7-H), 4.59 (1H, d, *J* = 5.3 Hz, C2-H), 4.04 (1H, ddd, *J* = 10.2, 7.4, 6.0 Hz, C5-H$_A$), 3.83 – 3.89 (1H, m, C5-H$_B$), 3.70 (3H, s, OMe), 3.38 (1H, d, *J* = 6.0 Hz, C6-H), 2.60 – 2.65 (1H, m, C3-H), 2.12 – 2.20 (1H, m, C4-H$_A$), 1.99 – 2.07 (1H, m, C4-H$_B$). **^{13}C NMR (125 MHz, CDCl$_3$) δ** 158.8 (C Ar), 147.6 (C Ar), 142.4 (C Ar), 137.0 (C Ar), 133.7 (C Ar), 133.1 (C Ar), 132.7 (C Ar), 132.0 (C8), 131.09 (C Ar), 130.3 (C7), 128.8 (2 × C Ar), 128.7 (2 × C Ar), 128.0 (2 × C Ar), 128.0 (2 × C Ar), 127.6 (C Ar), 126.9 (C Ar), 126.4 (C Ar), 126.4 (2 × C Ar), 123.5 (C Ar), 113.6 (2 × C Ar), 66.5 (C2), 55.4 (OMe), 54.8 (C3), 51.5 (C6), 48.9 (C5), 27.7 (C4).

Data for **3k:** $\mathbf{R_f}$ 0.50 (35% EtOAc – pentane). **HRMS** (ESI): calculated for $C_{32}H_{30}N_2O_5SNa$ $[M+Na]^+$ requires *m/z* 577.1773, found *m/z* 577.1764. **IR** (film) ν_{max}: 2980, 2888, 1541, 1511, 1372, 1159 cm^{-1}.

(3l): (±)-(2*S*,3*R*)-2-(4-Methoxyphenyl)-1-[(2-nitrophenyl)sulfonyl]-3-[(1'*S*,2'*E*)-pent-3-en-2-yl]-pyrrolidine and (±)-(2*S*,3*R*)-2-(4-Methoxyphenyl)-1-[(2-nitrophenyl)sulfonyl]-3-[(1'*R*,2'*E*)-pent-3-en-2-yl]pyrrolidine

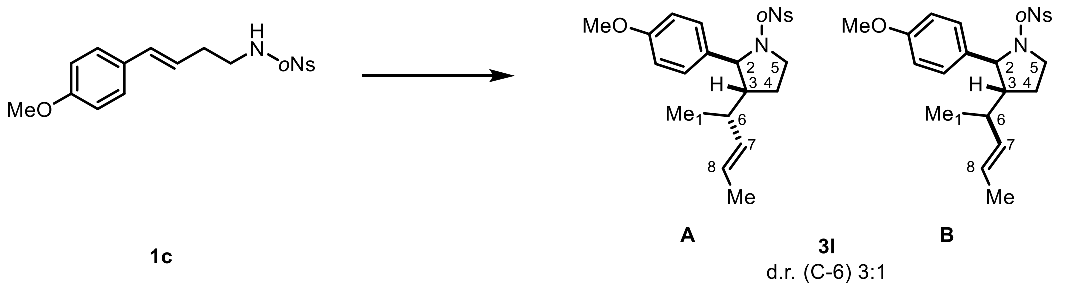

Prepared according to **General Procedure K** using **1c** (40 mg, 0.11 mmol) and 3-penten-2-ol (19 mg, 0.22 mmol, 2.0 equiv.) at 0 ˚C for 48 h. Flash column chromatography (gradient elution 10% → 15% EtOAc – pentane) afforded **3l** as an inseparable 3:1 mixture of diastereomers as a colourless oil (47 mg, 99%).

Data for **3l-A** (from the mixture): **^{1}H NMR (500 MHz, $CDCl_3$) δ** 7.42 – 7.51 (2H, m, Ar), 7.16 – 7.24 (2H, m, Ar), 6.99 (2H, d, *J* = 8.7 Hz, Ar), 6.57 (2H, d, *J* = 8.7 Hz, Ar), 5.32 (1H, dq, *J* = 15.1, 6.3 Hz, C8-H), 4.98 – 5.05 (1H, m, C7-H), 4.61 (1H, d, *J* = 5.8 Hz, C2-H), 3.98 (1H, ddd, *J* = 10.6, 7.6, 3.3 Hz, C5-H_A), 3.76 (1H, ddd, *J* = 10.3, 9.2, 6.1 Hz, C5-H_B), 3.71 (3H, s, OMe), 2.04 – 2.14 (3H, m, C4-H_A + C6-H), 1.71 – 1.81(1H, m, C4-H_B), 1.53 (3H, dd, *J* = 6.4, 1.7 Hz, Me_2), 0.93 (3H, d, *J* = 6.3 Hz, Me_1). **^{13}C NMR (125 MHz, $CDCl_3$) δ** 158.8 (C Ar), 147.4 (C Ar), 135.5 (C7), 134.2 (C Ar), 133.5 (C Ar), 132.4 (C Ar), 130.9 (C Ar), 130.9 (C Ar), 129.2 (2 × C Ar), 124.9 (C8), 123.5 (C Ar), 113.4 (2 × C Ar), 67.0 (C2), 55.5 (C3), 55.4 (OMe), 49.6 (C5), 39.7 (C6), 28.6 (C4), 18.6 (Me_1), 17.8 (Me_2). **NOESY--2D (500 MHz, $CDCl_3$)**: between C2-H and Me_1, between C2-H and C7-H, between C2-H and C8-H.

Partial data for **3l-B** (from the mixture): **^{1}H NMR (500 MHz, $CDCl_3$) δ** 7.42 – 7.51 (2H, m, Ar), 7.16 – 7.24 (2H, m, Ar), 7.04 (2H, d, *J* = 8.7 Hz, Ar), 6.59 (2H, d, *J* = 8.7 Hz, Ar), 5.43 (1H, dq, *J* = 15.2, 6.3 Hz, C8-H), 5.23 – 5.28 (1H, m, C7-H), 4.54 (1H, d, *J* = 7.8 Hz, C2-H), 3.95 – 4.02 (1H, m, C5-H_A), 3.73 – 3.79 (1H, m, C5-H_B), 3.72 (3H, s, OMe), 2.04 – 2.14 (2H, m, C3-H + C6-H), 1.97 (1H, dtd, *J* = 12.1, 6.0, 2.9 Hz, C4-H_A), 1.71 – 1.81(1H, m, C4-H_B), 1.67 (3H, dd, *J* = 6.3, 1.6 Hz, Me_2), 0.92 (3H, d, *J* = 6.4 Hz, Me_1). **^{13}C NMR (125 MHz, $CDCl_3$) δ** 158.8 (C Ar), 134.17 (C Ar), 134.15 (C Ar), 132.7 (C7), 132.5 (C Ar), 132.5 (C Ar), 130.9 (C Ar), 130.9 (C Ar), 129.0 (2 × C Ar), 126.7 (C8), 123.5 (C Ar), 113.7 (2 × C Ar), 66.8 (C2), 55.6 (OMe), 55.4 (C3), 49.5 (C5), 37.0 (C6), 26.8 (C4), 20.2 (Me_1), 18.2 (Me_2). **NOESY- 2D (500 MHz, $CDCl_3$)**: between C2-H and Me_1, between C2-H and C7-H, between C2-H and C8-H.

Data for **3l**: **R_f** 0.40 (30% EtOAc – pentane). **HRMS** (ESI): calculated for $C_{22}H_{27}N_2O_5S$ $[M+H]^+$ requires *m/z* 431.1641, found *m/z* 431.1637. **IR** (film) ν_{max}: 2980, 2888, 1541, 1511, 1372, 1159 cm^{-1}.

(3m): (±)-(2*S*,3*R*)-2-(4-Methoxyphenyl)-1-[(2-nitrophenyl)sulfonyl]-3-[(1'*S*,2'*E*)-4-phenylbut-3-en-2-yl]pyrrolidine, (±)-(2*S*,3*R*)-2-(4-Methoxyphenyl)-1-[(2-nitrophenyl)sulfonyl]-3-[(1'*R*,2'*E*)-4-phenylbut-3-en-2-yl]pyrrolidine, (±)-(2*S*,3*R*)-2-(4-Methoxyphenyl)-1-[(2-nitrophenyl)sulfonyl]-3-[(1'*S*,2'*E*)-1-phenylbut-2-en-1-yl]pyrrolidine, (±)-(2*S*,3*R*)-2-(4-methoxyphenyl)-1-[(2-nitro-phenyl)sulfonyl]-3-[(1'*R*,2'*E*)-1-phenylbut-2-en-1-yl]pyrrolidine

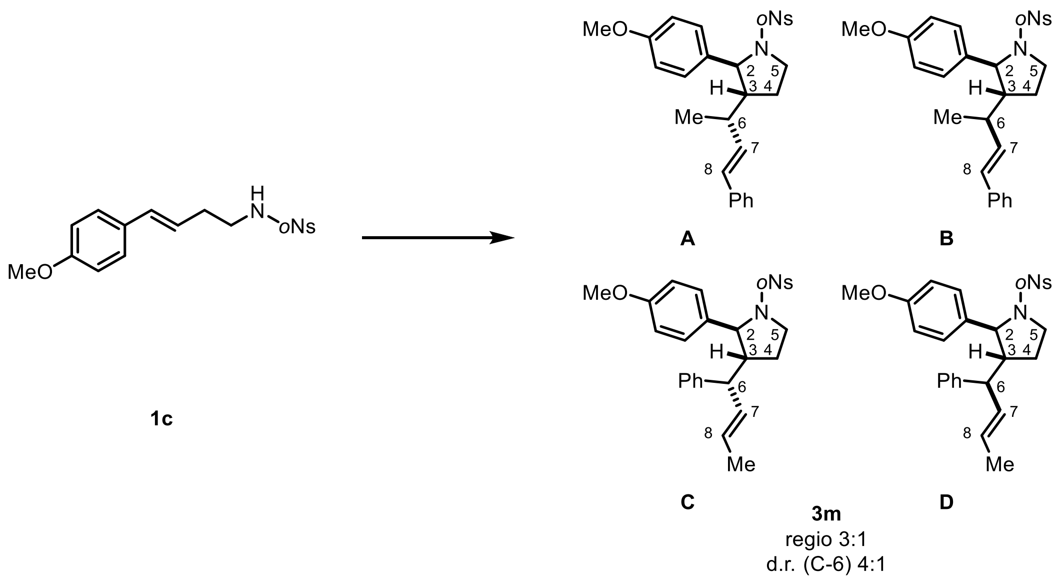

Prepared according to **General Procedure K** using **1c** (40 mg, 0.11 mmol) and **2m** (16.5 mg, 0.22 mmol) at 0 ˚C for 48 h. Flash column chromatography (gradient elution 15% → 25% EtOAc – pentane) afforded **3m** as an inseparable 9:3:3:1 mixture of diastereomers as a colourless oil (53 mg, 99%).

Data for **3m-A** (from the mixture): **^{1}H NMR (500 MHz, $CDCl_3$) δ** 7.00 – 7.52 (9H, m, Ar), 6.97 (2H, d, *J* = 8.7 Hz, Ar), 6.49 (2H, d, *J* = 8.7 Hz, Ar), 6.30 (1H, d, *J* = 15.8 Hz, C8-H), 5.72 (1H, dd, *J* = 15.8, 8.8 Hz, C7-H), 4.66 (1H, d, *J* = 7.3 Hz, C2-H), 4.00 – 4.10 (1H, m, C5-H_A), 3.75 – 3.84 (1H, m, C5-H_B), 3.68 (3H, s, OMe), 2.29 – 2.41 (1H, m, C6-H), 2.20 – 2.29 (1H, m, C3-H), 2.10 – 2.20 (1H, m, C4-H_A), 1.75 – 1.85 (1H, m, C4-H_B), 1.08 (3H, d, *J* = 6.7 Hz, Me). **^{13}C NMR (125 MHz, $CDCl_3$) δ** 158.8 (C Ar), 137.2 (C Ar), 134.6 (C7), 134.2 (C Ar), 132.7 (C Ar), 132.4 (C Ar), 131.0 (C Ar), 130.9 (C Ar), 129.5 (C8), 129.5 (2 × C Ar), 128.8 (C Ar), 128.8 (C Ar), 128.5 (2 × C Ar), 126.2 (2 × C Ar), 123.5 (C Ar), 113.6 (2 × C Ar), 67.3 (C2), 55.8 (C3), 55.4 (OMe), 49.7 (C5), 40.8 (C6), 29.2 (C4), 19.9 (Me). **NOESY- 2D (500 MHz, $CDCl_3$)**: between C2-H and C6-H, between C2-H and C7-H, between C2-H and Me.

Partial data for **3m-B** (from the mixture): **^{1}H NMR (500 MHz, $CDCl_3$) δ** 7.00 – 7.52 (13H, m, Ar), 6.36 (1H, d, *J* = 15.8 Hz, C8-H), 6.02 (1H, dd, *J* = 15.8, 8.8 Hz, C7-H), 4.60 (1H, d, *J* = 8.0 Hz, C2-H),

3.99 – 4.05 (1H, m, C5-H_A), 3.75 – 3.84 (1H, m, C5-H_B), 3.74 (3H, s, OMe), 2.29 – 2.41 (1H, m, C6-H), 2.20 – 2.29 (1H, m, C3-H), 2.00 – 2.09 (1H, m, C4-H_A), 1.75 – 1.85 (1H, m, C4-H_B), 1.06 (3H, d, J = 6.7 Hz, Me). **^{13}C NMR (125 MHz, $CDCl_3$) δ** 159.2 (C Ar), 147.6 (C Ar), 143.2 (C Ar), 133.9 (C Ar), 133.77 (C Ar), 131.7 (C7), 131.1 (C8), 128.65 (2 × C Ar), 128.5 (C Ar), 127.5 (C Ar), 127.2 (2 × C Ar), 126.7 (C Ar), 126.3 (2 × C Ar), 126.2 (C Ar), 123.5 (C Ar), 113.8 (2 × C Ar), 66.8 (C2), 55.7 (C3), 55.39 (OMe), 49.5 (C5), 37.8 (C6), 27.2 (C4), 19.9 (Me). **NOESY- 2D (500 MHz, $CDCl_3$)**: between C2-H and C6-H, between C2-H and C7-H, between C2-H and Me.

Characteristic data for **3m-C** (from the mixture): **^{1}H NMR (500 MHz, $CDCl_3$) δ** 7.00 – 7.52 (11H, m, Ar), 6.65 (2H, d, J = 8.7 Hz, Ar), 5.30 – 5.44 (2H, m, C7-H + C8-H), 4.80 (1H, d, J = 5.1 Hz, C2-H), 3.74 – 3.97 (2H, m, C5-H_2), 3.74 (3H, s, OMe), 3.08 – 3.16 (1H, m, C6-H), 2.43 – 2.53 (1H, m, C3-H), 1.86 – 1.92 (1H, m, C4-H_A), 1.60 – 1.53 (4H, m, C4-H_B + Me). **^{13}C NMR (125 MHz, $CDCl_3$) δ** 131.4 (C7), 126.8 (C8), 66.9 (C2), 55.4 (OMe), 54.7 (C3), 52.8 (C6), 49.0 (C5), 28.5 (C4), 17.9 (Me). **NOESY- 2D (500 MHz, $CDCl_3$)**: between C2-H and C6-H, between C2-H and C7-H.

Data for **3m: R_f** 0.50 (40% EtOAc – pentane). **HRMS** (ESI): calculated for $C_{27}H_{29}N_2O_5S$ $[M+H]^+$ requires *m/z* 493.1797, found *m/z* 493.1792. **IR** (film) ν_{max}: 2980, 2888, 1541, 1511, 1372, 1247 cm^{-1}.

(3n): (±)-(2*S*,3*R*)-3-[(1'*R*)-Cyclohex-2-en-1-yl]-2-(4-methoxyphenyl)-1-[(2-nitrophenyl)sulfonyl]-pyrrolidine and (±)-(2*S*,3*R*)-3-[(1'*S*)-Cyclohex-2-en-1-yl]-2-(4-methoxyphenyl)-1-[(2-nitro-phenyl)sulfonyl]pyrrolidine

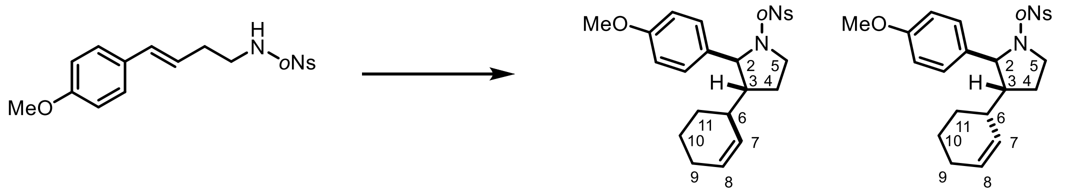

1c

A

3n

B

d.r. (C-6) 2.5:1

Prepared according to **General Procedure K** using **1c** (30 mg, 0.083 mmol) and 2-cyclohexen-1-ol (8.0 mg, 0.083 mmol) at 70 ˚C. Flash column chromatography (gradient elution 10% → 15% EtOAc – pentane) afforded **3n** as an inseparable 2.5:1 mixture of diastereomers as a colourless oil (21.5 mg, 58%).

Data for **3n-A** (from the mixture): **^{1}H NMR (500 MHz, $CDCl_3$) δ** 7.42 – 7.51 (2H, m, Ar), 7.14 – 7.23 (2H, m, Ar), 7.05 (2H, d, J = 8.7 Hz, Ar), 6.58 (2H, d, J = 8.7 Hz, Ar), 5.74 – 5.80 (1H, m, C8-H), 5.60 (1H, dd, J = 10.5, 2.3 Hz, C7-H), 4.65 (1H, d, J = 8.1 Hz, C2-H), 4.00 – 4.08 (1H, m, C5-H_A), 3.72 – 3.79 (1H, m, C5-H_B), 3.72 (3H, s, OMe), 2.17 – 2.25 (1H, m, C3-H), 2.10 – 2.17 (1H, m, C6-H), 2.06 (1H, dtd, J = 12.6, 6.3, 2.7 Hz, C4-H_A), 1.88 – 1.96 (2H, m, C9-H_2), 1.76 – 1.86 (1H, m, C4-H_B), 1.59 – 1.65 (1H, m, C11-H_A), 1.36 – 1.48 (2H, m, C10-H_2), 1.15 – 1.29 (1H, m, C11-H_B). **^{13}C NMR**

(125 MHz, CDCl$_3$) δ 159.1 (C Ar), 147.4 (C Ar), 134.3 (C Ar), 133.0 (C Ar), 132.4 (C Ar), 131.1 (C Ar), 130.9 (C Ar), 129.8 (C8), 129.1 (2 × C Ar), 127.6 (C7), 123.5 (C Ar), 113.7 (2 × C Ar), 66.5 (C2), 55.4 (OMe), 54.9 (C3), 49.8 (C5), 36.1 (C6), 28.6 (C9), 27.9 (C4), 25.3 (C11), 21.8 (C10). **NOESY--2D (500 MHz, CDCl$_3$)**: between C2-H and C7-H.

Partial data for **3n-B** (from the mixture): **^{1}H NMR (500 MHz, CDCl$_3$) δ** 7.42 – 7.51 (2H, m, Ar), 7.16 – 7.23 (2H, m, Ar), 7.06 (2H, d, *J* = 8.7 Hz, Ar), 6.59 (2H, d, *J* = 8.7 Hz, Ar), 5.62 – 5.66 (1H, m, C8-H), 5.39 (1H, dd, *J* = 10.2, 2.3 Hz, C7-H), 4.67 (1H, d, *J* = 8.4 Hz, C2-H), 4.00 – 4.08 (1H, m, C5-H$_A$), 3.72 – 3.79 (1H, m, C5-H$_B$), 3.72 (3H, s, OMe), 2.17 – 2.25 (1H, m, C3-H), 2.10 – 2.17 (1H, m, C6-H), 2.00 (1H, dtd, *J* = 12.4, 6.6, 3.2 Hz, C4-H$_A$), 1.88 – 1.96 (2H, m, C9-H$_2$), 1.76 – 1.86 (1H, m, C4-H$_B$), 1.68 – 1.76 (2H, m, C10-H$_A$ + C11-H$_A$), 1.59 – 1.65 (1H, m, C10-H$_B$), 1.15 – 1.29 (1H, m, C11-H$_B$). **^{13}C NMR (125 MHz, CDCl$_3$) δ** 159.1 (C Ar), 147.4 (C Ar), 134.3 (C Ar), 133.0 (C Ar), 132.4 (C Ar), 130.9 (C Ar), 130.9 (C Ar), 129.6 (C7), 129.1 (2 C Ar), 128.7 (C8), 123.5 (C Ar), 113.7 (2 × C Ar), 66.5 (C2), 55.4 (OMe), 54.8 (C3), 49.5 (C5), 36.3 (C6), 27.8 (C4), 25.8 (C9), 25.2 (C11), 21.9 (C10). **NOESY- 2D (500 MHz, CDCl$_3$)**: between C2-H and C7-H.

Data for **3n**: **R$_f$** 0.40 (35% EtOAc – pentane). **HRMS** (ESI): calculated for $C_{23}H_{27}N_2O_5S$ [M+H]$^+$ requires *m/z* 443.1641, found *m/z* 443.1635. **IR** (film) ν_{max}: 2980, 1541, 1512, 1370, 1159 cm^{-1}.

(3o): (±)-(2*S*,3*R*)-3-[(1'*R*)-Cyclopent-2-en-1-yl]-2-(4-methoxyphenyl)-1-[(2-nitrophenyl)sulfonyl]-pyrrolidine and (±)-(2*S*,3*R*)-3-[(1'*S*)-Cyclopent-2-en-1-yl]-2-(4-methoxyphenyl)-1-[(2-nitro-phenyl)sulfonyl]pyrrolidine

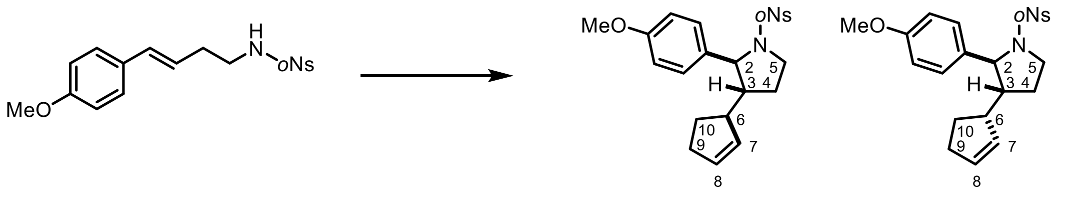

1c

3o
d.r. (C-6) 3.5:1

Prepared according to **General Procedure K** using **1c** (30 mg, 0.083 mmol) and **3o** (14 mg, 0.17 mmol, 2.0 equiv.). Flash column chromatography (gradient elution 15% → 20% EtOAc – pentane) afforded **3o** as an inseparable 3.5:1 mixture of diastereomers as a colourless oil (33 mg, 93%).

Data for **3o-A** (from the mixture): **^{1}H NMR (500 MHz, CDCl$_3$) δ** 7.49 (1H, dd, *J* = 7.9, 1.4 Hz, Ar), 7.42 – 7.46 (1H, m, Ar), 7.15 – 7.23 (2H, m, Ar), 7.05 (2H, d, *J* = 8.7 Hz, Ar), 6.59 (2H, d, *J* = 8.7 Hz, Ar), 5.77 – 5.81 (1H, m, C8-H), 5.61 – 5.64 (1H, m, C7-H), 4.57 (1H, d, *J* = 7.9 Hz, C2-H), 4.03 (1H, ddd, *J* = 10.6, 7.9, 3.0 Hz, C5-H$_A$), 3.72 – 3.79 (1H, m, C5-H$_B$), 3.72 (3H, s, OMe), 2.66 – 2.74 (1H, m, C3-H), 2.14 – 2.28 (3H, m, C6-H + C9-H$_2$), 2.02 – 2.10 (1H, m, C4-H$_A$), 1.83 – 1.91 (1H, m, C10-H$_A$), 1.71 – 1.81 (1H, m, C4-H$_B$), 1.23 – 1.33 (1H, m, C10-H$_B$). **^{13}C NMR (125 MHz, CDCl$_3$) δ** 159.1

(C Ar), 147.4 (C Ar), 134.2 (C Ar), 132.9 (C8), 132.8 (C Ar), 132.5 (C Ar), 131.5 (C7), 130.9 (C Ar), 130.9 (C Ar), 129.0 (2 × C Ar), 123.5 (C Ar), 113.6 (2 × C Ar), 67.9 (C2), 55.4 (OMe), 55.0 (C6), 49.6 (C5), 47.0 (C3), 32.2 (C9), 28.7 (C10), 28.5 (C4). **NOESY- 2D (500 MHz, $CDCl_3$)**: between C2-H and C7-H.

Data for **3o-B** (from the mixture): **^{1}H NMR (500 MHz, $CDCl_3$) δ** 7.49 (1H, dd, *J* = 7.9, 1.4 Hz, Ar), 7.42 – 7.46 (1H, m, Ar), 7.15 – 7.23 (2H, m, Ar), 7.06 (2H, d, *J* = 8.7 Hz, Ar), 6.59 (2H, d, *J* = 8.7 Hz, Ar), 5.65 – 5.69 (1H, m, C8-H), 5.41 – 5.45 (1H, m, C7-H), 4.58 (1H, d, *J* = 8.1 Hz, C2-H), 3.99 – 4.05 (1H, m, C5-H_A), 3.72 – 3.79 (1H, m, C5-H_B), 3.72 (3H, s, OMe), 2.66 – 2.74 (1H, m, C3-H), 2.14 – 2.28 (3H, m, C6-H + C9-H_2), 2.02 – 2.10 (1H, m, C4-H_A), 1.94 – 2.01 (1H, m, C10-H_A), 1.71 – 1.81 (1H, m, C4-H_B), 1.48 (1H, ddt, *J* = 13.2, 8.9, 6.8 Hz, C10-H_B). **^{13}C NMR (125 MHz, $CDCl_3$) δ** 159.2 (C Ar), 147.4 (C Ar), 132.8 (C Ar), 132.8 (C Ar), 132.5 (C7), 132.5 (C Ar), 132.0 (C8), 130.9 (C Ar), 130.9 (C Ar), 129.0 (2 × C Ar), 123.6 (C Ar), 113.7 (2 × C Ar), 67.7 (C2), 55.4 (OMe), 54.7 (C6), 49.4 (C5), 46.8 (C3), 32.1 (C9), 28.5 (C4), 26.8 (C10). **NOESY- 2D (500 MHz, $CDCl_3$)**: between C2-H and C7-H.

Data for **3o**: **R_f** 0.40 (35% EtOAc – pentane). **HRMS** (ESI): calculated for $C_{22}H_{25}N_2O_5S$ $[M+H]^+$ requires *m/z* 429.1484, found *m/z* 429.1476. **IR** (film) ν_{max}: 2980, 2890, 1541, 1511, 1371, 1159 cm^{-1}.

(3p): (±)-(2*S*,3*R*)-2-(4-Methoxyphenyl)-3-[(1'*R*)-3-methylcyclopent-2-en-1-yl]-1-[(2-nitrophenyl)-sulfonyl]pyrrolidine and (±)-(2*S*,3*R*)-2-(4-Methoxyphenyl)-3-[(1'*S*)-3-methylcyclopent-2-en-1-yl]-1-[(2-nitrophenyl)sulfonyl]pyrrolidine

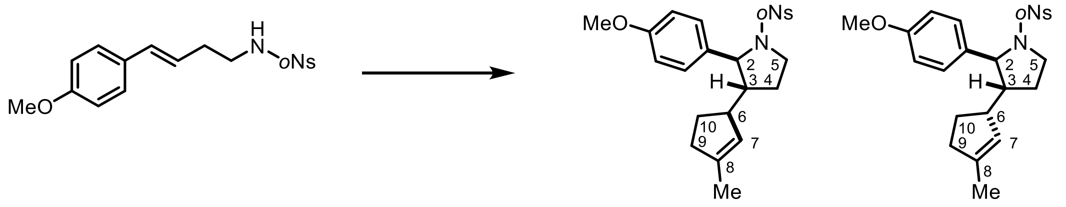

1c A 3p B
d.r. (C-6) 13:1

Prepared according to **General Procedure K** using **1c** (30 mg, 0.083 mmol) and **2p** (16 mg, 0.17 mmol, 2.0 equiv.). Flash column chromatography (gradient elution 10% → 20% EtOAc – pentane) afforded **3p** as an inseparable 13:1 mixture of diastereomers as a colourless oil (13 mg, 36%).

Data for **3p-A** (from the mixture): **^{1}H NMR (500 MHz, $CDCl_3$) δ** 7.42 – 7.51 (2H, m, Ar), 7.16 – 7.23 (2H, m, Ar), 7.04 (2H, d, *J* = 8.7 Hz, Ar), 6.58 (2H, d, *J* = 8.7 Hz, Ar), 5.20 – 5.24 (1H, m, C7-H), 4.54 (1H, d, *J* = 7.9 Hz, C2-H), 4.03 (1H, ddd, *J* = 10.7, 8.0, 3.0 Hz, C5-H_A), 3.73 – 3.79 (1H, m, C5-H_B), 3.72 (3H, s, OMe), 2.62 – 2.72 (1H, m, C6-H), 2.10 – 2.20 (3H, m, C3-H + C9-H_2), 2.05 (1H, dtd, *J* = 12.5, 6.2, 3.1 Hz, C4-H_A), 1.86 – 1.94 (1H, m, C10-H_A), 1.76 (dtd, J = 12.3, 9.8, 8.0 Hz, C4-H_B), 1.69 (1H, s, Me), 1.29 – 1.37 (1H, m, C10-H_B). **^{13}C NMR (125 MHz, $CDCl_3$) δ** 159.1 (C Ar), 147.5 (C Ar), 142.8 (C Ar), 134.3 (C Ar), 133.0 (C8), 132.4 (C Ar), 131.0 (C Ar), 130.9 (C Ar), 129.1

(2 × C Ar), 125.3 (C7), 123.5 (C Ar), 113.6 (2 × C Ar), 67.6 (C2), 55.4 (C3 + OMe), 49.7 (C5), 47.1 (C6), 36.6 (C9), 29.5 (C10), 28.5 (C4), 16.8 (Me).

Partial data for **3p-B** (from the mixture): **^{1}H NMR (500 MHz, $CDCl_3$) δ** 7.42 – 7.51 (2H, m, Ar), 7.16 – 7.23 (2H, m, Ar), 7.04 (2H, d, *J* = 8.7 Hz, Ar), 6.59 (2H, d, *J* = 8.7 Hz, Ar), 5.03 – 5.07 (1H, m, C7-H), 4.57 (1H, d, *J* = 7.9 Hz, C2-H), 4.00 – 4.05 (1H, m, C5-H_A), 3.73 – 3.79 (1H, m, C5-H_B), 3.72 (3H, s, OMe), 2.62 – 2.72 (1H, m, C6-H), 2.10 – 2.20 (3H, m, C3-H + C9-H_2), 2.02 – 2.08 (1H, m, C4-H_A), 1.97 – 2.00 (1H, m, C10-H_A), 1.72 – 1.81 (1H, m, C4-H_B), 1.64 (1H, s, Me), 1.29 – 1.37 (1H, m, C10-H_B). **^{13}C NMR (125 MHz, $CDCl_3$) δ** 159.1 (C Ar), 147.5 (C Ar), 141.9 (C Ar), 134.3 (C Ar), 133.0 (C8), 132.4 (C Ar), 131.0 (C Ar), 130.9 (C Ar), 129.0 (2 × C Ar), 126.3 (C7), 123.5 (C Ar), 113.7 (2 × C Ar), 67.7 (C2), 55.4 (OMe), 54.7 (C3), 49.4 (C5), 46.9 (C6), 36.4 (C9), 28.4 (C10), 27.7 (C4), 16.8 (Me).

Data for **3p**: **R_f** 0.50 (25% EtOAc – pentane). **HRMS** (ESI): calculated for $C_{23}H_{27}N_2O_5S$ $[M+H]^+$ requires *m/z* 443.1641, found *m/z* 443.1635. **IR** (film) ν_{max}: 2980, 2889, 1543, 1512, 1374, 1249 cm^{-1}.

(3q): (±)-(2*S*,3*R*)-2-(4-Methoxyphenyl)-1-[(2-nitrophenyl)sulfonyl)-3-[(1'*R*)-3-phenylcyclopent-2-en-1-yl]pyrrolidine

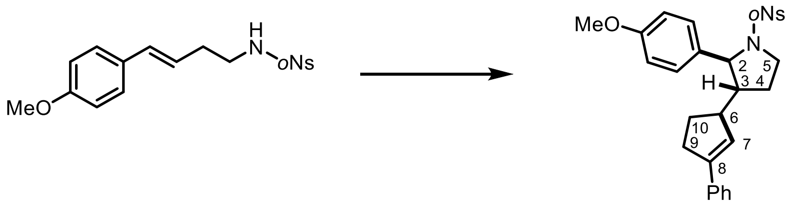

1c **3q**

Prepared according to **General Procedure K** using **1c** (31 mg, 0.086 mmol) and **2q** (14 mg, 0.086 mmol) at 0 °C for 48 h. Flash column chromatography (gradient elution 15% → 20% EtOAc – pentane) afforded **3q** as a colourless oil (43 mg, 99%).

Data for **3q: R_f** 0.40 (35% EtOAc – pentane). **^{1}H NMR (500 MHz, $CDCl_3$) δ** 7.53 (1H, dd, *J* = 7.9, 1.4 Hz, Ar), 7.45 – 7.50 (1H, m, Ar), 7.38 – 7.42 (2H, m, Ar), 7.31 – 7.36 (2H, m, Ar), 7.24 – 7.28 (2H, m, Ar), 7.19 – 7.23 (1H, m, Ar), 7.10 (2H, d, *J* = 8.7 Hz, Ar), 6.62 (2H, d, *J* = 8.7 Hz, Ar), 6.07 (1H, q, *J* = 2.0 Hz, C7-H), 4.67 (1H, d, *J* = 8.0 Hz, C2-H), 4.09 (1H, ddd, *J* = 10.7, 8.0, 2.9 Hz, C5-H_A), 3.79 – 3.85 (1H, m, C5-H_B), 3.75 (3H, s, OMe), 2.93 – 3.00 (1H, m, C6-H), 2.61 – 2.72 (2H, m, C9-H_2), 2.35 (1H, ddt, *J* = 10.1, 8.0, 6.5 Hz, C3-H), 2.13 – 2.19 (1H, m, C4-H_A), 2.09 (1H, ddt, *J* = 13.2, 8.4, 4.2 Hz, C10-H_A), 1.82 – 1.91 (1H, m, C4-H_B), 1.54 (1H, ddt, *J* = 13.3, 8.6, 6.9 Hz, C10-H_B). **^{13}C NMR (125 MHz, $CDCl_3$) δ** 159.2 (C Ar), 147.4 (C Ar), 144.2 (C Ar), 136.2 (C Ar), 134.2 (C Ar), 132.9 (C8), 132.5 (C Ar), 131.0 (C Ar), 130.9 (C Ar), 129.1 (2 × C Ar), 128.4 (C Ar), 127.5 (C7), 126.5 (2 × C Ar), 125.8 (C Ar), 123.6 (2 × C Ar), 113.7 (2 × C Ar), 67.8 (C2), 55.4 (OMe), 55.1 (C3), 49.7 (C5), 47.6

(C6), 32.9 (C9), 28.8 (C10), 28.7 (C4). **NOESY- 2D (500 MHz, $CDCl_3$)**: between C2-H and C6-H, between C2-H and C7-H, between C2-H and C10-H_A, between C2-H and C10-H_B. **HRMS** (ESI): calculated for $C_{28}H_{29}N_2O_5S$ $[M+H]^+$ requires *m/z* 505.1797, found *m/z* 505.1792. **IR** (film) ν_{max}: 2980, 2889, 1729, 1514, 1369, 1150 cm^{-1}.

(3r): (±)-{(2*S*,3*S*)-2-(4-Methoxyphenyl)-1-[(2-nitrophenyl)sulfonyl]pyrrolidin-3-yl}methanol

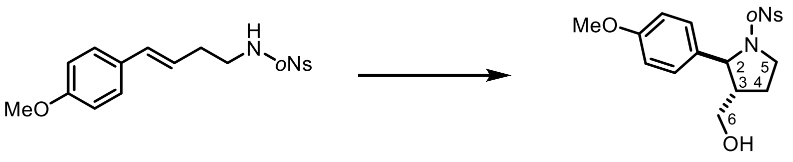

1c **3r**

Prepared according to **General Procedure K** using **1c** (26 mg, 0.071 mmol) and paraformaldehyde (43 mg, 1.43 mmol, 20 equiv.) at 0 °C for 48 h. Flash column chromatography (gradient elution 20% → 30% EtOAc – pentane) afforded **3r** as a colourless oil (20 mg, 74%).

Data for **3r: R_f** 0.20 (30% EtOAc – pentane). **^{1}H NMR (500 MHz, $CDCl_3$) δ** 7.46 – 7.53 (2H, m, Ar), 7.34 (1H, dd, *J* = 8.0, 1.3 Hz, Ar), 7.22 – 7.27 (1H, m, Ar), 7.07 (2H, d, *J* = 8.7 Hz, Ar), 6.62 (2H, d, *J* = 8.7 Hz, Ar), 4.70 (1H, d, *J* = 6.9 Hz, C2-H), 3.99 (1H, ddd, *J* = 10.3, 8.0, 4.2 Hz, C5-H_A), 3.76 – 3.84 (1H, m, C5-H_B), 3.72 (3H, s, OMe), 3.59 – 3.64 (1H, m, C6-H_A), 3.53 – 3.59 (1H, m, C6-H_B), 2.33 – 2.41 (1H, m, C3-H), 2.10 – 2.19 (1H, m, C4-H_A), 1.85 (1H, dq, *J* = 12.5, 8.4 Hz, C4-H_B), 1.60 (1H, *br.* s, *O*H). **^{13}C NMR (125 MHz, $CDCl_3$) δ** 159.2 (C Ar), 147.5 (C Ar), 133.8 (C Ar), 132.8 (C Ar), 132.6 (C Ar), 131.1 (C Ar), 131.1 (C Ar), 128.5 (2 × C Ar), 123.7 (C Ar), 113.8 (2 × C Ar), 65.7 (C2), 63.0 (C6), 55.4 (OMe), 52.3 (C3), 49.4 (C5), 27.7 (C4). **NOESY- 2D (500 MHz, $CDCl_3$)**: between C2-H and C6-H_A, between C2-H and C6-H_B. **HRMS** (ESI): calculated for $C_{18}H_{20}N_2O_6SNa$ $[M+Na]^+$ requires *m/z* 415.0940, found *m/z* 415.0925. **IR** (film) ν_{max}: 3649, 2980, 2888, 2360, 1382, 1154 cm^{-1}.

(3s): (±)-(2*S*,3*R*)-3-Benzhydryl-1-[(2-nitrophenyl)sulfonyl]-2-phenylpyrrolidine

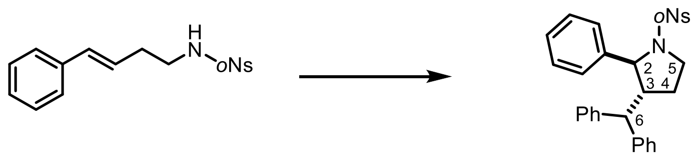

1g **3s**

Prepared according to **General Procedure K** using **1g** (41 mg, 0.12 mmol) and benzhydrol (23.5 mg, 0.12 mmol). Flash column chromatography (gradient elution 8% → 16% EtOAc – pentane) afforded **3s** as a colourless oil (61 mg, 99%).

Data for **3s:** $\mathbf{R_f}$ 0.50 (30% EtOAc – pentane). **^{1}H NMR (500 MHz, $CDCl_3$) δ** 7.46 – 7.53 (2H, m, Ar), 7.34 (1H, dd, *J* = 8.0, 1.3 Hz, Ar), 7.22 – 7.27 (4H, m, Ar), 7.08 – 7.21 (7H, m, Ar), 6.97 – 7.04 (3H, m, Ar), 6.80 – 6.84 (2H, m, Ar), 4.71 (1H, d, *J* = 3.2 Hz, C2-H), 3.99 (1H, dt, *J* = 10.1, 7.6 Hz, C5-H_A), 3.91 (1H, ddd, *J* = 10.1, 7.8, 4.7 Hz, C5-H_B), 3.80 (1H, d, *J* = 11.7 Hz, C6-H), 3.04 – 3.12 (1H, m, C3-H), 2.08 – 2.17 (1H, m, C4-H_A), 1.69 – 1.73 (1H, m, C4-H_B). **^{13}C NMR (125 MHz, $CDCl_3$) δ** 147.7 (C Ar), 142.9 (C Ar), 142.9 (C Ar), 141.6 (C Ar), 133.3 (C Ar), 133.0 (C Ar), 131.149 (CH9 Ar), 131.1 (C Ar), 128.9 (2 × C Ar), 128.8 (2 × C Ar), 128.3 (2 × C Ar), 128.2 (2 × C Ar), 127.9 (2 × C Ar), 127.1 (C Ar), 126.8 (C Ar), 126.8 (C Ar), 126.4 (2 × C Ar), 123.6 (C Ar), 67.3 (C2), 54.8 (C6), 53.6 (C3), 48.6 (C5), 28.0 (C4). **HRMS** (ESI): calculated for $C_{29}H_{27}N_2O_4S$ $[M+H]^+$ requires *m/z* 499.1692, found *m/z* 499.1684. **IR** (film) ν_{max}: 2980, 2890, 2360, 1710, 1542, 1164 cm^{-1}.

(3t): (±)-(2*S*,3*S*)-3-(4-Methoxybenzyl)-1-[(2-nitrophenyl)sulfonyl]-2-phenylpyrrolidine

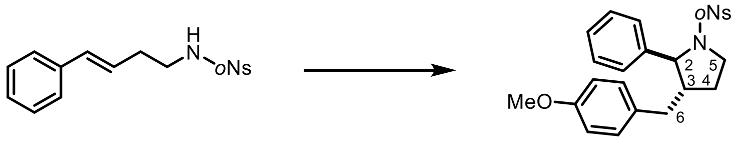

1g **3t**

Prepared according to **General Procedure K** using **1g** (72 mg, 0.22 mmol) and 4-methoxybenzyl alcohol (31 mg, 0.22 mmol). Flash column chromatography (gradient elution 10% → 20% EtOAc – pentane) afforded **3t** as a colourless oil 86 mg, 85%).

Data for **3t:** $\mathbf{R_f}$ 0.50 (35% EtOAc – pentane). **^{1}H NMR (500 MHz, $CDCl_3$) δ** 7.51 (1H, dd, *J* = 8.0, 1.5 Hz, Ar), 7.43 – 7.48 (1H, m, Ar), 7.26 (1H, dd, *J* = 8.0, 1.5 Hz, Ar) 7.14 – 7.20 (1H, m, Ar), 7.04 – 7.10 (5H, m, Ar), 6.96 (2H, d, *J* = 8.7 Hz, Ar), 6.78 (2H, d, *J* = 8.7 Hz, Ar), 4.56 (1H, d, *J* = 6.8 Hz, C2-H), 4.06 (1H, ddd, *J* = 10.3, 7.7, 4.1 Hz, C5-H_A), 3.77 – 3.82 (1H, m, C5-H_B), 3.77 (OMe), 2.71 (1H, dd, *J* = 13.3, 5.5 Hz, C6-H_A), 2.48 (1H, dd, *J* = 13.3, 9.0 Hz, C6-H_B), 2.35 – 2.43 (1H,

m, C3-H), 1.97 – 2.06 (1H, m, C4-H_A), 1.72 (1H, dq, *J* = 12.3, 7.7 Hz, C4-H_B). **^{13}C NMR (125 MHz, $CDCl_3$) δ** 158.2 (C Ar), 147.5 (C Ar), 140.4 (C Ar), 133.8 (C Ar), 132.8 (C Ar), 131.4 (C Ar), 131.0 (C Ar), 130.9 (C Ar), 129.9 (2 × C Ar), 128.3 (2 × C Ar), 127.6 (C Ar), 127.3 (2 × C Ar), 123.6 (C Ar), 114.0 (2 × C Ar), 68.8 (C2), 55.3 (OMe), 52.4 (C3), 49.5 (C5), 37.1 (C6), 30.2 (C4). **HRMS** (ESI): calculated for $C_{24}H_{24}N_2O_5SNa$ $[M+Na]^+$ requires *m/z* 475.1304, found *m/z* 475.1295. **IR** (film) ν_{max}: 3029, 2918, 1710, 1542, 1359, 1161 cm^{-1}.

(3u): (±)-(2*S*,3*R*)-3-Benzhydryl-2-(4-bromophenyl)-1-[(2-nitrophenyl)sulfonyl]pyrrolidine

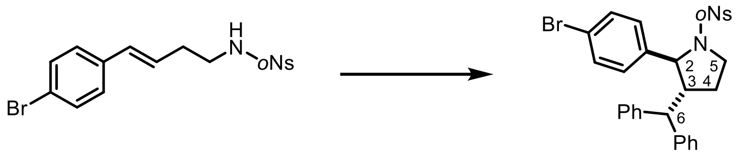

1h 3u

Prepared according to **General Procedure K** using **1h** (15.5 mg, 0.038 mmol) and benzhydrol (7.0 mg, 0.038 mmol). Flash column chromatography (gradient elution 10% → 20% EtOAc – pentane) afforded **3u** as a colourless oil (19 mg, 87%).

Data for **3u: R_f** 0.40 (25% EtOAc – pentane). **^{1}H NMR (500 MHz, $CDCl_3$) δ** 7.52 – 7.58 (2H, m, Ar), 7.33 – 7.37 (1H, m, Ar), 7.21 – 7.29 (5H, m, Ar), 7.12 – 7.19 (4H, m, Ar), 7.05 – 7.10 (4H, m, Ar), 6.67 (2H, d, *J* = 8.4 Hz, Ar), 4.64 (1H, d, *J* = 3.8 Hz, C2-H), 3.98 (1H, dt, *J* = 10.2, 7.4 Hz, C5-H_A), 3.88 (1H, ddd, *J* = 10.2, 7.6, 5.2 Hz, C5-H_B), 3.79 (1H, d, *J* = 11.7 Hz, C6-H), 3.02 – 3.09 (1H, m, C3-H), 2.05 – 2.14 (1H, m, C4-H_A), 1.74 (1H, ddt, *J* = 12.6, 7.2, 5.4 Hz, C4-H_B). **^{13}C NMR (125 MHz, $CDCl_3$) δ** 147.7 (C Ar), 142.7 (C Ar), 142.7 (C Ar), 140.8 (C Ar), 133.3 (C Ar), 133.1 (C Ar), 131.3 (C Ar), 131.2 (2 × C Ar), 131.1 (C Ar), 129.0 (2 × C Ar), 128.8 (2 × C Ar), 128.4 (2 × C Ar), 128.3 (2 × C Ar), 127.8 (2 × C Ar), 127.0 (C Ar), 126.9 (C Ar), 123.7 (C Ar), 121.0 (C Ar), 67.0 (C2), 55.2 (C6), 53.8 (C3), 48.9 (C5), 28.6 (C4). **NOESY- 2D (500 MHz, $CDCl_3$)**: between C2-H and C6-H. **HRMS** (ESI): calculated for $C_{29}H_{26}N_2O_4SBr$ $[M+H]^+$ requires *m/z* 577.0797, found *m/z* 577.0791. **IR** (film) ν_{max}: 2922, 1709, 1543, 1359, 1163, 1221 cm^{-1}.

(3v): (±)-3-Benzhydryl-2,2-dimethyl-1-[(2-nitrophenyl)sulfonyl]pyrrolidine

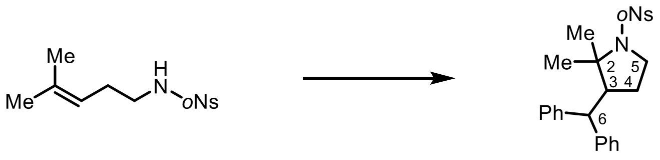

1i **3v**

Prepared according to **General Procedure K** using **1i** (29 mg, 0.10 mmol) and benzhydrol (19 mg, 0.10 mmol). Flash column chromatography (gradient elution 15% → 20% EtOAc – pentane) afforded **3v** as a colourless oil (43 mg, 93%).

Data for **3v:** $\mathbf{R_f}$ 0.30 (35% EtOAc – pentane). **^{1}H NMR (400 MHz, $CDCl_3$) δ** 8.01 (1H, dd, *J* = 7.6, 1.7 Hz, Ar), 7.59 – 7.67 (2H, m, Ar), 7.57 (1H, dd, *J* = 7.1, 2.1 Hz, Ar), 7.30 – 7.35 (4H, m, Ar), 7.20 – 7.29 (4H, m, Ar), 7.10 – 7.17 (2H, m, Ar), 3.74 (1H, d, *J* = 11.5 Hz, C6-H), 3.56 (1H, td, *J* = 9.6, 2.1 Hz, C5-H_A), 3.33 (td, *J* = 9.6, 7.7 Hz, C5-H_B), 2.94 (1H, td, *J* = 11.5, 6.5 Hz, C3-H), 1.71 – 1.79 (1H, m, C4-H_A), 1.55 (3H, s, Me), 1.48 – 1.58 (1H, m, C4-H_B), 1.40 (3H, s, Me). **^{13}C NMR (101 MHz, $CDCl_3$) δ** 148.3 (C Ar), 144.1 (C Ar), 142.8 (C Ar), 135.8 (C Ar), 133.3 (C Ar), 131.5 (C Ar), 130.4 (C Ar), 128.9 (2 × C Ar), 128.8 (2 × C Ar), 128.3 (2 × C Ar), 127.8 (2 × C Ar), 126.9 (C Ar), 126.7 (C Ar), 123.9 (C Ar), 68.6 (C2), 53.9 (C6), 53.4 (C3), 47.0 (C5), 28.5 (C4), 27.5 (Me), 22.2 (Me). **HRMS** (ESI): calculated for $C_{25}H_{26}N_2O_4SNa$ $[M+Na]^+$ requires *m/z* 473.1511, found *m/z* 473.1506. **IR** (film) ν_{max}: 2980, 1542, 1370, 1337, 1216, 1159 cm^{-1}.

(3w): (±)- 4-Benzhydryl-1-[(2-nitrophenyl)sulfonyl]-1-azaspiro[4.4]nonane

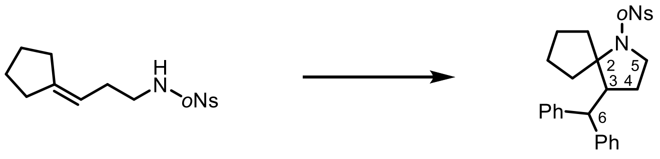

1j **3w**

Prepared according to **General Procedure K** using **1j** (32.5 mg, 0.11 mmol) and benzhydrol (19 mg, 0.11 mmol). Flash column chromatography (gradient elution 15% → 25% EtOAc – pentane) afforded **3w** as a colourless oil (50 mg, 99%).

Data for **3w:** $\mathbf{R_f}$ 0.30 (30% EtOAc – pentane). **^{1}H NMR (400 MHz, $CDCl_3$) δ** 8.00 – 8.05 (1H, m, Ar), 7.62 – 7.70 (3H, m, Ar), 7.33 – 7.37 (2H, m, Ar), 7.19 – 7.28 (6H, m, Ar), 7.09 – 7.16 (2H, m, Ar), 3.87 (1H, d, *J* = 10.5 Hz, C6-H), 3.41 (1H, ddd, *J* = 9.9, 8.8, 5.3 Hz, C5-H_A), 3.34 (1H, ddd, *J* = 9.9, 8.3, 6.7 Hz, C5-H_B), 3.07 (1H, ddd, *J* = 10.5, 8.5, 6.3 Hz, C3-H), 2.00 – 2.11 (2H, m, 2 × C-H), 1.90 (1H, ddd, *J* = 13.5, 8.3, 5.3 Hz, C4-H_A), 1.74 – 1.84 (1H, m, C-H), 1.65 – 1.74 (1H, m, C-H), 1.51 – 1.64

(3H, m, 3 × C-H), 1.42 – 1.50 (1H, m, C4-H_B), 1.17 – 1.29 (1H, m, C-H), 1.00 – 1.10 (1H, m, C-H). **^{13}C NMR (101 MHz, $CDCl_3$) δ** 149.7 (C Ar), 144.6 (C Ar), 144.1 (C Ar), 135.4 (C Ar), 133.4 (C Ar), 131.7 (C Ar), 130.3 (C Ar), 128.8 (2 × C Ar), 128.8 (2 × C Ar), 128.0 (2 × C Ar), 128.0 (2 × C Ar), 126.6 (C Ar), 126.5 (C Ar), 124.3 (C Ar), 80.1 (C2), 52.7 (C6), 51.5 (C3), 47.1 (C5), 36.6 (CH_2), 33.3 (CH_2), 28.2 (C4), 25.0 (CH_2), 24.3 (CH_2). **HRMS** (ESI): calculated for $C_{27}H_{28}N_2O_4SNa$ $[M+Na]^+$ requires *m/z* 499.1667, found *m/z* 499.1660. **IR** (film) ν_{max}: 2980, 2888, 1710, 1541, 1371, 1159 cm^{-1}.

(3x): (±)-(2*R*,3*R*)-3-Benzhydryl-2-[(1'*E*)-4-methoxystyryl]-1-[(2-nitrophenyl)sulfonyl]-pyrrolidine and (±)-(2*R*,3*S*)-3-Benzhydryl-2-[(1'*E*)-4-methoxystyryl]-1-[(2-nitrophenyl)sulfonyl]-pyrrolidine

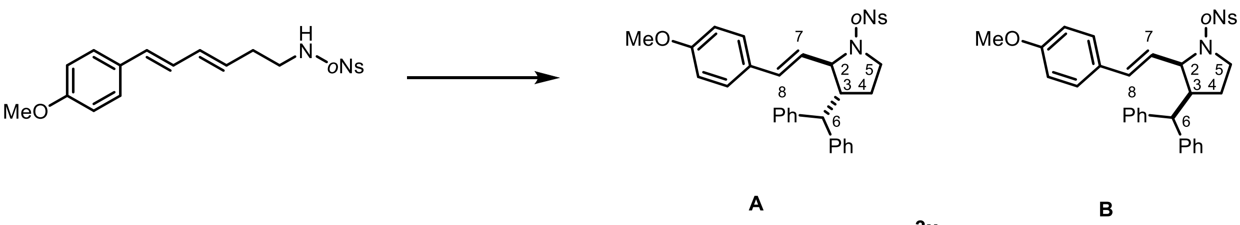

1k

3x
d.r. (C2-C3) 5.5:1

Prepared according to **General Procedure K** using **1k** (15 mg, 0.040 mmol) and benzhydrol (7 mg, 0.040 mmol). Flash column chromatography (gradient elution 10% → 20% EtOAc – pentane) afforded **3w** as a partially separable 5.5:1 mixture of diastereomers, **3x-A** was isolated as a colourless oil (3 mg, 15%) and a 4.5:1 mixture of **3x-A** + **3x-B** was isolated as a colourless oil (17 mg, 78%).

Data for **3x-A: R_f** 0.45 (30% EtOAc – pentane). **^{1}H NMR (500 MHz, $CDCl_3$) δ** 7.83 (1H, dd, *J* = 8.0, 1.3 Hz, Ar), 7.55 (1H, dd, *J* = 8.0, 1.4 Hz, Ar), 7.47 – 7.52 (1H, m, Ar), 7.31 – 7.36 (1H, m, Ar), 7.22 – 7.29 (4H, m, Ar), 7.14 – 7.21 (5H, m, Ar), 7.06 – 7.11 (1H, m, Ar), 6.94 (2H, d, *J* = 8.7 Hz, Ar), 6.74 (2H, d, *J* = 8.7 Hz, Ar), 5.92 (1H, d, *J* = 15.7 Hz, C8-H), 5.57 (1H, dd, *J* = 15.7, 7.8 Hz, C7-H), 4.27 (1H, dd, *J* = 7.8, 3.6 Hz, C2-H), 3.87 (1H, dt, *J* = 10.2, 7.2 Hz, C5-H_A), 3.79 (OMe), 3.75 (1H, d, *J* = 11.5 Hz, C6-H), 3.62 (1H, ddd, *J* = 10.2, 7.2, 5.7 Hz, C5-H_B), 2.92 – 3.00 (1H, m, C3-H), 2.07 (1H, dq, *J* = 13.8, 7.2 Hz, C4-H_A), 1.61 – 1.69 (1H, m, C4-H_B). **^{13}C NMR (125 MHz, $CDCl_3$) δ** 159.4 (C Ar), 148.1 (C Ar), 143.1 (C Ar), 142.9 (C Ar), 134.2 (C Ar), 133.1 (C Ar), 131.7 (C Ar), 131.4 (C Ar), 131.4 (C8), 129.1 (C Ar), 128.9 (2 × C Ar), 128.8 (2 × C Ar), 128.3 (2 × C Ar), 128.0 (2 × C Ar), 127.7 (2 × C Ar), 126.9 (C Ar), 126.8 (C Ar), 125.9 (C7), 123.8 (C Ar), 113.8 (2 × C Ar), 66.3 (C2), 55.4 (OMe), 54.5 (C6), 50.5 (C3), 48.0 (C5), 28.9 (C4). **NOESY- 2D (500 MHz, $CDCl_3$)**: between C2-H and C6-H, between C3-H and C7-H, between C3-H and C8-H. **HRMS** (ESI): calculated for $C_{32}H_{30}N_2O_5SNa$ $[M+Na]^+$ requires *m/z* 577.1773, found *m/z* 577.1761. **IR** (film) ν_{max}: 2980, 2888, 2360, 1381, 1251, 1158 cm^{-1}.

Data for **3x-B** (from the mixture): **R_f** 0.40 (30% EtOAc – pentane). **^{1}H NMR (500 MHz, $CDCl_3$) δ** 7.93 – 7.97 (1H, m, Ar), 7.43 – 7.49 (2H, m, Ar), 7.08 – 7.39 (11H, m, Ar), 7.02 (2H, d, *J* = 8.7 Hz, Ar), 6.82 (2H, d, *J* = 8.7 Hz, Ar), 5.84 (1H, d, *J* = 15.7 Hz, C8-H), 5.71 (1H, dd, *J* = 15.7, 7.8 Hz, C7-H), 4.65 (1H, t, *J* = 7.4 Hz, C2-H), 3.83 (OMe), 3.74 – 3.79 (1H, m, C5-H_A), 3.62 – 3.64 (1H, m, C6-H), 3.48 (1H, td, *J* = 10.2, 7.2 Hz, C5-H_A), 3.17 – 3.27 (1H, m, C3-H), 1.75 – 1.89 (1H, m, C4-H_2). **^{13}C NMR (125 MHz, $CDCl_3$) δ** 159.4 (C Ar), 148.2 (C Ar), 143.5 (C Ar), 142.4 (C Ar), 133.3 (C Ar), 133.3 (C8), 131.7 (C Ar), 131.3 (C Ar), 129.0 (2 × C Ar), 128.9 (2 × C Ar), 128.8 (2 × C Ar), 128.1 (2 × C Ar), 127.8 (2 × C Ar), 127.7 (C Ar + 2 × C Ar), 126.7 (C Ar), 123.8 (C Ar), 121.8 (C7), 114.0 (2 × C Ar), 64.1 (C2), 55.4 (OMe), 53.0 (C6), 48.5 (C3), 47.5 (C5), 29.3 (C4).

(3y): (±)-(2*R*,3*R*)-2-[(1'*E*)-4-Methoxystyryl]-1-[(2-nitrophenyl)sulfonyl]-3-[(1''*R*)-3-phenylcyclopent-2-en-1-yl]pyrrolidine and (±)-(2*R*,3*S*)-2-[(1'*E*)-4-Methoxystyryl]-1-[(2-nitrophenyl)-sulfonyl]-3-[(1''*R*)-3-phenylcyclopent-2-en-1-yl]pyrrolidine

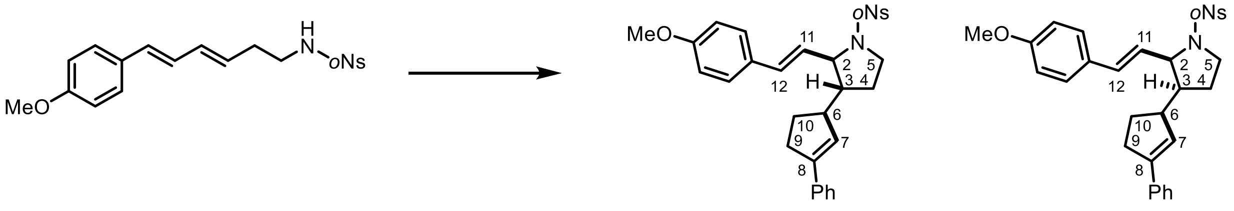

1k **A** **3y** **B**

d.r. (C2-C3) 16:1

Prepared according to **General Procedure K** using **1k** (13 mg, 0.032 mmol) and **2q** (5.0 mg, 0.032 mmol) at 0 °C for 48 h. Flash column chromatography (gradient elution 15% → 25% EtOAc – pentane) afforded **3y-A** as a colourless oil (15 mg, 86%). (Note the d.r. was determined *via* ^{1}H NMR integration of the crude mixture).

Data for **3y-A**: **R_f** 0.45 (35% EtOAc – pentane). **^{1}H NMR (500 MHz, $CDCl_3$) δ** 7.91 (1H, dd, *J* = 8.0, 1.4 Hz, Ar), 7.55 (1H, dd, *J* = 7.9, 1.3 Hz, Ar), 7.45 – 7.50 (1H, m, Ar), 7.37 – 7.41 (2H, m, Ar), 7.28 – 7.33 (3H, m, Ar), 7.20 – 7.25 (1H, m, Ar), 7.04 (2H, d, *J* = 8.7 Hz, Ar), 6.78 (2H, d, *J* = 8.7 Hz, Ar), 6.40 (1H, d, *J* = 15.7 Hz, C12-H), 6.07 (1H, q, *J* = 2.0 Hz, C7-H), 5.61 (1H, dd, *J* = 15.7, 8.7 Hz, C11-H), 4.26 (1H, t, *J* = 8.7 Hz, C2-H), 3.95 (1H, ddd, *J* = 10.8, 6.3, 3.1 Hz, C5-H_A), 3.80 (OMe), 3.53 (td, *J* = 9.9, 6.3 Hz, C5-H_B), 2.93 – 3.00 (1H, m, C6-H), 2.61 – 2.75 (2H, m, C9-H_2), 2.18 – 2.23 (1H, m, C10-H_A), 2.10 – 2.17 (1H, m, C3-H), 2.02 – 2.09 (1H, m, C4-H_A), 1.73 – 1.82 (1H, m, C4-H_B), 1.66 – 1.73 (1H, m, C10-H_B). **^{13}C NMR (125 MHz, $CDCl_3$) δ** 159.6 (C Ar), 148.0 (C Ar), 144.2 (C Ar), 136.2 (C Ar), 135.0 (C Ar), 133.2 (C12), 132.9 (C Ar), 131.6 (C Ar), 131.4 (C Ar), 128.9 (C8), 128.5 (2 × C Ar), 127.8 (2 × C Ar), 127.5 (C Ar), 126.5 (C7), 126.2 (C11), 125.8 (2 × C Ar), 123.8 (C Ar), 113.9 (2 × C Ar), 67.0 (C2), 55.4 (OMe), 51.9 (C3), 48.9 (C5), 47.4 (C6), 32.9 (C9), 29.0 (C10), 28.4 (C4). **NOESY- 2D (500 MHz, $CDCl_3$)**: between C2-H and C6-H, between C2-H and C7-H. **HRMS**

(ESI): calculated for $C_{30}H_{30}N_2O_5SNa$ $[M+Na]^+$ requires *m/z* 533.1773, found *m/z* 533.1765. **IR** (film) ν_{max}: 2980, 2889, 2360, 1461, 1381, 1251 cm^{-1}.

Data for **3y-B:** not isolated after flash column chromatography.

(3z): (±)-{(2*R*,3*S*)-1-[(2-Nitrophenyl)sulfonyl]-2-[(1'*E*)-styryl]pyrrolidin-3-yl}methanol and (±)-{(2*R*,3*R*)-1-[(2-Nitrophenyl)sulfonyl]-2-[(1'*E*)-styryl]pyrrolidin-3-yl}methanol

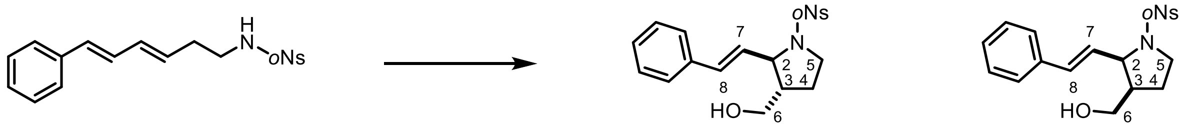

1l

A **B**

3z
d.r. (C2-C3) 10:1

Prepared according to **General Procedure K** using **1l** (9.5 mg, 0.026 mmol) and paraformaldehyde (16 mg, 0.53 mmol, 20.0 equiv.). Flash column chromatography (gradient elution 10% → 20% EtOAc – pentane) afforded **3z** as an inseparable 10:1 mixture of diastereomers as a colourless oil (3 mg, 20%).

Data for **3z-A** (from the mixture): **^{1}H NMR (500 MHz, $CDCl_3$) δ** 7.94 (1H, dd, *J* = 8.0, 1.3 Hz, Ar), 7.57 (1H, dd, *J* = 7.9, 1.3 Hz, Ar), 7.51 (1H, td, *J* = 7.7, 1.4 Hz, Ar), 7.39 (1H, td, *J* = 7.7, 1.4 Hz, Ar), 7.27 – 7.21 (3H, m, Ar), 7.17 – 7.12 (2H, m, Ar), 6.47 (1H, d, *J* = 15.8 Hz, C8-H), 5.85 (1H, dd, *J* = 15.8, 8.1 Hz, C7-H), 4.36 (1H, dd, *J* = 8.1, 6.2 Hz, C2-H), 3.88 (1H, ddd, *J* = 10.3, 8.0, 4.6 Hz, C5-H_A), 3.55 – 3.68 (3H, m, C5-H_B + C6-H_2), 2.28 (1H, dp, *J* = 7.9, 6.2 Hz, C3-H), 2.12 (1H, dtd, *J* = 11.5, 6.9, 4.6 Hz, C4-H_A), 1.83 (1H, dq, *J* = 12.6, 8.0 Hz, C4-H_B). **^{13}C NMR (126 MHz, $CDCl_3$) δ** 148.1 (C Ar), 136.0 (C Ar), 134.3 (C Ar), 133.2 (C8), 133.2 (C Ar), 131.6 (C Ar), 131.5 (C Ar), 128.6 (2 × C Ar), 128.1 (C7), 128.0 (C Ar), 126.6 (2 × C Ar), 123.9 (C Ar), 64.6 (C2), 62.8 (C6), 48.8 (C3), 48.6 (C5), 27.3 (C4).

Partial data for **3z-B** (from the mixture): **^{1}H NMR (500 MHz, $CDCl_3$) δ** 7.96 – 8.00 (1H, m, Ar), 7.49 – 7.53 (1H, m, Ar), 7.42 – 7.46 (1H, m, Ar), 7.17 – 7.27 (6H, m, Ar), 6.48 (1H, d, *J* = 15.9 Hz, C8-H), 5.93 (1H, dd, *J* = 15.8, 8.3 Hz, C7-H), 4.73 (1H, t, *J* = 8.0 Hz, C2-H), 3.84 – 3.90 (1H, m, C5-H_A), 3.74 – 3.81 (2H, m, C5-H_B + C6-H_A), 3.55 – 3.68 (1H, m, C6-H_B), 2.54 – 2.63 (1H, m, C3-H), 2.06 – 2.16 (1H, m, C4-H_A), 1.77 – 1.88 (1H, m, C4-H_B). **^{13}C NMR (125 MHz, $CDCl_3$) δ** 148.1 (C Ar), 136.0 (C Ar), 134.3 (C Ar), 133.6 (C Ar), 133.4 (C8), 131.6 (C Ar), 131.4 (C Ar), 128.7 (2 × C Ar), 128.2 (C7), 128.0 (C Ar), 126.7 (2 × C Ar), 124.8 (C Ar), 63.8 (C2), 62.1 (C6), 48.8 (C3), 46.5 (C5), 26.9 (C4).

Data for **3z**: **R_f** 0.50 (30% EtOAc – pentane). **HRMS** (ESI): calculated for $C_{19}H_{20}N_2O_5SNa$ $[M+Na]^+$ requires *m/z* 411.0991, found *m/z* 411.0985. **IR** (film) ν_{max}: 3658, 2980, 2888, 1738, 1542, 1378 cm^{-1}.

(3aa): (±)-(2*S*,3*S*)-3-Benzhydryl-1-[(2-nitrophenyl)sulfonyl]-2-[(triisopropylsilyl)methyl]-pyrrolidine and (±)-(2*S*,3*R*)-3-Benzhydryl-1-[(2-nitrophenyl)sulfonyl]-2-[(triisopropylsilyl)-methyl]pyrrolidine

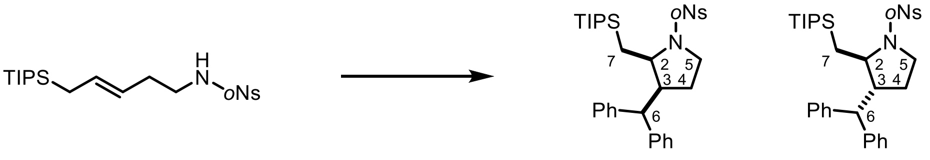

1m
E:Z
4:1

A **3aa** B
d.r. (C2-C3) 4:1

Prepared according to **General Procedure K** using **1m** (22 mg, 0.052 mmol) and benzhydrol (16 mg, 0.053 mmol) at 0 °C for 16 h. Flash column chromatography (gradient elution 10% → 20% EtOAc – pentane) afforded **3aa** as a separable 4:1 mixture of diastereomers as a colourless oil (31 mg, 99%).

Data for **3aa-A**: **R$_f$** 0.40 (50% EtOAc – pentane). **^{1}H NMR (500 MHz, CDCl$_3$) δ** 8.02 (1H, dd, *J* = 7.7, 1.6 Hz, Ar), 7.73 – 7.82 (2H, m, Ar), 7.68 (1H, dd, *J* = 7.7, 1.6 Hz, Ar), 7.24 – 7.29 (2H, m, Ar), 7.14 – 7.20 (5H, m, Ar), 7.05 – 7.12 (3H, m, Ar), 4.22 (1H, ddd, *J* = 12.6, 5.8, 2.0 Hz, C2-H), 3.71 – 3.77 (1H, m, C5-H$_A$), 3.70 (1H, d, *J* = 11.7 Hz, C6-H), 3.52 (1H, ddd, *J* = 12.9, 8.7, 4.7 Hz, C5-H$_B$), 2.43 – 2.52 (1H, m, C3-H), 1.68 – 1.78 (2H, m, C4-H$_2$), 1.11 (3H, hept, *J* = 7.5 Hz, 3 × C**H**(CH$_3$)$_2$), 0.95 (9H, d, *J* = 7.4 Hz, 3 × CH(C**H$_3$**)$_2$), 0.82 (9H, d, *J* = 7.4 Hz, 3 × CH(C**H$_3$**)$_2$), 0.76 – 0.80 (1H, m, C7-H$_A$), 0.69 (1H, dd, *J* = 14.8, 2.0 Hz, C7-H$_B$). **^{13}C NMR (125 MHz, CDCl$_3$) δ** 148.8 (C Ar), 143.6 (C Ar), 142.6 (C Ar), 133.7 (C Ar), 132.7 (C Ar), 131.6 (C Ar), 131.3 (C Ar), 129.1 (2 × C Ar), 128.8 (2 × C Ar), 127.9 (2 × C Ar), 127.4 (2 × C Ar), 127.0 (C Ar), 126.6 (C Ar), 124.1 (C Ar), 60.4 (C2), 53.7 (C6), 49.0 (C3), 46.6 (C5), 28.3 (C4), 19.3 (3 × CH(**C**H$_3$)$_2$), 19.0 (3 × CH(**C**H$_3$)$_2$), 11.6 (3 × **C**H(CH$_3$)$_2$), 7.6 (C7). **NOESY- 2D (500 MHz, CDCl$_3$)**: between C2-H and C3-H, between C6-H and C7-H$_A$, between C6-H and C7-H$_B$. **HRMS** (ESI): calculated for $C_{33}H_{44}N_2O_4SSiNa$ [M+Na]$^+$ requires *m/z* 615.2689, found *m/z* 615.2678. **IR** (film) ν_{max}: 2980, 2889, 1544, 1371, 1163, 1127 cm^{-1}.

Data for **3aa-B**: **R$_f$** 0.50 (50% EtOAc – pentane). **^{1}H NMR (500 MHz, CDCl$_3$) δ** 8.09 (1H, dd, *J* = 7.8, 1.4 Hz, Ar), 7.65 – 7.77 (2H, m, Ar), 7.25 – 7.30 (3H, m, Ar), 7.21 – 7.24 (2H, m, Ar), 7.15 – 7.20 (1H, m, Ar), 7.05 – 7.14 (3H, m, Ar), 6.92 – 6.96 (2H, m, Ar), 3.97 (1H, dd, *J* = 12.1, 2.8 Hz, C2-H), 3.63 – 3.69 (1H, m, C5-H$_A$), 3.62 (1H, d, *J* = 11.8 Hz, C6-H), 3.51 (td, *J* = 9.6, 1.9 Hz, C5-H$_B$), 2.71 (1H, dd, *J* = 11.9, 6.2 Hz, C3-H), 2.16 – 2.27 (1H, m, C4-H$_A$), 1.77 – 1.85 (1H, m, C4-H$_B$), 1.06 (1H, dd, *J* = 15.0, 2.8 Hz, C7-H$_A$), 0.96 – 1.03 (1H, m, C7-H$_B$), 0.94 (9H, d, *J* = 7.4 Hz, 3 × CH(C**H$_3$**)$_2$), 0.82 (9H, d, *J* = 7.4 Hz, 3 × CH(C**H$_3$**)$_2$), 0.64 (3H, hept, *J* = 7.5 Hz, 3 × C**H**(CH$_3$)$_2$). **^{13}C NMR (125 MHz, CDCl$_3$) δ** 148.4 (C Ar), 143.2 (C Ar), 143.0 (C Ar), 134.5 (C Ar), 133.4 (C Ar), 131.7 (C Ar), 131.5 (C Ar), 128.9 (2 × C Ar), 128.7 (2 × C Ar), 128.6 (2 × C Ar), 128.4 (2 × C Ar), 126.7 (C Ar), 126.7 (C Ar), 124.3 (C Ar), 61.6 (C2), 53.0 (C6), 49.7 (C3), 45.6 (C5), 27.2 (C4), 19.2 (3 × CH(**C**H$_3$)$_2$), 18.9

(3 × CH(**C**H$_3$)$_2$), 16.7 (C7), 11.5 (3 × **C**H(CH$_3$)$_2$). **NOESY- 2D (500 MHz, CDCl$_3$)**: between C2-H and C6-H. **HRMS** (ESI): calculated for $C_{33}H_{44}N_2O_4SSiNa$ [M+Na]$^+$ requires *m/z* 615.2689, found *m/z* 615.2677. **IR** (film) ν_{max}: 2980, 2889, 1544, 1371, 1163, 1127 cm^{-1}.

(3ab): (±)-(2*S*,3*R*)-3-(4-Methoxybenzyl)-1-[(2-nitrophenyl)sulfonyl]-2-[(triisopropylsilyl)methyl]-pyrrolidine and (±)-(2*S*,3*S*)-3-(4-methoxybenzyl)-1-[(2-nitrophenyl)sulfonyl]-2-[(triisopropylsilyl)methyl]pyrrolidine

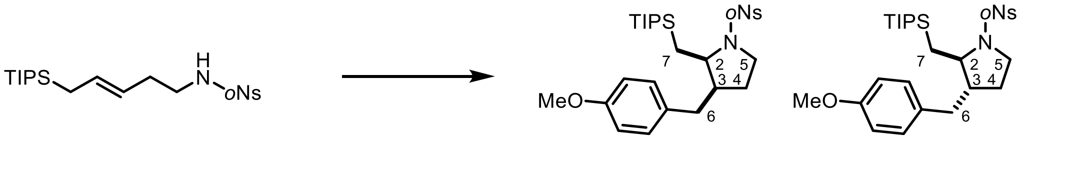

A **B**

1m
E:Z
4:1

3ab
d.r. (C2-C3) 4:1

Prepared according to **General Procedure K** using **1m** (27 mg, 0.063 mmol) and 4-methoxybenzyl alcohol (9.0 mg, 0.063 mmol) at 0 °C for 16 h. Flash column chromatography (gradient elution 20% → 35% EtOAc – pentane) afforded **3ab** as a separable 4:1 mixture of diastereomers as a colourless oil (34.5 mg, 99%).

Data for **3ab-A**: **R$_f$** 0.50 (60% EtOAc – pentane). **^{1}H NMR (500 MHz, CDCl$_3$) δ** 7.99 (1H, dd, *J* = 7.7, 1.6 Hz, Ar), 7.62 – 7.71 (2H, m, Ar), 7.60 (1H, dd, *J* = 7.6, 1.6 Hz, Ar), 6.95 (2H, d, *J* = 8.7 Hz, Ar), 6.80 (2H, d, *J* = 8.7 Hz, Ar), 4.24 (1H, dt, *J* = 8.6, 5.9 Hz, C2-H), 3.78 (OMe), 3.68 (1H, ddd, *J* = 11.7, 9.0, 5.9 Hz, C5-H$_A$), 3.47 (1H, ddd, *J* = 11.7, 8.6, 5.8 Hz, C5-H$_B$), 2.65 (1H, dd, *J* = 14.0, 6.2 Hz, C6-H$_A$), 2.46 (1H, dd, *J* = 14.0, 6.9 Hz, C6-H$_B$), 2.02 – 2.10 (1H, m, C3-H), 1.75 – 1.84 (1H, m, C4-H$_A$), 1.69 (1H, dtd, *J* = 12.7, 8.6, 5.8 Hz, C4-H$_B$), 1.17 (3H, hept, *J* = 7.5 Hz, 3 × C**H**(CH$_3$)$_2$), 1.05 (9H, d, *J* = 7.4 Hz, 3 × CH(C**H$_3$**)$_2$), 1.02 (9H, d, *J* = 7.4 Hz, 3 × CH(C**H$_3$**)$_2$), 0.87 (1H, dd, *J* = 14.8, 8.6 Hz, C7-H$_A$), 0.75 (1H, dd, *J* = 14.8, 5.9 Hz, C7-H$_B$). **^{13}C NMR (125 MHz, CDCl$_3$) δ** 158.2 (C Ar), 148.5 (C Ar), 133.6 (C Ar), 133.5 (C Ar), 131.9 (C Ar), 131.4 (C Ar), 131.1 (C Ar), 129.6 (2 × C Ar), 124.1 (C Ar), 114.1 (2 × C Ar), 62.0 (C2), 55.4 (OMe), 46.5 (C5), 45.5 (C3), 34.2 (C6), 28.1 (C4), 19.2 (3 × CH(**C**H$_3$)$_2$), 19.1 (3 × CH(**C**H$_3$)$_2$), 11.7 (3 × C**H**(CH$_3$)$_2$), 9.2 (CC-7). **NOESY- 2D (500 MHz, CDCl$_3$)**: between C2-H and C6-H, between C3-H$_A$ and C7-H$_B$, between C6-H$_B$ and C7-H$_B$. **HRMS** (ESI): calculated for $C_{28}H_{43}N_2O_5SSi$ [M+H]$^+$ requires *m/z* 547.2662, found *m/z* 547.2654. **IR** (film) ν_{max}: 2980, 2866, 1544, 1545, 1512, 1372 cm^{-1}.

Data for **3ab-B**: **R$_f$** 0.45 (60% EtOAc – pentane). **^{1}H NMR (500 MHz, CDCl$_3$) δ** 8.07-8.11 (1H, m, Ar), 7.66 – 7.71 (2H, m, Ar), 7.60 – 7.64 (1H, m, Ar), 6.88 (2H, d, *J* = 8.7 Hz, Ar), 6.78 (2H, d, *J* = 8.7 Hz, Ar), 4.05 (1H, dd, *J* = 12.7, 2.5 Hz, C2-H), 3.77 (OMe), 3.53 – 3.59 (2H, m, C5-H$_2$), 2.45 – 2.51 (1H, m, C6-H$_A$), 2.06 – 2.17 (3H, m, C3-H + C4-H$_A$ + C6-H$_B$), 1.69 – 1.76 (1H, m, C4-H$_B$), 1.04 – 1.13 (2H, m, C7-H$_2$), 0.99 (9H, d, *J* = 7.4 Hz, 3 × CH(C**H$_3$**)$_2$), 0.97 (9H, d, *J* = 7.4 Hz,

3 × CH(C**H$_3$**)$_2$), 0.86 (3H, hept, J = 7.5 Hz, 3 × C**H**(CH$_3$)$_2$). **^{13}C NMR (125 MHz, CDCl$_3$) δ** 158.3 (C Ar), 148.4 (C Ar), 133.4 (C Ar), 133.4 (C Ar), 131.7 (C Ar),131.4 (C Ar), 131.0 (C Ar), 130.1 (2 × C Ar), 124.2 (C Ar), 114.0 (2 × C Ar), 63.8 (C2), 55.4 (OMe), 46.8 (C3), 45.7 (C5), 37.8 (C6), 27.6 (C4), 19.0 (3 × CH(**C**H$_3$)$_2$), 18.9 (3 × CH(**C**H$_3$)$_2$), 18.0 (C7), 11.4 (3 × **C**H(CH$_3$)$_2$). **HRMS** (ESI): calculated for $C_{28}H_{43}N_2O_5SSi$ $[M+H]^+$ requires *m/z* 547.2662, found *m/z* 547.2654. **IR** (film) ν_{max}: 2980, 2866, 1544, 1545, 1512, 1372 cm^{-1}.

(3ac): (±)-(2*S*,3*S*)-3-Benzhydryl-2-[(*tert*-butyldiphenylsilyl)methyl]-1-[(2-nitrophenyl)sulfonyl]-pyrrolidine and (±)-(2*S*,3*R*)-3-Benzhydryl-2-[(*tert*-butyldiphenylsilyl)methyl]-1-[(2-nitrophenyl)-sulfonyl]pyrrolidine

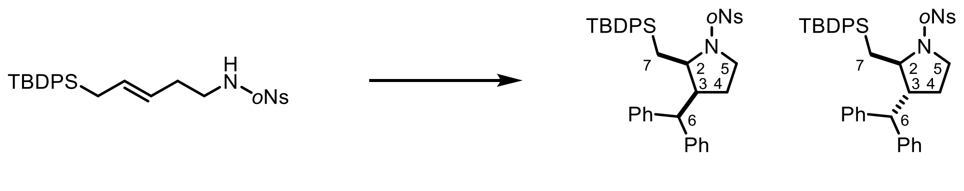

1n
E:Z
16:1

A **3ac** B
d.r. (C2-C3) 5:1

Prepared according to **General Procedure K** using **1n** (114 mg, 0.225 mmol) and benzhydrol (42 mg, 0.225 mmol) at 0 °C for 16 h. Flash column chromatography (gradient elution 6% → 10% EtOAc – pentane) afforded **3ac** as a separable 5:1 mixture of diastereomers as a colourless oil (150 mg, 99%).

Data for **3ac-A**: **R$_f$** 0.45 (30% EtOAc – pentane). **^{1}H NMR (500 MHz, CDCl$_3$) δ** 7.51 – 7.60 (3H, m, Ar), 7.45 – 7.48 (1H, m, Ar), 7.15 – 7.40 (17H, m, Ar), 6.94 – 6.98 (2H, m, Ar), 6.65 – 6.68 (1H, m, Ar), 4.11 (1H, ddd, J = 12.2, 5.9, 2.0 Hz, C2-H), 3.86 (1H, d, J = 12.0 Hz, C6-H), 3.81 – 3.89 (1H, m, C5-H$_A$), 3.52 (1H, ddd, J = 13.0, 9.4, 3.8 Hz, C5-H$_B$), 2.65 – 2.76 (1H, m, C3-H), 1.78 – 1.93 (1H, m, C4-H$_2$), 1.45 (1H, dd, J = 15.1, 12.2 Hz, C7-H$_A$), 1.34 (1H, dd, J = 15.2, 2.1 Hz, C7-H$_B$). 0.83 (9H, s, 3 × C(C**H$_3$**)$_3$). **^{13}C NMR (125 MHz, CDCl$_3$) δ** 147.2 (C Ar), 143.4 (C Ar), 142.9 (C Ar), 136.03 (2 × C Ar), 136.02 (C Ar), 135.9 (2 × C Ar), 133.5 (C Ar), 132.9 (C Ar), 132.7 (C Ar), 131.9 (C Ar), 131.3 (C Ar), 129.3 (2 × C Ar), 129.0 (C Ar), 128.9 (2 × C Ar), 128.7 (C Ar), 128.0 (2 × C Ar), 127.8 (2 × C Ar), 127.4 (2 × C Ar), 127.3 (2 × C Ar), 127.0 (C Ar), 126.8 (C Ar), 123.9 (C Ar), 59.5 (C2), 53.7 (C6), 49.5 (C3), 46.9 (C5), 28.3 (C4), 27.5 (3 × C(**C**H$_3$)$_3$), 18.6 (**C**(CH$_3$)$_3$), 7.0 (C7). **NOESY-2D (500 MHz, CDCl$_3$)**: between C2-H and C3-H, between C6-H and C7-H$_A$, between C6-H and C7-H$_B$. **HRMS** (ESI): calculated for $C_{40}H_{42}N_2O_4SSiNa$ $[M+Na]^+$ requires *m/z* 697.2532, found *m/z* 697.2521. **IR** (film) ν_{max}: 2980, 2360, 1543, 1372, 1164, 1126 cm^{-1}.

Data for **3ac-B**: **R$_f$** 0.50 (30% EtOAc – pentane). **^{1}H NMR (500 MHz, CDCl$_3$) δ** 7.11 – 7.15 (2H, m, Ar), 7.61 – 7.67 (2H, m, Ar), 7.48 – 7.56 (3H, m, Ar), 7.35 – 7.45 (4H, m, Ar), 7.22 – 7.31 (3H, m, Ar), 7.07 – 7.13 (2H, m, Ar), 7.02 – 7.07 (1H, m, Ar), 6.80 – 6.86 (1H, m, Ar), 6.74 – 6.79 (2H, m, Ar), 6.58 – 6.64 (2H, m, Ar), 6.01 – 6.06 (2H, m, Ar), 3.71 – 3.74 (1H, m, C2-H), 3.67 – 3.71 (1H, m,

C5-H_A), 3.58 (1H, td, *J* = 9.6, 1.6 Hz, C5-H_B), 3.19 (1H, d, *J* = 12.0 Hz, C6-H), 2.64 (1H, dd, *J* = 12.1, 6.0 Hz, C3-H), 2.07 – 2.19 (1H, m, C4-H_A), 1.87 (1H, dd, *J* = 15.1, 2.2 Hz, C7-H_A), 1.61 (1H, dd, *J* = 15.1, 11.9 Hz, C7-H_B), 1.49 – 1.56 (1H, m, C4-H_B), 0.93 (9H, s, 3 × C(C**H**$_3$)$_3$). **^{13}C NMR (125 MHz, CDCl$_3$) δ** 148.1 (C Ar), 143.5 (C Ar), 142.5 (C Ar), 136.4 (C Ar), 136.4 (C Ar), 134.9 (C Ar), 133.6 (C Ar), 133.0 (C Ar), 132.8 (C Ar), 131.8 (C Ar), 131.6 (C Ar), 129.7 (C Ar), 129.1 (C Ar), 128.6 (2 × C Ar), 128.3 (2 × C Ar), 128.0 (2 × C Ar), 127.9 (2 × C Ar), 127.7 (2 × C Ar), 127.7 (2 × C Ar), 126.4 (C Ar), 126.0 (C Ar), 123.8 (C Ar), 60.7 (C2), 52.8 (C6), 48.3 (C3), 45.5 (C5), 27.6 (3 × C(**C**H$_3$)$_3$), 26.6 (C4), 18.4 (C7), 18.2 (3 × **C**(CH$_3$)$_3$). **NOESY- 2D (500 MHz, CDCl$_3$)**: between C2-H and C6-H. **HRMS** (ESI): calculated for $C_{40}H_{42}N_2O_4SSiNa$ [M+Na]$^+$ requires *m/z* 697.2532, found *m/z* 697.2521. **IR** (film) ν_{max}: 2980, 2360, 1543, 1372, 1164, 1126 cm^{-1}.

((–)-3ad): (–)-(2*R*,3*R*,4*S*)-3-Benzhydryl-2-(4-methoxyphenyl)-1-[(2-nitrophenyl)sulfonyl]-4-phenylpyrrolidine and (–)-(2*S*,3*S*,4*S*)-3-Benzhydryl-2-(4-methoxyphenyl)-1-[(2-nitrophenyl)sulfonyl]-4-phenylpyrrolidine

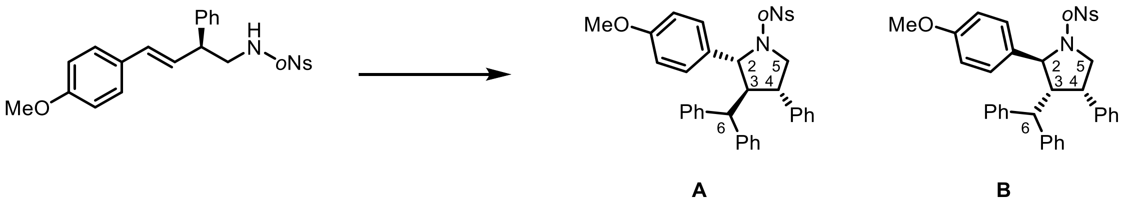

(-)-1o

(-)-3ad
d.r. (C3-C4) 7:1

Prepared according to **General Procedure K** using **(–)-1o** (9.5 mg, 0.022 mmol) and benzhydrol (4.0 mg, 0.022 mmol) at 0 °C for 48 h. Flash column chromatography (gradient elution 15% → 25% EtOAc – pentane) afforded **(–)-3ad** as an inseparable 7:1 mixture of diastereomers as a colourless oil (30 mg, 99%, 99% *ee*).

Data for **(–)-3ad-A** (from the mixture): **^{1}H NMR (500 MHz, CDCl$_3$) δ** 7.51 (1H, dd, *J* = 7.9, 1.4 Hz, Ar), 7.43 – 7.48 (1H, m, Ar), 7.00 – 7.24 (8H, m, Ar), 6.86 – 7.00 (6H, m, Ar), 6.72 – 6.85 (3H, m, Ar), 6.62 (2H, d, *J* = 8.6 Hz, Ar), 6.45 (2H, d, *J* = 8.6 Hz, Ar), 4.85 (1H, d, *J* = 5.9 Hz, C2-H), 4.35 (1H, dd, *J* = 10.8, 8.7 Hz, C5-H_A), 4.01 (1H, d, *J* = 10.4 Hz, C6-H), 3.86 – 3.93 (1H, m, C5-H_B), 3.70 (OMe), 3.43 – 3.51 (1H, m, C3-H), 3.26 (1H, q, *J* = 8.7 Hz, C4-H). **^{13}C NMR (125 MHz, CDCl$_3$) δ** 158.7 (C Ar), 147.6 (C Ar), 142.5 (C Ar), 141.6 (C Ar), 140.6 (C Ar), 134.0 (C Ar), 133.8 (C Ar), 132.6 (C Ar), 131.1 (C Ar), 131.1 (C Ar), 128.7 (2 × C Ar), 128.6 (2 × C Ar), 128.5 (2 × C Ar), 128.5 (2 × C Ar), 128.4 (2 × C Ar), 128.2 (2 × C Ar), 128.0 (2 × C Ar), 126.8 (C Ar), 126.5 (C Ar), 126.4 (C Ar), 123.6 (C Ar), 113.6 (2 × C Ar), 68.7 (C2), 61.0 (C3), 58.8 (C6), 57.9 (C5), 55.4 (OMe), 51.0 (C4). **NOESY- 2D (500 MHz, CDCl$_3$)**: between C2-H and C4-H, between C2-H and C6-H, between C4-H and C6-H.

Partial data for **(–)-3ad-B** (from the mixture): **^{1}H NMR (500 MHz, $CDCl_3$) δ** 7.43 – 7.48 (1H, m, Ar), 7.32 – 7.37 (1H, m, Ar), 7.00 – 7.24 (8H, m, Ar), 6.86 – 7.00 (6H, m, Ar), 6.72 – 6.85 (3H, m, Ar), 6.54 (2H, d, J = 8.6 Hz, Ar), 6.18 (2H, d, J = 8.6 Hz, Ar), 4.84 (1H, d, J = 12.7 Hz, C2-H), 4.37 – 4.40 (1H, m, C5-H_A), 4.17 – 4.22 (1H, m, C5-H_B), 3.74 – 3.79 (1H, m, C3-H), 3.62 (OMe), 3.43 – 3.51 (2H, m, C4-H + C6-H). **^{13}C NMR (125 MHz, $CDCl_3$) δ** 158.3 (C Ar), 146.6 (C Ar), 142.8 (C Ar), 142.0 (C Ar), 140.2 (C Ar), 134.0 (C Ar), 133.8 (C Ar), 132.0 (C Ar), 131.1 (C Ar), 130.8 (C Ar), 129.0 (2 × C Ar), 128.6 (2 × C Ar), 128.5 (4 × C Ar), 128.3 (2 × C Ar), 128.1 (2 × C Ar), 127.9 (2 × C Ar), 127.2 (C Ar), 126.6 (C Ar), 126.4 (C Ar), 123.4 (C Ar), 113.2 (2 × C Ar), 67.4 (C2), 57.2 (C3), 56.4 (C5), 55.4 (OMe), 53.5 (C6), 46.3 (C4). *The stereochemistry of the minor isomer was not proven.*

Data for **(–)-3ad**: **R_f** 0.40 (30% EtOAc – pentane). **$[\alpha]_D^{25}$** (of the mixture) = –0.159 (c = 10.0, $CHCl_3$). **HRMS** (ESI): calculated for $C_{36}H_{33}N_2O_5S$ $[M+H]^+$ requires m/z 605.2110, found m/z 605.2100. **IR** (film) ν_{max}: 2980, 2889, 2360, 1542, 1372, 1162 cm^{-1}. **Chiral HPLC** (Chiralpak IC with guard, 20% IPA, 80% hexane, 1.0 mL/min, 25 ˚C, λ = 210 nm, 10 µL injection).

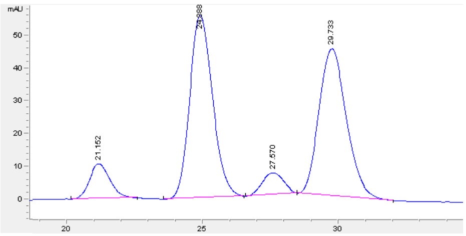

#	Time	Type	Area	Height	Width	Area%	Symmetry
1	3.313	BV	38.8	7.4	0.0795	0.518	1.625
2	3.424	VB	144	26.9	0.0784	1.924	0.742
3	21.152	BB	556.8	10.6	0.7797	7.437	0.797
4	24.888	BB	3250.6	55.5	0.8991	43.417	0.821
5	27.57	BB	361.3	6.6	0.7638	4.826	1.052
6	29.733	BB	3135.2	44.7	1.08	41.877	0.826

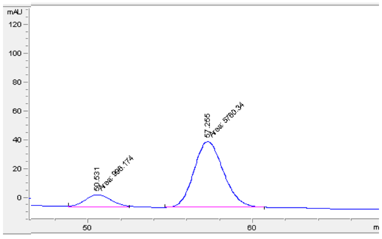

#	Time	Type	Area	Height	Width	Area%	Symmetry
1	50.531	MM	996.2	8.7	1.9054	14.744	0.908
2	57.255	MM	5760.3	45.7	2.0997	85.256	0.864

Racemic **(±)-3ad** was made *via* the same procedure using **(±)-1o** (99%, 7:1 d.r.).

((–)-3ae): (–)-(2*R*,3*R*,4*S*)-3-(4-Methoxybenzyl)-2-(4-methoxyphenyl)-1-[(2-nitrophenyl)sulfonyl]-4-phenylpyrrolidine and (–)-(2*S*,3*S*,4*S*)-3-(4-Methoxybenzyl)-2-(4-methoxyphenyl)-1-[(2-nitrophenyl)sulfonyl]-4-phenylpyrrolidine

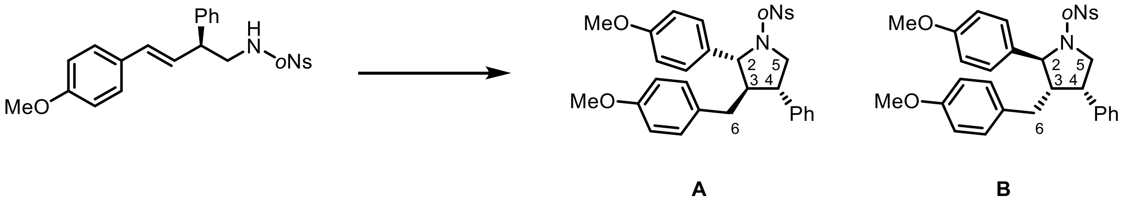

(-)-1o

(-)-3ae
d.r. (C3-C4) 4:1

Prepared according to **General Procedure K** using **(–)-1o** (38.5 mg, 0.088 mmol) and 4-methoxybenzyl alcohol (12 mg, 0.088 mmol) at 0 °C for 48 h. Flash column chromatography (gradient elution 10% → 30% EtOAc – pentane) afforded **(–)-3ae** as an inseparable 4:1 mixture of diastereomers as a colourless oil (39.5 mg, 80%, 99% *ee*).

Data for **(–)-3ae-A** (from the mixture): **^{1}H NMR (500 MHz, $CDCl_3$) δ** 7.49 (1H, dd, *J* = 8.0, 1.3 Hz, Ar), 7.40 – 7.45 (1H, m, Ar), 7.30 – 7.36 (2H, m, Ar), 7.24 – 7.30 (3H, m, Ar), 7.08 – 7.15 (2H, m, Ar), 7.06 (2H, d, *J* = 8.7 Hz, Ar), 6.78 (2H, d, *J* = 8.7 Hz, Ar), 6.64 (2H, d, *J* = 8.7 Hz, Ar), 6.56 (2H, d, *J* = 8.6 Hz, Ar), 4.62 (1H, d, *J* = 9.6 Hz, C2-H), 4.32 (1H, dd, *J* = 10.7, 7.8 Hz, C5-H_A), 3.74 (3H, s, OMe), 3.73 (3H, s, OMe), 3.67 – 3.71 (1H, m, C5-H_B), 3.12 (1H, td, *J* = 11.4, 7.8 Hz, C4-H), 2.79 (1H, ddt, *J* = 11.4, 9.6, 5.5 Hz, C3-H), 2.59 (1H, d, *J* = 5.5 Hz, C6-H_2). **^{13}C NMR (125 MHz, $CDCl_3$) δ** 159.2 (C Ar), 158.2 (C Ar), 147.3 (C Ar), 138.2 (C Ar), 134.5 (C Ar), 132.4 (C Ar), 131.6 (C Ar), 130.9 (2 × C Ar), 130.8 (2 × C Ar), 129.5 (2 × C Ar), 129.5 (C Ar), 129.0 (C Ar), 128.2 (2 × C Ar), 127.5 (C Ar), 123.6 (C Ar), 113.7 (2 × C Ar), 113.7 (2 × C Ar), 67.7 (C2), 57.0 (C5), 56.3 (C3), 55.5 (OMe), 55.4 (OMe), 48.5 (C4), 33.8 (C6). **NOESY- 2D (500 MHz, $CDCl_3$)**: between C2-H and C4-H, between C2-H and C6-H_2, between C4-H and C6-H_2.

Partial data for **(–)-3ae-B** (from the mixture): **^{1}H NMR (500 MHz, $CDCl_3$) δ** 7.45 – 7.49 (1H, m, Ar), 7.40 – 7.45 (1H, m, Ar), 7.30 – 7.36 (2H, m, Ar), 7.24 – 7.30 (3H, m, Ar), 7.17 – 7.21 (2H, m, Ar), 6.95 (2H, d, *J* = 8.7 Hz, Ar), 6.89 (2H, d, *J* = 8.7 Hz, Ar), 6.69 – 6.73 (2H, m, Ar), 6.52 (2H, d, *J* = 8.7 Hz, Ar), 4.54 (1H, d, *J* = 9.8 Hz, C2-H), 4.27 (1H, dd, *J* = 10.6, 7.8 Hz, C5-H_A), 3.82 (3H, s, OMe), 3.79 (3H, s, OMe), 3.66 – 3.70 (1H, m, C5-H_B), 3.04 (1H, td, *J* = 11.4, 7.8 Hz, C4-H), 2.68 – 2.74 (1H, m, C3-H), 2.44 – 2.49 (2H, m, C6-H_2). **^{13}C NMR (125 MHz, $CDCl_3$) δ** 159.21 (C Ar), 158.0 (C Ar), 155.9 (C Ar), 143.6 (C Ar), 133.0 (C Ar), 132.3 (C Ar), 131.7 (C Ar), 130.9 (2 × C Ar), 130.3 (2 × C Ar), 130.0 (C Ar), 129.6 (2 × C Ar), 129.0 (2 × C Ar), 128.2 (2 × C Ar), 127.4 (C Ar), 123.5 (C Ar), 114.0 (2 × C Ar), 113.6 (2 × C Ar), 67.1 (C2), 57.0 (C5), 56.0 (C3), 55.6 (OMe), 55.4 (OMe), 47.8 (C4), 33.0 (C6). **NOESY- 2D (500 MHz, $CDCl_3$)**: between C2-H and C6-H_2,

Data for **(–)-3ae**: **R$_f$** 0.40 (30% EtOAc – pentane). **[α]$_D^{25}$** (of the mixture) = –3.15 (*c* = 10.0, $CHCl_3$). **HRMS** (ESI): calculated for $C_{31}H_{30}N_2O_6SNa$ $[M+Na]^+$ requires *m/z* 581.1722, found *m/z* 581.1715. **IR** (film) ν_{max}: 2921, 1711, 1611, 1542, 1511, 1464 cm^{-1}. **Chiral HPLC** (Chiralpak IA with guard, 5% IPA, 95% hexane, 1.0 mL/min, 25 ˚C, λ = 210 nm, 10 μL injection).

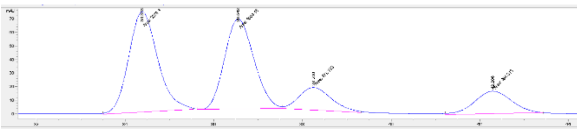

#	Time	Type	Area	Height	Width	Area%	Symmetry
1	34.378	MM	3376.3	73.8	0.7626	40.696	0.851
2	36.543	MM	3053.2	66.2	0.7688	36.801	0.842
3	38.25	MM	872.7	16.8	0.8642	10.519	0.76
4	42.286	MM	994.3	16.7	0.9905	11.985	0.954

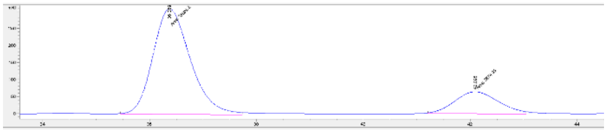

#	Time	Type	Area	Height	Width	Area%	Symmetry
1	36.369	MM	15262.2	310.8	0.8185	79.752	0.783
2	42.095	MM	3874.9	66.2	0.9756	20.248	0.864

Racemic **(±)-3ae** was made *via* the same procedure using **(±)-1o** (99%, 4:1 d.r.).

((–)-3af): (–)-(2*R*,3*R*,4*S*)-2-(4-Methoxyphenyl)-1-[(2-nitrophenyl)sulfonyl]-4-phenyl-3-[(1'*S*)-3-phenylcyclopent-2-en-1-yl]pyrrolidine and (–)-(2*S*,3*S*,4*S*)-2-(4-Methoxyphenyl)-1-[(2-nitrophenyl)sulfonyl]-4-phenyl-3-[(1'*R*)-3-phenylcyclopent-2-en-1-yl]pyrrolidine

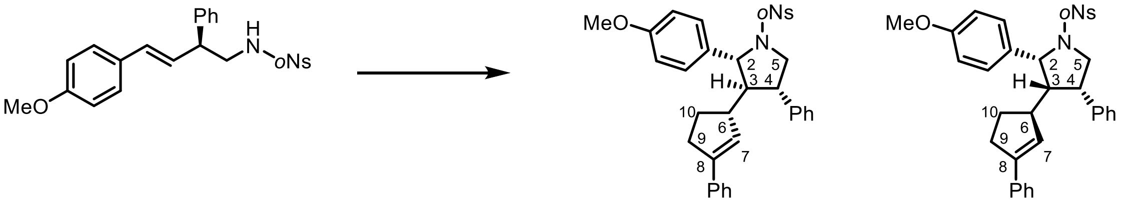

Prepared according to **General Procedure K** using **(–)-1o** (9.0 mg, 0.020 mmol) and **2q** (3.0 mg, 0.020 mmol) at 0 °C for 48 h. Flash column chromatography (gradient elution 15% → 25% EtOAc – pentane) afforded **(–)-3af** as an inseparable 12:1 mixture of diastereomers as a colourless oil (9.5 mg, 83%, 99% *ee*).

Data for **(–)-3af-A** (from the mixture): **^{1}H NMR (500 MHz, $CDCl_3$) δ** 7.48 – 7.52 (1H, m, Ar), 7.39 – 7.46 (2H, m, Ar), 7.06 – 7.39 (13H, m, Ar), 6.56 (2H, d, *J* = 8.7 Hz, Ar), 5.55 (1H, q, *J* = 2.3 Hz, C7-H), 4.80 (1H, d, *J* = 9.1 Hz, C2-H), 4.39 (1H, dd, *J* = 10.7, 7.6 Hz, C5-H_A), 3.82 – 3.90 (1H, m, C5-H_B), 3.73 (OMe), 3.26 (1H, td, *J* = 11.4, 7.5 Hz, C4-H), 2.97 – 3.04 (1H, m, C6-H), 2.81 (1H, ddd, *J* = 11.4, 9.1, 4.4 Hz, C3-H), 2.46 – 2.62 (2H, m, C10-H_2), 2.02 (1H, dtd, *J* = 13.3, 9.0, 4.4 Hz, C9-H_A), 1.54 – 1.66 (1H, m, C9-H_B). **^{13}C NMR (125 MHz, $CDCl_3$) δ** 159.2 (C Ar), 147.4 (C Ar), 142.6 (C Ar), 139.0 (C Ar), 136.2 (C Ar), 134.5 (C Ar), 133.1 (C8), 132.4 (C Ar), 131.0 (C Ar), 130.9 (C Ar), 129.7 (2 × C Ar), 129.0 (2 × C Ar), 128.3 (2 × C Ar), 128.1 (2 × C Ar), 127.7 (C Ar), 127.6 (C7), 127.3 (C Ar), 125.8 (2 × C Ar), 123.6 (C Ar), 113.7 (2 × C Ar), 66.7 (C2), 60.2 (C3), 57.1 (C5), 55.5 (OMe), 48.3 (C4), 45.9 (C3), 32.7 (C10), 26.0 (C9). **NOESY- 2D (500 MHz, $CDCl_3$)**: between C2-H and C4-H,

between C2-H and C6-H, between C4-H and C7-H, between C4-H and C9-H_A, between C4-H and C6-H, between C4-H and C7-H, between C4-H and C9-H_B.

(–)-3af-B *could not be assigned due to low intensity of signals in* ^{1}*H and* ^{13}*C spectra; the stereochemistry is not proven.*

Data for **(–)-3af**: **R_f** 0.50 (35% EtOAc – pentane). **$[\alpha]^{25}_{D}$** (of the mixture) = –3.12 (c = 10.0, $CHCl_3$). **HRMS** (ESI): calculated for $C_{34}H_{32}N_2O_5SNa$ $[M+Na]^+$ requires m/z 603.1930, found m/z 603.1917. **IR** (film) ν_{max}: 2980, 2889, 1738, 1542, 1372, 1250 cm^{-1}.

Chiral HPLC (Chiralpak IC with guard, 10% IPA, 90% hexane, 1.0 mL/min, 25 ˚C, λ = 210 nm, 10 µL injection).

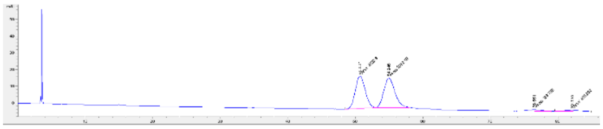

#	Time	Type	Area	Height	Width	Area%	Symmetry
1	50.661	MM	2023.8	19.5	1.7296	45.165	0.877
2	54.946	MM	2072.6	17.8	1.9456	46.254	0.8
3	76.551	MM	183.7	9.8E-1	3.1093	4.100	1.026
4	82.135	MM	200.8	9.8E-1	3.4311	4.481	0.85

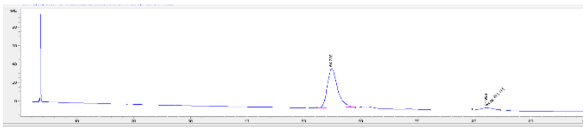

#	Time	Type	Area	Height	Width	Area%	Symmetry
1	54.738	BB	5363	42.3	1.8075	91.924	0.711
2	81.999	MM	471.2	2.8	2.8347	8.076	0.833

Racemic **(±)-3af** was made *via* the same procedure using **(±)-1o** (99%, 12:1 d.r.).

(5-Ts): (±)-(2*S*,3*R*)-3-Benzhydryl-2-(4-methoxyphenyl)-1-tosylpiperidine and (±)-(2*S*,3*S*)-3-Benzhydryl-2-(4-methoxyphenyl)-1-tosylpiperidine

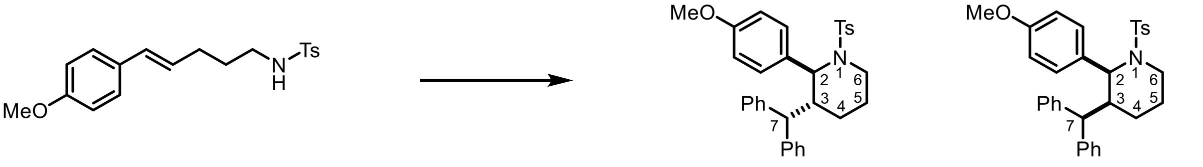

4-Ts **A** **5-Ts** **B**
dr (C2-C3) 5:1

Prepared according to **General Procedure K** using **4-Ts** (14 mg, 0.040 mmol) and benzhydrol (7.5 mg, 0.040 mmol). Flash column chromatography (gradient elution 0% → 25% EtOAc – pentane) afforded **5-Ts** as an inseparable 5:1 mixture of diastereomers as a colourless oil (20 mg, 95%).

Data for **5-Ts-A** (from the mixture): **^{1}H NMR (500 MHz, $CDCl_3$) δ** 7.50 (2H, d, J = 8.3 Hz, Ar), 7.45 – 7.39 (2H, m, Ar), 7.33 (2H, t, J = 7.7 Hz, Ar), 7.30 – 7.17 (8H, m, Ar), 7.04 – 6.95 (2H, m, Ar), 6.73 (2H, d, J = 8.7 Hz, Ar), 5.07 (1H, *br.* s, C2-H), 4.24 (1H, d, J = 11.9 Hz, C7-H), 3.93 (1H, dd, J = 13.2, 5.0 Hz, C6-H_A), 3.77 (3H, s, OMe), 3.15 (1H, td, J = 13.1, 3.4 Hz, C6-H_B), 3.02 – 2.96 (1H, m, C3-H), 2.41 (3H, s, TsMe), 1.93 – 1.69 (1H, m, C5-H_A), 1.52 (1H, tt, J = 13.8, 4.0 Hz, C4-H_A), 1.42 – 1.34 (1H, m, C4-H_B), 1.34 – 1.28 (1H, m, C5-H_B). **^{13}C NMR (126 MHz, $CDCl_3$) δ** 158.4 (C Ar), 143.6 (C Ar), 142.8 (C Ar), 138.0 (C Ar), 132.7 (C Ar), 131.6 (C Ar), 129.4 (2 × C Ar), 128.9 (2 × C Ar), 128.8 (2 × C Ar), 128.3 (2 × C Ar), 128.2 (2 × C Ar), 128.2 (2 × C Ar), 127.3 (2 × C Ar), 126.8

(C Ar), 126.4 (C Ar), 113.8 (2 × C Ar), 57.4 (C1), 55.4 (OMe), 52.1 (C7), 42.2 (C6), 41.9 (C3), 21.6 (TsMe), 20.4 (C4), 19.7 (C5).

Partial data for **5-Ts-B** (from the mixture): **^{1}H NMR (500 MHz, CDCl$_3$) δ** 7.08 – 7.04 (2H, m, Ar), 6.78 (2H, d, *J* = 8.7 Hz, Ar), 6.61 (2H, d, *J* = 8.7 Hz, Ar), 5.04 (1H, d, *J* = 5.4 Hz, C2-H), 3.85 – 3.80 (1H, m, C6-H$_A$), 3.80 (3H, s, OMe), 3.11 – 3.05 (1H, m, C6-H$_B$), 2.88 – 2.79 (1H, m, C3-H), 2.33 (3H, s, TsMe), 1.73 – 1.68 (1H, m, C5-H$_A$), 1.62 (1H, td, *J* = 13.2, 3.8 Hz, C4-H$_A$). **^{13}C NMR (126 MHz, CDCl$_3$) δ** 159.1 (C Ar), 143.5 (C Ar), 142.4 (C Ar), 142.0 (C Ar), 137.0 (C Ar), 129.4 (C Ar), 129.0 (2 × C Ar), 128.7 (2 × C Ar), 128.4 (2 × C Ar), 128.0 (C Ar), 127.3 (2 × C Ar), 126.7 (C Ar), 113.2 (2 × C Ar), 58.1 (C2), 55.3 (OMe), 55.2 (C7), 43.3 (C3), 41.1 (C6), 25.3 (C5), 23.4 (C4), 21.5 (TsMe).

Data for **5-Ts**: **R$_f$** 0.52 (20% EtOAc – pentane). **HRMS** (ESI): calculated for $C_{32}H_{33}O_3SNNa$ [M+Na]$^+$ requires m/z 534.2073, found m/z 534.2069. **IR** (film) ν_{max}: 3671, 3649, 3026, 2981, 2972, 2889, 2359, 2339, 1512, 1382 cm^{-1}.

(5-Ms): (±)-(2*S*,3*R*)-3-Benzhydryl-2-(4-methoxyphenyl)-1-(methylsulfonyl)piperidine and (±)-(2*S*,3*R*)-3-Benzhydryl-2-(4-methoxyphenyl)-1-(methylsulfonyl)piperidine

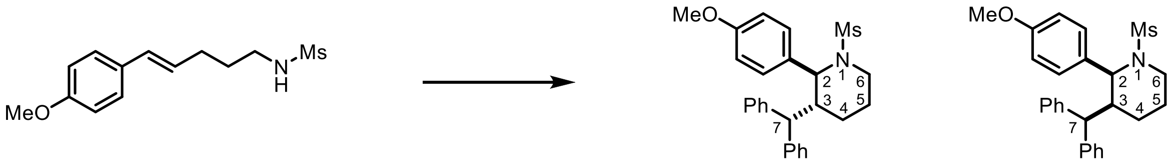

4-Ms **A** **5-Ms** **B**
dr (C2-C3) 2:1

Prepared according to **General Procedure K** using **4-Ms** (28 mg, 0.10 mmol) and benzhydrol (18 mg, 0.10 mmol). Flash column chromatography (gradient elution 0% → 25% EtOAc – pentane) afforded **5-Ms** as an inseparable 2:1 mixture of diastereomers as a colourless oil (28 mg, 64%).

Data for **5-Ms-A** (from the mixture): **^{1}H NMR (500 MHz, CDCl$_3$) δ** 7.49 – 7.44 (2H, d, *J* = 8.7 Hz, Ar), 7.39 – 7.14 (10H, m, Ar), 6.88 (2H, d, *J* = 8.7 Hz, 2H), 4.91 (1H, s, C2-H), 4.32 (1H, d, *J* = 11.9 Hz, C7-H), 3.87 (1H, dd, *J* = 13.1, 5.3 Hz, C6-H$_A$), 3.80 (3H, s, OMe), 3.28 (1H, td, *J* = 12.8, 3.4 Hz, C6-H$_B$), 2.99 – 2.94 (1H, m, C3-H), 2.62 (3H, s, SO$_2$Me), 1.98 – 1.91 (1H, m, C5-H$_A$), 1.72 – 1.65 (1H, m, C4-H$_A$), 1.60 – 1.53 (1H, m, C4-H$_B$), 1.50 – 1.41 (1H, m, C5-H$_B$). **^{13}C NMR (126 MHz, CDCl$_3$) δ** 158.8 (C Ar), 143.5 (C Ar), 143.3 (C Ar), 131.9 (C Ar), 128.9 (2 × C Ar), 128.8 (2 × C Ar), 128.5 (2 × C Ar), 128.3 (2 × C Ar), 128.1 (2 × C Ar), 126.9 (C Ar), 126.5 (C Ar), 114.2 (2 × C Ar), 57.3 (C2), 55.4 (OMe), 52.5 (C7), 42.0 (C6), 41.8 (C3), 39.5 (SO$_2$Me), 20.4 (C4), 19.9 (C5).

Partial data for **5-Ms-B** (from the mixture): **^{1}H NMR (500 MHz, CDCl$_3$) δ** 7.08 – 7.04 (2H, m, Ar), 6.97 (2H, d, *J* = 8.7 Hz, Ar), 6.78 (2H, d, *J* = 8.7 Hz, Ar), 4.99 (1H, d, *J* = 5.5 Hz, C2-H), 3.82 (3H, s, OMe), 3.83 – 3.79 (1H, m, C7-H), 3.74 – 3.68 (1H, m, C6-H$_A$), 3.16 (1H, td, *J* = 12.4, 3.0 Hz, C6-H$_B$),

3.02 – 2.99 (1H, m, C3-H), 2.06 (3H, s, OMe), 1.91 – 1.83 (1H, m, C5-H_A), 1.83 – 1.74 (2H, m, C4-H_A + C5-H_B), 1.63 – 1.59 (1H, m, C4-H_B). **^{13}C NMR (126 MHz, $CDCl_3$) δ** 159.4 (C Ar), 143.4 (C Ar), 141.8 (C Ar), 132.8 (C Ar), 129.0 (2 × C Ar), 128.7 (2 × C Ar), 128.5 (2 × C Ar), 128.3 (2 × C Ar), 128.0 (2 × C Ar), 126.7 (C Ar), 126.5 (C Ar), 113.7 (2 × C Ar), 58.3 (C2), 55.6 (OMe), 55.3 (C7), 43.5 (C6), 40.6, 37.3 (SO_2Me), 25.5 (C4), 23.8 (C5).

Data for **5-Ms**: **R_f** 0.32 (20% EtOAc – pentane). **HRMS** (ESI): calculated for $C_{26}H_{26}O_3SNNa$ [M+Na]$^+$ requires m/z 458.1760, found m/z 458.1756. **IR** (film) ν_{max}: 3026, 2935, 1609, 1512, 1451, 1333, 1251, 1151, 1031 cm^{-1}.

(5-*o*Ns): (±)-(2*S*,3*S*)-3-Benzhydryl-2-(4-methoxyphenyl)-1-((2-nitrophenyl)sulfonyl)piperidine and (±)-(2*S*,3*R*)-3-Benzhydryl-2-(4-methoxyphenyl)-1-((2-nitrophenyl)sulfonyl)piperidine

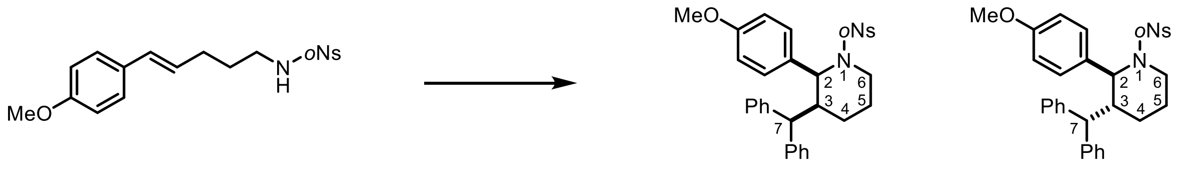

4-*o*Ns   **5-*o*Ns**
dr (C2-C3) 2:1

Prepared according to **General Procedure K** using **4-*o*Ns** (30 mg, 0.080 mmol) and benzhydrol (14 mg, 0.080 mmol). Flash column chromatography (gradient elution 0% → 25% EtOAc – pentane) afforded **5-*o*Ns** as a separable 2:1 mixture of diastereomers as a colourless oil (37 mg, 84%).

Data for **5-*o*Ns-A**: **R_f** 0.45 (20% EtOAc – pentane). **^{1}H NMR (500 MHz, $CDCl_3$) δ** 7.64 – 7.62 (1H, m, Ar), 7.58 – 7.54 (2H, m, Ar), 7.43 (1H, ddd, *J* = 7.9, 5.4, 3.4 Hz, Ar), 7.29 – 7.22 (3H, m, Ar), 7.20 – 7.16 (5H, m, Ar), 7.13 – 7.08 (3H, m, Ar), 6.89 – 6.85 (2H, m, Ar), 5.01 (1H, d, *J* = 5.5 Hz, C2-H), 3.79 (3H, s, OMe), 3.75 (1H, d, *J* = 2.4 Hz C6-H_A), 3.40 (1H, td, *J* = 13.1, 3.2 Hz, C6-H_B), 3.19 (1H, d, *J* = 11.9 Hz, C7-H), 2.94 (1H, app. qd, *J* = 7.0, 6.4, 4.4 Hz, C3-H), 1.89 – 1.86 (1H, m, C5-H_A), 1.73 – 1.69 (2H, m, C4-H_A + C5-H_B), 1.50 (1H, tt, *J* = 9.9, 2.7 Hz, C4-H_B). **^{13}C NMR (126 MHz, $CDCl_3$) δ** 159.2 (C Ar), 148.0 (C Ar), 143.3 (C Ar), 141.9 (C Ar), 133.8 (C Ar), 133.1 (C Ar), 131.4 (3 × C Ar), 131.0 (C Ar), 129.3 (C Ar), 128.8 (2 × C Ar), 128.7 (2 × C Ar), 128.4 (2 × C Ar), 128.0 (2 × C Ar), 126.7 (C Ar), 126.5 (C Ar), 124.3 (C Ar), 113.4 (2 × C Ar), 58.5 (C2), 55.3 (OMe), 55.3 (C7), 43.2 (C3), 41.4 (C6), 25.5 (C5), 23.5 (C4). **HRMS** (ESI): calculated for $C_{31}H_{30}O_5SN_2Na$ [M+Na]$^+$ requires m/z 565.1768, found m/z 565.1766. **IR** (film) ν_{max}: 2981, 2361, 2341, 1609, 1542, 1512, 1494, 1453, 1372, 1341 cm^{-1}.

Data for **5-*o*Ns-B**: **R_f** 0.49 (20% EtOAc – pentane). **^{1}H NMR (500 MHz, $CDCl_3$) δ** 7.62 (1H, dd, *J* = 8.0, 1.3 Hz, Ar), 7.54 (1H, td, *J* = 7.7, 1.4 Hz, Ar), 7.42 (1H, dd, *J* = 8.0, 1.3 Hz, Ar), 7.30 – 7.20 (11H, m, Ar), 7.19 – 7.16 (1H, m, Ar), 7.14 – 7.11 (3H, m, Ar), 6.77 – 6.75 (2H, m, Ar), 4.96 (1H, *br.* s, C2-H), 4.25 (1H, d, *J* = 11.8 Hz, C7-H), 4.20 (1H, dd, *J* = 13.6, 5.1 Hz, C6-H_A), 3.75 (3H, s, OMe),

3.48 (1H, td, J = 13.0, 3.3 Hz, C6-H$_B$), 3.03 (1H, *app.* d, J = 11.6 Hz, C3-H), 1.94 (1H, dtd, J = 14.5, 11.7, 10.5, 6.9 Hz, C4-H$_A$), 1.52 – 1.44 (1H, m, C5-H$_A$), 1.44 – 1.36 (2H, m, C4-H$_B$ + C5-H$_B$). **^{13}C NMR (126 MHz, CDCl$_3$) δ** 158.5 (C Ar), 143.5 (C Ar), 143.4 (C Ar), 134.3 (C Ar), 133.0 (C Ar), 131.9 (C Ar), 131.7 (2 × C Ar), 131.7 (C Ar), 128.9 (C Ar), 128.9 (2 × C Ar), 128.2 (2 × C Ar), 128.2 (2 × C Ar), 128.0 (2 × C Ar), 126.6 (C Ar), 126.5 (C Ar), 123.9 (C Ar), 113.9 (2 × C Ar), 58.1 (C2), 55.4 (OMe), 51.7 (C7), 43.6 (C6), 42.5 (C2), 20.5 (C5), 19.9 (C4). **HRMS** (ESI): calculated for $C_{31}H_{30}O_5SN_2Na$ [M+Na]$^+$ requires m/z 565.1768, found m/z 565.1764. **IR** (film) ν_{max}: 2981, 2361, 2341, 1609, 1542, 1512, 1494, 1453, 1372, 1341 cm^{-1}.

(5-*p*Ns): (±)-(2*S*,3*R*)-3-Benzhydryl-2-(4-methoxyphenyl)-1-((2-nitrophenyl)sulfonyl)piperidine and (±)-(2*S*,3*S*)-3-Benzhydryl-2-(4-methoxyphenyl)-1-((2-nitrophenyl)sulfonyl)piperidine

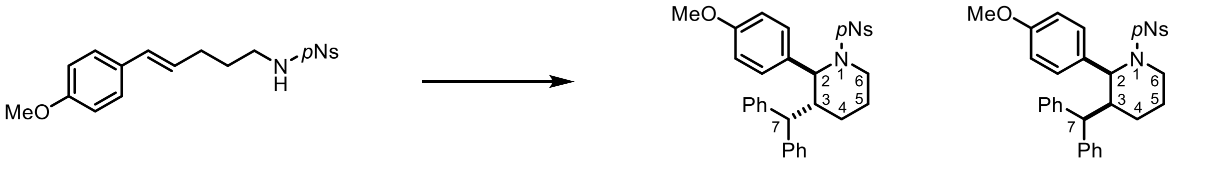

4-*p*Ns **A** **5-*p*Ns** **B**
dr (C2-C3) 3:1

Prepared according to **General Procedure K** using **4-*p*Ns** (15 mg, 0.040 mmol) and benzhydrol (7.5 mg, 0.040 mmol). Flash column chromatography (gradient elution 0% → 25% EtOAc – pentane) afforded **5-*p*Ns** as an inseparable 3:1 mixture of diastereomers as a colourless oil (19 mg, 88%).

Data for **5-*p*Ns-A** (from the mixture): **^{1}H NMR (500 MHz, CDCl$_3$) δ** 8.11 (2H, d, J = 8.9 Hz, Ar), 7.63 (2H, d, J = 8.8 Hz, Ar), 7.47 – 7.32 (5H, m, Ar), 7.32 – 7.07 (5H, m, Ar), 6.91 – 6.77 (2H, m, Ar), 6.72 – 6.61 (2H, m, Ar), 5.03 (1H, *br.* s, C2-H), 4.25 (1H, d, J = 11.9 Hz, C7-H), 4.03 (1H, dd, J = 12.8, 5.3 Hz, C6-H$_A$), 3.73 (3H, s, OMe), 3.20 (1H, td, J = 12.8, 3.3 Hz, C6-H$_B$), 2.91 (1H, dt, J = 12.5, 3.2 Hz, C3-H), 1.98 – 1.81 (1H, m, C5-H$_A$), 1.70 – 1.51 (1H, m, C4-H$_A$), 1.51 – 1.38 (2H, m, C4-H$_B$ + C5-H$_B$). **^{13}C NMR (126 MHz, CDCl$_3$) δ** 158.8 (C Ar), 149.6 (C Ar), 145.9 (C Ar), 143.3 (C Ar), 143.0 (C Ar), 132.2 (C Ar), 129.0 (2 × C Ar), 129.0 (2 × C Ar), 128.4 (2 × C Ar), 128.3 (2 × C Ar), 128.2 (2 × C Ar), 128.1 (2 × C Ar), 127.1 (C Ar), 126.6 (C Ar), 123.8 (2 × C Ar), 113.9 (2 × C Ar), 58.0 (C2), 55.4 (OMe), 52.2 (C7), 42.7 (C3), 42.6 (C6), 19.9 (C4), 19.6 (C5).

Partial data for **5-*p*Ns-B** (from the mixture): **^{1}H NMR (500 MHz, CDCl$_3$) δ** 7.98 – 7.92 (2H, m, Ar), 7.40 (2H, d, J = 8.8 Hz, Ar), 6.59 – 6.50 (2H, m, Ar), 5.11 (1H, d, J = 5.4 Hz, C2-H), 3.95 – 3.86 (1H, m, C6-H$_A$), 3.75 (3H, s, OMe), 3.13 – 3.04 (2H, m, 1 × C6-H$_B$ + 1 × C7-H). **^{13}C NMR (126 MHz, CDCl$_3$) δ** 159.5 (C Ar), 149.3 (C Ar), 145.2 (C Ar), 143.2 (C Ar), 141.7 (C Ar), 131.5 (2 × C Ar), 128.9 (2 × C Ar), 128.8 (2 × C Ar), 128.6 (C Ar), 128.3 (2 × C Ar), 128.0 (2 × C Ar), 126.9 (C Ar), 126.6 (C Ar), 123.4 (2 × C Ar), 113.2 (2 × C Ar), 58.7 (C2), 55.4 (OMe), 55.1 (C7), 43.7 (C3), 41.8 (C6), 25.5 (C5), 23.0 (C4).

Data for **5-*p*Ns**: **R$_f$** 0.54 (20% EtOAc – pentane). **HRMS** (ESI): calculated for $C_{31}H_{30}O_5SN_2Na$ [M+Na]$^+$ requires m/z 565.1768, found m/z 565.1764. **IR** (film) ν_{max}: 3649, 3104, 3062, 3026, 2981, 2889, 2360, 2342, 1528, 1512 cm^{-1}.

(5-Cbz): (±)-Benzyl (2*S*,3*R*)-3-benzhydryl-2-(4-methoxyphenyl)piperidine-1-carboxylate

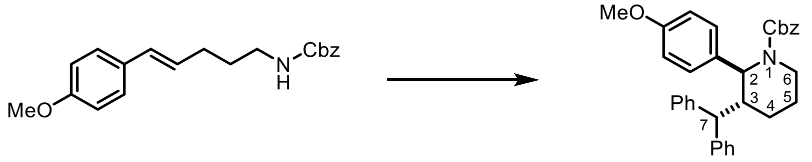

4-Cbz **5-Cbz**

Prepared according to **General Procedure K** using **4-Cbz** (13 mg, 0.040 mmol) and benzhydrol (7.5 mg, 0.040 mmol). Flash column chromatography (gradient elution 0% → 25% EtOAc – pentane) afforded **5-Cbz** as a colourless oil (17 mg, 86%).

Data for **5-Cbz**: **R$_f$** 0.39 (20% EtOAc – pentane). **^{1}H NMR (500 MHz, CDCl$_3$) δ** 7.42 – 7.05 (16H, m, Ar), 6.87 (2H, d, *J* = 8.8 Hz, Ar), 5.26 (1H, s, C2-H), 5.16 – 4.90 (2H, m, CH$_2$Ph), 4.32 – 4.14 (2H, m, C6-H$_A$ + C7-H), 3.79 (3H, s, OMe), 3.22 (1H, ddt, *J* = 12.1, 4.3, 2.2 Hz, C3-H), 2.90 (1H, td, *J* = 13.2, 3.5 Hz, C6-H$_B$), 1.84 – 1.72 (1H, m, C5-H$_A$), 1.63 – 1.50 (1H, m, C4-H$_A$), 1.46 – 1.39 (1H, m, C4-H$_B$), 1.30 – 1.22 (1H, m, C5-H$_B$). **^{13}C NMR (126 MHz, CDCl$_3$) δ** 158.4 (C Ar), 144.0 (C Ar), 143.6 (C Ar), 132.1 (C Ar), 128.9 (2 × C Ar), 128.9 (2 × C Ar), 128.4 (2 × C Ar), 128.1 (2 × C Ar), 127.9 (2 × C Ar), 127.8 (2 × C Ar), 127.5 (2 × C Ar), 127.1 (2 × C Ar), 126.6 (2 × C Ar), 126.5 (2 × C Ar), 114.2 (2 × C Ar), 67.2 (CH$_2$Ph), 55.4 (OMe), 55.1 (C2), 52.3 (C7), 40.2 (C6), 39.4 (C3), 21.0 (C4), 19.7 (C5). *A signal corresponding to C(O) was not observed.* **HRMS** (ESI): calculated for $C_{33}H_{34}O_3N$ [M+H]$^+$ requires m/z 492.2533, found m/z 492.2533. **IR** (film) ν_{max}: 3028, 2947, 2360, 1691, 1511, 1423, 1245, 1127, 1035, 732 cm^{-1}.

(5a): (±)-Methyl (2*S*,3*R*)-3-benzhydryl-2-(4-methoxyphenyl)piperidine-1-carboxylate

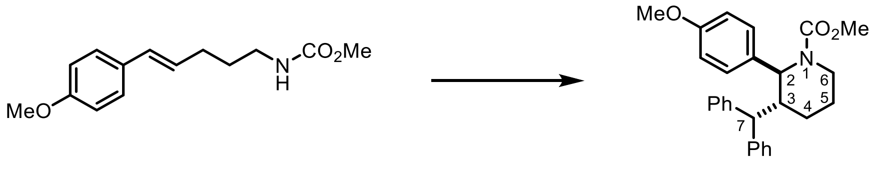

4a **5a**

Prepared according to **General Procedure K** using **4a** (25 mg, 0.10 mmol) and benzhydrol (19 mg, 0.10 mmol). Flash column chromatography (gradient elution 0% → 25% EtOAc – pentane) afforded **5a** as a colourless oil (40 mg, 98%).

Data for **5a**: **R$_f$** 0.37 (20% EtOAc – pentane). **^{1}H NMR (500 MHz, CDCl$_3$) δ** 7.43 – 7.36 (4H, m, Ar), 7.28 (4H, dt, *J* = 10.1, 7.7 Hz, Ar), 7.22 – 7.09 (4H, m, Ar), 6.91 – 6.86 (2H, m, Ar), 5.15 – 5.11 (1H,

br. s, C2-H), 4.23 (1H, *br*. s, C6-H_A), 4.19 (1H, d, *J* = 11.9 Hz, C7-H), 3.79 (3H, s, OMe), 3.56 (3H, s, OMe), 3.20 (1H, ddt, *J* = 11.8, 4.2, 2.2 Hz, C3-H), 2.88 (1H, td, *J* = 13.2, 3.5 Hz, C6-H_B), 1.85 – 1.67 (1H, m, C5-H_A), 1.55 (1H, tt, *J* = 13.7, 4.2 Hz, C4-H_A), 1.46 – 1.37 (1H, m, C4-H_B), 1.29 – 1.21 (1H, m, C5-H_B). **^{13}C NMR (126 MHz, $CDCl_3$) δ** 158.4 (C Ar), 157.5 (CO), 144.2 (C Ar), 143.5 (C Ar), 132.0 (C Ar), 128.9 (2 × C Ar), 128.8 (2 × C Ar), 128.1 (2 × C Ar), 128.0 (2 × C Ar), 127.4 (2 × C Ar), 126.7 (C Ar), 126.5 (C Ar), 114.2 (2 × C Ar), 55.4 (OMe), 55.0 (C2), 52.5 (C7), 52.4 (OMe), 40.0 (C6), 39.8 (C3), 21.0 (C4), 19.7 (C5). **HRMS** (ESI): calculated for $C_{27}H_{30}O_3N$ $[M+H]^+$ requires m/z 416.2220, found m/z 416.2222. **IR** (film) ν_{max}: 2950, 2360, 1694, 1511, 1446, 1348, 1249, 1179, 1141, 706 cm^{-1}.

(5b): (±)-Methyl (2*S*,3*R*)-3-(4-methoxybenzyl)-2-(4-methoxyphenyl)piperidine-1-carboxylate and (±)-Methyl (2*S*,3*S*)-3-(4-methoxybenzyl)-2-(4-methoxyphenyl)piperidine-1-carboxylate

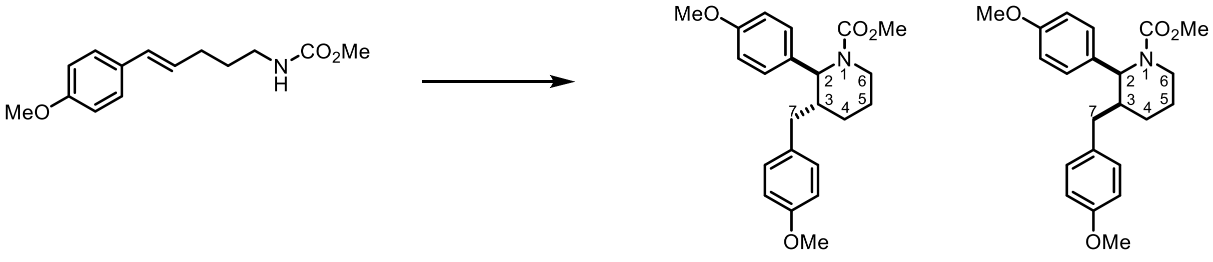

4a

5b
dr (C2-C3) 7.5:1

Prepared according to **General Procedure K** using **4a** (25 mg, 0.10 mmol) and 4-methoxybenzyl alcohol (14 mg, 0.10 mmol) at 70 °C. Flash column chromatography (gradient elution 0% → 25% EtOAc – pentane) afforded **5b** as an inseparable 7.5:1 mixture of diastereomers as a colourless oil (27 mg, 74%).

Data for **5b-A** (from the mixture): **^{1}H NMR (500 MHz, $CDCl_3$) δ** 7.19 – 7.13 (2H, m, Ar), 7.13 – 7.06 (2H, m, Ar), 6.88 – 6.82 (4H, m, Ar), 5.17 (1H, *br*. s, C2-H), 4.18 – 4.10 (1H, m, C6-H_A), 3.79 (3H, s, OMe), 3.78 (3H, s, OMe), 3.76 (3H, s, OMe), 2.88 – 2.82 (2H, m, C6-H_B + C7-H_A), 2.70 (1H, dd, *J* = 13.7, 7.0 Hz, C7-H_B), 2.53 (1H, tq, *J* = 6.8, 3.2 Hz, C3-H), 1.81 (1H, qt, *J* = 13.0, 4.6 Hz, C5-H_A), 1.68 – 1.58 (1H, m, C4-H_A), 1.52 – 1.44 (1H, m, C4-H_B), 1.41 – 1.33 (1H, m, C5-H_B). **^{13}C NMR (126 MHz, $CDCl_3$) δ** 158.4 (C Ar), 158.1 (C Ar), 157.5 (CO), 132.9 (C Ar), 132.1 (C Ar), 130.2 (2 × C Ar), 127.7 (2 × C Ar), 114.1 (2 × C Ar), 113.9 (2 × C Ar), 56.3 (C2), 55.4 (OMe), 55.4 (OMe), 52.9 (OMe), 40.1 (C6), 38.3 (C3), 37.3 (C7), 23.4 (C4), 20.3 (C5).

Partial data for **5b-B** (from the mixture): **^{1}H NMR (500 MHz, $CDCl_3$) δ** 7.01 – 6.89 (2H, d, *J* = 8.7 Hz, Ar), 6.78 (2H, d, *J* = 8.7 Hz, Ar), 3.81 (3H, s, OMe), 3.78 (3H, s, OMe), 3.63 (3H, s, OMe), 3.14 (1H, td, *J* = 13.4, 3.3 Hz, C6-H_A), 2.45 (1H, m, C7-H_A), 2.25 – 2.04 (2H, m, C3-H + C7-H_B). **^{13}C NMR (126 MHz, $CDCl_3$) δ** 158.8 (C Ar), 158.0 (C Ar), 132.2 (C Ar), 130.1 (2 × C Ar), 113.8 (2 × C Ar), 113.6 (2 × C Ar), 55.4 (OMe), 55.3 (OMe), 52.6 (OMe), 41.9 (C6), 39.3 (C3).

Data for **5b**: **R_f** 0.30 (20% EtOAc – pentane). **HRMS** (ESI): calculated for $C_{22}H_{27}O_4NNa$ $[M+Na]^+$ requires m/z 392.1832, found m/z 392.1832. **IR** (film) ν_{max}: 2938, 1694, 1611, 1511, 1443, 1408, 1247, 1179, 1125, 1037 cm^{-1}.

(5c): (±)-Methyl (2*S*,3*R*)-3-cinnamyl-2-(4-methoxyphenyl)piperidine-1-carboxylate

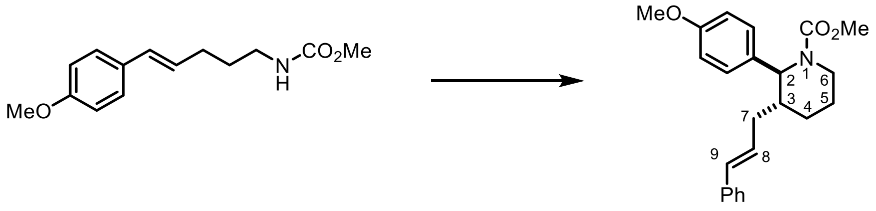

4a **5c**

Prepared according to **General Procedure K** using **4a** (21 mg, 0.080 mmol) and cinnamyl alcohol (11 mg, 0.080 mmol) at 70 °C. Flash column chromatography (gradient elution 0% → 25% EtOAc – pentane) afforded **5c** as a colourless oil (15 mg, 51%).

Data for **5c**: **R_f** 0.57 (20% EtOAc – pentane). **^{1}H NMR (500 MHz, $CDCl_3$) δ** 7.41 – 7.33 (2H, m, Ar), 7.31 – 7.28 (2H, m, Ar), 7.23 – 7.18 (1H, m, Ar), 7.18 – 7.14 (2H, m, Ar), 6.91 – 6.83 (2H, m, Ar), 6.46 (1H, d, *J* = 15.8 Hz, C9-H), 6.27 (1H, dt, *J* = 15.7, 7.2 Hz, C8-H), 5.24 (1H, *br.* s, C2-H), 4.18 – 4.05 (1H, m, C6-H_A), 3.80 (3H, s, OMe), 3.75 (3H, s, OMe), 2.84 (1H, td, *J* = 12.9, 3.5 Hz, C6-H_B), 2.52 (1H, dtd, *J* = 13.1, 7.2, 1.3 Hz, C7-H_A), 2.45 (1H, dq, *J* = 7.0, 3.1 Hz, C3-H), 2.38 (1H, dtd, *J* = 13.9, 7.0, 1.3 Hz, C7-H_B), 1.76 (1H, dt, *J* = 12.7, 4.5 Hz, C5-H_A), 1.69 (1H, tt, *J* = 12.9, 3.8 Hz, C4-H_A), 1.60 – 1.52 (1H, m, C4-H_B), 1.43 – 1.33 (1H, m, C5-H_B). **^{13}C NMR (126 MHz, $CDCl_3$) δ** 158.4 (C Ar), 157.5 (CO), 137.7 (C Ar), 132.2 (C9), 132.0 (C Ar), 128.8 (C8), 128.7 (2 × C Ar), 127.8 (2 × C Ar), 127.2 (2 × C Ar), 126.2 (2 × C Ar), 114.1 (2 × C Ar), 56.7 (C2), 55.4 (OMe), 52.9 (COMe), 40.0 (C6), 36.1 (C3), 35.5 (C7), 23.4 (C4), 20.2 (C5). **HRMS** (ESI): calculated for $C_{23}H_{27}O_3NNa$ $[M+Na]^+$ requires m/z 388.1883, found m/z 388.1884. **IR** (film) ν_{max}: 3649, 2981, 2360, 2342, 1693, 1511, 1443, 1407, 1248, 1178 cm^{-1}.

(5d): (±)-Methyl (2*S*,3*R*)-2-(4-methoxyphenyl)-3-((*E*)-3-(4-methoxyphenyl)allyl)piperidine-1-carboxylate

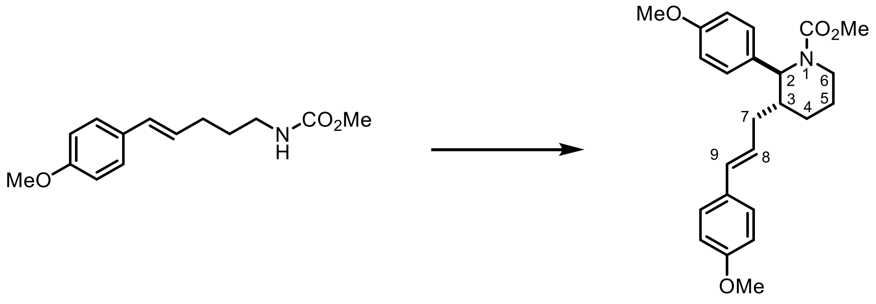

4a **5d**

Prepared according to **General Procedure K** using **4a** (10.5 mg, 0.040 mmol) and **2d** (6.5 mg, 0.040 mmol) at 70 °C. Flash column chromatography (gradient elution 0% → 25% EtOAc – pentane) afforded **5d** as a colourless oil (8 mg, 51%).

Data for **5d**: $\mathbf{R_f}$ 0.48 (20% EtOAc – pentane). **^{1}H NMR (500 MHz, $CDCl_3$) δ** 7.31 – 7.27 (2H, m, Ar), 7.20 – 7.11 (2H, m, Ar), 6.90 – 6.86 (2H, m, Ar), 6.86 – 6.81 (2H, m, Ar), 6.40 (1H, d, *J* = 15.7 Hz, C9-H), 6.11 (1H, dt, *J* = 15.6, 7.2 Hz, C8-H), 5.23 (1H, *br.* s, C2-H), 4.11 (1H, d, *J* = 13.1 Hz, C6-H_A), 3.80 (3H, s, OMe), 3.80 (3.80, s, OMe), 3.75 (3H, s, OMe), 2.83 (1H, td, *J* = 13.0, 3.5 Hz, C6-H_B), 2.49 (1H, dtd, *J* = 13.0, 7.2, 1.3 Hz, C7-H_A), 2.42 (1H, tq, *J* = 6.6, 3.0 Hz, C3-H), 2.38 – 2.31 (1H, m, C7-H_B), 1.81 – 1.62 (2H, m, C4-H_A + C5-H_A), 1.57 – 1.52 (1H, m, C4-H_B), 1.42 – 1.33 (1H, m, C5-H_B). **^{13}C NMR (126 MHz, $CDCl_3$) δ** 159.0 (C Ar), 158.4 (C Ar), 157.5 (CO), 132.1 (C Ar), 131.5 (C9), 130.5 (C Ar), 127.8 (2 × C Ar), 127.3 (2 × C Ar), 126.6 (C8), 114.0 (2 × C Ar), 114.1 (2 × C Ar), 56.7 (C2), 55.5 (OMe), 55.4 (OMe), 52.9 (OMe), 40.0 (C6), 36.2 (C3), 35.4 (C7), 23.4 (C4), 20.2 (C5). **HRMS** (ESI): calculated for $C_{24}H_{29}O_4NNa$ $[M+Na]^+$ requires m/z 418.1989, found m/z 418.1984. **IR** (film) ν_{max}: 3649, 2981, 2971, 2837, 2359, 2342, 1694, 1510, 1443, 1407 cm^{-1}.

(5e): (±)-Methyl (2*S*,3*R*)-3-((*S*,*E*)-1,3-diphenylallyl)-2-(4-methoxyphenyl)piperidine-1-carboxylate and (±)-Methyl (2*S*,3*R*)-3-((*R*,*E*)-1,3-diphenylallyl)-2-(4-methoxyphenyl)-piperidine-1-carboxylate

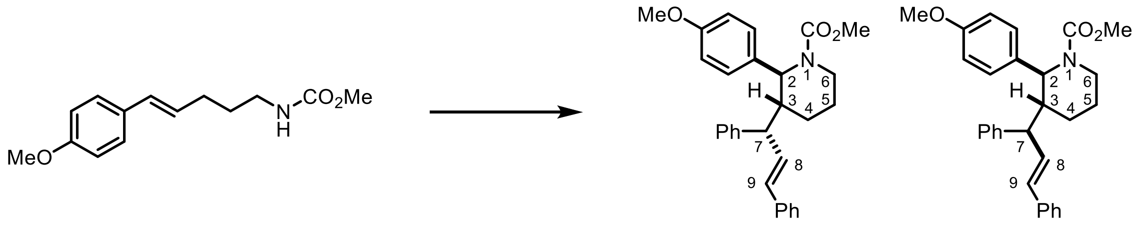

4a **A** **5e** **B**

d.r. (C-7) 8:1

Prepared according to **General Procedure K** using **4a** (25 mg, 0.10 mmol) and (*E*)-1,3-diphenyl-2-propen-1-ol (21 mg, 0.10 mmol) at rt. Flash column chromatography (gradient elution 0% → 25% EtOAc – pentane) afforded **5e** as an inseparable 8:1 mixture of diastereomers as a colourless oil (38 mg, 85%).

Data for **5e-A** (from the mixture): **^{1}H NMR (500 MHz, $CDCl_3$) δ** 7.39 – 7.29 (6H, m, Ar), 7.28 – 7.22 (3H, m, Ar), 7.19 – 7.15 (3H, m, Ar), 6.89 (2H, d, *J* = 8.8 Hz, Ar), 6.49 (1H, d, *J* = 15.6 Hz, C9-H), 6.37 (1H, dd, *J* = 15.7, 9.4 Hz, C8-H), 5.61 (1H, s, C2-H), 4.21 (1H, d, *J* = 14.0 Hz, C6-H_A), 3.80 (3H, s, OMe), 3.72 (3H, s, OMe), 3.67 (1H, d, *J* = 16.4 Hz, C7-H), 2.86 (1H, td, *J* = 13.2, 3.3 Hz, C6-H_B), 2.69 (1H, dp, *J* = 11.2, 2.2 Hz, C3-H), 1.71 (1H, dt, *J* = 13.0, 4.3 Hz, C5-H_A), 1.46 (1H, tt, *J* = 14.6, 4.2 Hz, C4-H_A), 1.30 – 1.16 (2H, m, C4-H_B + C5-H_B). **^{13}C NMR (126 MHz, $CDCl_3$) δ** 158.4 (C Ar), 157.6 (CO), 143.0 (C Ar), 137.4 (C Ar), 132.9 (C8), 132.1 (C9), 129.1 (2 × C Ar), 128.6 (2 × C Ar), 127.9 (2 × C Ar), 127.6 (2 × C Ar), 127.4 (C Ar), 127.4 (C Ar), 126.6 (C Ar), 126.3 (2 × C Ar), 114.2 (2 × C Ar), 55.5 (OMe), 54.9 (C2), 52.9 (OMe), 50.3 (C7), 40.7 (C3), 39.9 (C6), 20.8 (C4), 20.0 (C5).

Partial data for **5e-B** (from the mixture): **^{1}H NMR (500 MHz, $CDCl_3$) δ** 7.11 – 7.01 (2H, m, Ar), 6.86 – 6.82 (2H, m, Ar), 4.99 (1H, *br.* s, C2-H), 3.78 (3H, s, OMe), 2.92 (1H, td, *J* = 13.1, 3.5 Hz, C6-H_B), 2.73 (1H, d, *J* = 11.6 Hz, C3-H), 1.85 (1H, m, C5-H_A), 1.36 (1H, d, *J* = 13.0 Hz, C5-H_B).**^{13}C NMR (126 MHz, $CDCl_3$) δ** 158.4 (C Ar), 143. (C Ar), 132.2 (C9), 132.0 (C8), 130.6 (C Ar), 128.9 (2 × C Ar), 128.6 (2 × C Ar), 128.1 (2 × C Ar), 126.8 (C Ar), 126.3 (2 × C Ar), 114.1 (2 × C Ar), 55.4 (OMe), 54.6 (C7), 52.6 (OMe), 41.2 (C3), 21.4 (C4, 19.5 (C5).

Data for **5e**: **R_f** 0.52 (20% EtOAc – pentane). **HRMS** (ESI): calculated for $C_{29}H_{31}O_3NNa$ $[M+Na]^+$ requires m/z 464.2196, found m/z 464.2195. **IR** (film) ν_{max}: 3649, 3059, 3025, 2981, 2954, 2836, 2358, 2338, 2246, 1691 cm^{-1}.

(5f): (±)-Methyl (2*S*,3*R*)-2-(4-methoxyphenyl)-3-((*S*,*E*)-pent-3-en-2-yl)piperidine-1-carboxylate and (±)-Methyl (2*S*,3*R*)-2-(4-methoxyphenyl)-3-((*R*,*E*)-pent-3-en-2-yl)piperidine-1-carboxylate

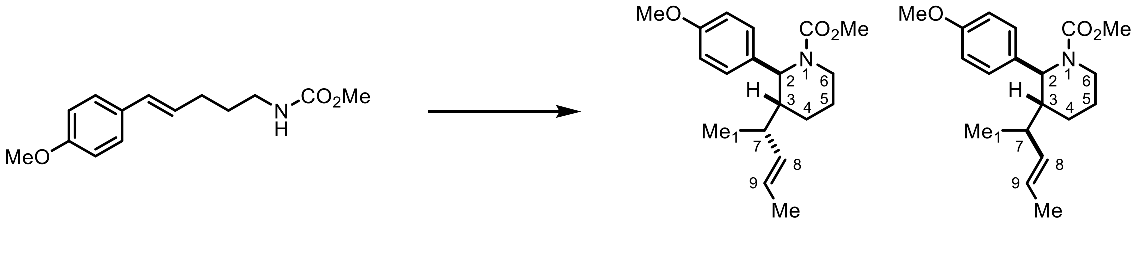

4a **A** **5f** **B**
d.r. (C-7) 2.5:1

Prepared according to **General Procedure K** using **4a** (10 mg, 0.040 mmol) and 3-penten-2-ol (7 mg, 0.08 mmol, 2.0 equiv.) at 0 °C for 48 h. Flash column chromatography (gradient elution 0% → 25% EtOAc – pentane) afforded **5f** as an inseparable 2.5:1 mixture of diastereomers as a colourless oil (12 mg, 95%).

Data for **5f-A** (from the mixture): **^{1}H NMR (500 MHz, CDCl$_3$) δ** 7.11 (2H, d, *J* = 8.9 Hz, Ar), 6.87 (2H, d, *J* = 8.8 Hz, Ar), 5.46 (1H, ddq, *J* = 15.0, 8.6, 6.3 Hz, C9-H), 5.36 (1H, *br.* s, C2-H), 5.31 (1H, ddd, *J* = 15.1, 8.8, 1.6 Hz, C8-H), 4.15 – 4.08 (1H, m, C6-H_A), 3.80 (3H, s, OMe), 3.70 (3H, s, OMe), 2.89 – 2.80 (1H, m, C6-H_B), 2.39 – 2.27 (1H, m, C7-H), 1.95 – 1.88 (1H, m, C3-H), 1.66 (3H, dd, *J* = 6.5, 1.6 Hz, Me_2), 1.69 – 1.57 (2H, m, C4-H_A + C5-H_A), 1.55 – 1.44 (1H, m, C4-H_B), 1.38 – 1.30 (1H, m, C5-H_B), 1.01 (3H, d, *J* = 6.7 Hz, Me_1). **^{13}C NMR (126 MHz, CDCl$_3$) δ** 158.3 (C Ar), 157.4 (CO), 136.4 (C8), 132.7 (C Ar), 127.6 (2 × C Ar), 125.8 (C9), 114.1 (2 × C Ar), 55.5 (OMe), 55.4 (C2), 52.7 (OMe), 41.4 (C3), 40.1 (C6), 36.5 (C7), 20.6 (C4), 20.3 (C5), 18.5 (Me_1), 18.2 (Me_2).

Partial data for **5f-B** (from the mixture): **^{1}H NMR (500 MHz, CDCl$_3$) δ** 7.15 (2H, d, *J* = 8.9 Hz, Ar), 6.88 (2H, d, *J* = 9.0 Hz, Ar), 4.08 – 4.02 (1H, m, C6-H_A), 3.80 (3H, s, OMe), 3.73 (3H, s, OMe), 1.12 (3H, d, *J* = 6.6 Hz, Me_1). **^{13}C NMR (126 MHz, CDCl$_3$) δ** 158.3 (C Ar), 157.4 (CO), 135.5 (C8), 132.5 (C Ar), 127.8 (2 × C Ar), 124.4 (C9), 55.4 (OMe), 54.9 (C2), 52.9 (OMe), 41.4 (C3), 39.8 (C6), 36.6 (C7), 21.5 (C4), 20.5 (Me_1), 20.1 (C5), 18.1 (Me_2).

Data for **5f**: **R_f** 0.46 (20% EtOAc – pentane). **HRMS** (ESI): calculated for $C_{19}H_{28}O_3N$ $[M+H]^+$ requires m/z 318.2064, found m/z 318.2066. **IR** (film) ν_{max}: 3735, 3649, 2980, 2971, 2338, 4697, 1512, 1444, 1407, 1262 cm^{-1}.

(5g): (±)-Methyl (2*S*,3*R*)-3-((*R*)-cyclopent-2-en-1-yl)-2-(4-methoxyphenyl)piperidine-1-carboxylate and (±)-Methyl (2*S*,3*R*)-3-((*S*)-cyclopent-2-en-1-yl)-2-(4-methoxyphenyl)-piperidine-1-carboxylate

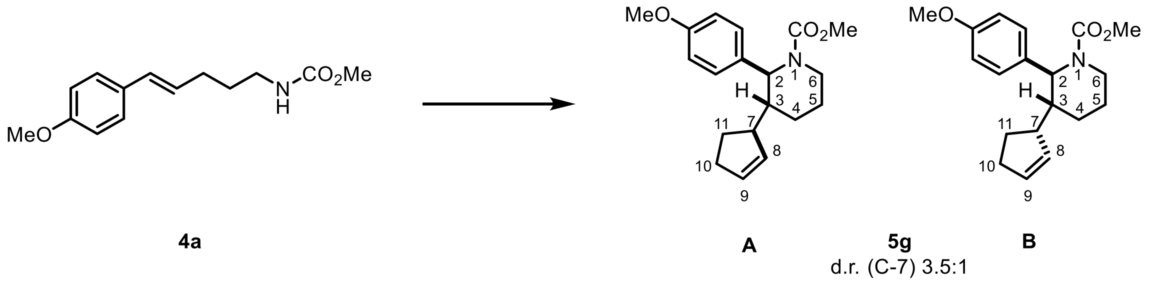

Prepared according to **General Procedure K** using **4a** (10 mg, 0.040 mmol) and **2o** (13 mg, 0.08 mmol, 2.0 equiv.) at 0 °C for 48 h. Flash column chromatography (gradient elution 0% → 25% EtOAc – pentane) afforded **5g** as an inseparable 3.5:1 mixture of diastereomers as a colourless oil (12 mg, 95%).

Data for **5g-A** (from the mixture): **^{1}H NMR (500 MHz, CDCl$_3$) δ** 7.14 (2H, d, *J* = 8.9 Hz, Ar), 6.88 (2H, d, *J* = 8.7 Hz, Ar), 5.83 (1H, dt, *J* = 6.4, 2.2 Hz, C8-H), 5.78 (1H, dq, *J* = 5.9, 2.1 Hz, C9-H), 5.31 (1H, s, C2-H), 4.14 – 4.04 (1H, m, C6-H$_A$), 3.80 (3H, s, OMe), 3.74 (3H, s, OMe), 3.07 – 2.96 (1H, m, C7-H), 2.86 (1H, td, *J* = 13.1, 3.5 Hz, C6-H$_B$), 2.45 – 2.38 (1H, m, C10-H$_A$), 2.38 – 2.30 (1H, m, C10-H$_B$), 2.25 (1H, ddd, *J* = 12.6, 8.3, 4.0 Hz, C11-H$_A$), 2.07 – 1.97 (1H, m, C3-H), 1.79 – 1.73 (1H, m, C5-H$_A$), 1.72 – 1.66 (1H, m, C4-H$_A$), 1.66 – 1.53 (2H, m, 1 × C4-H$_B$ + 1 × C11-H$_B$), 1.36 (1H, ddd, *J* = 12.9, 5.2, 2.9 Hz, C5-H$_B$). **^{13}C NMR (126 MHz, CDCl$_3$) δ** 158.4 (C Ar), 157.5 (CO), 132.6 (C9), 132.2 (C8), 132.0 (C Ar), 127.7 (2 × C Ar), 114.1 (2 × C Ar), 56.3 (C2), 55.4 (OMe), 52.9 (COMe), 45.3 (C7), 41.9 (C3), 40.1 (C6), 32.2 (C10), 30.2 (C11), 22.2 (C4), 20.4 (C5).

Partial data for **5g-B** (from the mixture): **^{1}H NMR (500 MHz, CDCl$_3$) δ** 5.92 (1H, dq, *J* = 6.1, 2.1 Hz, C8-H), 5.82 – 5.80 (1H, m, C9-H), 5.42 (1H, d, *J* = 2.3 Hz, C2-H), 3.73 (3H, s, OMe), 2.09 – 2.05 (1H, m, C3-H), 1.51 – 1.45 (1H, m, C4-H). **^{13}C NMR (126 MHz, CDCl$_3$) δ** 157.4 (CO), 134.0 (C8), 131.6 (C9), 127.8 (2 × C Ar), 114.1 (2 × C Ar), 56.2 (C2), 45.3 (C7), 41.3 (C3), 40.0 (C6), 32.3 (C10), 29.9 (C11), 21.7 (C4), 20.3 (C3).

Data for **5g**: **R$_f$** 0.43 (20% EtOAc – pentane). **HRMS** (ESI): calculated for $C_{19}H_{26}O_3N$ $[M+H]^+$ requires m/z 316.1907, found m/z 316.1910. **IR** (film) ν_{max}: 3735, 3649, 3050, 2981, 2971, 2954, 2868, 2380, 2341, 1697 cm^{-1}.

(5h): (±)-Methyl (2*S*,3*R*)-2-(4-methoxyphenyl)-3-((*R*)-3-phenylcyclopent-2-en-1-yl)piperidine-1-carboxylate and (±)-Methyl (2*S*,3*R*)-2-(4-methoxyphenyl)-3-((*S*)-3-phenylcyclopent-2-en-1-yl)-piperidine-1-carboxylate

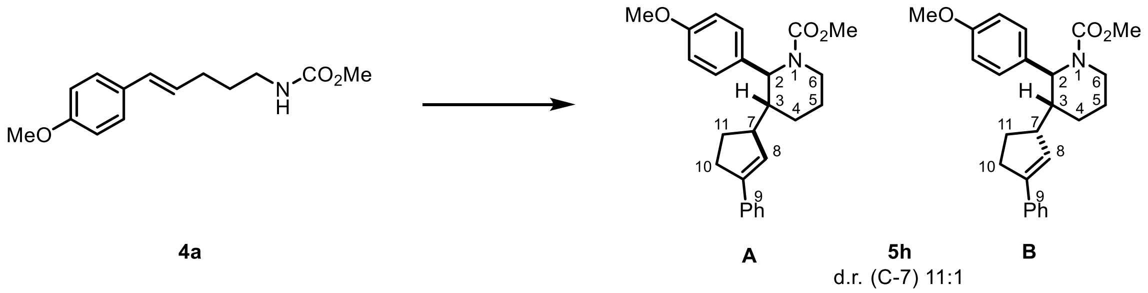

Prepared according to **General Procedure K** using **4a** (25 mg, 0.10 mmol) and **2q** (16 mg, 0.10 mmol). Flash column chromatography (gradient elution 0% → 25% EtOAc – pentane) afforded **5h** as an inseparable 11:1 mixture of diastereomers as a yellow oil (39 mg, 96%).

Data for **5h-A** (from the mixture): **^{1}H NMR (500 MHz, CDCl$_3$) δ** 7.51 – 7.43 (2H, m, Ar), 7.38 – 7.28 (2H, m, Ar), 7.28 – 7.21 (1H, m, Ar), 7.21 – 7.10 (2H, m, Ar), 6.93 – 6.84 (2H, m, Ar), 6.24 (1H, d, *J* = 2.0 Hz, C8-H), 5.36 (1H, s, C2-H), 4.21 – 4.06 (1H, m, C6-H$_A$), 3.81 (3H, s, OMe), 3.77 (3H, s, OMe), 3.23 – 3.14 (1H, m, C7-H), 2.89 (1H, td, *J* = 12.9, 3.5 Hz, C6-H$_B$), 2.84 – 2.80 (1H, m, C11-H$_A$), 2.77 – 2.70 (1H, m, C11-H$_B$), 2.43 (1H, dtd, *J* = 12.6, 8.5, 4.1 Hz, C10-H$_A$), 2.17 – 2.13 (1H, m, C3-H), 1.89 – 1.70 (3H, m, C4-H$_A$ + C5-H$_A$ + C10-H$_B$), 1.68 – 1.64 (1H, m, C4-H$_B$), 1.46 – 1.36 (1H, m, C5-H$_B$). **^{13}C NMR (126 MHz, CDCl$_3$) δ** 158.4 (C Ar), 157.5 (CO), 143.4 (C Ar), 136.7 (C Ar), 132.1 (C9), 128.5 (2 × C Ar), 127.7 (2 × C Ar), 127.6 (C8), 127.3 (C Ar), 125.8 (2 × C Ar), 114.1 (2 × C Ar), 56.1 (C2), 55.4 (OMe), 52.9 (OMe), 45.9 (C7), 41.9 (C3), 40.1 (C6), 32.7 (C11), 30.5 (C10), 22.3 (C4), 20.5 (C5).

Characteristic signals for **5h-B** (from the mixture): **^{1}H NMR (500 MHz, CDCl$_3$) δ** 6.37 (1H, s, C8-H), 5.52 (1H, s, C2-H), 3.74 (3H, s, OMe).

Data for **5h**: **R$_f$** 0.59 (20% EtOAc – pentane). **HRMS** (ESI): calculated for $C_{25}H_{29}O_3NNa$ $[M+Na]^+$ requires m/z 414.2040, found m/z 414.2040. **IR** (film) ν_{max}: 2946, 1695, 1612, 1511, 1445, 1408, 1345, 1250, 1179, 1122 cm^{-1}.

(5i): (±)-Methyl (2*S*,3*R*)-3-benzhydryl-2-phenylpiperidine-1-carboxylate

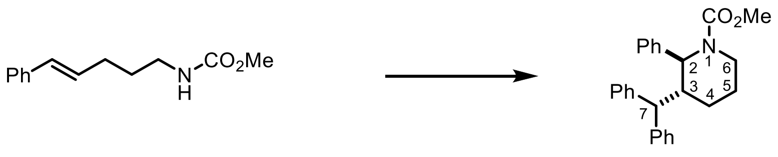

4b **5i**

Prepared according to **General Procedure K** using **4b** (18 mg, 0.08 mmol) and benzhydrol (15 mg, 0.08 mmol) at 70 °C. Flash column chromatography (gradient elution 0% → 25% EtOAc – pentane) afforded **5i** as a colourless oil (20 mg, 65%).

Data for **5i**: **R$_f$** 0.42 (20% EtOAc – pentane). **^{1}H NMR (500 MHz, CDCl$_3$) δ** 7.41 (4H, ddd, *J* = 8.1, 4.8, 1.4 Hz, Ar), 7.35 (2H, dd, *J* = 8.5, 6.6 Hz, Ar), 7.32 – 7.26 (4H, m, Ar), 7.25 – 7.20 (3H, m, Ar), 7.20 – 7.12 (2H, m, Ar), 5.18 (1H, s, C2-H), 4.27 (1H, *br.* s, C6-H$_A$), 4.20 (1H, d, *J* = 12.0 Hz, C7-H), 3.54 (3H, *br.* s, OMe), 3.25 (1H, ddt, *J* = 12.2, 4.3, 2.1 Hz, C3-H), 2.96 – 2.77 (1H, m, C6-H$_B$), 1.78 (1H, d, *J* = 13.3 Hz, C5-H$_A$), 1.52 (1H, tt, *J* = 13.5, 4.1 Hz, C4-H$_A$), 1.47 – 1.39 (1H, m, C4-H$_B$), 1.29 – 1.21 (2H, m, C5-H$_B$). **^{13}C NMR (126 MHz, CDCl$_3$) δ** 157.6 (CO), 144.1 (C Ar), 143.4 (C Ar), 140.2 (C Ar), 128.9 (2 × C Ar), 128.9 (2 × C Ar), 128.9 (2 × C Ar), 128.1 (2 × C Ar), 128.0 (2 × C Ar), 126.8 (C Ar), 126.7 (C Ar), 126.5 (C Ar), 126.3 (2 × C Ar), 55.5 (C2), 52.4 (OMe), 52.5 (C7), 40.1 (C6), 39.9 (C3), 21.0 (C4), 19.6 (C5). **HRMS** (ESI): calculated for $C_{26}H_{28}O_2N$ $[M+H]^+$ requires m/z 386.2115, found m/z 386.2118. **IR** (film) ν_{max}: 2948, 1696, 1599, 1494, 1446, 1408, 1348, 1266, 1191, 1132 cm^{-1}.

(5i-Ns): (±)-(2*S*,3*R*)-3-Benzhydryl-1-((2-nitrophenyl)sulfonyl)-2-phenylpiperidine

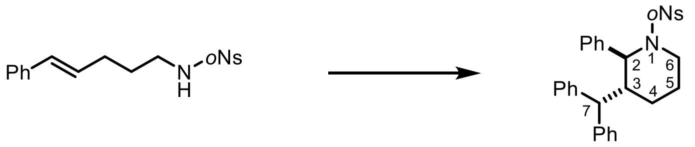

4b-Ns **5i-Ns**

Prepared according to **General Procedure K** using **4bNs** (28 mg, 0.08 mmol) and benzhydrol (15 mg, 0.08 mmol) in 3:1 HFIP:CH_2Cl_2. Flash column chromatography (gradient elution 0% → 25% EtOAc – pentane) afforded **5i-Ns** as a white solid (31 mg, 69%).

Data for **5i-Ns**: **R$_f$** 0.23 (20% EtOAc – pentane). **^{1}H NMR (400 MHz, CDCl$_3$) δ** 7.60 (1H, dd, *J* = 8.0, 1.5 Hz, Ar), 7.52 (1H, td, *J* = 7.5, 1.5 Hz, Ar), 7.42 (1H, dd, *J* = 8.0, 1.5 Hz, Ar), 7.36 – 7.07 (15H, m, Ar), 5.02 (1H, s, C2-H), 4.36 – 4.20 (2H, m, C6-H$_A$ + C7-H), 3.53 (1H, td, *J* = 13.0, 3.0 Hz, C6-H$_B$), 3.09 – 3.05 (1H, m, C3-H), 1.98 – 1.95 (1H, m, C5-H$_A$), 1.51 – 1.45 (1H, m, C4-H$_A$), 1.45 – 1.35 (2H, m, C4-H$_B$ + C5-H$_B$). **^{13}C NMR (101 MHz, CDCl$_3$) δ** 147.8 (C Ar), 143.5 (C Ar), 143.4 (C Ar), 140.1

(C Ar), 134.2 (C Ar), 133.0 (C Ar), 131.7 (C Ar), 131.7 (C Ar), 129.0 (2 × C Ar) 128.9 (2 × C Ar), 128.5 (2 × C Ar), 128.3 (2 × C Ar), 128.1 (2 × C Ar), 126.9 (C Ar), 126.8 (2 × C Ar), 126.6 (C Ar), 126.5 (C Ar), 123.9 (C Ar), 58.5 (C2), 51.9 (C7), 43.8 (C6), 42.7 (C3), 20.5 (C4), 19.7 (C5). **HRMS** (ESI): calculated for $C_{30}H_{32}O_4SN_2Na$ $[M+Na]^+$ requires m/z 535.1658, found m/z 535.1660. **IR** (film) ν_{max}: 2980, 2925, 1542, 1379, 1346, 1164, 1134, 942, 759, 706 cm^{-1}.

(5j): (±)-Methyl (2*S*,3*R*)-3-benzhydryl-2-(4-bromophenyl)piperidine-1-carboxylate

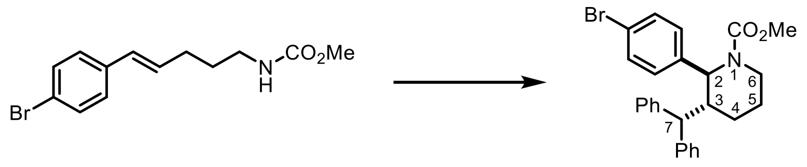

4c **5j**

Prepared according to **General Procedure K** using **4c** (12 mg, 0.040 mmol) and benzhydrol (7.5 mg, 0.08 mmol) at 70 °C. Flash column chromatography (gradient elution 0% → 15% EtOAc – pentane) afforded **5j** as a colourless oil (12 mg, 65%).

Data for **5j**: $\mathbf{R_f}$ 0.44 (20% EtOAc – pentane). **^{1}H NMR (500 MHz, $CDCl_3$)** 7.46 (2H, d, *J* = 8.6 Hz, Ar), 7.42 – 7.35 (4H, m, Ar), 7.29 (4H, td, *J* = 7.7, 5.8 Hz, Ar), 7.20 – 7.13 (2H, m, Ar), 7.09 (2H, d, *J* = 7.5 Hz, Ar), 5.11 (1H, *br.* s, C2-H), 4.25 (1H, *br.* s, C6-H_A), 4.17 (1H, d, *J* = 12.0 Hz, C7-H), 3.58 (3H, *br.* s, OMe), 3.17 (1H, ddt, *J* = 12.1, 4.0, 2.2 Hz, C3-H), 2.93 – 2.76 (1H, m, C6-H_B), 1.84 – 1.69 (1H, m, C5-H_A), 1.49 – 1.41 (2H, m, C4-H_2), 1.29 – 1.22 (1H, m, C5-H_B). **^{13}C NMR (126 MHz, $CDCl_3$) δ** 157.4 (CO), 143.9 (C Ar), 143.1 (C Ar), 139.4 (C Ar), 131.9 (2 × C Ar), 129.0 (2 × C Ar), 128.9 (2 × C Ar), 128.2 (2 × C Ar), 128.1 (2 × C Ar), 128.0 (2 × C Ar), 126.8 (C Ar), 126.6 (C Ar), 120.6 (C Ar), 55.2 (C2), 52.7 (OMe), 52.4 (C7), 40.0 (C3 + C6), 21.0 (C4), 19.5 (C6). **HRMS** (ESI): calculated for $C_{26}H_{27}O_2N^{78}Br$ $[M+H]^+$ requires m/z 464.1220, found m/z 464.1220. **IR** (film) ν_{max}: 2949, 1694, 1489, 1445, 1410, 1347, 1262, 1191, 1131, 1075 cm^{-1}.

(5k): (±)-Methyl (2*S*,3*R*)-3-(4-methoxybenzyl)-2-phenylpiperidine-1-carboxylate

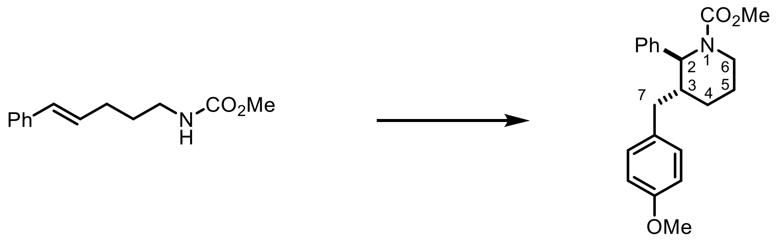

4b **5k**

Prepared according to **General Procedure K** using **4b** (9 mg, 0.04 mmol) and 4-methoxybenzyl alcohol (5.5 mg, 0.040 mmol) at 70 °C. Flash column chromatography (gradient elution 0% → 15% EtOAc – pentane) afforded **5k** as a colourless oil (10 mg, 74%).

Data for **5k**: **R_f** 0.33 (20% EtOAc – pentane). **^{1}H NMR (500 MHz, $CDCl_3$) δ** 7.32 (2H, t, *J* = 7.7 Hz, Ar), 7.24 – 7.12 (5H, m, Ar), 6.85 (2H, d, *J* = 8.6 Hz, Ar), 5.22 (1H, s, C2-H), 4.22 – 4.12 (1H, m, C6-H_A), 3.79 (3H, s, OMe), 3.77 (3H, s, OMe), 2.93 – 2.80 (2H, m, C6-H_B + C7-H_A), 2.72 (1H, dd, *J* = 13.7, 6.8 Hz, C7-H_B), 2.59 (1H, q, *J* = 6.8, 5.0 Hz, C3-H), 1.82 (1H, tdt, *J* = 13.0, 9.2, 4.6 Hz, C5-H_A), 1.69 – 1.55 (1H, m, C4-H_A), 1.49 (1H, dd, *J* = 13.5, 3.6 Hz, C4-H_B), 1.38 (1H, dq, *J* = 13.3, 3.4 Hz, C5-H_B). **^{13}C NMR (126 MHz, $CDCl_3$) δ** 158.1 (C Ar), 157.6 (C8), 140.2 (C Ar), 132.9 (C Ar), 130.2 (2 × C Ar), 128.7 (2 × C Ar), 126.7 (C Ar), 126.6 (2 × C Ar), 114.0 (2 × C Ar), 56.7 (C2), 55.4 (OMe), 52.9 (C9), 40.4 (C6), 38.4 (C3), 37.3 (C7), 23.5 (C4), 20.2 (C5). **HRMS** (ESI): calculated for $C_{21}H_{25}O_3N$ $[M+H]^+$ requires m/z 340.1907, found m/z 340.1908. **IR** (film) ν_{max}: 2942, 1697, 1512, 1445, 1408, 1247, 1179, 1126, 1035, 807 cm^{-1}.

(5l): (±)-Methyl (2*S*,3*R*)-2-(4-bromophenyl)-3-(4-methoxybenzyl)piperidine-1-carboxylate

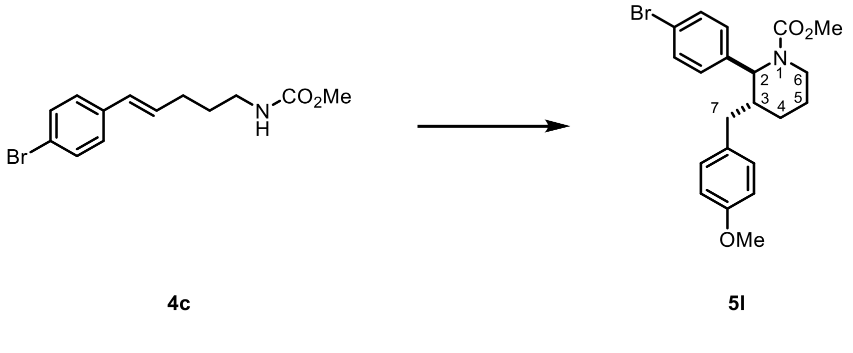

4c **5l**

Prepared according to **General Procedure K** using **4c** (12 mg, 0.04 mmol) and 4-methoxybenzyl alcohol (5.5 mg, 0.040 mmol) at 70 °C. Flash column chromatography (gradient elution 0% → 15% EtOAc – pentane) afforded **5l** as a colourless oil (10 mg, 60%).

Data for **5l**: **R_f** 0.33 (20% EtOAc – pentane). **^{1}H NMR (500 MHz, $CDCl_3$) δ** 7.43 (2H, d, *J* = 8.6 Hz, Ar), 7.15 (2H, d, *J* = 8.6 Hz, Ar), 7.04 (2H, d, *J* = 8.6 Hz, Ar), 6.85 (2H, d, *J* = 8.6 Hz, Ar), 5.15 (1H,

br. s, C2-H), 4.21 – 4.12 (1H, m, C6-H_A), 3.79 (3H, s, OMe), 3.76 (3H, s, OMe), 2.86 – 2.80 (2H, m, C6-H_B + C7-H_A), 2.69 (1H, dd, *J* = 13.8, 6.8 Hz, C7-H_B), 2.56 – 2.43 (1H, m, C3-H), 1.88 – 1.73 (1H, m, C5-H_A), 1.57 (1H, tdd, *J* = 12.9, 8.7, 4.4 Hz, C4-H_A), 1.52 – 1.46 (1H, m, C4-H_B), 1.39 (1H, dd, *J* = 13.5, 3.7 Hz, C5-H_B). **^{13}C NMR (126 MHz, $CDCl_3$) δ** 158.2 (C Ar), 157.4 (CO), 139.4 (C Ar), 132.6 (C Ar), 131.8 (2 × C Ar), 130.2 (2 × C Ar), 128.5 (2 × C Ar), 120.6 (C Ar), 114.0 (2 × C Ar), 56.4 (C2), 55.4 (OMe), 53.0 (OMe), 40.3 (C6), 38.5 (C3), 37.3 (C7), 23.4 (C4), 20.1 (C5). **HRMS** (ESI): calculated for $C_{21}H_{25}O_3N^{79}Br$ $[M+H]^+$ requires m/z 418.1012, found m/z 418.1012. **IR** (film) ν_{max}: 2941, 1696, 1512, 1488, 1443, 1409, 1247, 1179, 1127, 1037 cm^{-1}.

(5m): (±)-Methyl (2*R*,3*R*)-3-benzhydryl-2-((*E*)-4-methoxystyryl)piperidine-1-carboxylate and (±)-Methyl (2*R*,3*S*)-3-benzhydryl-2-((*E*)-4-methoxystyryl)piperidine-1-carboxylate

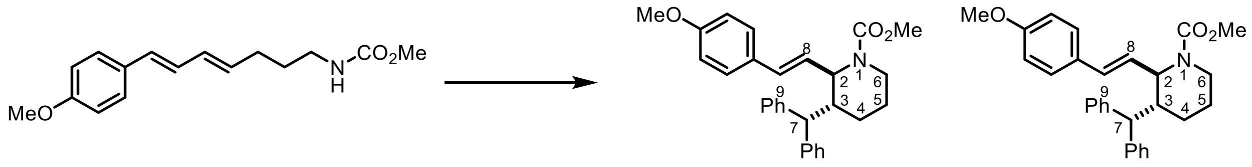

4d **A** **5m** d.r. (C2-C3): 5:1 **A**

Prepared according to **General Procedure K** using **4d** (10 mg, 0.040 mmol) and benzhydrol (7.5 mg, 0.040 mmol) at 0 °C for 48 h. Flash column chromatography (gradient elution 0% → 25% EtOAc – pentane) afforded **5m** as a colourless oil (12 mg, 65%).

Characteristic data for **5m-A,** 1.5:1 mixture of rotamers *A:B* (from the mixture): **^{1}H NMR (500 MHz, $CDCl_3$) δ** 7.35 – 7.11 (12H, m, Ar), 6.92 – 6.85 (2H, m, Ar), 6.24 (1H, dt, *J* = 16.2, 8.2 Hz, C8-H, *A*+*B*), 5.99 (1H, d, *J* = 15.7 Hz, C9-H, *A*), 5.91 (1H, d, *J* = 15.7 Hz, C9-H, *B*), 4.93 (1H, *br.* s, C2-H, *A*), 4.75 (1H, *br.* s, C2-H, *B*), 4.17 – 4.07 (1H, m, C6-H_A, *A*), 4.01 – 3.94 (1H, m, C6-H_A, *B*), 3.83 (3H, s, OMe), 3.67 (1H, s, OMe, *A*), 3.64 (1H, s, OMe, *B*), 3.54 (1H, *br.* s, C7-H, *A*), 3.52 (1H, *br.* s, C7-H, *B*), 3.01 (1H, *ap.* q, *J* = 13.7 Hz, C6-H_B, *A*+*B*), 2.64 – 2.52 (1H, m, C3-H, *A*+*B*), 1.78 – 1.62 (1H, m, C5-H_A, *A*+*B*), 1.55 – 1.40 (2H, m, C4-H_A + C5-H_B, *A*+*B*), 1.40 – 1.29 (1H, m, C4-H_B, *A*+*B*). **^{13}C NMR (126 MHz, $CDCl_3$) δ** 156.0 (CO, *A*+*B*), 134.6 (C9, *A*), 133.9 (C9, *B*), 119.6 (C8, *B*), 119.1 (C8, *A*), 55.8 (C2, *A*+*B*), 55.6 (C7, *A*+*B*), 55.5 (OMe, *A*), 55.4 (OMe, *B*), 52.8 (OMe, *B*), 52.6 (OMe, *A*), 43.4 (C3, *B*), 43.0 (C3, *A*), 40.0 (C6, *A*), 39.7 (C6, *B*), 26.0 (C5, *A*), 25.7 (C5, *B*), 24.9 (C4, *A*), 24.8 (C4, *B*).

Characteristic data for **5m-B** (from the mixture): **^{1}H NMR (500 MHz, $CDCl_3$) δ** 6.11 (1H, dd, *J* = 4.9 Hz, C9-H), 4.74 (1H, *br.* s, C2-H), 4.13 (1H, d, *J* = 12.1 Hz, C8-H), 3.83 (3H, s, OMe), 2.72 (1H, *ap.* d, *J* = 12.2 Hz, C3-H). **^{13}C NMR (126 MHz, $CDCl_3$) δ** 156.4 (CO), 129.9 (C9), 129.8 (C8), 55.4 (OMe), 54.3 (OMe), 52.1 (C7), 41.8 (C3).

Data for **5m**: **R_f** 0.33 (20% EtOAc – pentane). **HRMS** (ESI): calculated for $C_{29}H_{31}O_3NNa$ $[M+Na]^+$ requires m/z 464.2196, found m/z 464.2193. **IR** (film) ν_{max}: 3027, 2953, 2858, 1695, 1607, 1511, 1445, 1250, 1032, 706 cm^{-1}. Aromatic signals for **5m-A+B** (from the mixture): **^{13}C NMR (126 MHz, $CDCl_3$)**

δ 159.4, 159.3, 143.8, 143.7, 143.4, 142.5, 142.2, 128.9, 128.8, 128.8, 128.4, 128.1, 128.1, 127.8, 127.7, 127.6, 126.6, 126.6, 126.4, 126.4, 114.2, 114.1, 114.1.

(5n'): 2-(4-Methoxyphenyl)-1-((2-nitrophenyl)sulfonyl)piperidin-3-yl)methanol

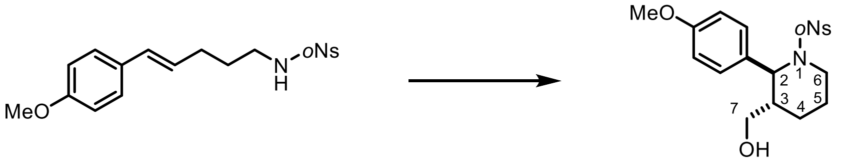

4-oNs **5nNs**

Prepared according to **General Procedure K** using **4-*o*Ns** (30 mg, 0.080 mmol) and paraformaldehyde (48 mg, 1.6 mmol, 20.0 equiv.) at 0 °C for 48 h. Flash column chromatography (gradient elution 0% → 25% EtOAc – pentane) afforded **5n-Ns** as a colourless oil (19 mg, 61%).

Data for **5n-Ns**: **R_f** 0.23 (20% EtOAc – pentane). **^{1}H NMR (400 MHz, $CDCl_3$) δ** 7.90 – 7.80 (1H, m, Ar), 7.70 – 7.61 (2H, m, Ar), 7.50 (1H, ddd, *J* = 7.9, 6.5, 2.3 Hz, Ar), 7.06 (2H, d, *J* = 9.0 Hz, Ar), 6.69 (2H, d, *J* = 8.8 Hz, Ar), 5.18 (1H, d, *J* = 3.0 Hz, C2-H), 4.02 – 3.90 (2H, m, C6-H_A + C7-H_A), 3.73 (3H, s, OMe), 3.60 (1H, ddd, *J* = 11.5, 6.5, 5.2 Hz, C7-H_B), 3.37 (1H, ddd, *J* = 13.7, 11.8, 3.2 Hz, C6-H_B), 2.42 (1H, dt, *J* = 8.7, 4.6 Hz, C3-H), 2.20 (1H, t, *J* = 6.2 Hz, *O*H), 1.76 (1H, dddd, *J* = 16.2, 11.9, 8.5, 4.6 Hz, C5-H_A), 1.64 (1H, ddt, J = 13.3, 9.9, 4.7 Hz, C5-H_B), 1.58 – 1.43 (2H, m, C4-H_2). **^{13}C NMR (101 MHz, $CDCl_3$) δ** 158.6 (C Ar), 147.5 (C Ar), 134.5 (C Ar), 133.3 (C Ar), 131.9 (C Ar), 130.9 (C Ar), 129.9 (C Ar), 128.1 (2 × C Ar), 124.1 (C Ar), 114.0 (2 × C Ar), 62.6 (C7), 56.7 (C2), 55.4 (OMe), 43.3 (C6), 40.2 (C3), 21.7 (C4), 21.3 (C5). **HRMS** (ESI): calculated for $C_{19}H_{23}O_6N_2S$ $[M+H]^+$ requires m/z 407.1271, found m/z 407.1271. **IR** (film) ν_{max}: 3423, 2936, 1612, 1584, 1542, 1513, 1463, 1441, 1371, 1250 cm^{-1}.

Product Derivatisations

(6a): (±)-(2*S*,3*S*)-3-(4-Methoxybenzyl)-2-(4-methoxyphenyl)pyrrolidine

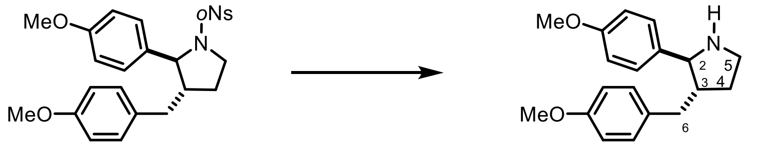

3g **6a**

Prepared according to **General Procedure L** using **3g** (34 mg, 0.070 mmol), LiOH (7.0 mg, 0.28 mmol) and thioglycolic acid (0.0097 mL, 0.14 mmol). Flash column chromatography (gradient elution 10% → 15% MeOH – CH_2Cl_2) afforded **6a** as a colourless oil (15 mg, 70%).

Data for **6a**: **R_f** 0.30 (15% MeOH – CH_2Cl_2). **^{1}H NMR (500 MHz, $CDCl_3$) δ** 7.36 (2H, d, *J* = 8.7 Hz, Ar), 6.95 (2H, d, *J* = 8.7 Hz, Ar), 6.87 (2H, d, *J* = 8.7 Hz, Ar), 6.77 (2H, d, *J* = 8.7 Hz, Ar), 3.85 (1H, d, *J* = 9.9 Hz, C2-H), 3.79 (3H, s, OMe), 3.77 (3H, s, OMe), 3.19 (1H, dt, *J* = 11.7, 8.2 Hz, C5-H_A), 3.05 (1H, td, *J* = 11.7, 10.4, 3.9 Hz, C5-H_B), 2.69 (1H, dd, *J* = 13.6, 4.1 Hz, C6-H_A), 2.40-2.49 (1H, m, C3-H), 2.36 (1H, dd, *J* = 13.5, 9.6 Hz, C6-H_B), 1.95-2.04 (1H, m, C4-H_A), 1.59-1.69 (1H, m, C4-H_B). **^{13}C NMR (125 MHz, $CDCl_3$) δ** 160.0 (C Ar), 158.3 (C Ar), 131.4 (C Ar), 129.9 (3 × C Ar), 129.5 (2 × C Ar), 114.4 (2 × C Ar), 114.0 (2 × C Ar), 67.6 (C2), 55.4 (OMe), 55.4 (OMe), 47.7 (C3), 44.2 (C5), 36.8 (C6), 30.3 (C4). **HRMS** (ESl): calculated for $C_{19}H_{24}O_2N$ $[M+H]^+$ requires *m/z* 298.1807, found *m/z* 298.1801. **IR** (film) ν_{max}: 2980,2888, 2360, 1738, 1612, 1512 cm^{-1}.

(6b): (±)-(2*S*,3*S*)-3-(4-Methoxybenzyl)-2-(4-methoxyphenyl)pyrrolidine

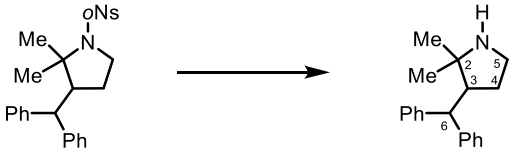

3v **6b**

Prepared according to **General Procedure L** using **3v** (42 mg, 0.093 mmol), LiOH (7.0 mg, 0.28 mmol) and thioglycolic acid (0.0097 mL, 0.14 mmol). Flash column chromatography (gradient elution 5% → 10% MeOH – CH_2Cl_2) afforded **6b** as a colourless oil (15 mg, 70%).

Data for **6b**: **R_f** 0.40 (15% MeOH – CH_2Cl_2). **^{1}H NMR (500 MHz, $CDCl_3$) δ** 7.33 – 7.38 (4H, m, Ar), 7.20 – 7.26 (4H, m, Ar), 7.09 – 7.16 (2H, m, Ar), 3.68 (1H, d, *J* = 11.7 Hz, C6-H), 2.87 (1H, dd, *J* = 8.4, 6.4 Hz, C5-H_2), 2.66 (1H, ddd, *J* = 11.7, 10.7, 8.4 Hz, C3-H), 1.85 (1H, ddt, *J* = 13.0, 8.4, 6.4 Hz, C4-H_A), 1.46 (1H, ddt, *J* = 13.2, 10.7, 8.4 Hz, C4-H_B), 1.05 (3H, s, Me), 0.70 (3H, s, Me). **^{13}C NMR (125 MHz, $CDCl_3$) δ** 145.7 (C Ar), 144.3 (C Ar), 128.7 (2 × C Ar), 128.6 (2 × C Ar), 128.3 (2 × C Ar), 127.8 (2 × C Ar), 126.5 (C Ar), 126.2 (C Ar), 60.6 (C2), 55.1 (C6), 52.7 (C3), 42.3 (C5), 33.2 (C4),

29.7 (Me), 22.3 (Me). **HRMS** (ESl): calculated for $C_{19}H_{23}N$ $[M+H]^+$ requires *m/z* 266.1909, found *m/z* 266.1904. **IR** (film) ν_{max}: 2980, 2886, 2360, 1738, 1451, 1380 cm^{-1}.

(6c): (±)- (2*S*,3*S*)-3-Cinnamyl-2-(4-methoxyphenyl)pyrrolidine

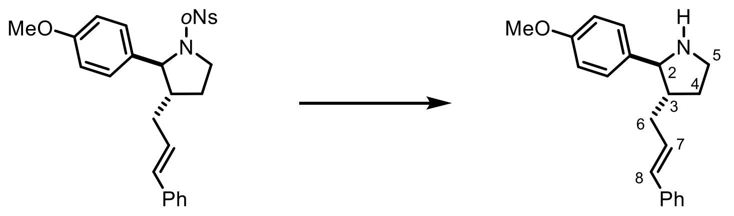

3h **6c**

Prepared according to **General Procedure L** using **3h** (43 mg, 0.089 mmol), LiOH (8.5 mg, 0.36 mmol) and thioglycolic acid (0.012 mL, 0.18 mmol). Flash column chromatography (gradient elution 3% → 6% MeOH – CH_2Cl_2) afforded **6c** as a colourless oil (18 mg, 67%).

Data for **6c**: $\mathbf{R_f}$ 0.40 (20% MeOH – CH_2Cl_2). **^{1}H NMR (500 MHz, $CDCl_3$) δ** 7.23 – 7.31 (6H, m, Ar), 7.15 – 7.21 (1H, m, Ar), 6.87 (2H, d, *J* = 8.7 Hz, Ar), 6.36 (1H, dt, *J* = 15.7, 1.4 Hz, C8-H), 6.11 (1H, dt, *J* = 15.8, 7.1 Hz, C7-H), 3.80 (3H, s, OMe), 3.67 (1H, d, *J* = 8.0 Hz, C2-H), 3.22 (1H, ddd, *J* = 10.1, 7.5, 6.1 Hz, C5-H_A), 3.07 (1H, ddd, *J* = 10.1, 8.5, 5.4 Hz, C5-H_B), 2.38 (1H, dddd, *J* = 13.5, 6.6, 4.8, 1.4 Hz, C6-H_A), 2.10 – 2.21 (2H, m, 1 × C4-H_A + 1 × C6-H_B), 2.03 – 2.10 (1H, m, C3-H), 1.64 (1H, dddd, *J* = 11.8, 8.5, 7.5, 6.1 Hz, C4-H_B). **^{13}C NMR (125 MHz, $CDCl_3$) δ** 158.9 (C Ar), 137.8 (C Ar), 135.8 (C Ar), 131.0 (C-8), 129.3 (C7), 128.6 (2 × C Ar), 128.5 (2 × C Ar), 127.0 (C Ar), 126.1 (2 × Ar), 114.0 (2 × C Ar), 68.3 (C2), 55.4 (OMe), 47.7 (C3), 46.0 (C5), 36.5 (C6), 32.0 (C4). **HRMS** (ESl): calculated for $C_{20}H_{24}ON$ $[M+H]^+$ requires *m/z* 294.1858, found *m/z* 294.1852. **IR** (film) ν_{max}: 2980, 2360, 1764, 1461, 1382, 1251 cm^{-1}.

(6d): (±)-(2*S*,3*R*)-2-(4-Methoxyphenyl)-3-[(1'*R*)-3-phenylcyclopent-2-en-1-yl]pyrrolidine

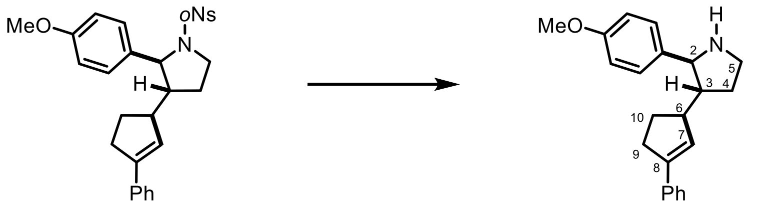

3q **6d**

Prepared according to **General Procedure L** using **3q** (27 mg, 0.053 mmol), LiOH (5.0 mg, 0.21 mmol) and thioglycolic acid (0.008 mL, 0.11 mmol). Flash column chromatography (gradient elution 1% → 15% MeOH – CH_2Cl_2) afforded **6d** as a colourless oil (10 mg, 60%).

Data for **6d**: $\mathbf{R_f}$ 0.40 (15% MeOH – CH_2Cl_2). **^{1}H NMR (500 MHz, $CDCl_3$) δ** 7.37 – 7.40 (2H, m, Ar), 7.35-7.37 (4H, m, Ar), 7.20 – 7.24 (1H, m, Ar), 6.86 (2H, d, *J* = 8.7 Hz, Ar), 3.89 (1H, d, *J* = 9.0 Hz,

C2-H), 3.79 (3H, s, OMe), 3.23 (1H, dt, J = 10.5, 7.3 Hz, C5-H_A), 3.03 (1H, ddd, J = 10.5, 8.9, 5.2 Hz, C5-H_B), 2.88 – 2.95 (1H, m, C6-H), 2.55 – 2.68 (2H, m, C9-H_2), 2.19 – 2.27 (1H, m, C3-H), 2.13 (1H, dtd, J = 12.6, 8.0, 5.2 Hz, C4-H_A), 1.98 – 2.06 (1H, m, C10-H_A), 1.75 (1H, dtd, J = 12.6, 8.9, 6.8 Hz, C4-H_B), 1.48 (1H, ddt, J = 13.0, 9.1, 7.1 Hz, C10-H_B). **^{13}C NMR (125 MHz, $CDCl_3$) δ** 159.3 (C Ar), 143.9 (C Ar), 136.5 (C Ar), 129.1 (2 × C Ar), 128.4 (2 × C Ar + C8), 127.4 (C Ar), 127.2 (C7), 125.8 (2 × C Ar), 114.1 (2 × C Ar), 67.0 (C2), 55.4 (OMe), 51.6 (C3), 48.2 (C6), 45.5 (C5), 32.9 (C9), 29.2 and 29.1 (C4 + C10). **NOESY-2D (500 MHz, $CDCl_3$)**: between C2-H and C6-H, between C2-H and C7-H, between C2-H and C10-H_A. **HRMS** (ESl): calculated for $C_{22}H_{26}ON$ $[M+H]^+$ requires m/z 320.2014, found m/z 320.2007. **IR** (film) ν_{max}: 2980, 2888, 2360, 1738, 1382, 1251 cm^{-1}.

(3k'): (±)-(2*S*,3*R*)-3-((*S*,*E*)-1,3-Diphenylallyl)-2-(4-methoxyphenyl)-*N*-(4-nitrophenyl)-pyrrolidine-1-carboxamide

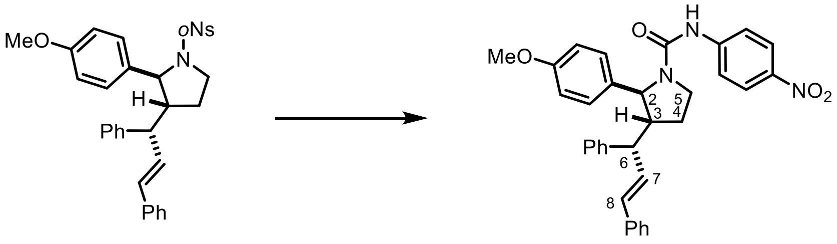

3k **3k'**

Prepared according to **General Procedure L** using **3k** (33 mg, 0.06 mmol), LiOH (4.5 mg, 0.18 mmol) and thioglycolic acid (0.008 mL, 0.088 mmol). The crude material was re-dissolved in CH_2Cl_2 (1 mL) and Et_3N (0.01 mL, 0.07 mmol) and 4-nitrophenylisocyanate (12 mg, 0.073 mmol) added. After 16 h the solvent was removed *in vacuo*. Flash column chromatography (gradient elution 1% → 5% MeOH – CH_2Cl_2) afforded **3k'** as a yellow solid (25 mg, 78%).

Data for **3k'**: **R_f** 0.40 (5% MeOH – CH_2Cl_2). **^{1}H NMR (400 MHz, $CDCl_3$) δ** 8.07 – 7.98 (3H, m, Ar), 7.36 – 7.14 (12H, m, Ar), 6.89 (2H, d, J = 8.7 Hz, Ar), 6.59 (1H, d, J = 9.0 Hz, Ar), 6.51 (1H, d, J = 15.7 Hz, C8-H), 6.46 (1H, *br.* s, *N*H), 6.21 (1H, dd, J = 15.7, 9.3 Hz, C7-H), 4.76 – 4.64 (1H, *br.* s, C2-H), 3.89 (1H, dt, J = 11.0, 7.3 Hz, C5-H_A), 3.79 (3H, s, OMe), 3.74 – 3.65 (1H, m, C5-H_B), 3.44 (1H, t, J = 9.8 Hz, C6-H), 2.76 – 2.59 (1H, m, C3-H), 1.97 (1H, dq, J = 14.1, 7.2 Hz, C4-H_A), 1.61 (1H, ddt, J = 13.0, 7.4, 5.5 Hz, C4-H_B). **^{13}C NMR (101 MHz, $CDCl_3$) δ** 159.7 (C Ar), 153.2 (CO), 145.3 (C Ar), 142.4 (C Ar), 142.3 (C Ar), 136.8 (C Ar), 132.1 (C7), 131.4 (C8), 129.1 (2 × C Ar), 128.7 (2 × C Ar), 128.0 (C Ar), 127.8 (2 × C Ar), 127.2 (C Ar), 126.5 (2 × C Ar), 126.4 (2 × C Ar), 125.1 (2 × C Ar), 118.0 (2 × C Ar), 115.0 (2 × C Ar), 113.5 (C Ar), 65.1 (C2), 55.5 (OMe), 55.5 (C3), 53.3 (C6), 46.4 (C5), 27.0 (C4). **HRMS** (ESI): calculated for $C_{33}H_{32}N_3O_4$ $[M+H]^+$ requires m/z 534.2387, found m/z 534.2388. **IR** (film) ν_{max}: 3655, 2981, 2918, 2850, 2361, 2341, 1380, 1250, 1137 cm^{-1}. **mp** (240 – 242 °C, EtOAc).

(7a): (±)-(2*S*,3*R*)-3-Benzhydryl-2-(4-methoxyphenyl)piperidin-1-ium iodide

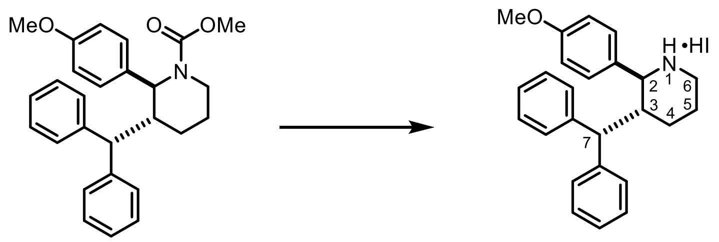

5a **7a**

Prepared according to **General Procedure M** using **5a** (37 mg, 0.089 mmol), trimethylsilyl iodide (0.038 mL, 0.27 mmol) in $CHCl_3$ (3.0 mL) at 55 °C for 2 h. Flash column chromatography (gradient elution 0% → 5% MeOH – CH_2Cl_2) afforded **7a** as an off white waxy solid (39 mg, 86%).

Data for **7a**: $\mathbf{R_f}$ 0.64 (10% MeOH – CH_2Cl_2). **^{1}H NMR (600 MHz, $CDCl_3$) δ** 8.75 – 8.41 (2H, m, Ar), 7.48 (2H, d, *J* = 8.2 Hz, Ar), 7.29 – 7.24 (3H, m, Ar), 7.22 (2H, t, *J* = 7.7 Hz, Ar), 7.17 – 7.12 (1H, m, Ar), 7.09 (2H, d, *J* = 8.3 Hz, Ar), 6.93 – 6.88 (2H, m, Ar), 6.87 – 6.79 (2H, m, Ar), 3.95 (1H, d, *J* = 3.7 Hz, C7-H), 3.77 (3H, s, OMe), 3.69 (1H, d, *J* = 11.2 Hz, C2-H), 3.17 (1H, tt, *J* = 11.5, 3.4 Hz, C3-H), 2.91 – 2.78 (1H, m, C6-H_A), 2.52 (1H, t, *J* = 12.7 Hz, C6-H_B), 2.18 – 2.00 (2H, m, C4-H_A + C5-H_A), 1.72 (1H, dt, *J* = 14.5, 2.9 Hz, C5-H_B), 1.36 (1H, qd, *J* = 13.1, 3.5 Hz, C4-H_B). **^{13}C NMR (101 MHz, $CDCl_3$) δ** 160.0 (C Ar), 142.6 (C Ar), 140.4 (C Ar), 130.7 (C Ar), 130.6 (2 × C Ar), 128.4 (4 × C Ar), 128.2 (4 × C Ar), 127.0 (C Ar), 126.2 (C Ar), 114.4 (2 × C Ar), 64.7 (C7), 55.4 (OMe), 50.6 (C2), 46.0 (C6), 43.0 (C3), 26.4 (C5), 23.7 (C4). **NOESY-2D (600 MHz, $CDCl_3$)**: between C2-H and C6-H_B, between C2-H and C4-H_B, between C3-H and C5-H_A. **HRMS** (ESI): calculated for $C_{25}H_{28}ON$ $[M+H]^+$ requires m/z 358.2165, found m/z 358.2162. **IR** (film) ν_{max}: 2981, 2930, 2360, 2341, 1611, 1515, 1451, 1255, 1181, 1302 cm^{-1}.

(7b): (±)-(2*S*,3*R*)-3-(4-Methoxybenzyl)-2-(4-methoxyphenyl)piperidin-1-ium iodide

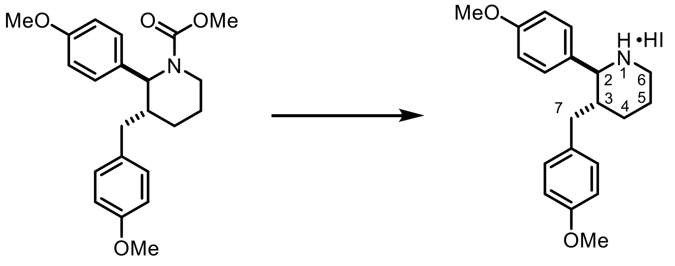

5b **7b**

Prepared according to **General Procedure M** using **5b** (38 mg, 0.10 mmol), trimethylsilyl iodide (0.044 mL, 0.30 mmol) in $CHCl_3$ (3.0 mL) at 55 °C for 2 h. Flash column chromatography (gradient elution 0% → 5% MeOH – CH_2Cl_2) afforded **7b** as an off white waxy solid (42 mg, 93%).

Data for **7b**: **R_f** 0.47 (10% MeOH – CH_2Cl_2). **^{1}H NMR (400 MHz, MeOD) δ** 7.47 (2H, d, *J* = 8.7 Hz, Ar), 7.06 (2H, d, *J* = 8.8 Hz, Ar), 6.92 (2H, d, *J* = 8.6 Hz, Ar), 6.79 (2H, d, *J* = 8.7 Hz, Ar), 3.93 (2H, d, *J* = 10.8 Hz, C2-H), 3.84 (3H, s, OMe), 3.74 (3H, s, OMe), 3.46 – 3.28 (1H, m, C6-H_A), 3.10 (1H, td, *J* = 12.9, 3.3 Hz, C6-H_B), 2.43 (1H, dd, *J* = 13.0, 2.8 Hz, C7-H_A), 2.26 – 2.04 (1H, m, C7-H_B), 1.99 – 1.83 (2H, m, 1 × C4-H_A + 1 × C5-H_A), 1.74 (1H, qt, *J* = 14.2, 4.0 Hz, C4-H_B), 1.44 – 1.25 (1H, m, C5-H_B). **^{13}C NMR (101 MHz, MeOD) δ** 162.1 (C Ar), 159.7 (C Ar), 131.8 (C Ar), 131.1 (2 × C Ar), 130.7 (2 × C Ar), 129.0 (C Ar), 115.9 (2 × C Ar), 114.9 (2 × C Ar), 66.4 (C2), 55.9 (OMe), 55.7 (OMe), 46.6 (C6), 42.6 (C3), 38.6 (C7), 29.5 (C5), 23.8 (C4). **HRMS** (ESI): calculated for $C_{20}H_{26}O_2N$ $[M+H]^+$ requires m/z 312.1958, found m/z 312.1957. **IR** (film) ν_{max}: 2935, 17096, 1692, 1676, 1586, 1513, 1349, 1363, 1247, 1198 cm^{-1}.

(7c): (±)-(2*S*,3*R*)-3-Cinnamyl-2-(4-methoxyphenyl)piperidin-1-ium iodide

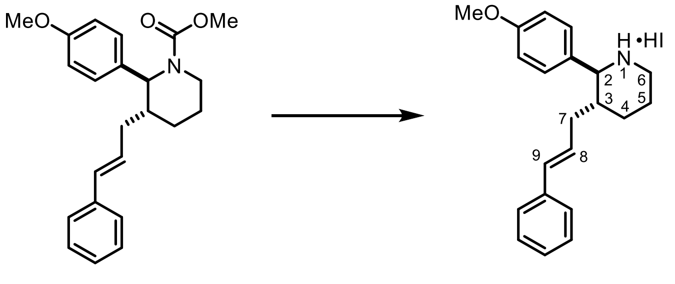

5c **7c**

Prepared according to **General Procedure M** using **5c** (46 mg, 0.13 mmol), trimethylsilyl iodide (0.050 mL, 0.39 mmol), isoprene (0.13 mL, 1.3 mmol) in $CHCl_3$ (3.0 mL) at 55 °C for 2 h. Flash column chromatography (gradient elution 0% → 5% MeOH – CH_2Cl_2) afforded **7c** as a white waxy solid (44 mg, 80%).

Data for **7c**: **R$_f$** 0.51 (10% MeOH – CH_2Cl_2). **^{1}H NMR (400 MHz, MeOD) δ** 7.44 (2H, d, *J* = 8.7 Hz, Ar), 7.31 – 7.22 (4H, m, Ar), 7.20 – 7.14 (1H, m, Ar), 7.04 (1H, d, *J* = 8.8 Hz, Ar), 6.20 (1H, d, *J* = 15.8 Hz, C9-H), 6.03 (1H, ddd, *J* = 15.7, 8.1, 6.5 Hz, C8-H), 3.98 (1H, d, *J* = 11.2 Hz, C2-H), 3.81 (3H, s, OMe), 3.39 (1H, ddd, *J* = 12.6, 3.9, 1.8 Hz, C6-H_A), 3.15 (1H, td, *J* = 13.0, 3.4 Hz, C6-H_B), 2.26 – 2.04 (3H, m, 1 × C3-H + 1 × C5-H_A + 1 × C7-H_A), 2.04 – 1.93 (3H, m, 2 × C4-H_2 + 1 × C7-H_B), 1.93 – 1.82 (1H, m, C5-H_B). **^{13}C NMR (101 MHz, MeOD) δ** 162.3 (C Ar), 138.6 (C Ar), 134.0 (C9), 130.7 (2 × C Ar), 129.5 (2 × C Ar), 128.3 (C Ar), 128.2 (C Ar), 127.0 (2 × C Ar), 126.9 (C8), 115.9 (2 × C Ar), 66.0 (C2), 55.9 (OMe), 46.4 (C6), 40.7 (C3), 36.9 (C7), 29.8 (C5), 23.6 (C4). **HRMS** (ESI): calculated for $C_{21}H_{26}ON$ $[M+H]^+$ requires m/z 308.2009, found m/z 308.2012. **IR** (film) ν_{max}: 3024, 2957, 2934, 2837, 2069, 1612, 1516, 1256, 1182, 1030, 968, 826, 746 cm^{-1}.

(8): (±)-Methyl (2*S*,3*R*)-3-benzhydryl-1-((2-nitrophenyl)sulfonyl)pyrrolidine-2-carboxylate

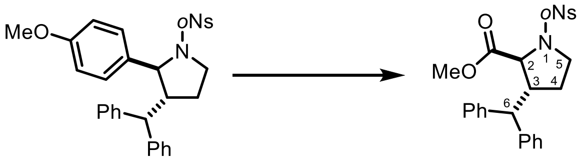

3c **8**

To a solution of **3c** (55 mg, 0.10 mmol) in a 2:2:3 mixture of CCl_4:MeCN:pH 7 buffer (Na_2HPO_4) (1.0 mL) was added $NaIO_4$ (445 mg, 2.08 mmol) at 0 °C. After 15 min $RuCl_3$ (1.0 mg, 5.0 mol%) was added and the reaction mixture allowed to warm up to rt before being stirred vigorously for 16 h. The reaction mixture was diluted with Et_2O and 1.0 M HCl. The aqueous later was extracted with Et_2O and the combined organic layers were washed with 1.0 M HCl and dried over $MgSO_4$ before concentrating *in vacuo*. The crude residue was re-dissolved in a 9:1 PhMe:MeOH mixture (1.0 mL) and trimethylsilyldiazomethane solution (2.0 M in Et_2O) was added dropwise until the yellow colour persisted. After 15 min the solvent was removed *in vacuo*. Flash column chromatography (gradient elution 10% → 25% EtOAc – pentane) afforded **8** as a colourless oil (25 mg, 52%).

Data for **8**: **R$_f$** 0.27 (30% EtOAc – pentane). **^{1}H NMR (600 MHz, $CDCl_3$) δ** 7.99 (1H, dd, *J* = 7.9, 1.4 Hz, Ar), 7.73 – 7.67 (1H, m, Ar), 7.67 – 7.58 (2H, m, Ar), 7.28 – 7.23 (6H, m, Ar), 7.22 – 7.15 (4H, m, Ar), 4.27 (1H, d, *J* = 2.4 Hz, C2-H), 3.83 (1H, dt, *J* = 9.4, 7.7 Hz, C5-H_A), 3.71 (1H, d, *J* = 12.0 Hz, C6-H), 3.70 – 3.63 (1H, m, C5-H_B), 3.52 (3H, s, OMe), 3.31 (1H, ddt, *J* = 12.2, 6.5, 3.0 Hz, C3-H), 2.07 (1H, dtd, *J* = 16.3, 8.2, 5.7 Hz, C4-H_A), 1.71 (1H, ddt, *J* = 12.6, 7.8, 4.1 Hz, C4-H_B). **^{13}C NMR (151 MHz, $CDCl_3$) δ** 172.2 (CO), 148.2 (C Ar), 142.6 (C Ar), 142.4 (C Ar), 133.6 (C Ar), 133.1 (C Ar), 131.8 (C Ar), 131.2 (C Ar), 129.0 (4 × C Ar), 128.1 (2 × C Ar), 128.0 (2 × C Ar), 127.1 (C Ar), 127.0 (C Ar), 124.2 (C Ar), 64.5 (C2), 54.1 (C6), 52.6 (OMe), 48.4 (C3), 47.6 (C5), 28.6 (C4). **HRMS** (ESI): calculated for $C_{25}H_{25}N_2O_6S$ $[M+H]^+$ requires *m/z* 481.1429, found *m/z* 481.1428. **IR** (film) ν_{max}: 2981, 2361, 2312, 1745, 1544, 1453, 1372, 1215, 1032 cm^{-1}.

(*trans*-9): (±)-2-{(2*S*,3*R*)-3-Benzhydryl-1-[(2-nitrophenyl)sulfonyl]pyrrolidin-2-yl}methanol

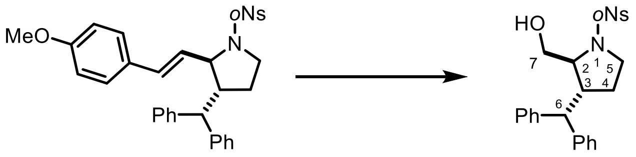

trans-**3x** *trans*-**9**

O_3 was bubbled through a solution of **3x** (26 mg, 0.048 mmol) in a 2:1 mixture of CH_2Cl_2:MeOH (0.5 mL) at -78 °C for 10 min (at which point a blue colour was observed) before bubbling with O_2 for a further 3 min and then N_2 for a further 3 min (at which point no blue colour was observed). $NaBH_4$ (0.5 mg, 0.1 mmol) was added and the reaction mixture left to warm to rt before concentrating *in vacuo.* Flash column chromatography (gradient elution 10% → 20% acetone – pentane) afforded *trans*-**9** as a colourless oil (10 mg, 45%).

Data for *trans*-**9**: **R_f** 0.30 (35% acetone – pentane). **^{1}H NMR (500 MHz, $CDCl_3$) δ** 7.98 (1H, dd, *J* = 8.2, 1.4 Hz, Ar), 7.73 – 7.79 (1H, m, Ar), 7.62 – 7.68 (2H, m, Ar), 7.10 – 7.25 (8H, m, Ar), 7.00 – 7.05 (2H, m, Ar), 3.72 – 3.77 (1H, m, C5-H_A), 6.67 – 3.72 (1H, m, C2-H), 3.56 (1H, ddd, *J* = 11.5, 6.5, 4.6 Hz, C7-H_A), 3.44 – 3.51 (2H, m, 1 × C5-H_B + 1 × C6-H), 3.36 –3.44 (1H, m, C7-H_B), 2.10 (1H, t, *J* = 6.5 Hz, *O*H), 1.98 – 2.07 (1H, m, C4-H_A), 1.49 – 1.57 (1H, m, C4-H_B). **^{13}C NMR (125 MHz, $CDCl_3$) δ** 143.0 (C Ar), 142.8 (C Ar), 134.0 (C Ar), 131.9 (C Ar), 131.8 (C Ar), 131.5 (C Ar), 129.0 (2 × C Ar), 128.9 (2 × C Ar), 128.1 (C Ar), 128.0 (2 × C Ar), 127.9 (2 × C Ar), 126.9 (C Ar), 126.8 (C Ar), 124.2 (C Ar), 65.5 (C2), 65.1 (C7), 54.6 (C6), 48.4 (C5), 45.7 (C3), 29.0 (C4). **HRMS** (ESl): calculated for $C_{24}H_{24}N_2O_5S$ $[M+H]^+$ requires *m/z* 475.1304, found *m/z* 475.1295. **IR** (film) ν_{max}: 3026, 2934, 1705, 1543, 1371, 1335 cm^{-1}.

(*cis*-9): (±)-2-{(2*S*,3*S*)-3-Benzhydryl-1-[(2-nitrophenyl)sulfonyl]pyrrolidin-2-yl}methanol

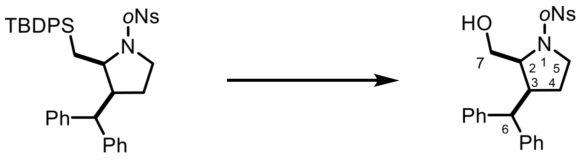

cis-**3ac** *cis*-**9**

To a solution of **3ac** (29 mg, 0.045 mmol) in anhydrous CH_2Cl_2 (0.45 mL) was added $BF_3{\cdot}2AcOH$ (0.03 mL, 0.22 mmol) before heating to reflux for 24 h. After cooling to rt aq. $NaHCO_3$ was added and the layers separated. The aqueous later was extracted with CH_2Cl_2 and the combined organic layers were washed with brine and dried over Na_2SO_4 before concentrating *in vacuo*. The crude material was re-dissolved in a 1:1 THF:MeOH mixture (0.60 mL) before addition of $NaHCO_3$ (4 mg, 0.05 mmol), KF (8.0 mg, 0.15 mmol) and H_2O_2 (30%, 0.42 mL, 0.44 mmol). The reaction mixture was heated to reflux for 6 h, cooled to rt and aq. $NaHCO_3$ added. The aqueous later was extracted with EtOAc and the

combined organic layers were washed with brine and dried over Na_2SO_4 before concentrating *in vacuo*. Flash column chromatography (gradient elution 10% → 20% acetone – pentane) afforded *cis*-**9** as a colourless oil (9 mg, 55%).

Data for *cis*-**9**: $\mathbf{R_f}$ 0.40 (35% acetone – pentane). **^{1}H NMR (500 MHz, $CDCl_3$) δ** 8.09 (1H, dd, *J* = 7.8, 1.5 Hz, Ar), 7.68 – 7.77 (2H, m, Ar), 7.67 (1H, dd, *J* = 7.8, 1.5 Hz, Ar), 7.34 – 7.38 (2H, m, Ar), 7.28 – 7.33 (2H, m, Ar), 7.17 – 7.22 (4H, m, Ar), 7.09 – 7.16 (2H, m, Ar), 4.08 (1H, dt, *J* = 7.4, 3.6 Hz, C2-H), 3.97 (1H, d, *J* = 11.9 Hz, C6-H), 3.65 (1H, ddd, *J* = 12.2, 5.3, 3.6 Hz, C7-H_A), 3.50 – 3.59 (2H, m, 1 × C7-H_B + 1 × C5-H_A), 3.30 (1H, td, *J* = 10.0, 7.3 Hz, C5-H_B), 3.01 – 3.10 (1H, m, C3-H), 1.98 – 2.10 (1H, m, C4-H_A), 1.93 (1H, dd, *J* = 7.7, 5.3 Hz, *O*H), 1.62 (1H, dt, *J* = 13.1, 7.3 Hz, C4-H_B). **^{13}C NMR (125 MHz, $CDCl_3$) δ** 143.9 (C Ar), 142.5 (C Ar), 134.0 (2 × C Ar), 131.9 (2 × C Ar), 131.7 (C Ar), 129.2 (2 × C Ar), 128.8 (2 × C Ar), 127.6 (4 × C Ar), 127.0 (C Ar), 126.7 (C Ar), 124.3 (C Ar), 63.2 (C2), 62.4 (C7), 52.2 (C6), 47.7 (C5), 46.5 (C3), 30.4 (C4). **HRMS** (ESl): calculated for $C_{24}H_{24}N_2O_5S$ $[M+H]^+$ requires *m/z* 475.1304, found *m/z* 475.1295. **IR** (film) ν_{max}: 3026, 2934, 1705, 1543, 1371, 1335 cm^{-1}.

Use of Tertiary Alcohols

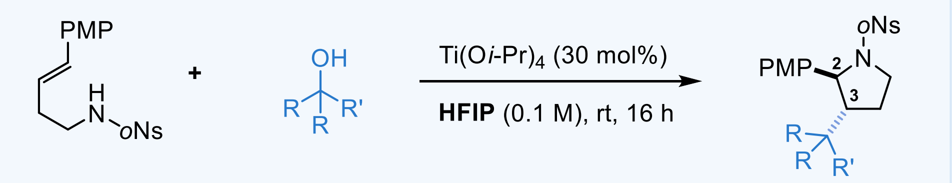

1c (1.0 equiv.) **2** (1.0 equiv.) **3**

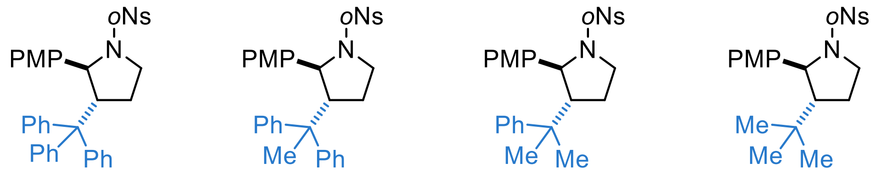

3ag, 0%; 0%[a] **3ah**, 0%; 13%[a] **3ai**, 33%[a] **3aj**, 0%, 0%[a]

[a] Performed at 70 °C

Tertiary alcohols were found to be partially compatible with the reaction conditions, however they gave diminished yields when compared to primary and secondary alcohols.

(3ah): (±)-(2*S*,3*R*)-3-(1,1-Diphenylethyl)-2-(4-methoxyphenyl)-1-((2-nitrophenyl)sulfonyl)-pyrrolidine

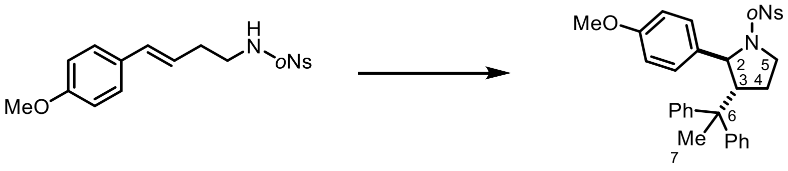

1c **3ah**

Prepared according to **General Procedure K** using **1c** (36 mg, 0.10 mmol) and 1,1-diphenylethanol (20 mg, 0.10 mmol) at 70 ˚C. Flash column chromatography (gradient elution 10% → 20% EtOAc – pentane) afforded **3ah** as a colourless oil (7 mg, 13%).

Data for **3ah**: **R$_f$** 0.35 (30% EtOAc – pentane). **^{1}H NMR (600 MHz, CDCl$_3$) δ** 7.45 – 7.38 (2H, m, Ar), 7.25 – 7.23 (2H, m, Ar), 7.22 – 7.17 (4H, m, Ar), 7.18 – 7.13 (5H, m, Ar), 7.10 (1H, ddd, *J* = 8.3, 7.2, 1.5 Hz, Ar), 6.51 (2H, d, *J* = 8.7 Hz, Ar), 6.41 (2H, d, *J* = 8.7 Hz, Ar), 4.73 (1H, d, *J* = 3.8 Hz, C2-H), 3.94 (1H, ddd, *J* = 9.9, 8.8, 4.1 Hz, C5-H$_A$), 3.70 – 3.67 (1H, m, C5-H$_B$), 3.66 (3H, s, OMe), 3.27 (1H, dt, *J* = 8.3, 3.8 Hz, C3-H), 2.53 (1H, dq, *J* = 13.6, 8.7 Hz, C4-H$_A$), 2.16 (1H, ddt, *J* = 13.4, 8.0, 4.0 Hz, C4-H$_B$), 1.76 (3H, s, C7-H$_3$). **^{13}C NMR (151 MHz, CDCl$_3$) δ** 158.8 (C Ar), 147.8 (C Ar), 147.8 (C Ar), 147.7 (C Ar), 135.0 (C Ar), 134.0 (C Ar), 132.7 (C Ar), 131.3 (C Ar), 131.0 (C Ar), 128.6 (2 × C Ar), 128.5 (2 × C Ar), 128.2 (3 × C Ar), 128.0 (2 × C Ar), 126.5 (C Ar), 126.4 (C Ar), 123.6 (2 × C Ar), 113.7 (2 × C Ar), 65.5 (C2), 57.6 (C3), 55.6 (OMe), 50.4 (C5), 50.1 (C6), 28.0 (C4), 23.0 (C7). **HRMS** (ESI): calculated for $C_{31}H_{30}N_2O_5SNa$ [M+Na]$^+$ requires *m/z* 565.1768, found *m/z* 565.1768. **IR** (film) ν_{max}: 2361, 1544, 1513, 1372, 1247, 1166, 1034, 912 cm^{-1}.

(3ai): (±)-(2*S*,3*R*)-2-(4-Methoxyphenyl)-1-((2-nitrophenyl)sulfonyl)-3-(2-phenylpropan-2-yl)pyrrolidine

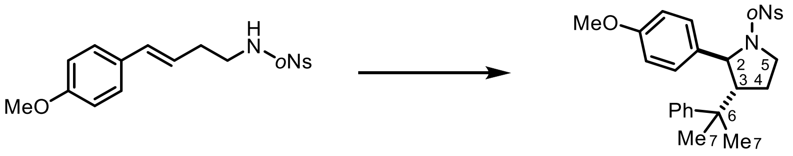

1c **3ai**

Prepared according to **General Procedure K** using **1c** (36 mg, 0.10 mmol) and 1-methyl-1-phenylethyl alcohol (13.5 mg, 0.10 mmol) at 70 ˚C. Flash column chromatography (gradient elution 10% → 20% EtOAc – pentane) afforded **3ai** as a colourless oil (16 mg, 33%).

Data for **3ai**: $\mathbf{R_f}$ 0.39 (30% EtOAc – pentane). **^{1}H NMR (600 MHz, CDCl$_3$) δ** 7.44 (1H, d, *J* = 7.6 Hz, Ar), 7.40 (1H, ddd, *J* = 7.9, 6.6, 2.3 Hz, Ar), 7.25 – 7.19 (4H, m, Ar), 7.16 – 7.09 (3H, m, Ar), 6.76 (2H, d, *J* = 8.5 Hz, Ar), 6.45 (2H, d, *J* = 8.5 Hz, Ar), 4.62 (1H, d, *J* = 6.6 Hz, C2-H), 3.89 (1H, ddd, *J* = 10.2, 7.8, 4.6 Hz, C5-H_A), 3.75 (1H, ddd, *J* = 10.1, 8.1, 7.0 Hz, C5-H_B), 3.68 (3H, s, OMe), 2.63 (1H, q, *J* = 7.5 Hz, C3-H), 1.98 – 1.90 (1H, m, C4-H_A), 1.90 – 1.85 (1H, m, C4-H_B), 1.31 (3H, s, C7-H_3), 1.27 (3H, s, C7-H_3). **^{13}C NMR (151 MHz, CDCl$_3$) δ** 158.7 (C Ar), 147.8 (C Ar), 147.4 (C Ar), 134.4 (C Ar), 134.2 (C Ar), 132.3 (C Ar), 130.9 (C Ar), 130.8 (C Ar), 128.7 (2 × C Ar), 128.3 (2 × C Ar), 126.3 (2 × C Ar), 126.2 (C Ar), 123.4 (C Ar), 113.5 (2 × C Ar), 65.1 (C2), 60.4 (C3), 55.4 (OMe), 49.8 (C5), 39.9 (C6), 27.4 (C4), 26.2 (C7), 25.9 (C7). **HRMS** (ESI): calculated for $C_{26}H_{29}N_2O_5S$ $[M+H]^+$ requires *m/z* 481.1792, found *m/z* 481.1794. **IR** (film) ν_{max}: 2363, 1545, 1513, 1373, 1248, 1165, 1033, 765, 653 cm^{-1}.

<u>Unreactive Nucleophiles</u>

Nucleophiles that were found to be unreactive with benzhydrol at elevated temperatures.

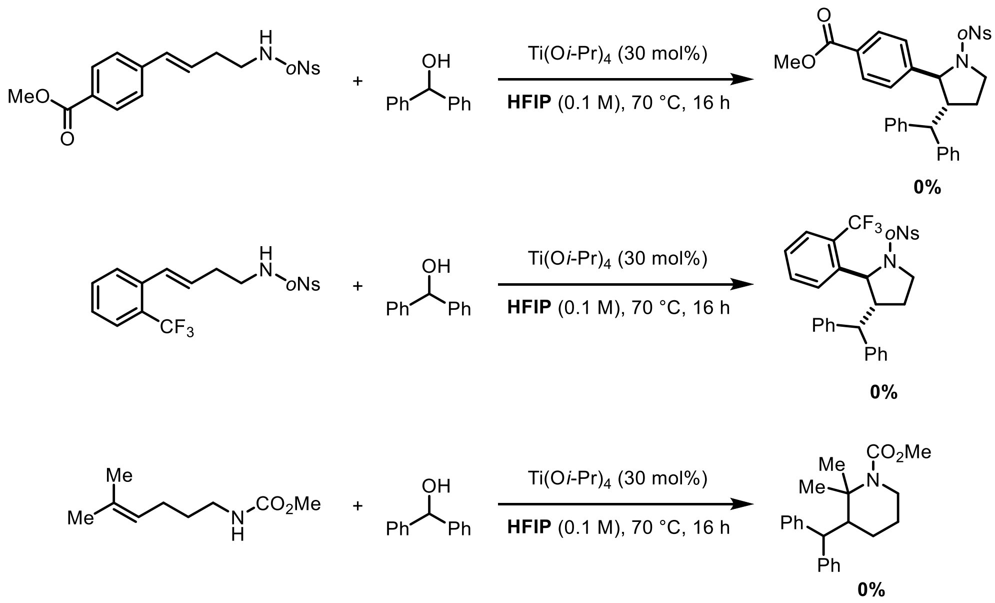

Supporting document for mechanistic hypothesis

a) Influence of alkene geometry in a parent system

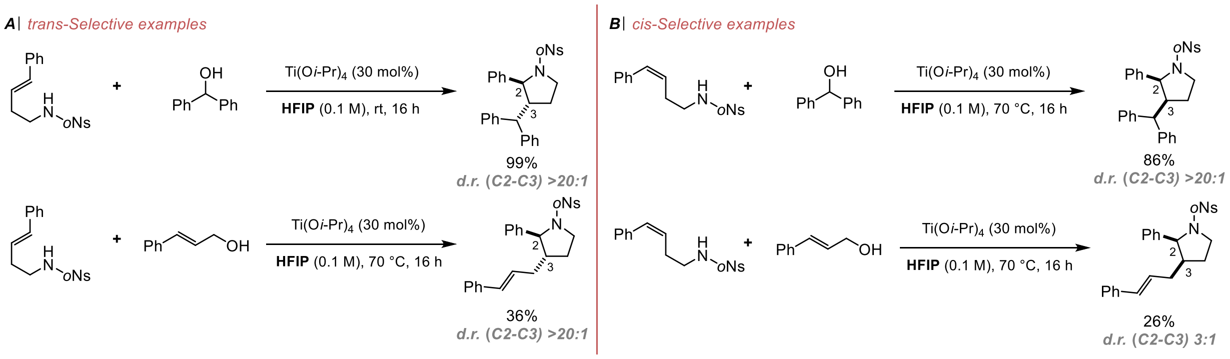

The role of the double bond geometry above shows that the *E* isomer gives exclusively the *trans* diastereomer with high stereoselectivity when reacted with benzhydrol and cinnamyl alcohol whereas the *Z* isomer leads to a major, but not exclusive, *cis* product when subjected to the same reaction suggesting a less stereoselective pathway.

b) Temperature experiments

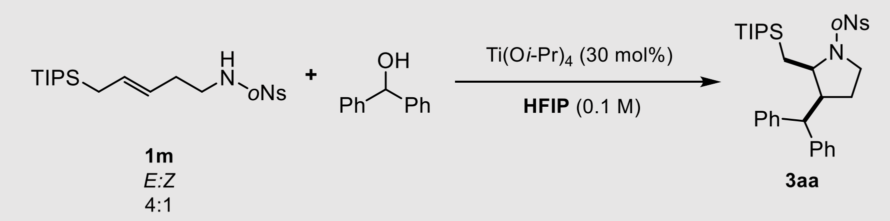

Entry	Temp / ˚C	Time / h	Yield **3aa** / %	*trans:cis*
1	0	16	99	*1:4*
2	70	16	99	*1:2*
3	70	24	99	*1:1.4*
4	70	40	99	*1:1.1*

Higher temperature experiments support the equilibration at high temperature between the *trans* and *cis* diastereomers of **3aa**, with the *cis*-diastereomer being the kinetic product whereas the *trans*-diastereomer is the thermodynamically more stable product.

c) Resubjection experiment

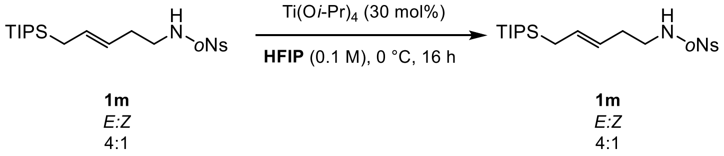

Finally, a control experiment confirmed that starting material **1m** was not simply being isomerised under the reaction conditions. Following 16 h at 0 ˚C the alkene geometry ratio was unchanged.

Supporting document for structural assignment of piperidines

Coupling constant analysis

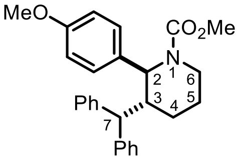

5a

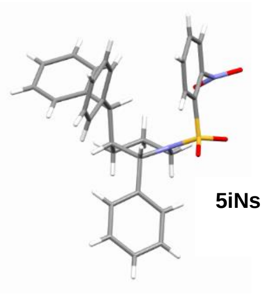

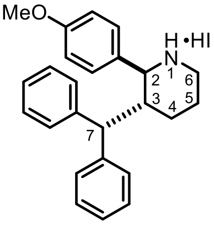

7a

$J_{C2\text{-}C3}$ = 2.2 Hz

$J_{C3\text{-}C7}$ = 11.9 Hz

$J_{C3\text{-}C4ax}$ = 4.2 Hz

$J_{C3\text{-}C4eq}$ = 2.2 Hz

$J_{C2\text{-}C3}$ = 11.2 Hz

$J_{C3\text{-}C7}$ = 3.7 Hz

$J_{C3\text{-}C4ax}$ = 11.5 Hz

$J_{C3\text{-}C4eq}$ = 3.5 Hz

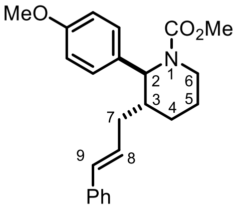

5c

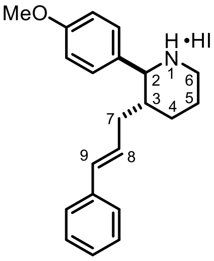

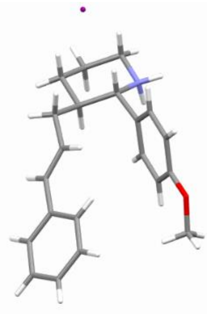

7c

$J_{C2\text{-}C3}$ = 3.0 Hz

$J_{C3\text{-}C7}$ = 7.0 Hz

$J_{C3\text{-}C4ax}$ = 3.0 Hz

$J_{C3\text{-}C4eq}$ = 3.0 Hz

$J_{C2\text{-}C3}$ = 11.2 Hz

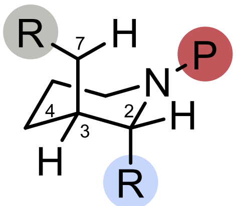

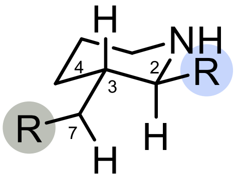

The relative stereochemistry of the piperidine products was corroborated through ^{1}H J coupling analysis (see above for representative examples) which support the 2,3-substituents adopting a diaxial conformation (nOe correlations provide little support for this due to the spatial positions of the key protons); X-ray data is available for **5iNs** which supports the above conclusion. Following the removal of the methyl carbamate group the 2,3-substituents then adopt a diequatorial conformation, which can be corroborated through 1H J coupling analysis (see above for representative examples) along with nOe correlations for **7a** and X-ray data for **7c**.

Crystallography

X-ray Crystallographic data for (5iNs): (±)-(2*S*,3*R*)-3-Benzhydryl-1-((2-nitrophenyl)-sulfonyl)-2-phenylpiperidine, 7372

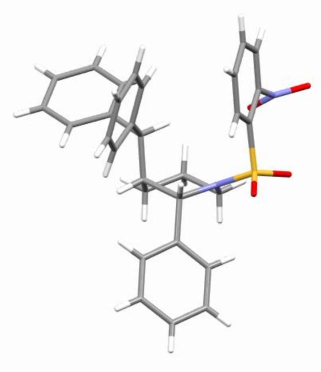

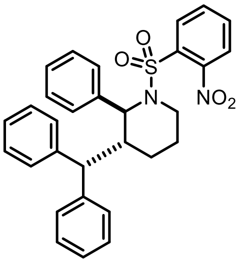

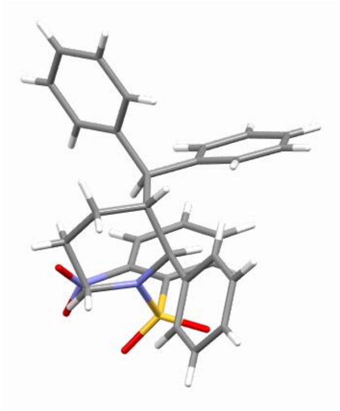

Crystals suitable for single crystal X-ray Crystallography were obtained *via* vapour diffusion of pentane into a solution of **5iNs** (10 mg) in EtOAc (0.5 mL).

Identification code	7372	
Empirical formula	C30 H28 N2 O4 S	
Formula weight	512.63	
Temperature	150 K	
Wavelength	1.54184 Å	
Crystal system	Monoclinic	
Space group	P 21/n	
Unit cell dimensions	a = 10.38990(10) Å	α= 90°.
	b = 23.2970(3) Å	β= 108.9135(16)°.
	c = 11.2415(2) Å	γ = 90°.
Volume	2574.13(7) $Å^3$	
Z	4	
Density (calculated)	1.323 Mg/m^3	
Absorption coefficient	1.437 mm^{-1}	
F(000)	1080	
Crystal size	0.20 x 0.18 x 0.09 mm^3	
Theta range for data collection	3.795 to 76.114°.	
Index ranges	-12<=h<=9, -29<=k<=28, -13<=l<=14	
Reflections collected	13843	
Independent reflections	5310 [R(int) = 0.026]	
Completeness to theta = 73.831°	99.9 %	
Absorption correction	Semi-empirical from equivalents	
Max. and min. transmission	0.88 and 0.85	
Refinement method	Full-matrix least-squares on F^2	
Data / restraints / parameters	5310 / 0 / 334	
Goodness-of-fit on F^2	0.9955	
Final R indices [I>2sigma(I)]	R1 = 0.0348, wR2 = 0.0885	
R indices (all data)	R1 = 0.0393, wR2 = 0.0935	
Largest diff. peak and hole	0.43 and -0.45 e.$Å^{-3}$	

X-ray Crystallographic data for (3k'): (±)-(2*S*,3*R*)-3-((*S*,*E*)-1,3-Diphenylallyl)-2-(4-methoxy-phenyl)-*N*-(4-nitrophenyl)-pyrro-lidine-1-carboxamide, 7493

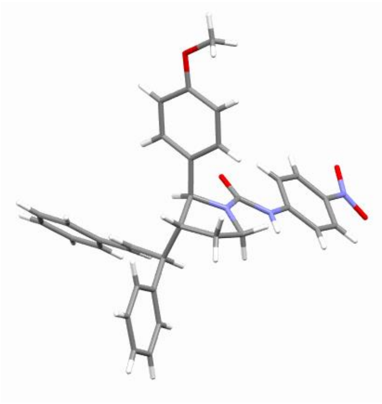

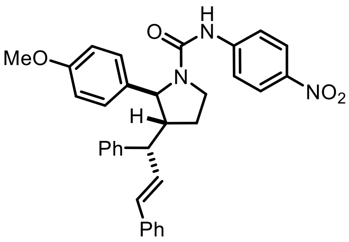

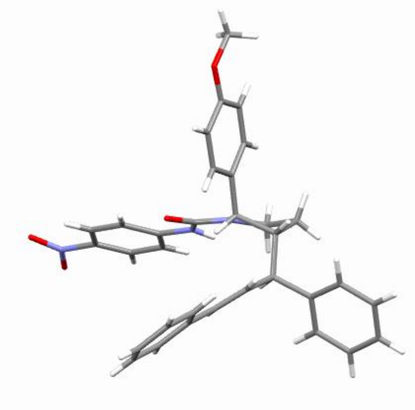

Crystals suitable for single crystal X-ray Crystallography were obtained *via* evaportation of a solution of **3k'** (18 mg) in heptane/EtOAc (0.5 mL).

Identification code	7493	
Empirical formula	C33 H31 N3 O4	
Formula weight	533.62	
Temperature	150 K	
Wavelength	1.54184 Å	
Crystal system	Orthorhombic	
Space group	P c a 21	
Unit cell dimensions	a = 9.4987(7) Å	α= 90°.
	b = 14.7759(11) Å	β= 90°.
	c = 19.5458(13) Å	γ = 90°.
Volume	2743.3(3) Å^3	
Z	4	
Density (calculated)	1.292 Mg/m^3	
Absorption coefficient	0.689 mm^{-1}	
F(000)	1127.993	
Crystal size	0.16 x 0.05 x 0.03 mm^3	
Theta range for data collection	4.524 to 76.720°.	
Index ranges	-11<=h<=11, -16<=k<=18, -21<=l<=24	
Reflections collected	16415	
Independent reflections	4936 [R(int) = 0.062]	
Completeness to theta = 73.831°	99.8 %	
Absorption correction	Semi-empirical from equivalents	
Max. and min. transmission	0.98 and 0.94	
Refinement method	Full-matrix least-squares on F^2	
Data / restraints / parameters	4932 / 1 / 362	
Goodness-of-fit on F^2	1.0100	
Final R indices [I>2sigma(I)]	R1 = 0.0556, wR2 = 0.1306	
R indices (all data)	R1 = 0.0845, wR2 = 0.1602	
Largest diff. peak and hole	0.19 and -0.15 e.Å^{-3}	

X-ray Crystallographic data for (7c): (±)-(2*S*,3*R*)-3-Cinnamyl-2-(4-methoxyphenyl)piperidin-1-ium iodide, 7509

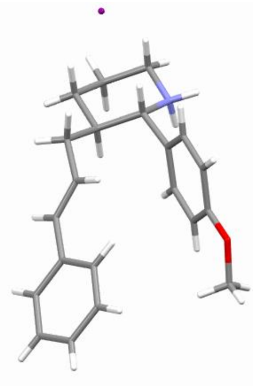

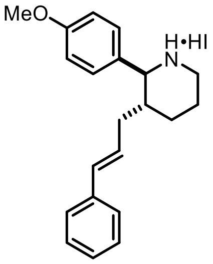

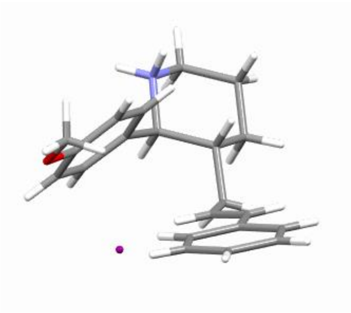

Crystals suitable for single crystal X-ray Crystallography were obtained *via* evaporation of **7c** (15 mg) in $CDCl_3$ (0.5 mL).

Identification code	7509	
Empirical formula	C21 H26 I N O	
Formula weight	435.35	
Temperature	150 K	
Wavelength	1.54184 Å	
Crystal system	Orthorhombic	
Space group	Pccn	
Unit cell dimensions	a = 45.0554(5) Å	α= 90°.
	b = 10.23090(10) Å	β= 90°.
	c = 8.46320(10) Å	γ = 90°.
Volume	3901.17(7) $Å^3$	
Z	8	
Density (calculated)	1.482 Mg/m^3	
Absorption coefficient	12.936 mm^{-1}	
F(000)	1760	
Crystal size	0.28 x 0.05 x 0.02 mm^3	
Theta range for data collection	3.925 to 76.529°.	
Index ranges	-56<=h<=56, -12<=k<=12, -10<=l<=10	
Reflections collected	95997	
Independent reflections	4096 [R(int) = 0.046]	
Completeness to theta = 76.529°	99.8 %	
Absorption correction	Semi-empirical from equivalents	
Max. and min. transmission	0.77 and 0.07	
Refinement method	Full-matrix least-squares on F^2	
Data / restraints / parameters	4095 / 0 / 217	
Goodness-of-fit on F^2	0.9999	
Final R indices [I>2sigma(I)]	R1 = 0.0709, wR2 = 0.1669	
R indices (all data)	R1 = 0.0718, wR2 = 0.1691	
Largest diff. peak and hole	2.68 and -0.38 e.$Å^{-3}$	

Copies of ^{1}H & ^{13}C NMR spectra for novel compounds

(1b): (*E*)-*N*-(4-(4-Methoxyphenyl)but-3-en-1-yl)methanesulfonamide

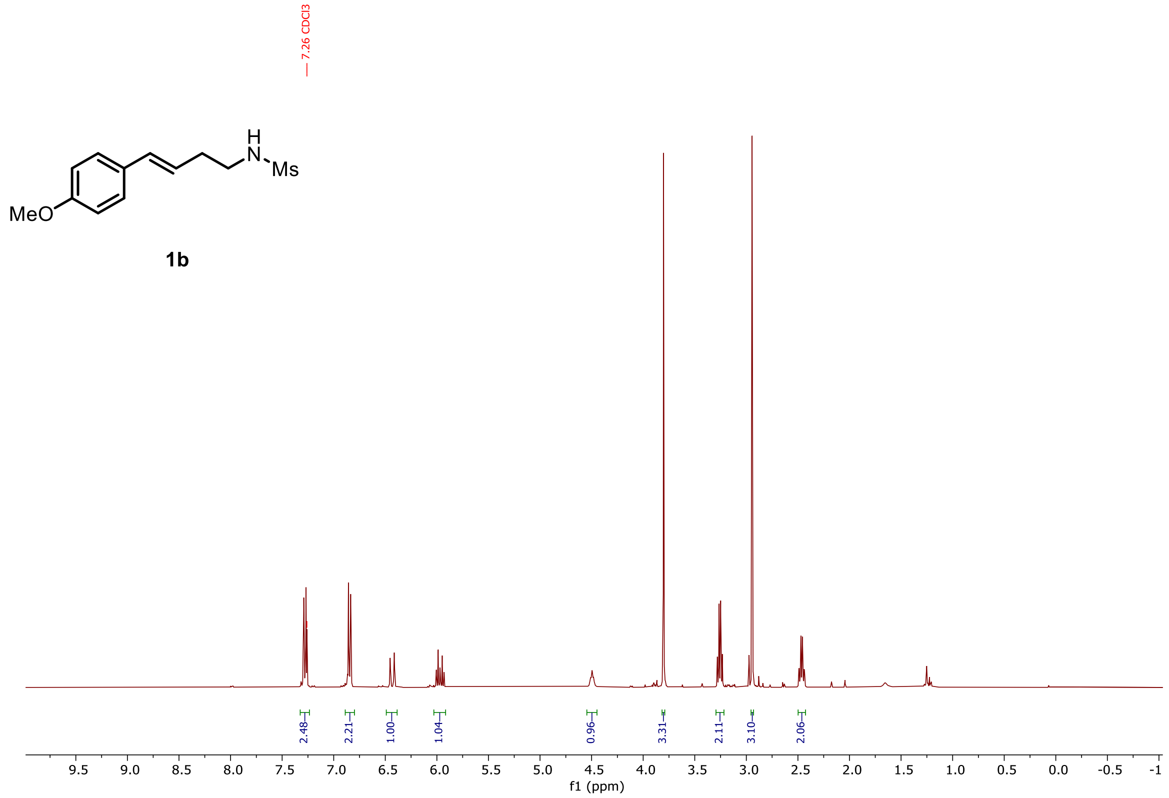

1b

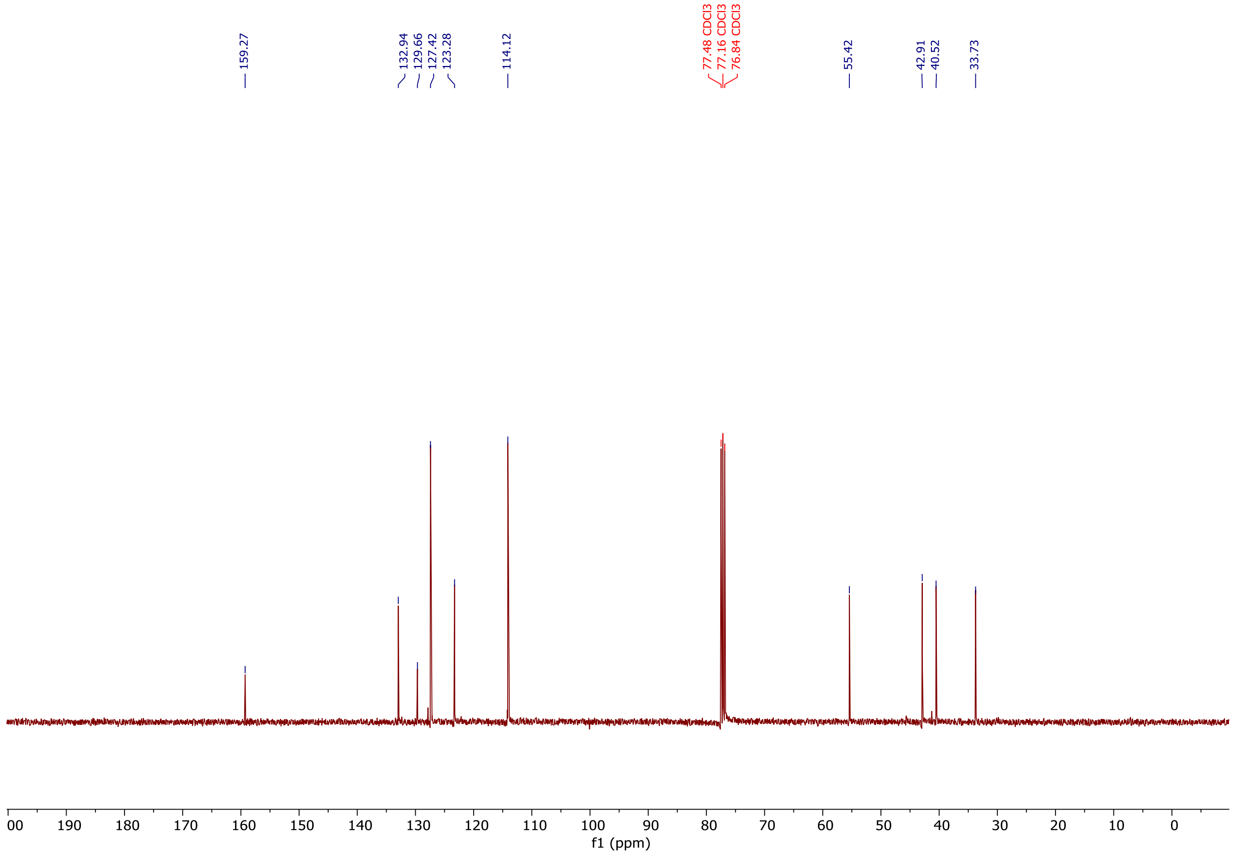

(1c): (3*E*)-*N*-[4-(4-Methoxyphenyl)but-3-en-1-yl]-2-nitrobenzenesulfonamide

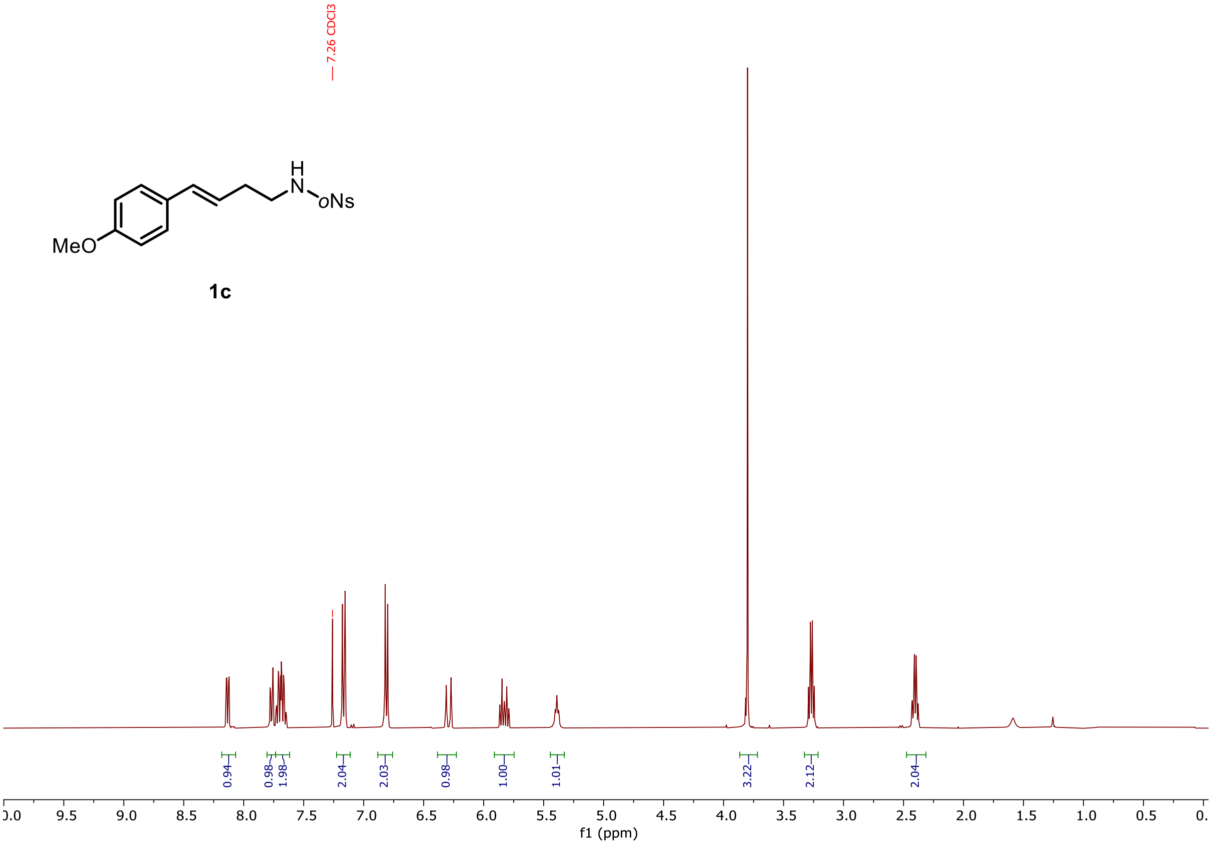

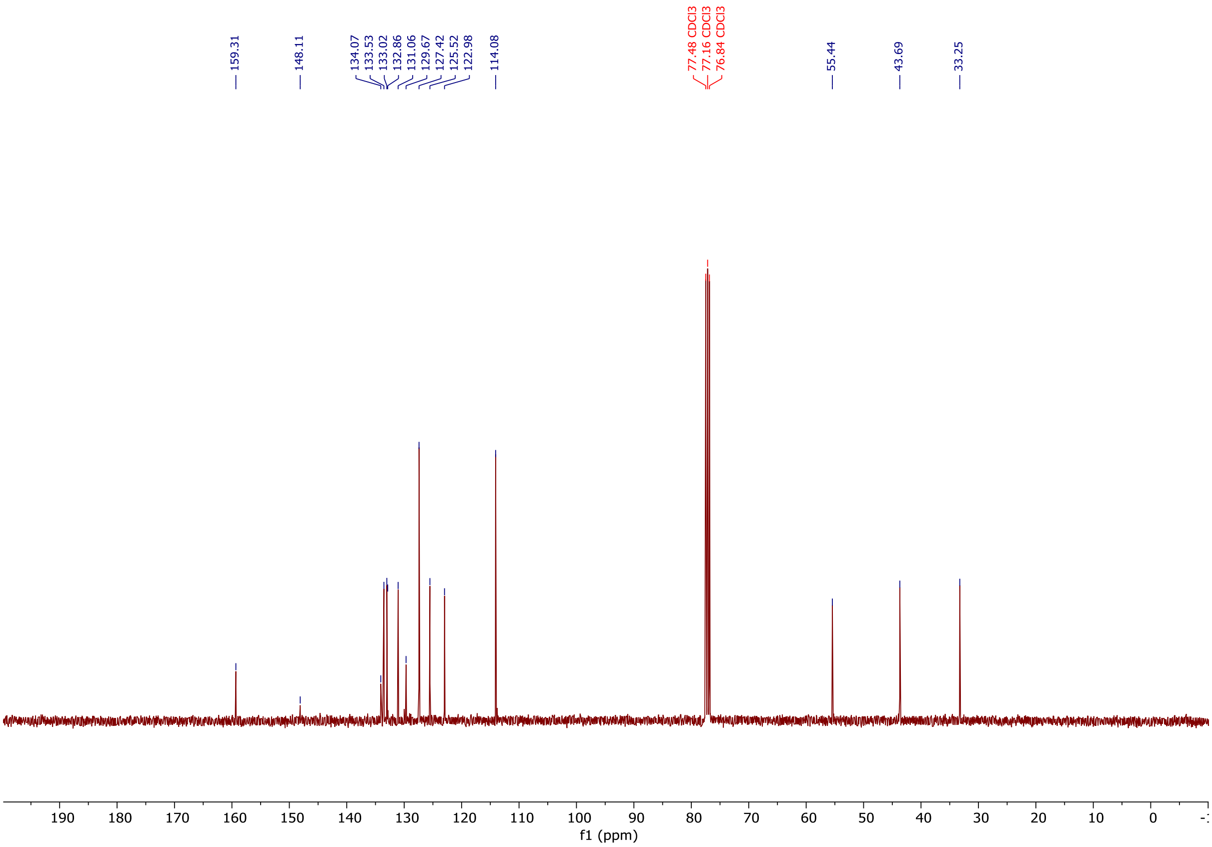

(1d): (3*E*)-*N*-[4-(4-Methoxyphenyl)but-3-en-1-yl]-4-nitrobenzenesulfonamide

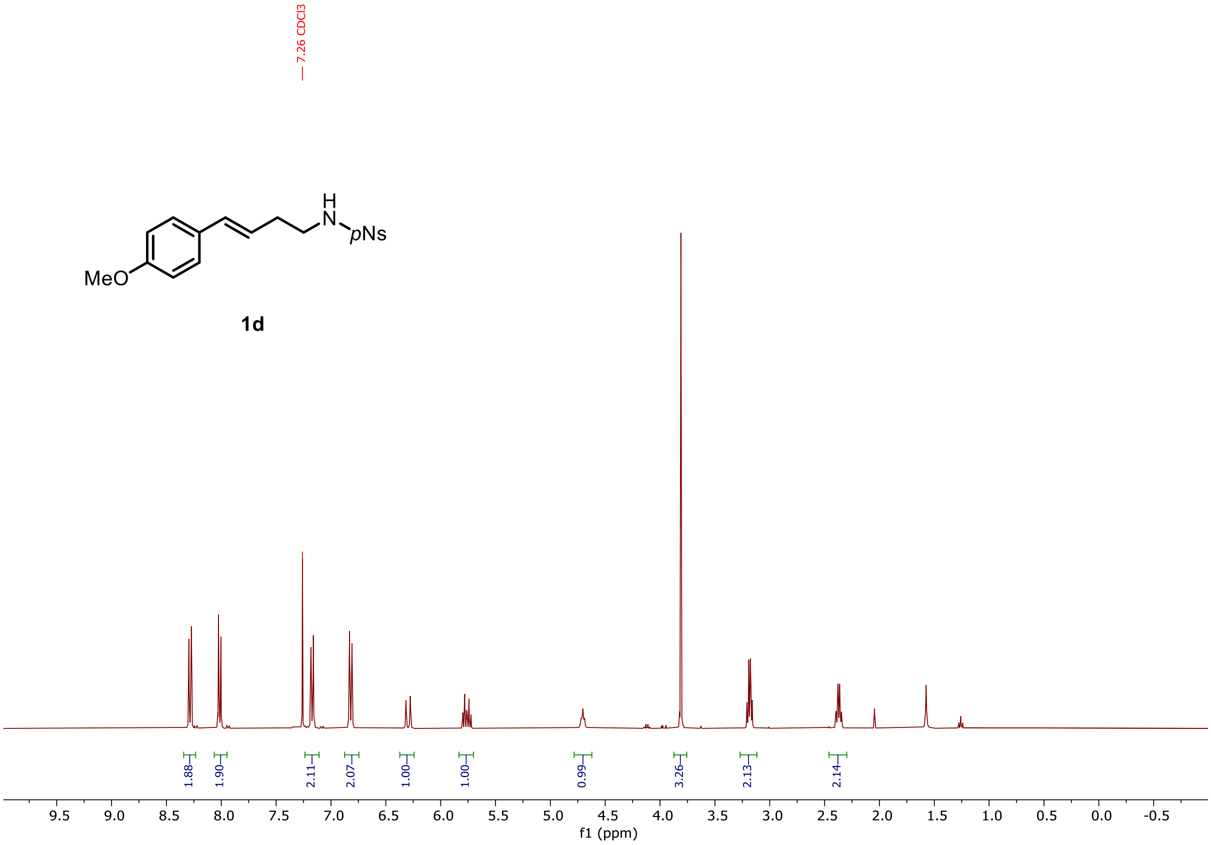

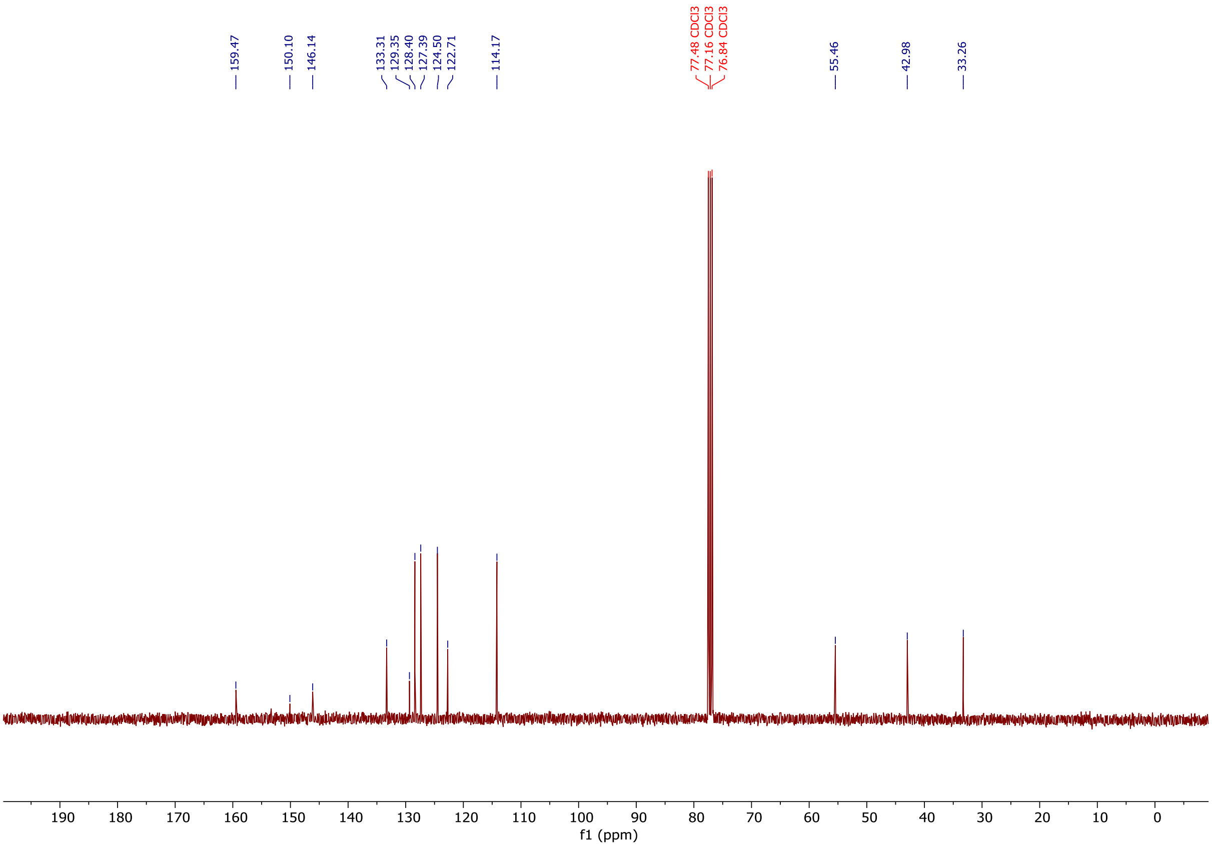

(1e): Benzyl (*E*)-(4-(4-methoxyphenyl)but-3-en-1-yl)carbamate

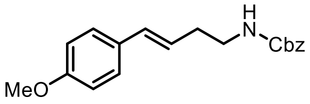

1e

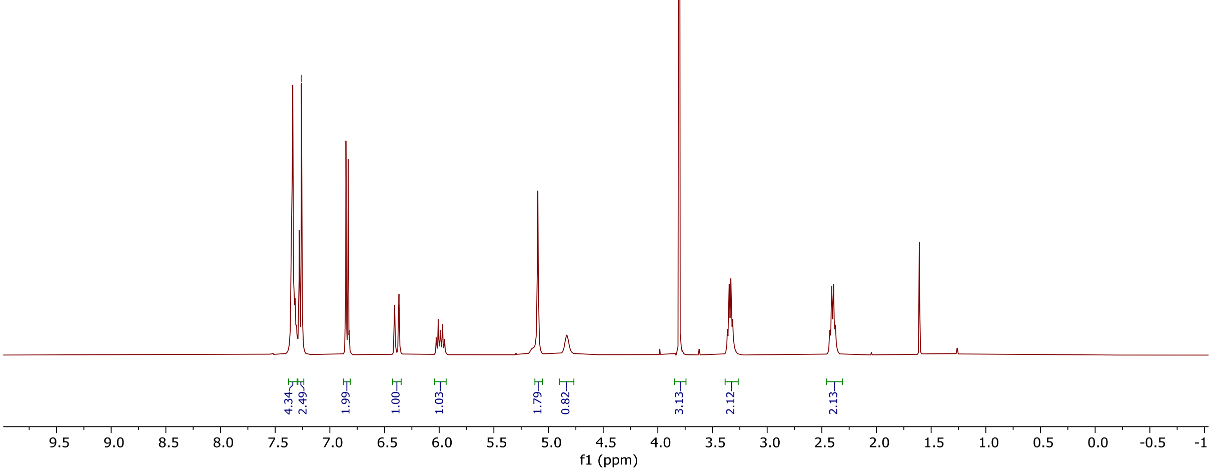

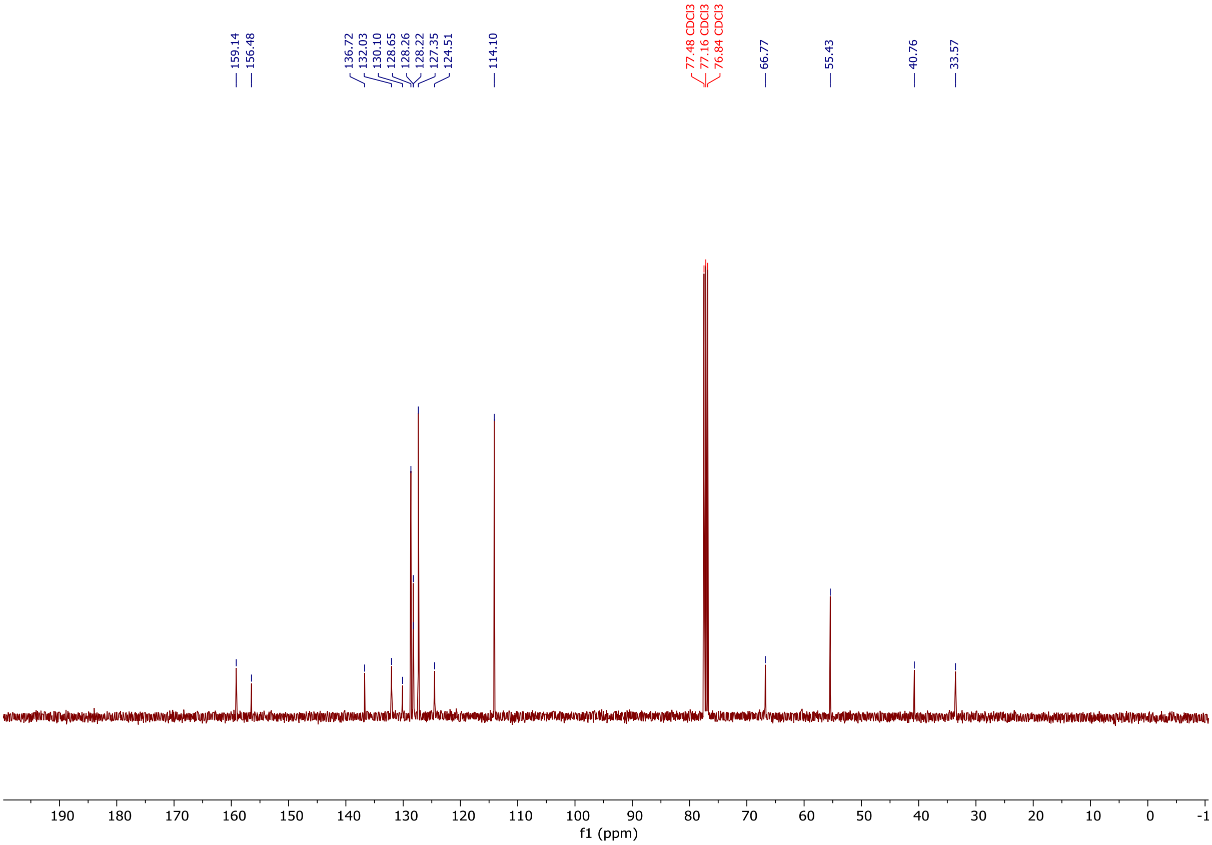

(1f): (3*E*)-*N*-Benzyl-4-(4-methoxyphenyl)but-3-en-1-amine

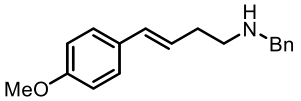

1f

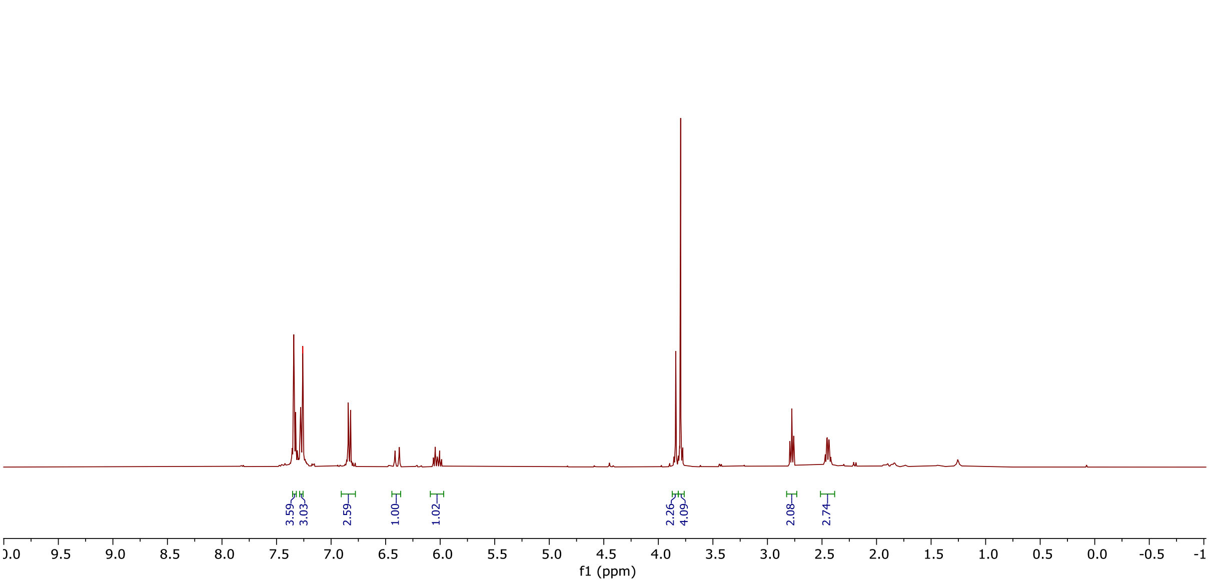

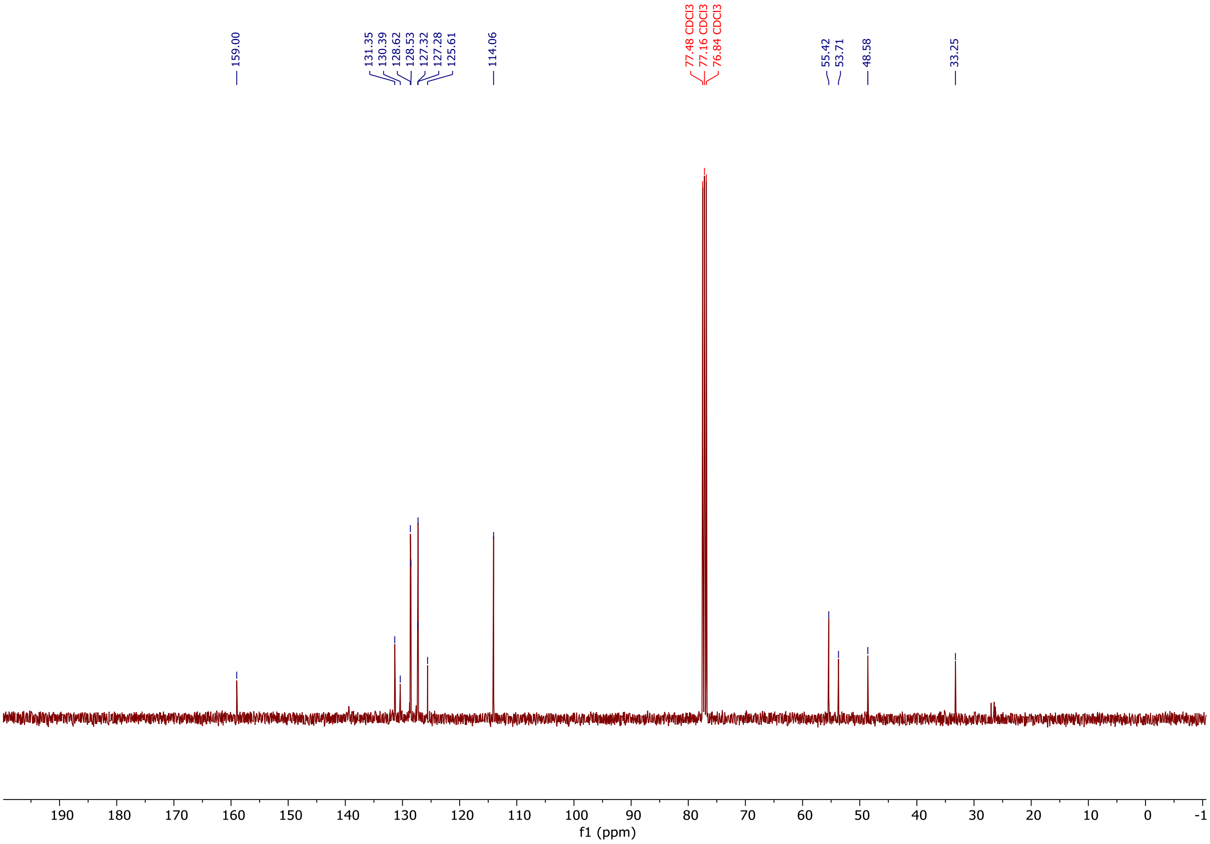

(S6): *tert*-Butyl (3*E*)-[(2-nitrophenyl)sulfonyl](4-phenylbut-3-en-1-yl)carbamate

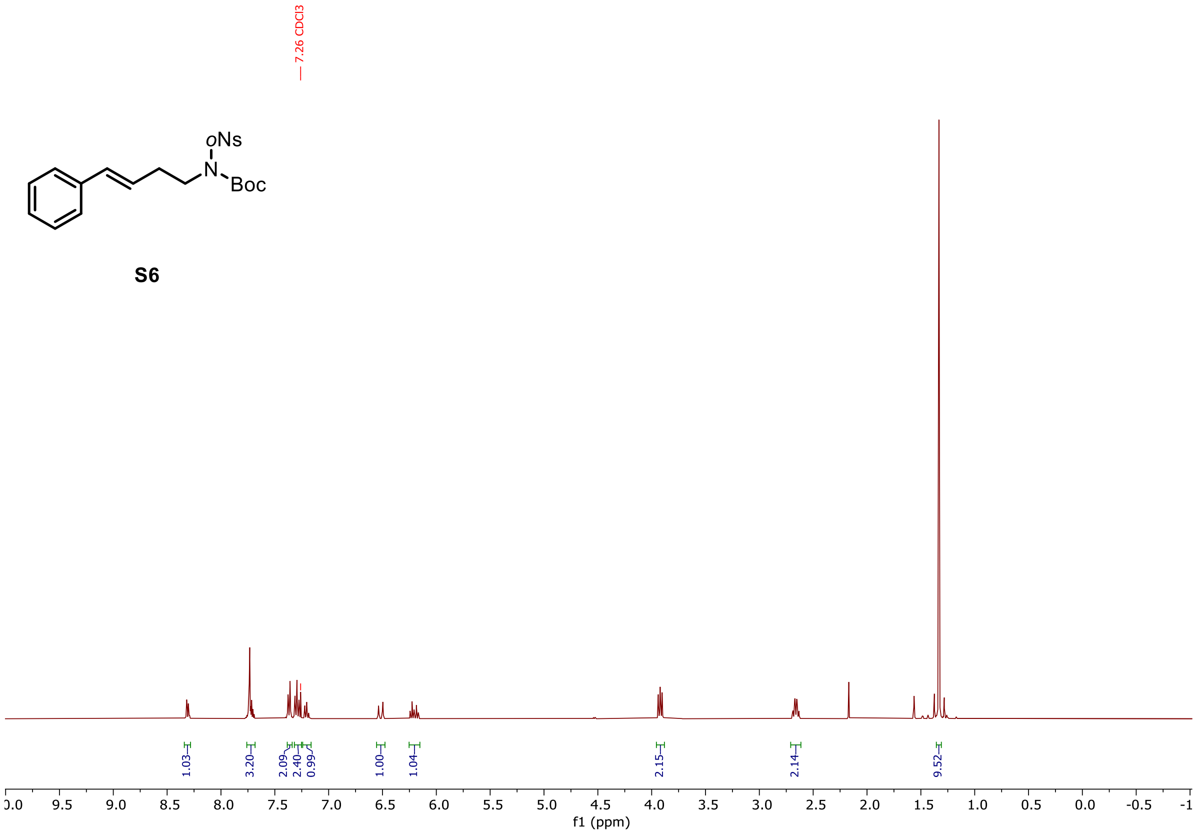

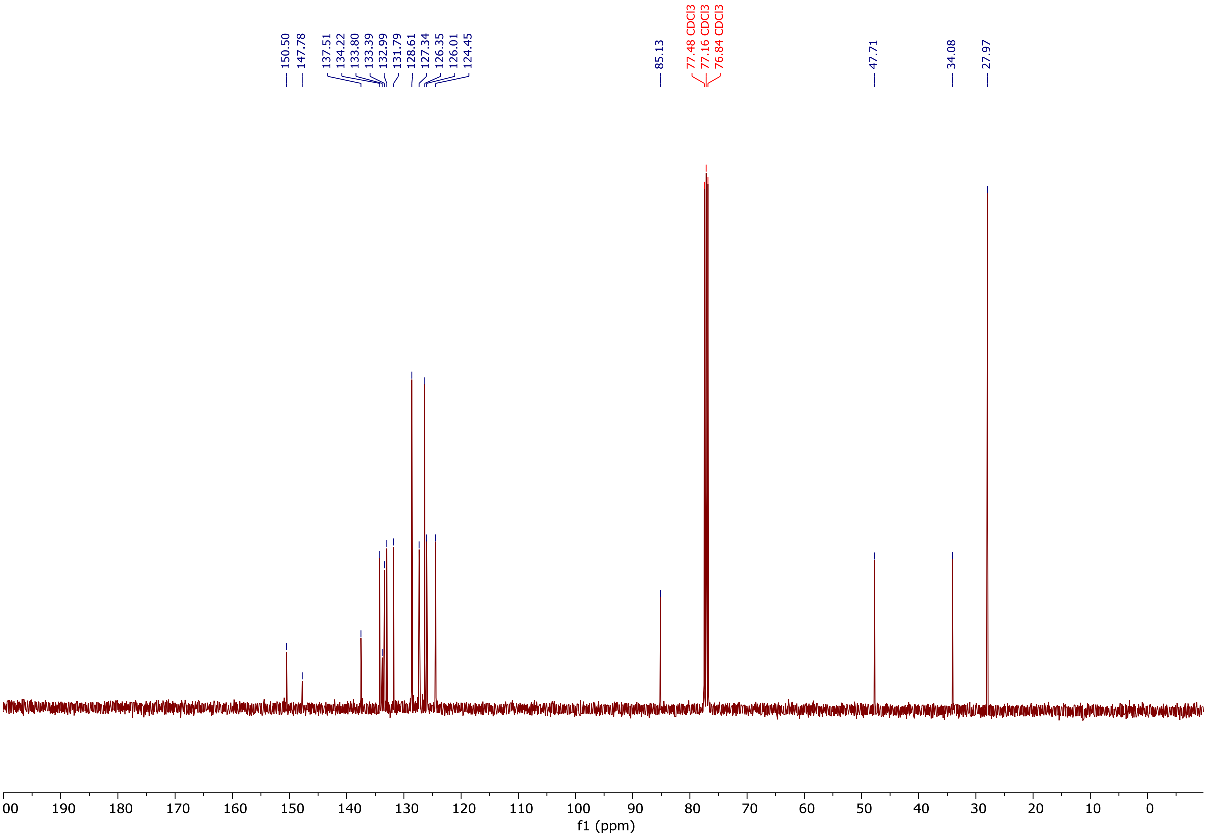

(S7): *tert*-Butyl but-3-en-1-yl[(2-nitrophenyl)sulfonyl]carbamate

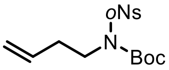

S7

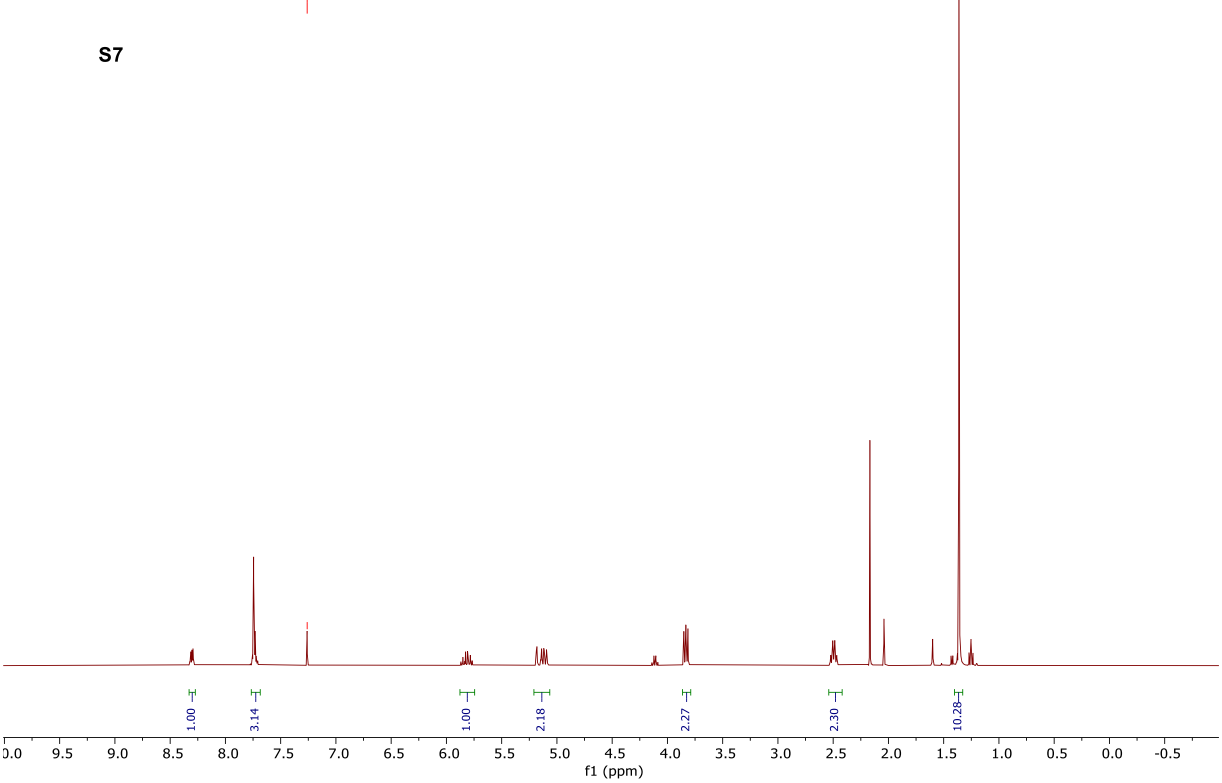

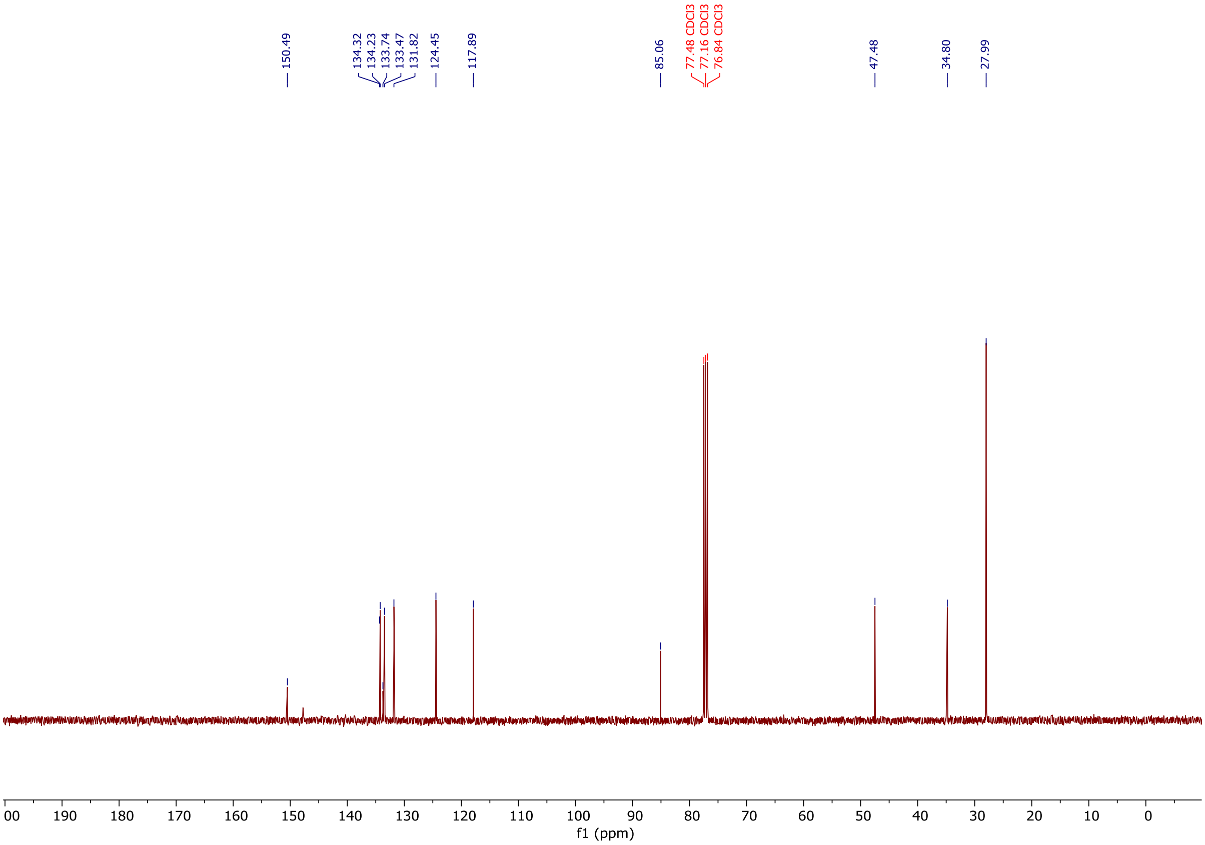

(1h): (3*E*)-*N*-[4-(4-Bromophenyl)but-3-en-1-yl]-2-nitrobenzenesulfonamide

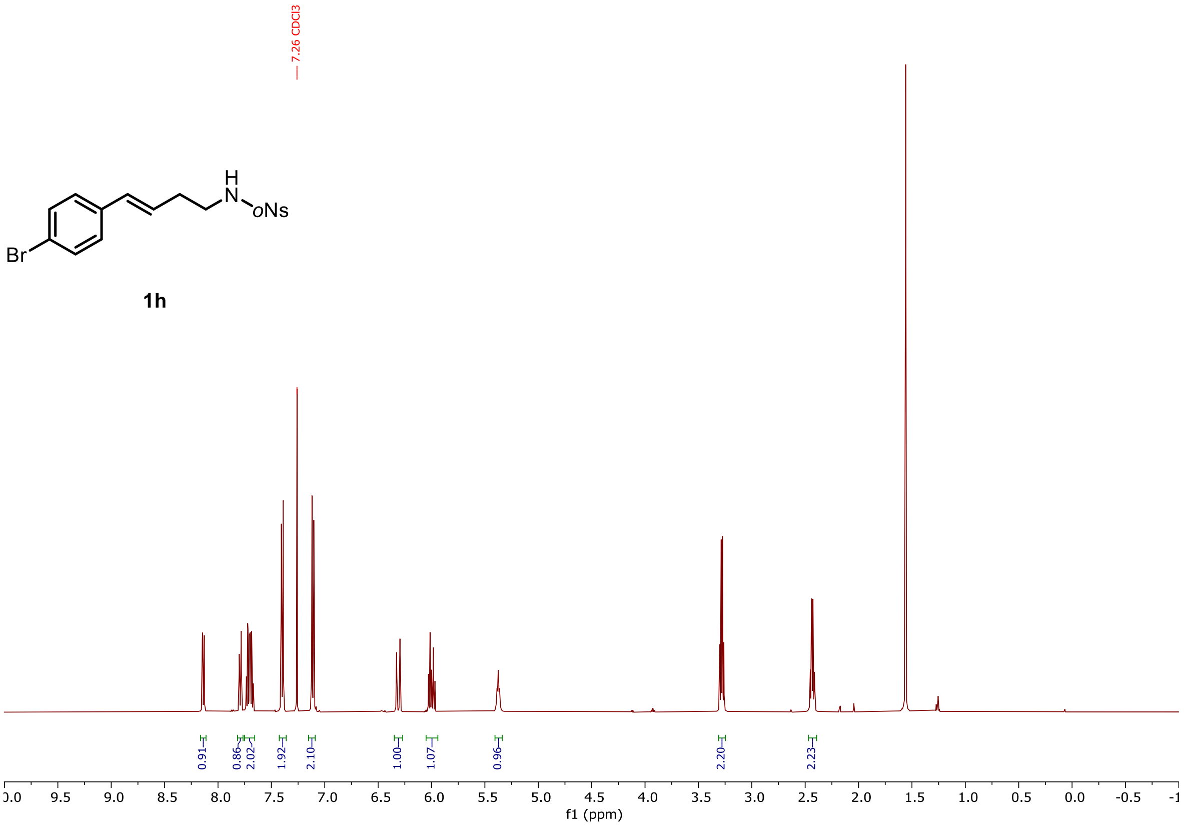

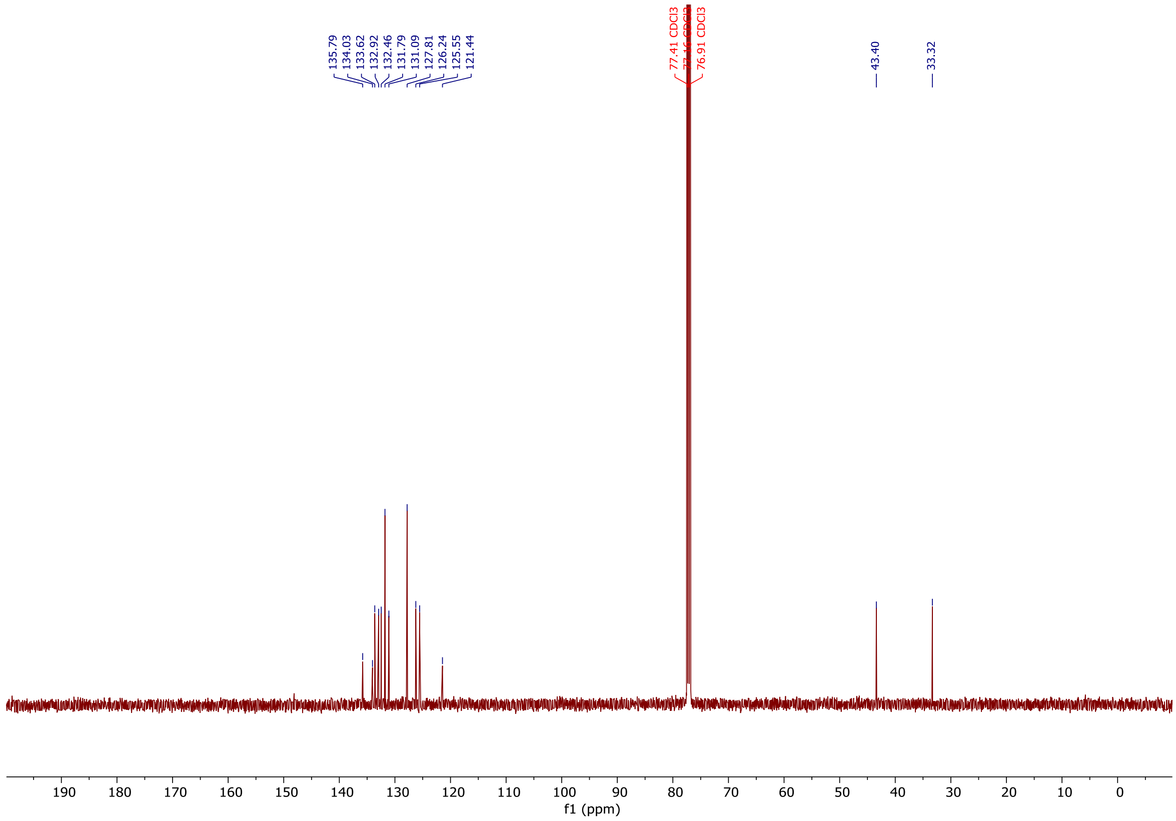

(1i): *N*-(4-Methylpent-3-en-1-yl)-2-nitrobenzenesulfonamide

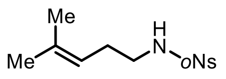

1i

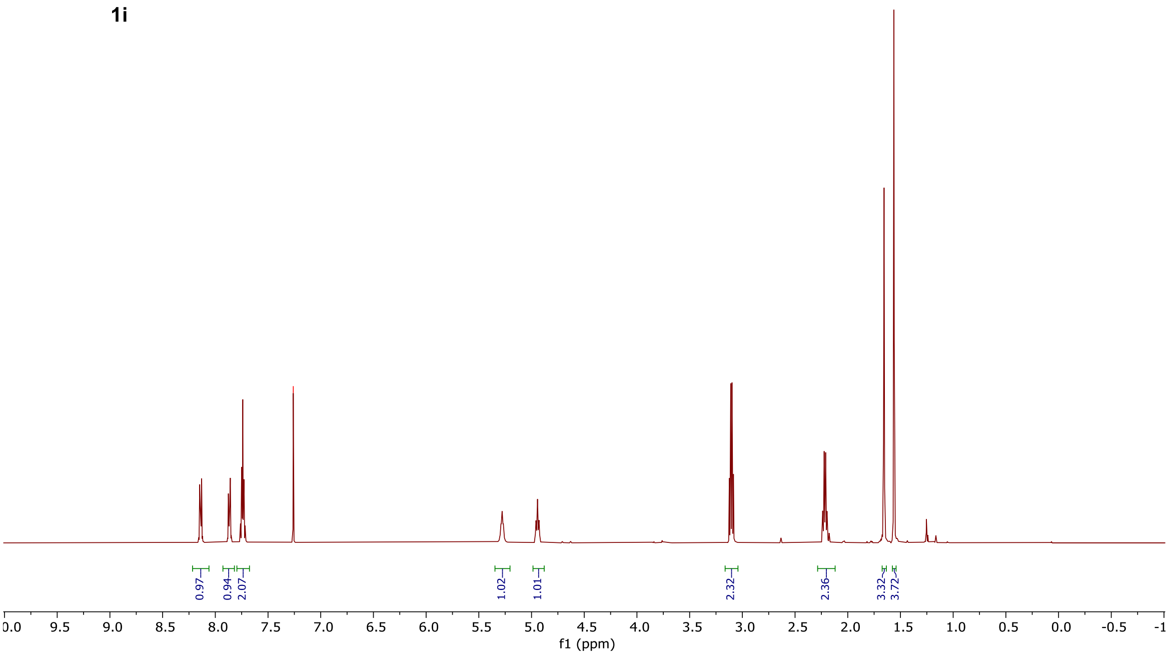

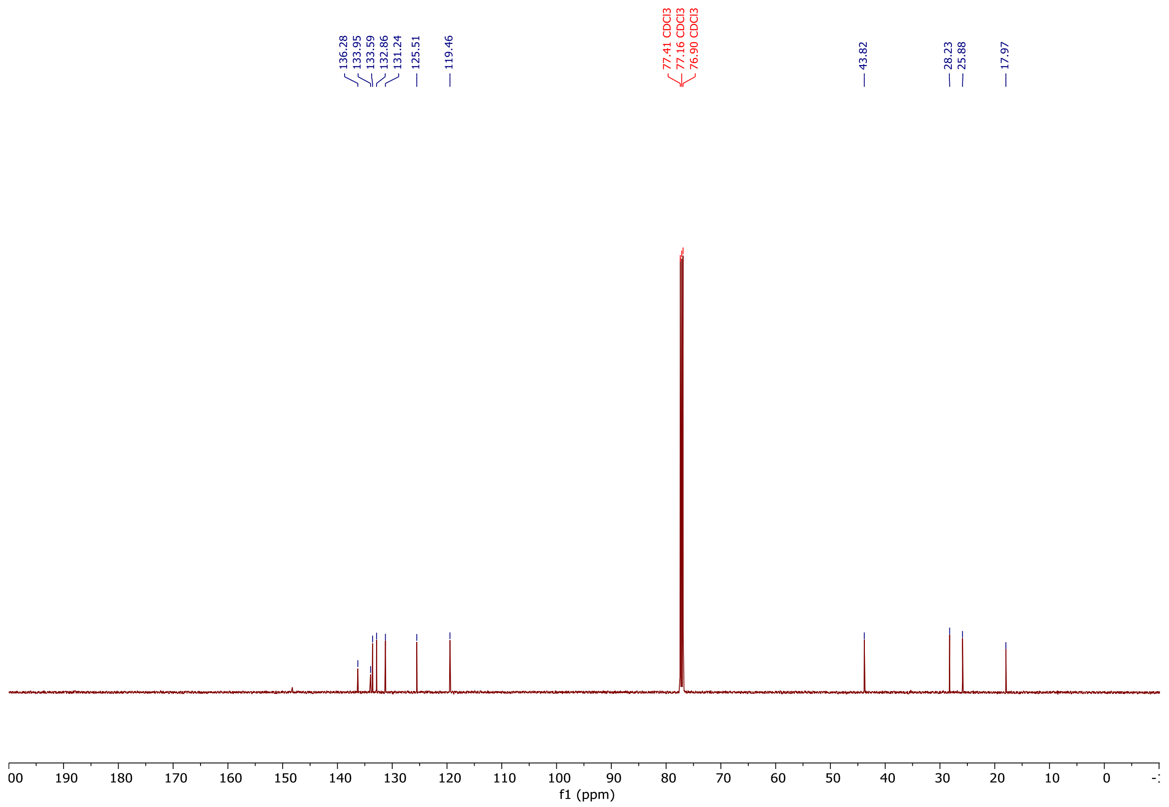

(1j): *N*-(3-Cyclopentylidenepropyl)-2-nitrobenzenesulfonamide

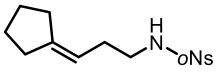

1j

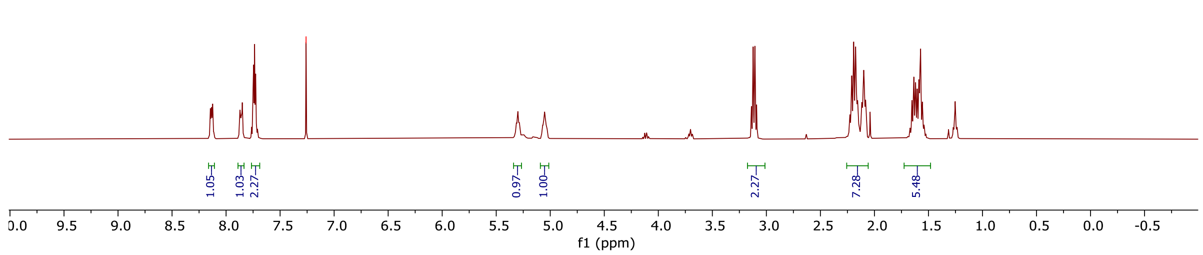

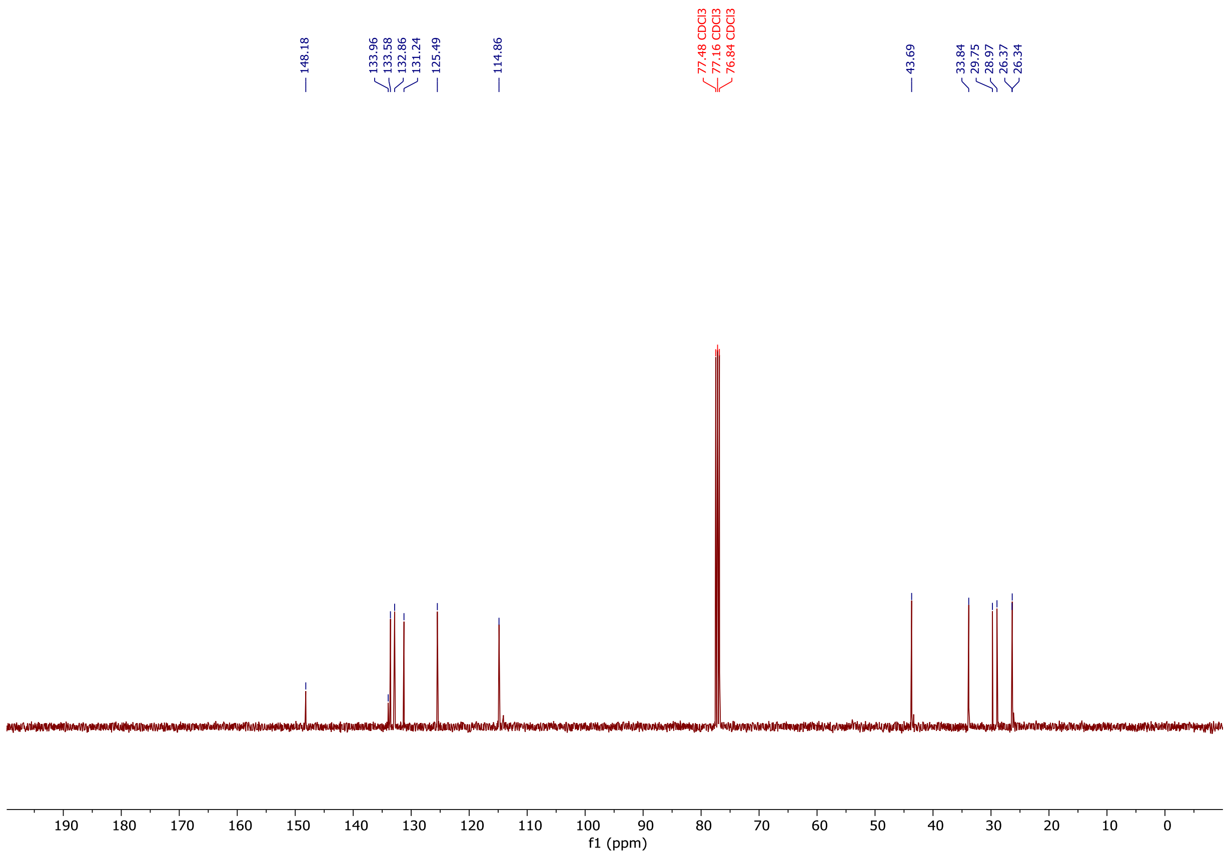

(1k): *N*-[(3*E*,5*E*)-6-(4-Methoxyphenyl)hexa-3,5-dien-1-yl]-2-nitrobenzenesulfonamide and *N*-[(3*E*,5*Z*)-6-(4-Methoxyphenyl)hexa-3,5-dien-1-yl]-2-nitrobenzenesulfonamide

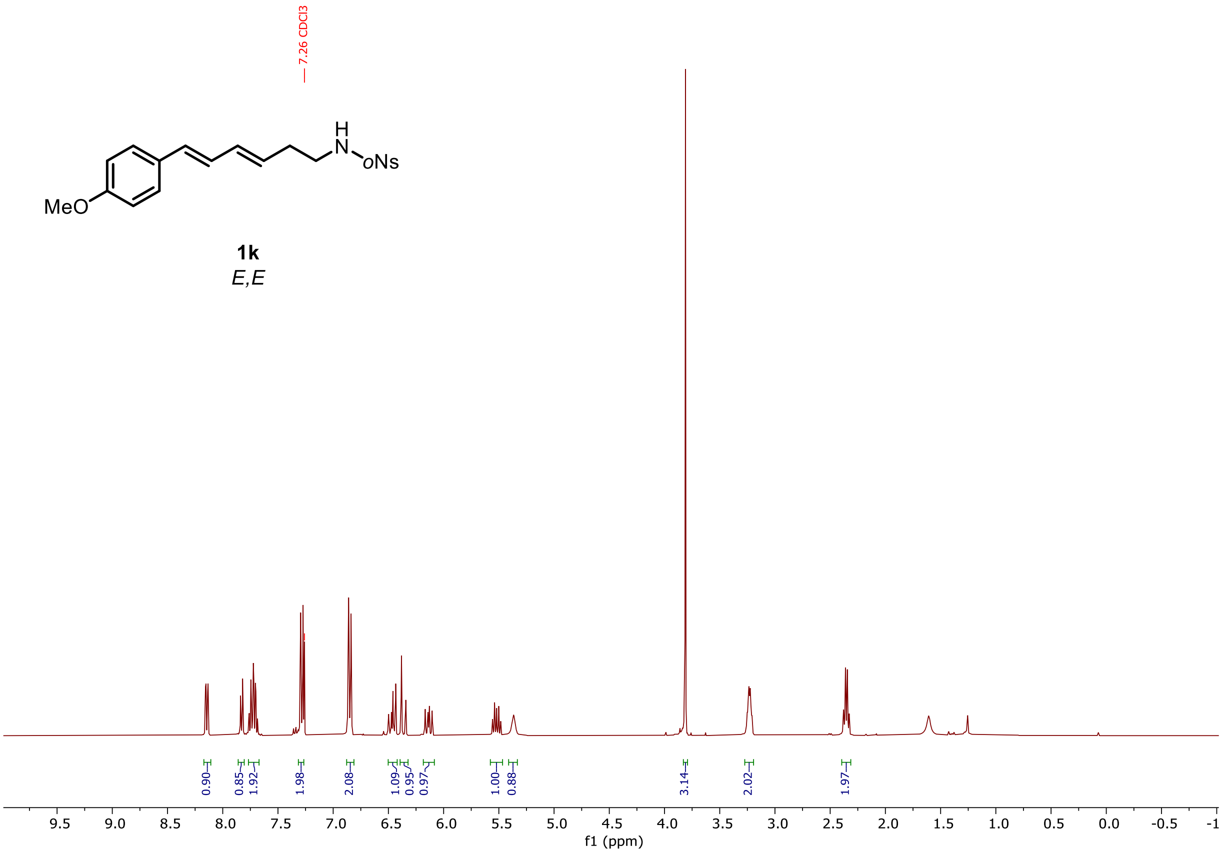

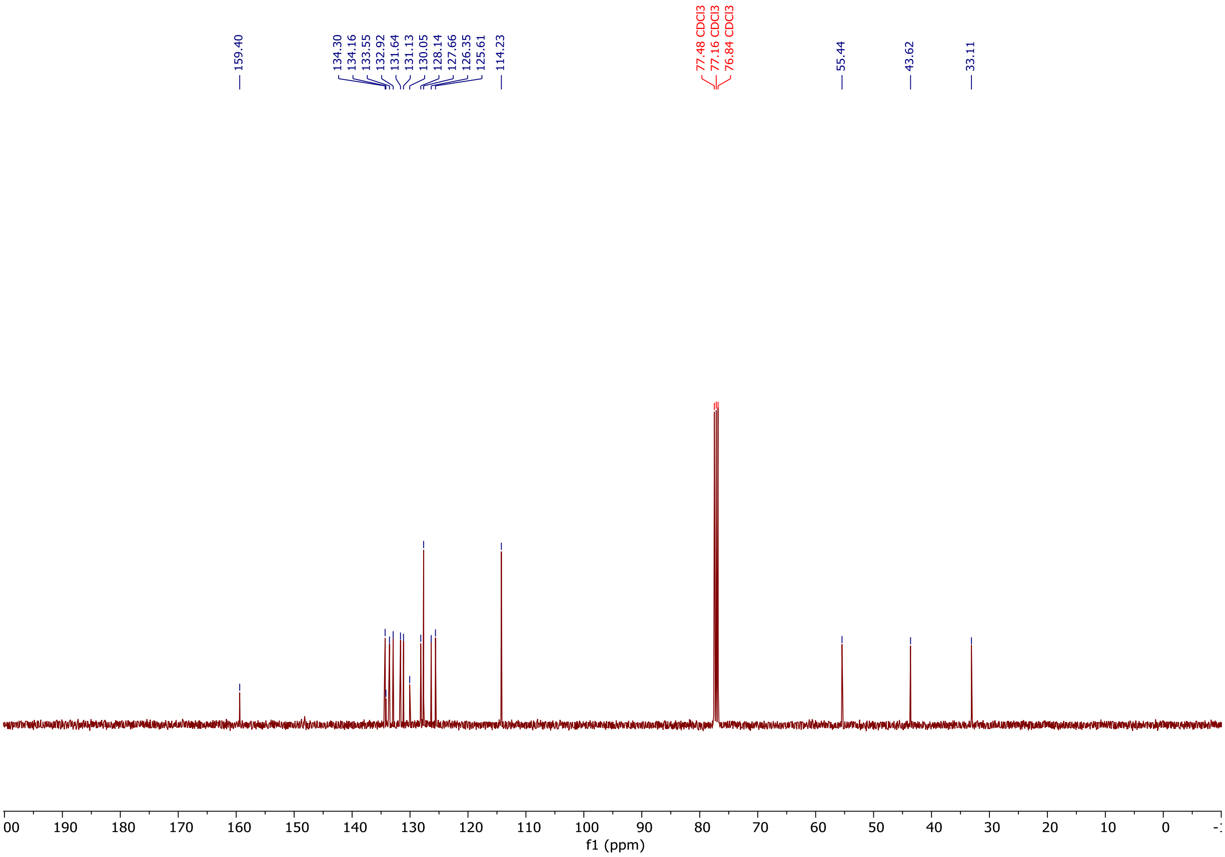

(1l): *N*-[(3*E*,5*E*)-6-Phenyl-hexa-3,5-dien-1-yl]-2-nitrobenzenesulfonamide and *N*-[(3*E*,5*Z*)-6-Phenyl-hexa-3,5-dien-1-yl]-2-nitrobenzenesulfonamide

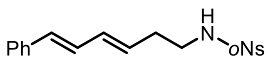

1l
E,E

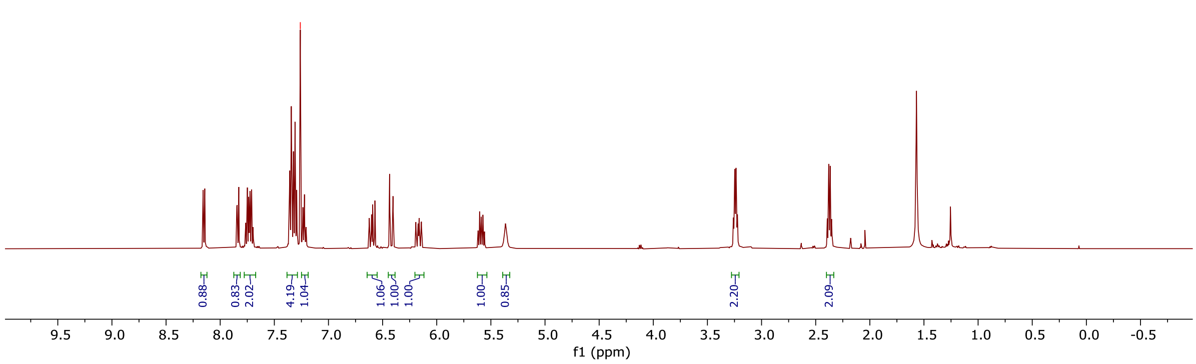

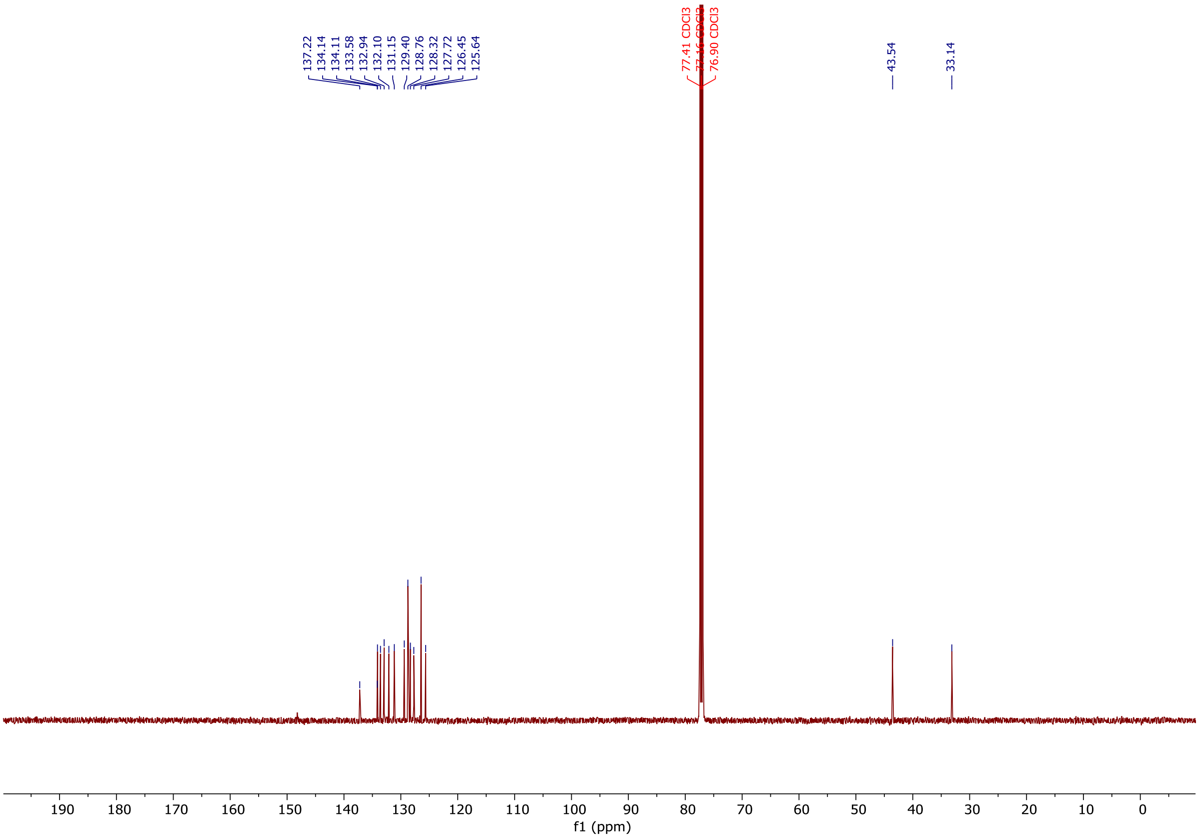

(1m): (3*E*)-2-Nitro-*N*-[5-(triisopropylsilyl)pent-3-en-1-yl]benzenesulfonamide and (3*Z*)-2-Nitro-*N*-[5-(triisopropylsilyl)pent-3-en-1-yl]benzenesulfonamide

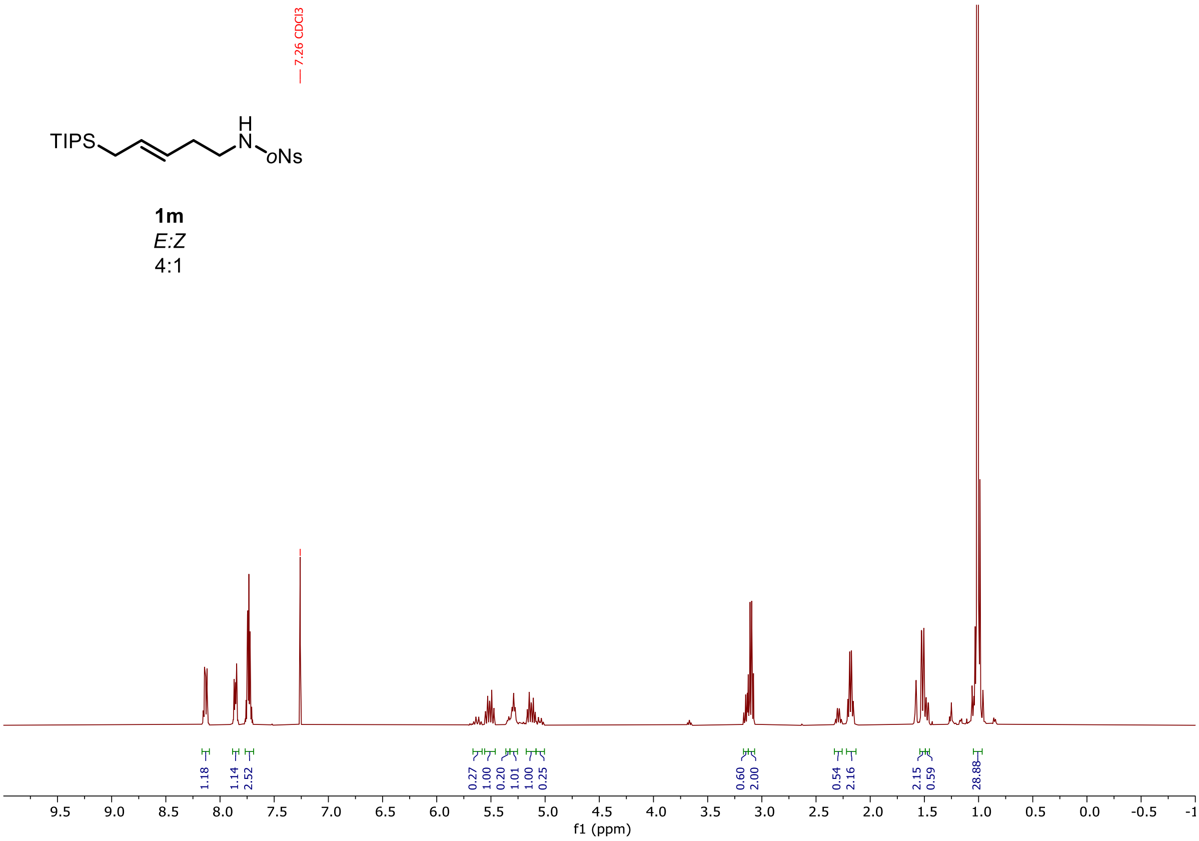

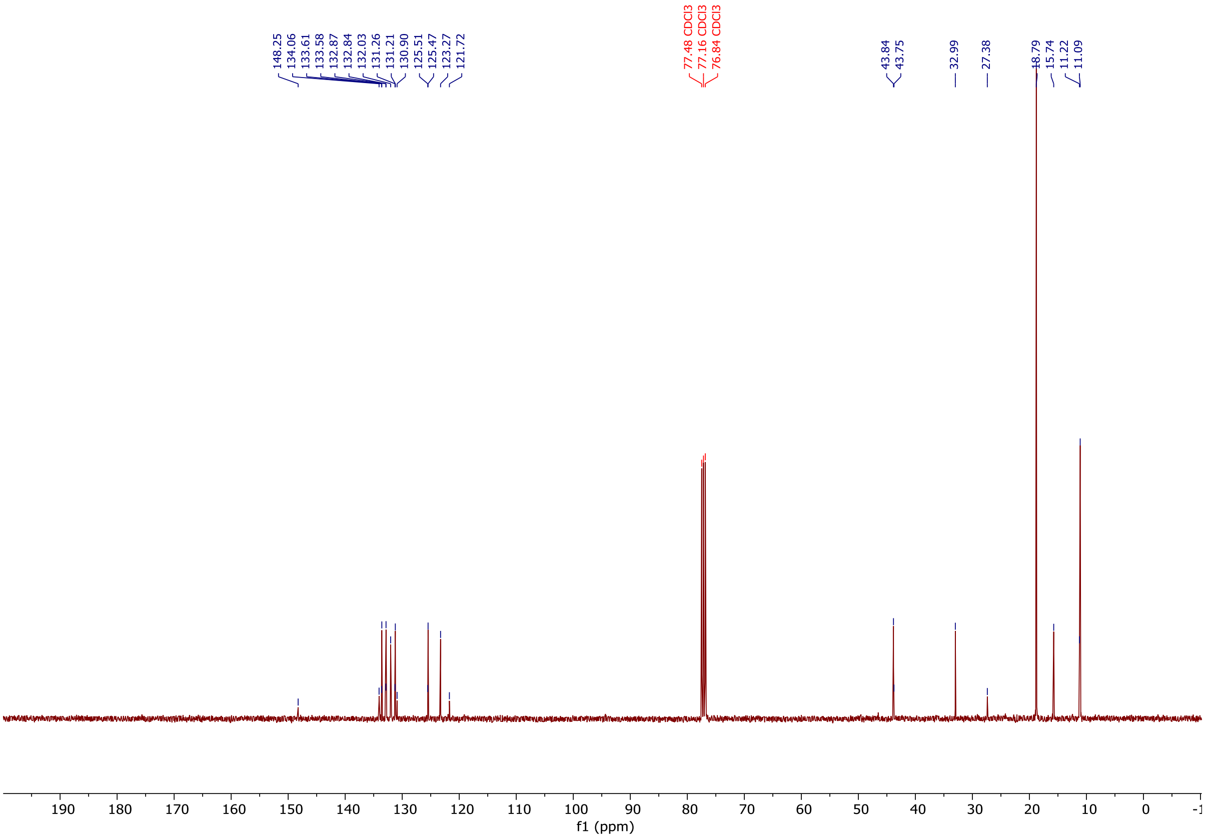

(1n): (3*E*)-2-Nitro-*N*-[5-(*tert*-butyldiphenylsilyl)pent-3-en-1-yl]benzenesulfonamide and (3*Z*)-2-Nitro-*N*-[5-(*tert*-butyldiphenylsilyl)pent-3-en-1-yl]benzenesulfonamide

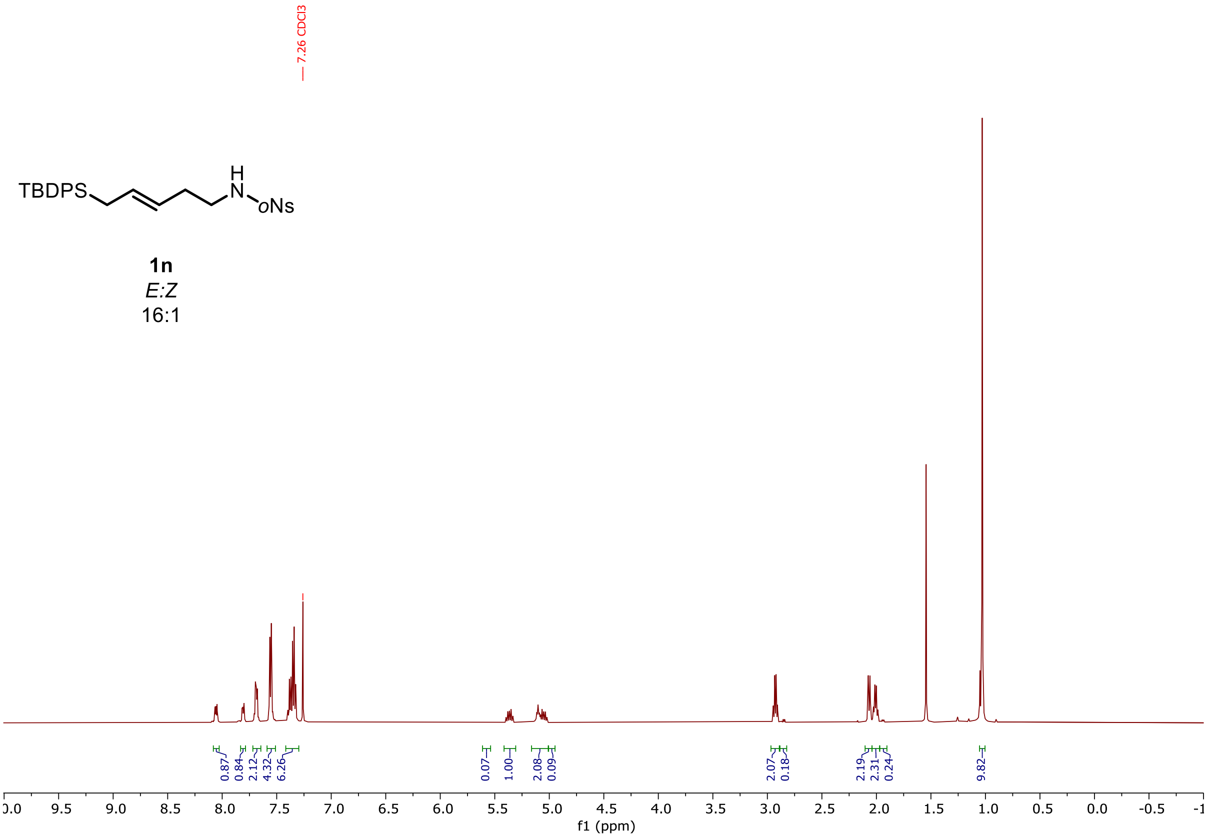

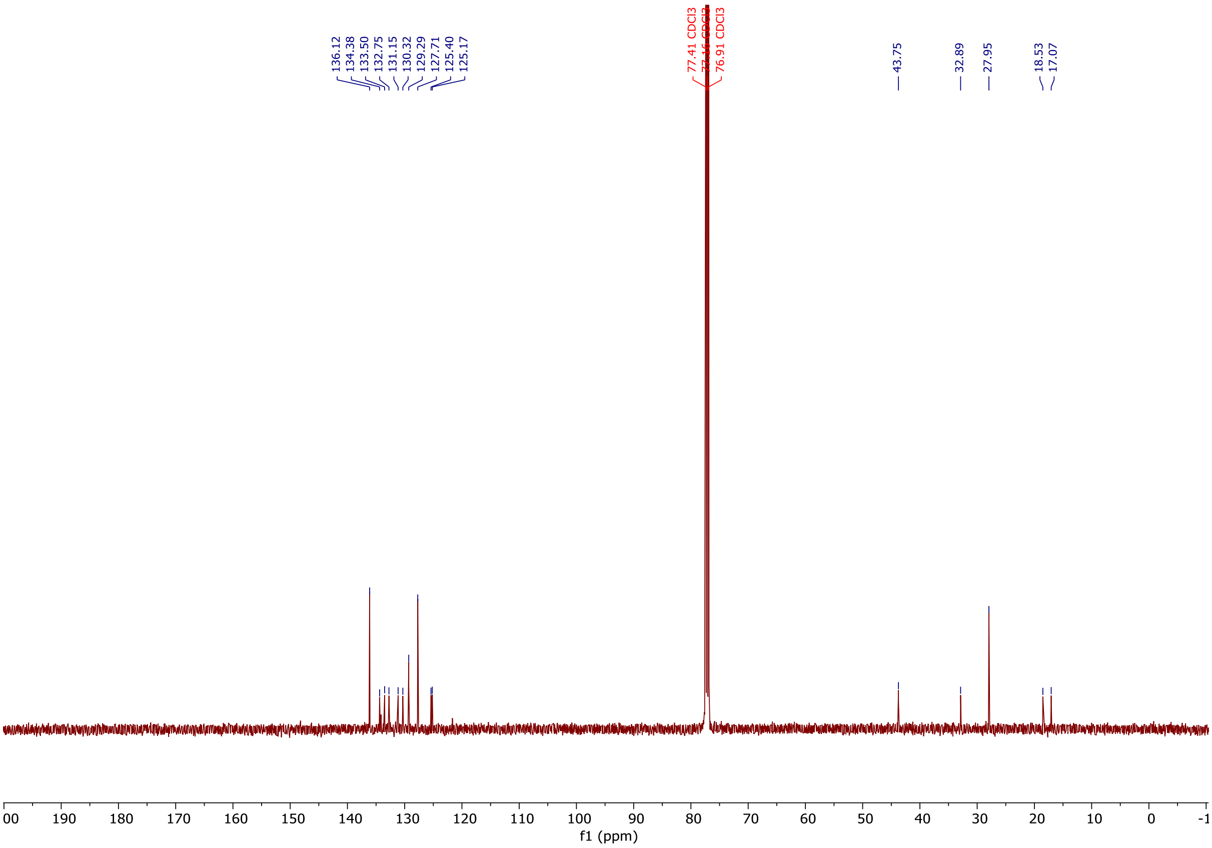

((–)-S14): (–)-*tert*-Butyl (2*S*)-[(2-nitrophenyl)sulfonyl](2-phenylbut-3-en-1-yl)carbamate

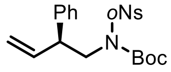

(–)-S14

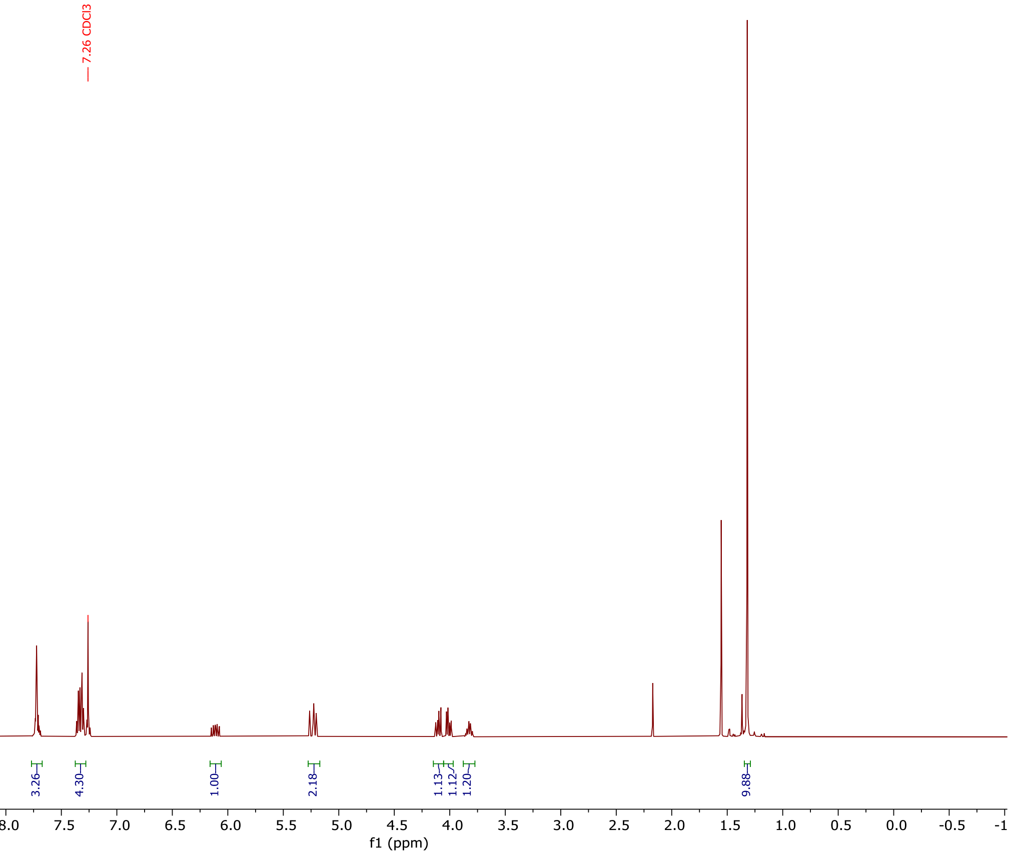

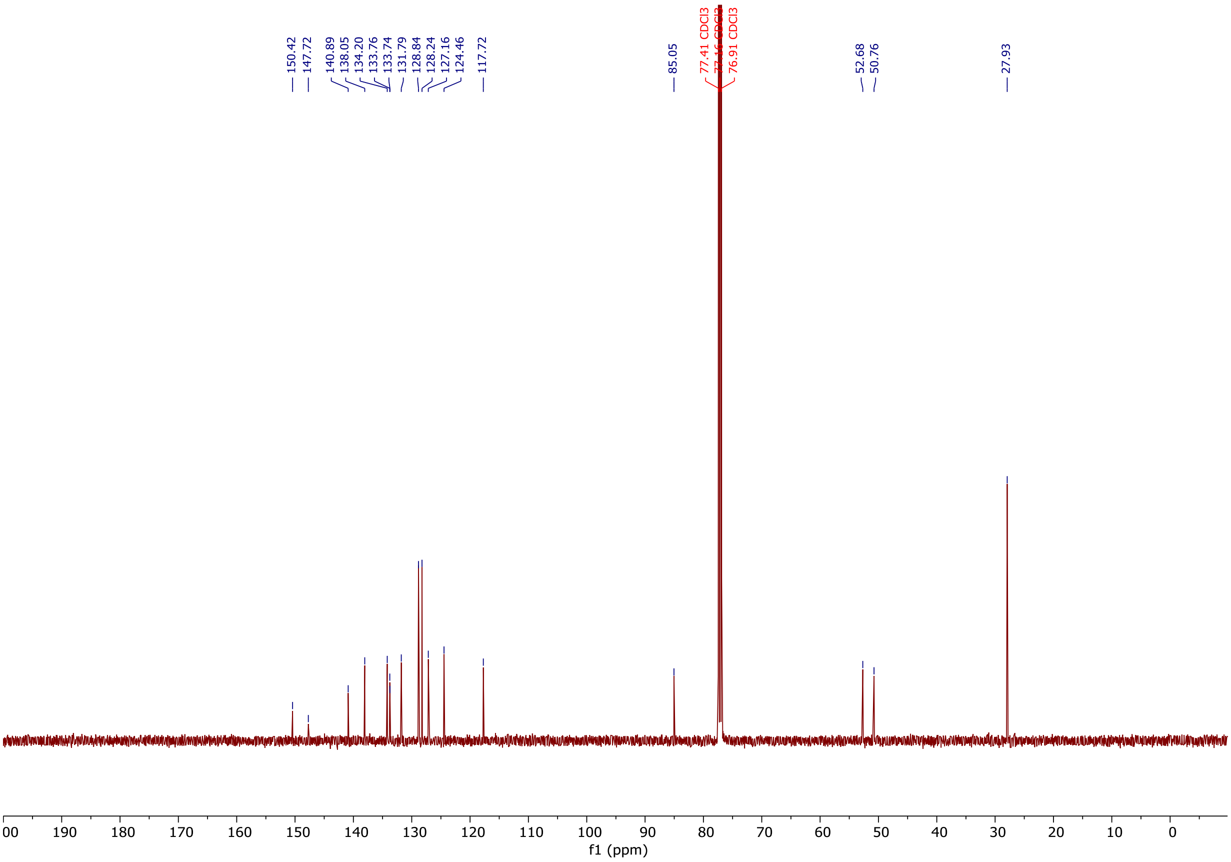

((+)-S15): (+)-(2*S*)-2-Nitro-*N*-(2-phenylbut-3-en-1-yl)benzenesulfonamide

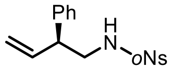

(+)-S15

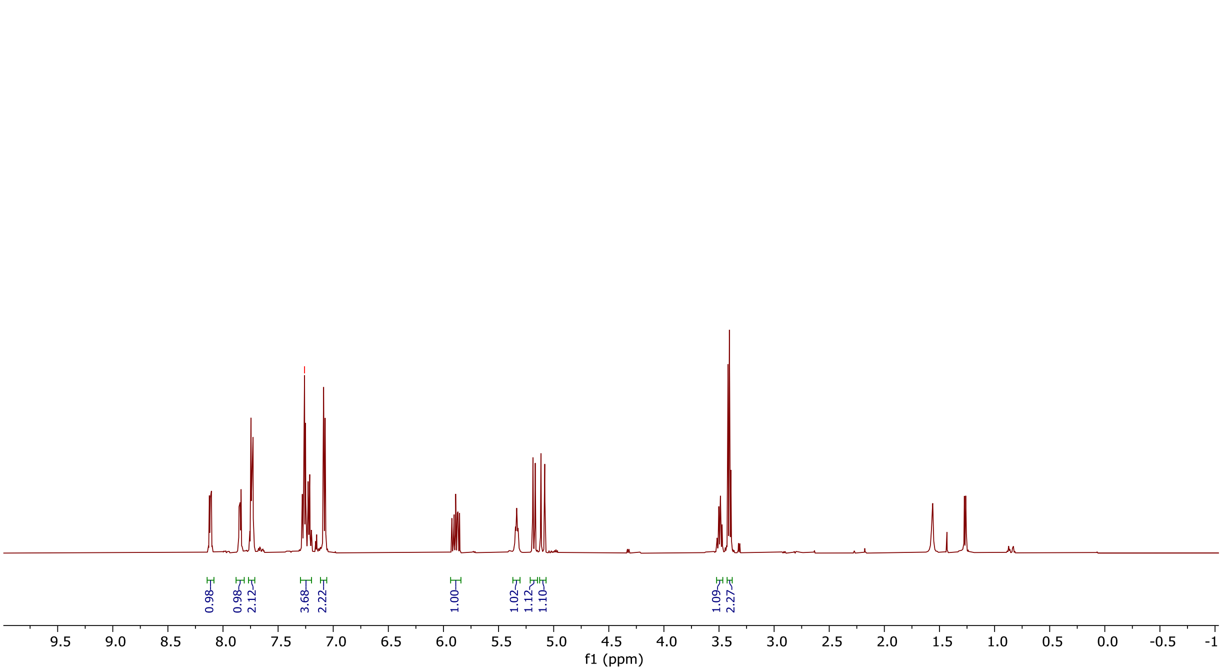

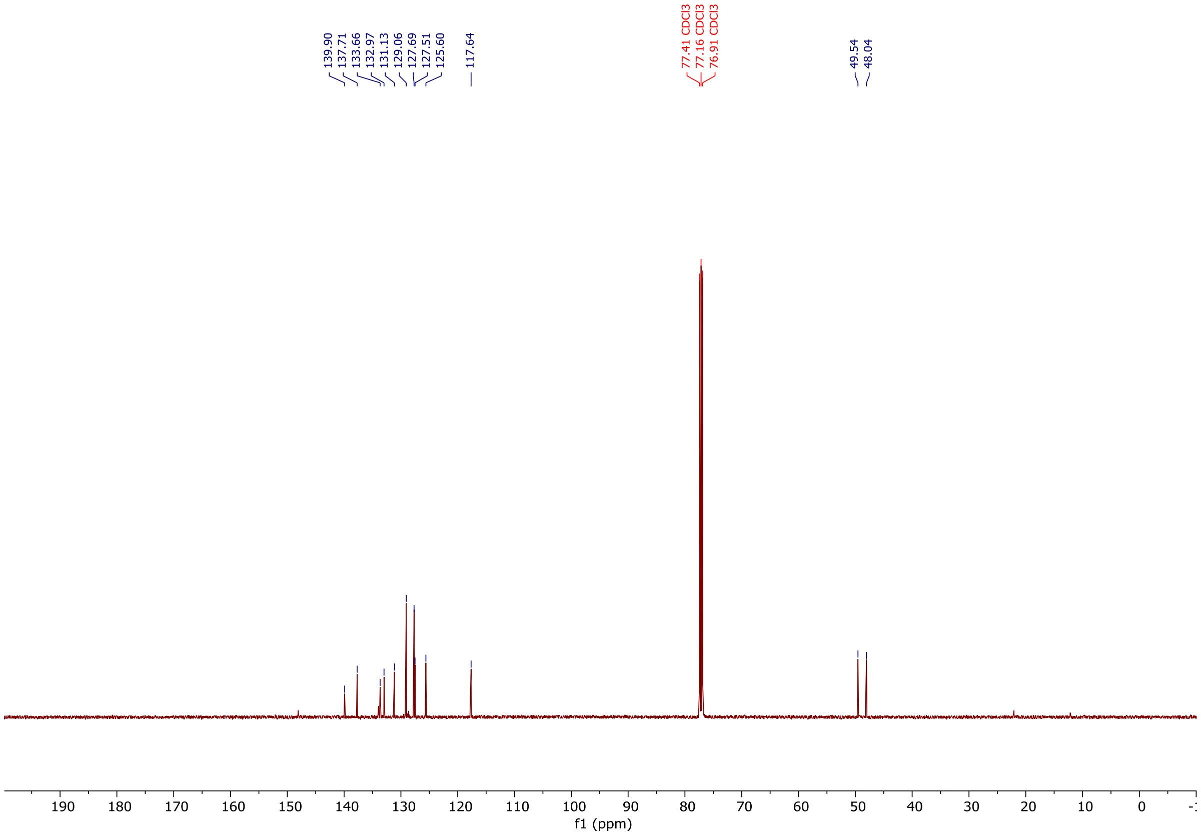

((–)-1o): (–)-(2*S*,3*E*)-*N*-[4-(4-Methoxyphenyl)-2-phenylbut-3-en-1-yl]-2-nitrobenzenesulfonamide

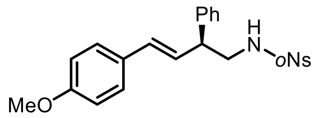

(–)-1o

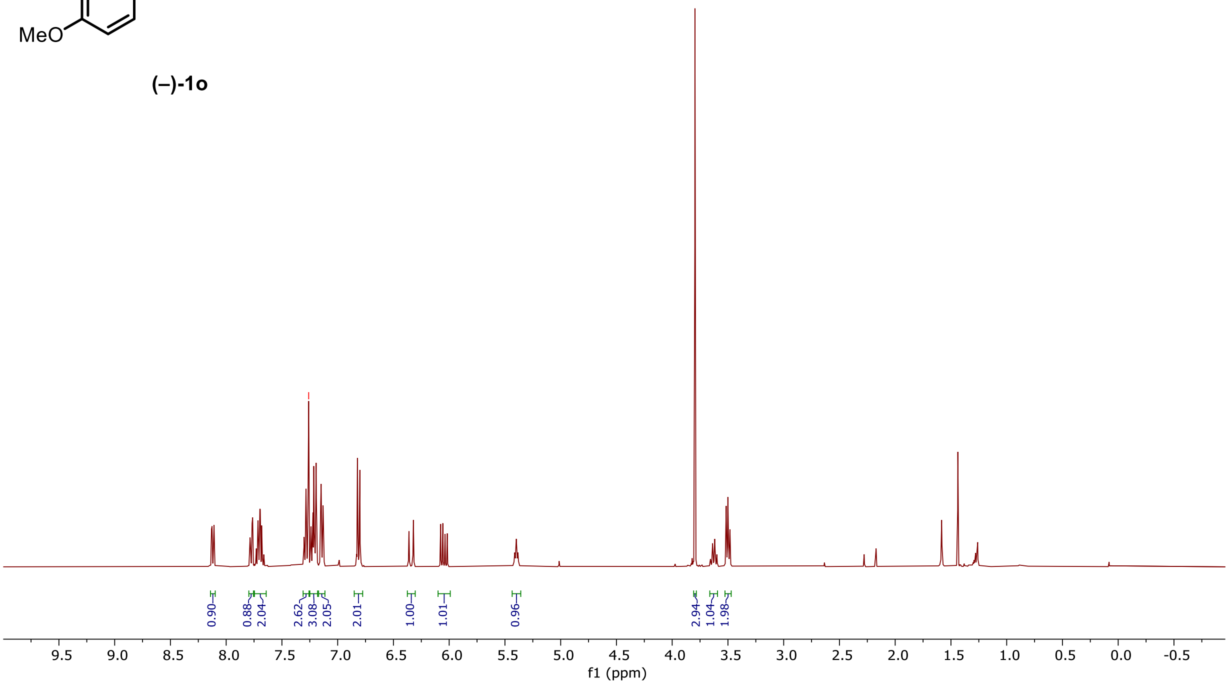

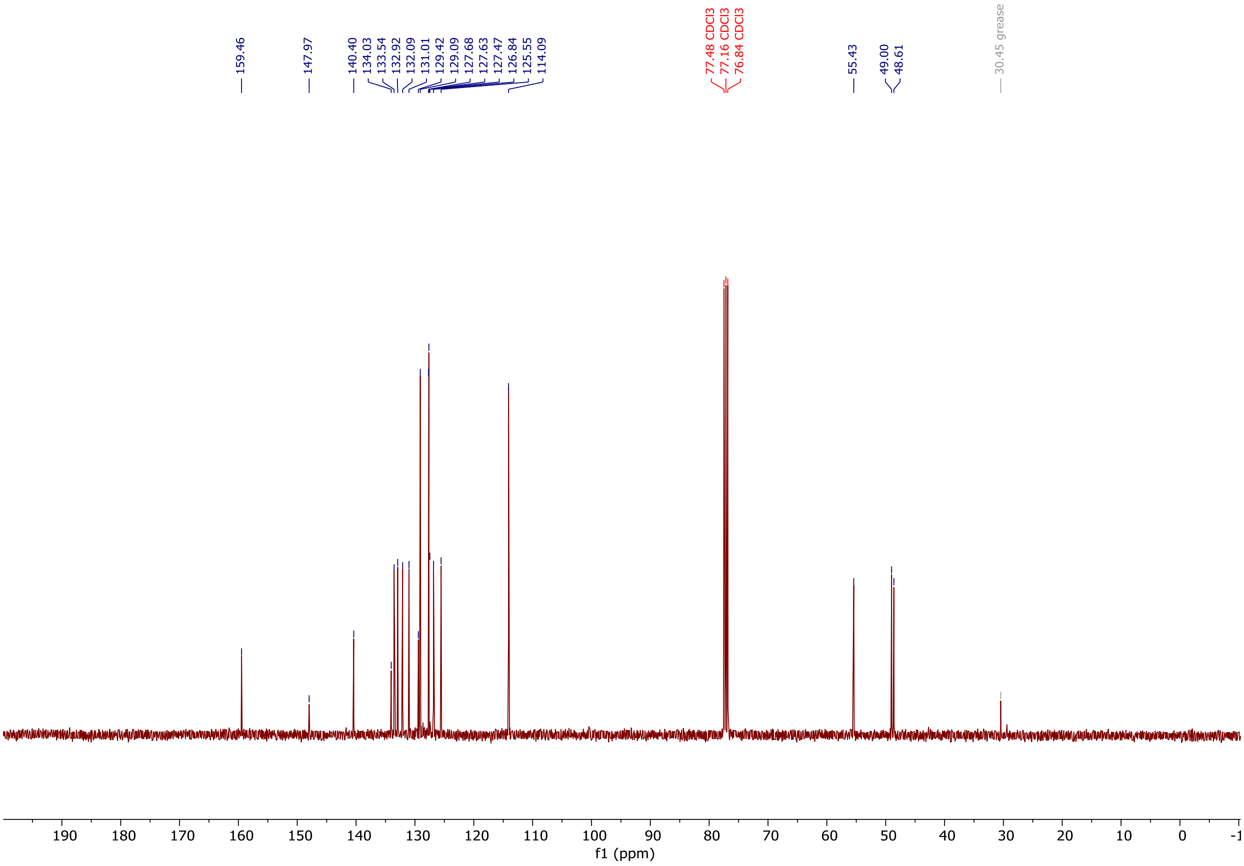

(4-Ms): (*E*)-*N*-(5-(4-Methoxyphenyl)pent-4-en-1-yl)methanesulfonamide

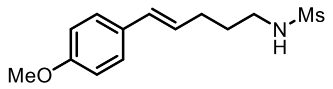

4-Ms

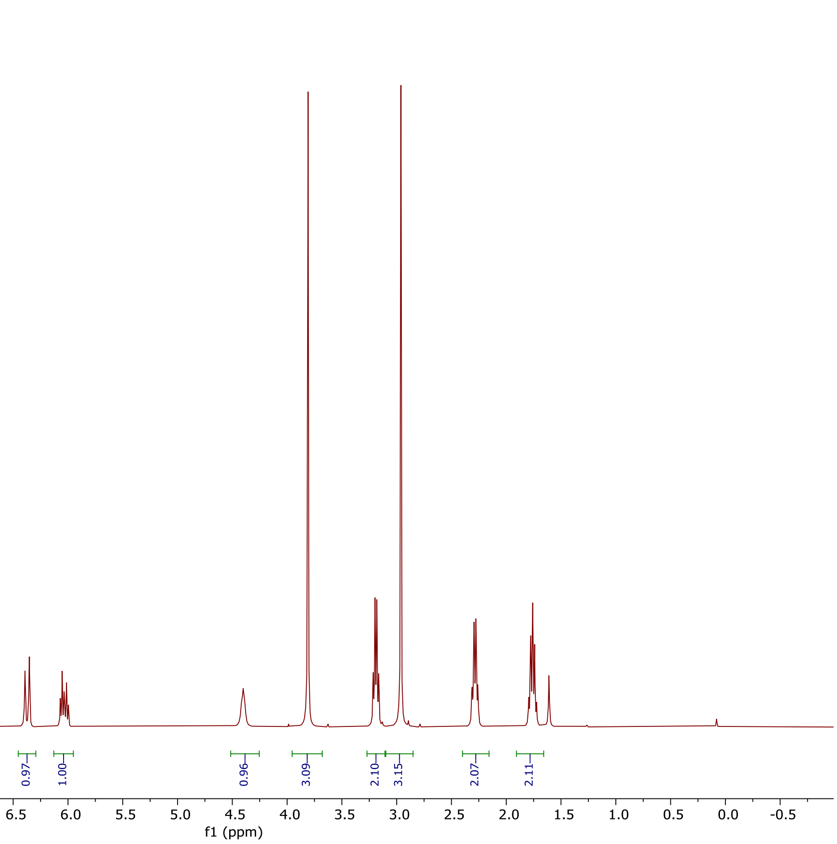

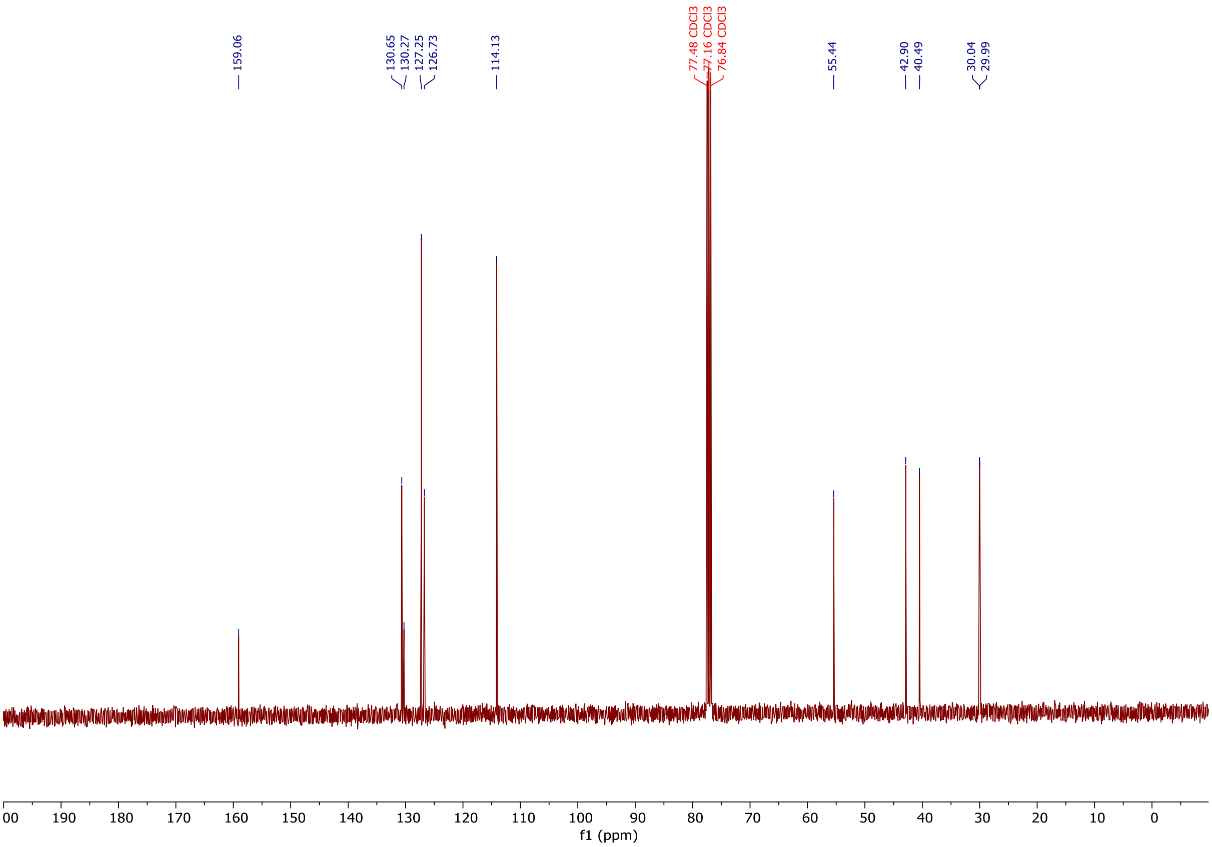

(4-*o*Ns): (*E*)-*N*-(5-(4-Methoxyphenyl)pent-4-en-1-yl)-2-nitrobenzenesulfonamide

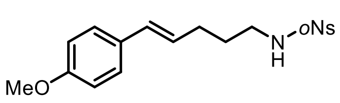

4-oNs

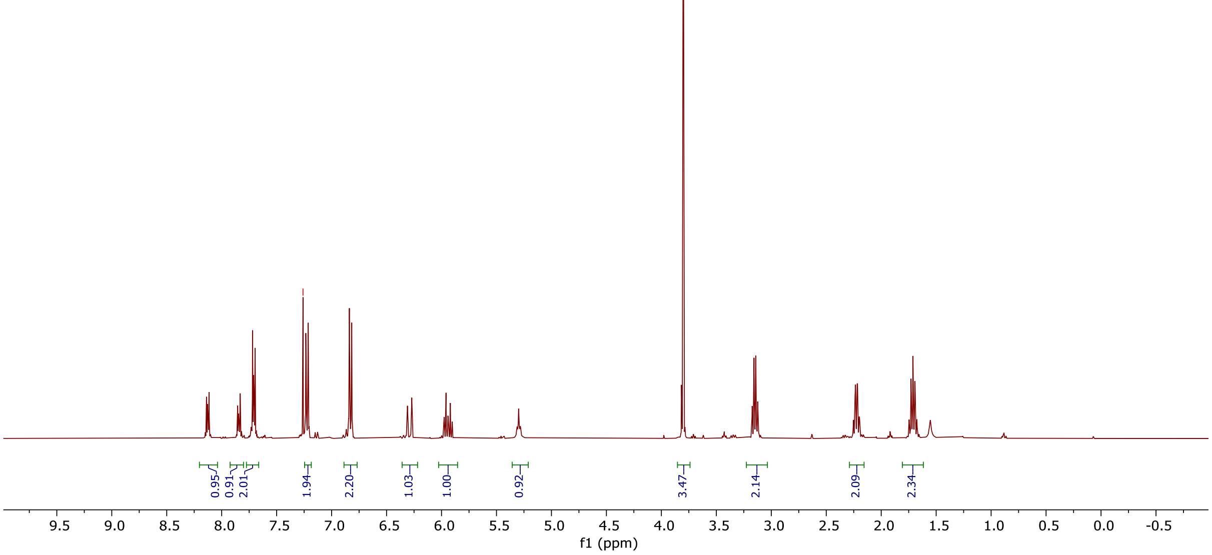

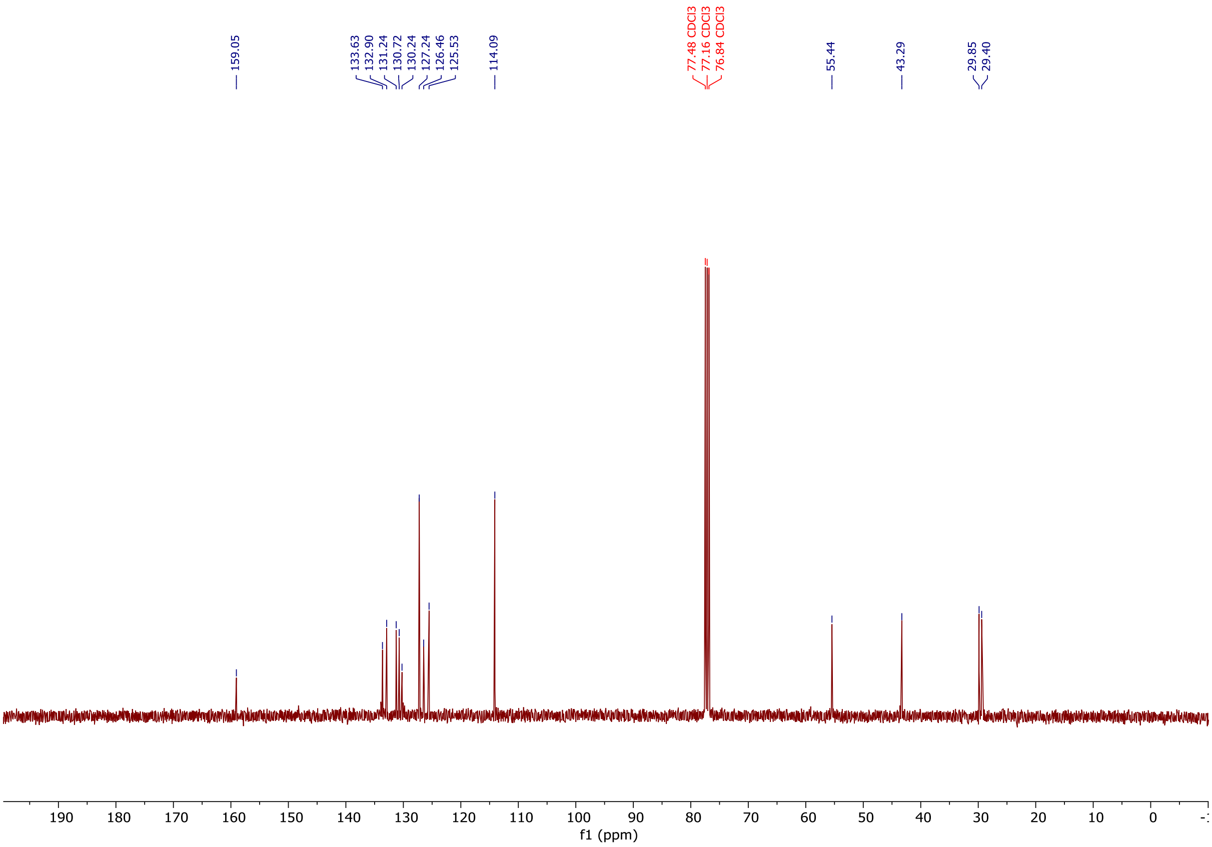

(S18): (*E*)-5-(4-Methoxyphenyl)pent-4-enamide

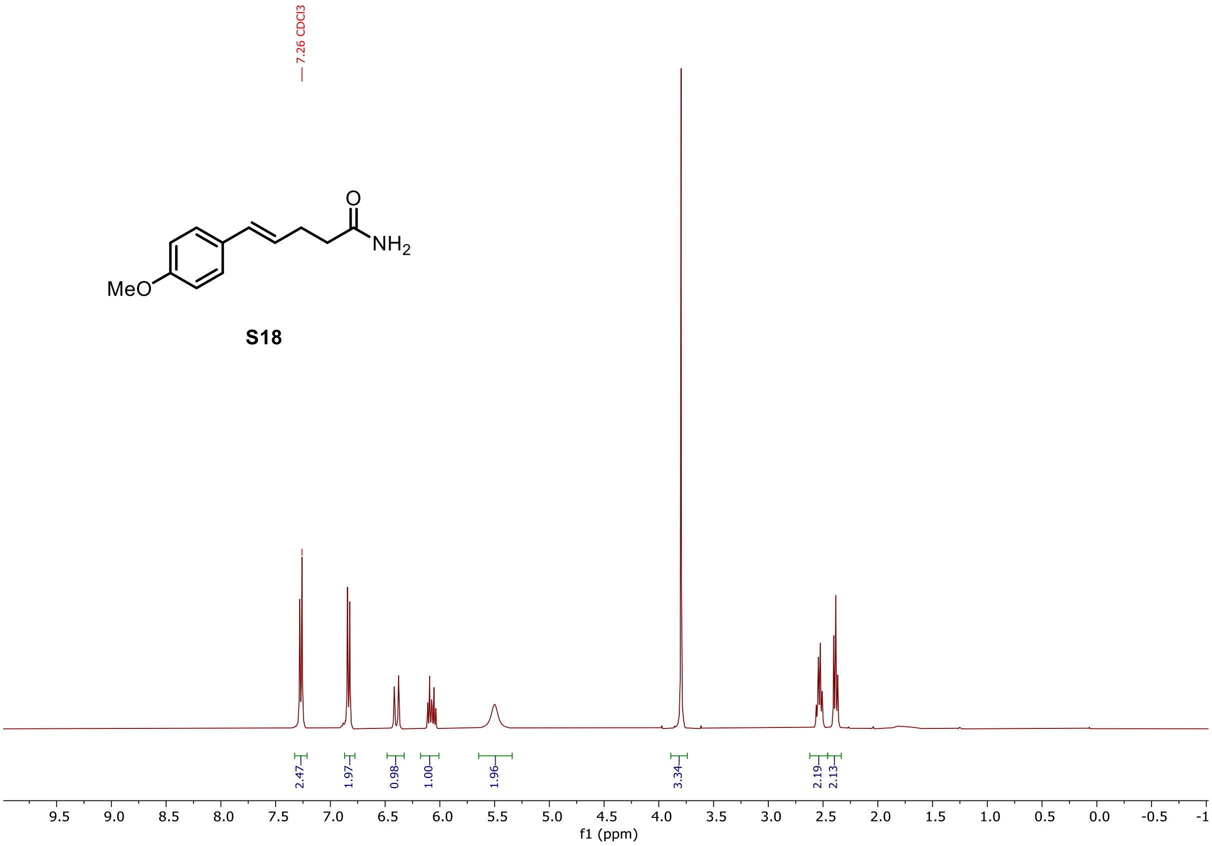

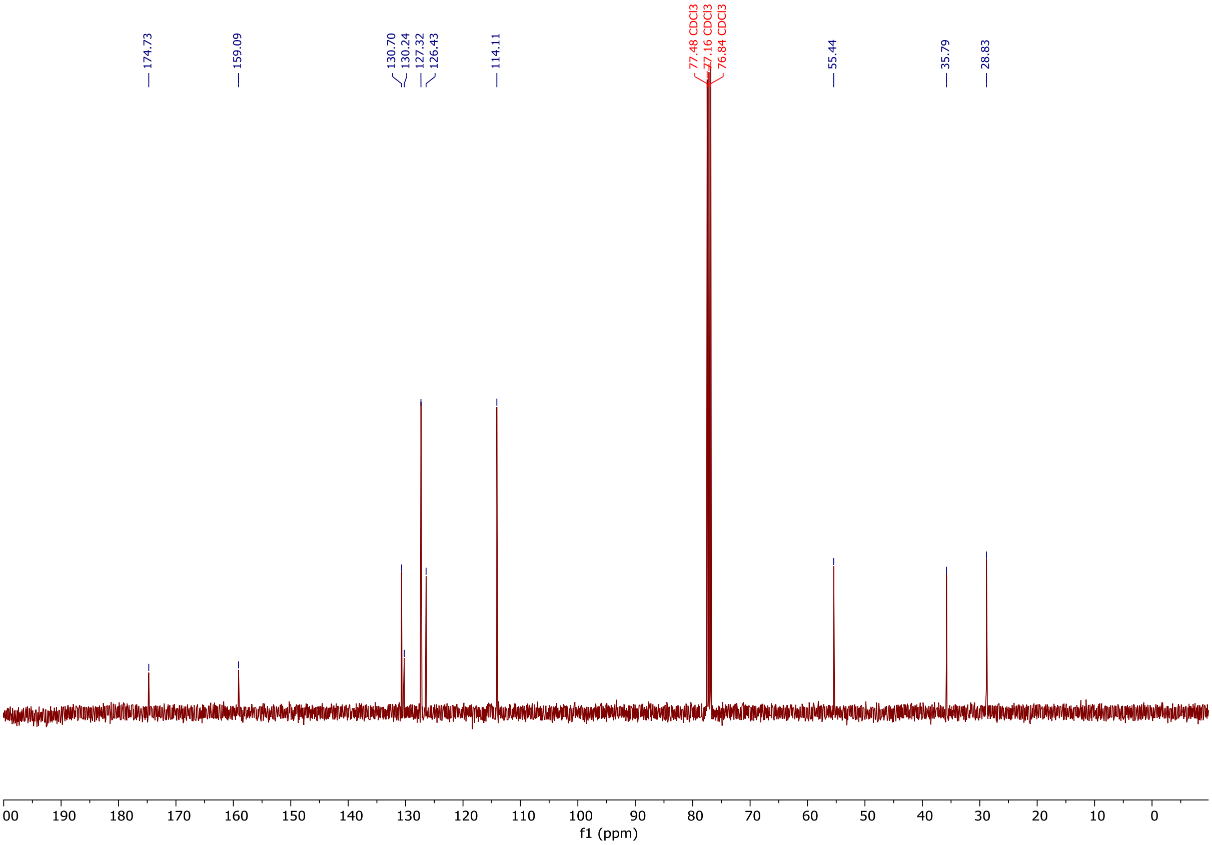

(4-Cbz): Benzyl (*E*)-(5-(4-methoxyphenyl)pent-4-en-1-yl)carbamate

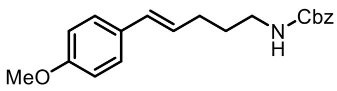

4-Cbz

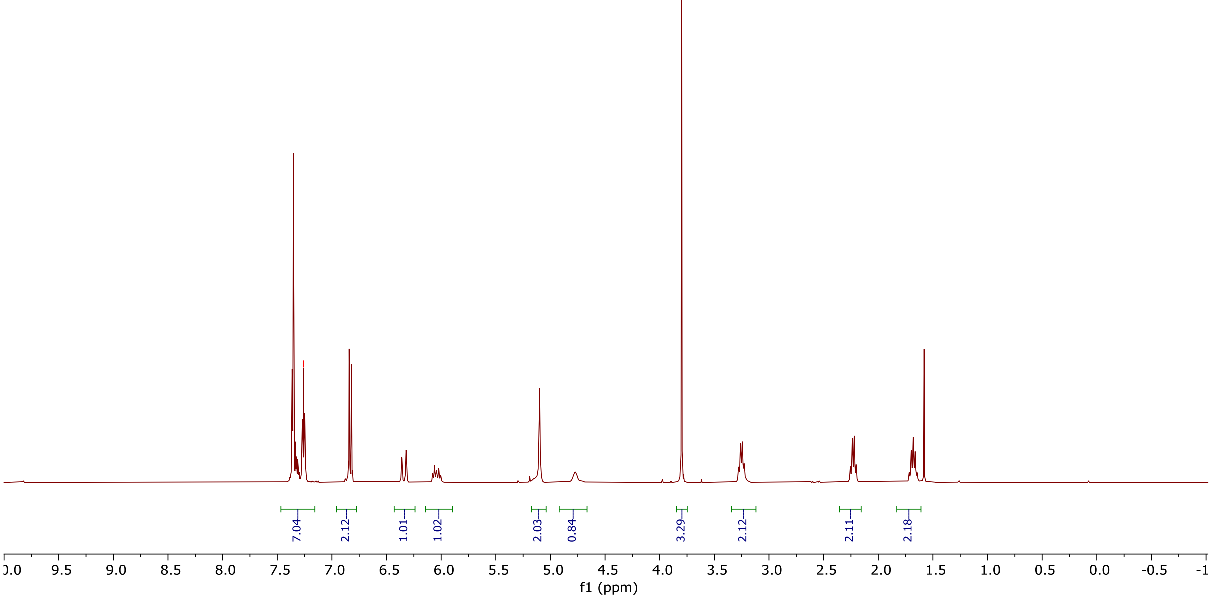

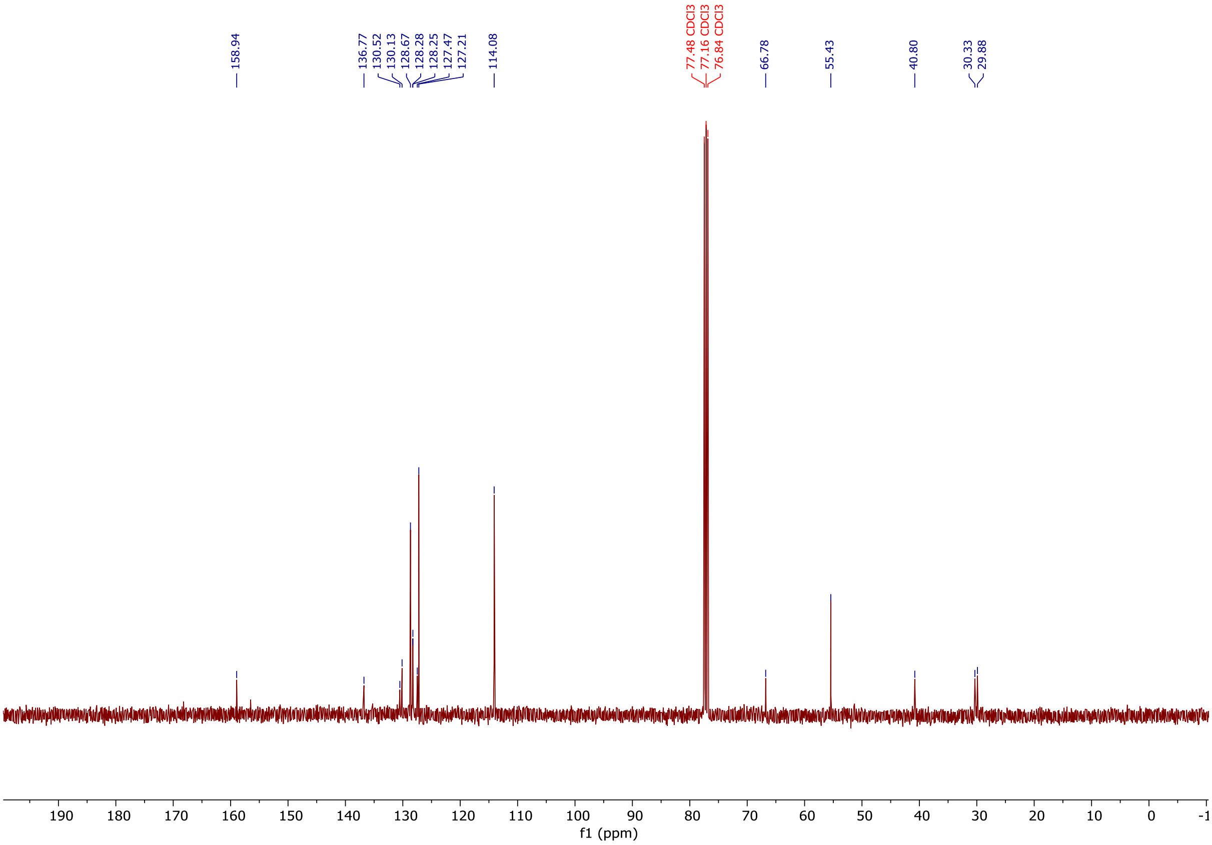

(4a): Methyl (*E*)-(5-(4-methoxyphenyl)pent-4-en-1-yl)carbamate

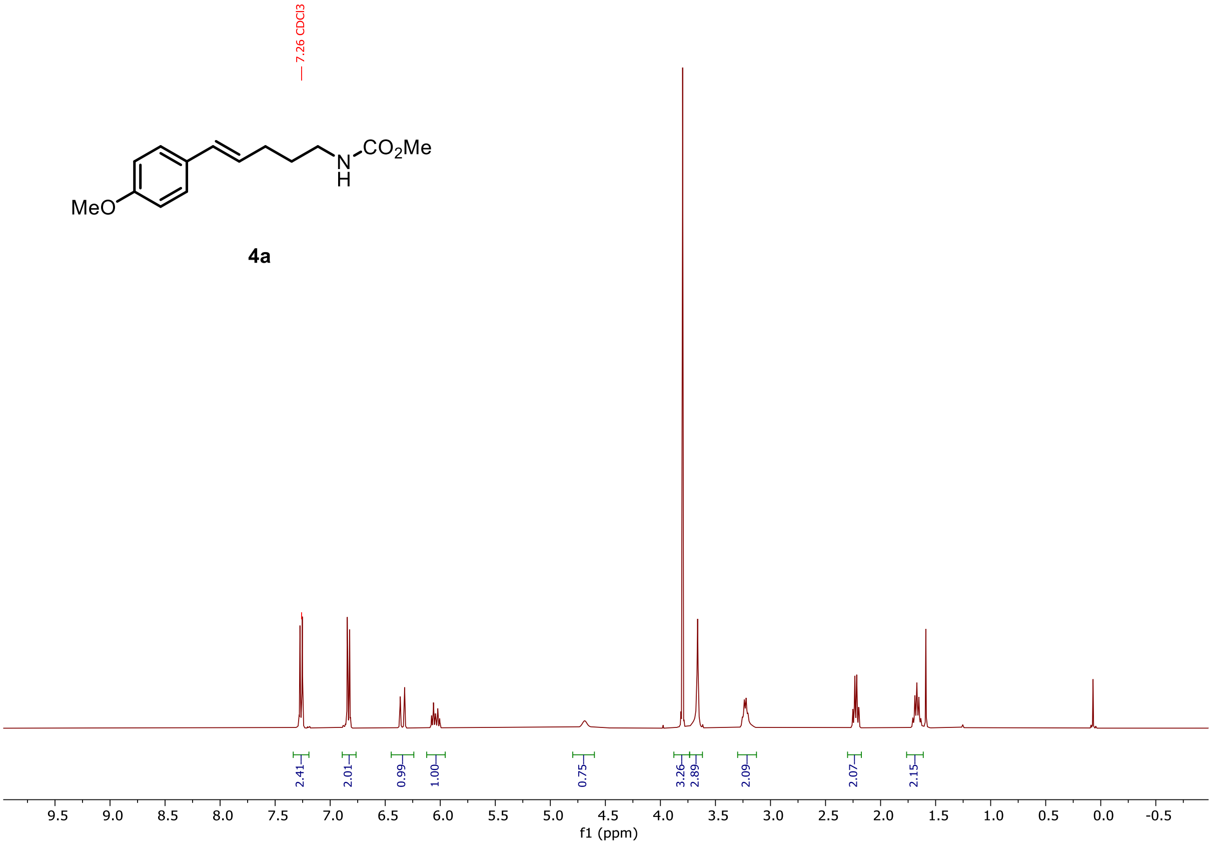

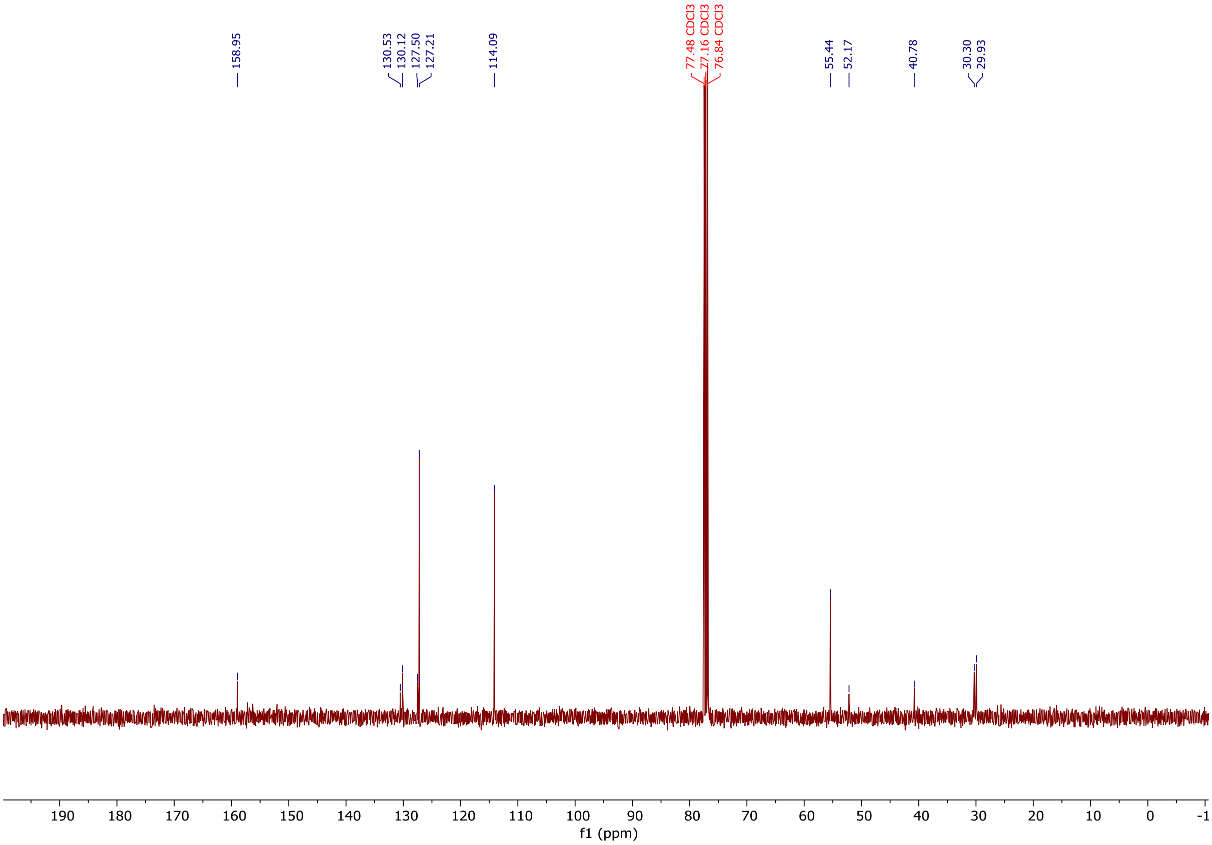

(4b): Methyl (*E*)-(5-phenylpent-4-en-1-yl)carbamate

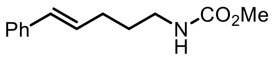

4b

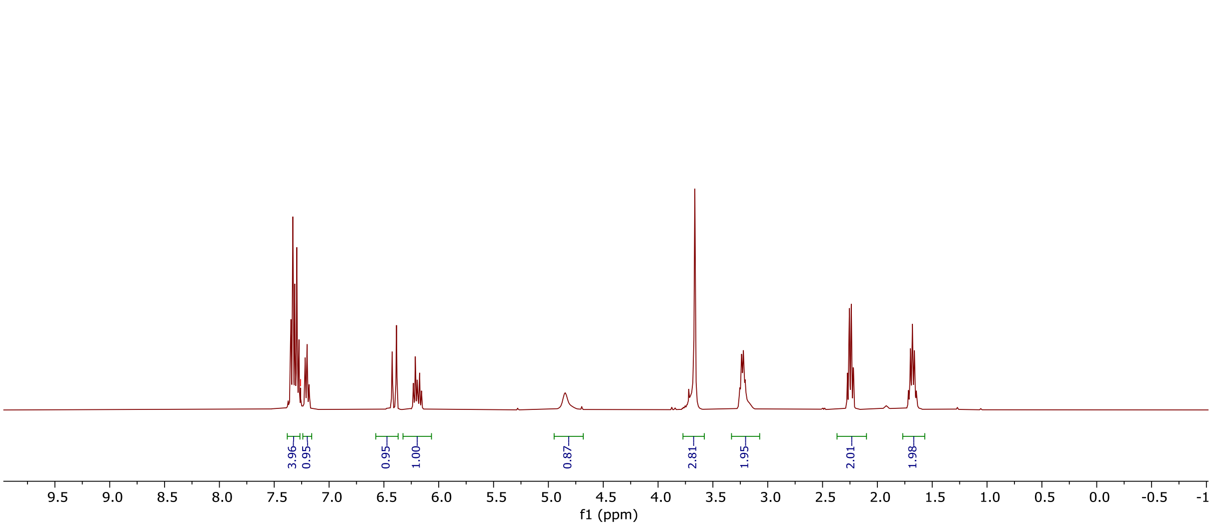

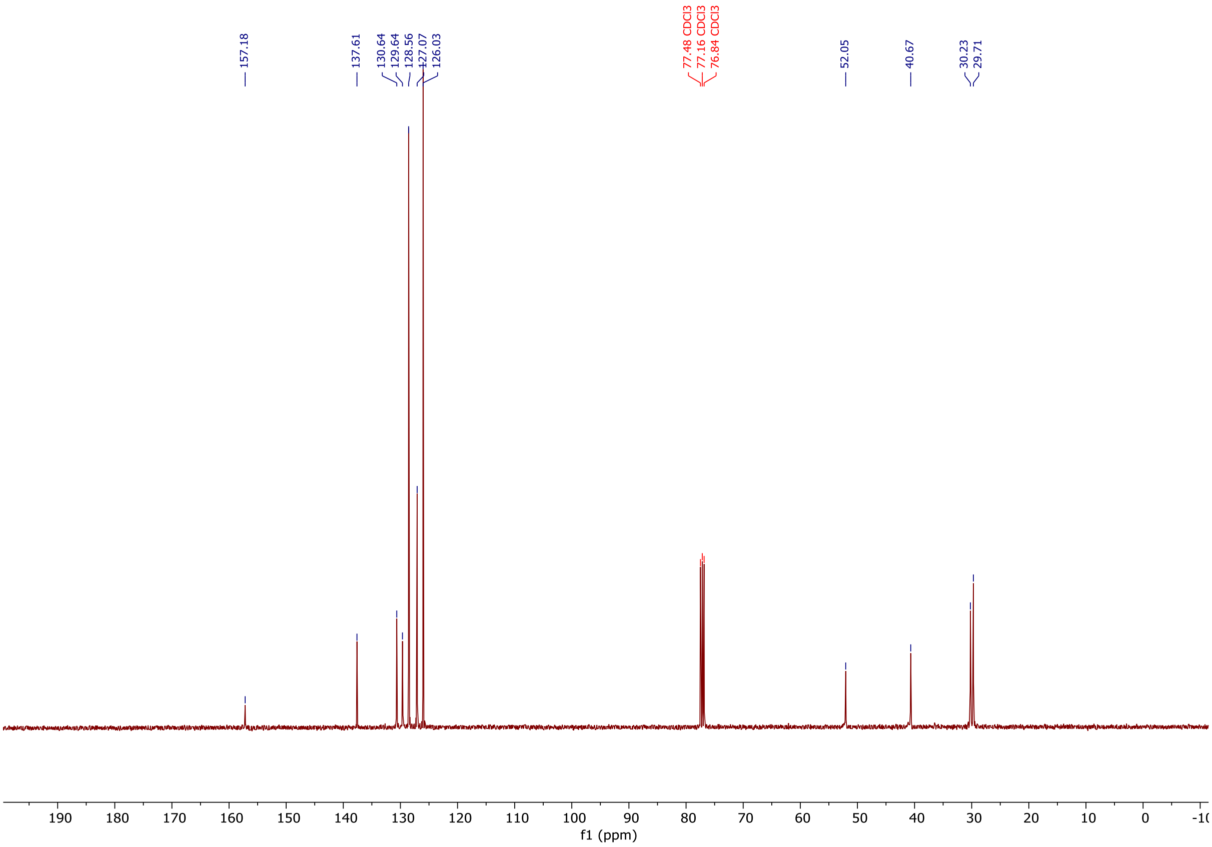

(4b-Ns): (*E*)-2-Nitro-*N*-(5-phenylpent-4-en-1-yl)benzenesulfonamide

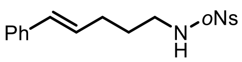

4b-Ns

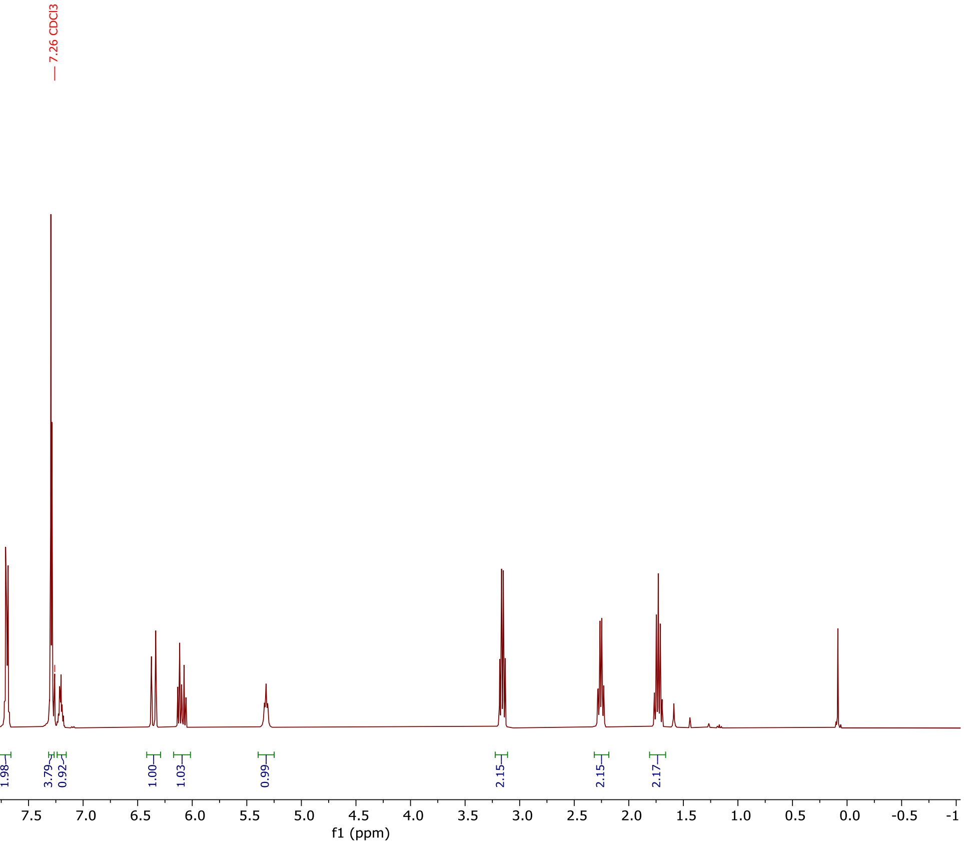

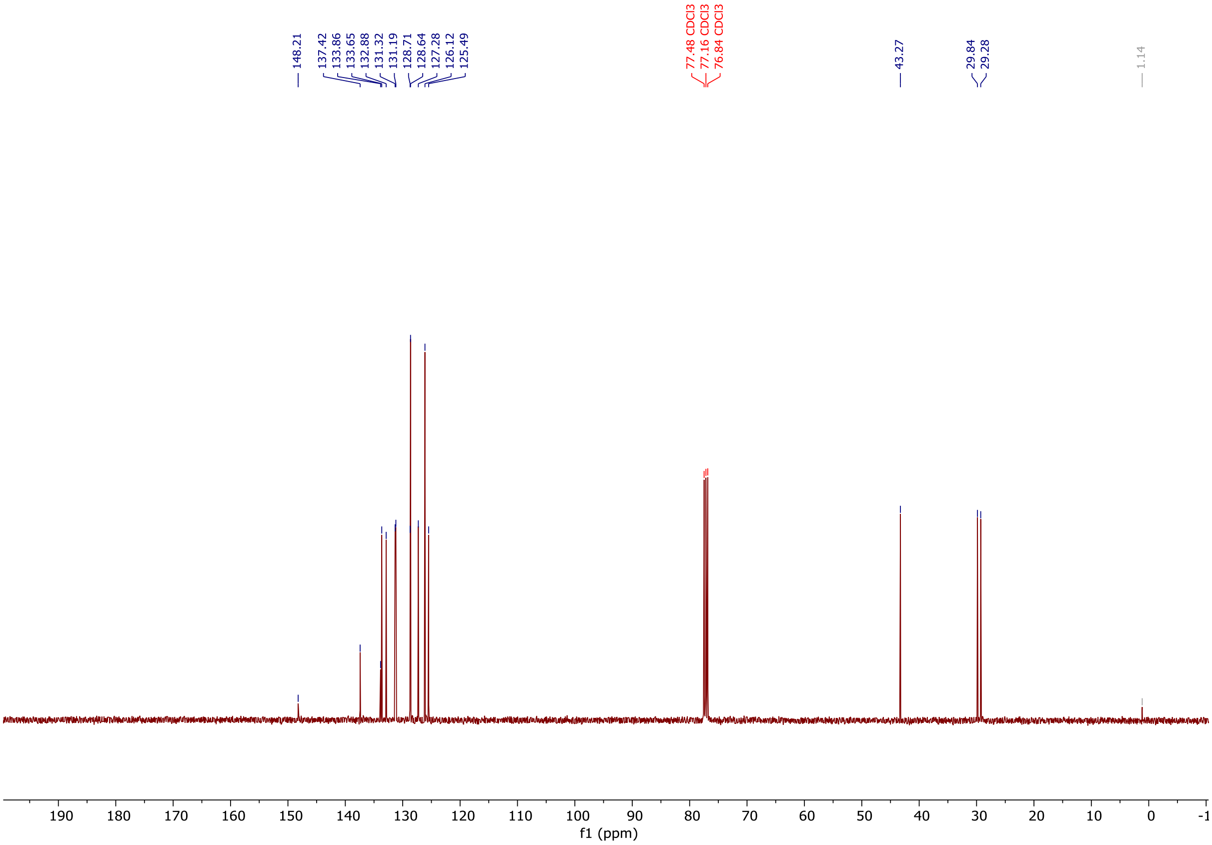

(S24): Methyl pent-4-en-1-ylcarbamate

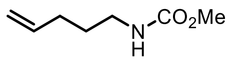

S24

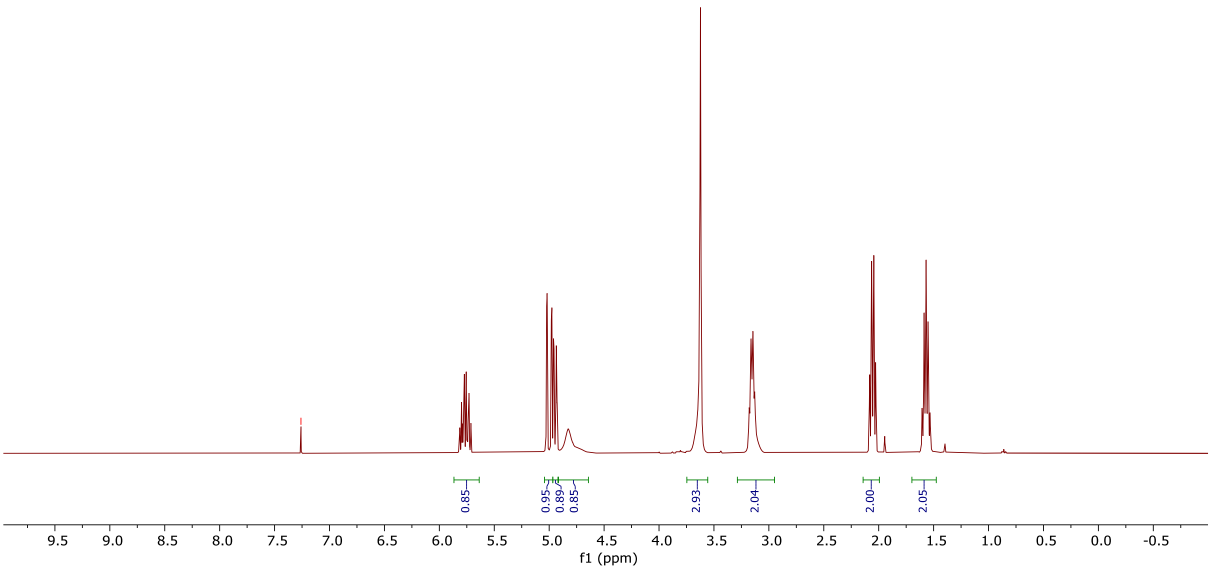

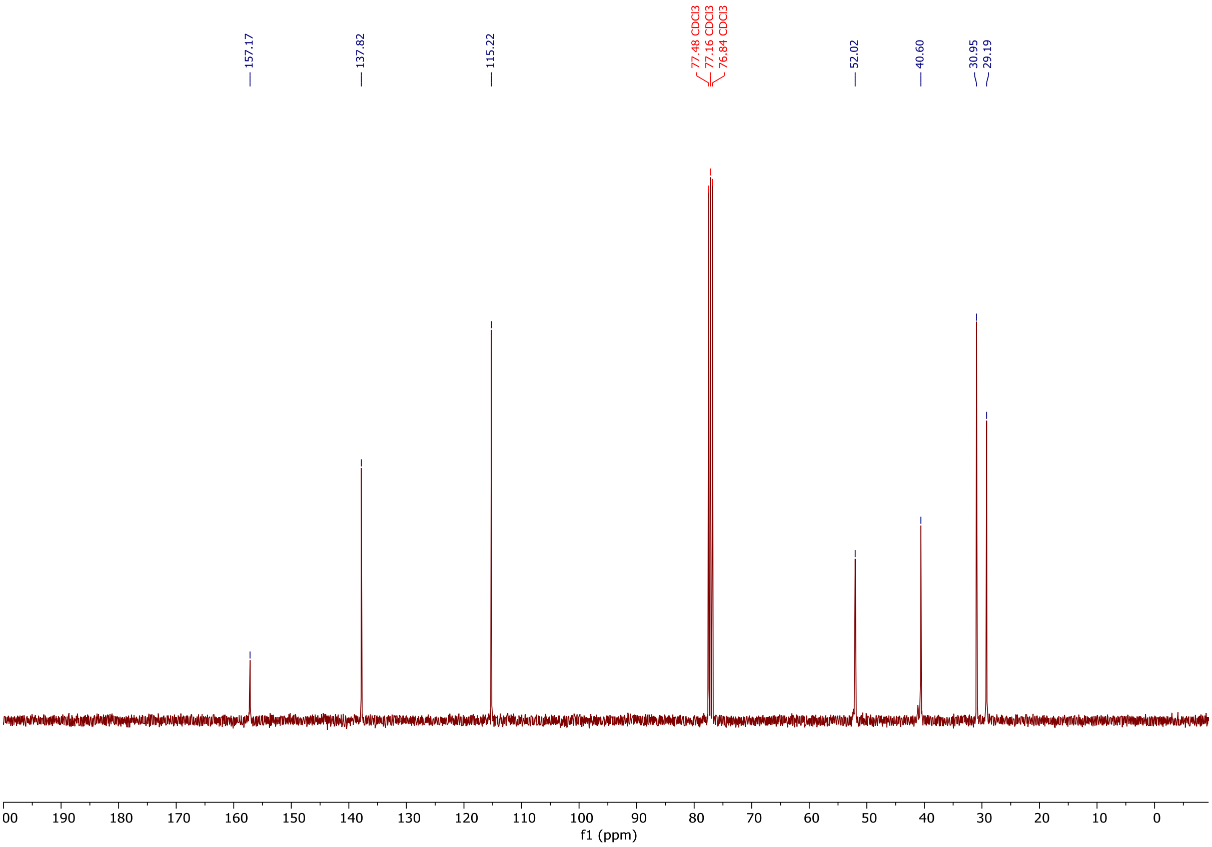

(4c): Methyl (*E*)-(5-(4-bromophenyl)pent-4-en-1-yl)carbamate

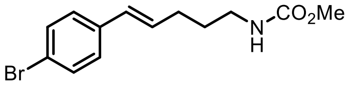

4c

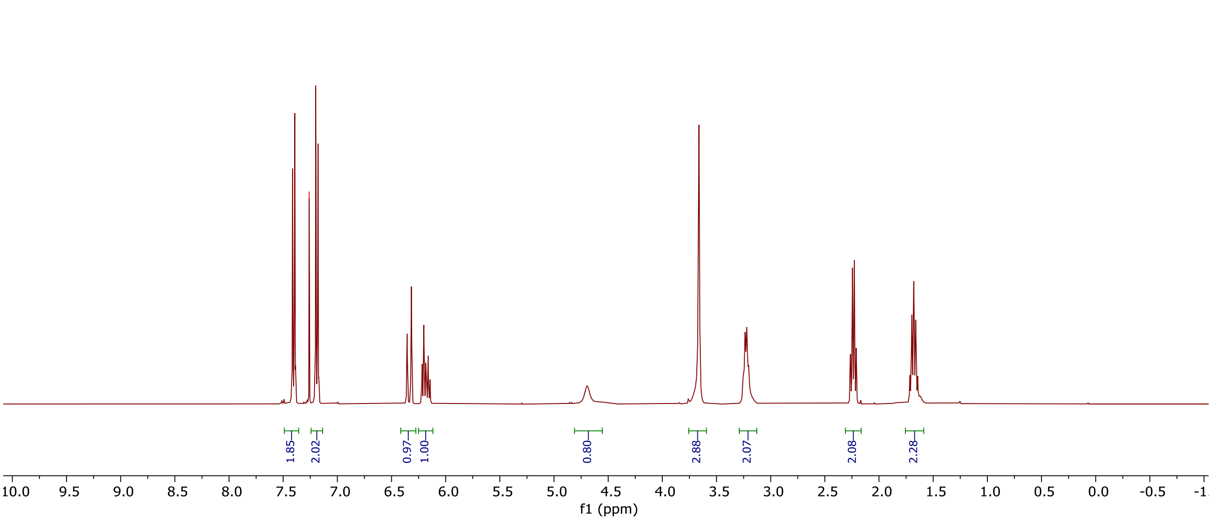

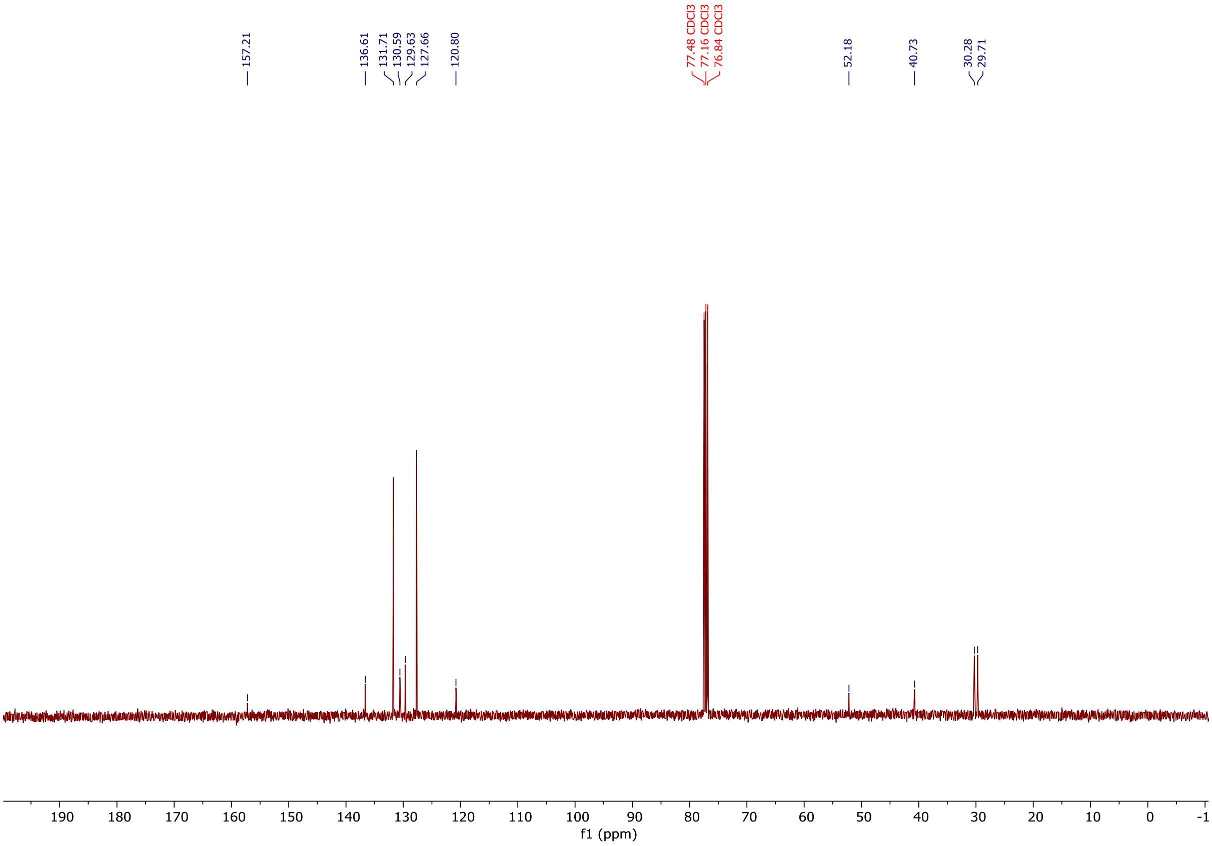

(4d): Methyl ((4*E*,6*E*)-7-(4-methoxyphenyl)hepta-4,6-dien-1-yl)carbamate

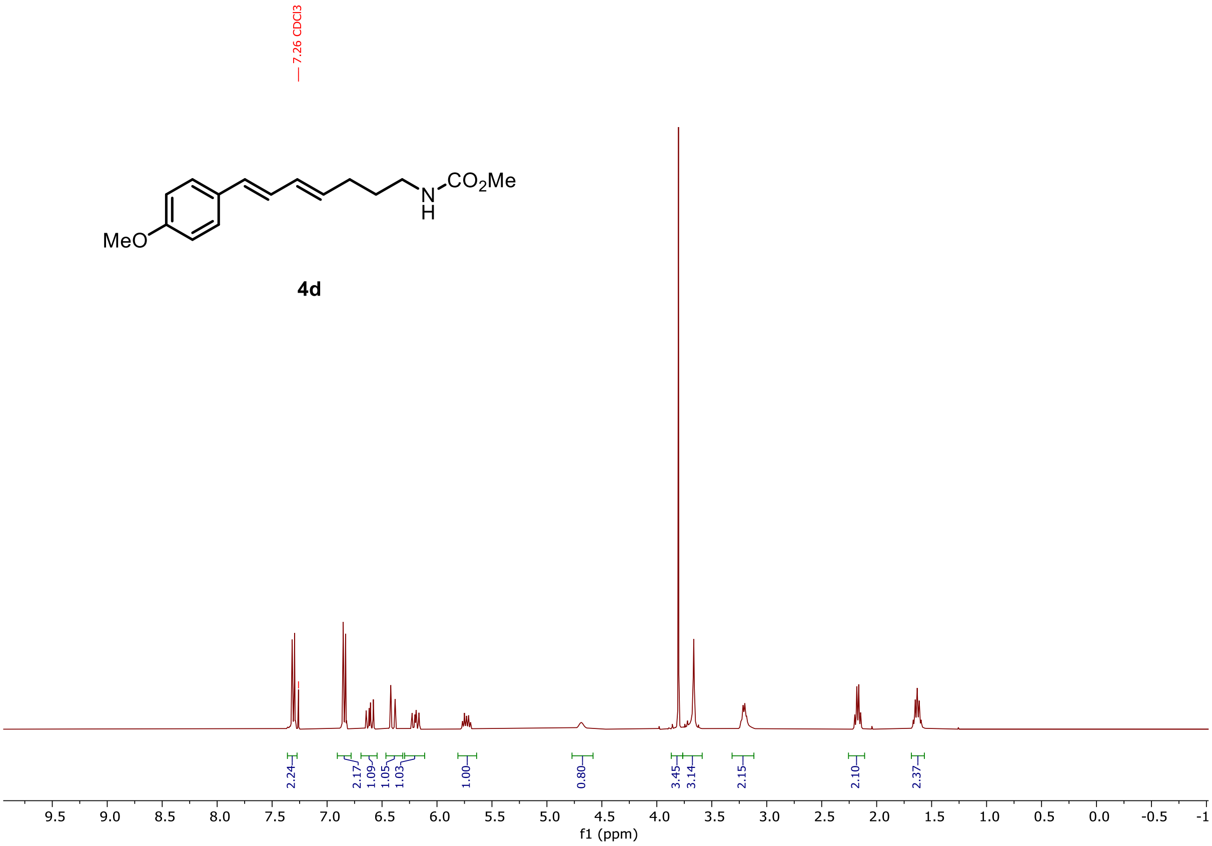

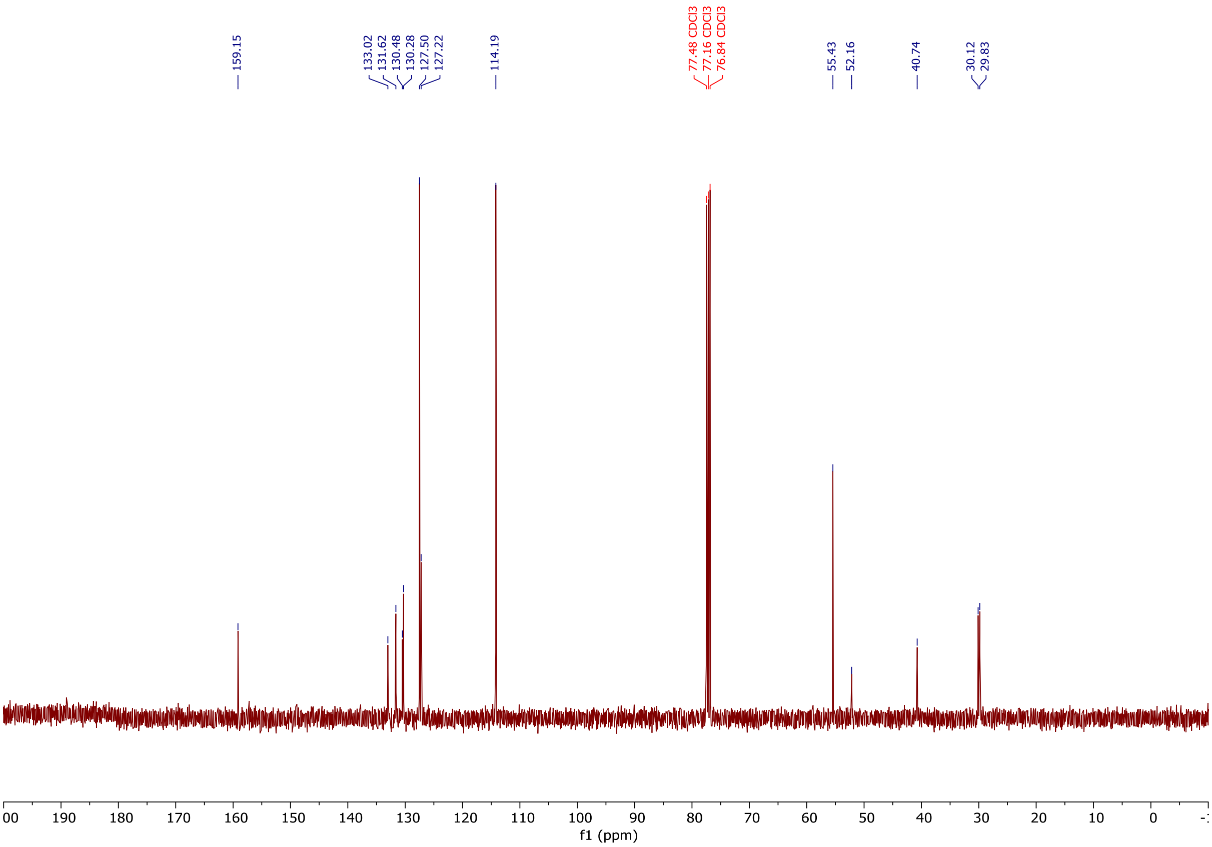

(S26): 5-Methylhex-4-enamide

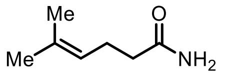

S26

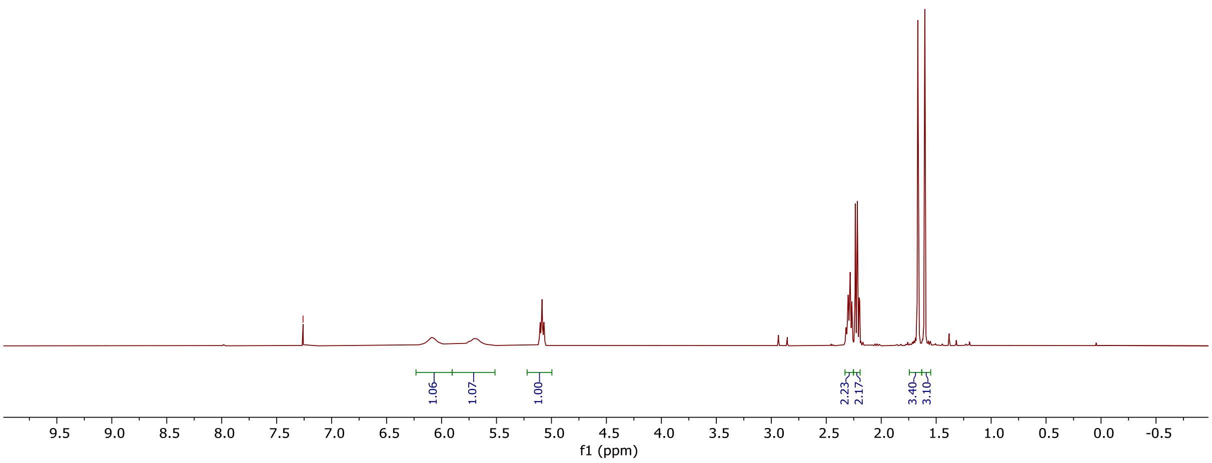

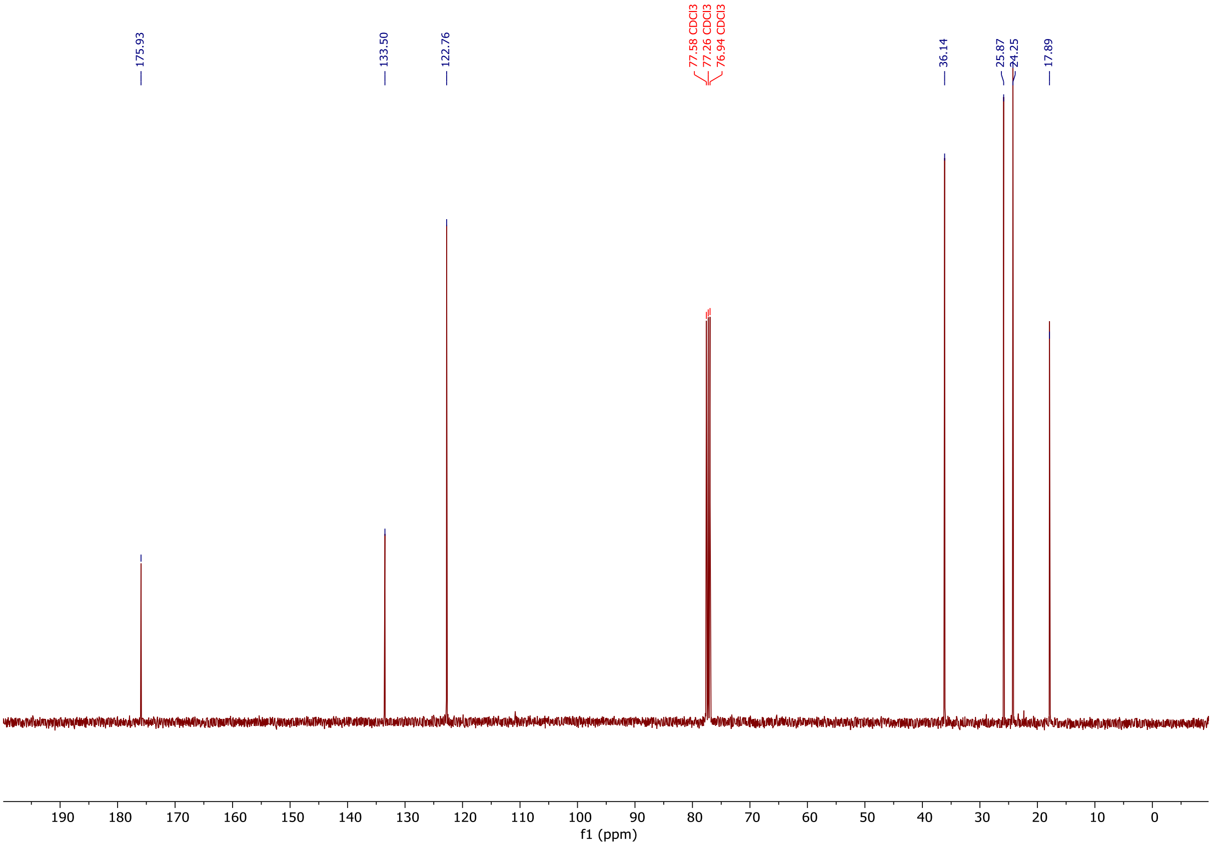

(4e): Methyl (5-methylhex-4-en-1-yl) carbamate

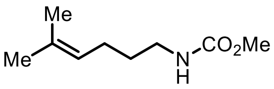

4e

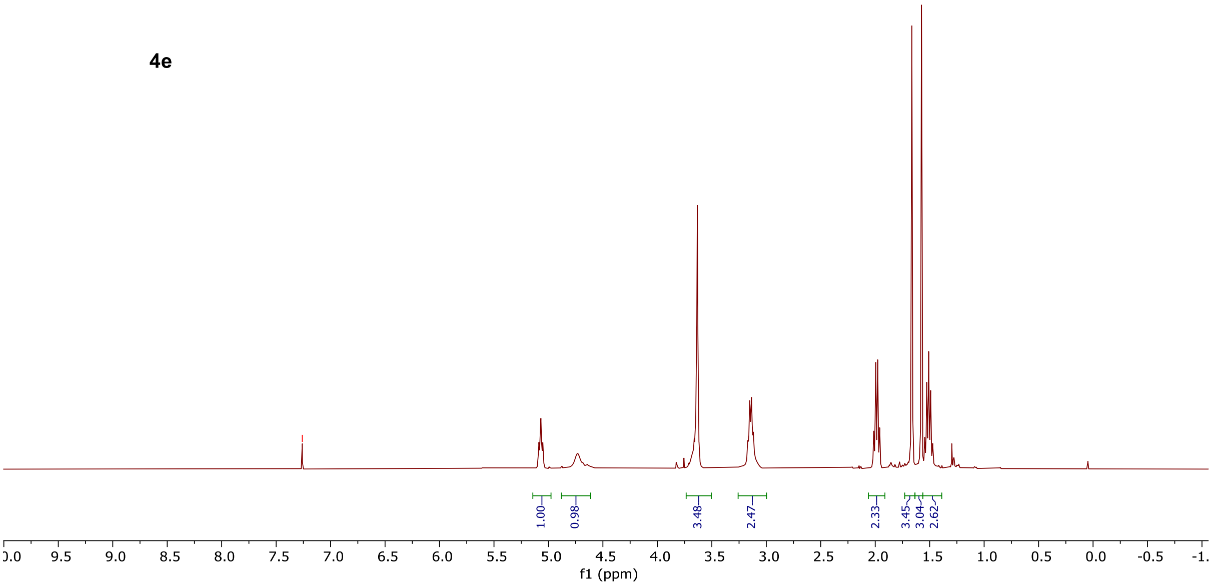

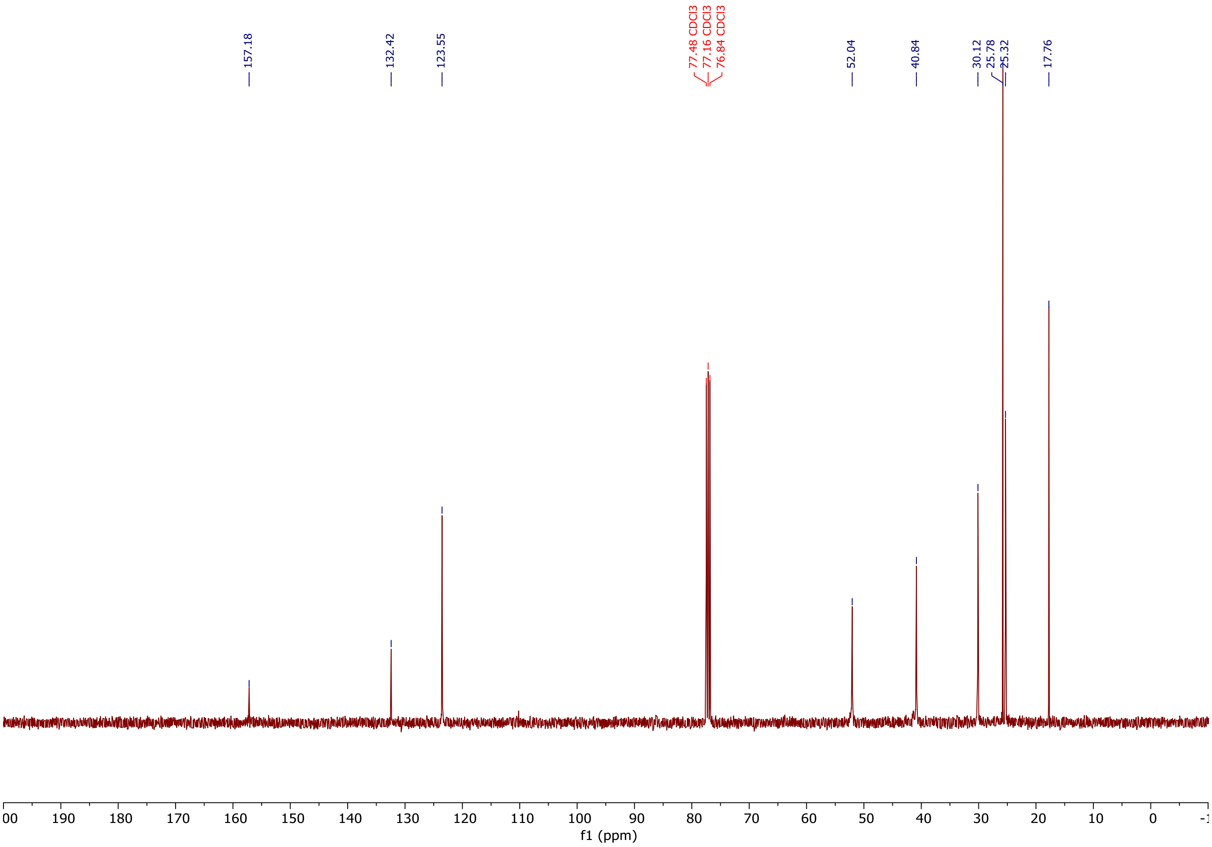

(3a): (±)-(2*S*,3*R*)-3-Benzhydryl-2-(4-methoxyphenyl)-1-tosylpyrrolidine

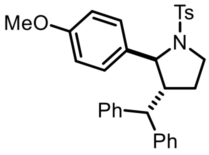

3a

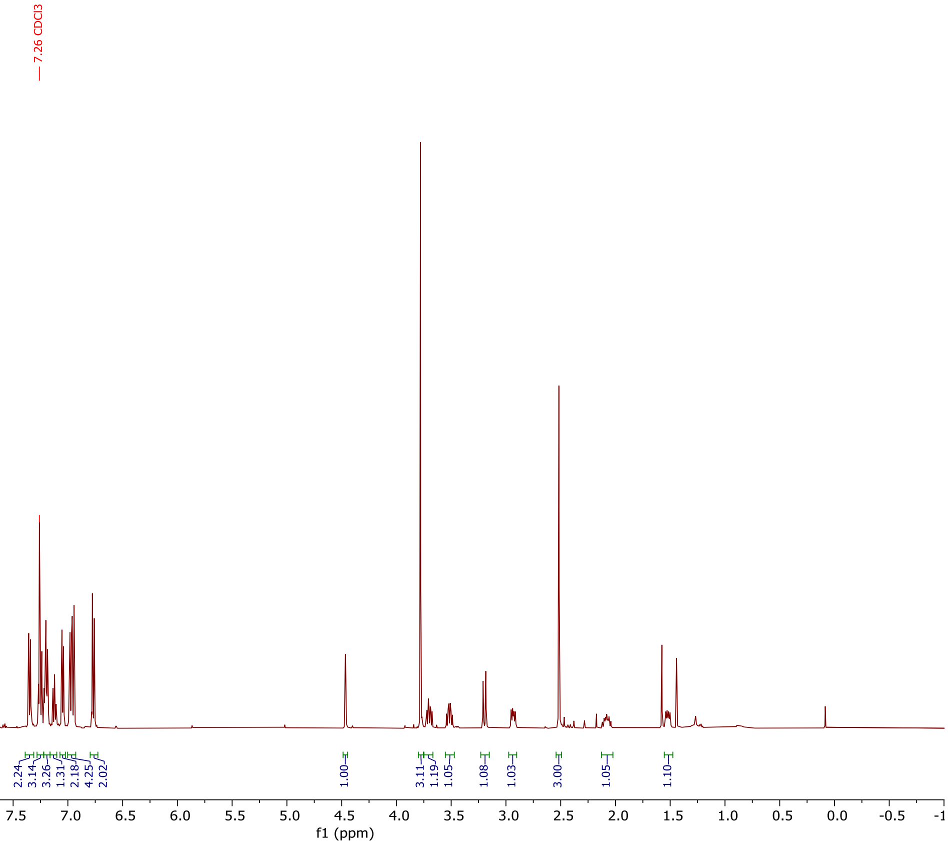

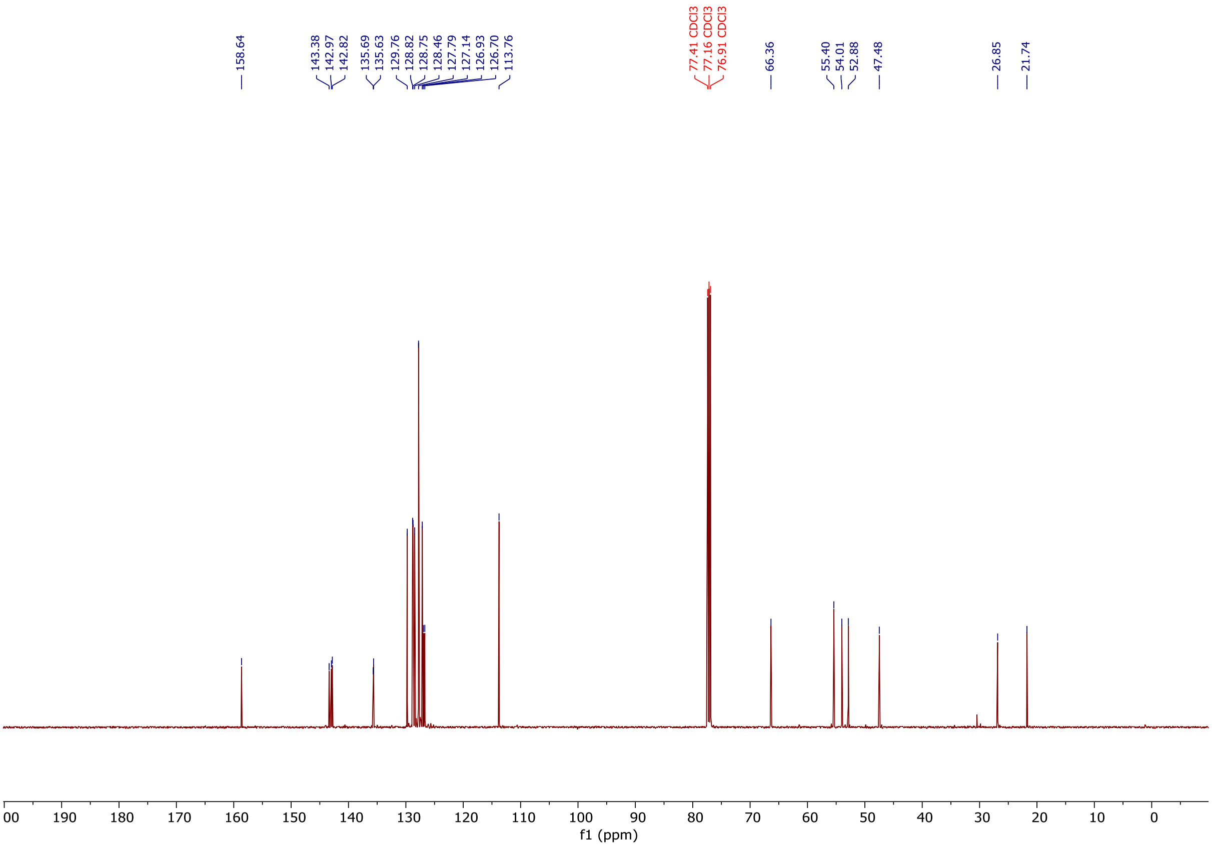

(3b): (±)-(2*S*,3*R*)-3-Benzhydryl-2-(4-methoxyphenyl)-1-(methylsulfonyl)pyrrolidine

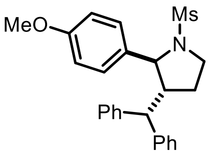

3b

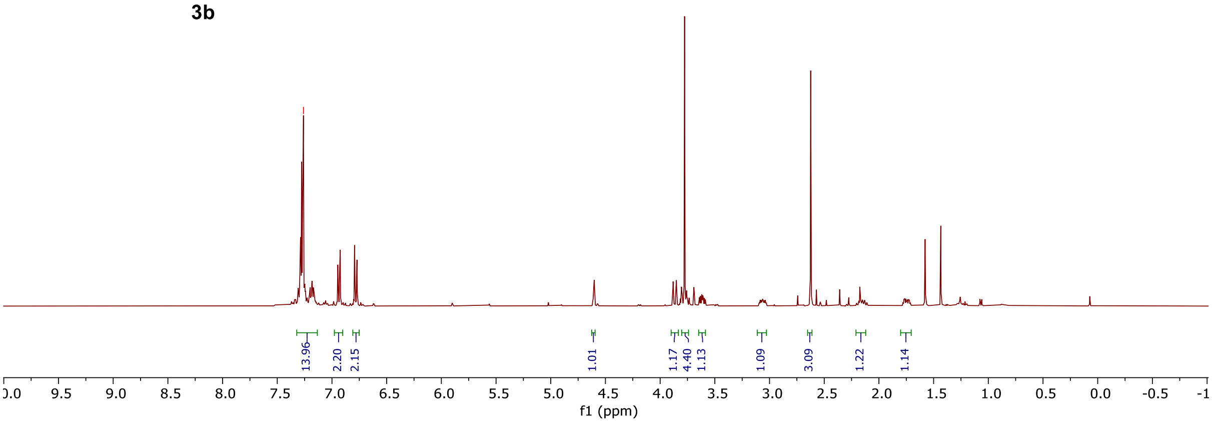

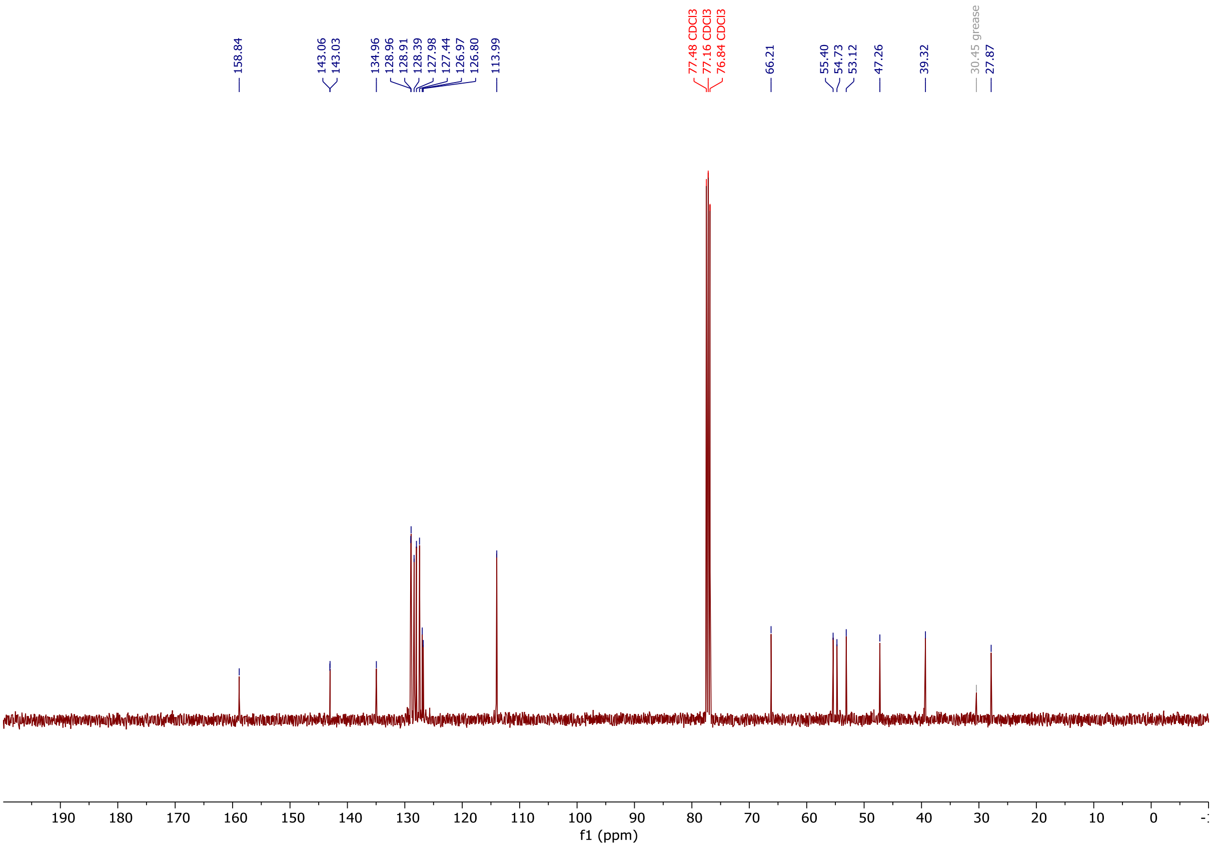

(3c): (±)-(2*S*,3*R*)-3-Benzhydryl-2-(4-methoxyphenyl)-1-[(2-nitrophenyl)sulfonyl]pyrrolidine

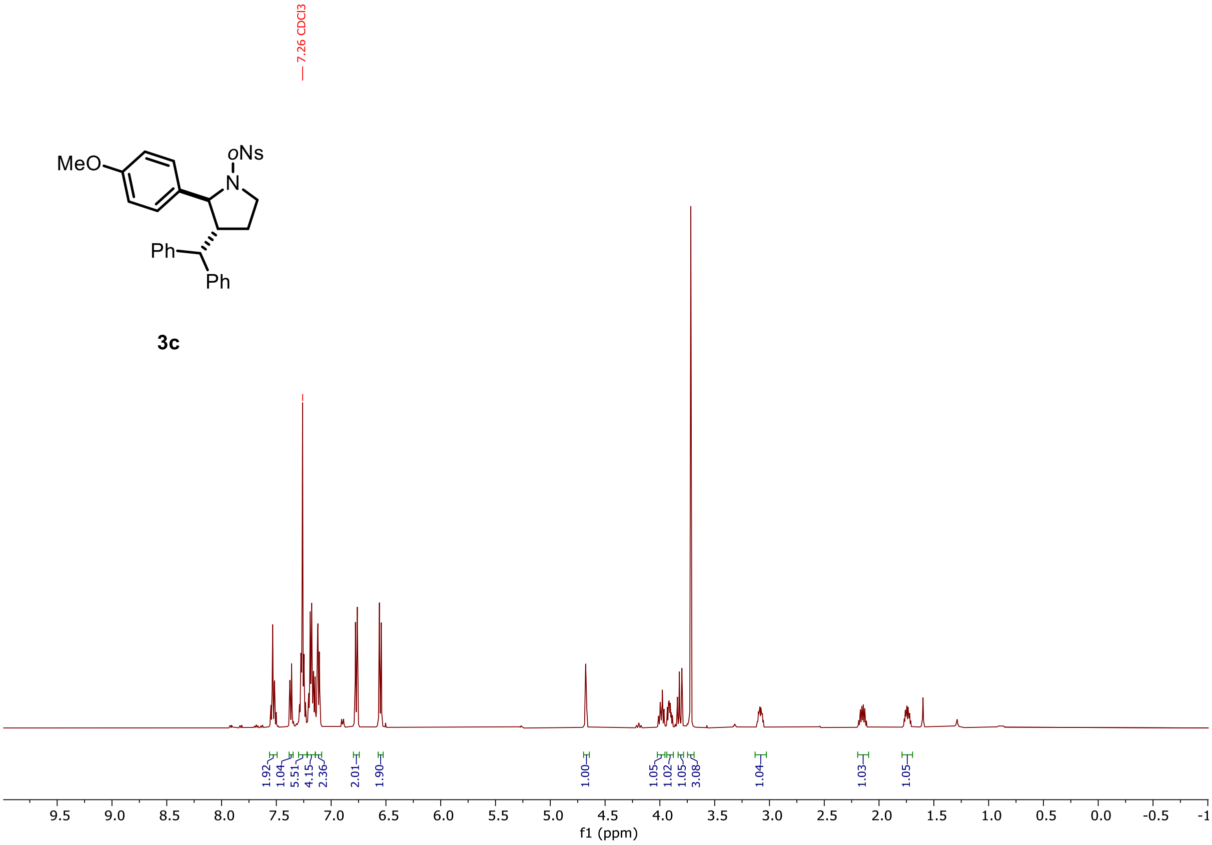

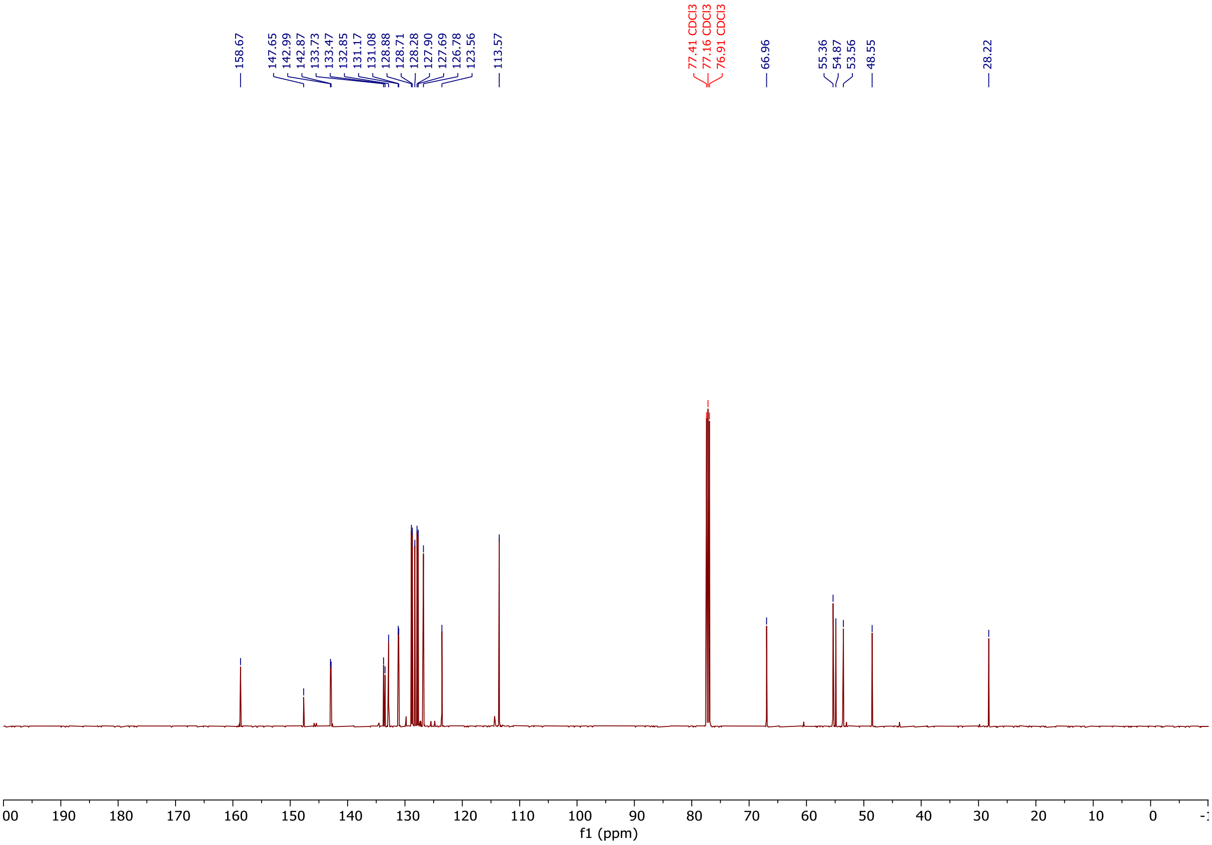

(3d): (±)-(2*S*,3*R*)-3-Benzhydryl-2-(4-methoxyphenyl)-1-[(4-nitrophenyl)sulfonyl]-pyrrolidine

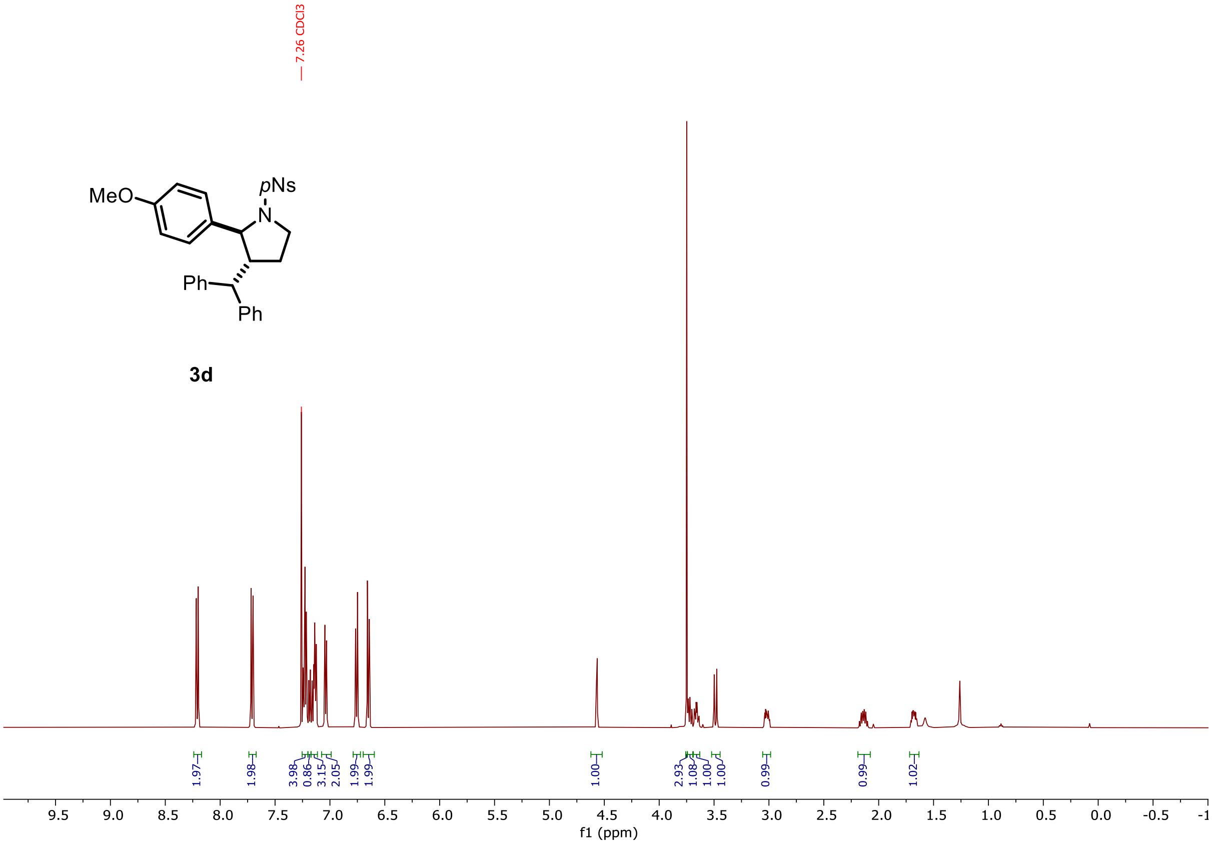

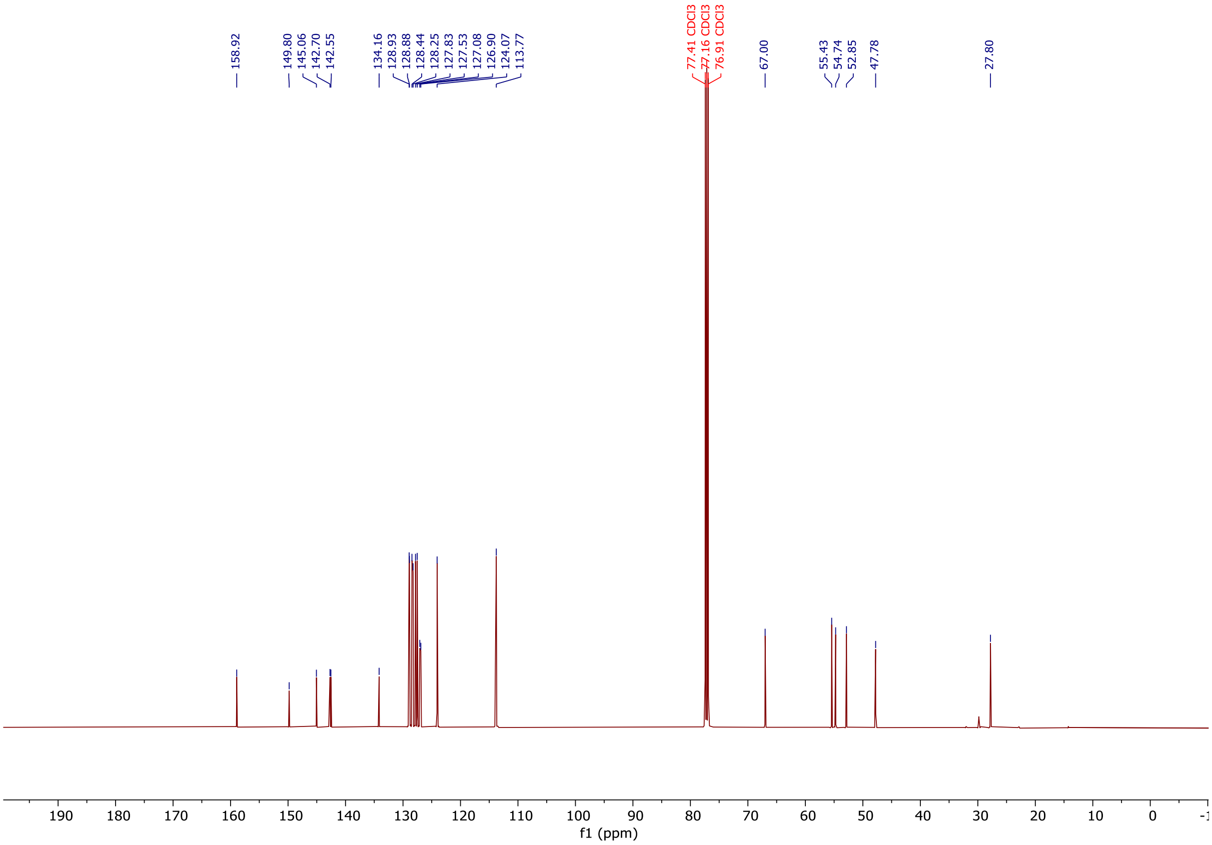

(3e): (±)-Benzyl (2*S*,3*R*)-3-benzhydryl-2-(4-methoxyphenyl)pyrrolidine-1-carboxylate

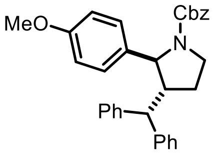

3e

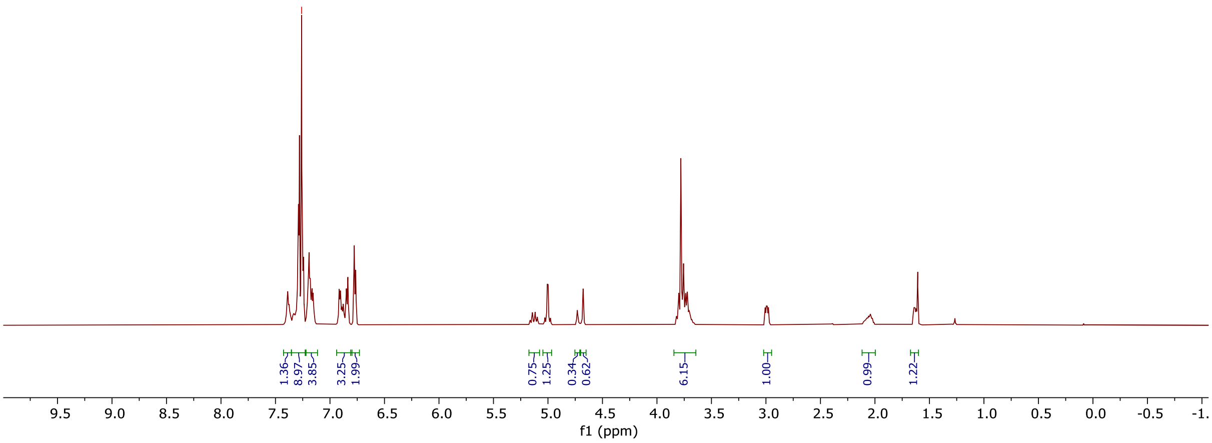

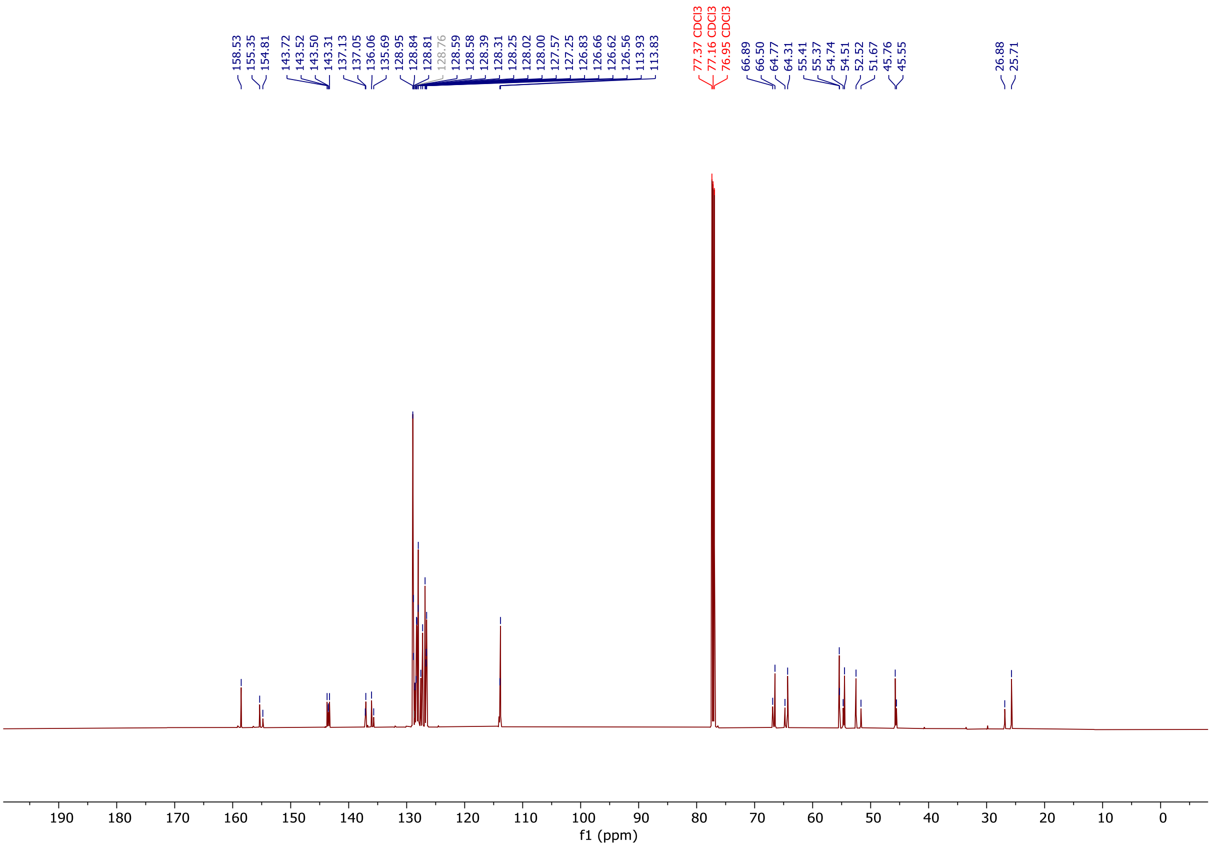

(3g): (±)-(2*S*,3*R*)-2-(4-Methoxyphenyl)-1-[(2-nitrophenyl)sulfonyl)-3-[(1'*R*)-3-phenylcyclopent-2-en-1-yl]pyrrolidine

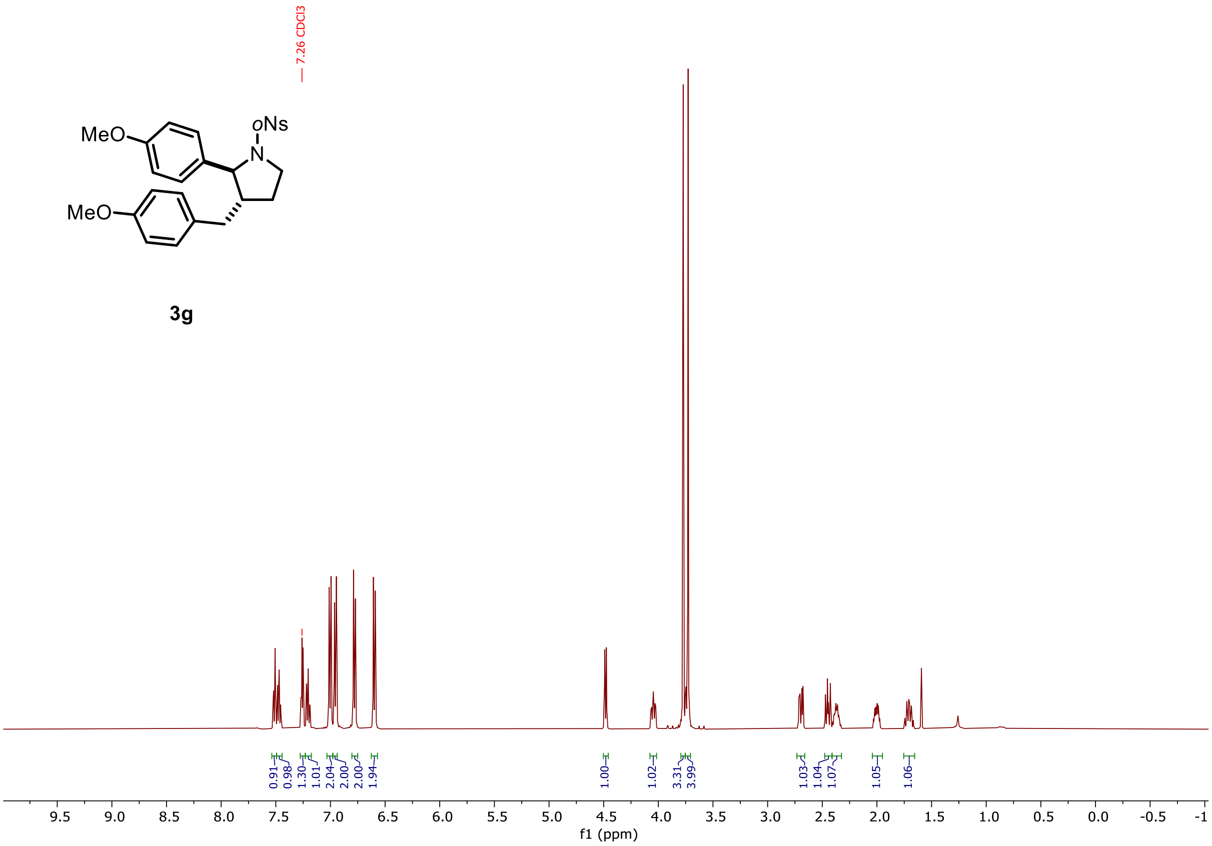

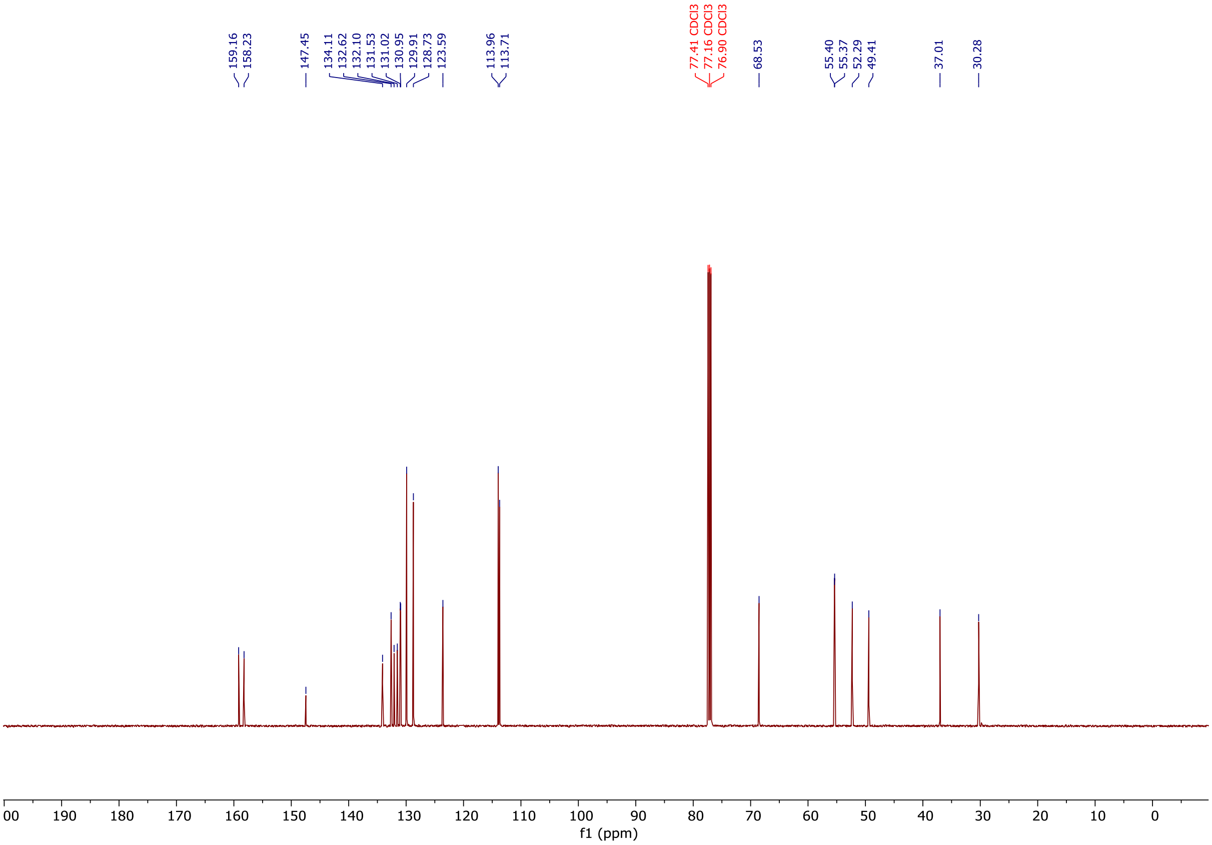

(3h): (±)-(2*S*,3*S*)-3-Cinnamyl-2-(4-methoxyphenyl)-1-[(2-nitrophenyl)sulfonyl]pyrrolidine

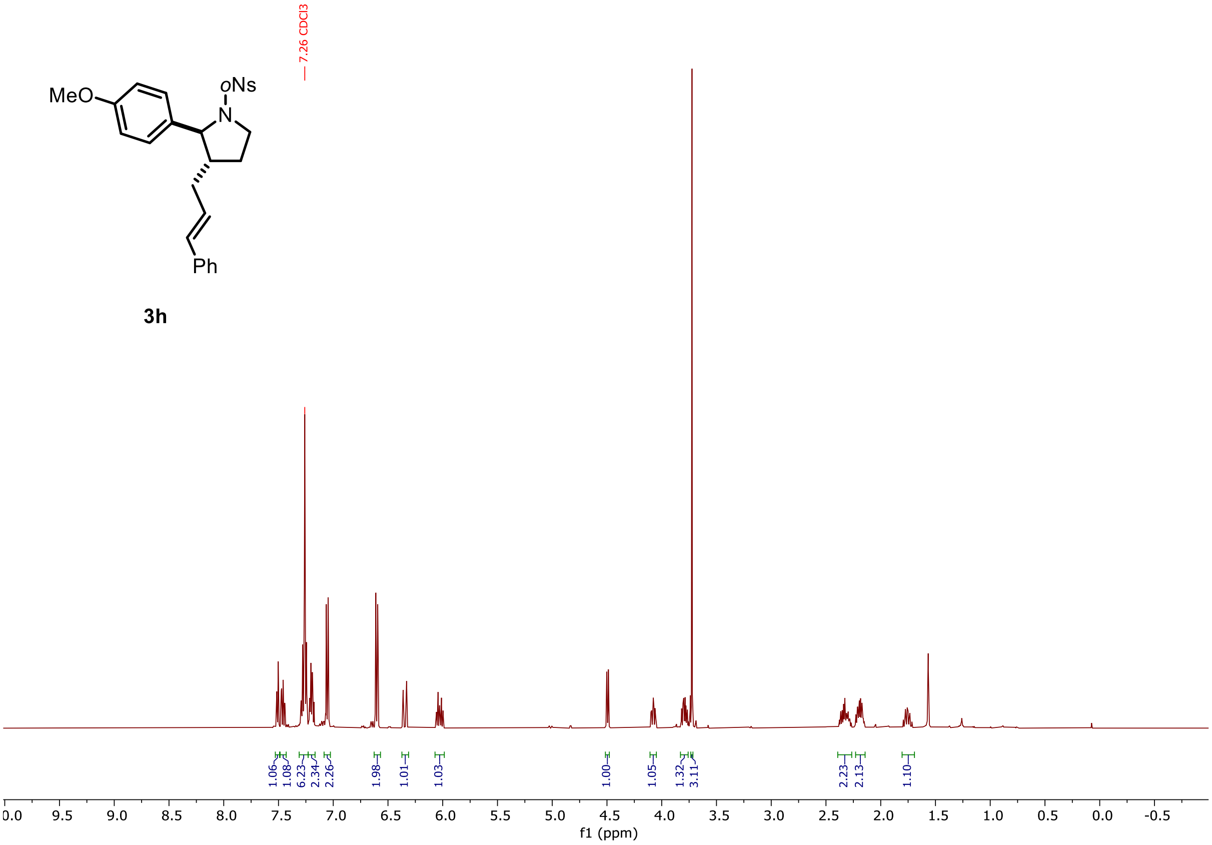

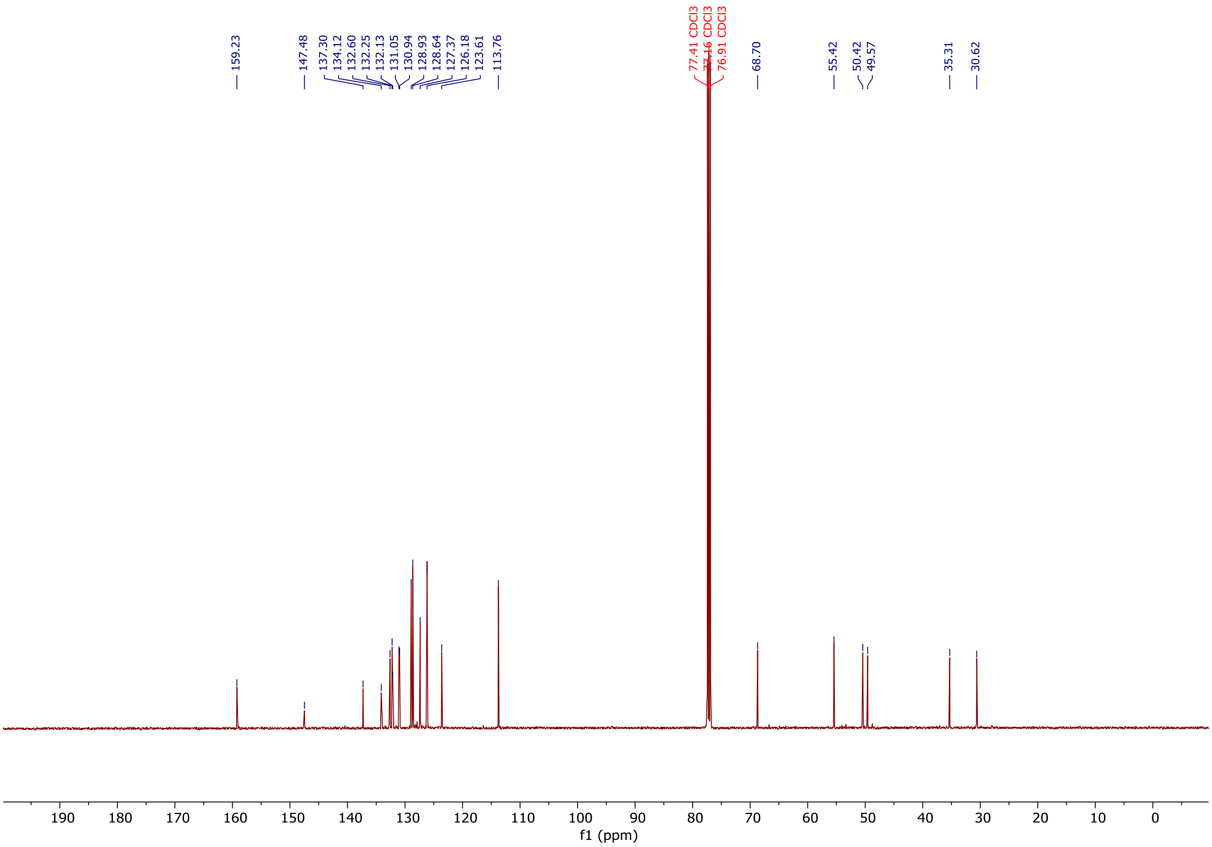

(3i): (±)-4-((*E*)-3-((2*S*,3*S*)-2-(4-Methoxyphenyl)-1-((2-nitrophenyl)sulfonyl)pyrrolidin-3-yl)prop-1-en-1-yl)phenol

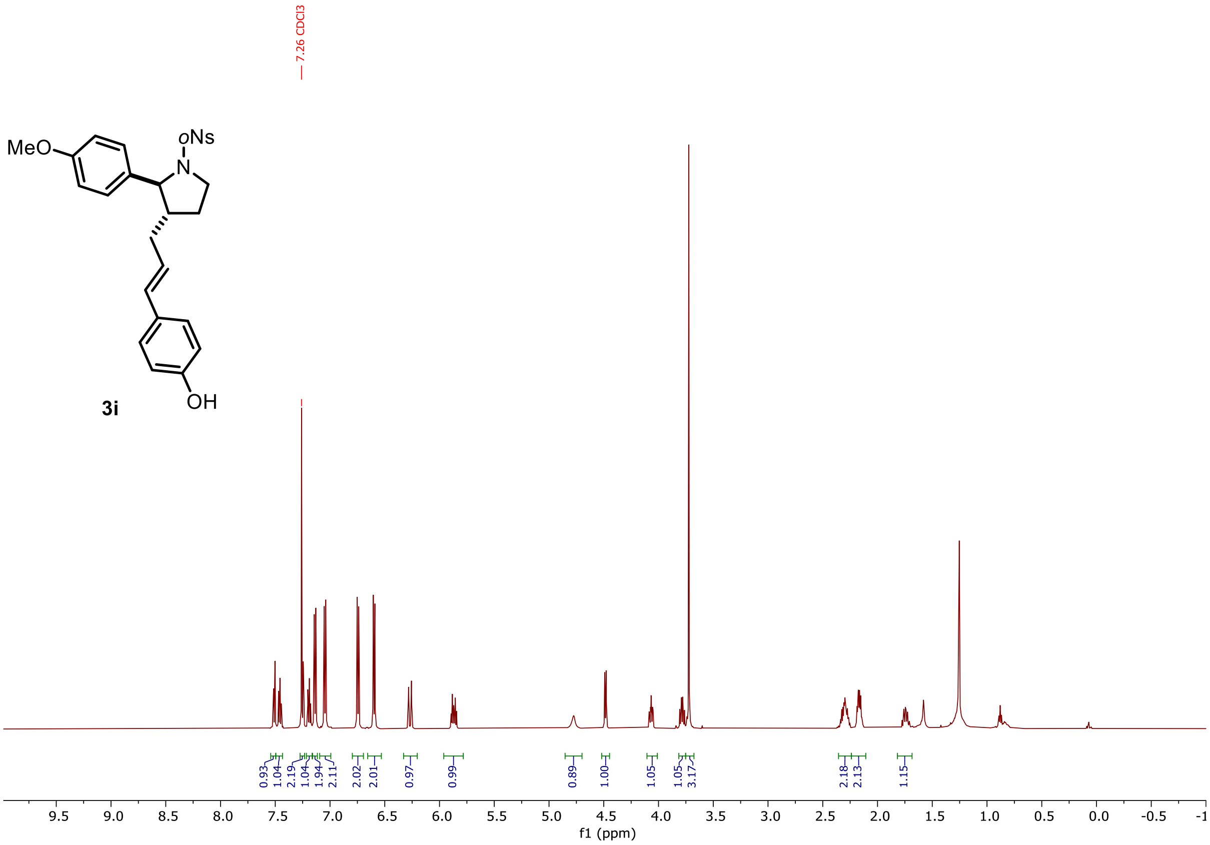

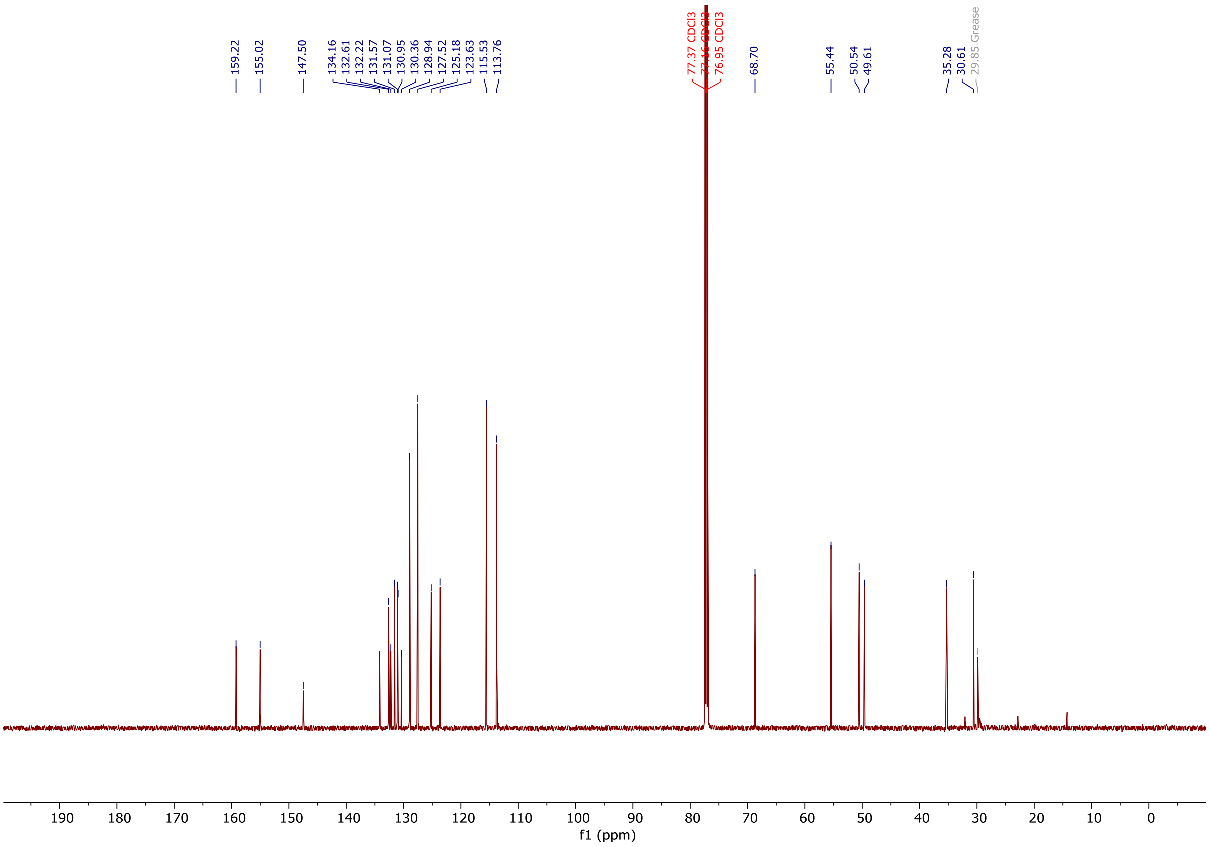

(3j): (±)-(2*S*,3*R*)-2-(4-Methoxyphenyl)-1-[(2-nitrophenyl)sulfonyl]-3-[(1’*S*,2’*E*)-pent-3-en-2-yl]-pyrrolidine

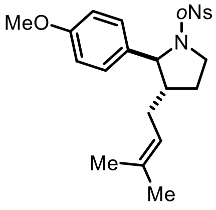

3j

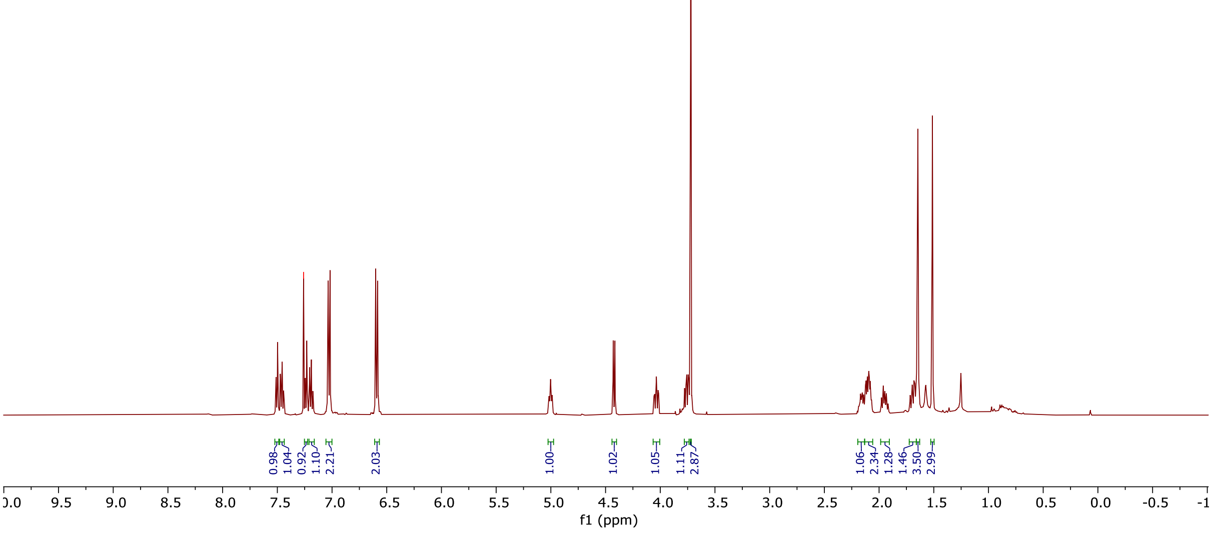

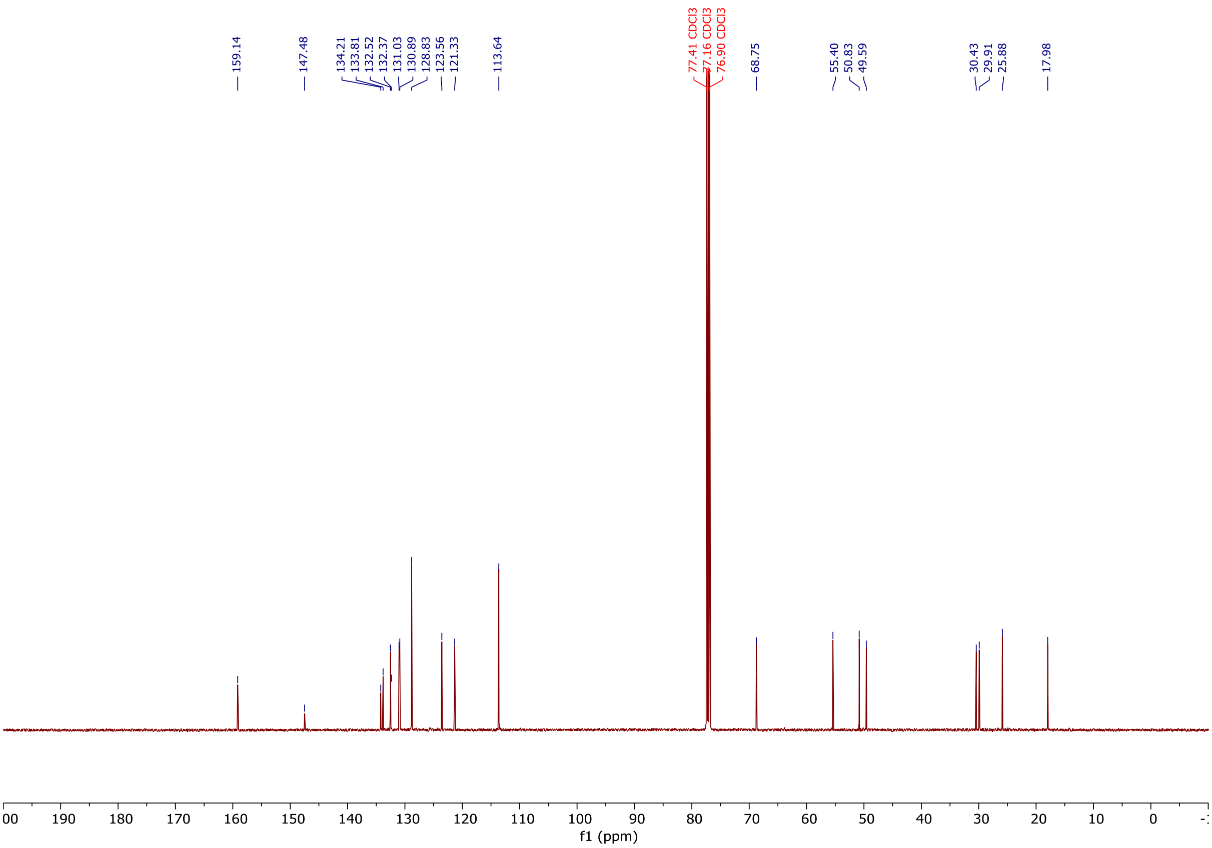

(3k): (±)-(2*S*,3*R*)-2-(4-Methoxyphenyl)-1-[(2-nitrophenyl)sulfonyl]-3-[(1'*S*,2'*E*)-4-phenylbut-3-en-2-yl]pyrrolidine and (±)-(2*S*,3*R*)-2-(4-Methoxyphenyl)-1-[(2-nitrophenyl)sulfonyl]-3-[(1'*R*,2'*E*)-4-phenylbut-3-en-2-yl]pyrrolidine

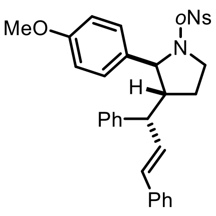

3k

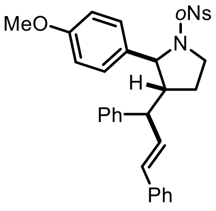

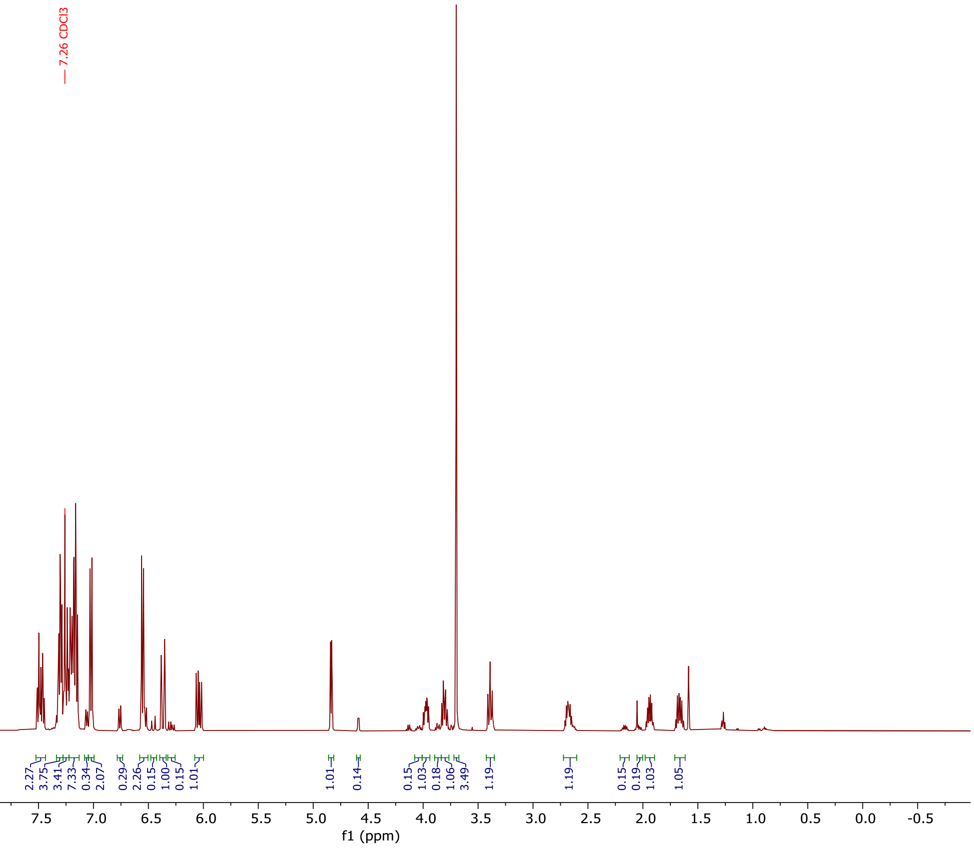

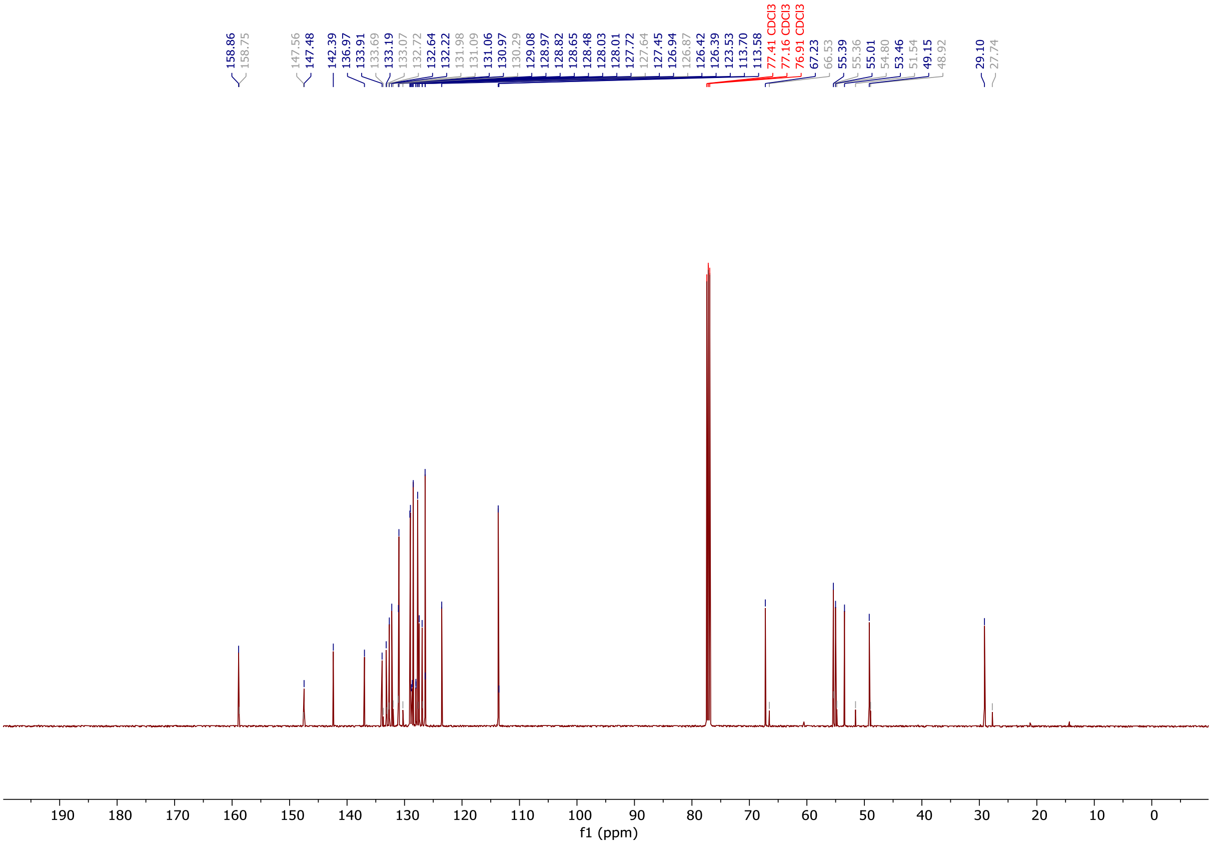

(3l): (±)-(2*S*,3*R*)-2-(4-Methoxyphenyl)-1-[(2-nitrophenyl)sulfonyl]-3-[(1'*S*,2'*E*)-pent-3-en-2-yl]-pyrrolidine and (±)-(2*S*,3*R*)-2-(4-Methoxyphenyl)-1-[(2-nitrophenyl)sulfonyl]-3-[(1'*R*,2'*E*)-pent-3-en-2-yl]pyrrolidine

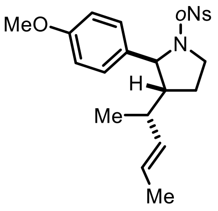

3l

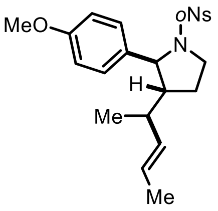

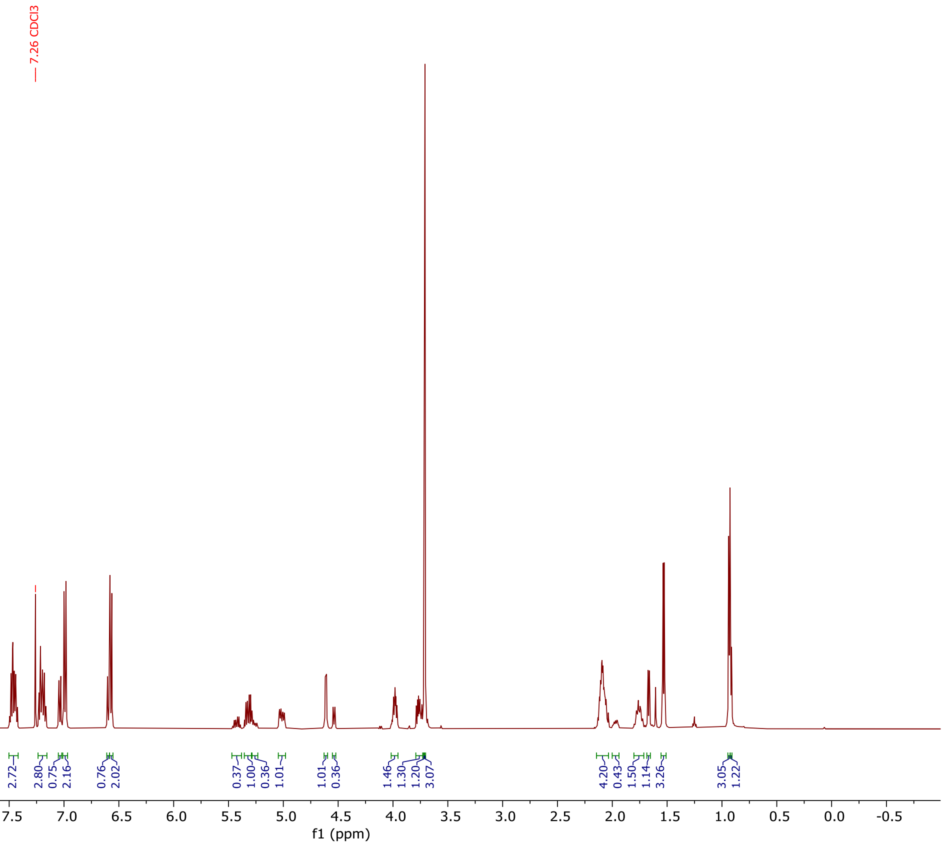

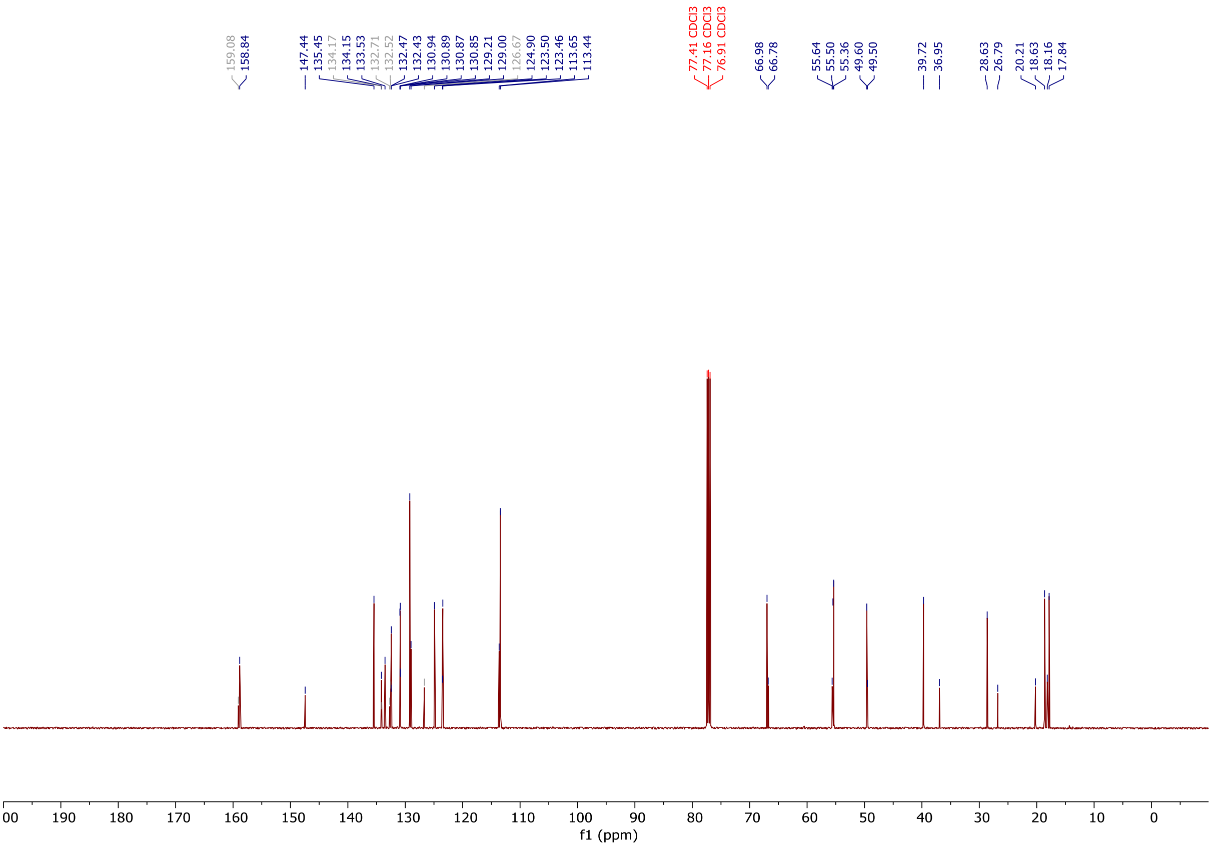

(3m): (±)-(2*S*,3*R*)-2-(4-Methoxyphenyl)-1-[(2-nitrophenyl)sulfonyl]-3-[(1’*S*,2’*E*)-4-phenylbut-3-en-2-yl]pyrrolidine, (±)-(2*S*,3*R*)-2-(4-Methoxyphenyl)-1-[(2-nitrophenyl)sulfonyl]-3-[(1’*R*,2’*E*)-4-phenylbut-3-en-2-yl]pyrrolidine, (±)-(2*S*,3*R*)-2-(4-Methoxyphenyl)-1-[(2-nitrophenyl)sulfonyl]-3-[(1’*S*,2’*E*)-1-phenylbut-2-en-1-yl]pyrrolidine, (±)-(2*S*,3*R*)-2-(4-methoxyphenyl)-1-[(2-nitro-phenyl)sulfonyl]-3-[(1’*R*,2’*E*)-1-phenylbut-2-en-1-yl]pyrrolidine

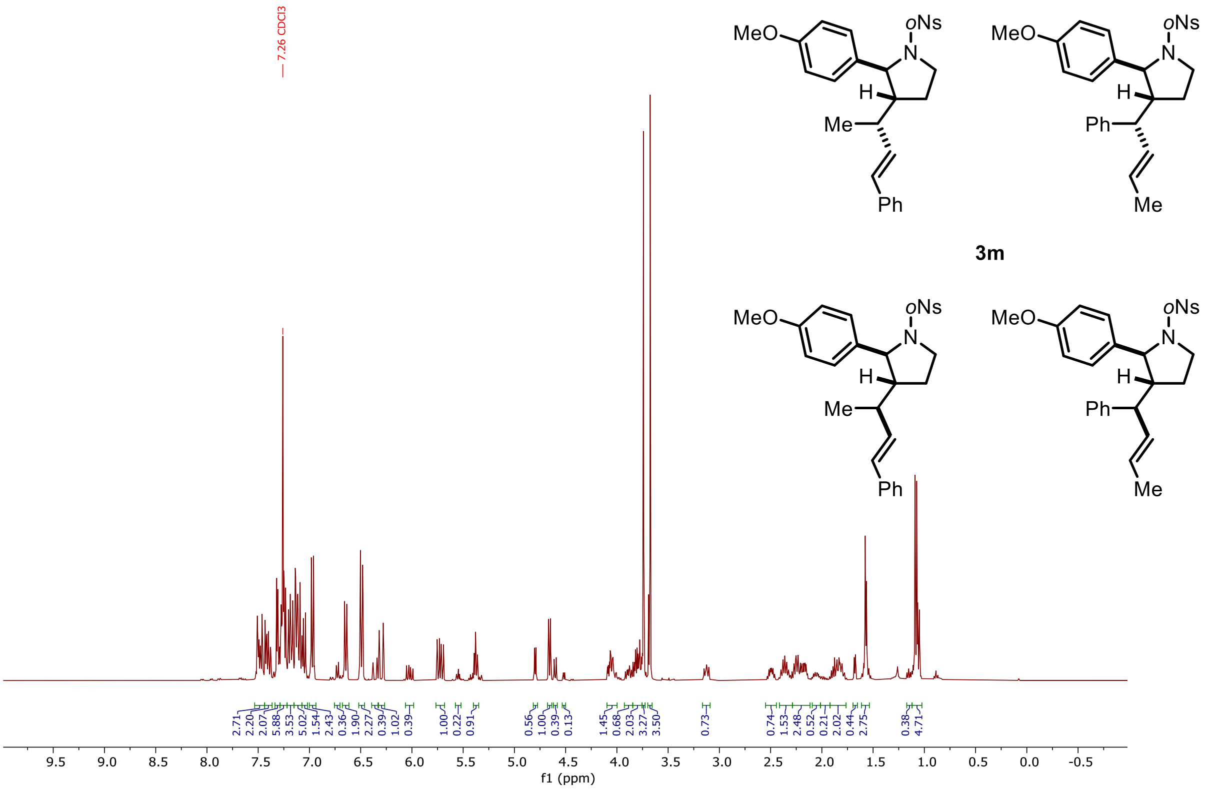

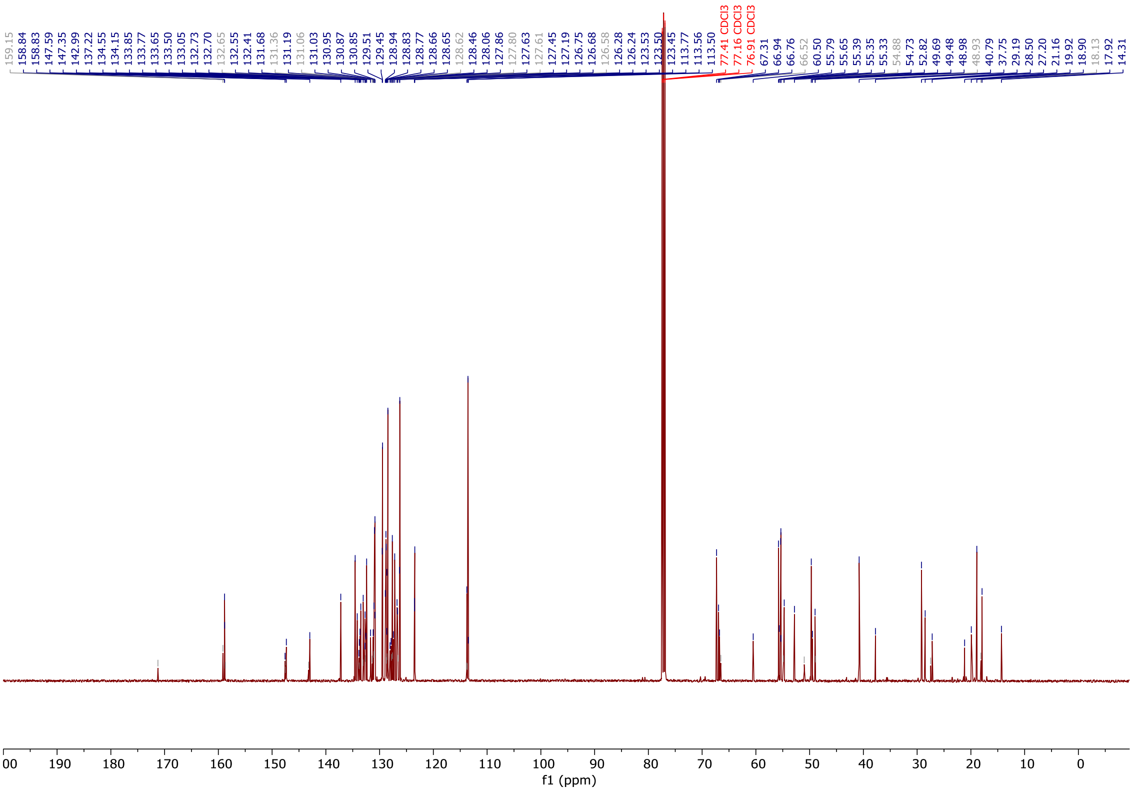

(3n): (±)-(2*S*,3*R*)-3-[(1'*R*)-Cyclohex-2-en-1-yl]-2-(4-methoxyphenyl)-1-[(2-nitrophenyl)sulfonyl]-pyrrolidine and (±)-(2*S*,3*R*)-3-[(1'*S*)-Cyclohex-2-en-1-yl]-2-(4-methoxyphenyl)-1-[(2-nitro-phenyl)sulfonyl]pyrrolidine

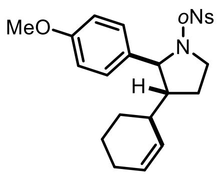

3n

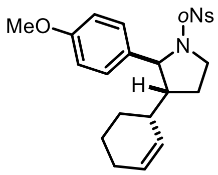

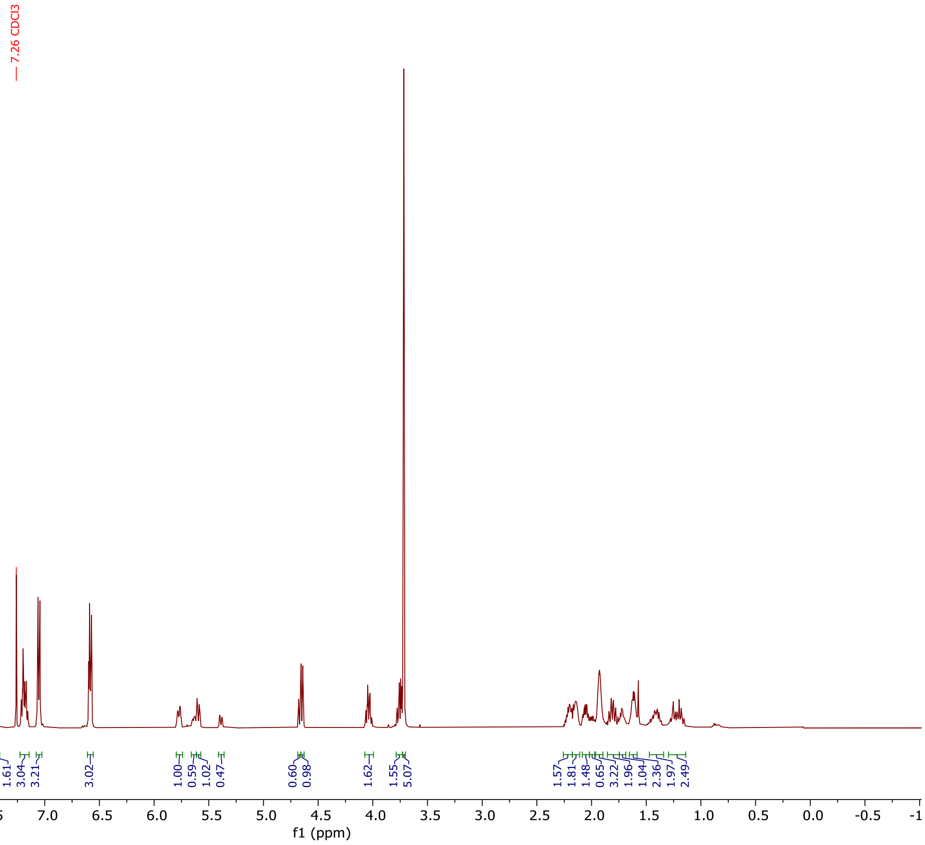

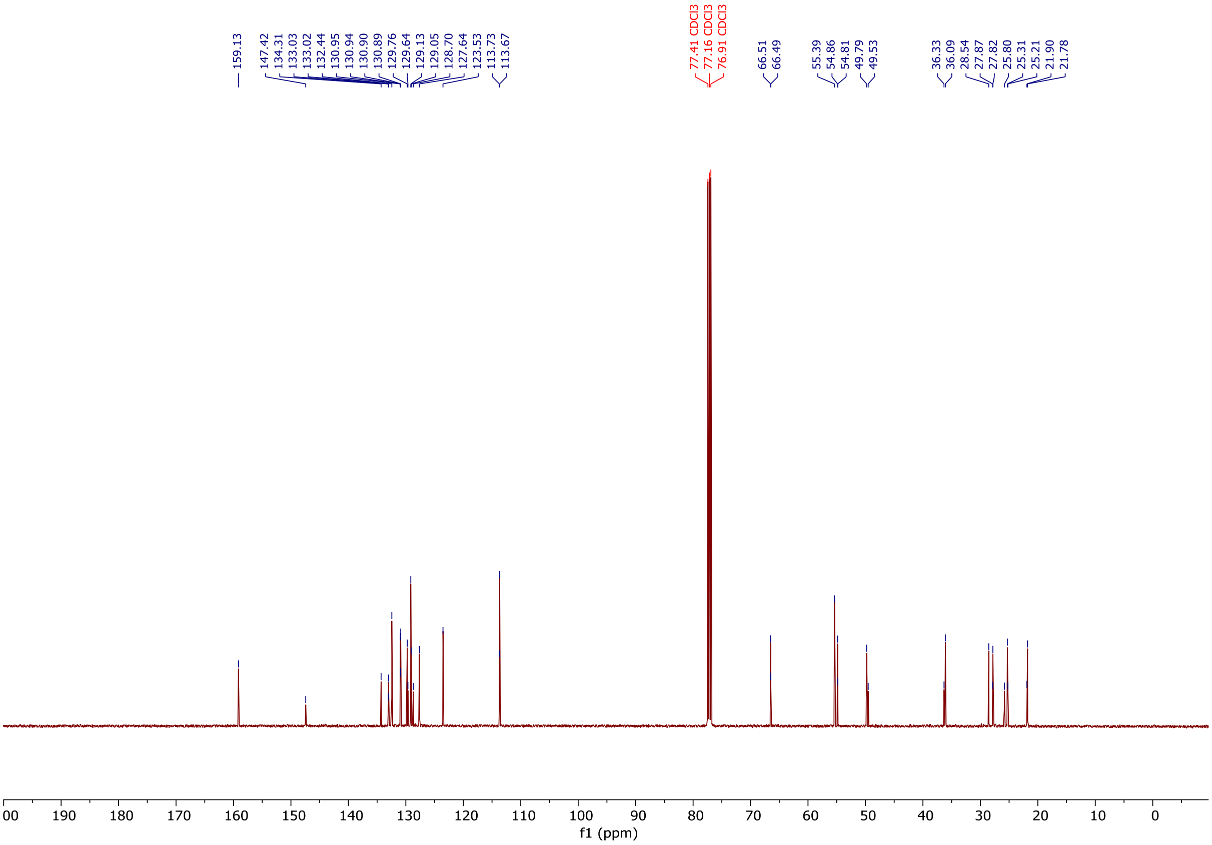

(3o): (±)-(2*S*,3*R*)-3-[(1'*R*)-Cyclopent-2-en-1-yl]-2-(4-methoxyphenyl)-1-[(2-nitrophenyl)sulfonyl]-pyrrolidine and (±)-(2*S*,3*R*)-3-[(1'*S*)-Cyclopent-2-en-1-yl]-2-(4-methoxyphenyl)-1-[(2-nitro-phenyl)sulfonyl]pyrrolidine

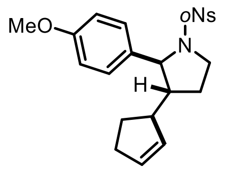

3o

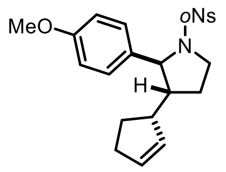

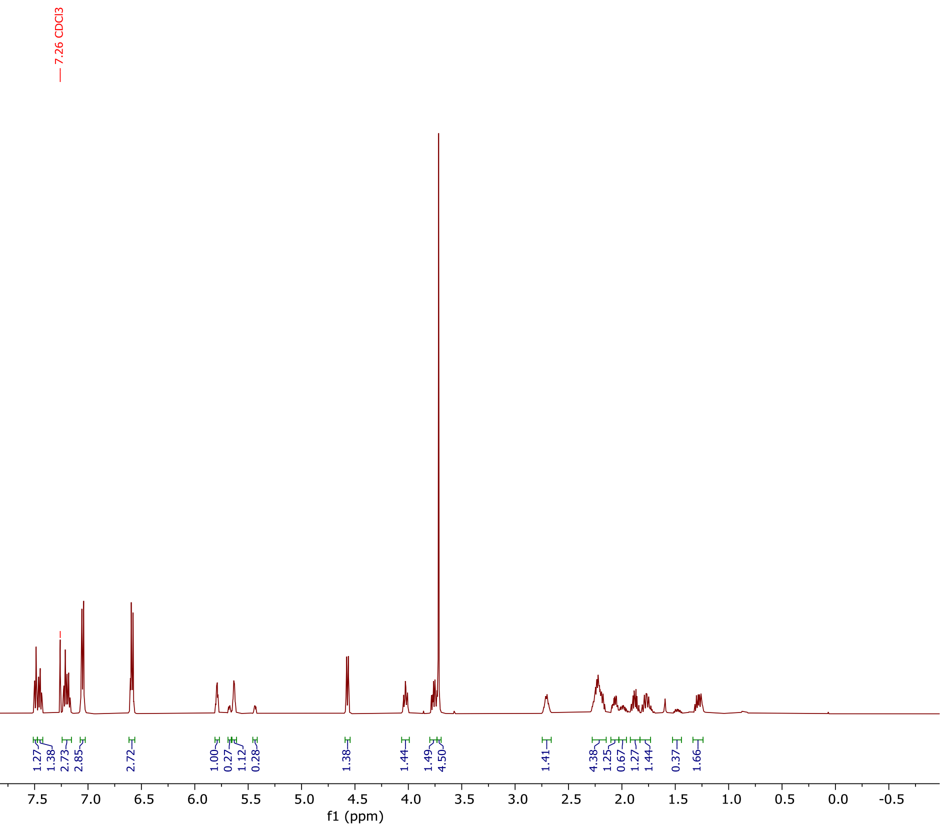

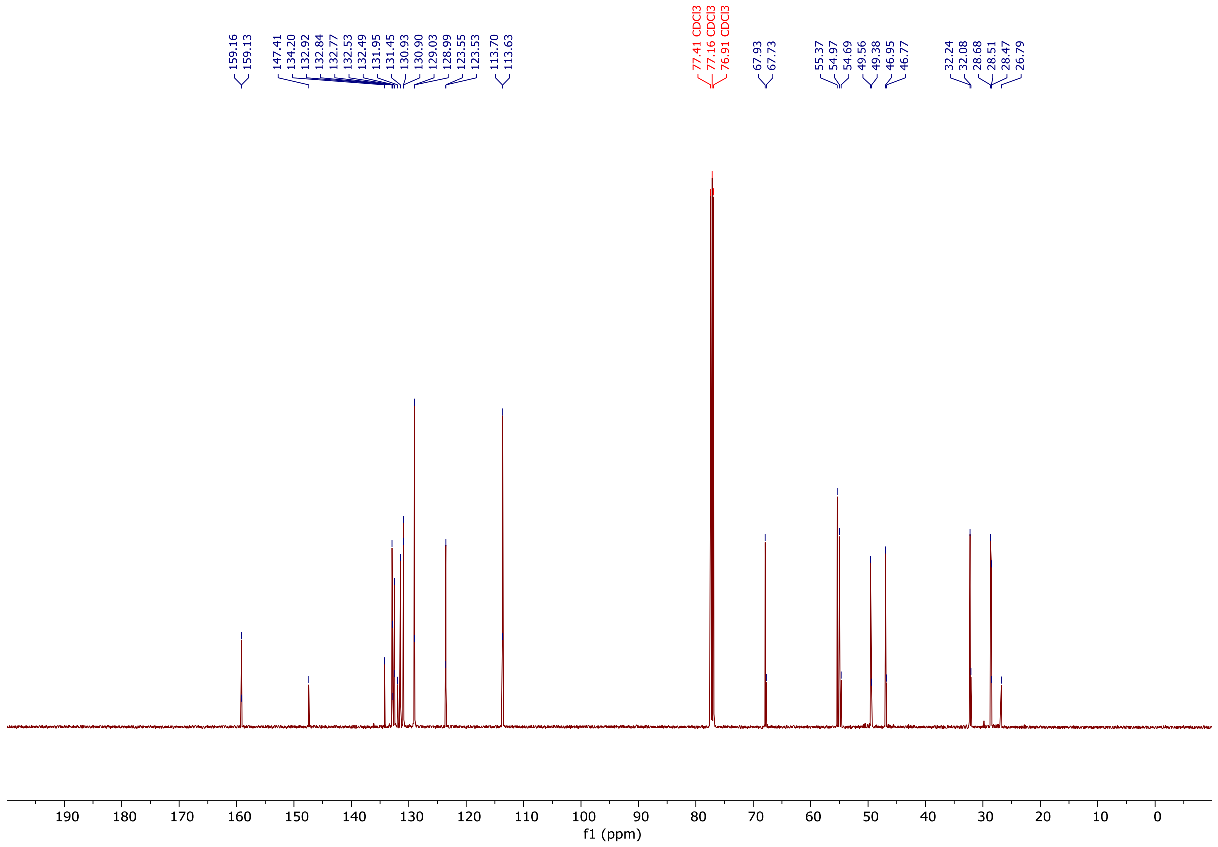

(3p): (±)-(2*S*,3*R*)-2-(4-Methoxyphenyl)-3-[(1'*R*)-3-methylcyclopent-2-en-1-yl]-1-[(2-nitrophenyl)-sulfonyl]pyrrolidine and (±)-(2*S*,3*R*)-2-(4-Methoxyphenyl)-3-[(1'*S*)-3-methylcyclopent-2-en-1-yl]-1-[(2-nitrophenyl)sulfonyl]pyrrolidine

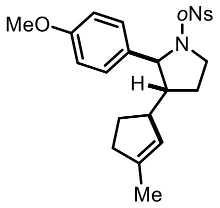

3p

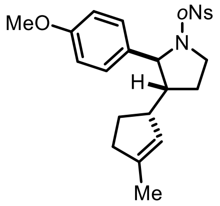

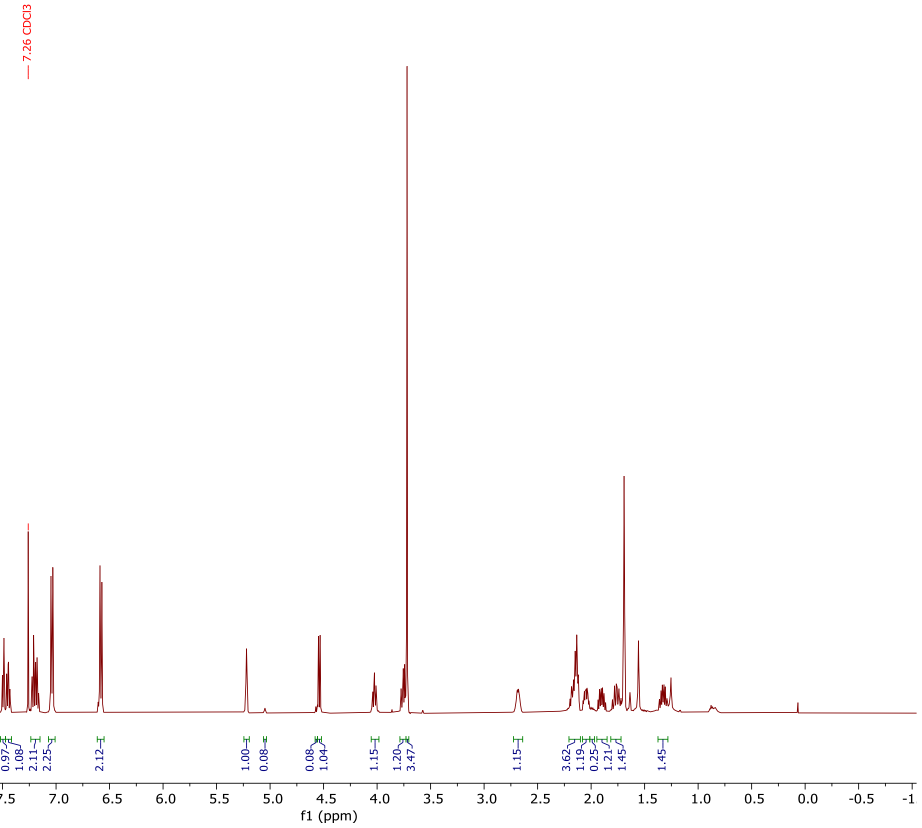

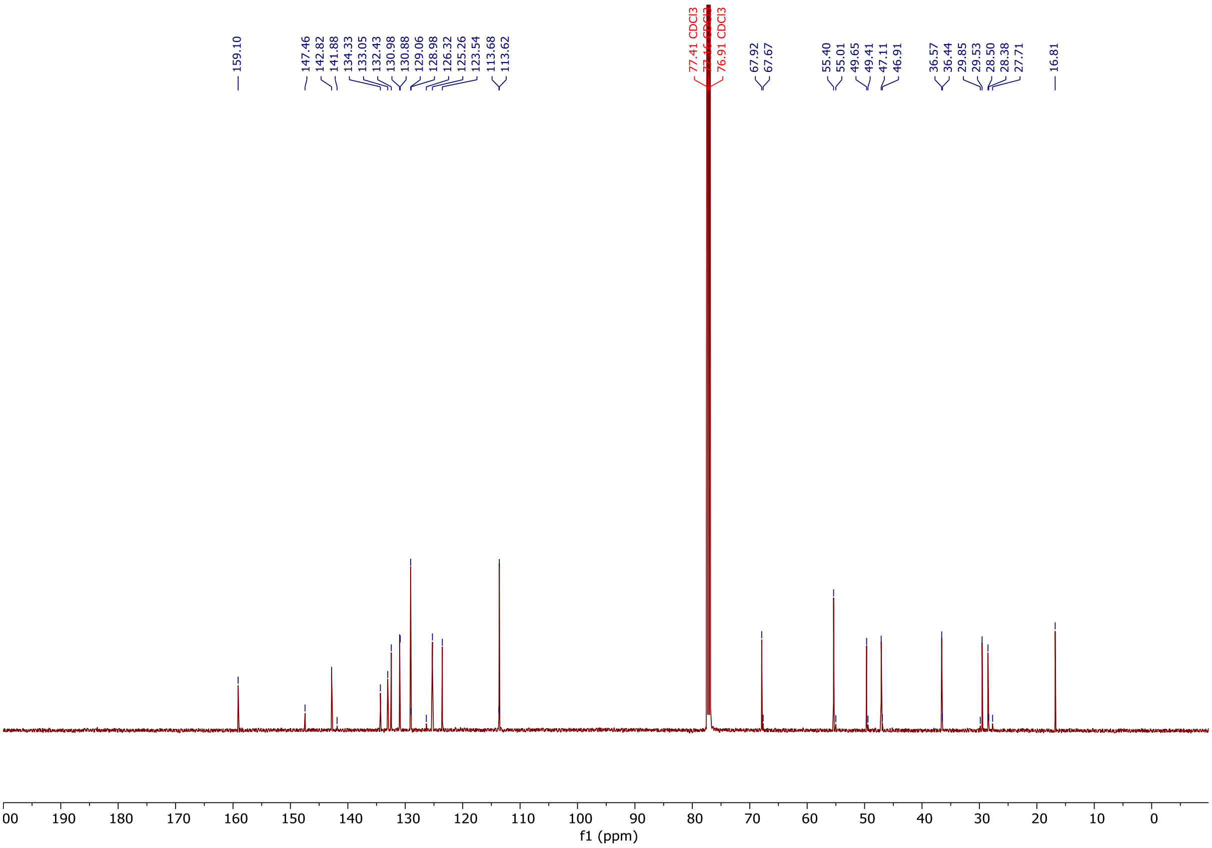

(3q): (±)-(2*S*,3*R*)-2-(4-Methoxyphenyl)-1-[(2-nitrophenyl)sulfonyl)-3-[(1’*R*)-3-phenylcyclopent-2-en-1-yl]pyrrolidine

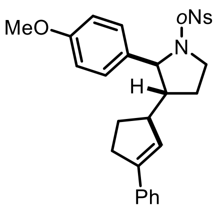

3q

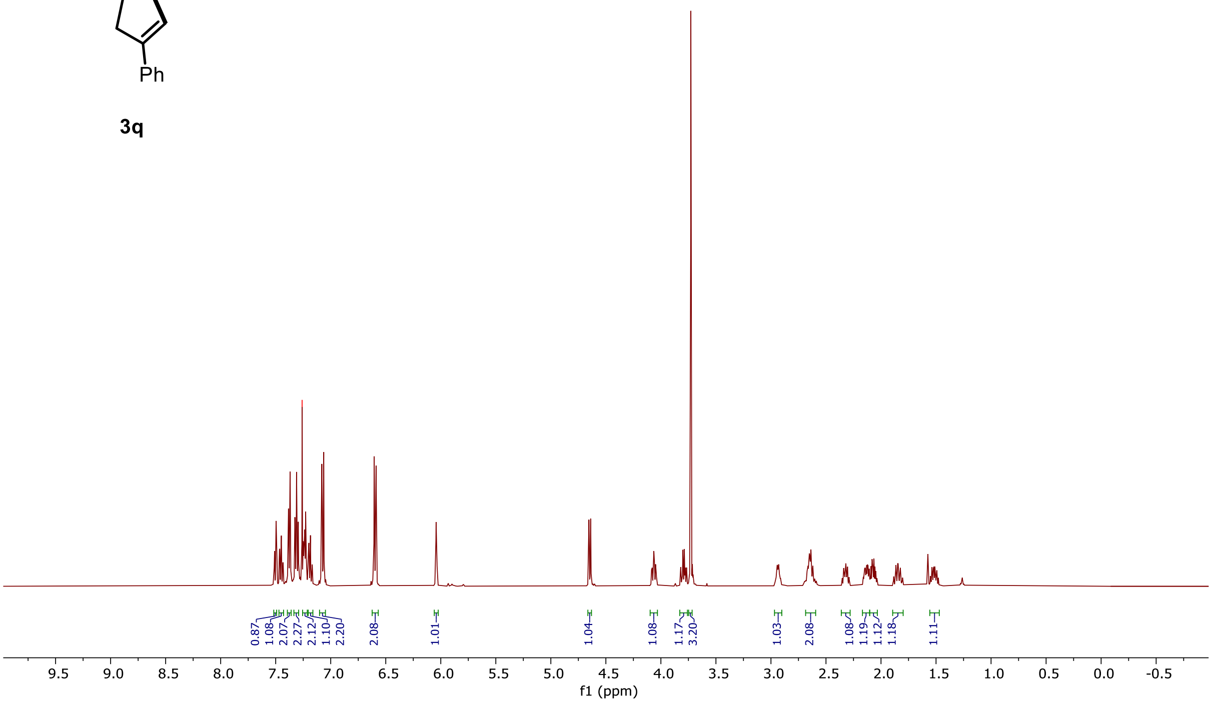

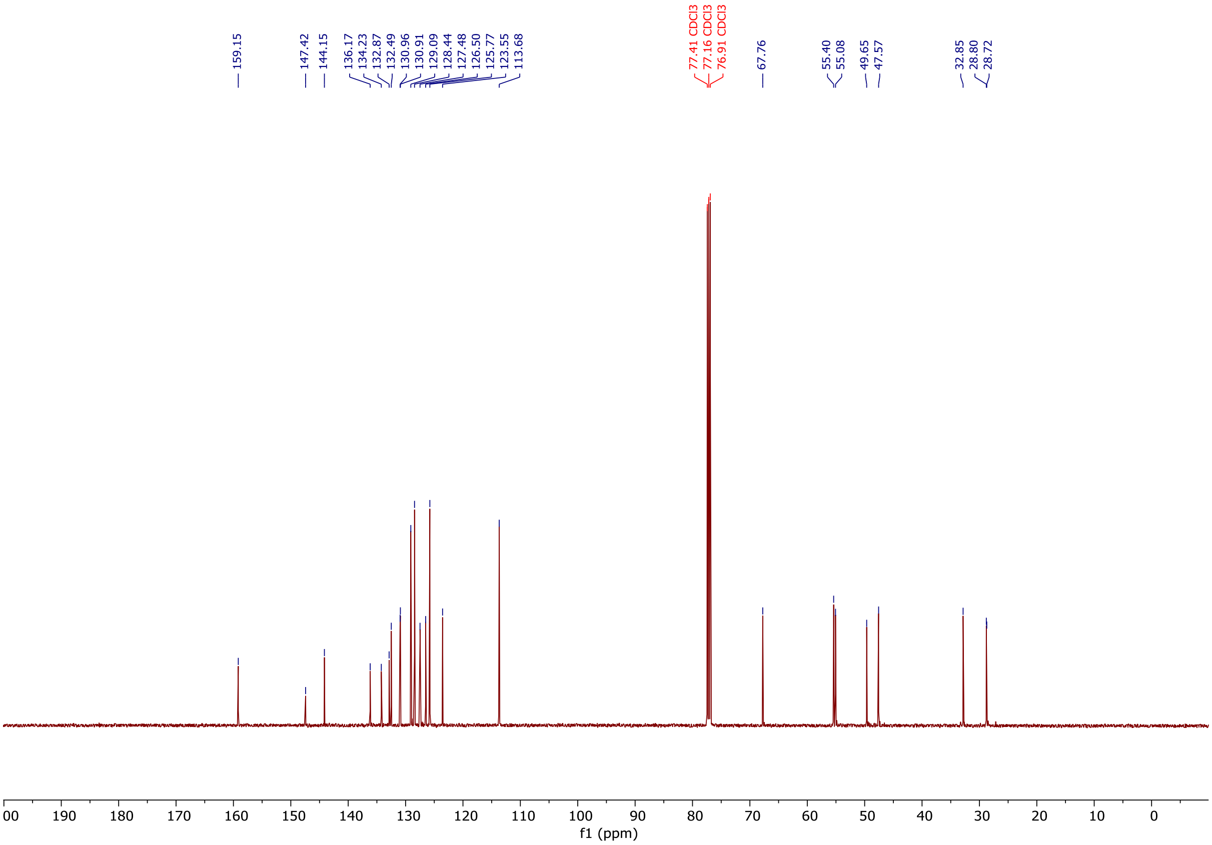

(3r): (±)-{(2*S*,3*S*)-2-(4-Methoxyphenyl)-1-[(2-nitrophenyl)sulfonyl]pyrrolidin-3-yl}methanol

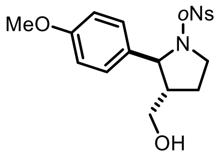

3r

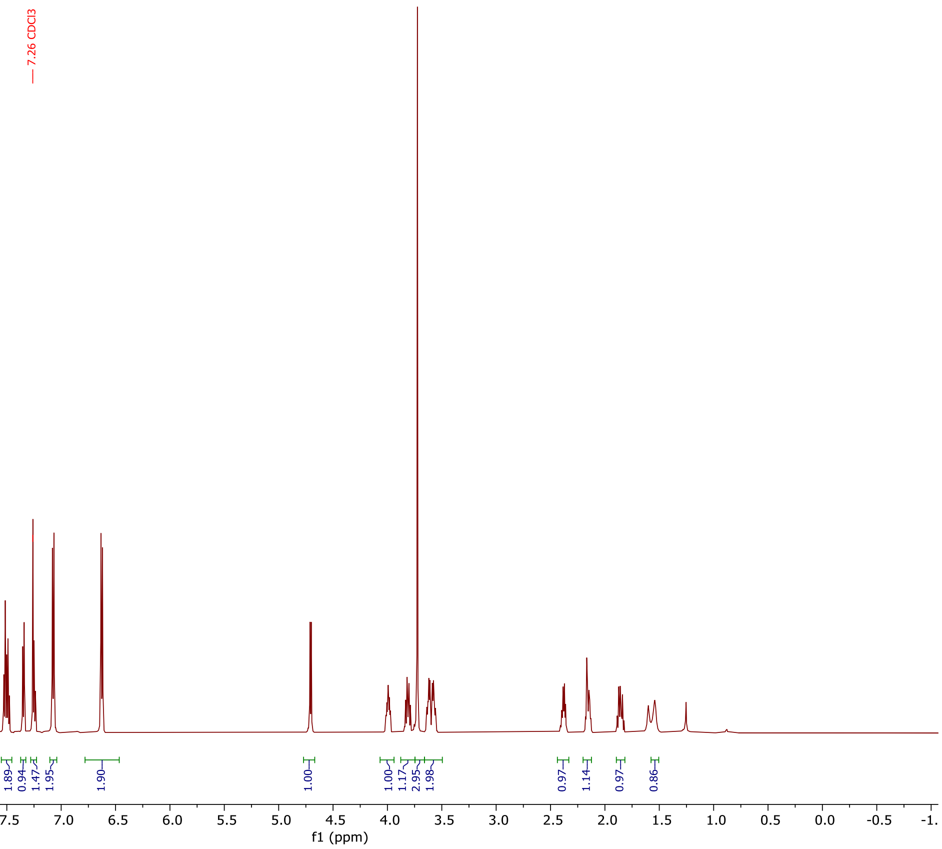

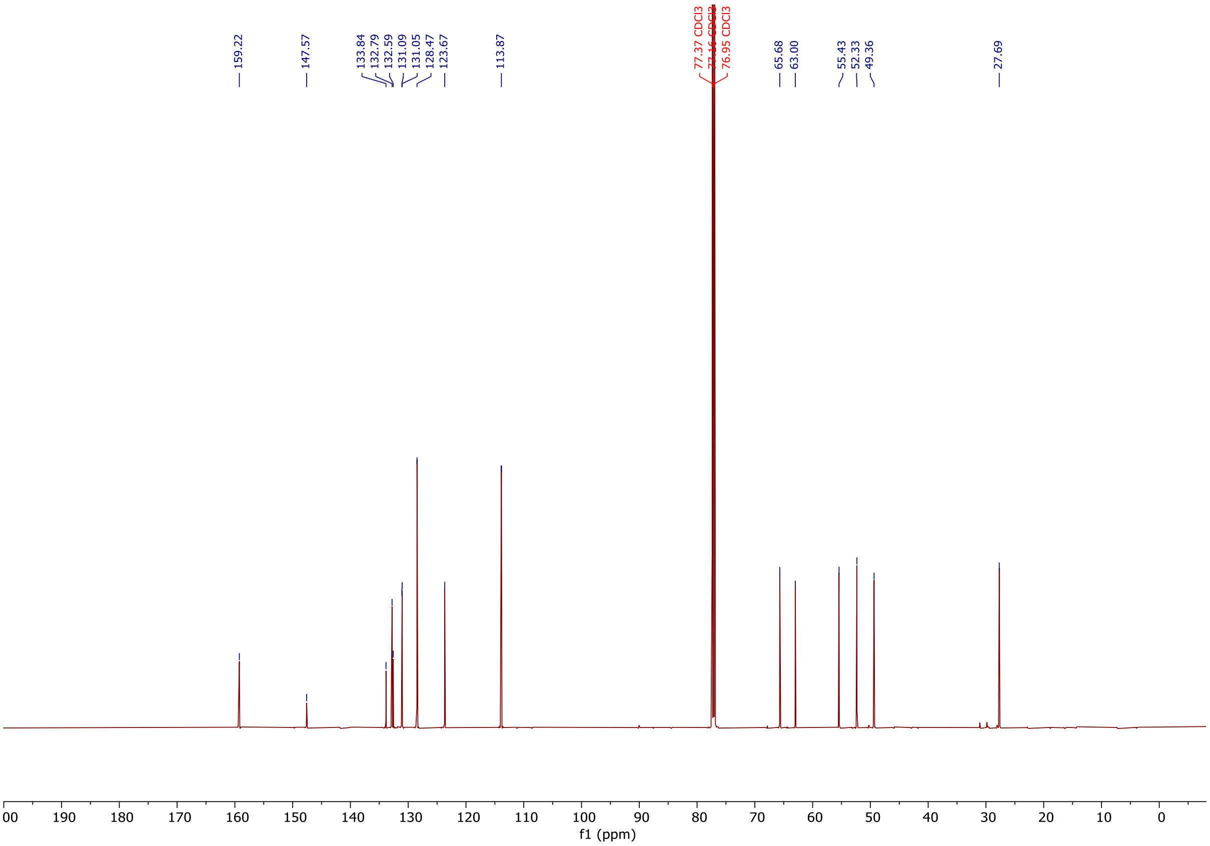

(3s): (±)-(2*S*,3*R*)-3-Benzhydryl-1-[(2-nitrophenyl)sulfonyl]-2-phenylpyrrolidine

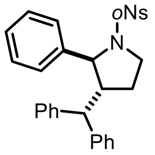

3s

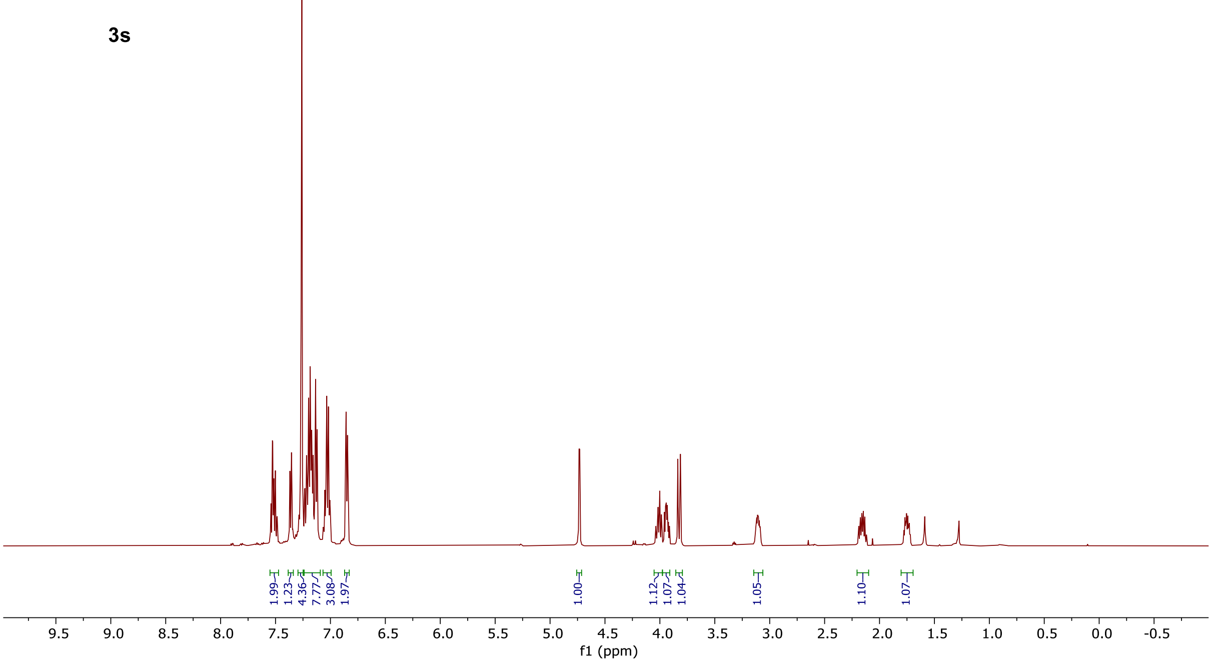

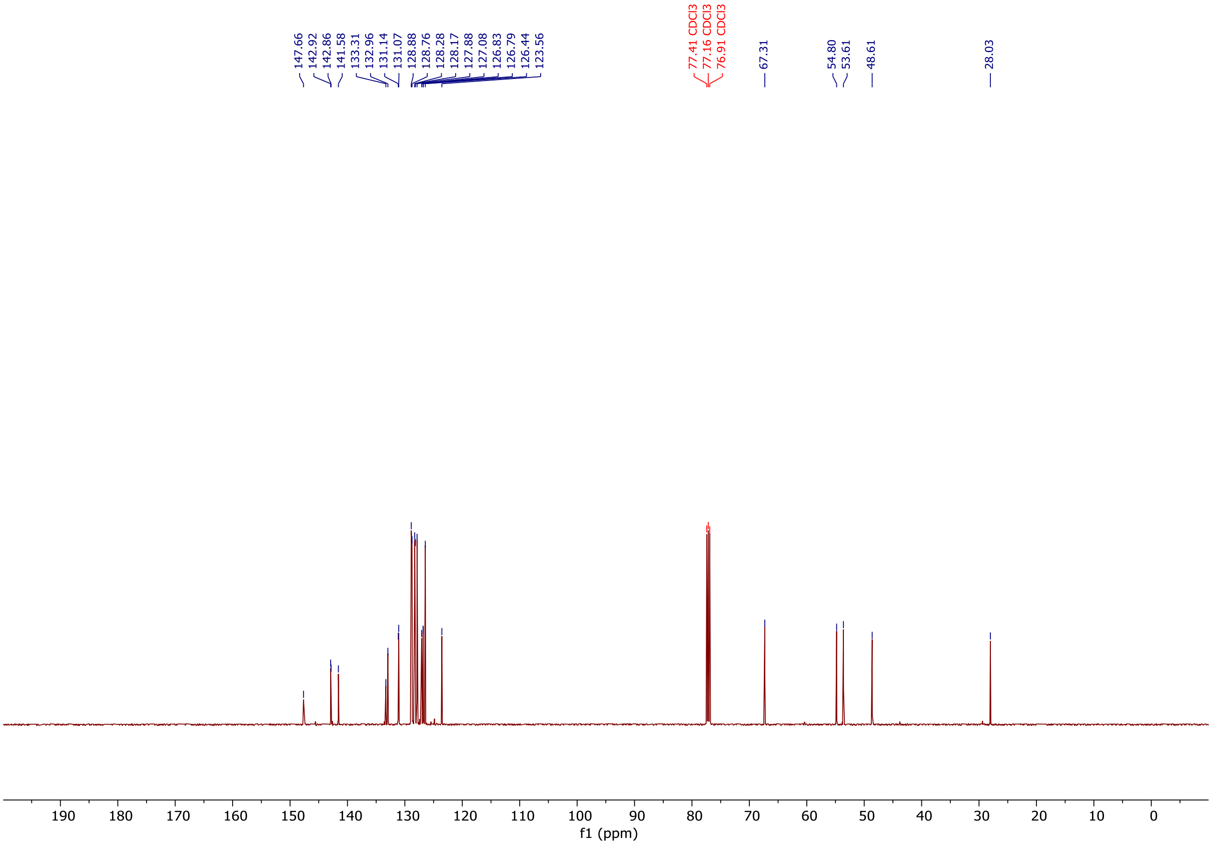

(3t): (±)-(2*S*,3*S*)-3-(4-Methoxybenzyl)-1-[(2-nitrophenyl)sulfonyl]-2-phenylpyrrolidine

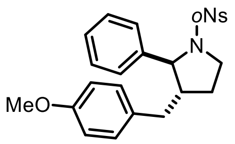

3t

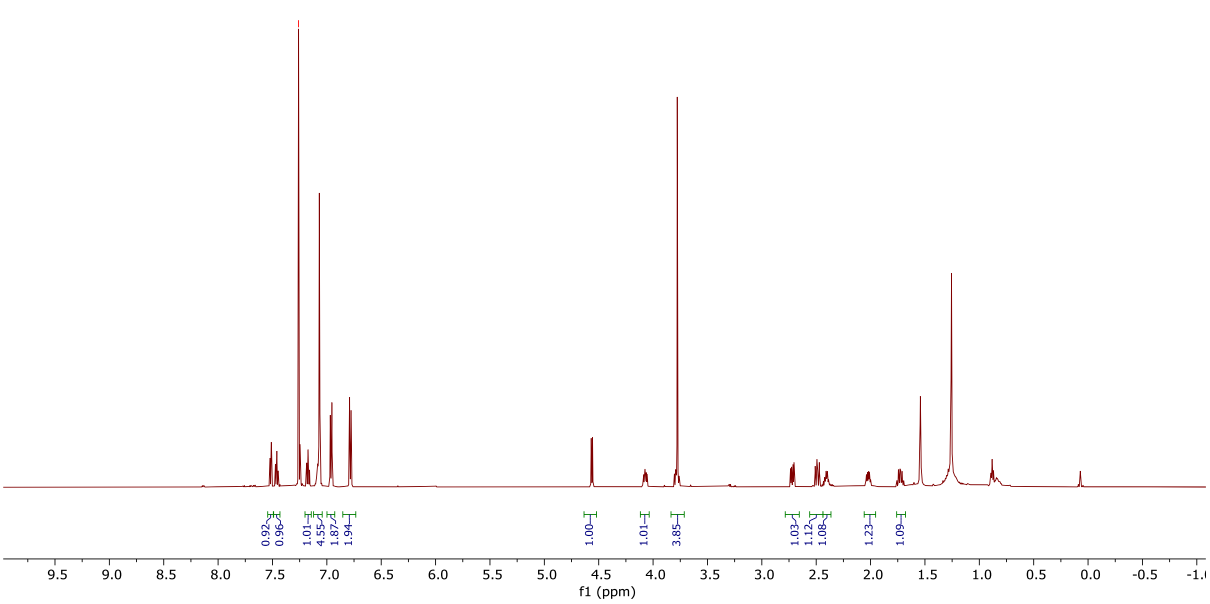

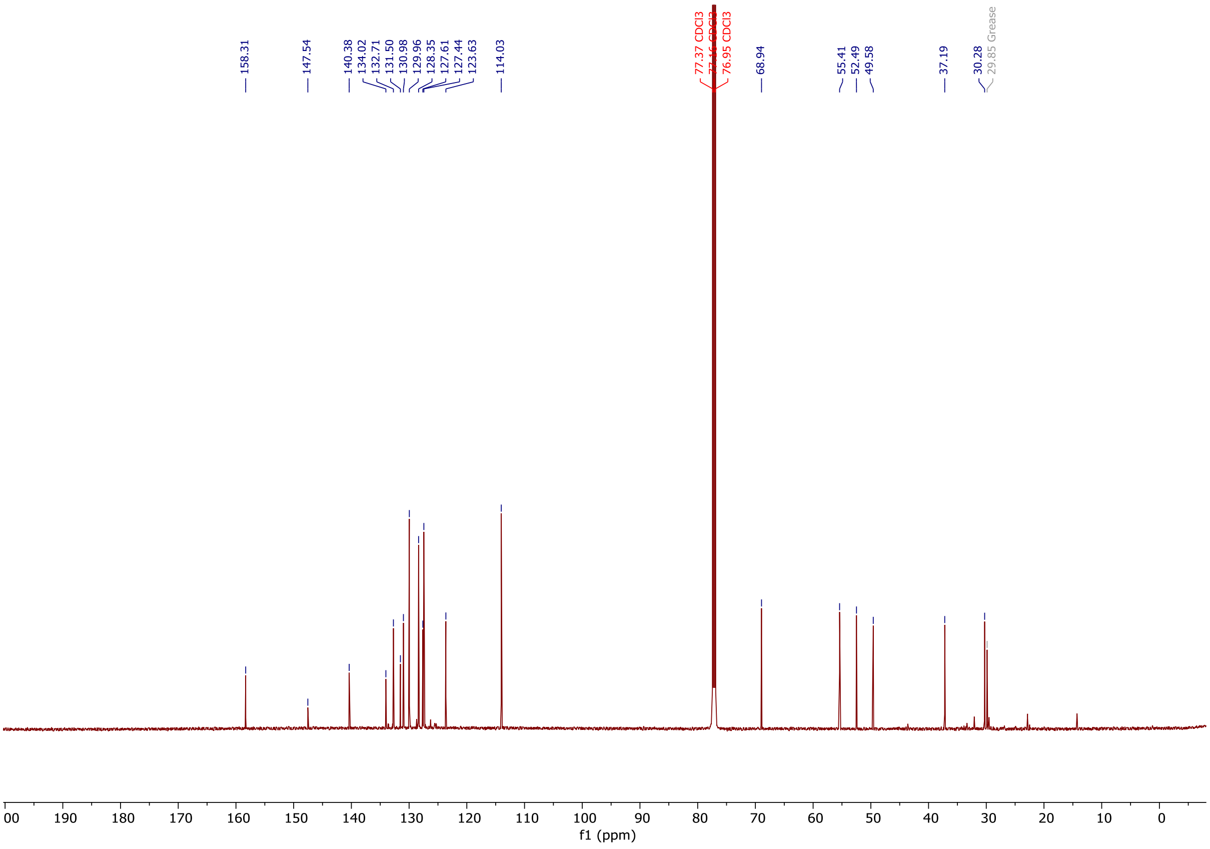

(3u): (±)-(2*S*,3*R*)-3-Benzhydryl-2-(4-bromophenyl)-1-[(2-nitrophenyl)sulfonyl]pyrrolidine

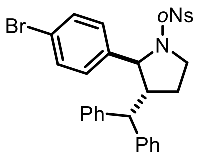

3u

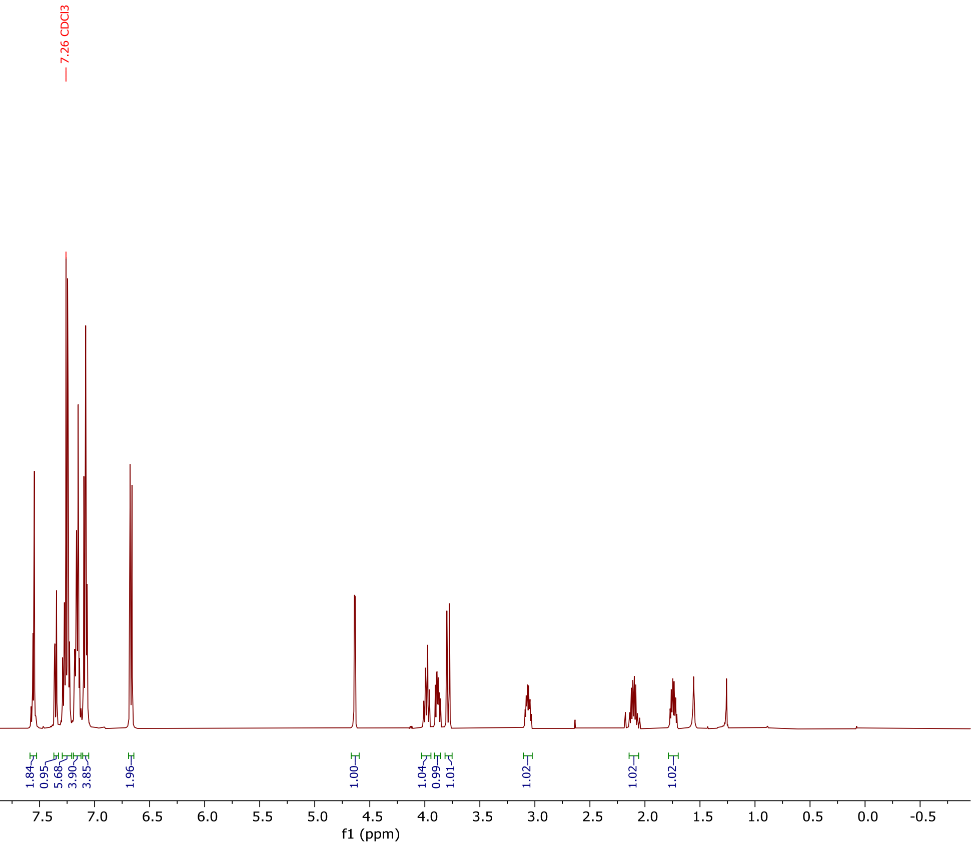

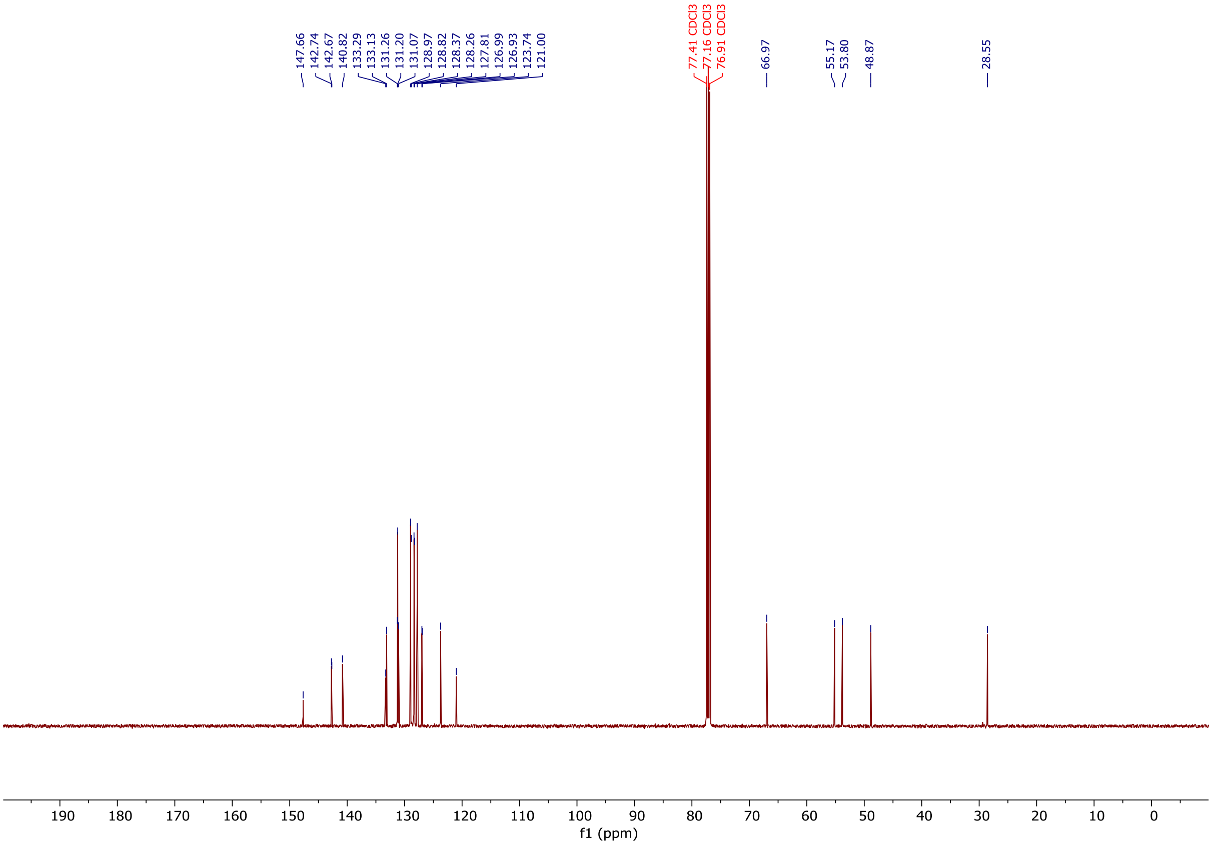

(3v): (±)-3-Benzhydryl-2,2-dimethyl-1-[(2-nitrophenyl)sulfonyl]pyrrolidine

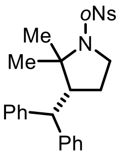

3v

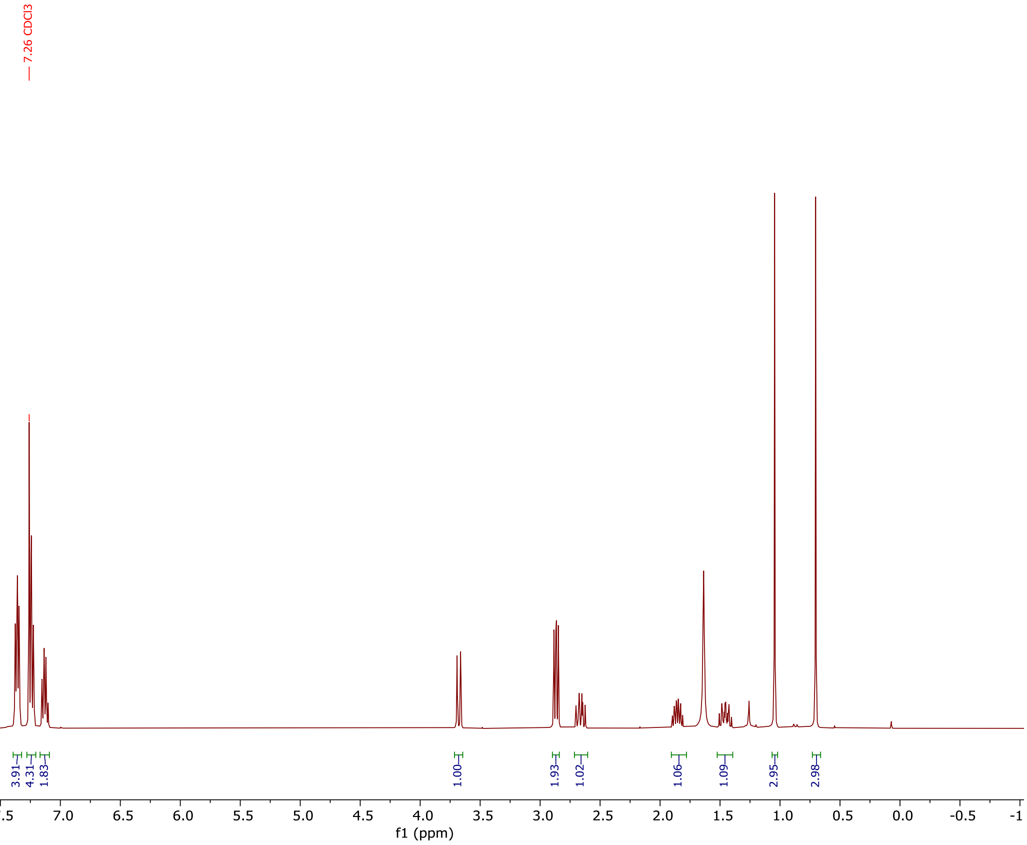

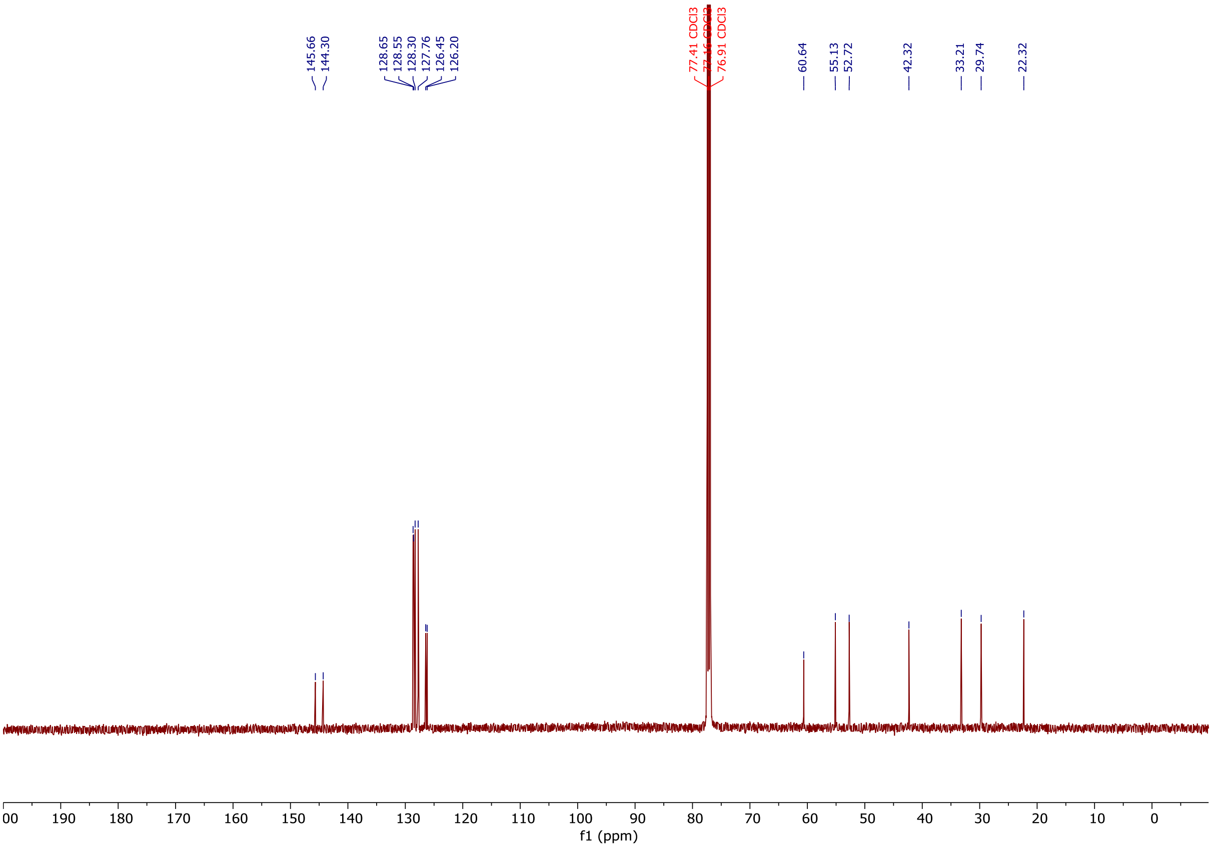

(3w): (±)- 4-Benzhydryl-1-[(2-nitrophenyl)sulfonyl]-1-azaspiro[4.4]nonane

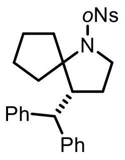

3w

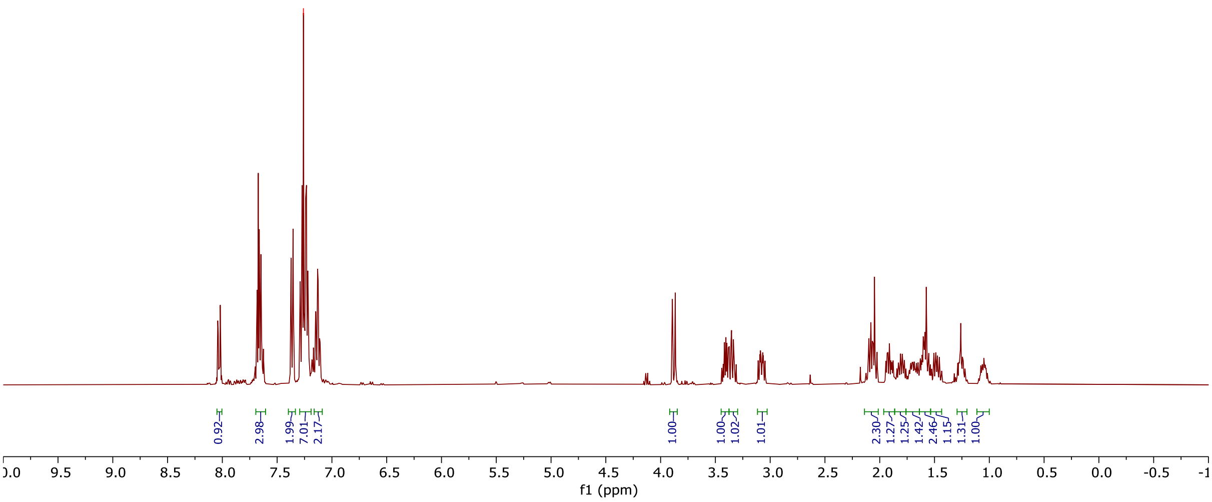

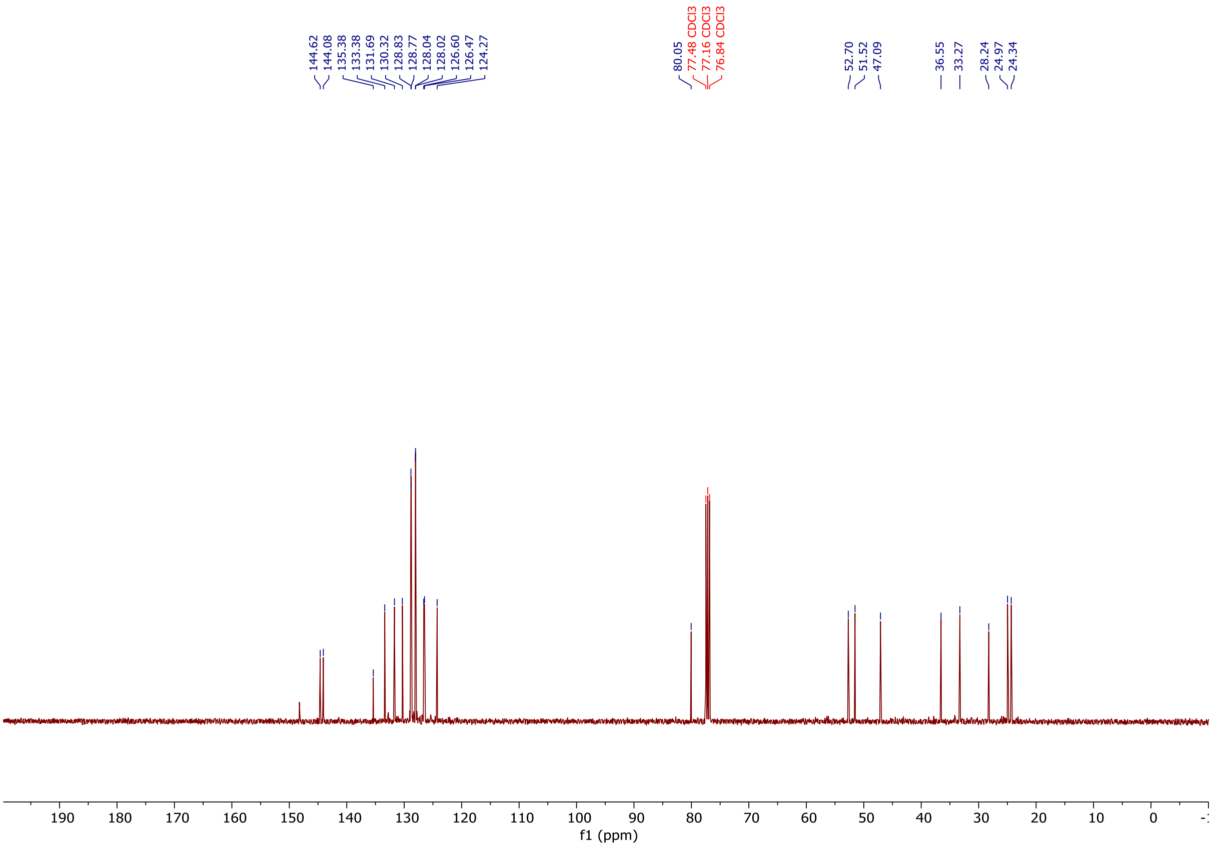

(3x): (±)-(2*R*,3*R*)-3-Benzhydryl-2-[(1'*E*)-4-methoxystyryl]-1-[(2-nitrophenyl)sulfonyl]-pyrrolidine and (±)-(2*R*,3*S*)-3-Benzhydryl-2-[(1'*E*)-4-methoxystyryl]-1-[(2-nitrophenyl)sulfonyl]-pyrrolidine

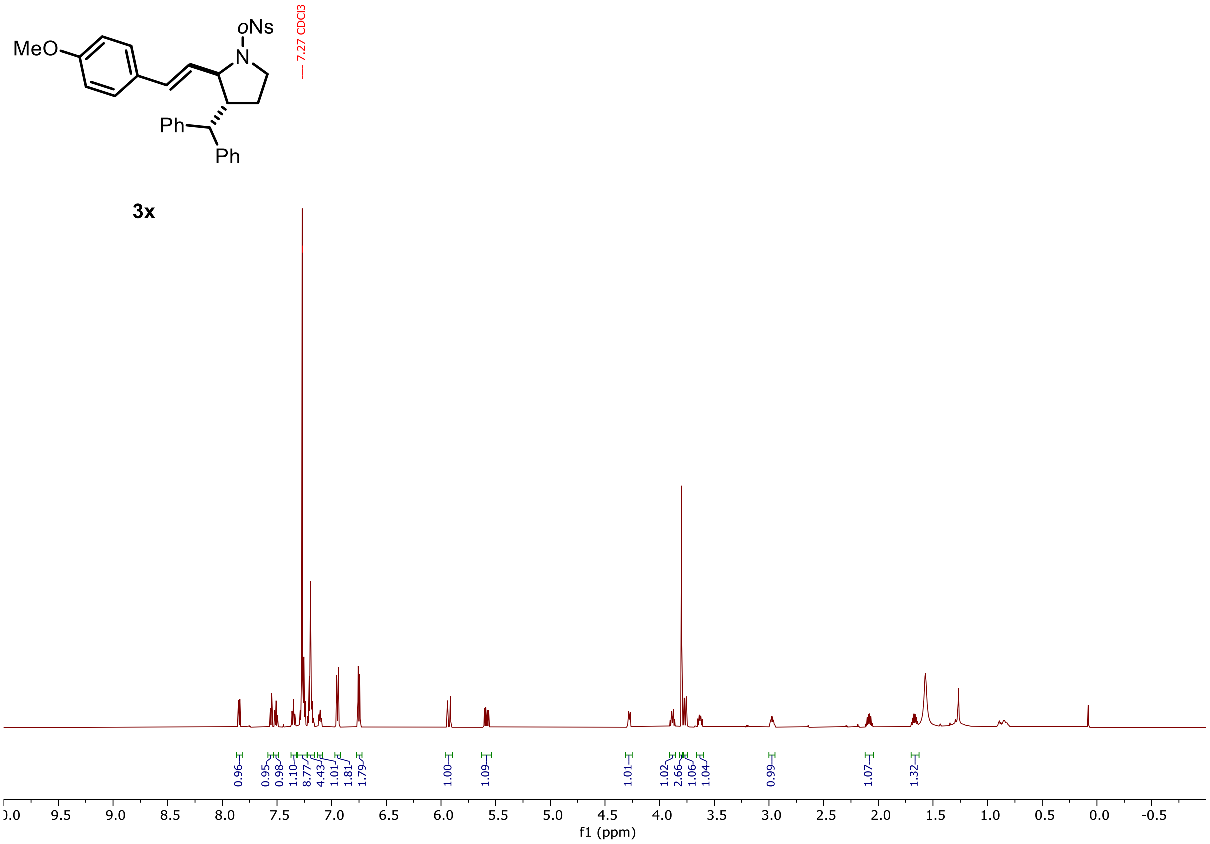

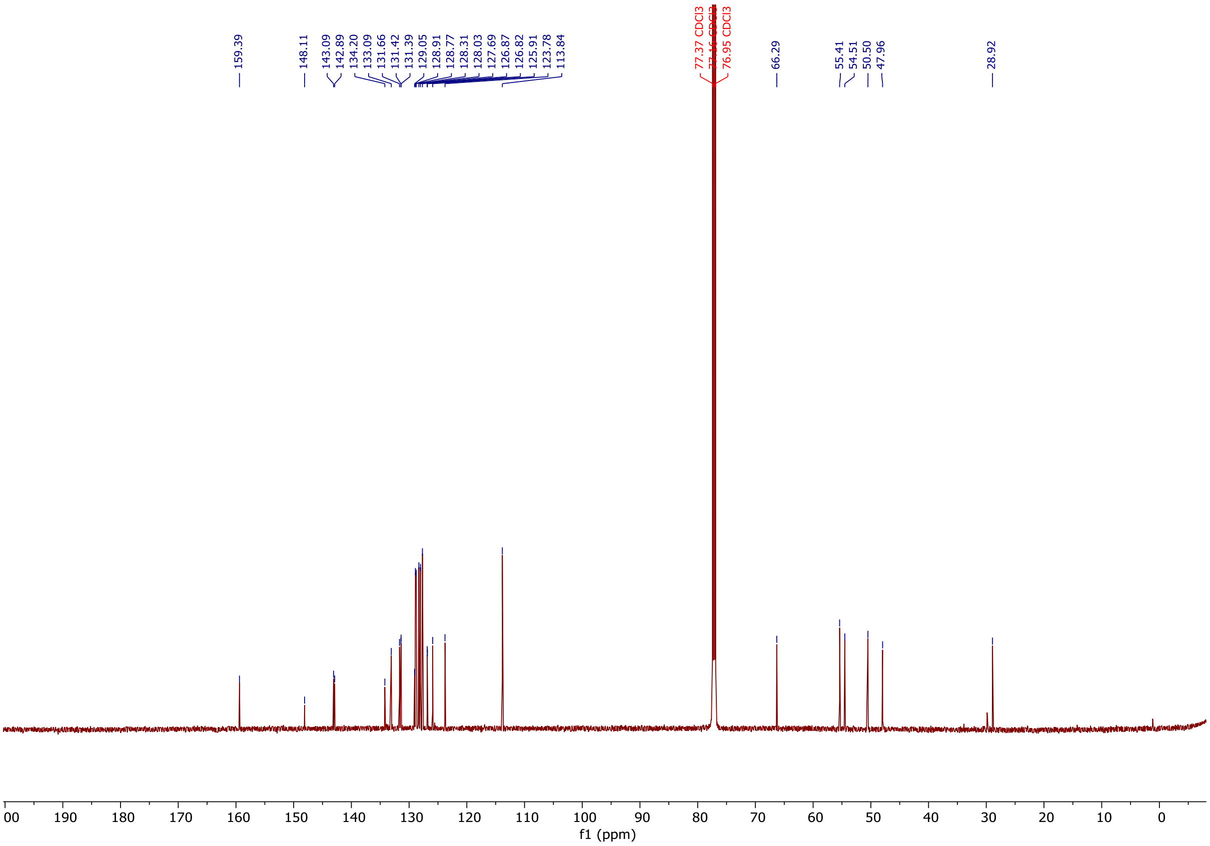

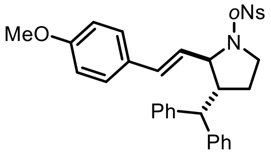
MeO
oNs
N
Ph
Ph

3x

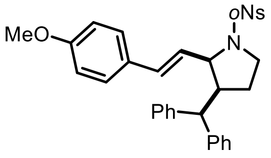
MeO
oNs
N
Ph
Ph

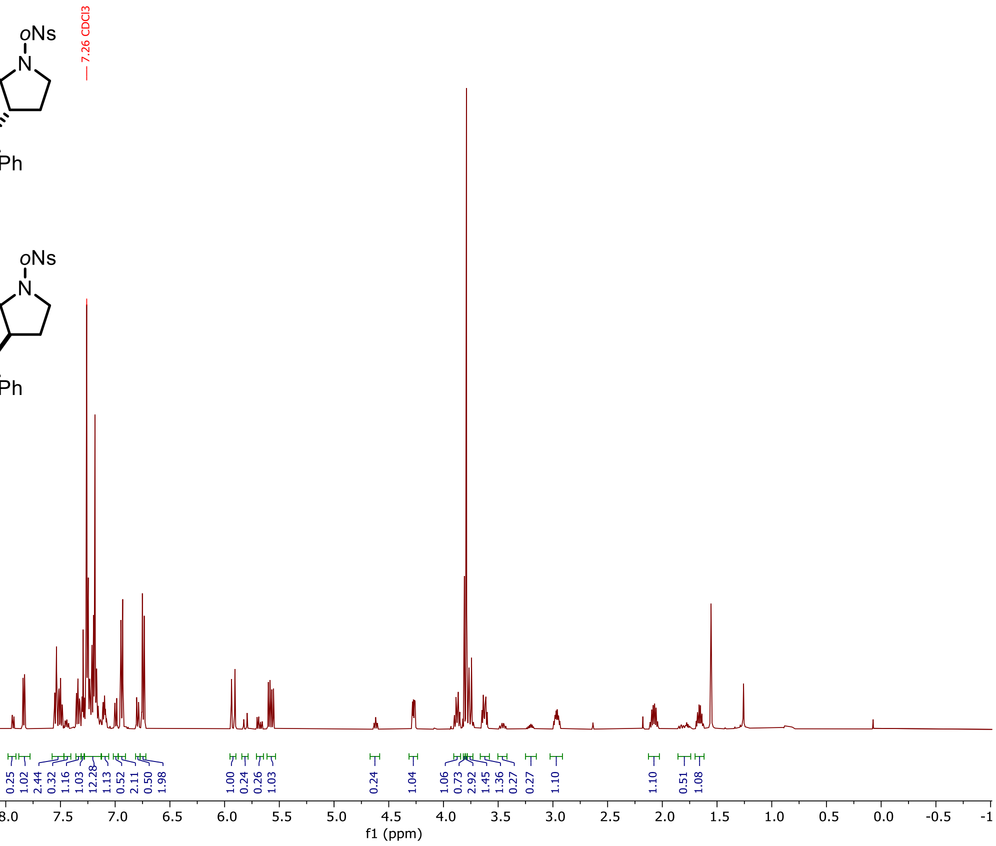
7.26 CDCl3
f1 (ppm)

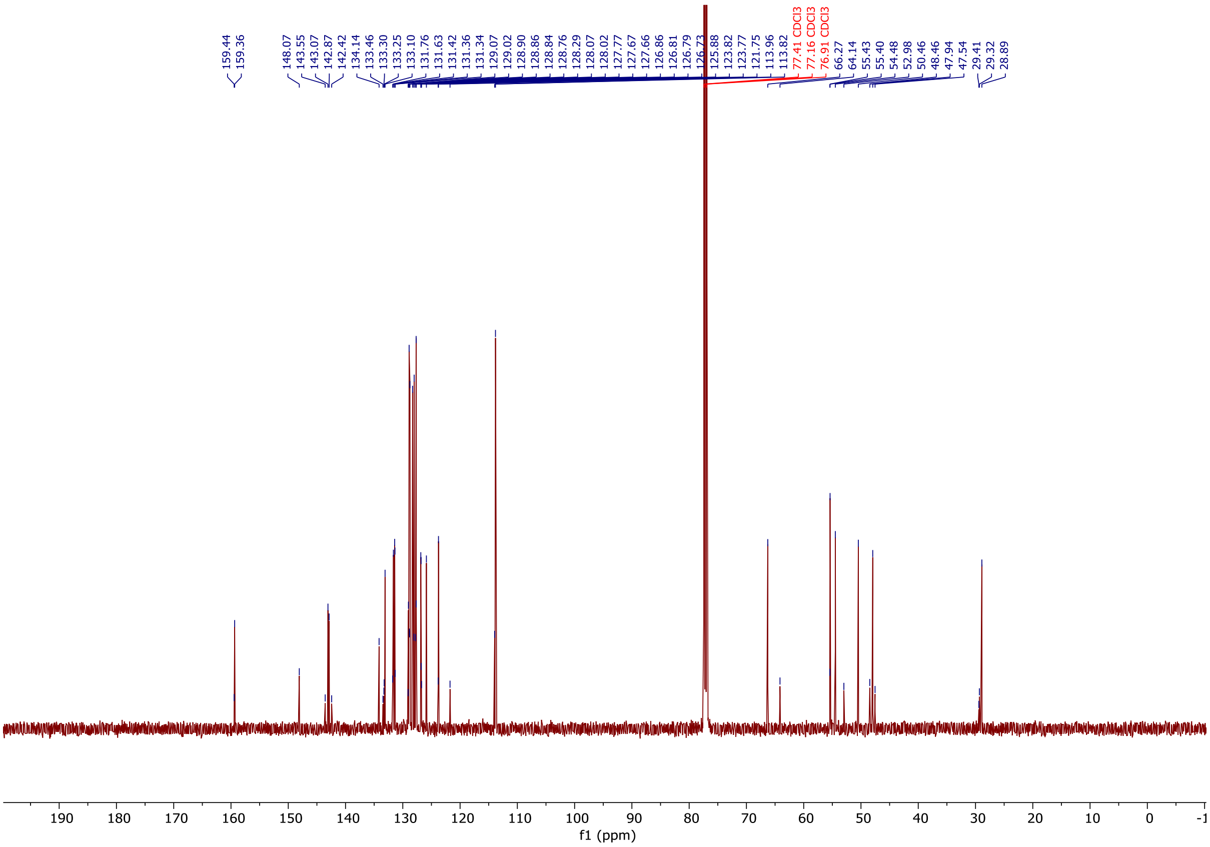
77.41 CDCl3
77.16 CDCl3
76.91 CDCl3
f1 (ppm)

(3y): (±)-(2*R*,3*R*)-2-[(1'*E*)-4-Methoxystyryl]-1-[(2-nitrophenyl)sulfonyl]-3-[(1''*R*)-3-phenylcyclopent-2-en-1-yl]pyrrolidine

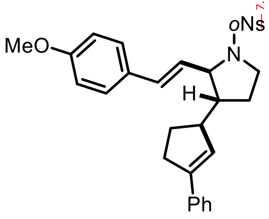

3y

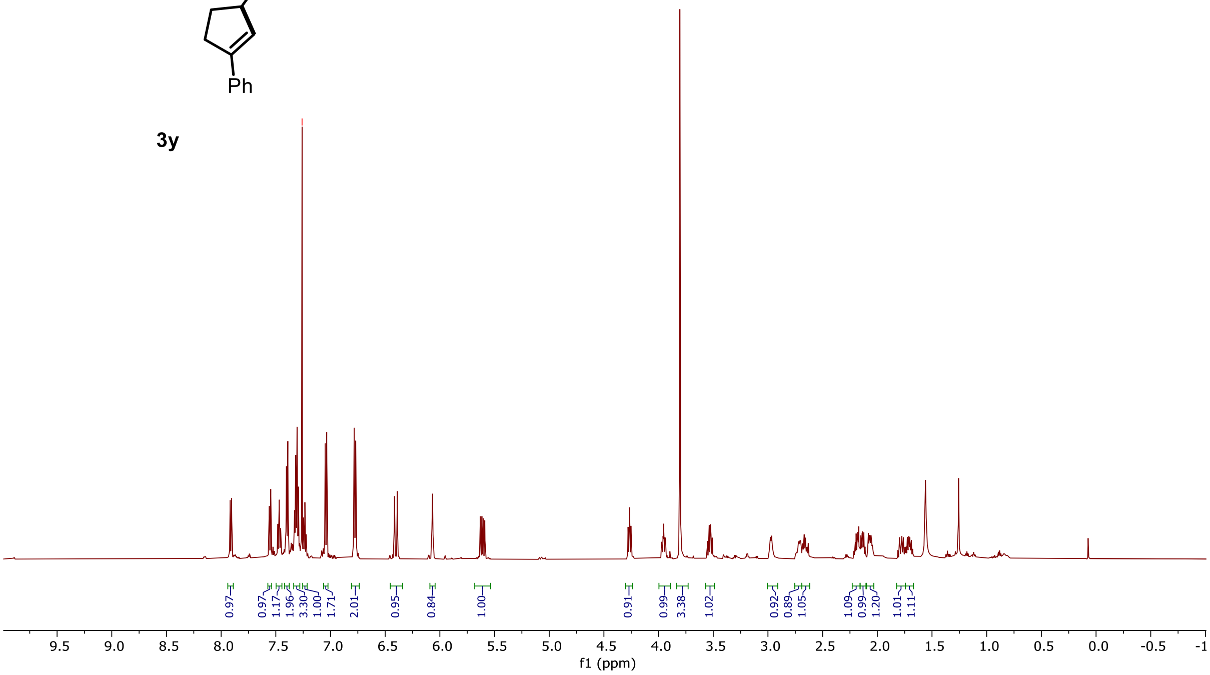

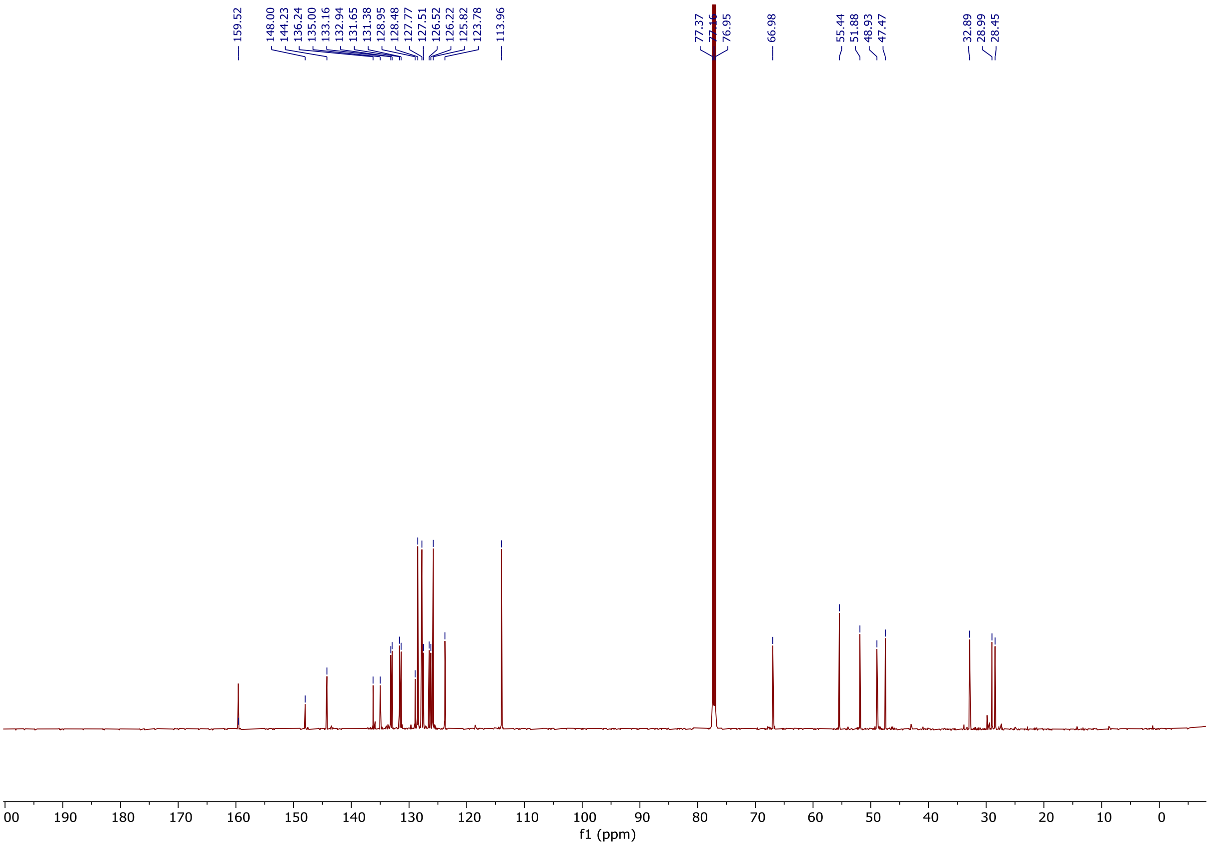

(3z): (±)-{(2*R*,3*S*)-1-[(2-Nitrophenyl)sulfonyl]-2-[(1'*E*)-styryl]pyrrolidin-3-yl}methanol and (±)-{(2*R*,3*R*)-1-[(2-Nitrophenyl)sulfonyl]-2-[(1'*E*)-styryl]pyrrolidin-3-yl}methanol

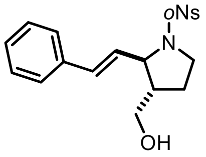

3z

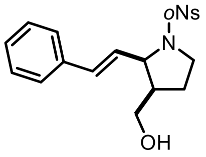

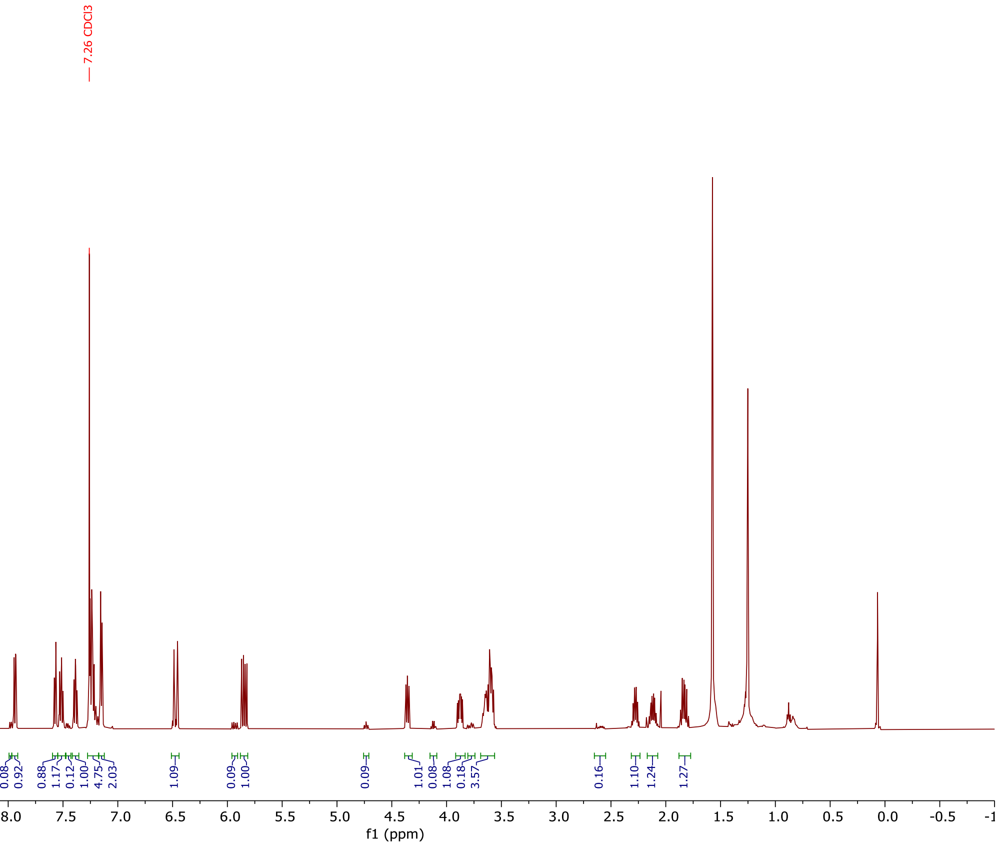

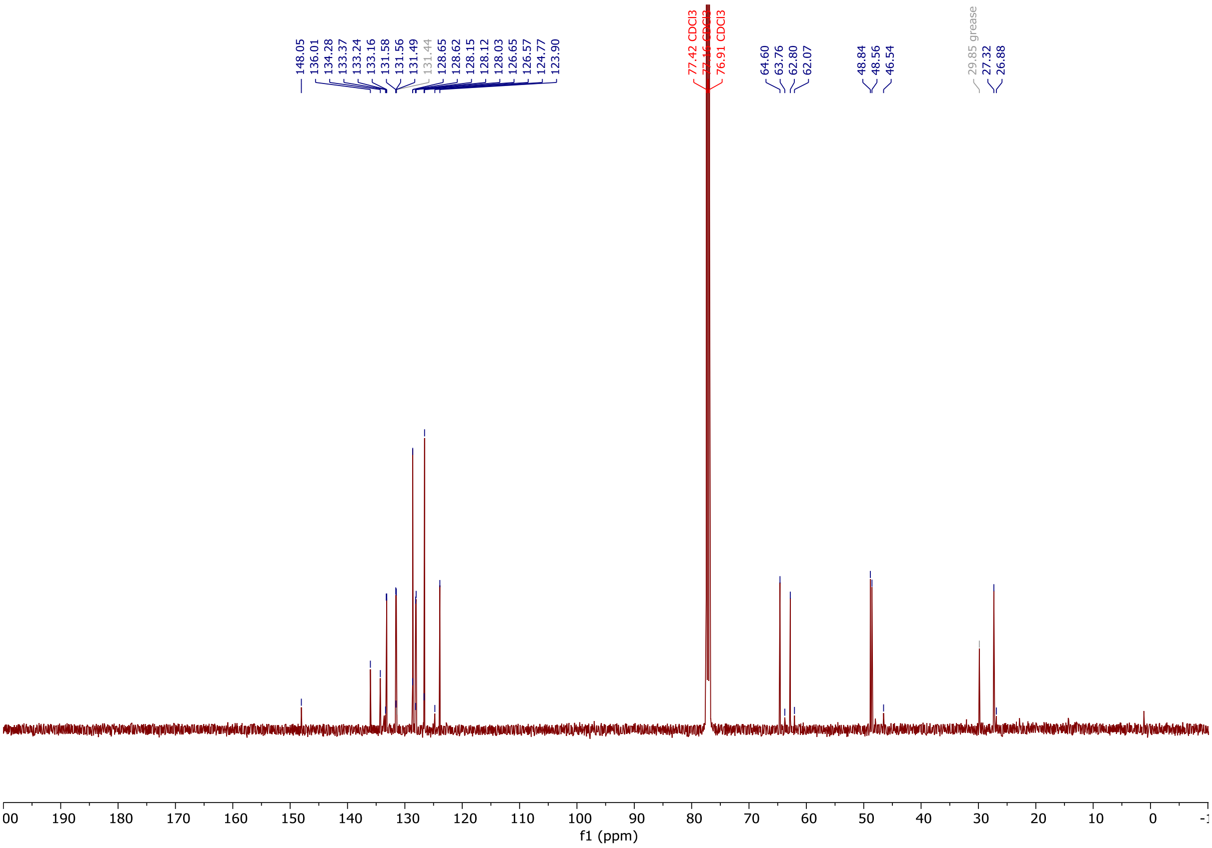

(*cis*-3aa): (±)-(2*S*,3*S*)-3-Benzhydryl-1-[(2-nitrophenyl)sulfonyl]-2-[(triisopropylsilyl)methyl]-pyrrolidine

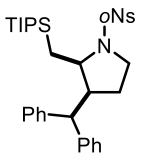

***cis*-3aa**

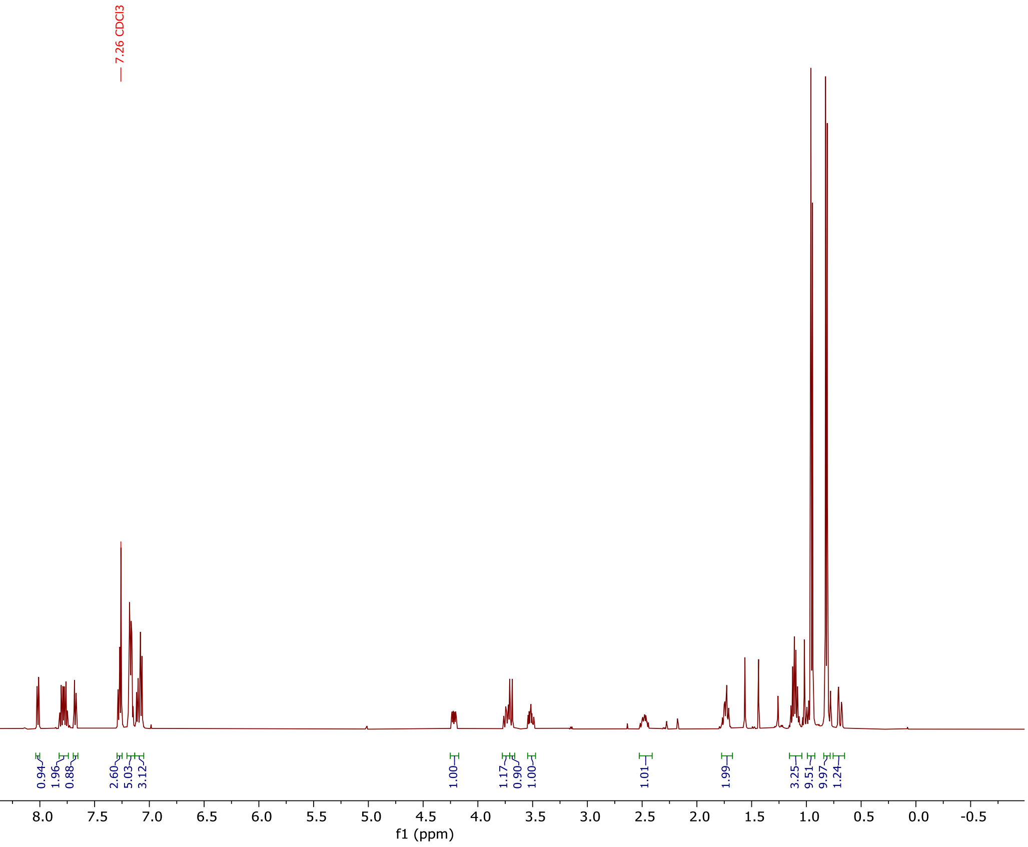

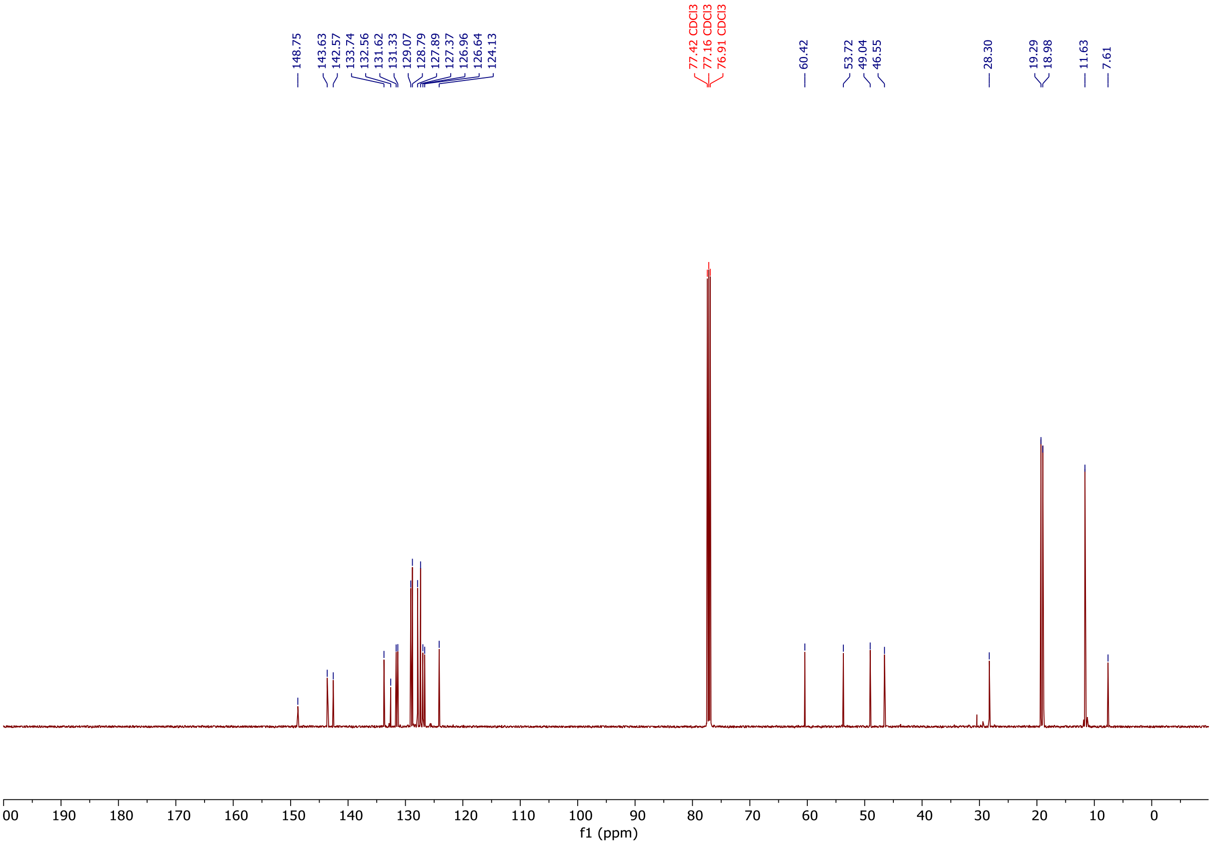

(*trans*-3aa): (±)-(2*S*,3*R*)-3-Benzhydryl-1-[(2-nitrophenyl)sulfonyl]-2-[(triisopropylsilyl)methyl]-pyrrolidine

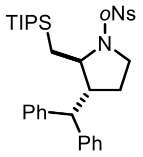

***trans*-3aa**

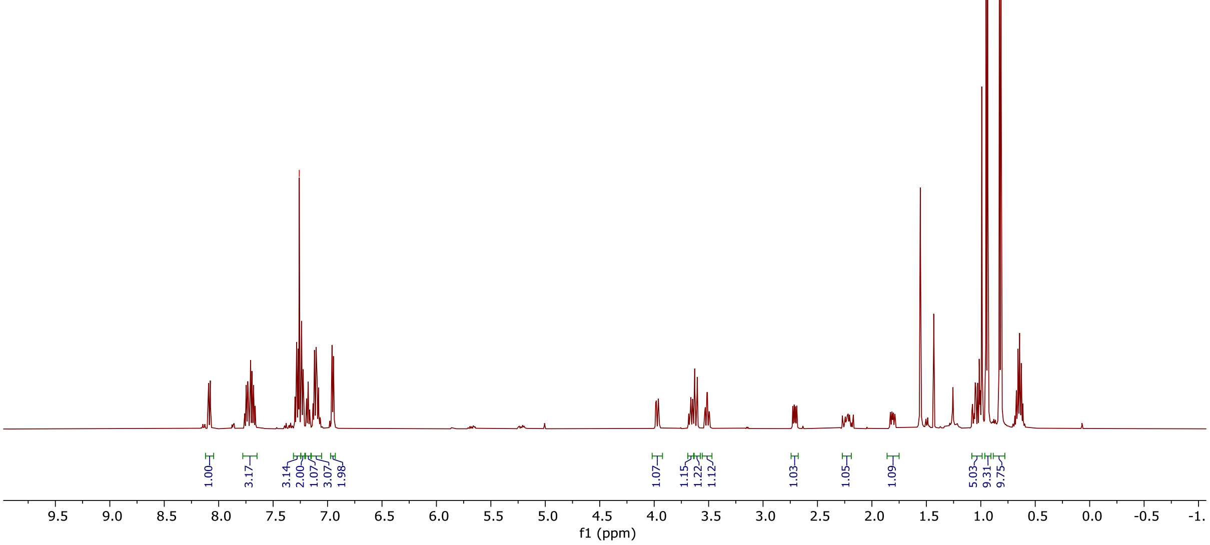

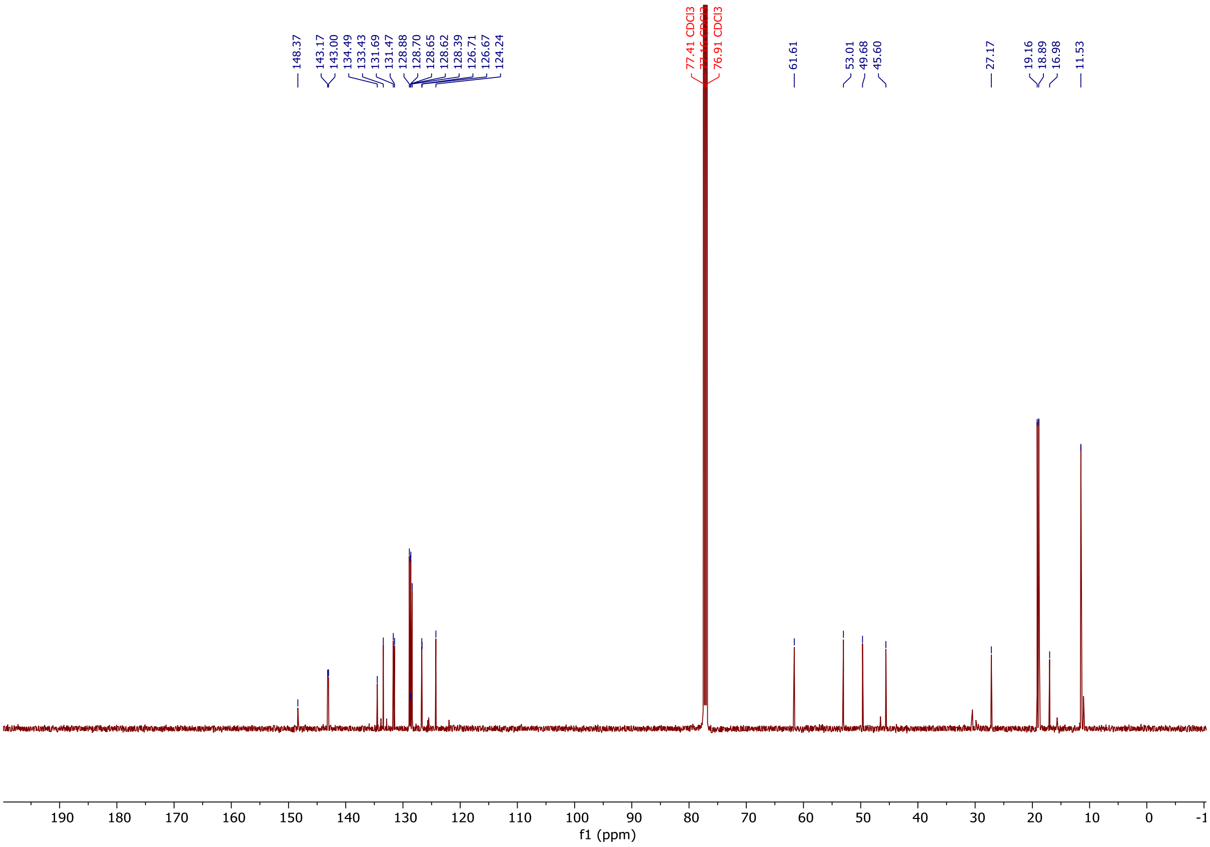

(*cis*-3ab): (±)-(2*S*,3*R*)-3-(4-Methoxybenzyl)-1-[(2-nitrophenyl)sulfonyl]-2-[(triisopropylsilyl)-methyl]-pyrrolidine

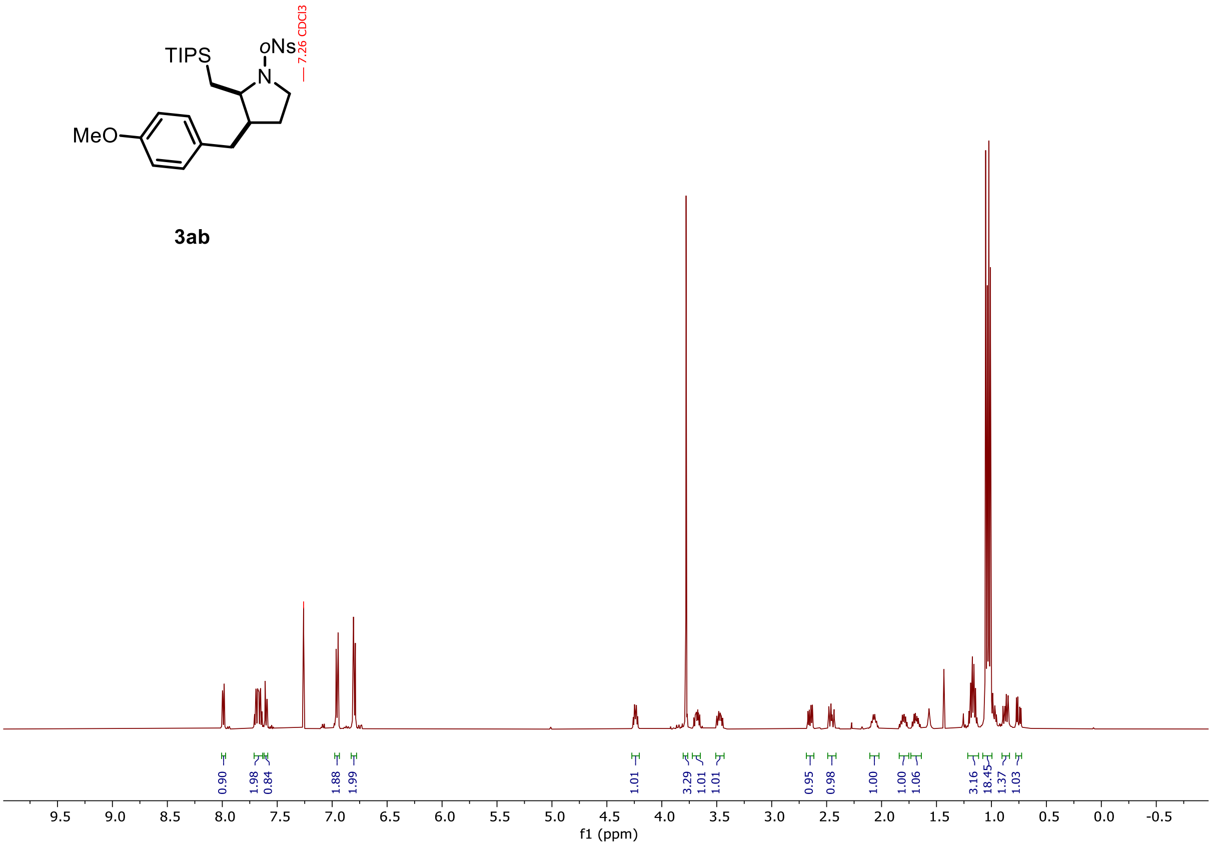

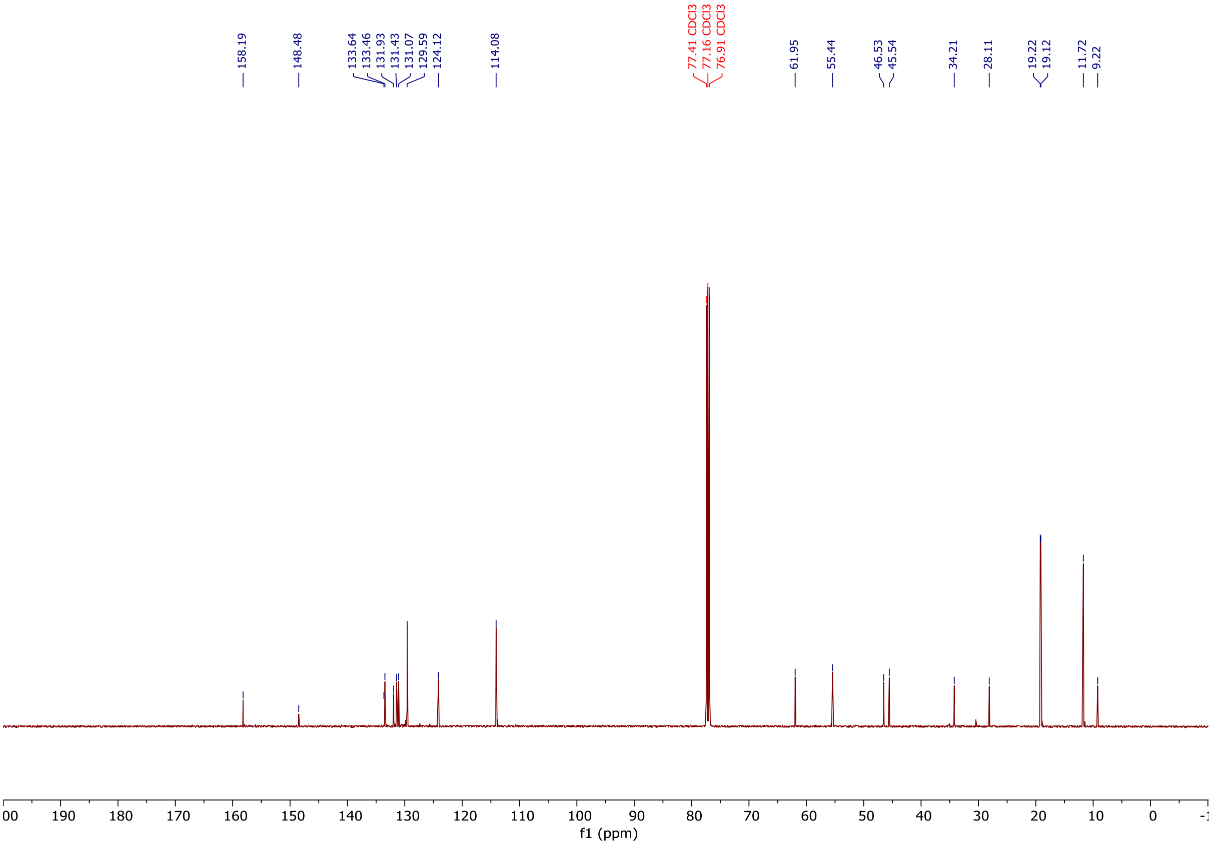

(*trans*-3ab): (±)-(2*S*,3*S*)-3-(4-Methoxybenzyl)-1-[(2-nitrophenyl)sulfonyl]-2-[(triisopropylsilyl)-methyl]-pyrrolidine

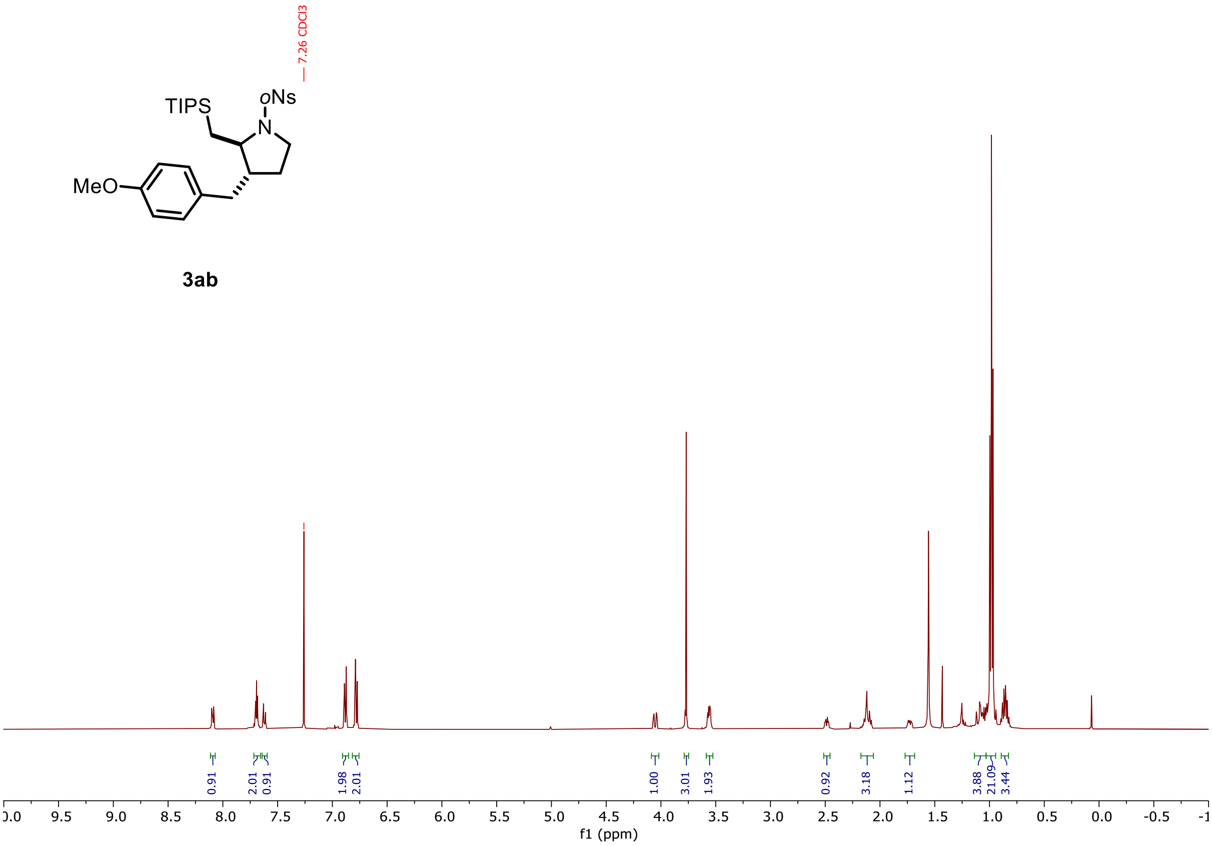

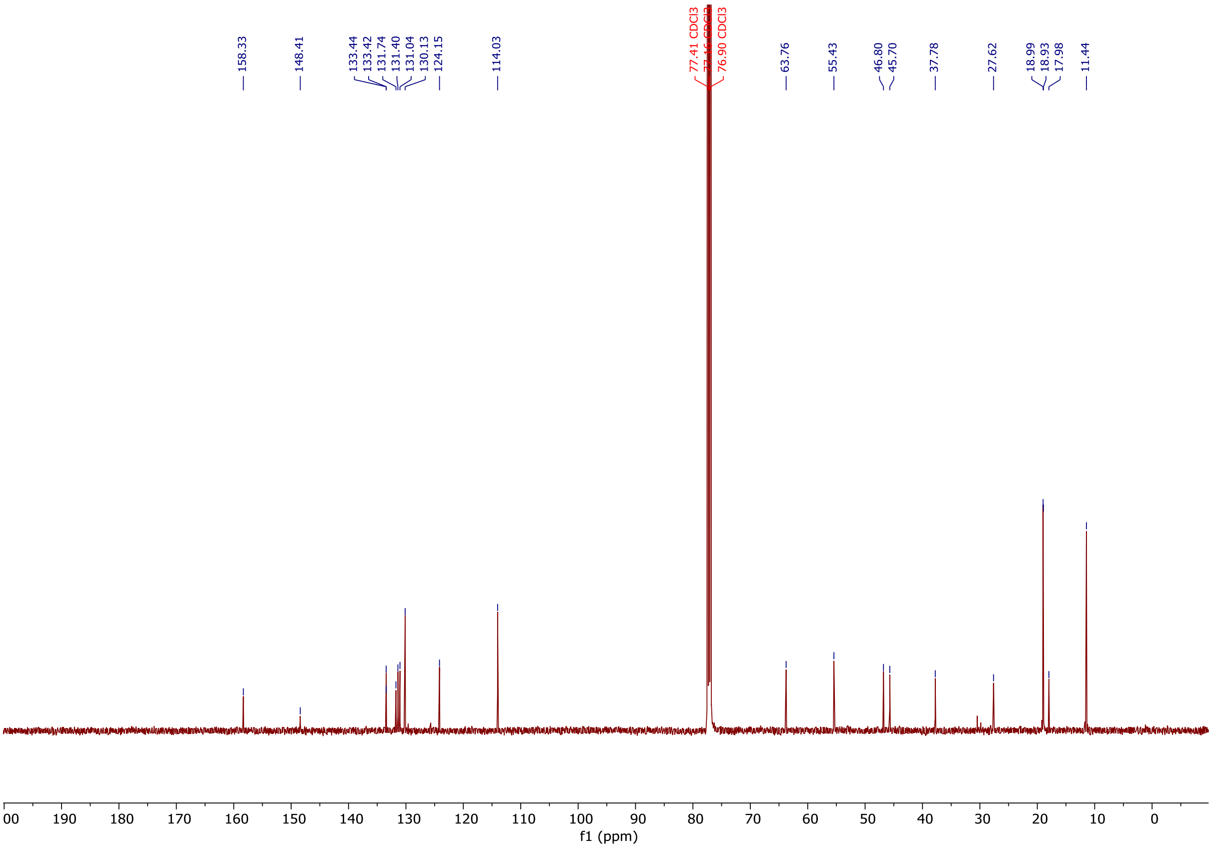

(*cis*-3ac): (±)-(2*S*,3*S*)-3-Benzhydryl-2-[(*tert*-butyldiphenylsilyl)methyl]-1-[(2-nitrophenyl)-sulfonyl]-pyrrolidine

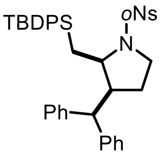

3ac

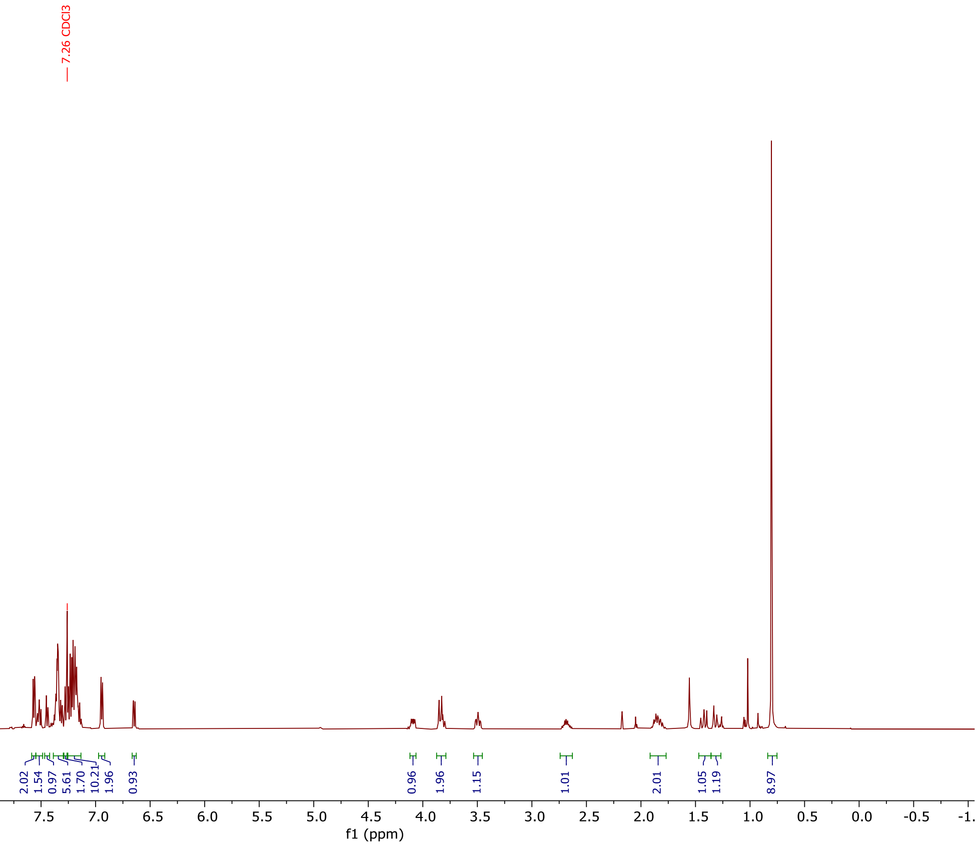

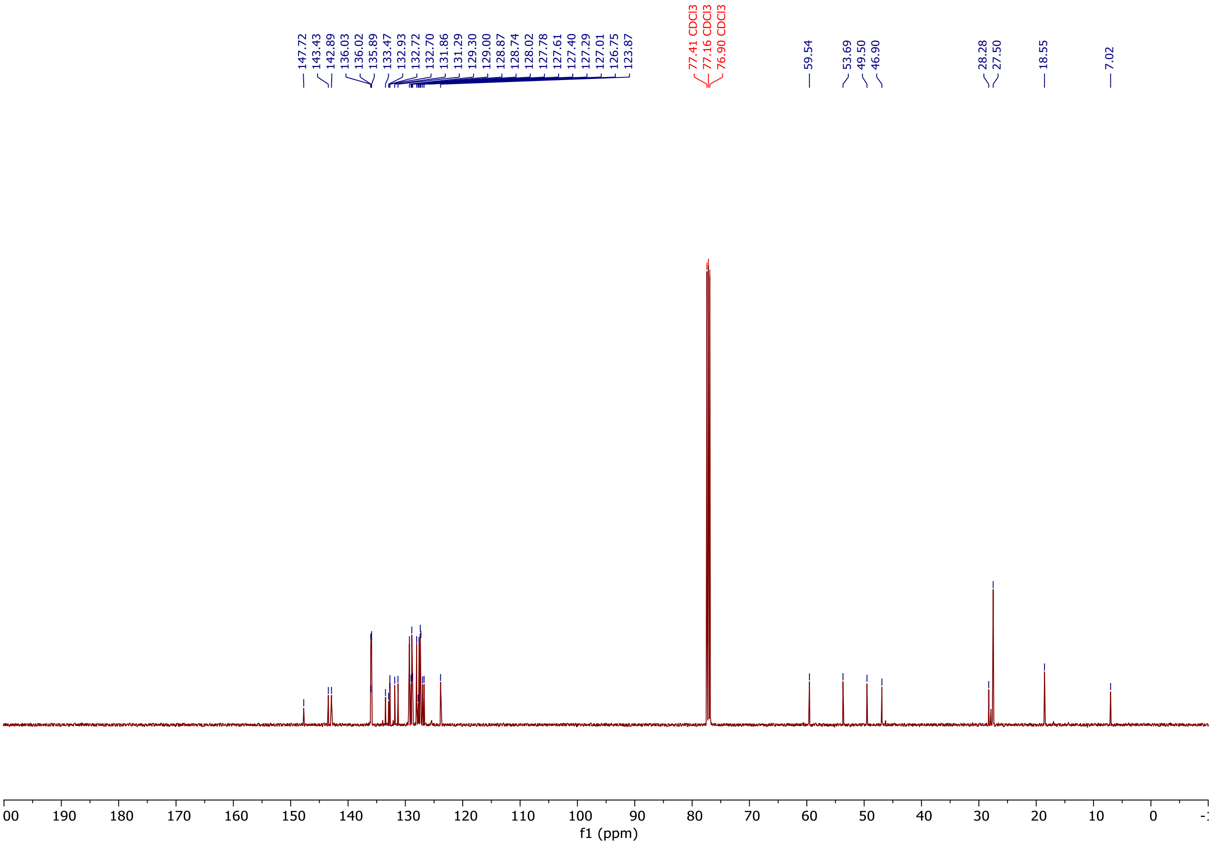

(*trans*-3ac): (±)-(2*S*,3*R*)-3-Benzhydryl-2-[(*tert*-butyldiphenylsilyl)methyl]-1-[(2-nitrophenyl)-sulfonyl]-pyrrolidine

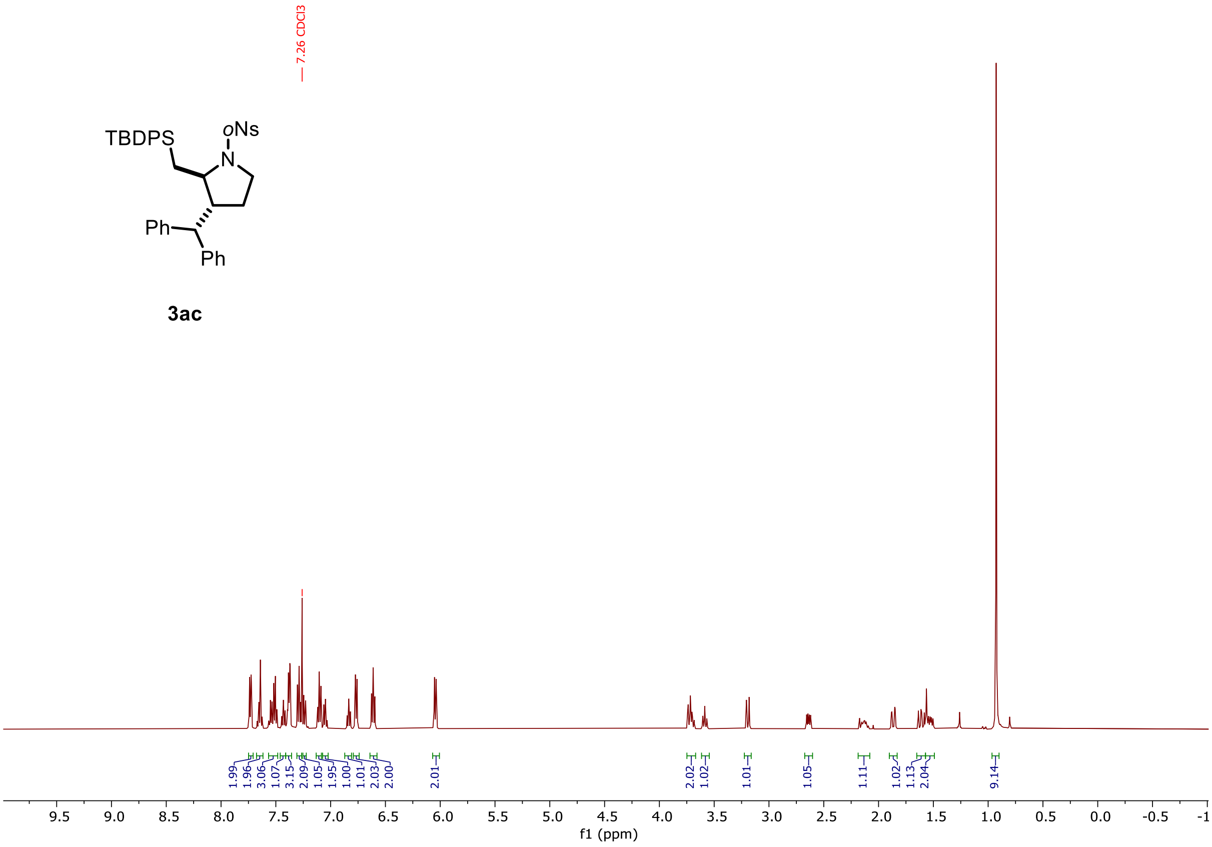

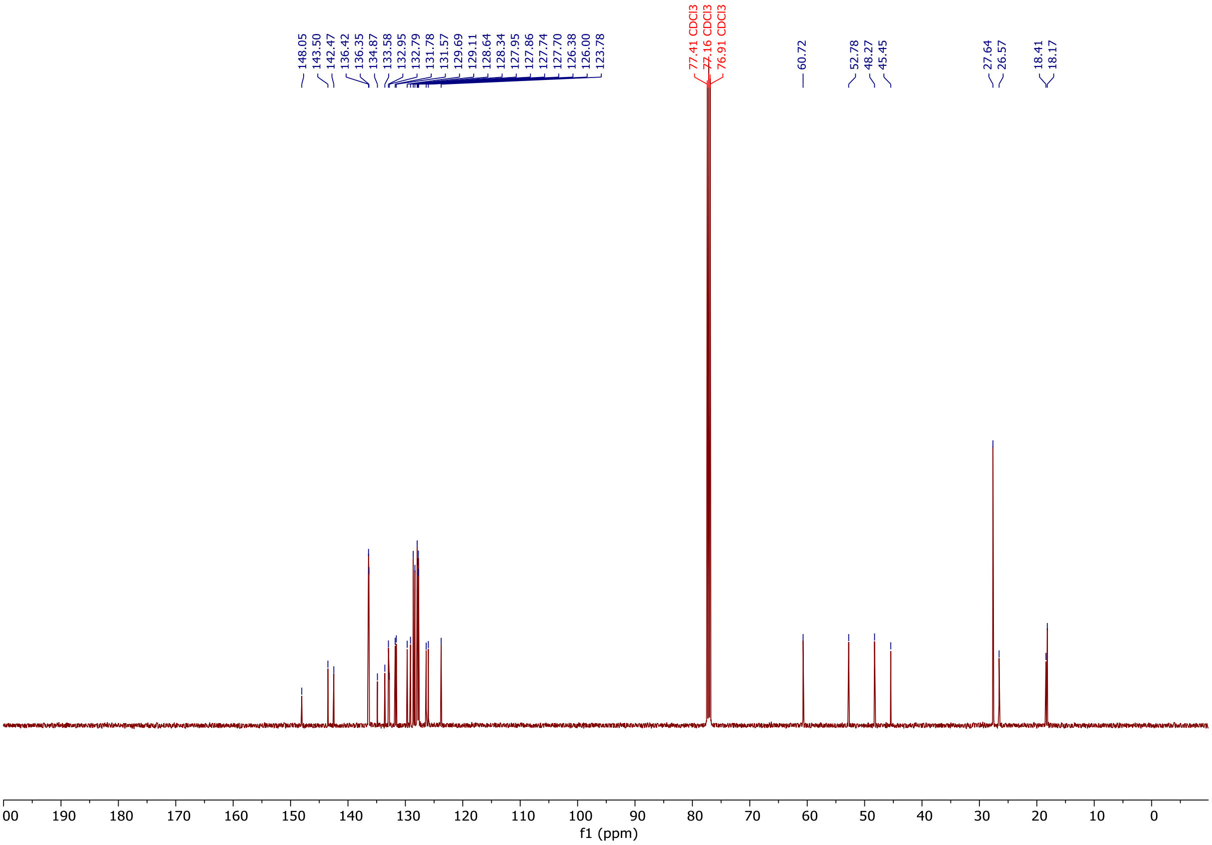

((–)-3ad): (–)-(2*R*,3*R*,4*S*)-3-Benzhydryl-2-(4-methoxyphenyl)-1-[(2-nitrophenyl)sulfonyl]-4-phenyl-pyrrolidine and (–)-(2*S*,3*S*,4*S*)-3-Benzhydryl-2-(4-methoxyphenyl)-1-[(2-nitro-phenyl)sulfonyl]-4-phenylpyrrolidine

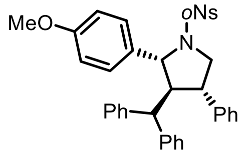

(-)-3ad

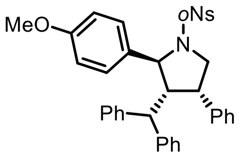

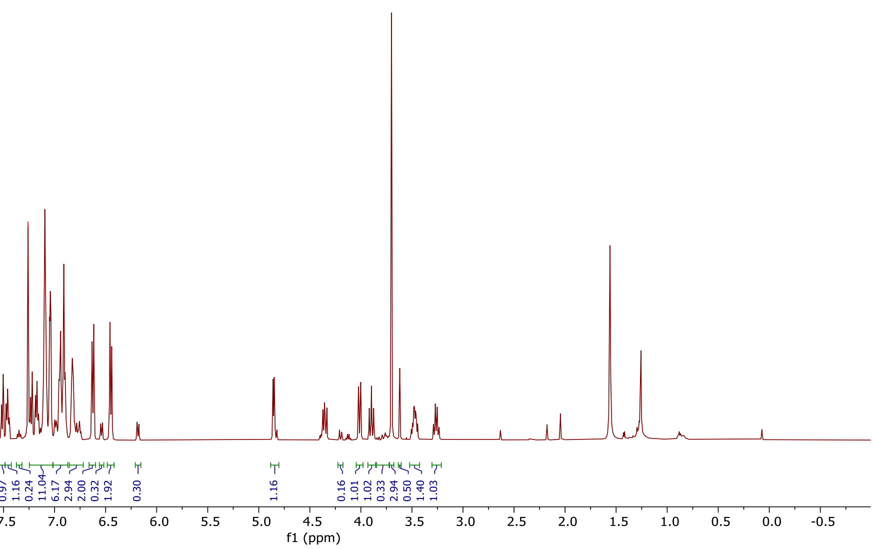

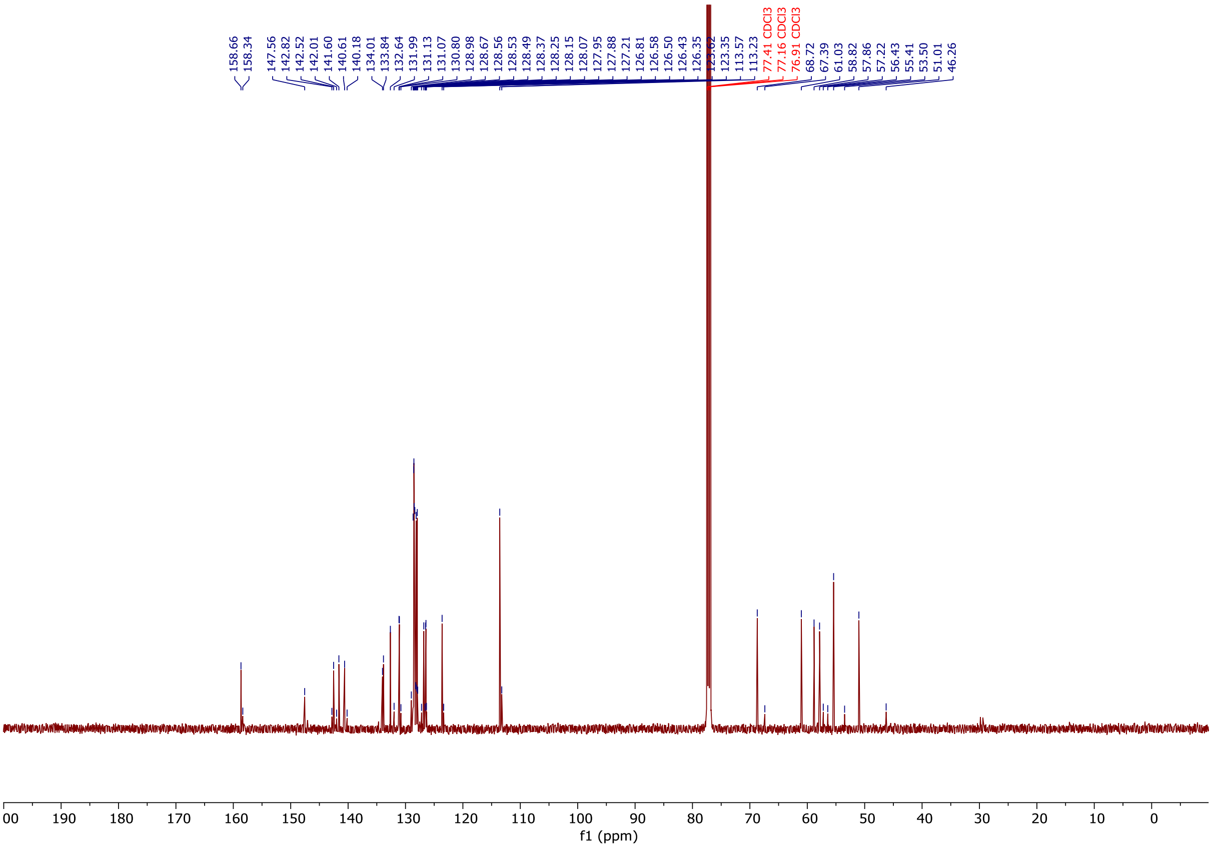

((–)-3ae): (–)-(2*R*,3*R*,4*S*)-3-(4-Methoxybenzyl)-2-(4-methoxyphenyl)-1-[(2-nitrophenyl)sulfonyl]-4-phenylpyrrolidine and (–)-(2*S*,3*S*,4*S*)-3-(4-Methoxybenzyl)-2-(4-methoxyphenyl)-1-[(2-nitrophenyl)sulfonyl]-4-phenylpyrrolidine

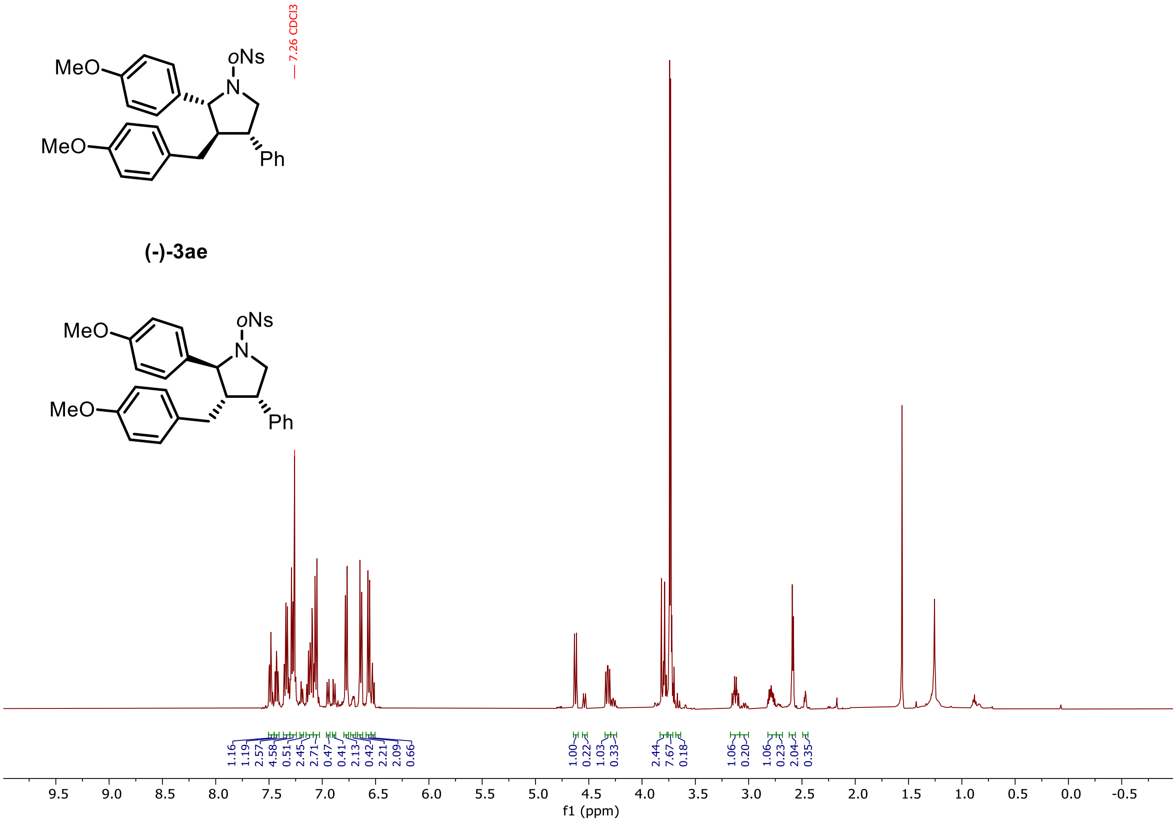

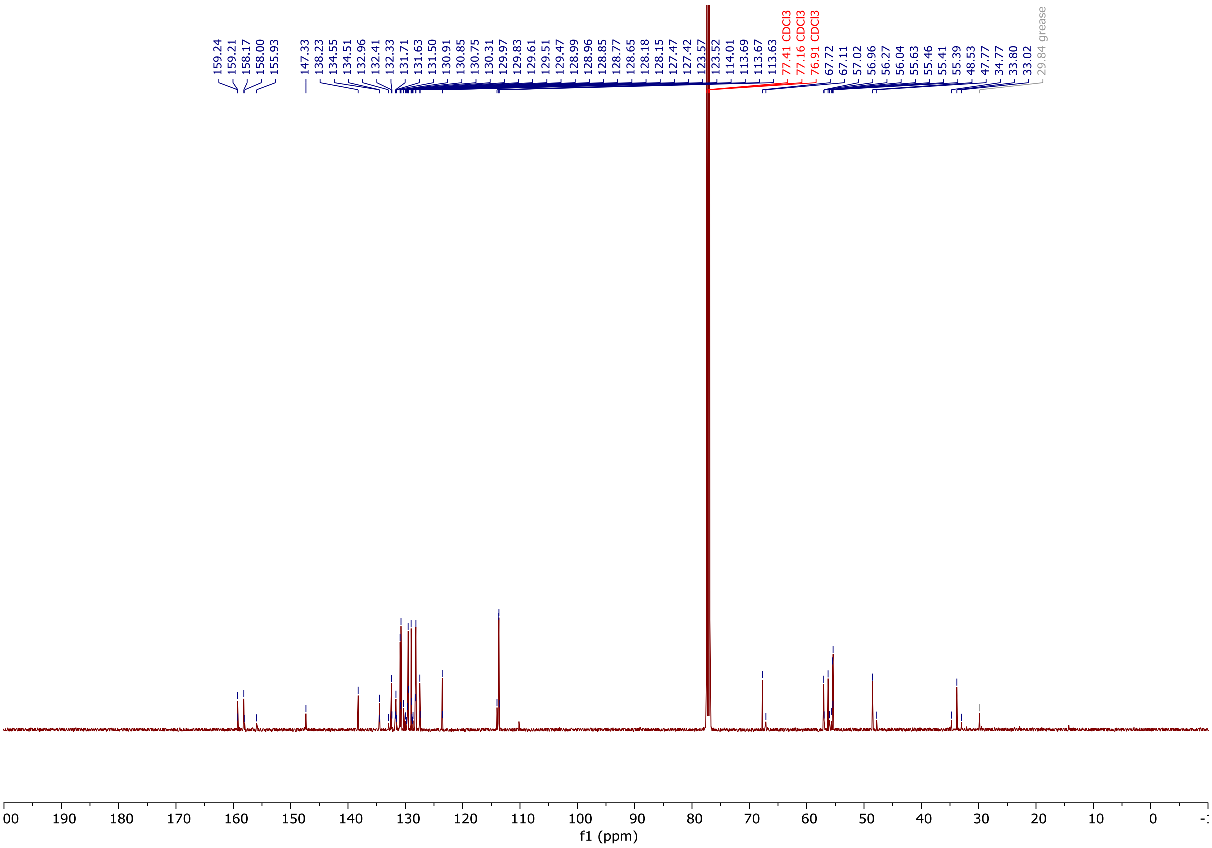

((–)-3af): (–)-(2*R*,3*R*,4*S*)-2-(4-Methoxyphenyl)-1-[(2-nitrophenyl)sulfonyl]-4-phenyl-3-[(1’*S*)-3-phenylcyclopent-2-en-1-yl]pyrrolidine and (–)-(2*S*,3*S*,4*S*)-2-(4-Methoxyphenyl)-1-[(2-nitrophenyl)sulfonyl]-4-phenyl-3-[(1’*R*)-3-phenylcyclopent-2-en-1-yl]pyrrolidine

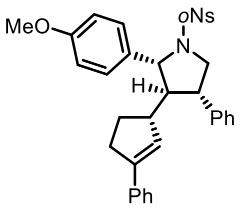

(-)-3af

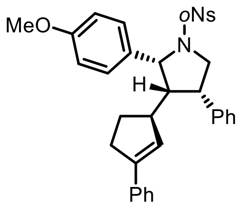

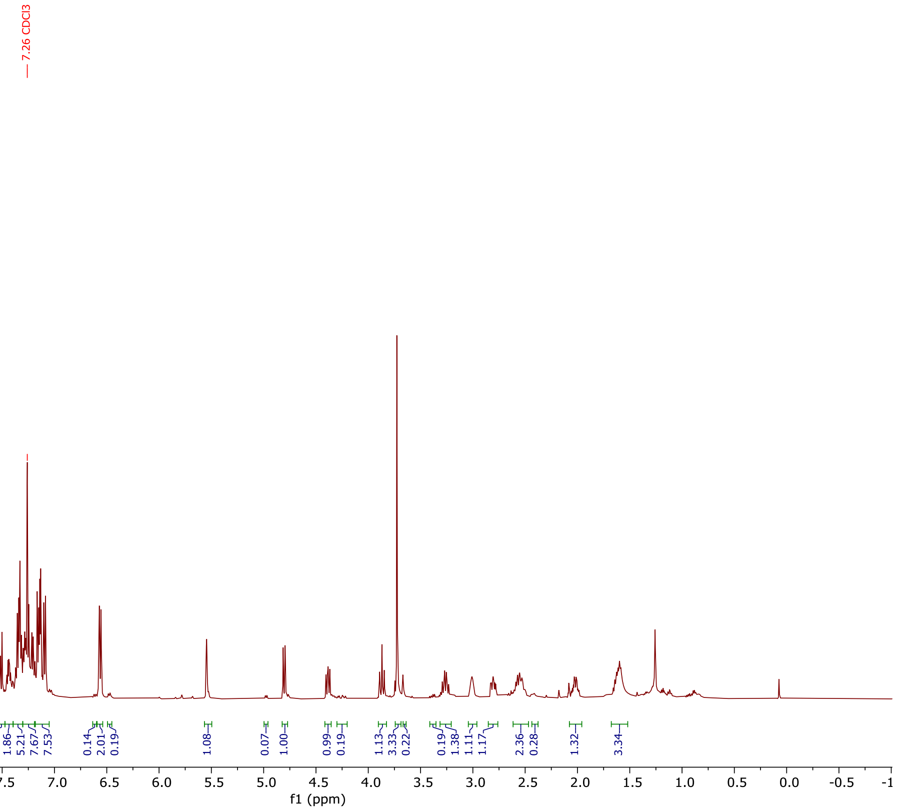

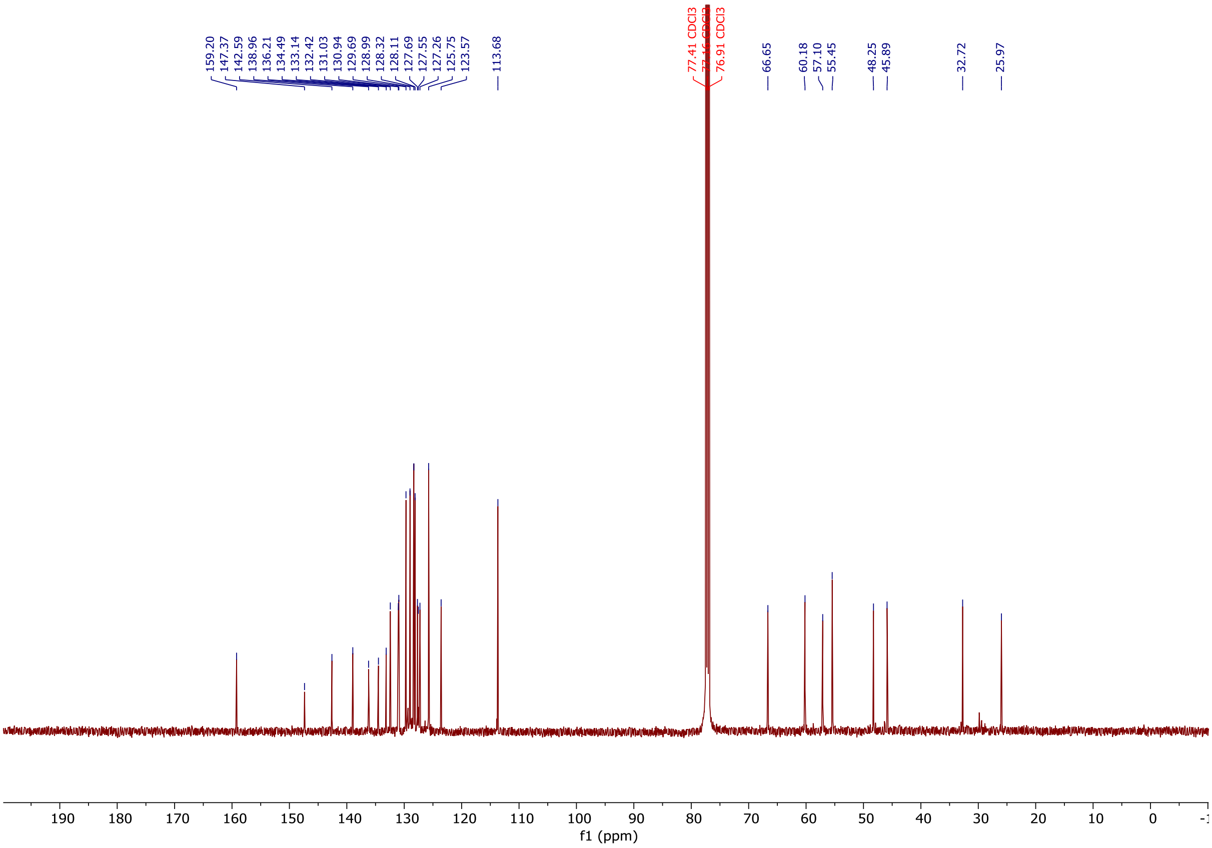

(5-Ts): (±)-(2*S*,3*R*)-3-Benzhydryl-2-(4-methoxyphenyl)-1-tosylpiperidine and (±)-(2*S*,3*S*)-3-Benzhydryl-2-(4-methoxyphenyl)-1-tosylpiperidine

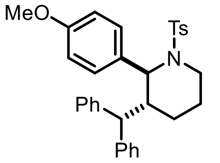

5-Ts

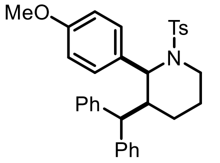

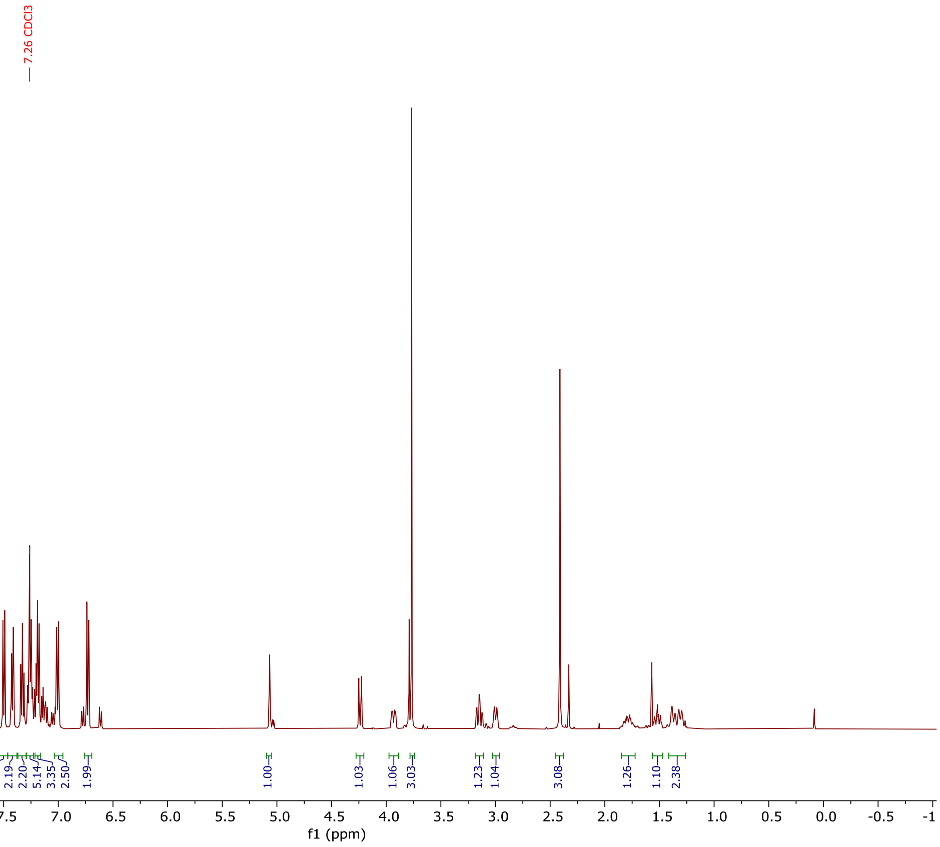

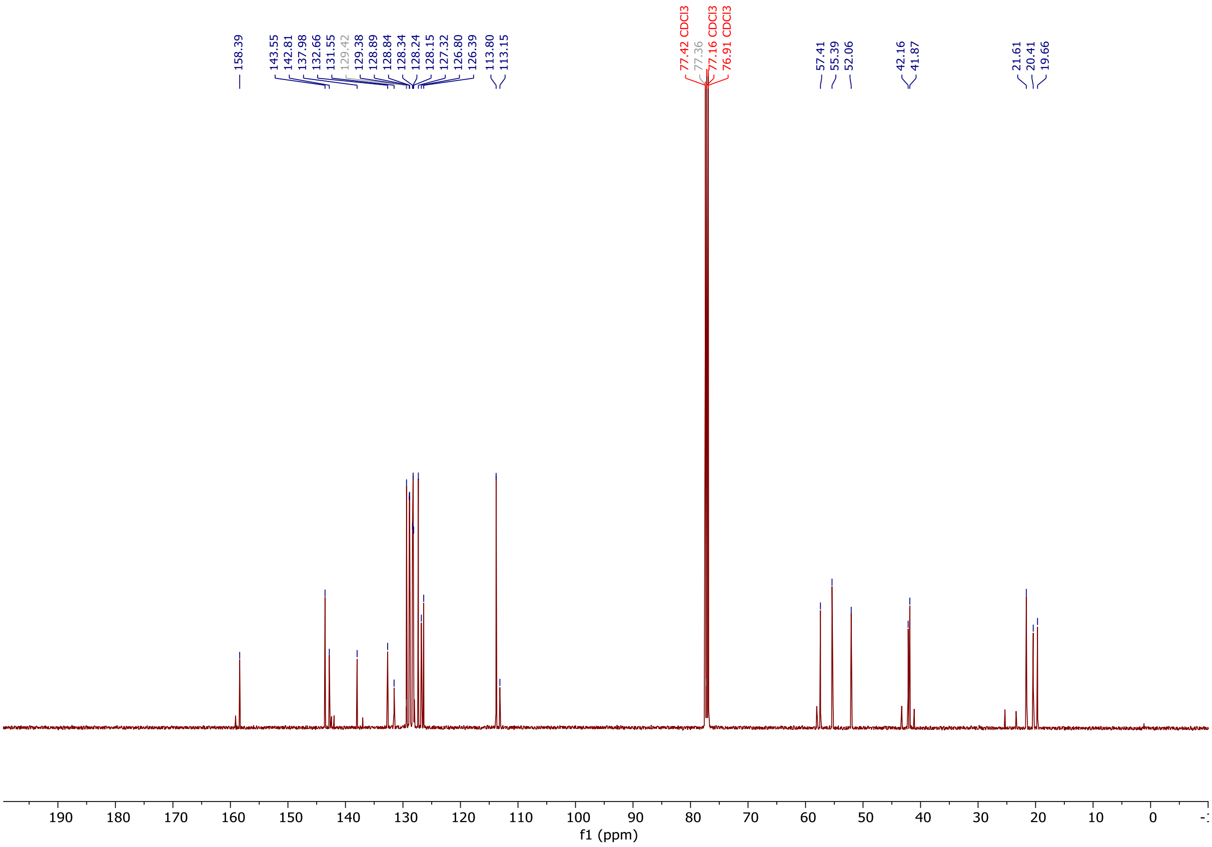

(5-Ms): (±)-(2*S*,3*R*)-3-Benzhydryl-2-(4-methoxyphenyl)-1-(methylsulfonyl)piperidine and (±)-(2*S*,3*R*)-3-Benzhydryl-2-(4-methoxyphenyl)-1-(methylsulfonyl)piperidine

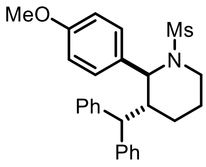

5-Ms

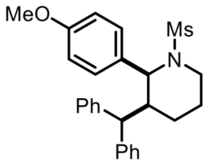

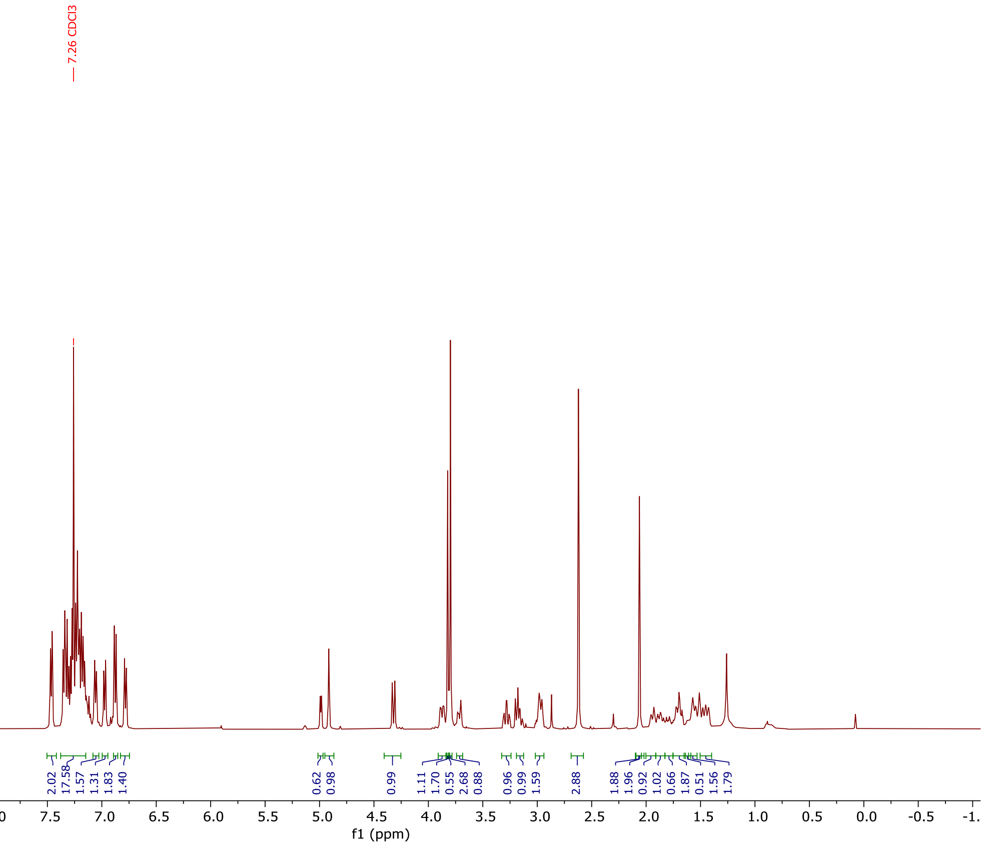

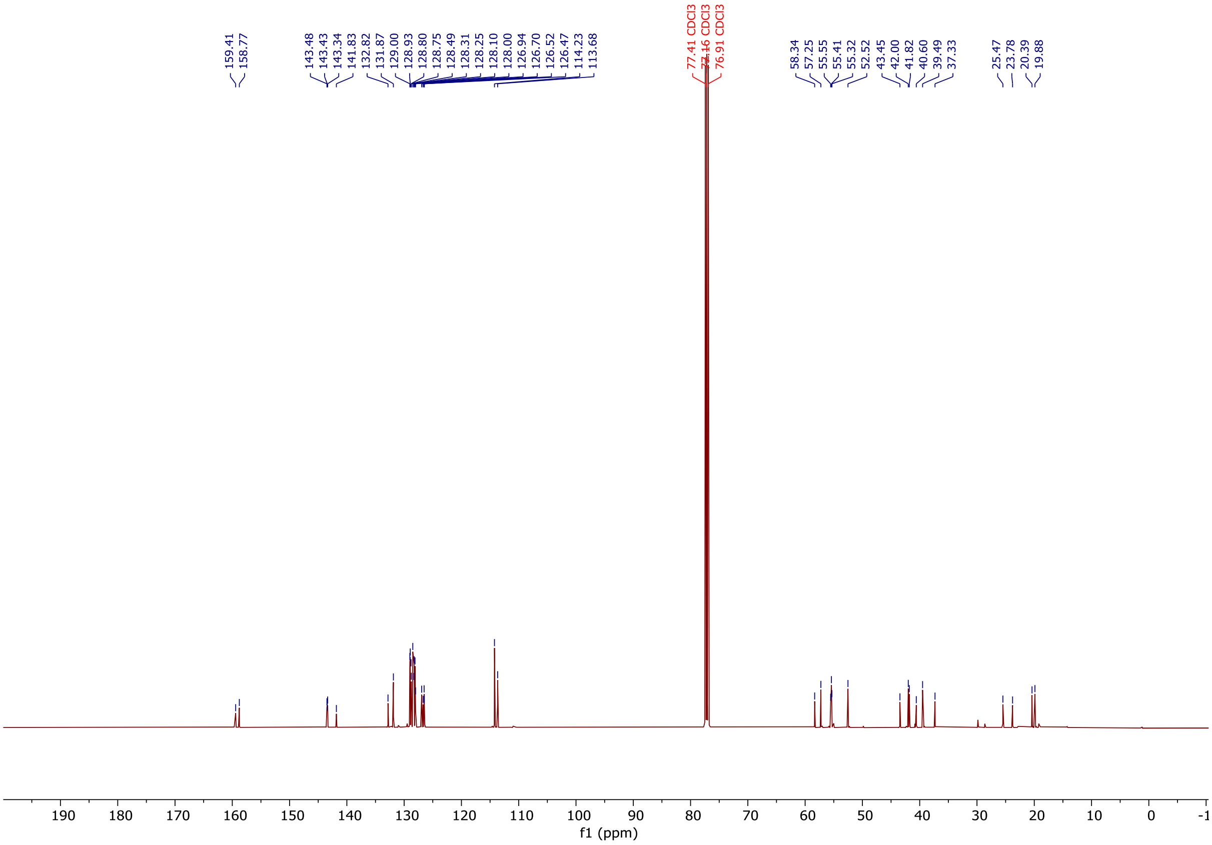

(5-*o*Ns-A): (±)-(2*S*,3*S*)-3-Benzhydryl-2-(4-methoxyphenyl)-1-((2-nitrophenyl)sulfonyl)piperidine

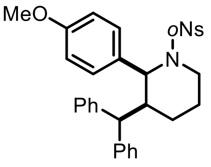

5-*o*Ns-A

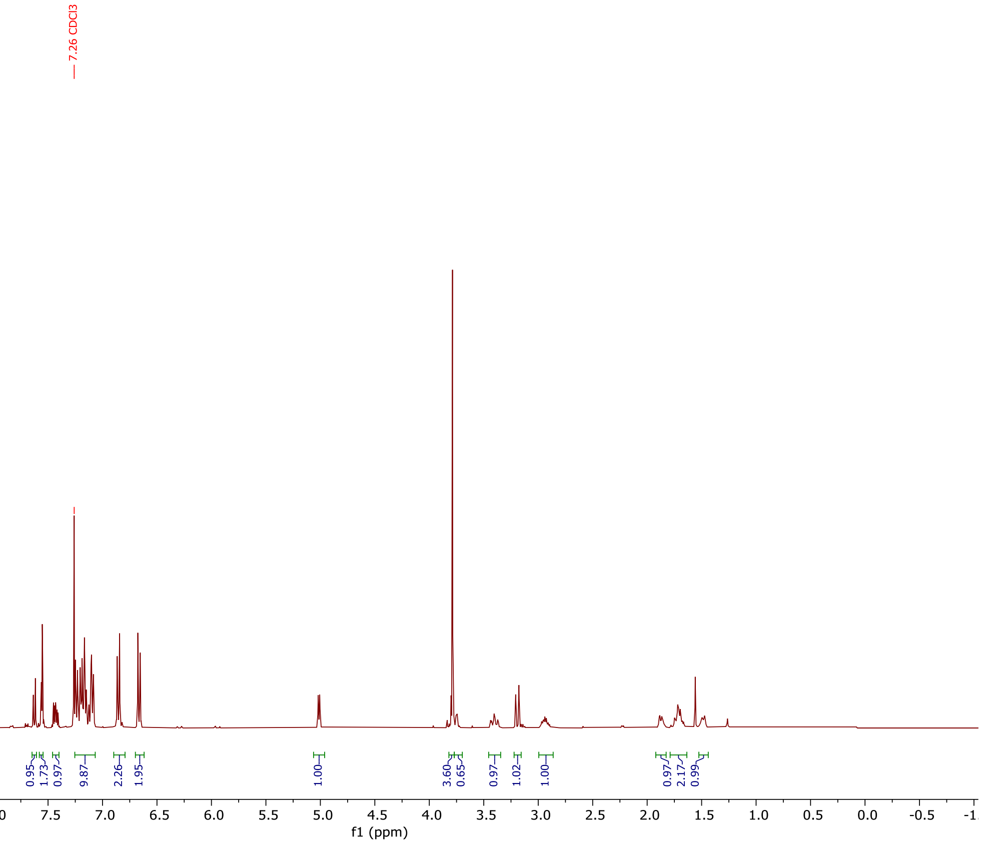

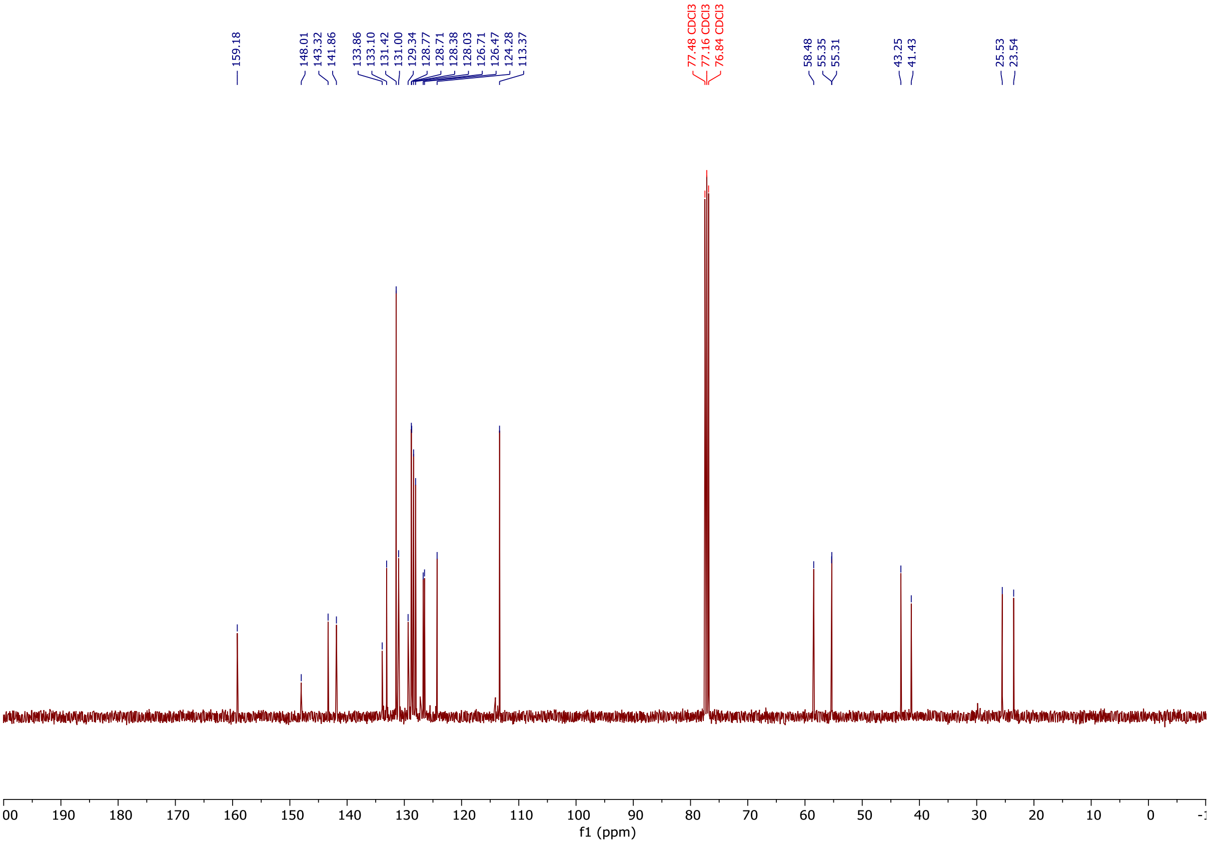

(5-*o*Ns-B): (±)-(2*S*,3*R*)-3-Benzhydryl-2-(4-methoxyphenyl)-1-((2-nitrophenyl)sulfonyl)piperidine

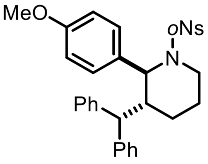

5-*o*Ns-B

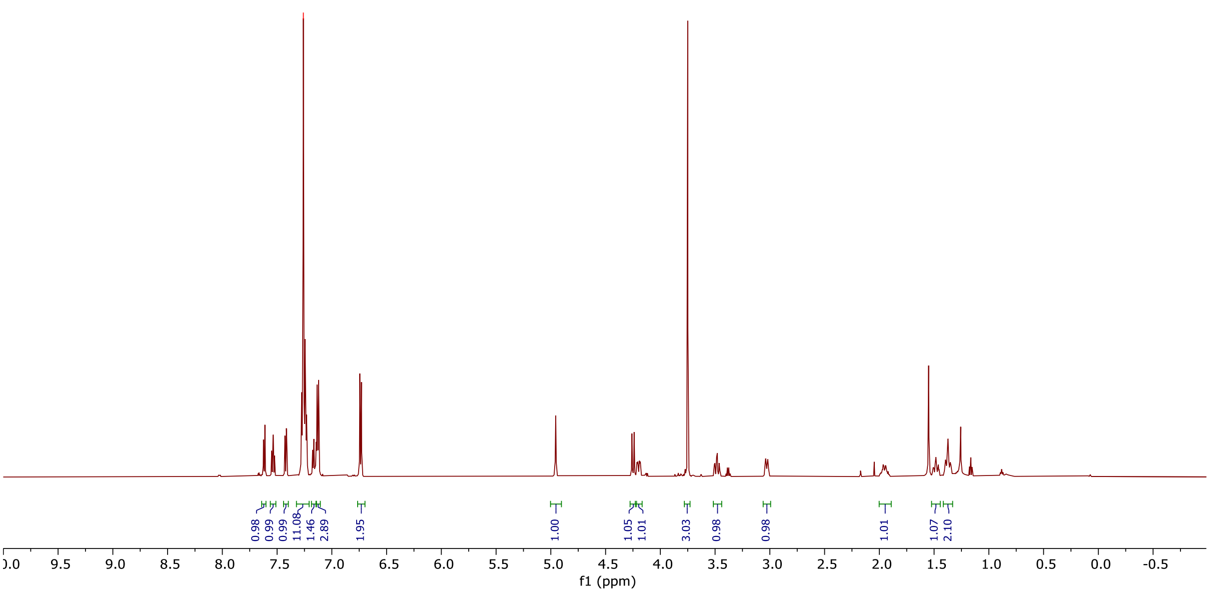

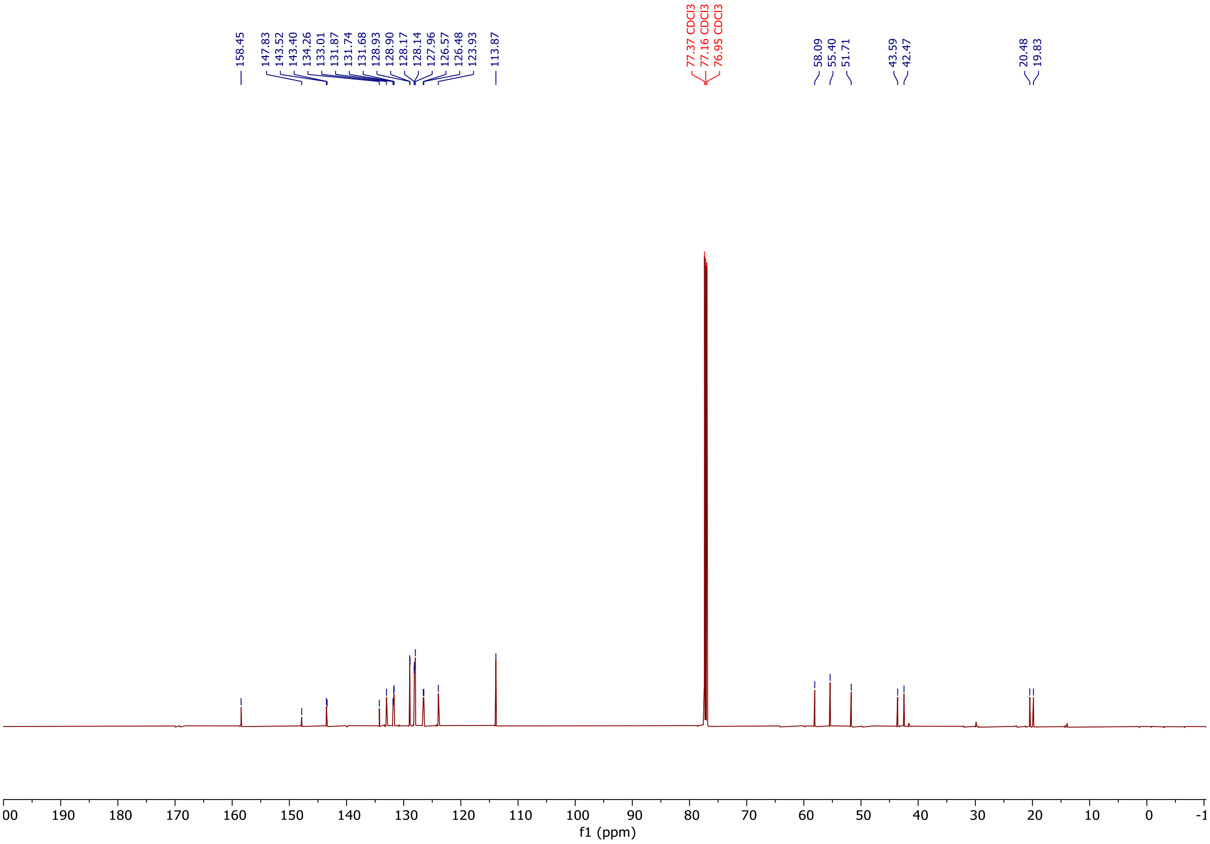

(5-*p*Ns): (±)-(2*S*,3*R*)-3-Benzhydryl-2-(4-methoxyphenyl)-1-((2-nitrophenyl)sulfonyl)piperidine and (±)-(2*S*,3*S*)-3-Benzhydryl-2-(4-methoxyphenyl)-1-((2-nitrophenyl)sulfonyl)piperidine

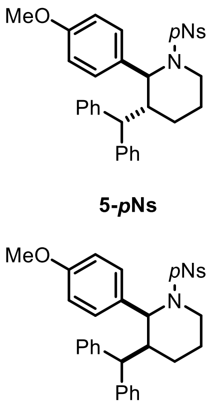

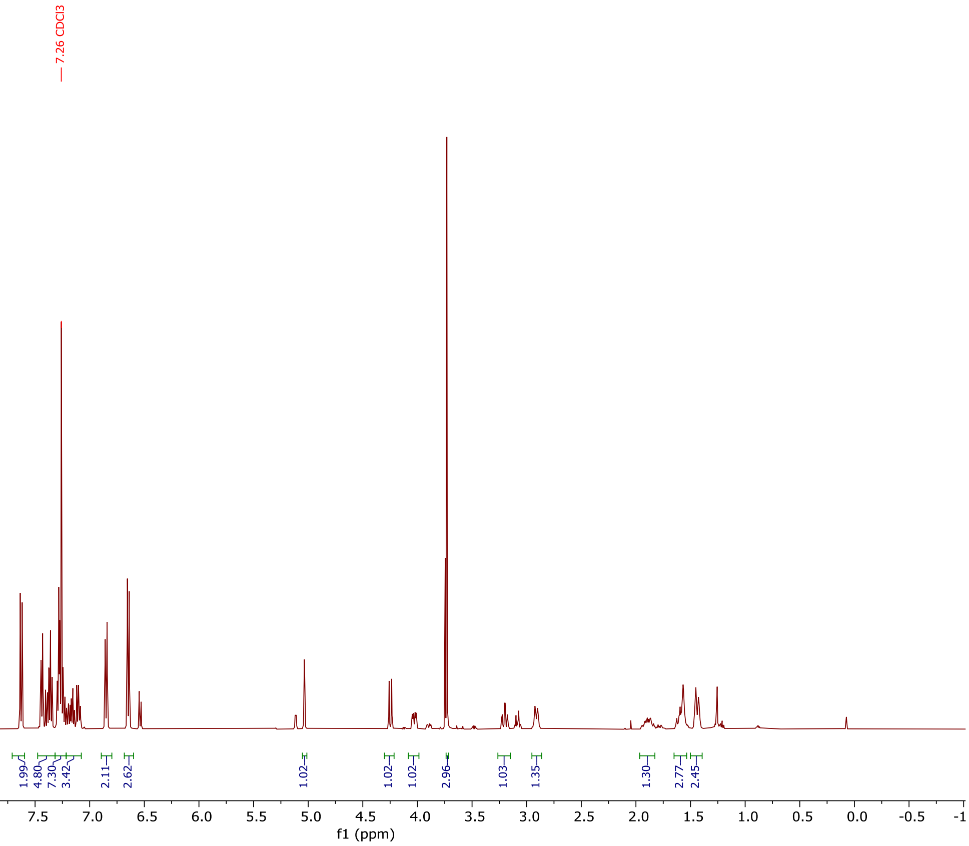

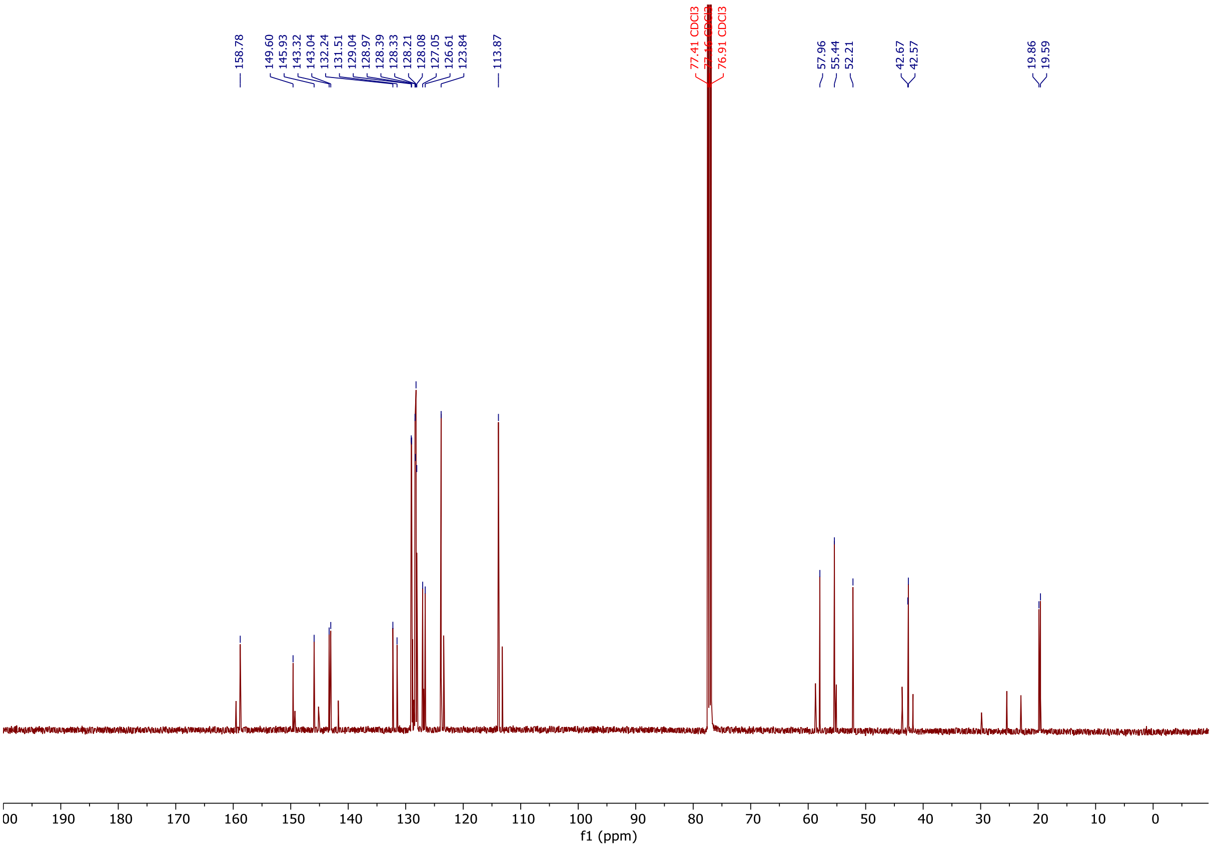

(5-Cbz): (±)-Benzyl (2*S*,3*R*)-3-benzhydryl-2-(4-methoxyphenyl)piperidine-1-carboxylate

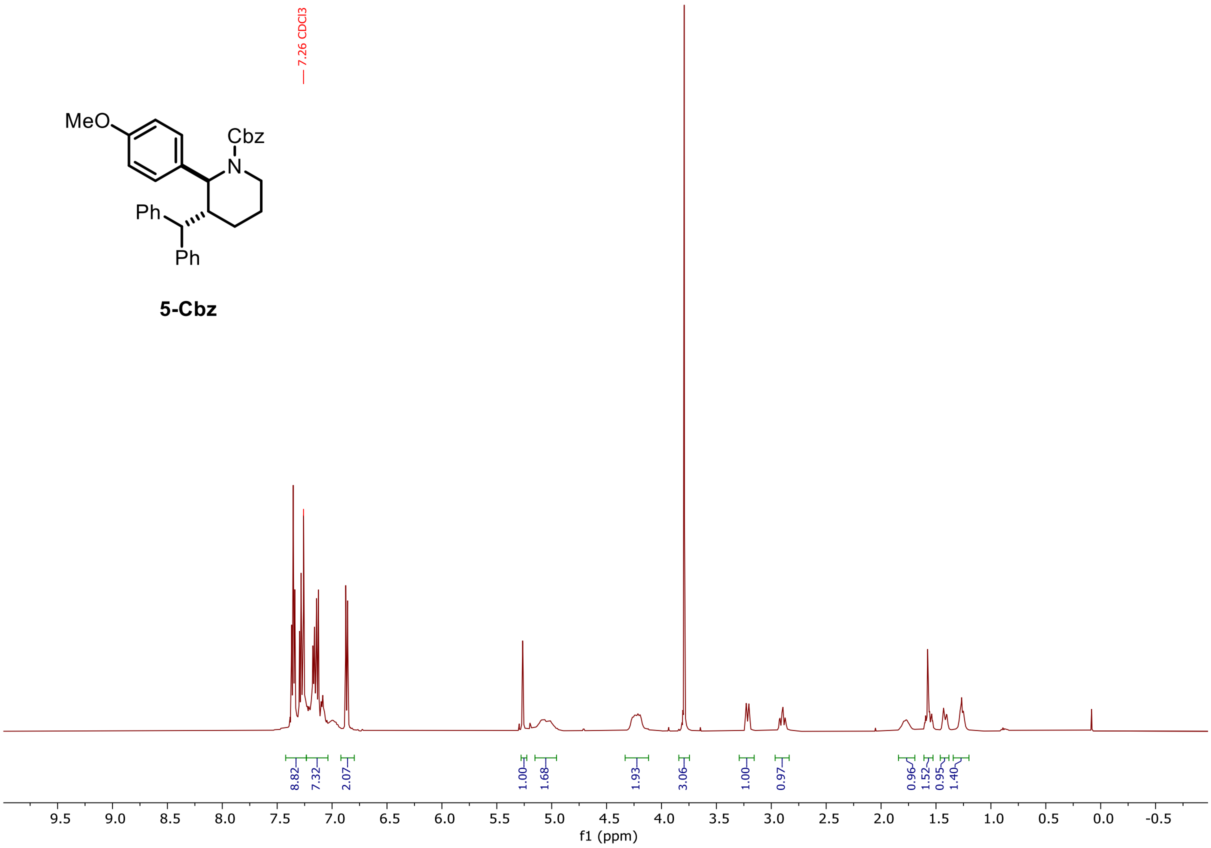

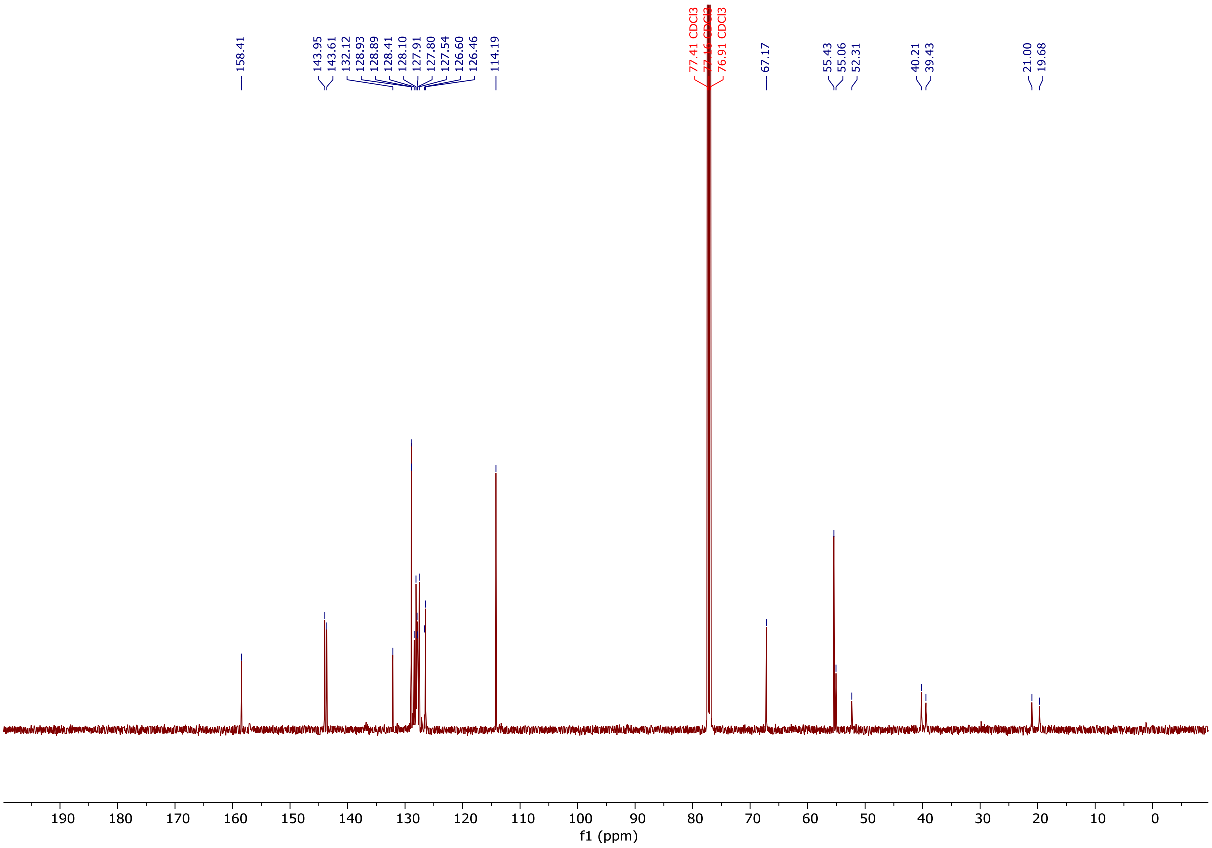

(5a): (±)-Methyl (2*S*,3*R*)-3-benzhydryl-2-(4-methoxyphenyl)piperidine-1-carboxylate

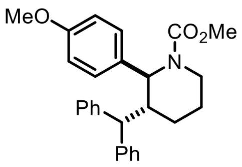

5a

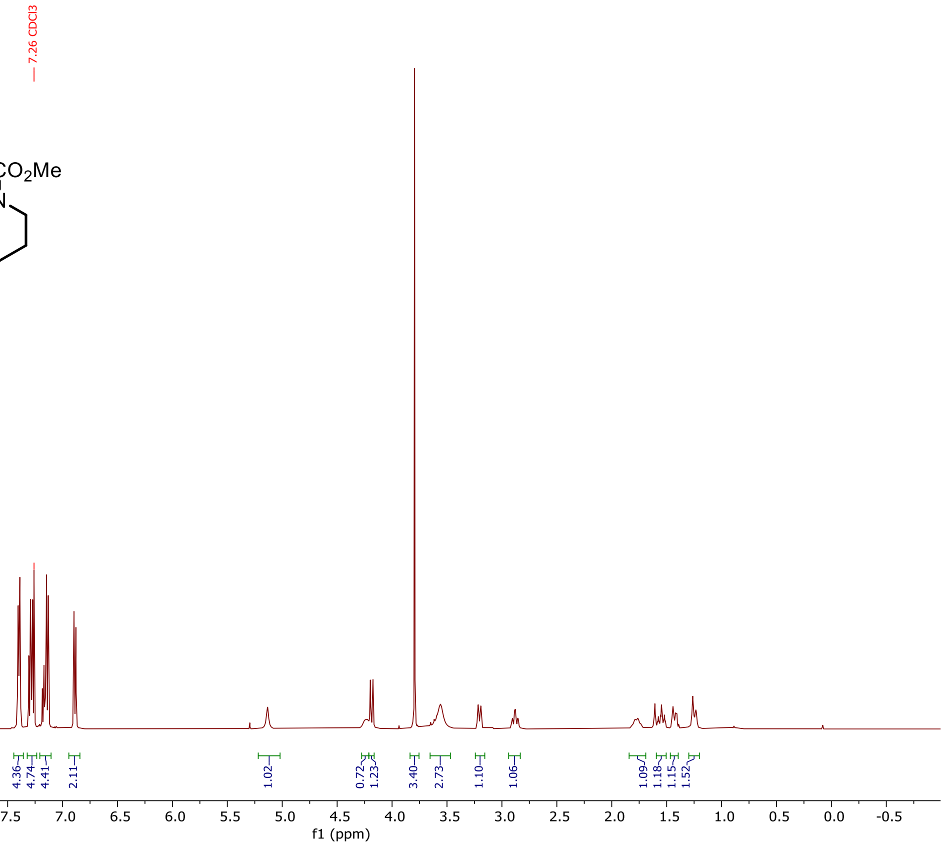

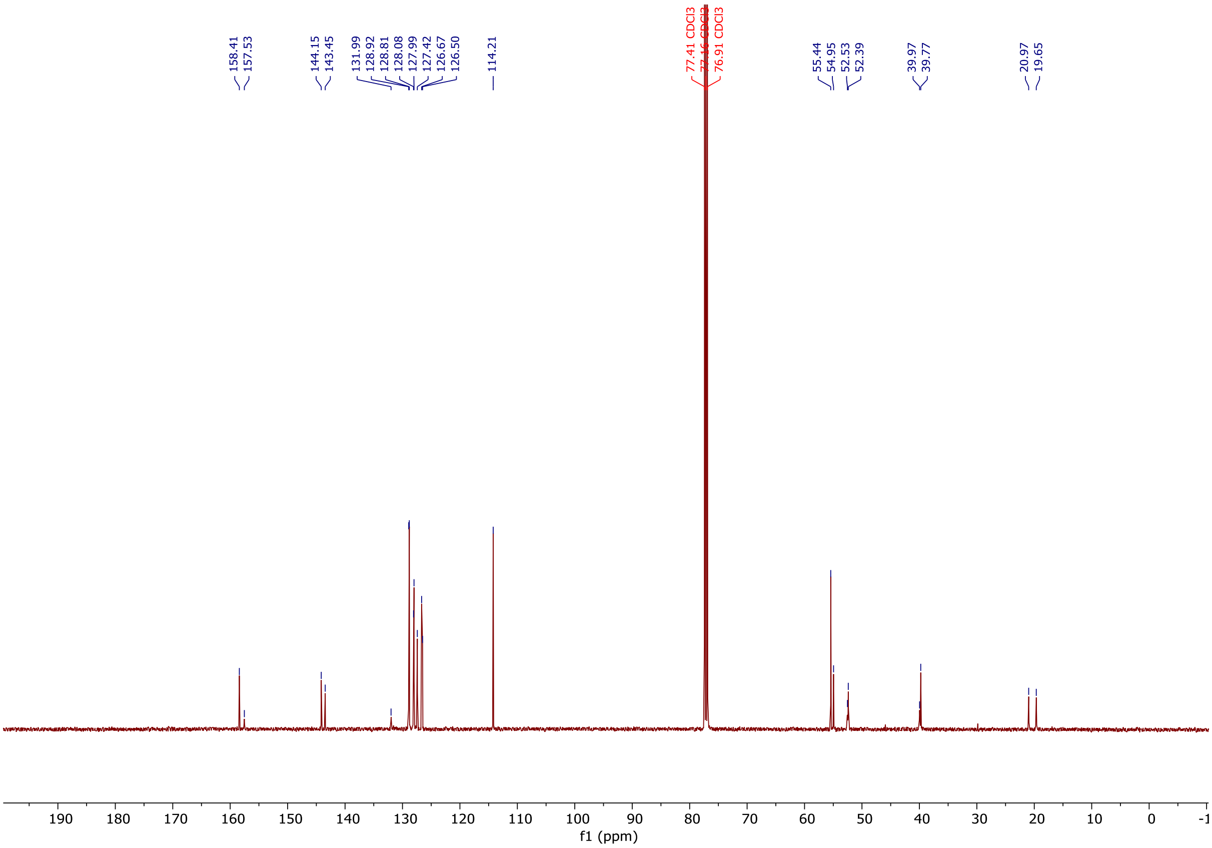

(5b): (±)-Methyl (2*S*,3*R*)-3-(4-methoxybenzyl)-2-(4-methoxyphenyl)piperidine-1-carboxylate and (±)-Methyl (2*S*,3*S*)-3-(4-methoxybenzyl)-2-(4-methoxyphenyl)piperidine-1-carboxylate

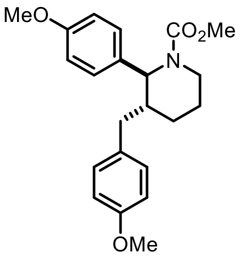

5b

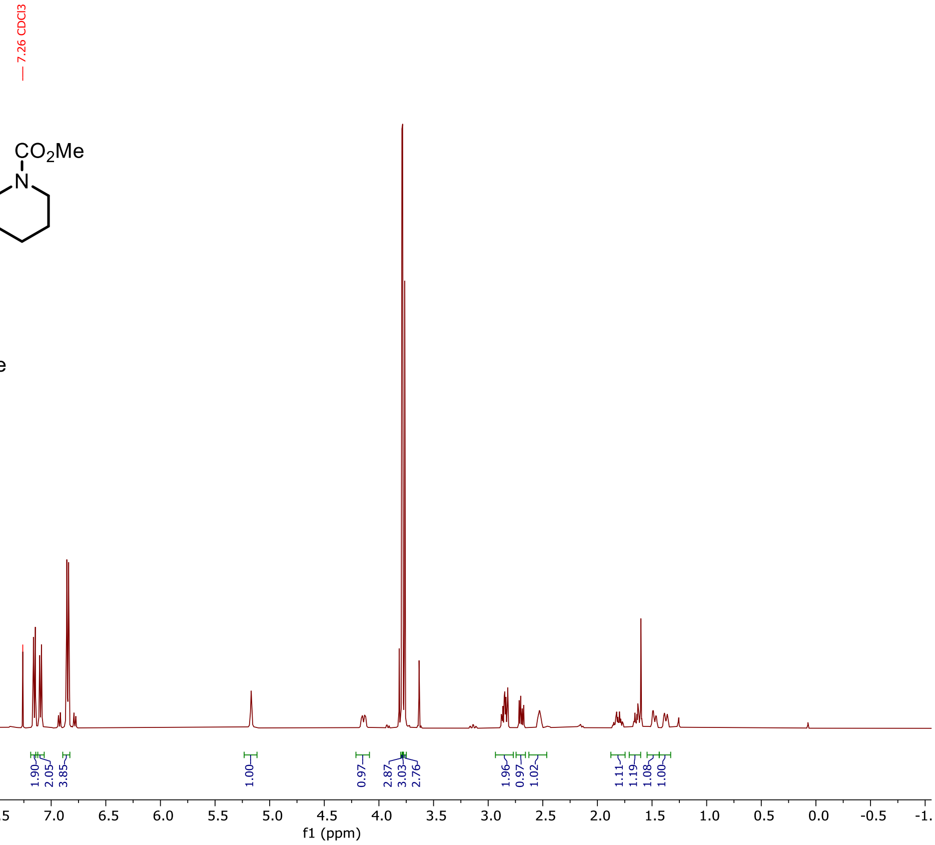

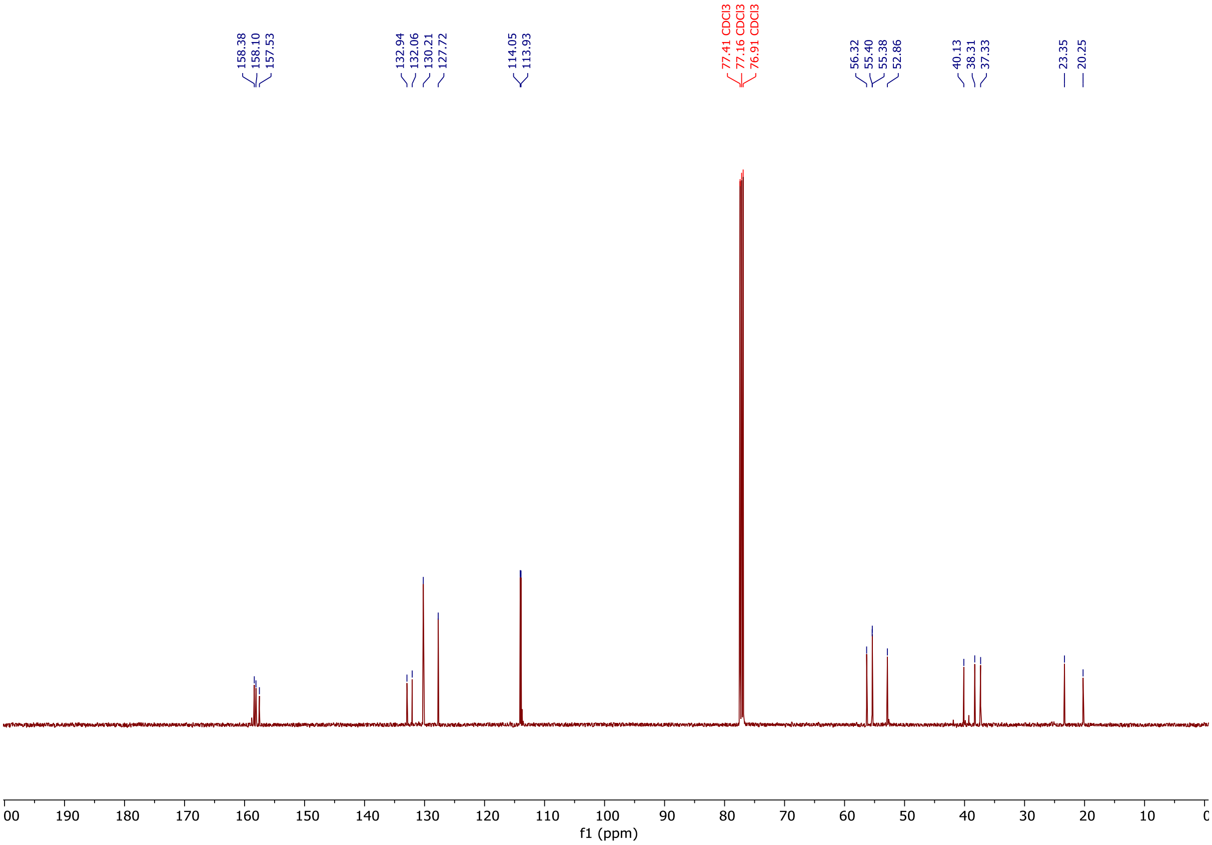

(5c): (±)-Methyl (2*S*,3*R*)-3-cinnamyl-2-(4-methoxyphenyl)piperidine-1-carboxylate

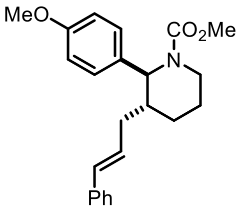

5c

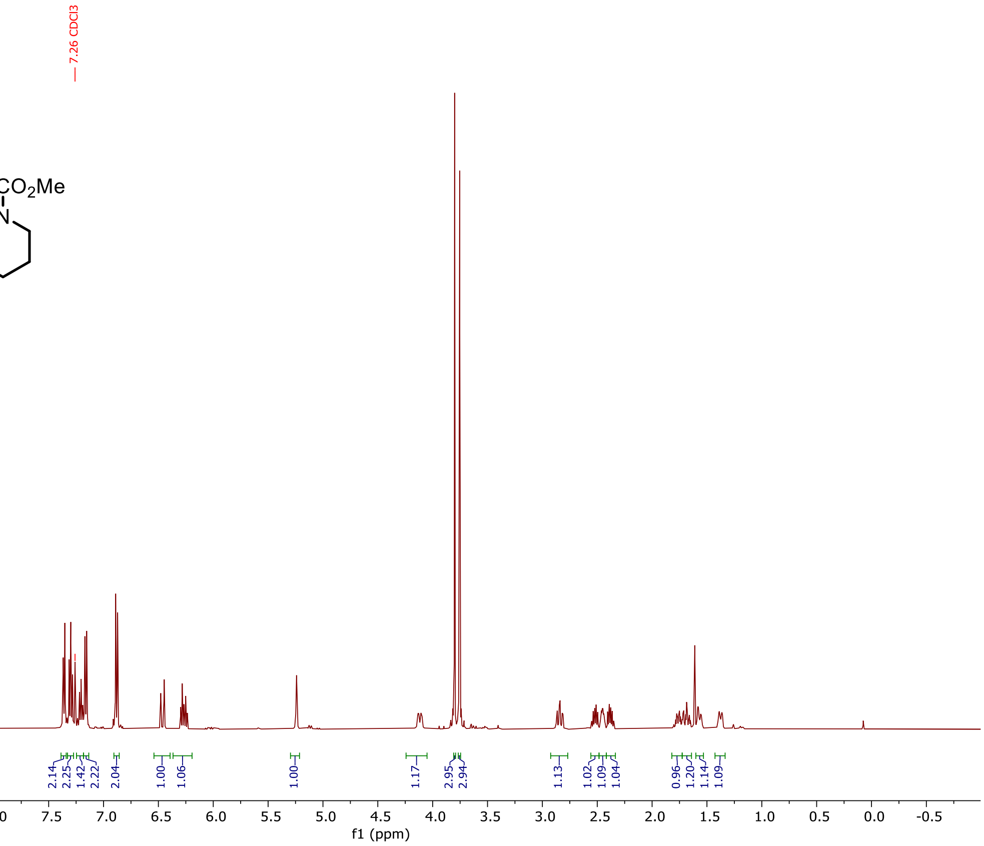

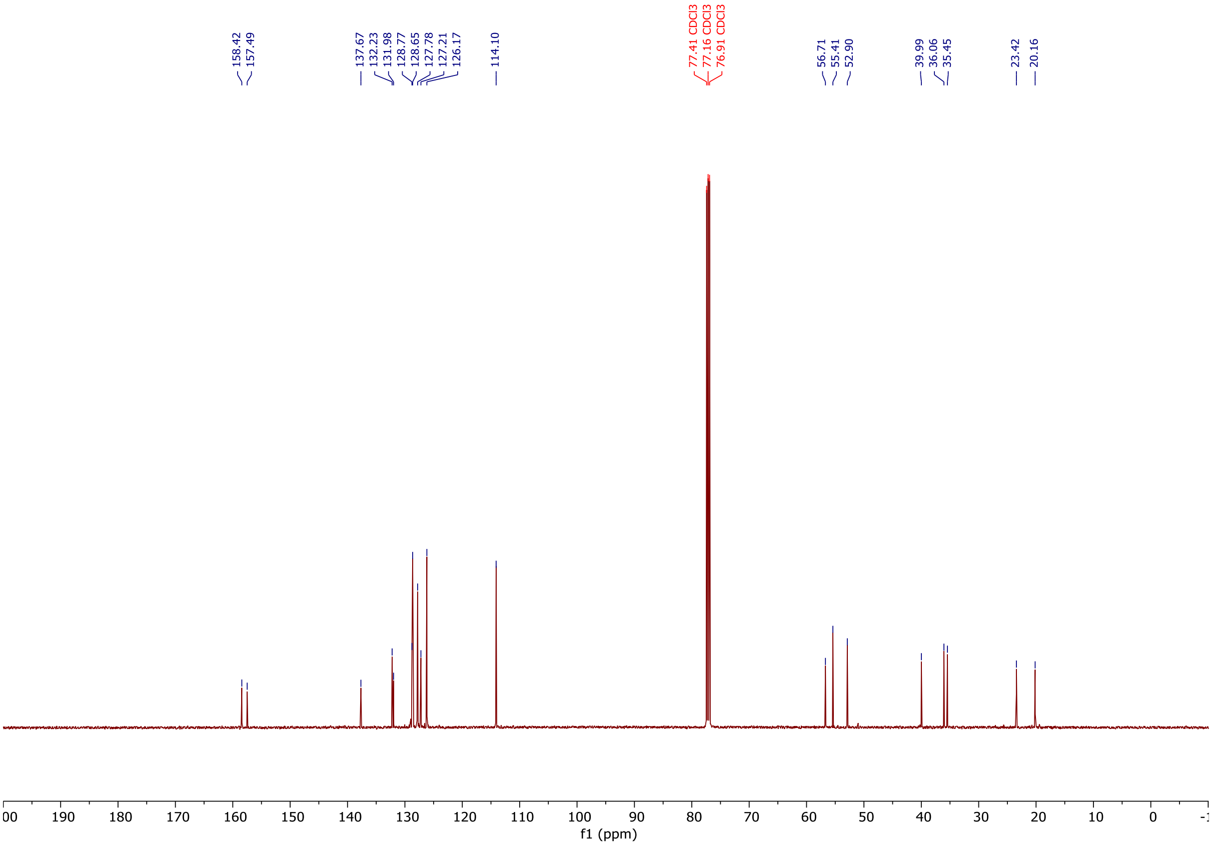

(5d): (±)-Methyl (2*S*,3*R*)-2-(4-methoxyphenyl)-3-((*E*)-3-(4-methoxyphenyl)allyl)piperidine-1-carboxylate

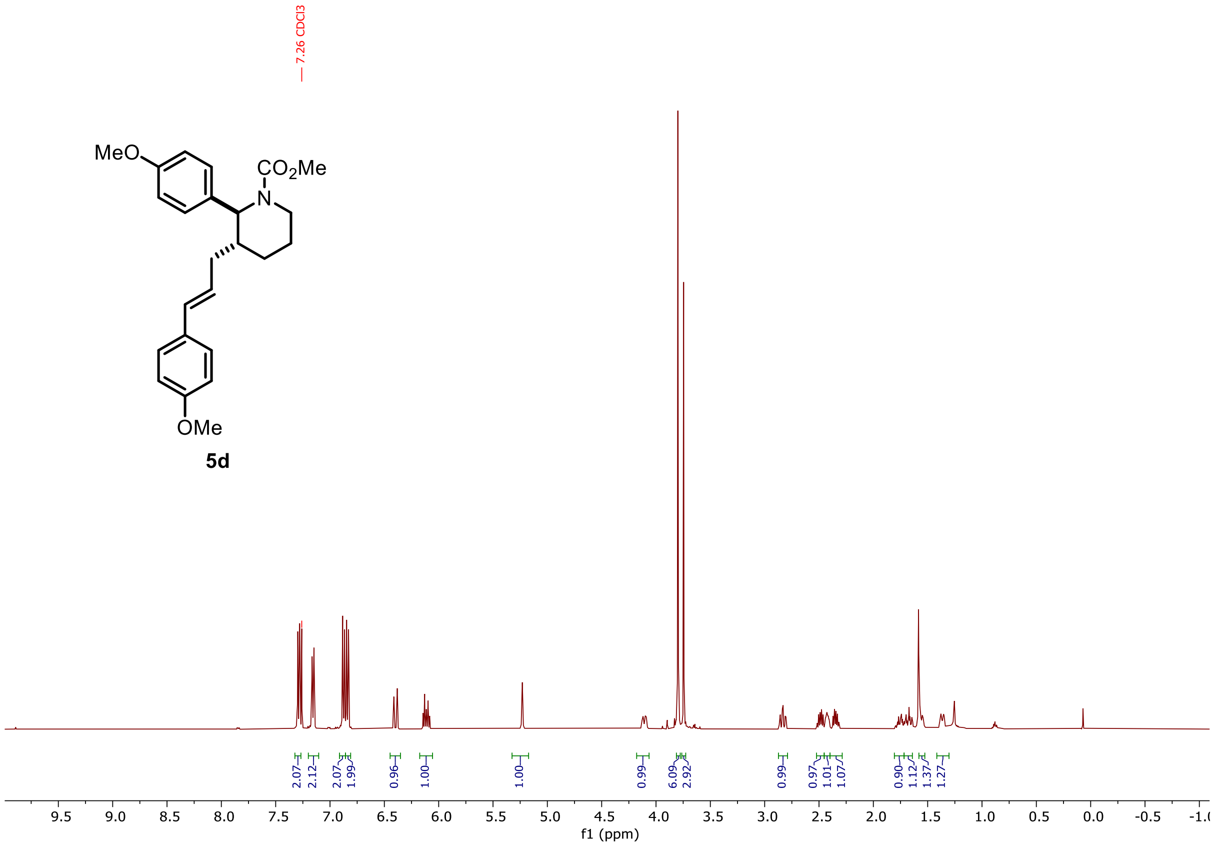

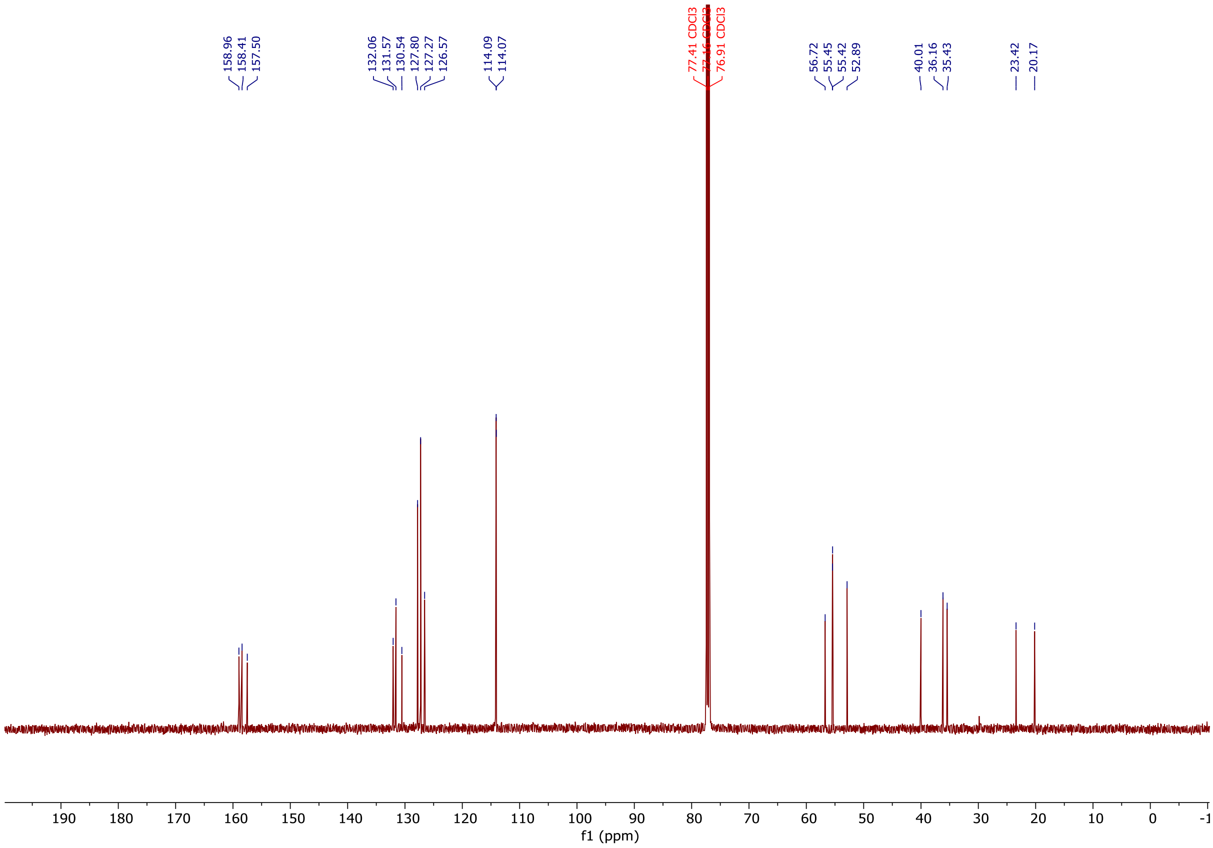

(5e): (±)-Methyl (2*S*,3*R*)-3-((*S*,*E*)-1,3-diphenylallyl)-2-(4-methoxyphenyl)piperidine-1-carboxylate and (±)-Methyl (2*S*,3*R*)-3-((*R*,*E*)-1,3-diphenylallyl)-2-(4-methoxyphenyl)-piperidine-1-carboxylate

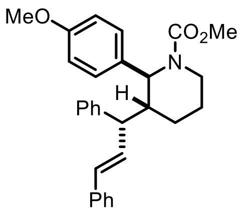

5e

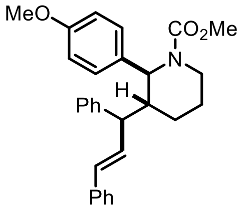

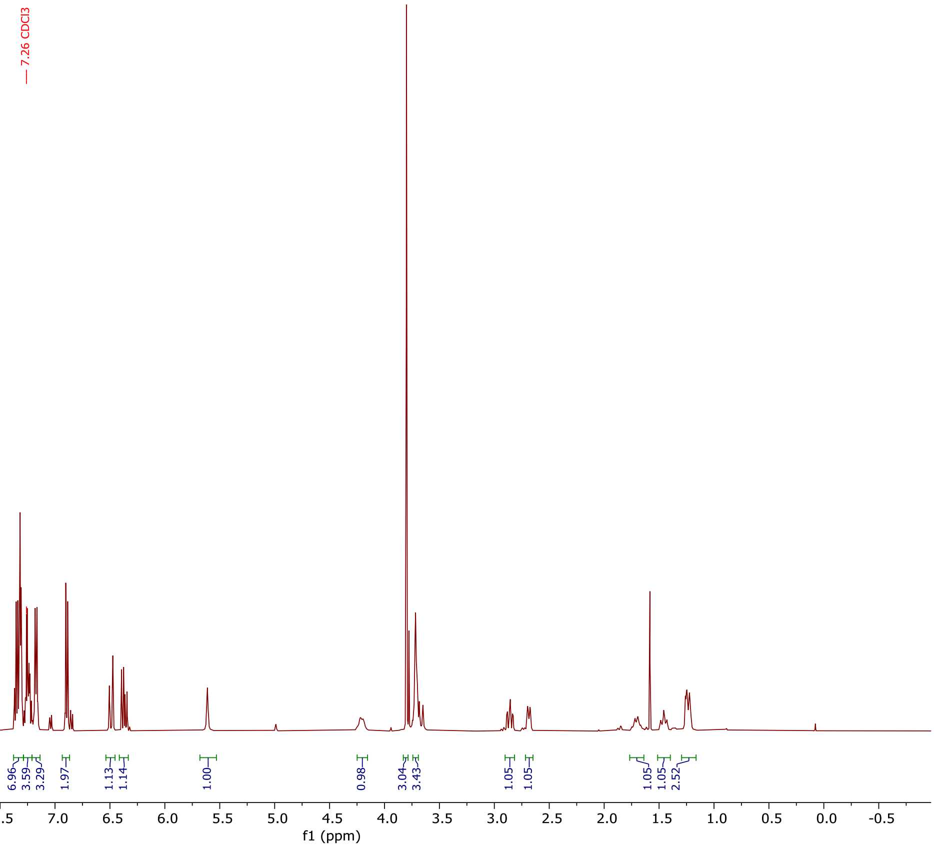

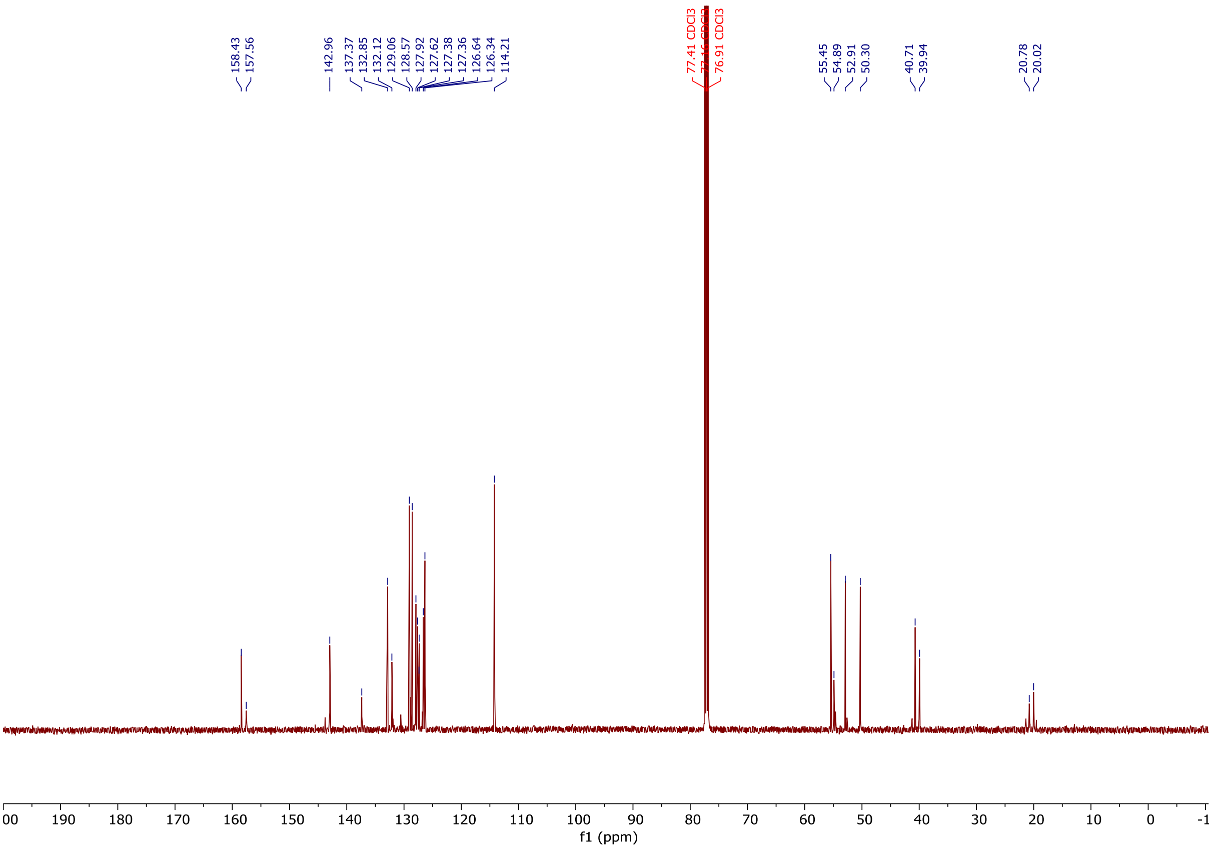

(5f): (±)-Methyl (2*S*,3*R*)-2-(4-methoxyphenyl)-3-((*S*,*E*)-pent-3-en-2-yl)piperidine-1-carboxylate and (±)-Methyl (2*S*,3*R*)-2-(4-methoxyphenyl)-3-((*R*,*E*)-pent-3-en-2-yl)piperidine-1-carboxylate

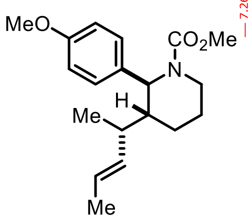

5f

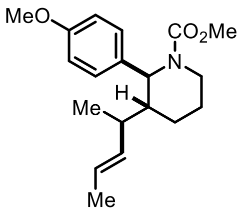

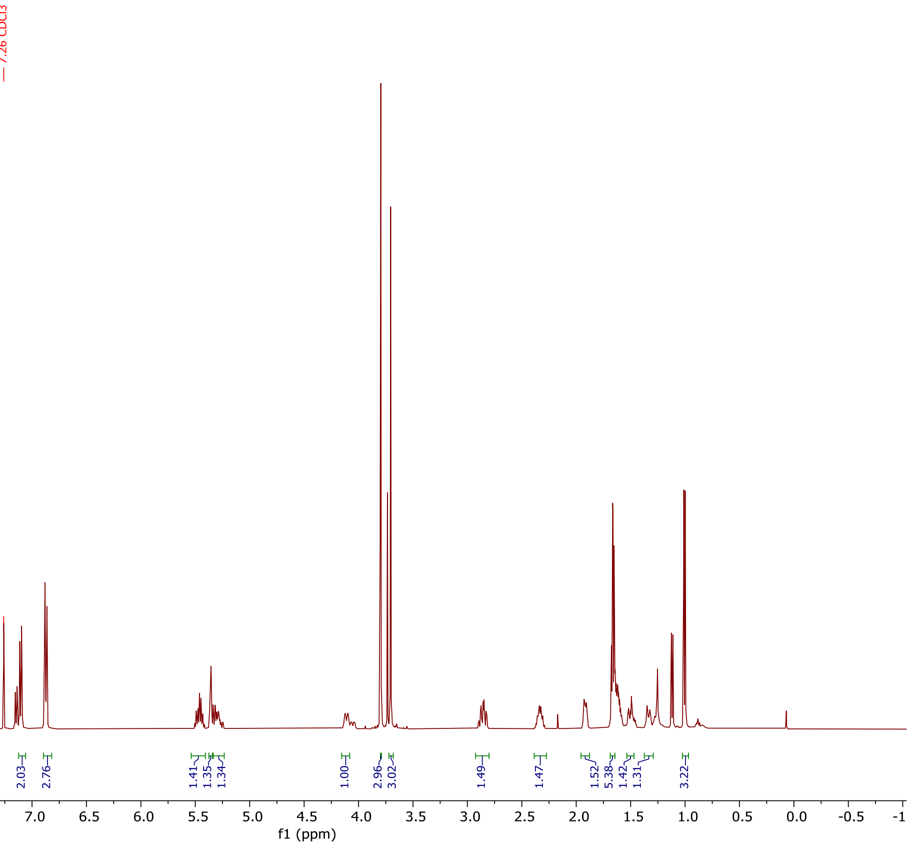

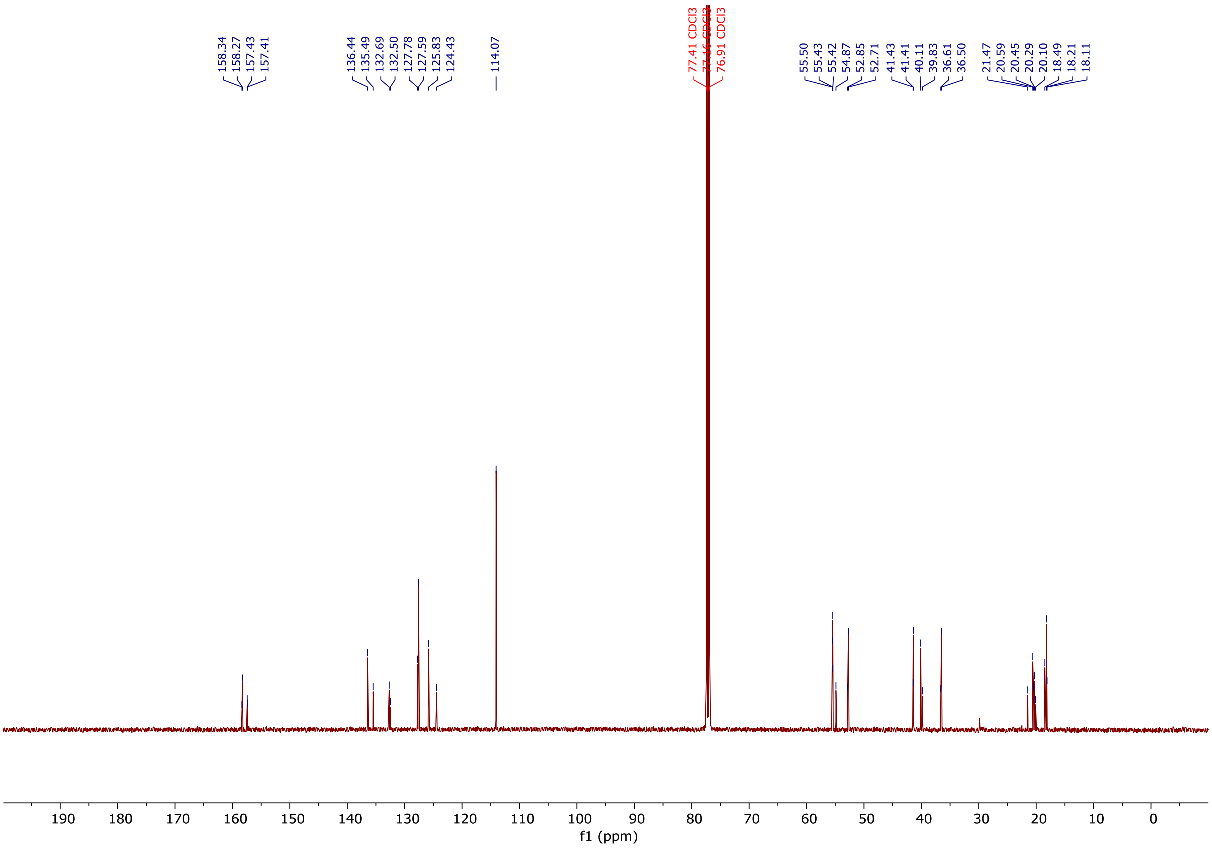

(5g): (±)-Methyl (2*S*,3*R*)-3-((*R*)-cyclopent-2-en-1-yl)-2-(4-methoxyphenyl)piperidine-1-carboxylate and (±)-Methyl (2*S*,3*R*)-3-((*S*)-cyclopent-2-en-1-yl)-2-(4-methoxyphenyl)-piperidine-1-carboxylate

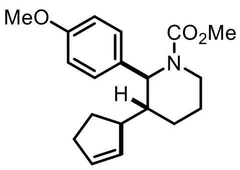

5g

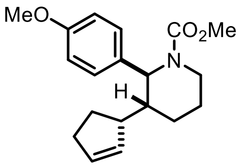

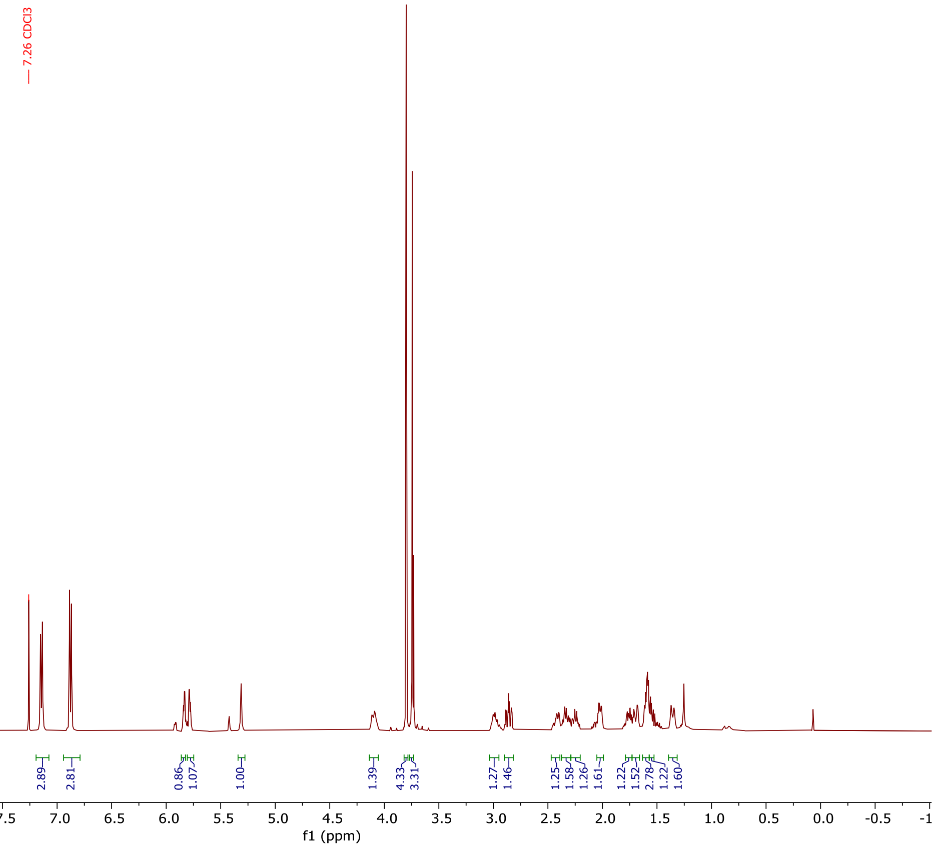

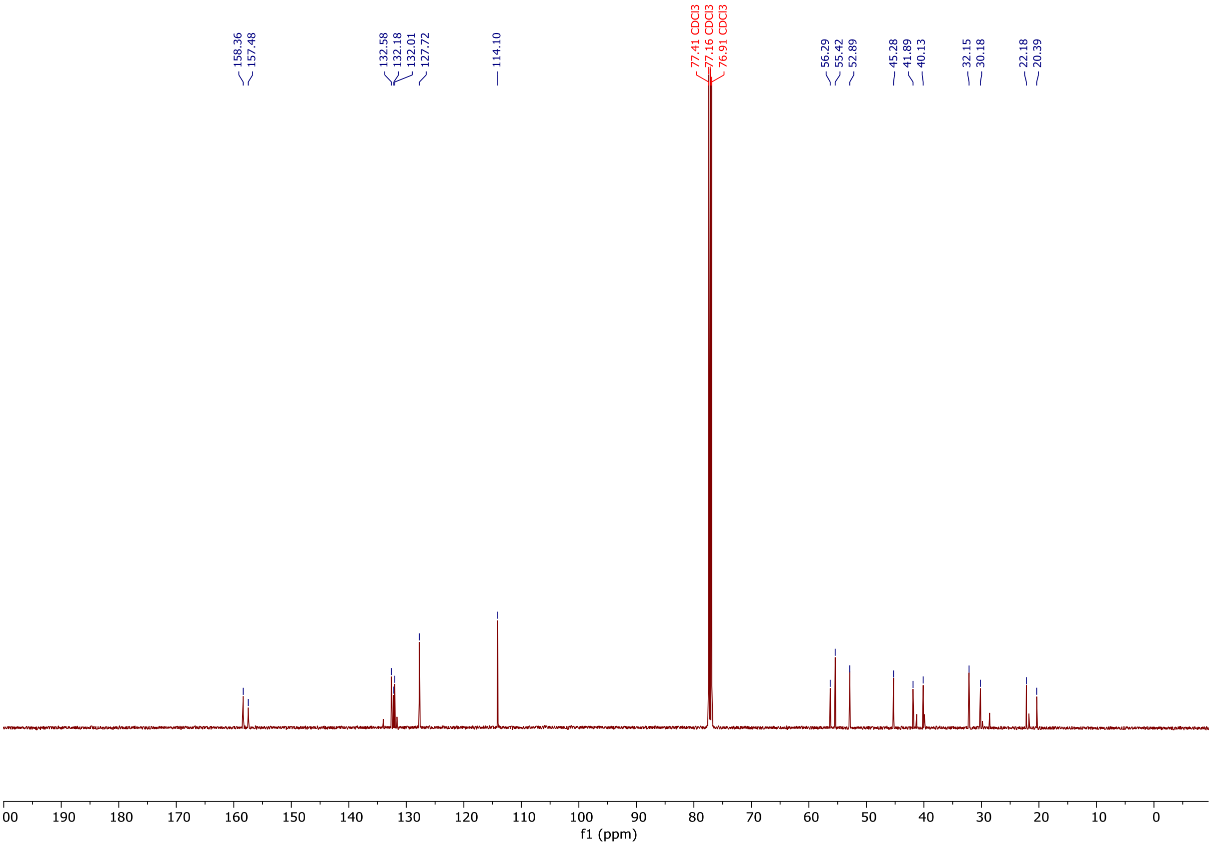

(5h): (±)-Methyl (2*S*,3*R*)-2-(4-methoxyphenyl)-3-((*R*)-3-phenylcyclopent-2-en-1-yl)piperidine-1-carboxylate and (±)-Methyl (2*S*,3*R*)-2-(4-methoxyphenyl)-3-((*S*)-3-phenylcyclopent-2-en-1-yl)-piperidine-1-carboxylate

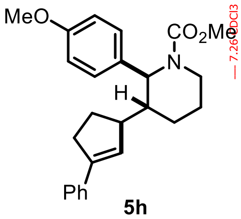

5h

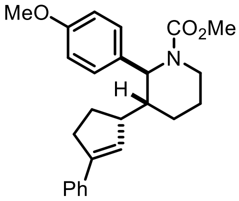

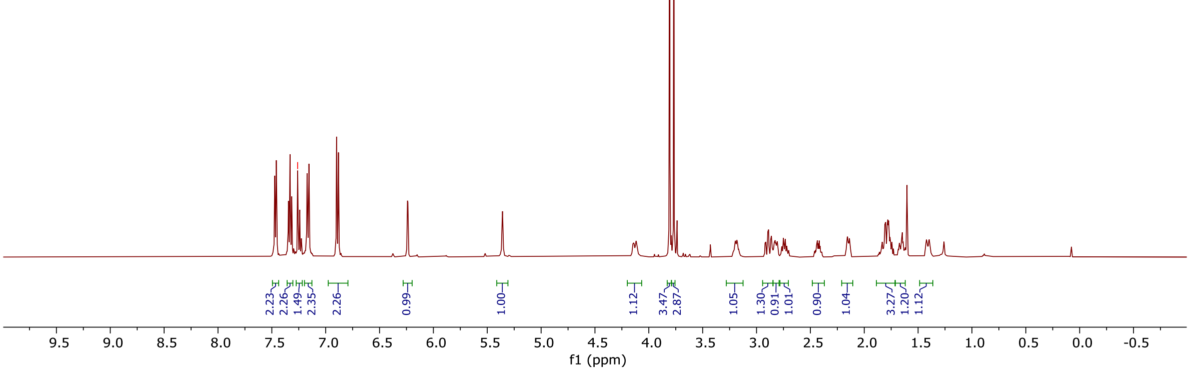

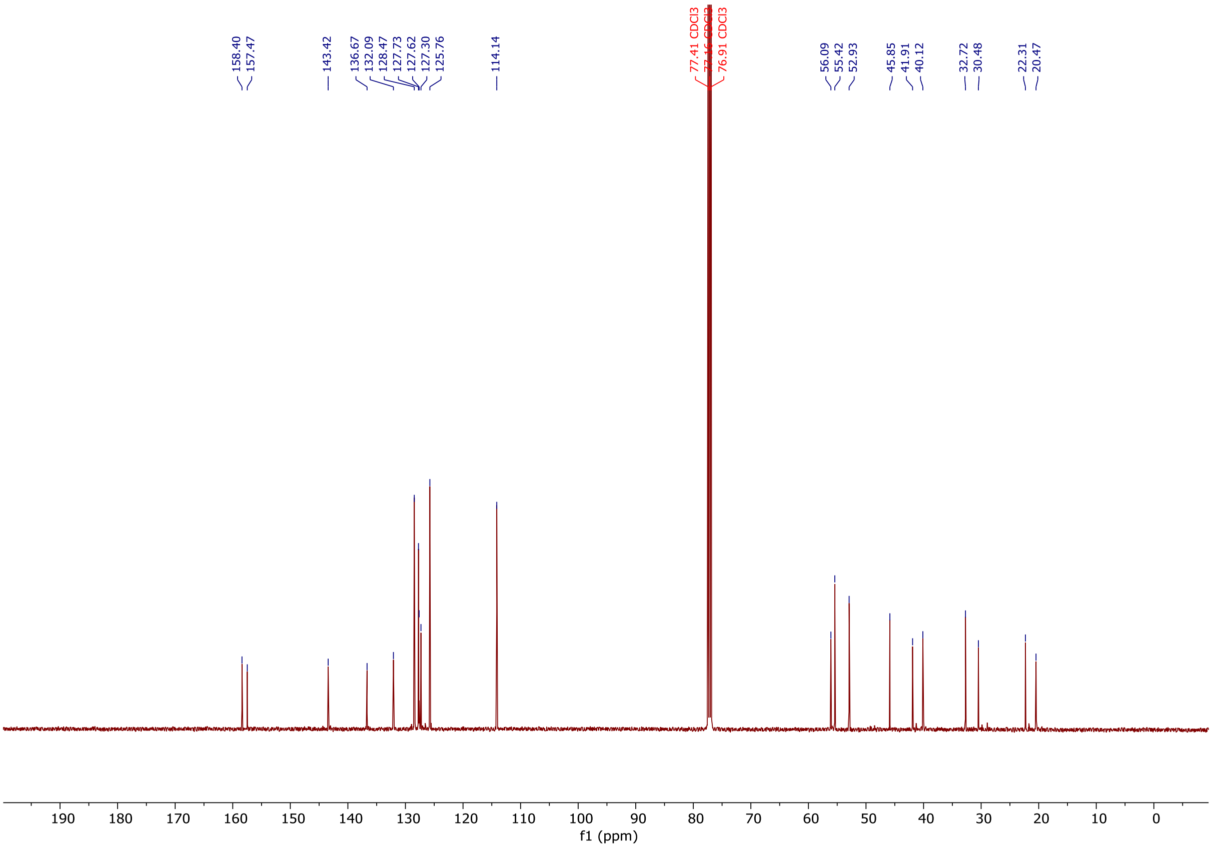

(5i): (±)-Methyl (2*S*,3*R*)-3-benzhydryl-2-phenylpiperidine-1-carboxylate

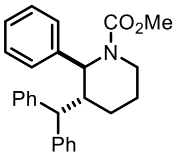

5i

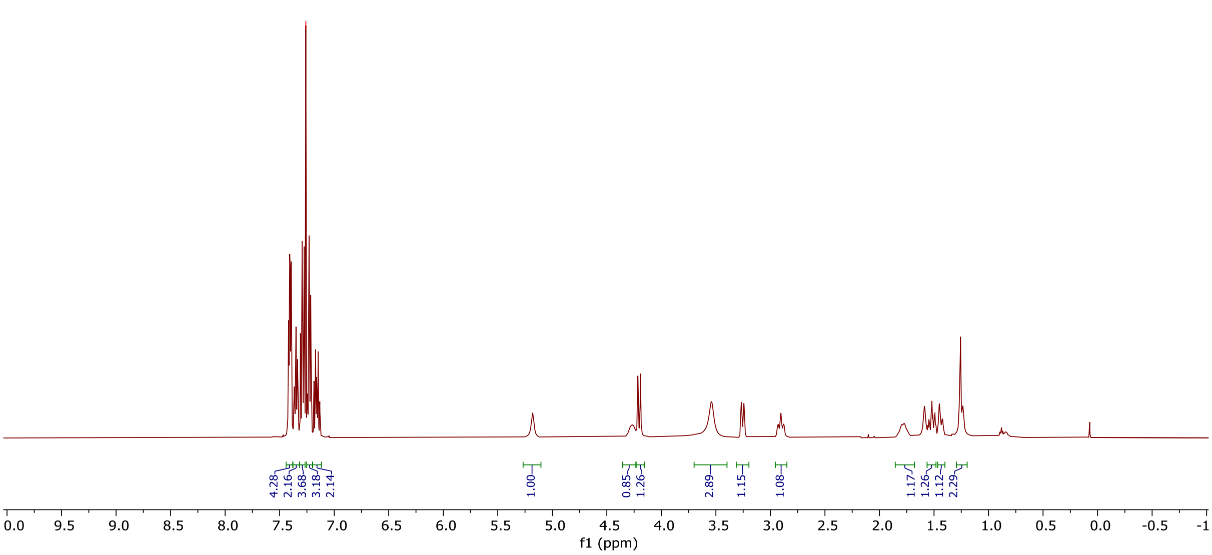

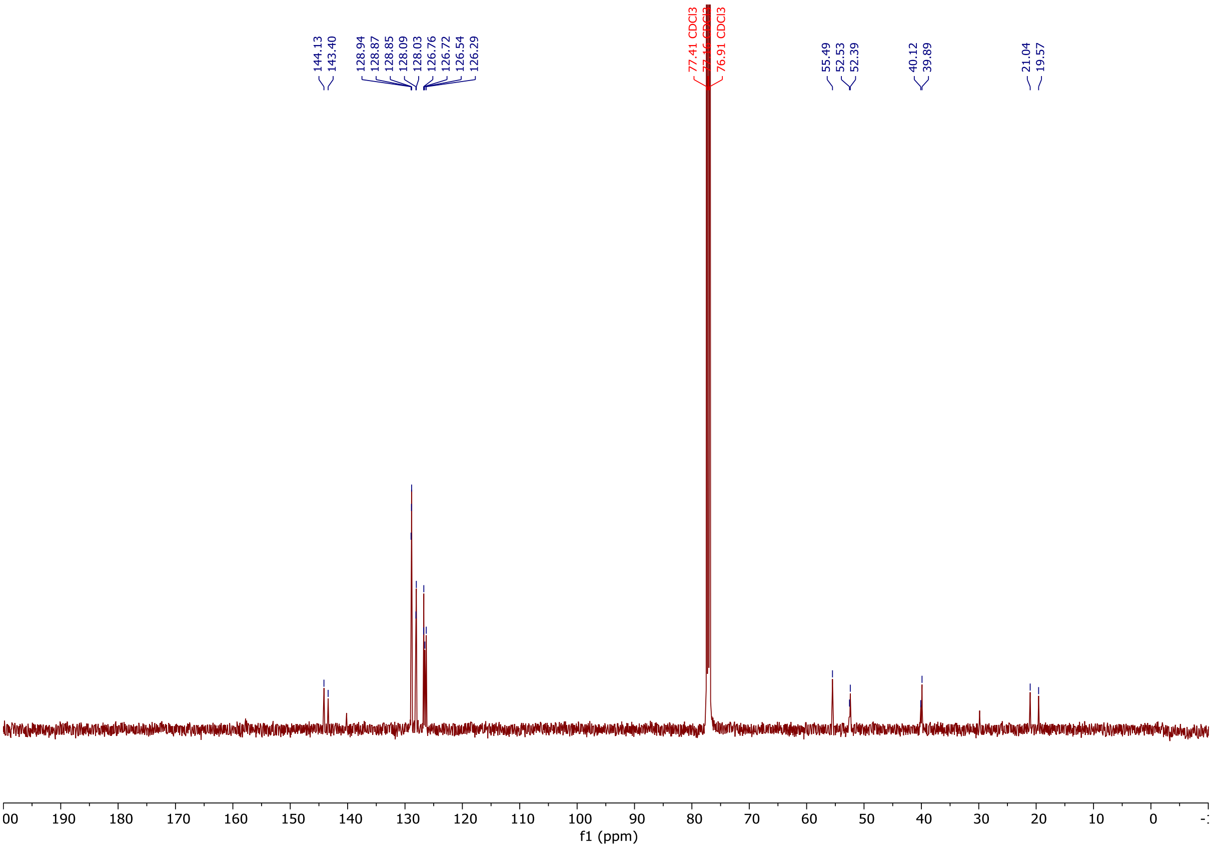

(5i-Ns): (±)-(2*S*,3*R*)-3-Benzhydryl-1-((2-nitrophenyl)sulfonyl)-2-phenylpiperidine

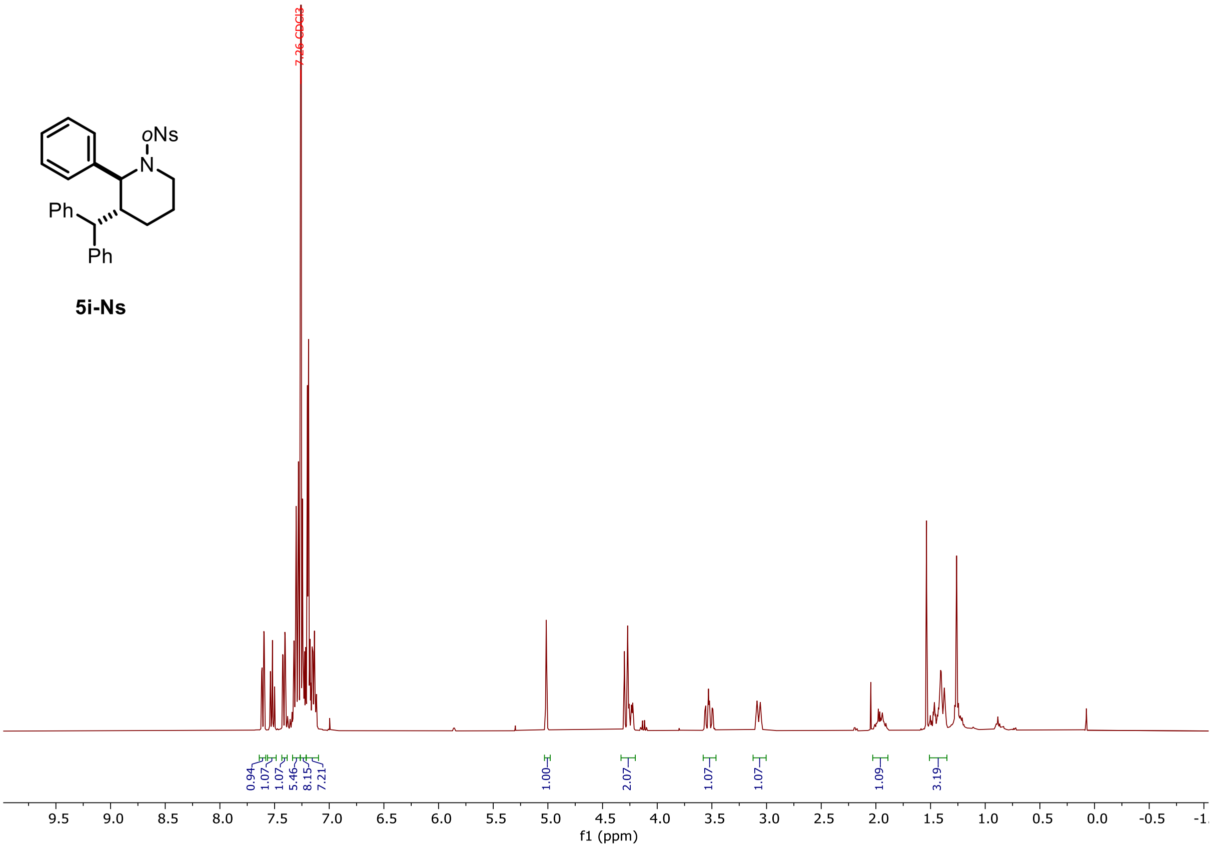

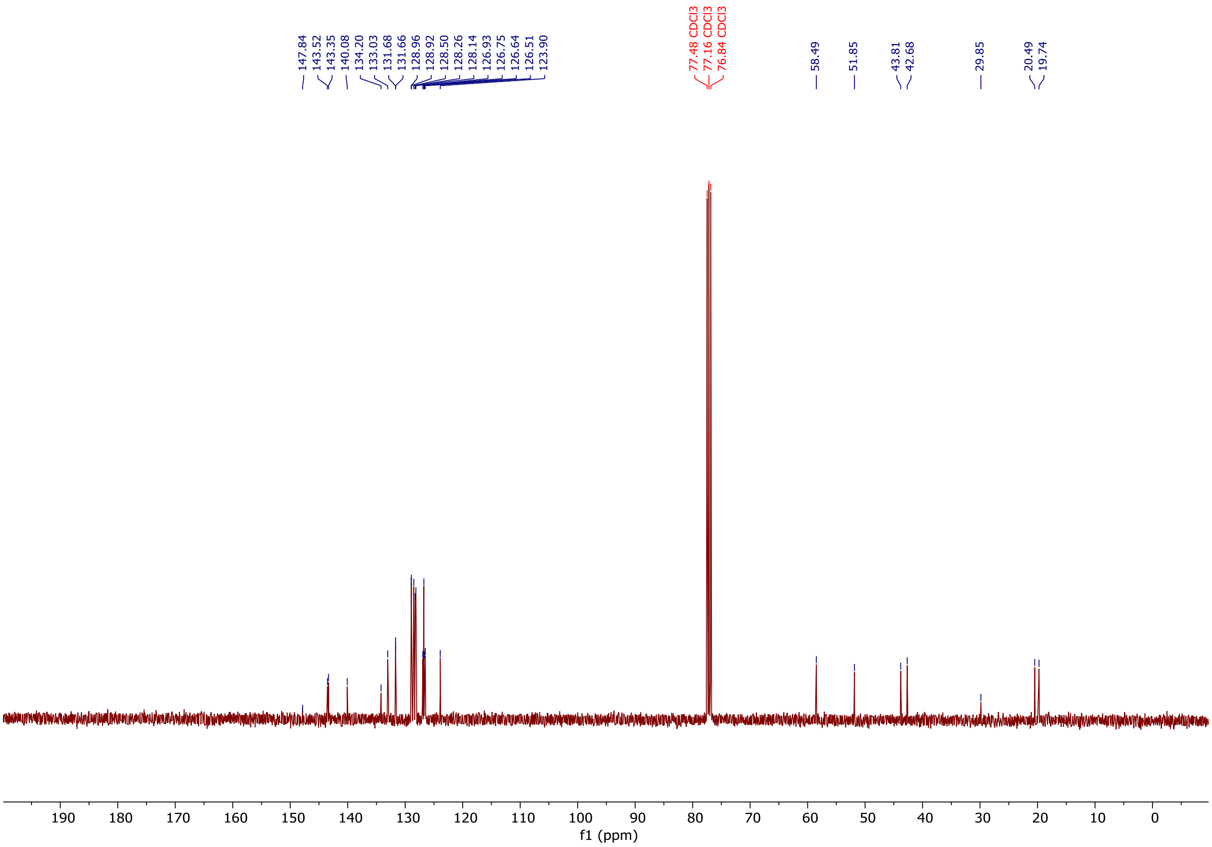

(5j): (±)-Methyl (2*S*,3*R*)-3-benzhydryl-2-(4-bromophenyl)piperidine-1-carboxylate

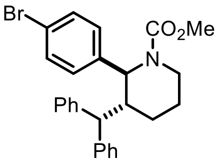

5j

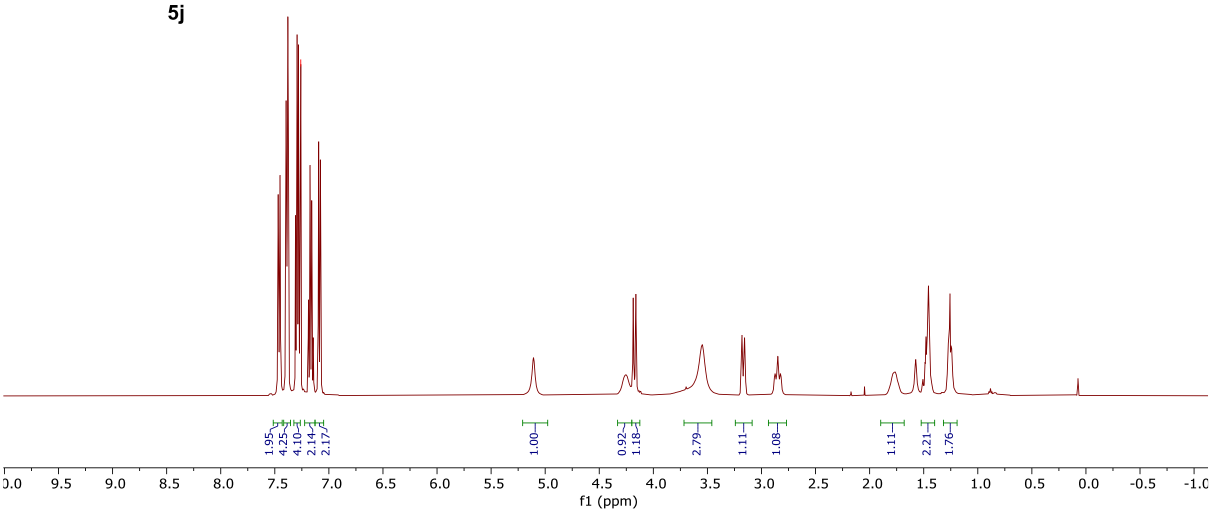

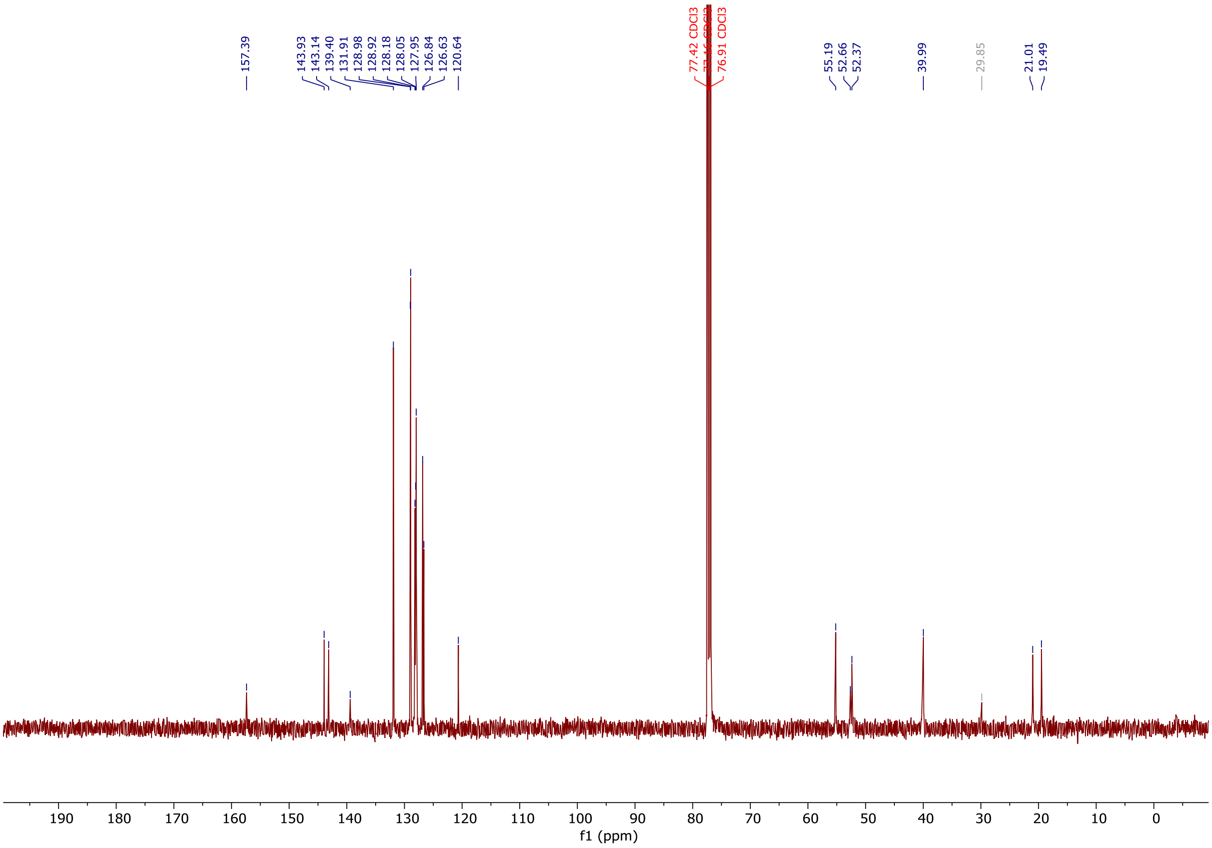

(5k): (±)-Methyl (2*S*,3*R*)-3-(4-methoxybenzyl)-2-phenylpiperidine-1-carboxylate

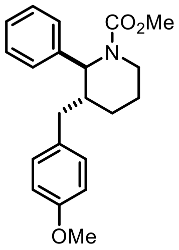

5k

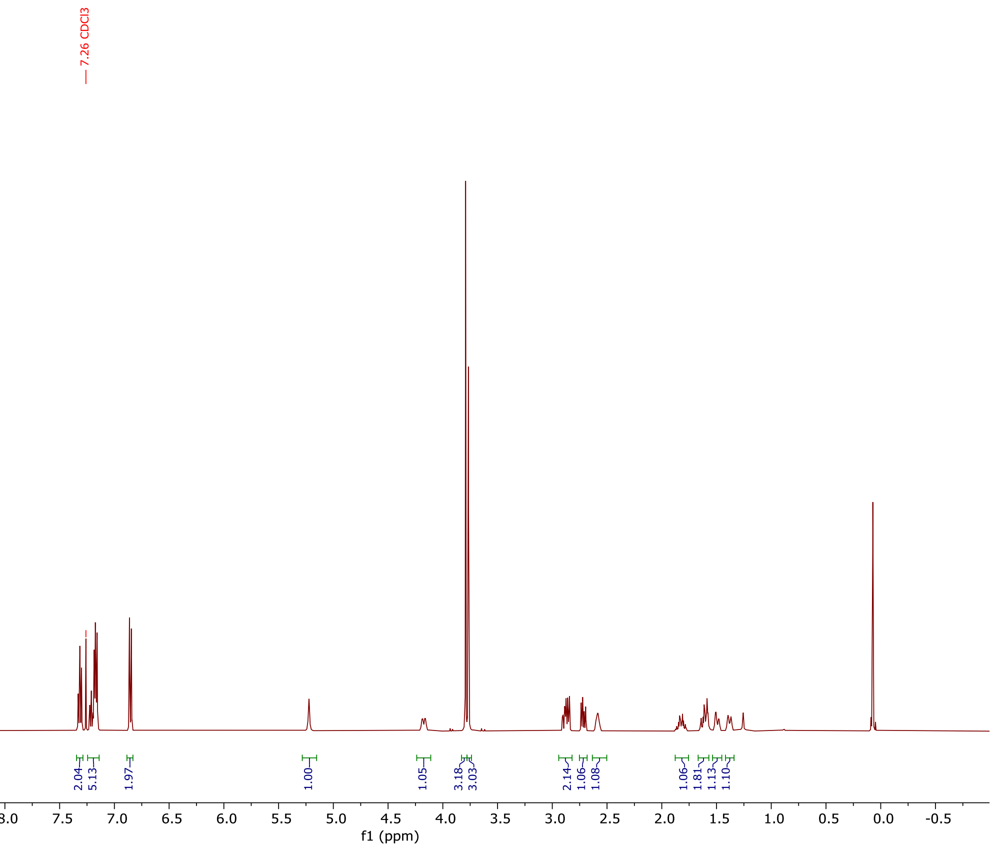

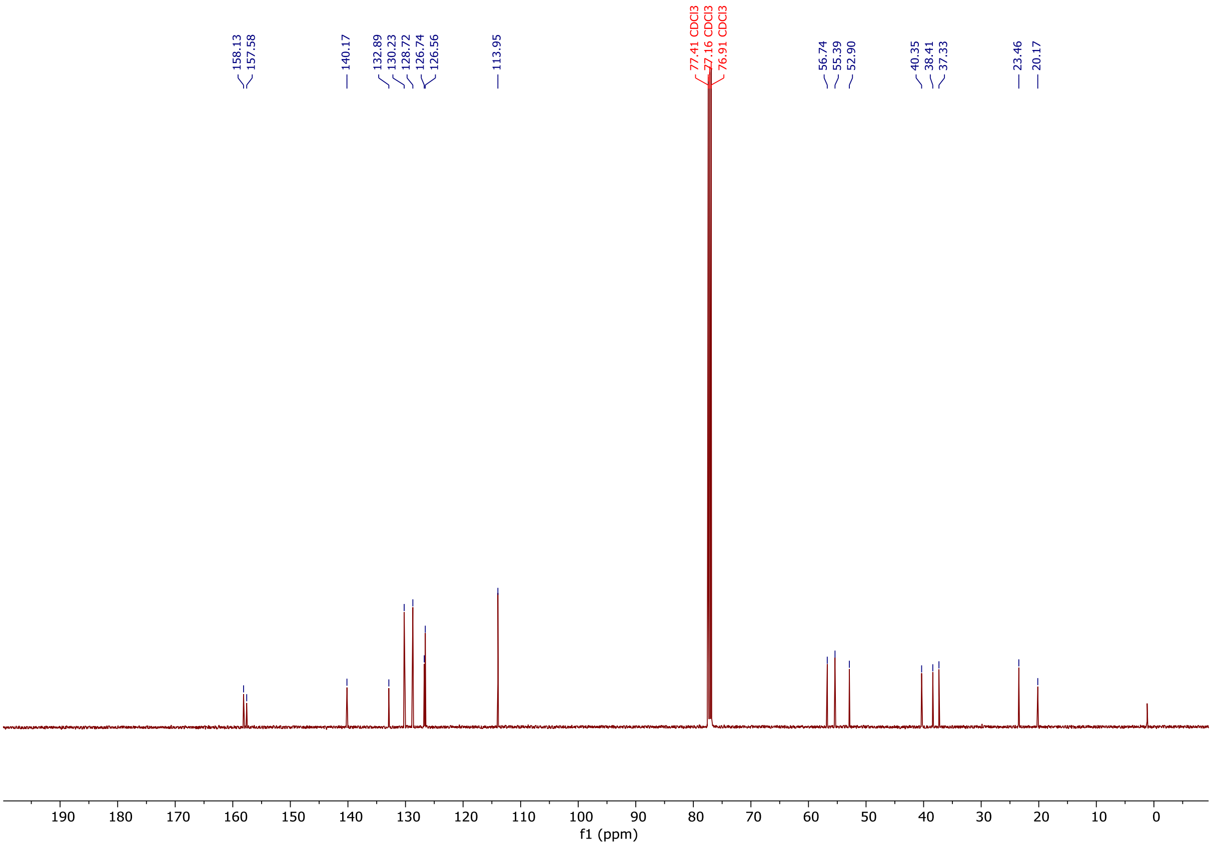

(5l): (±)-Methyl (2*S*,3*R*)-2-(4-bromophenyl)-3-(4-methoxybenzyl)piperidine-1-carboxylate

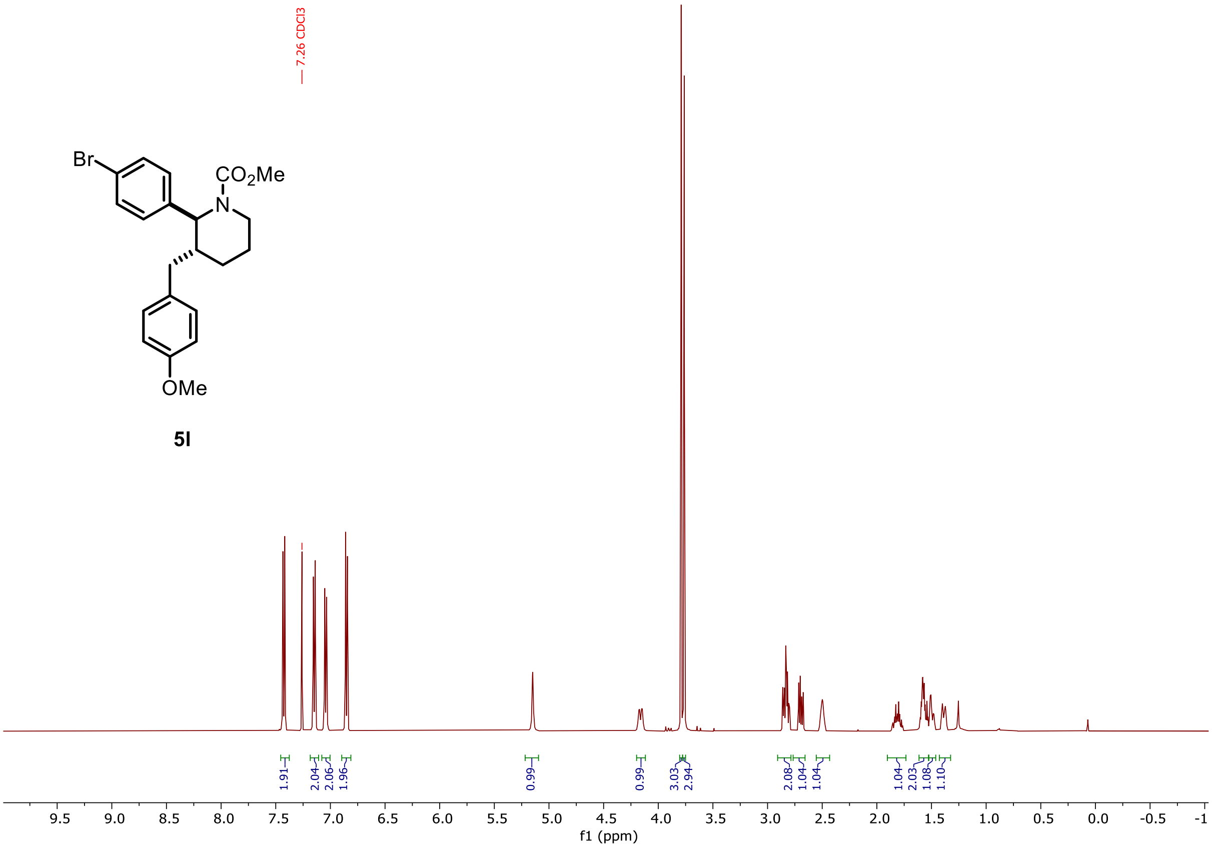

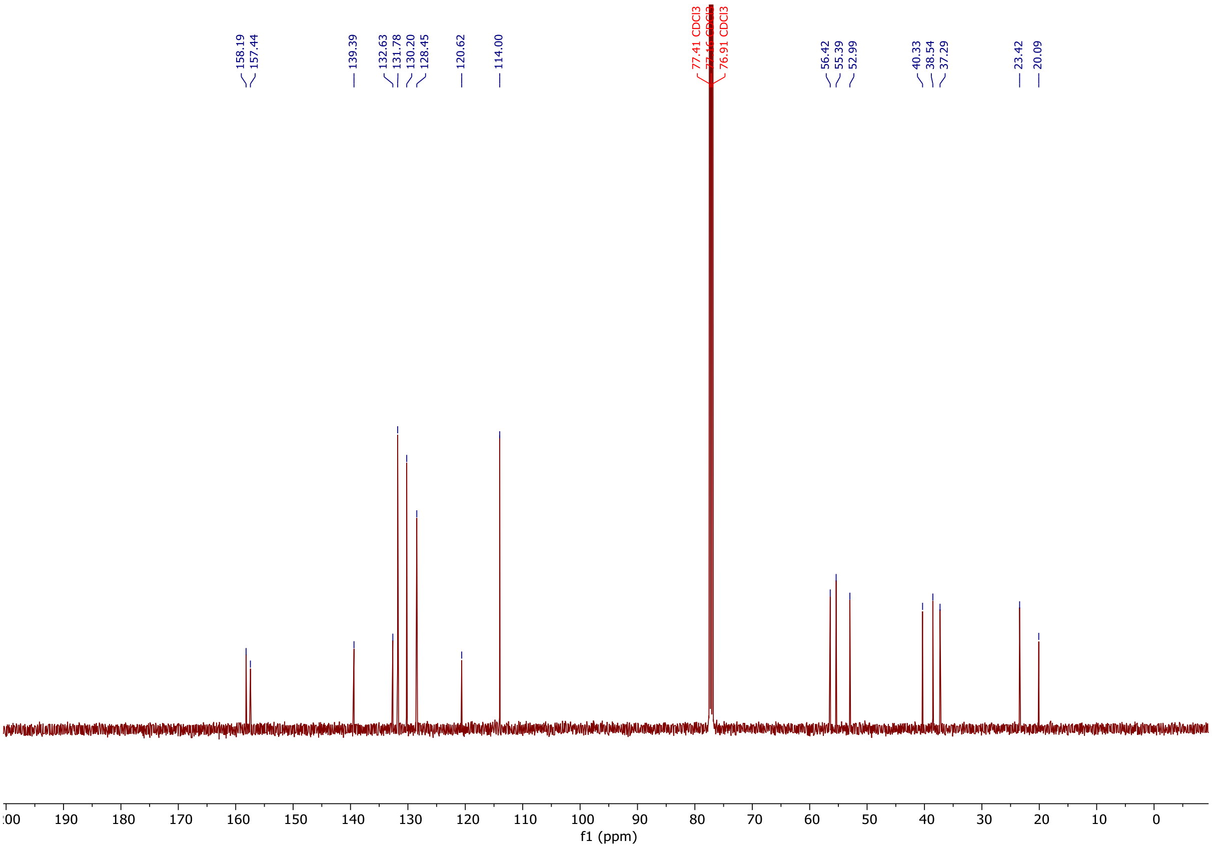

(5m): (±)-Methyl (2*R*,3*R*)-3-benzhydryl-2-((*E*)-4-methoxystyryl)piperidine-1-carboxylate and (±)-methyl (2*R*,3*S*)-3-benzhydryl-2-((*E*)-4-methoxystyryl)piperidine-1-carboxylate

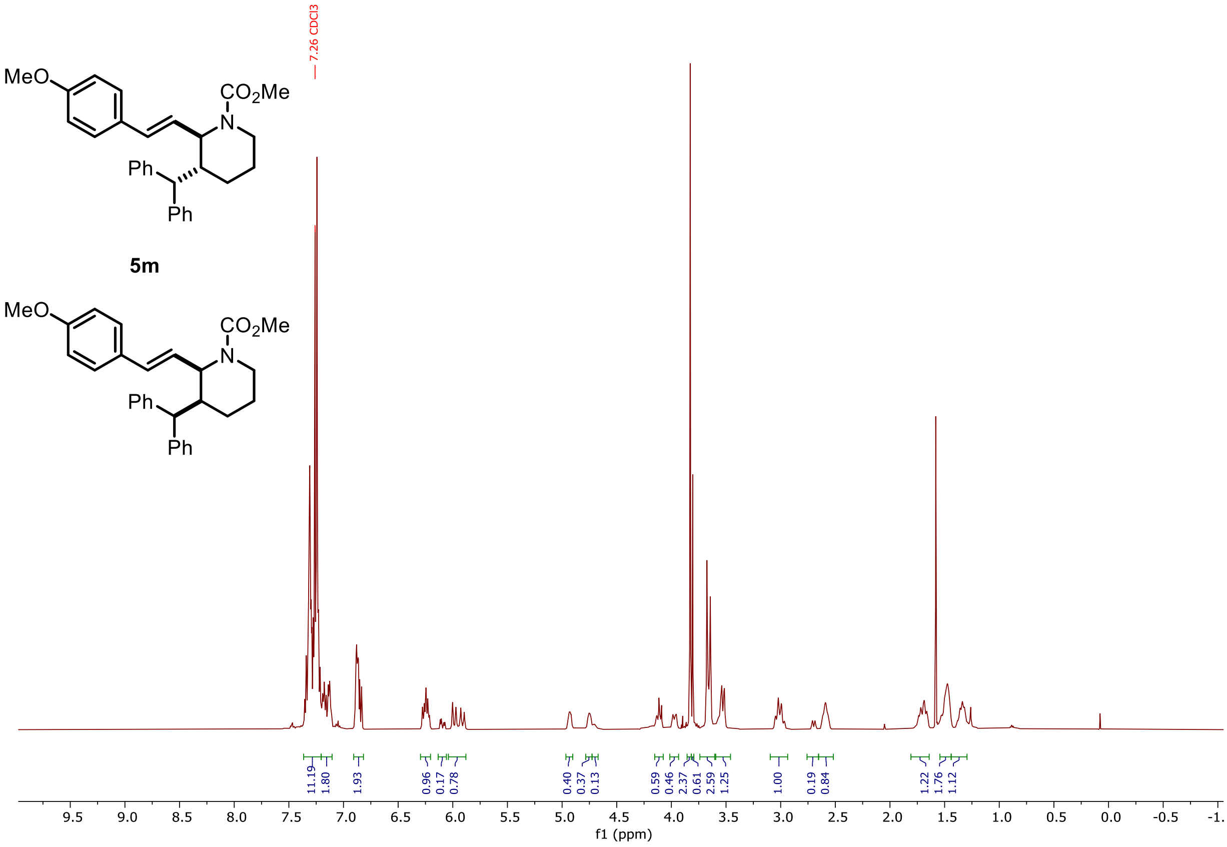

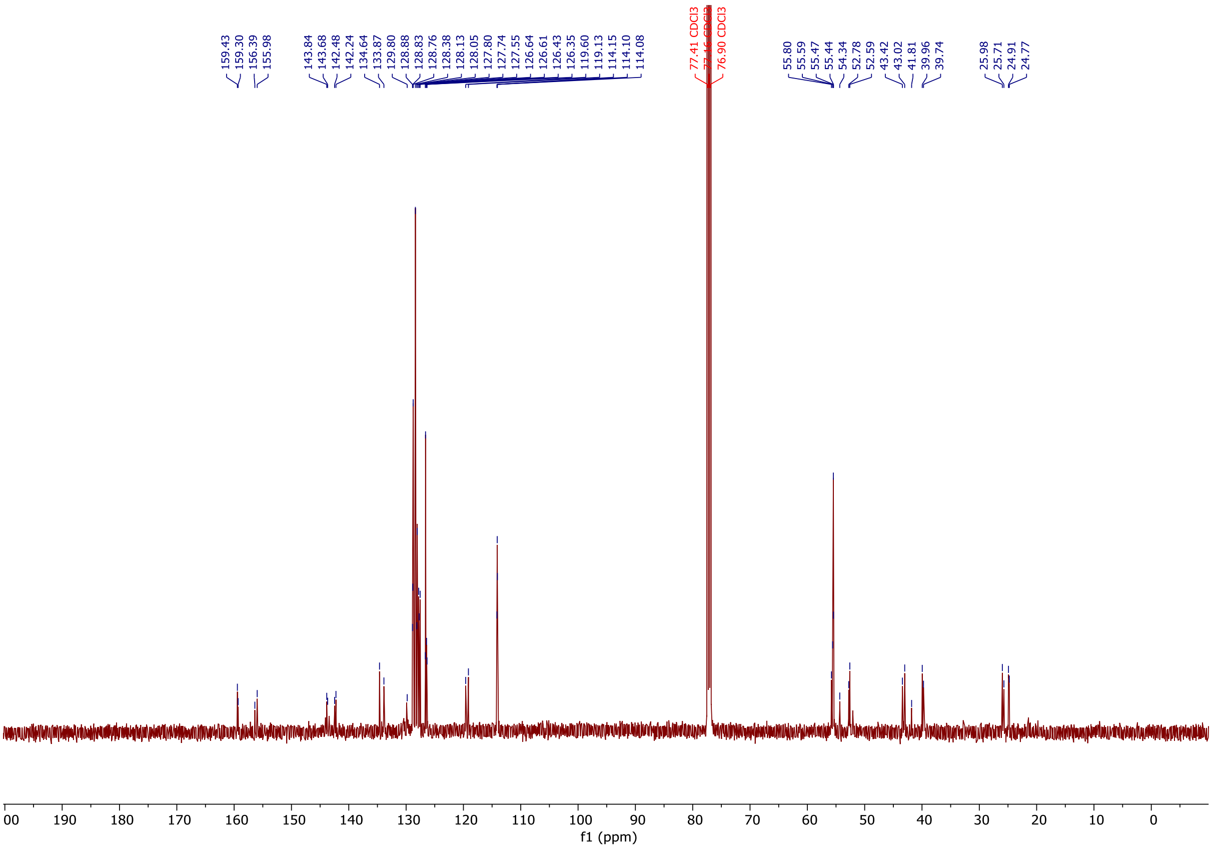

(5n-Ns): 2-(4-Methoxyphenyl)-1-((2-nitrophenyl)sulfonyl)piperidin-3-yl)methanol

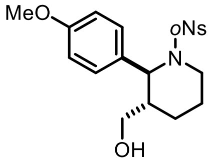

5n-Ns

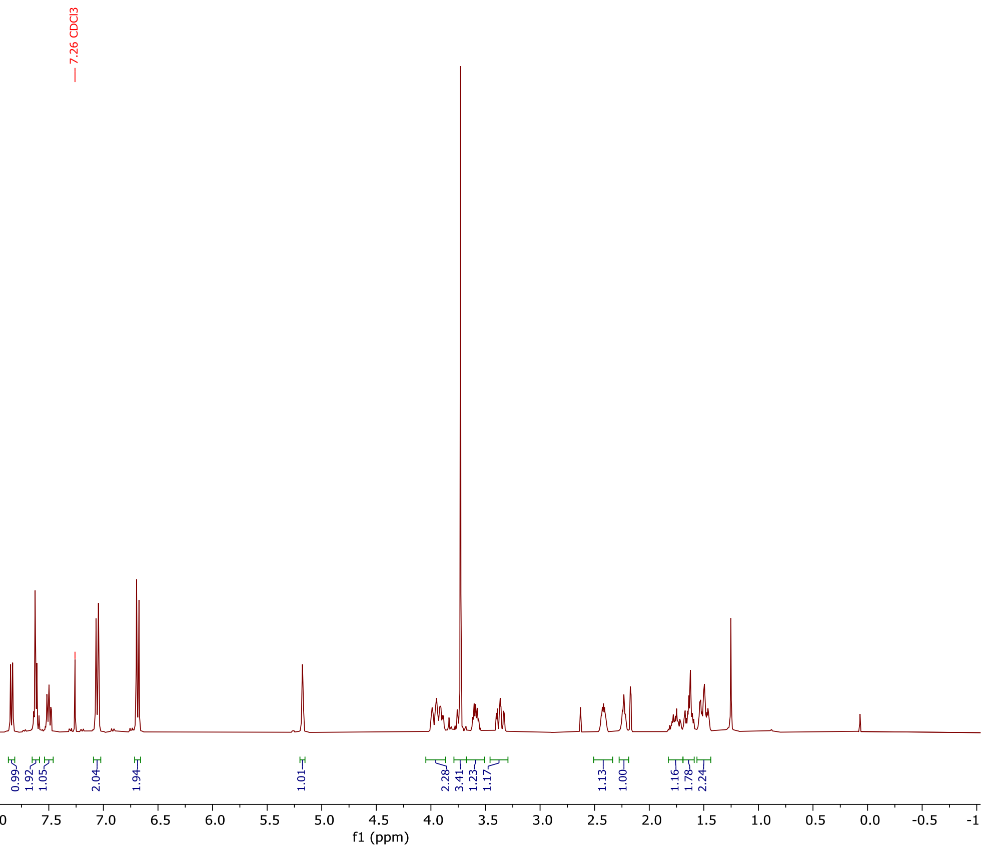

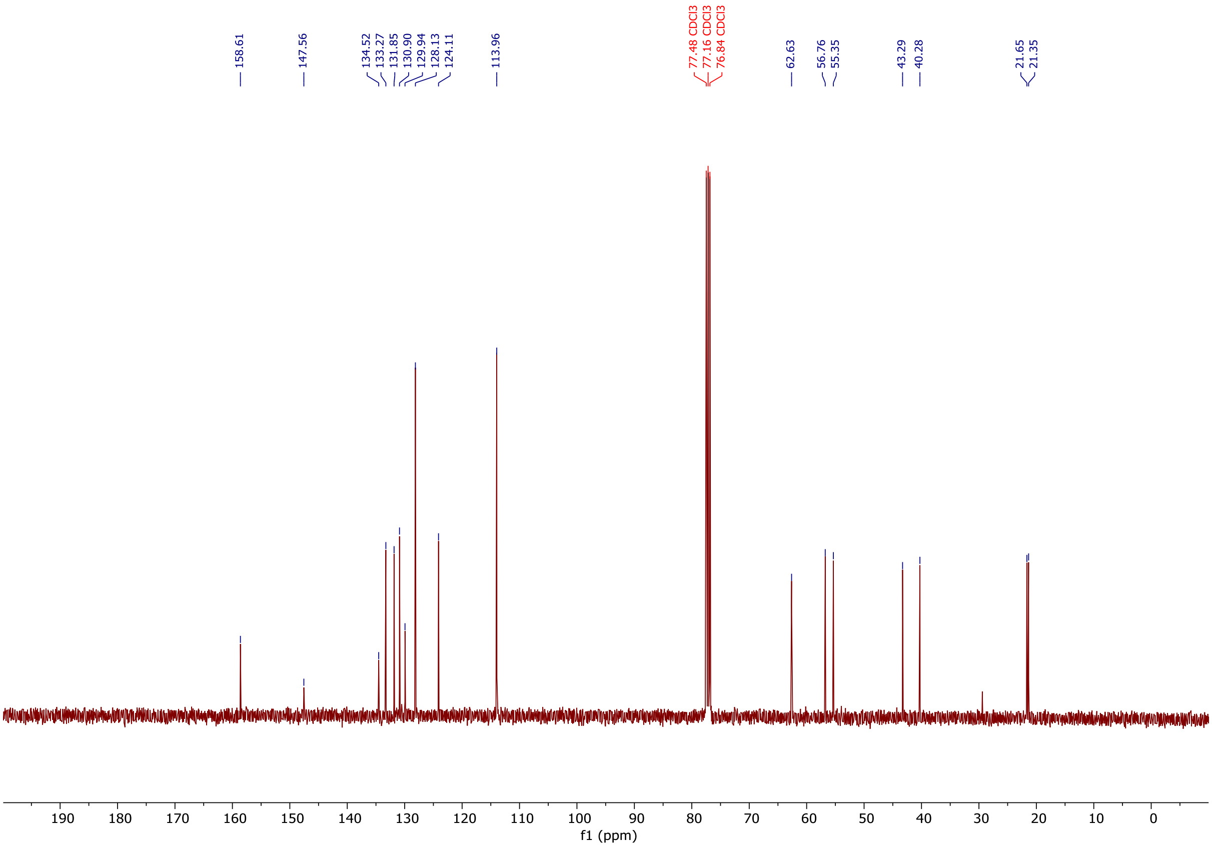

(6a): (±)-(2*S*,3*S*)-3-(4-Methoxybenzyl)-2-(4-methoxyphenyl)pyrrolidine

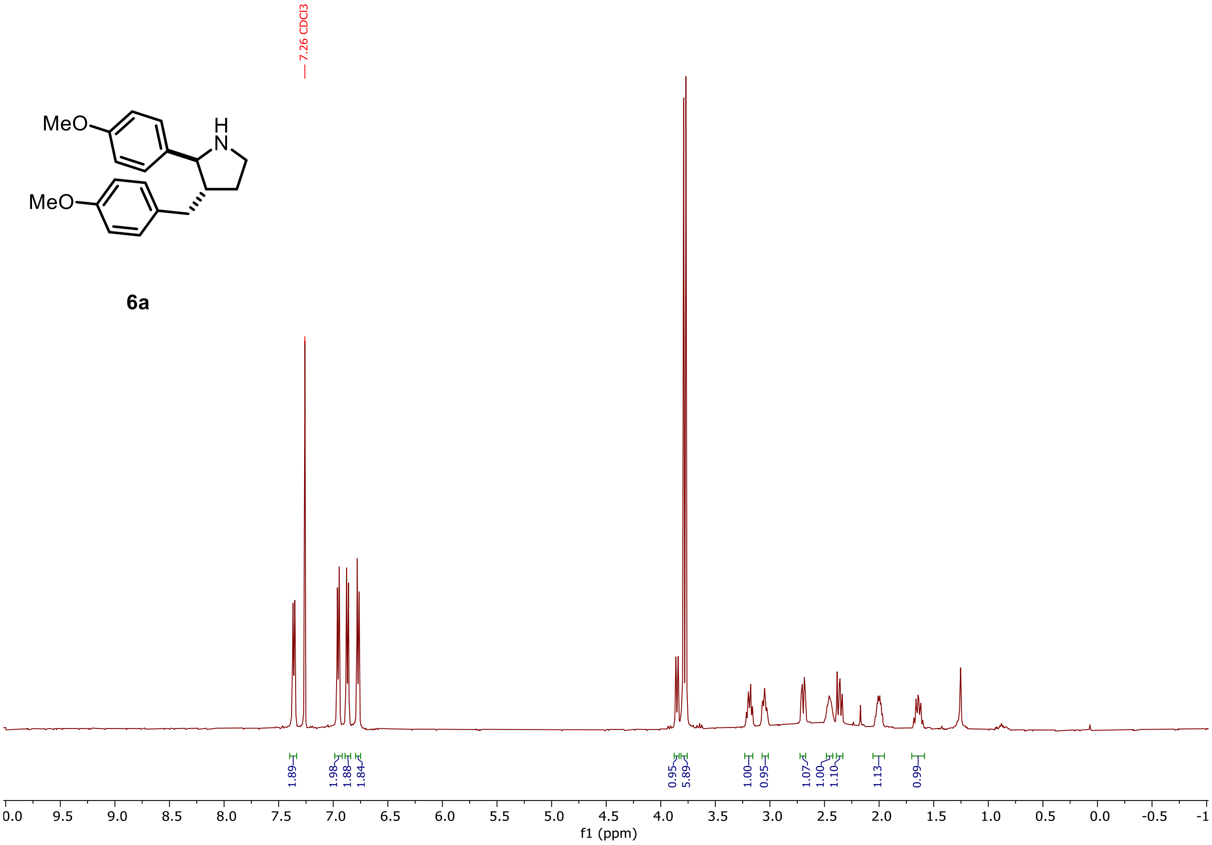

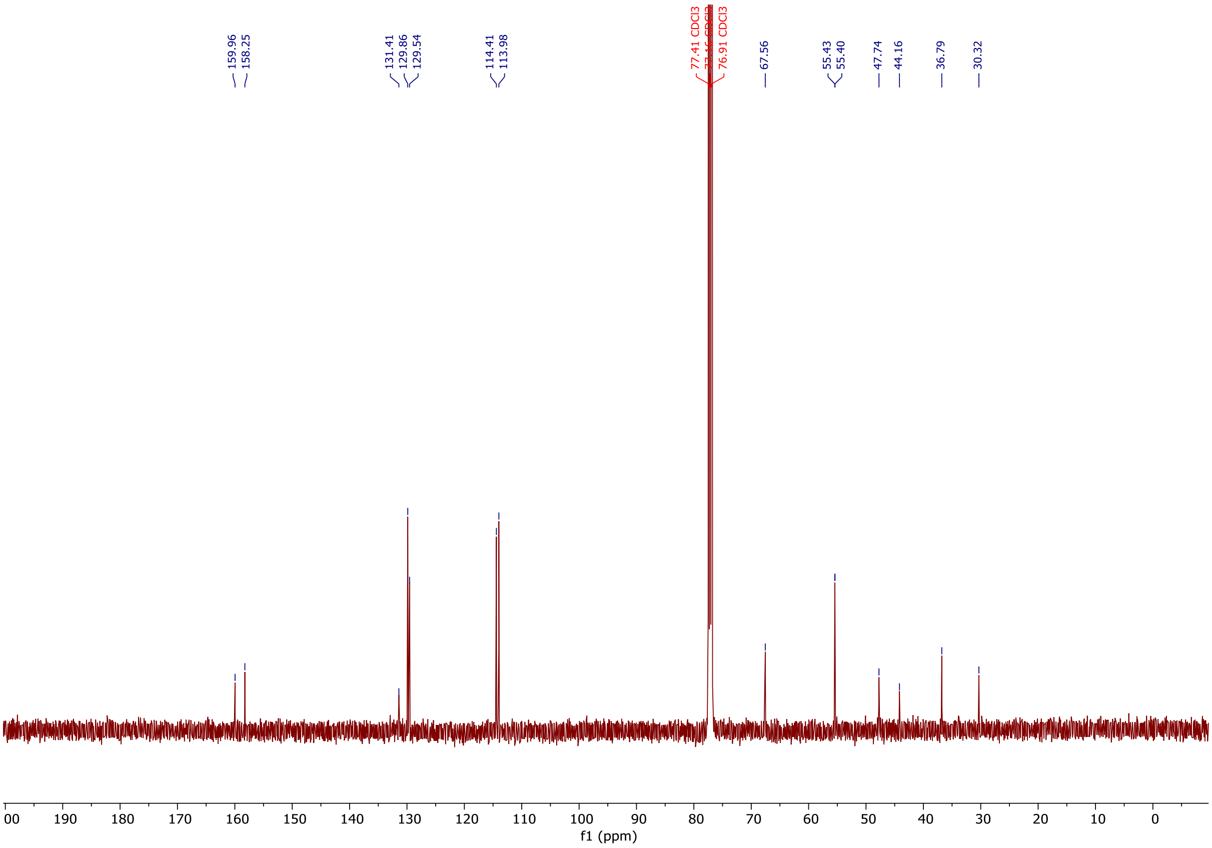

(6b): (±)-(2*S*,3*S*)-3-(4-Methoxybenzyl)-2-(4-methoxyphenyl)pyrrolidine

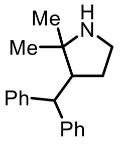

6b

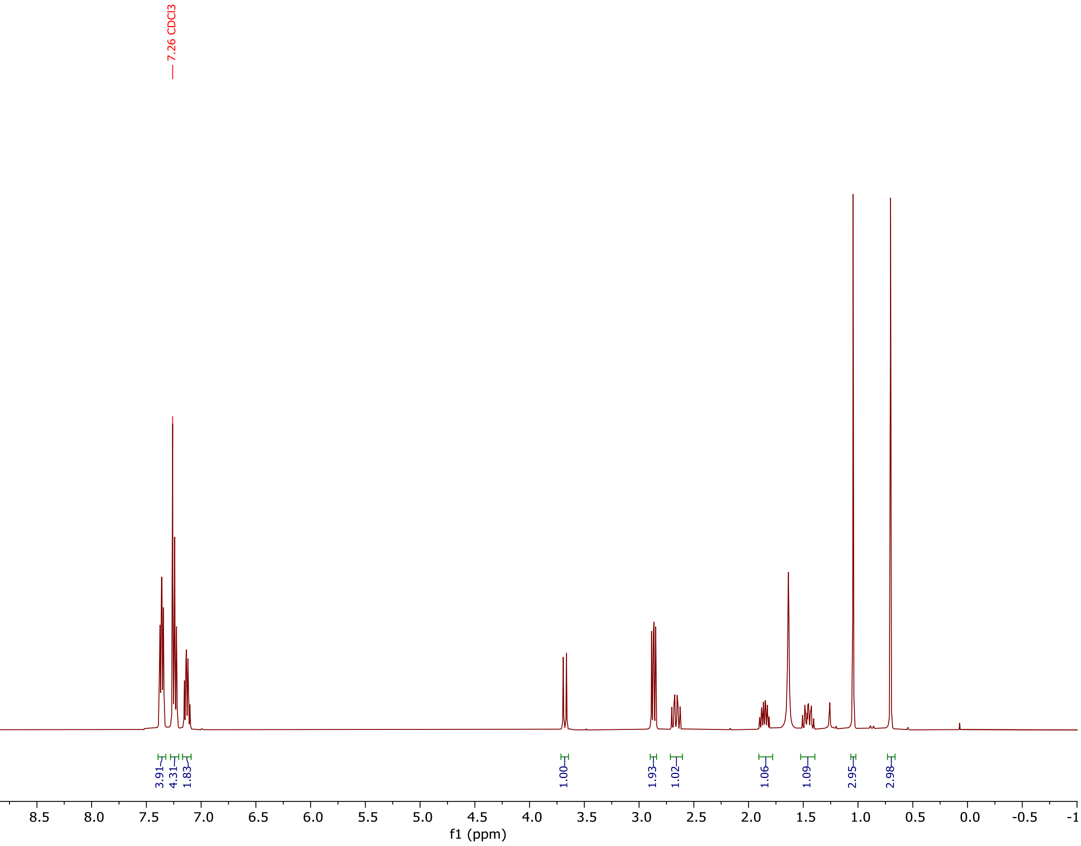

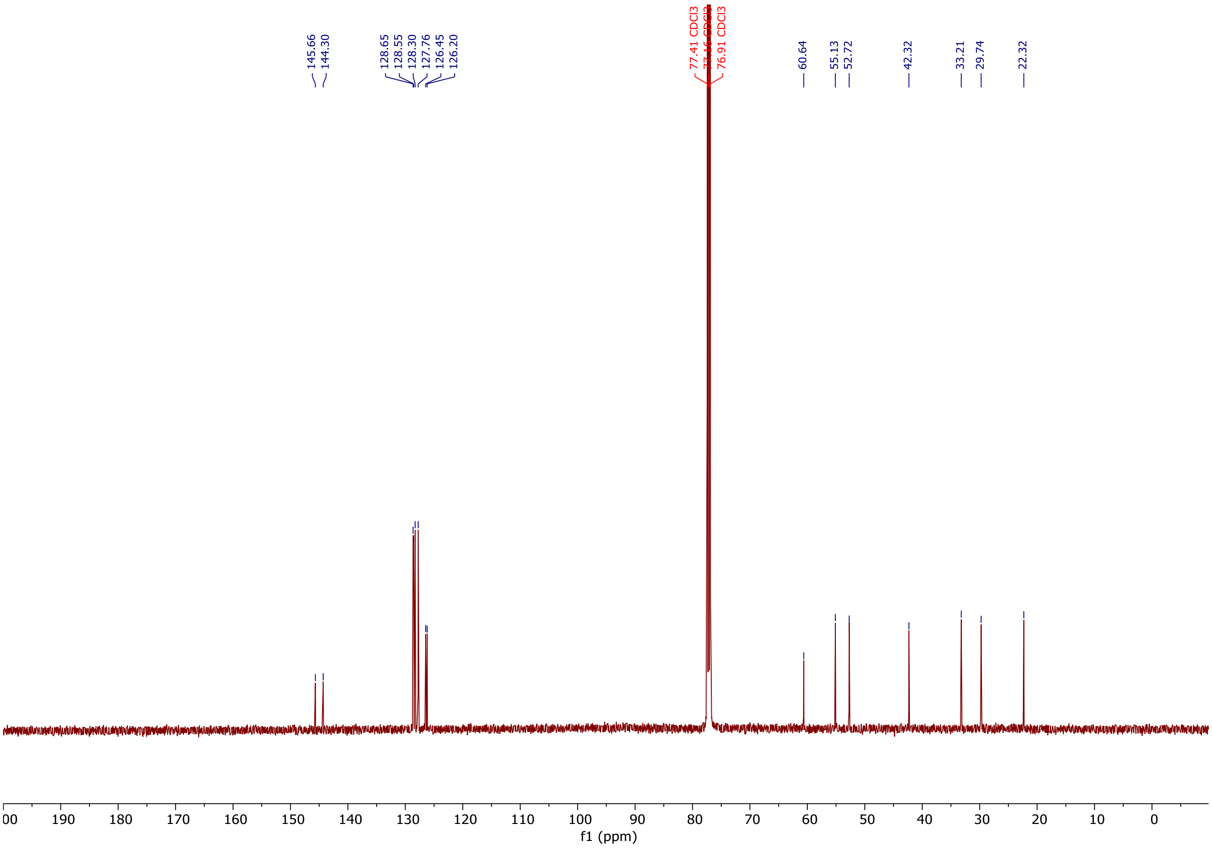

(6c): (±)- (2*S*,3*S*)-3-Cinnamyl-2-(4-methoxyphenyl)pyrrolidine

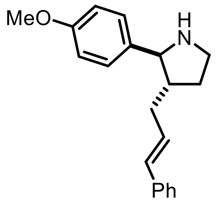

6c

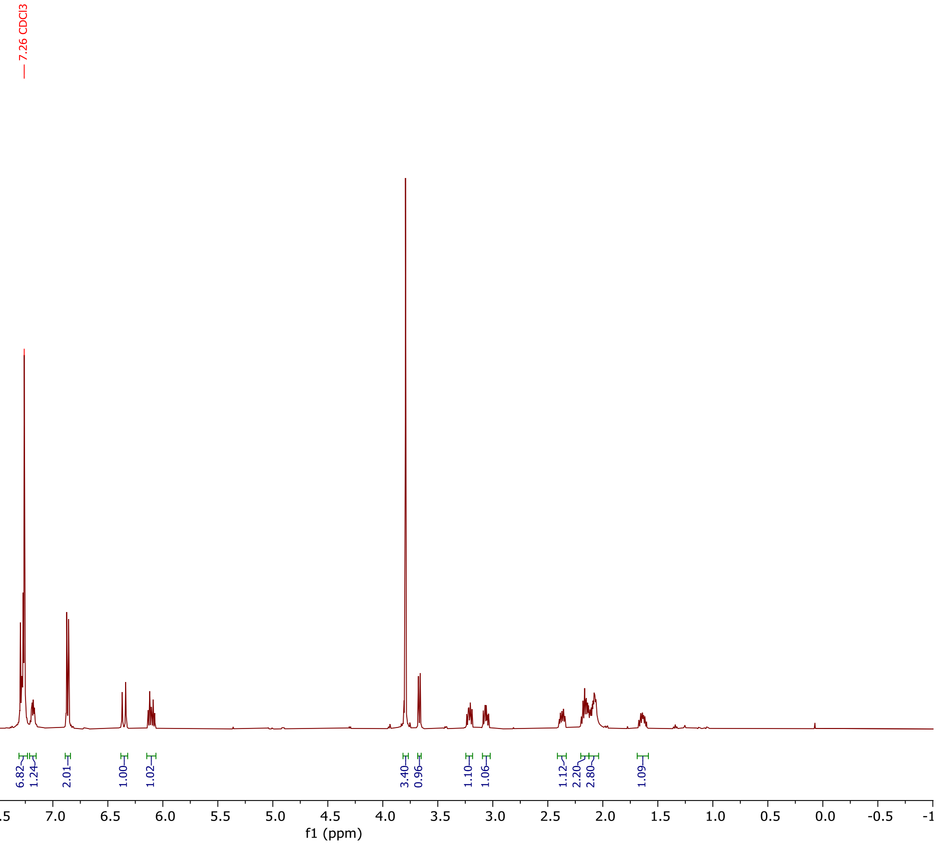

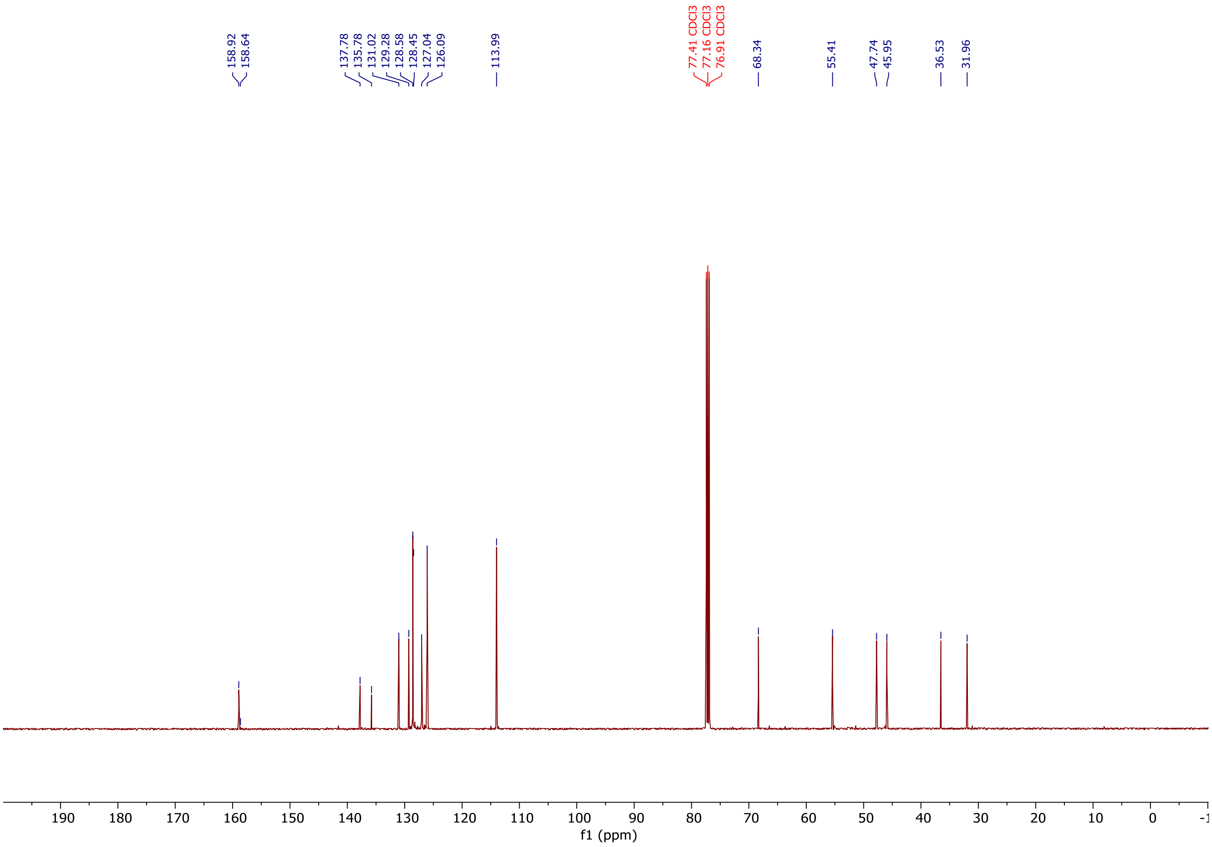

(6d): (±)-(2*S*,3*R*)-2-(4-Methoxyphenyl)-3-[(1'*R*)-3-phenylcyclopent-2-en-1-yl]pyrrolidine

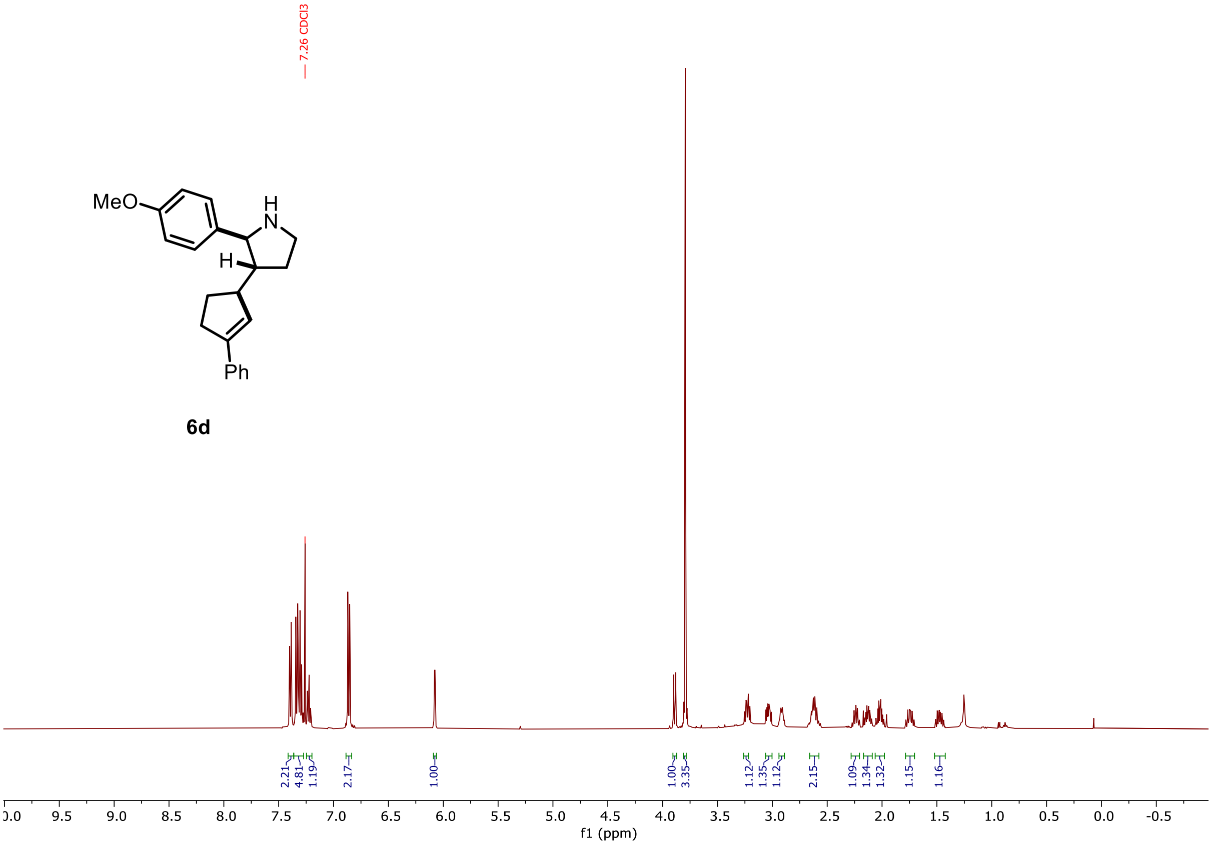

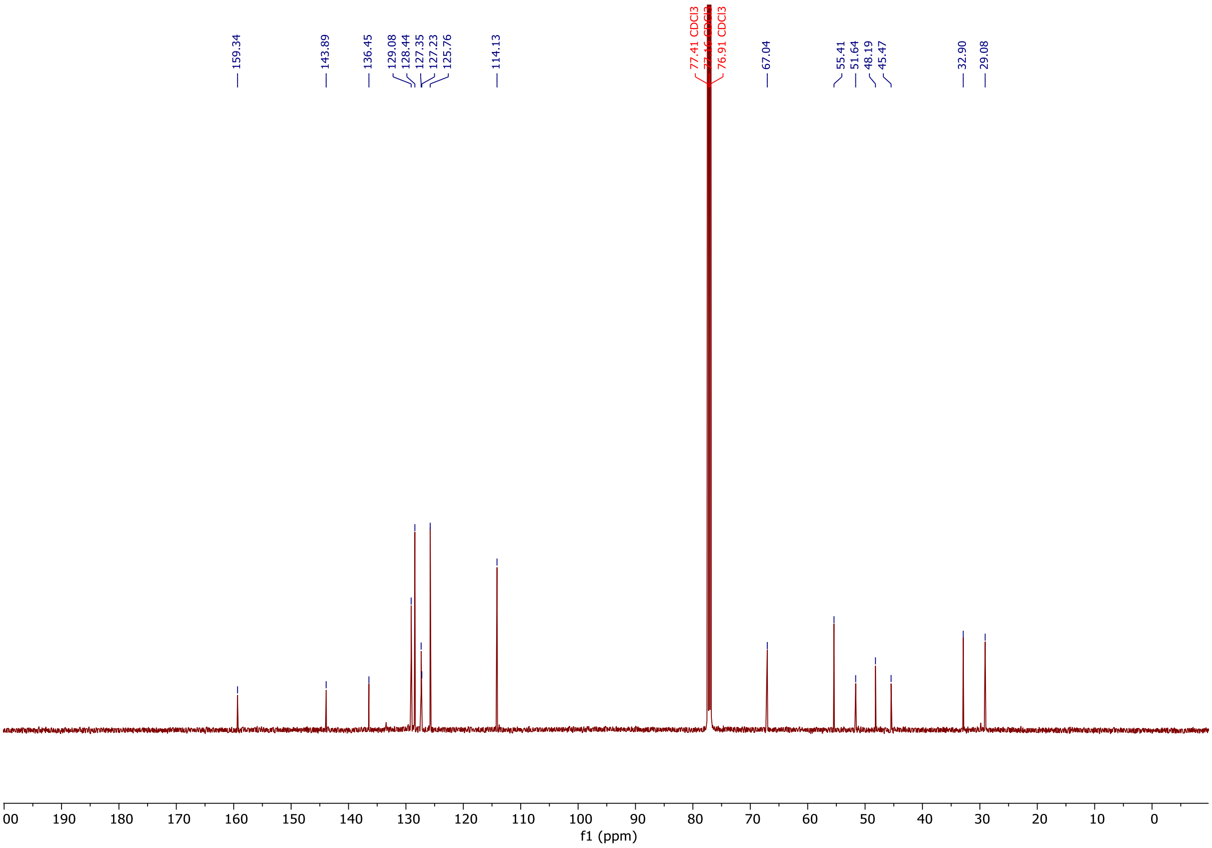

(3k'): (±)-(2*S*,3*R*)-3-((*S*,*E*)-1,3-Diphenylallyl)-2-(4-methoxyphenyl)-*N*-(4-nitrophenyl)-pyrrolidine-1-carboxamide

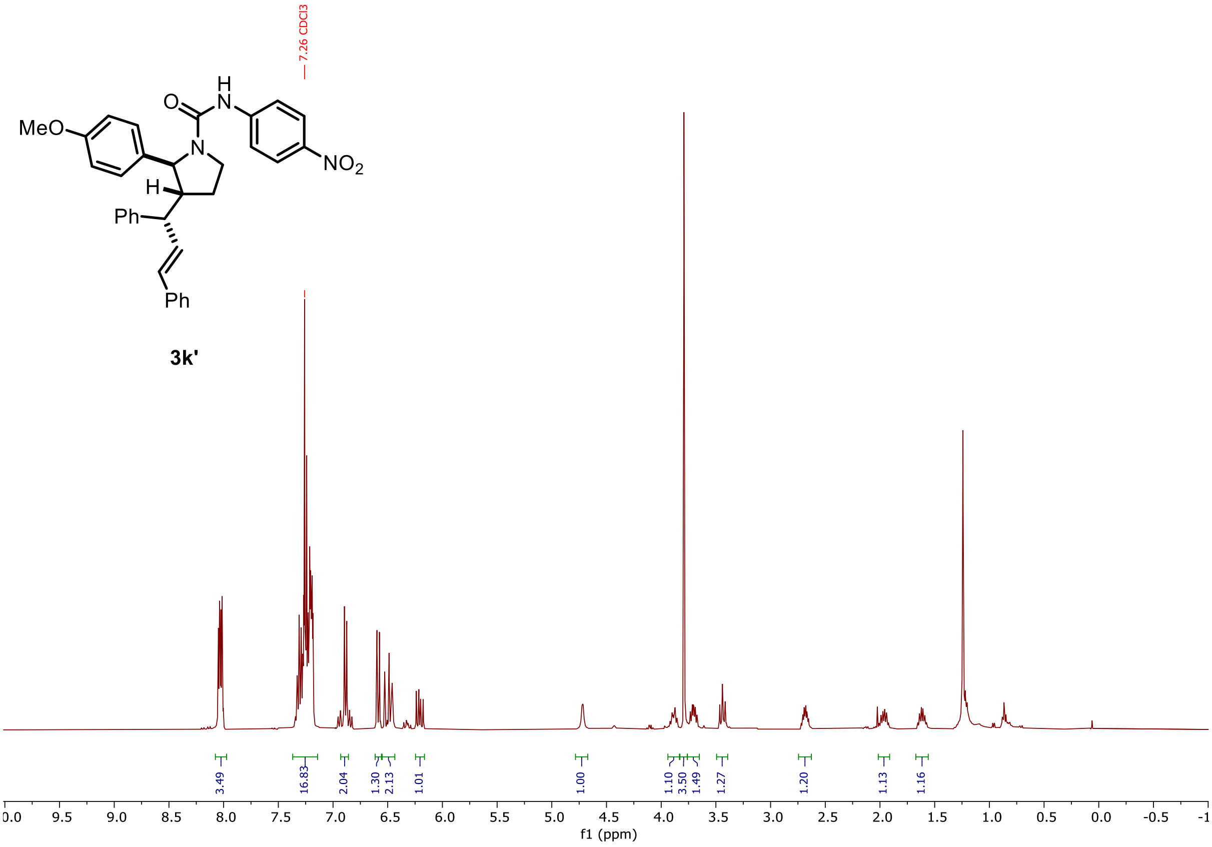

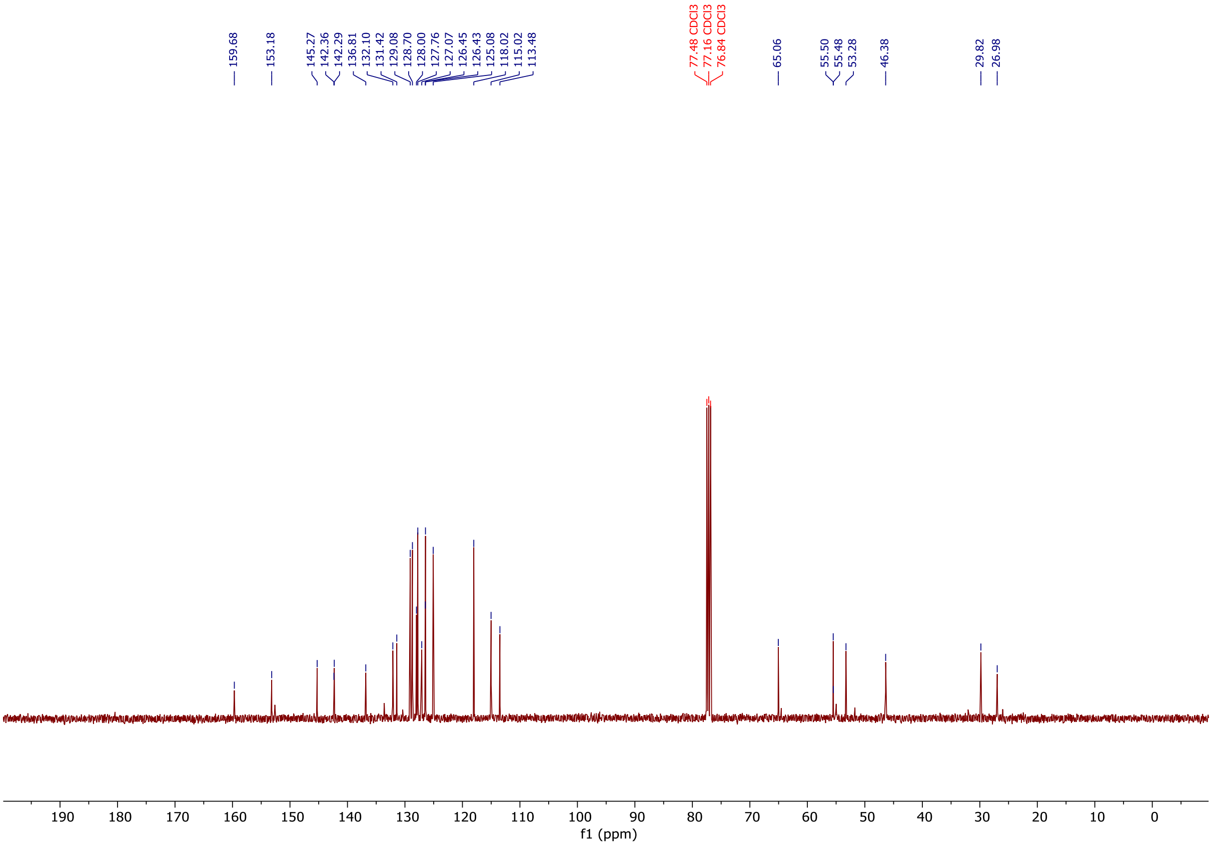

(7a): (±)-(2*S*,3*R*)-3-Benzhydryl-2-(4-methoxyphenyl)piperidin-1-ium iodide

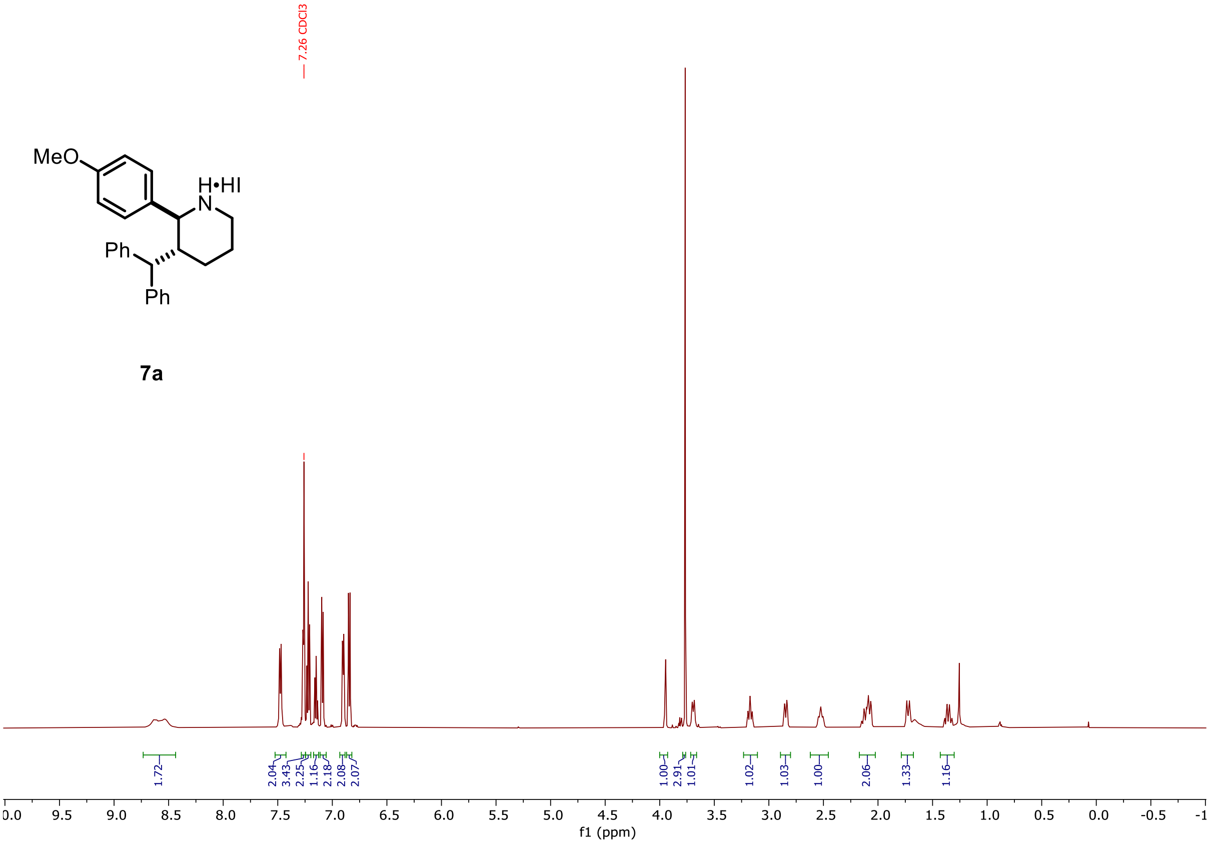

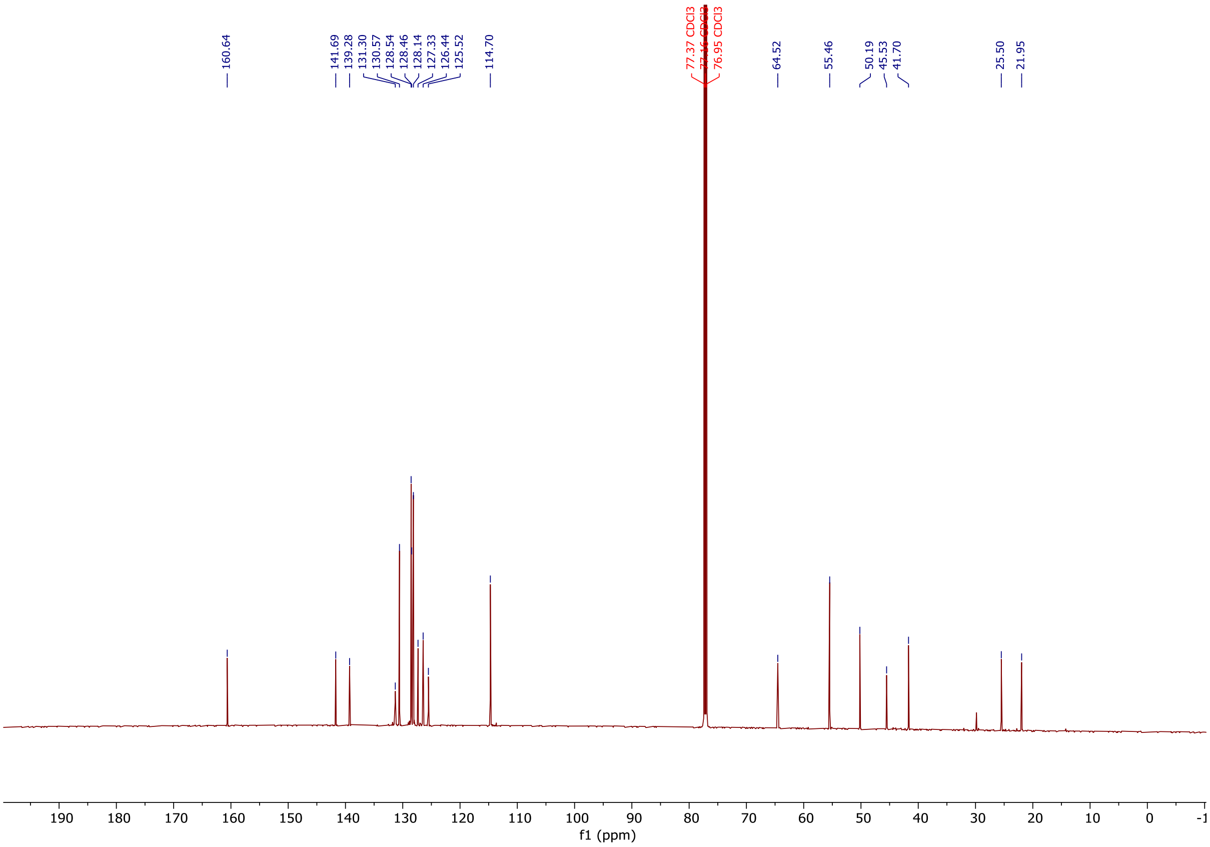

(7b): (±)-(2*S*,3*R*)-3-(4-Methoxybenzyl)-2-(4-methoxyphenyl)piperidin-1-ium iodide

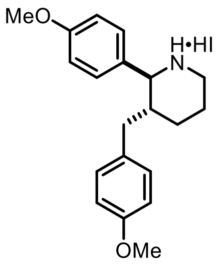

7b

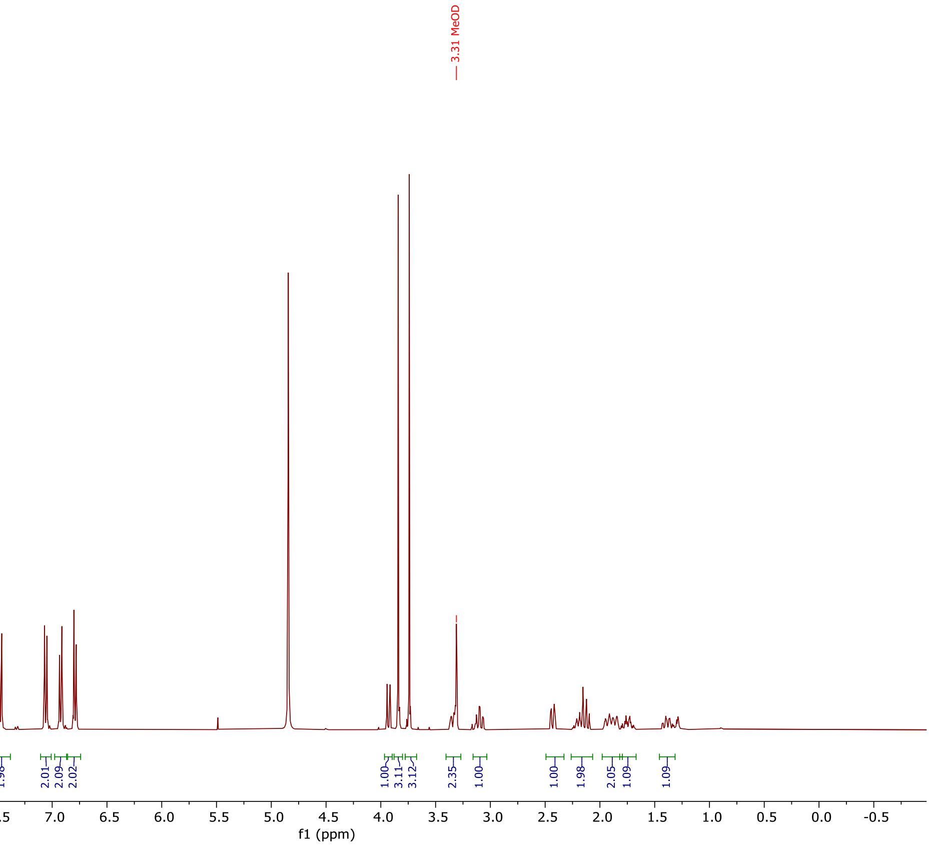

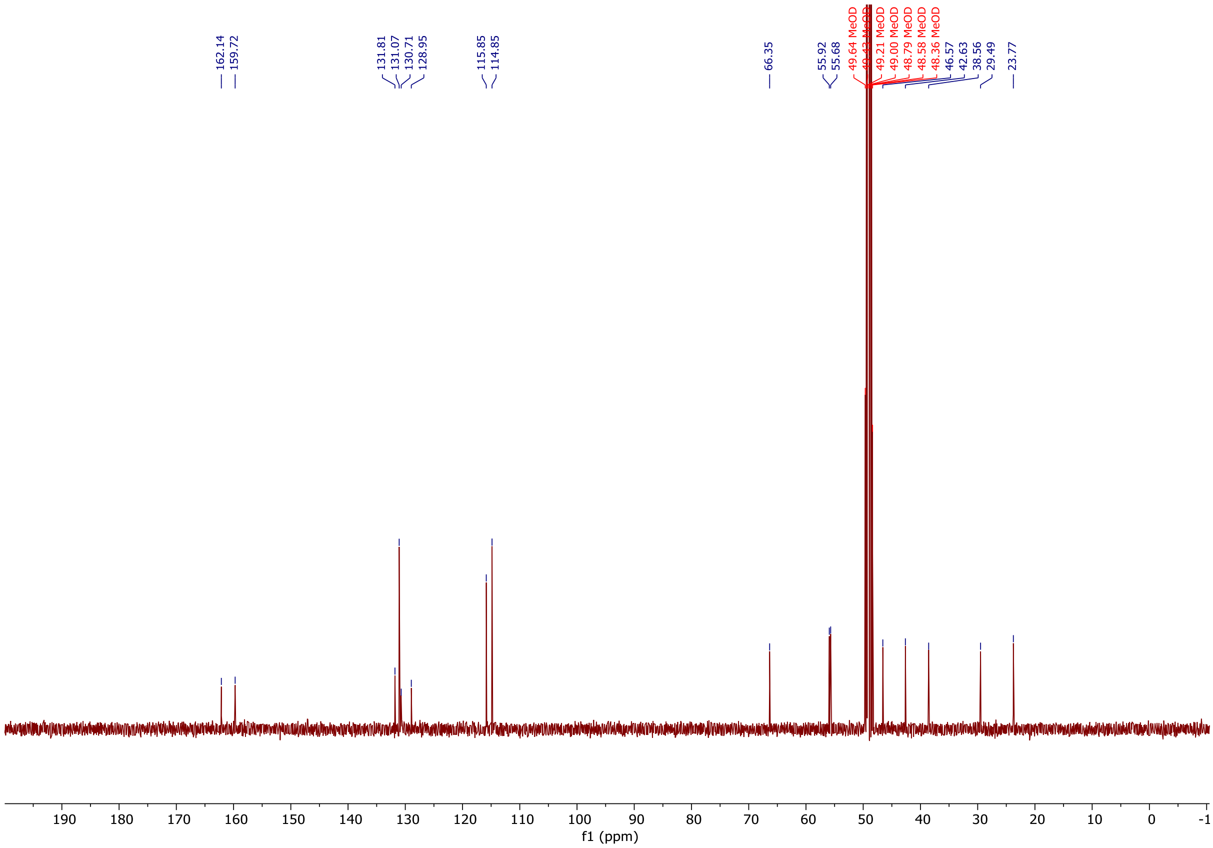

(7c): (±)-(2*S*,3*R*)-3-Cinnamyl-2-(4-methoxyphenyl)piperidin-1-ium iodide

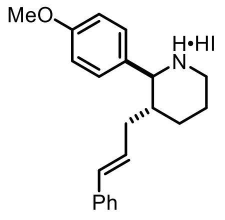

7c

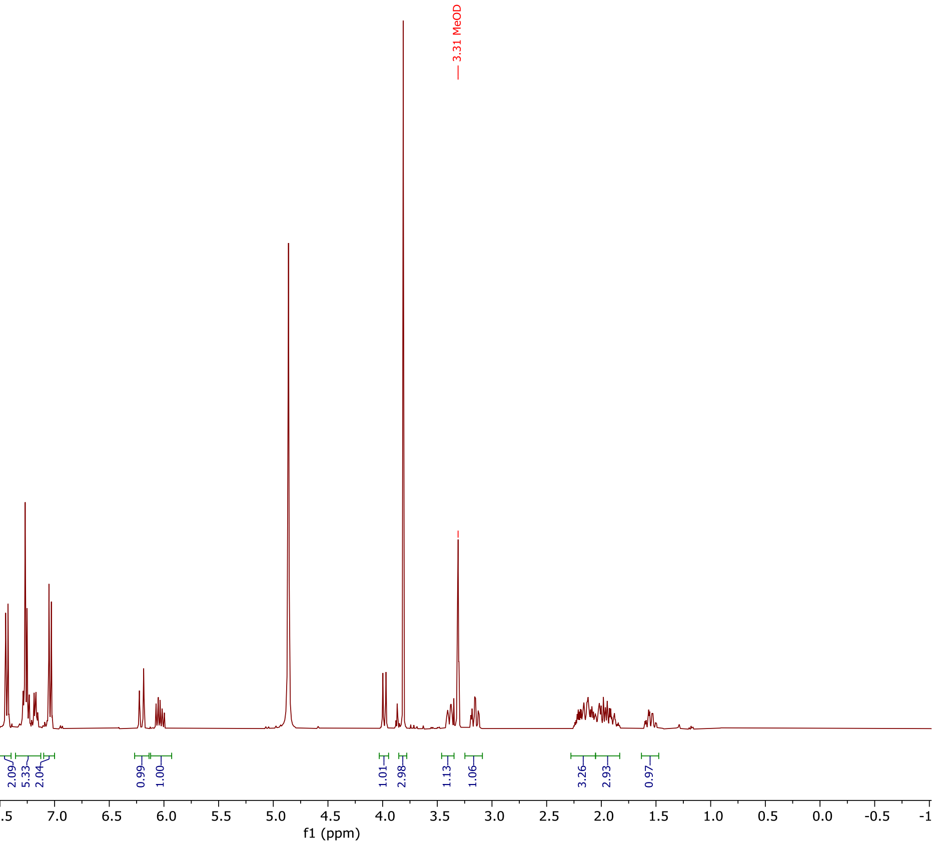

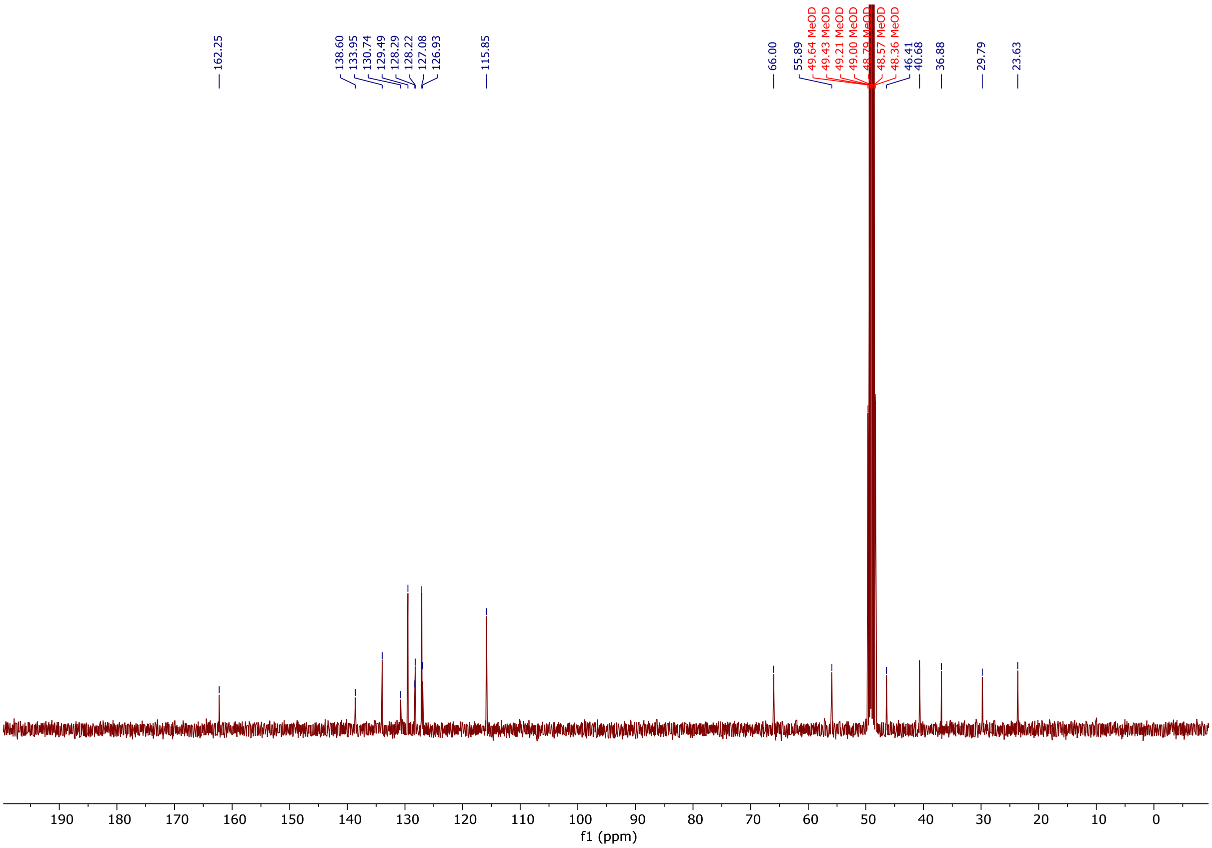

(8): (±)-Methyl (2*S*,3*R*)-3-benzhydryl-1-((2-nitrophenyl)sulfonyl)pyrrolidine-2-carboxylate

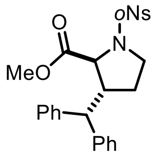

8

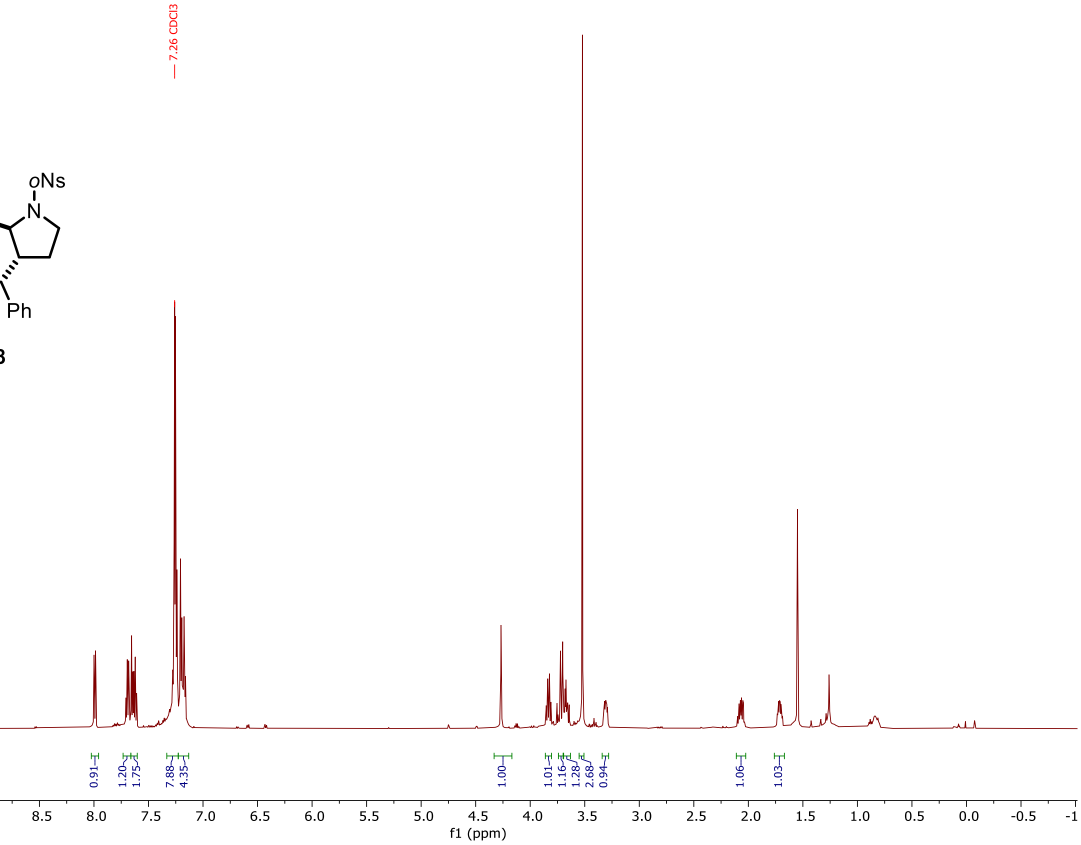

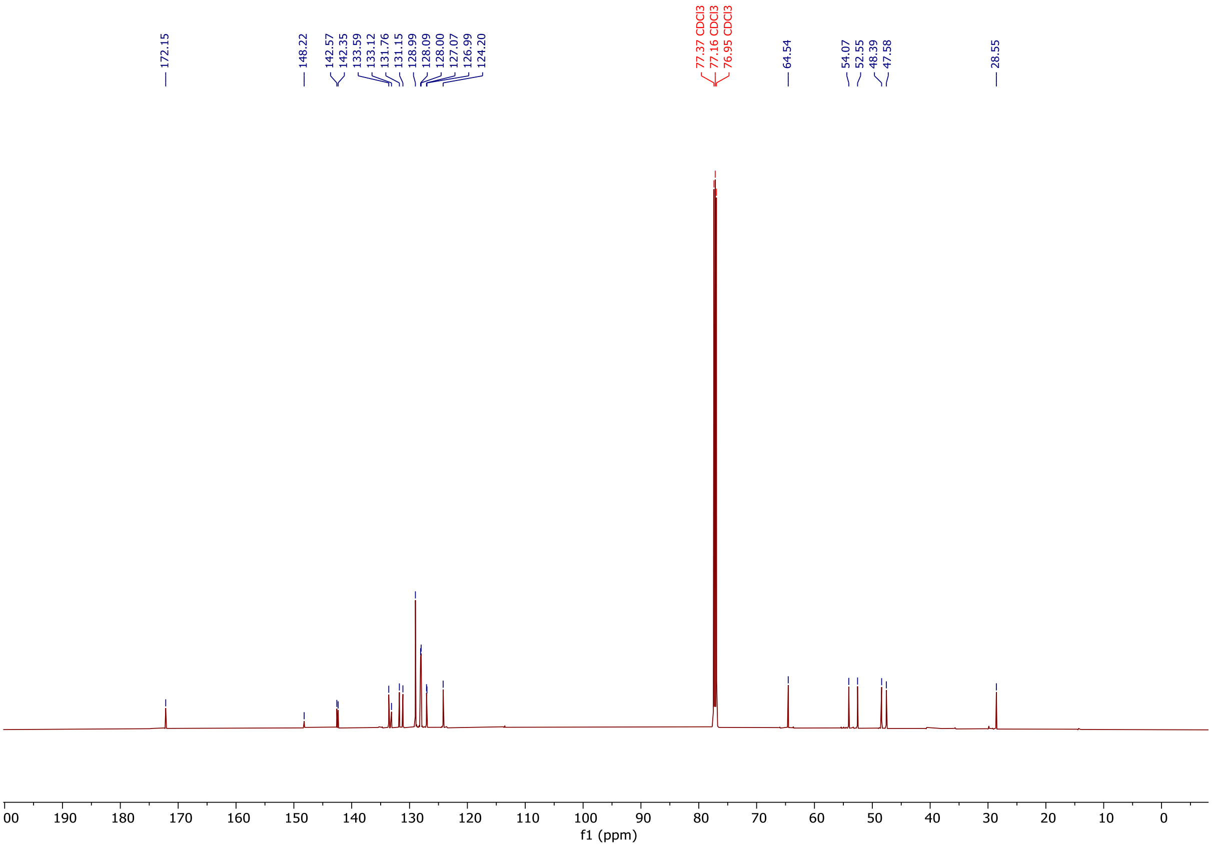

(*trans*-9): (±)-2-{(2*S*,3*R*)-3-Benzhydryl-1-[(2-nitrophenyl)sulfonyl]pyrrolidin-2-yl}methanol

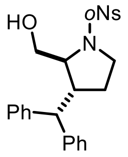

***trans*-9**

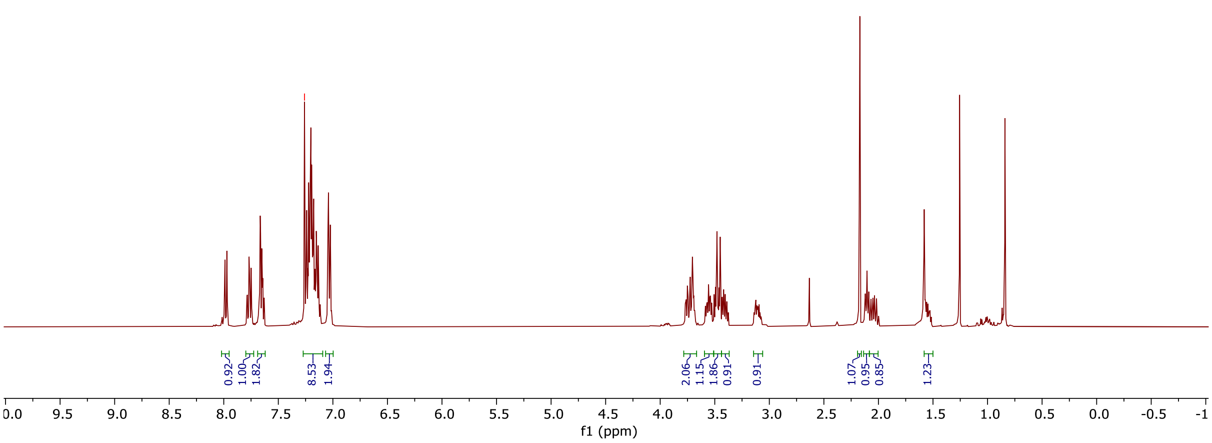

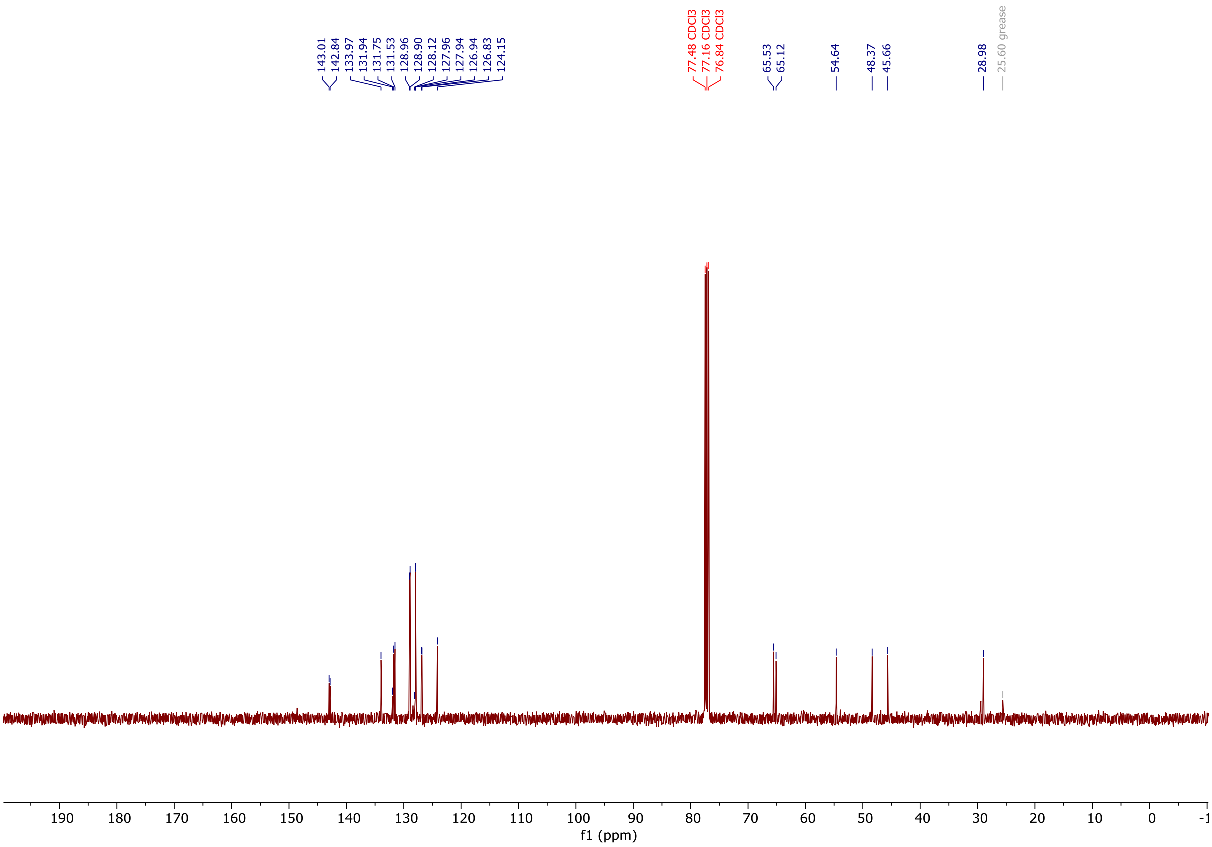

(*cis*-9): (±)-2-{(2*S*,3*S*)-3-Benzhydryl-1-[(2-nitrophenyl)sulfonyl]pyrrolidin-2-yl}methanol

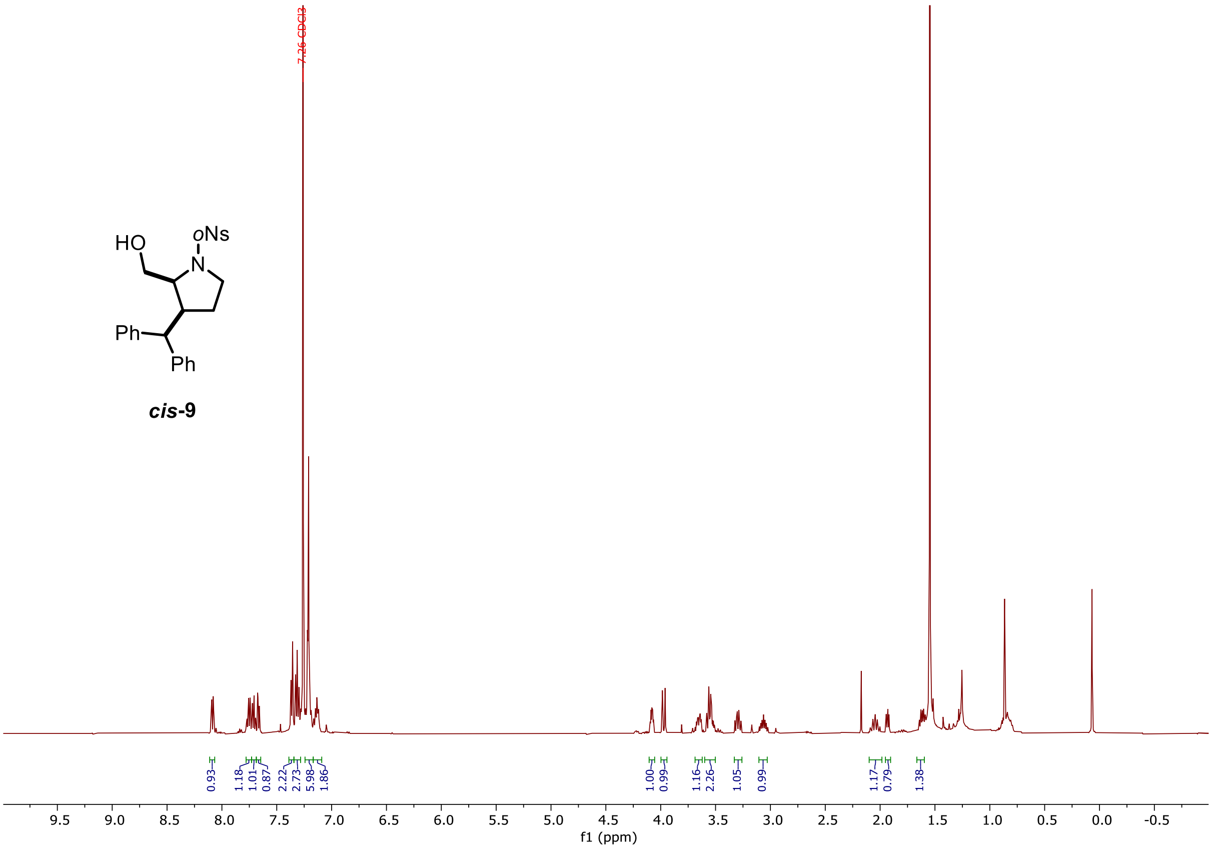

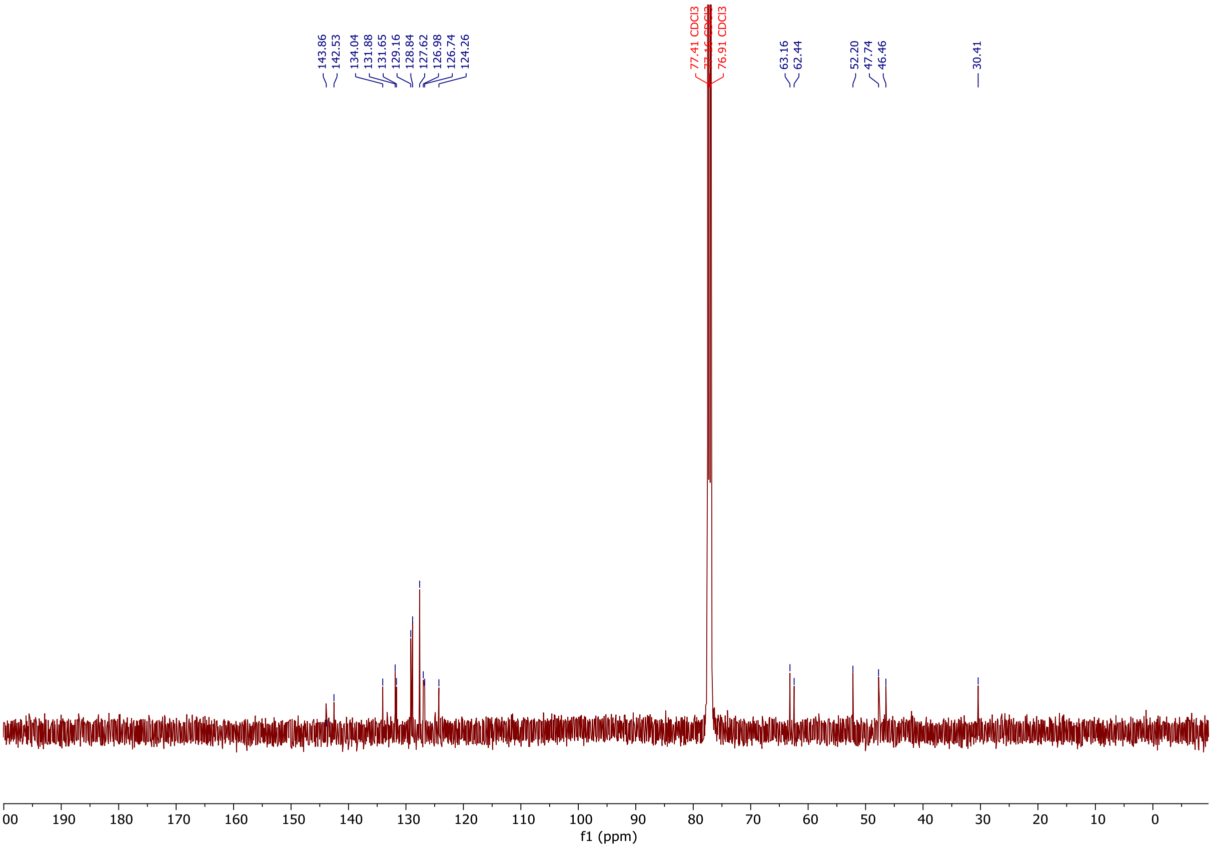

(3ah): (±)-(2*S*,3*R*)-3-(1,1-Diphenylethyl)-2-(4-methoxyphenyl)-1-((2-nitrophenyl)sulfonyl)-pyrrolidine

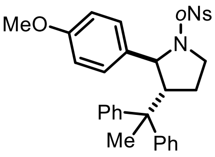

3ah

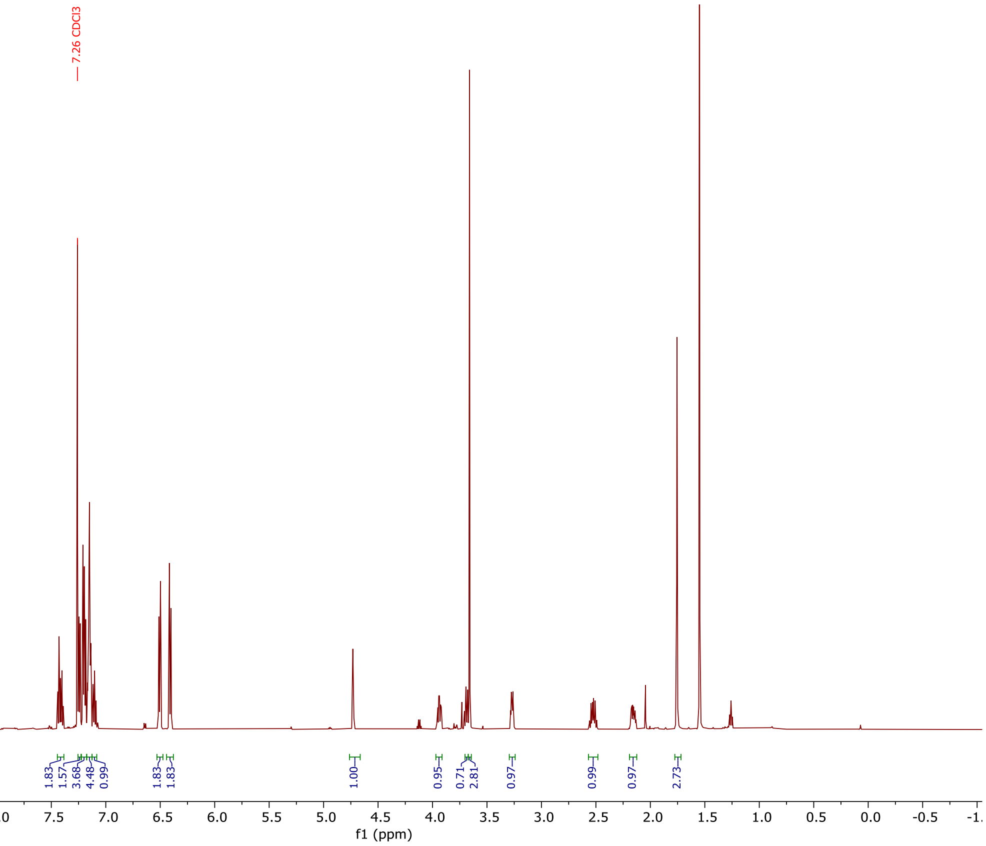

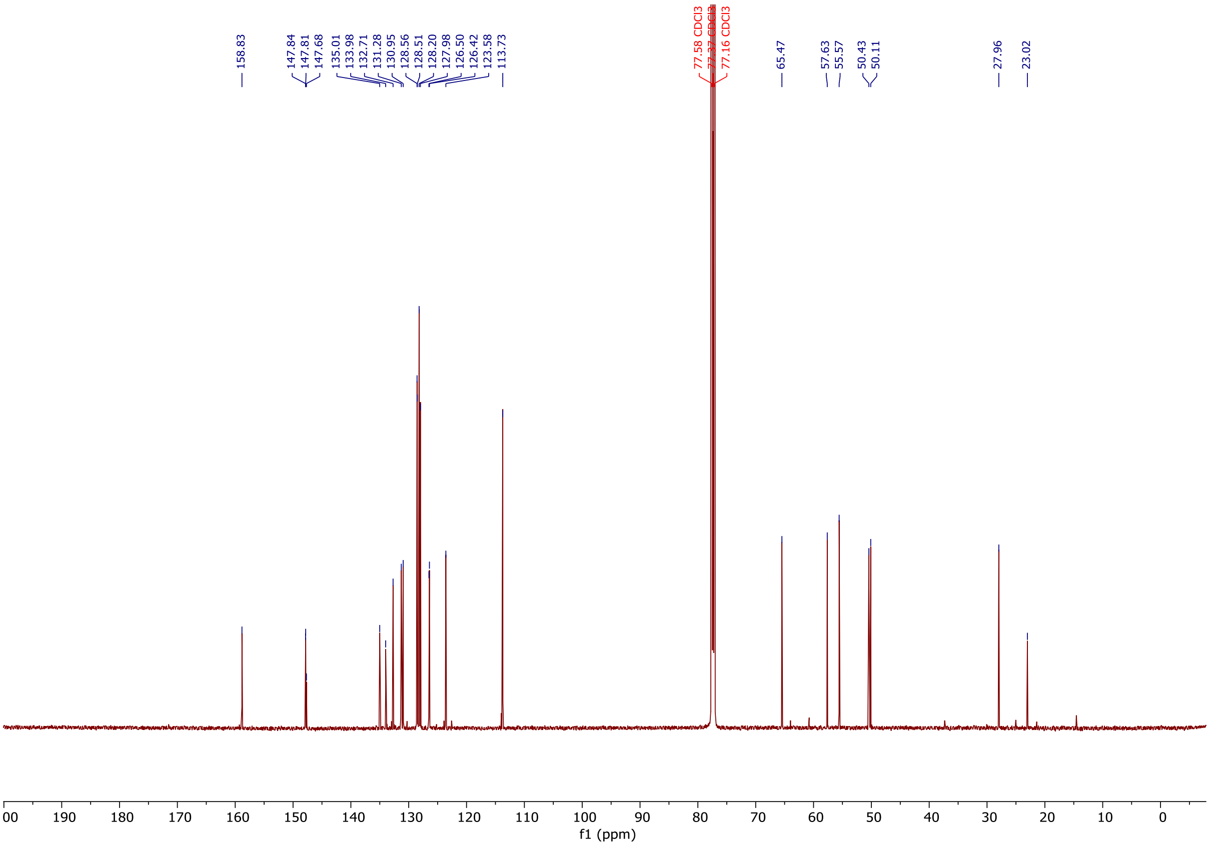

(3ai): (±)-(2*S*,3*R*)-2-(4-Methoxyphenyl)-1-((2-nitrophenyl)sulfonyl)-3-(2-phenylpropan-2-yl)pyrrolidine

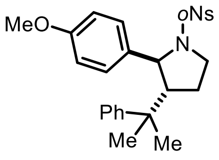

3ai

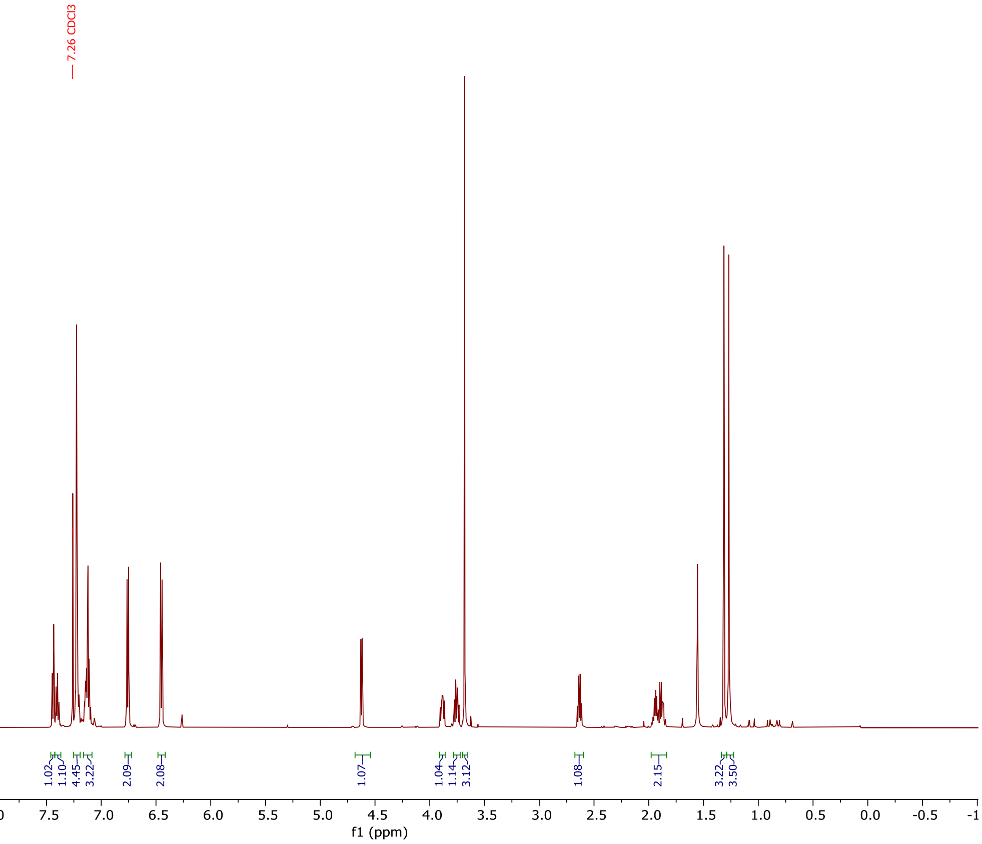

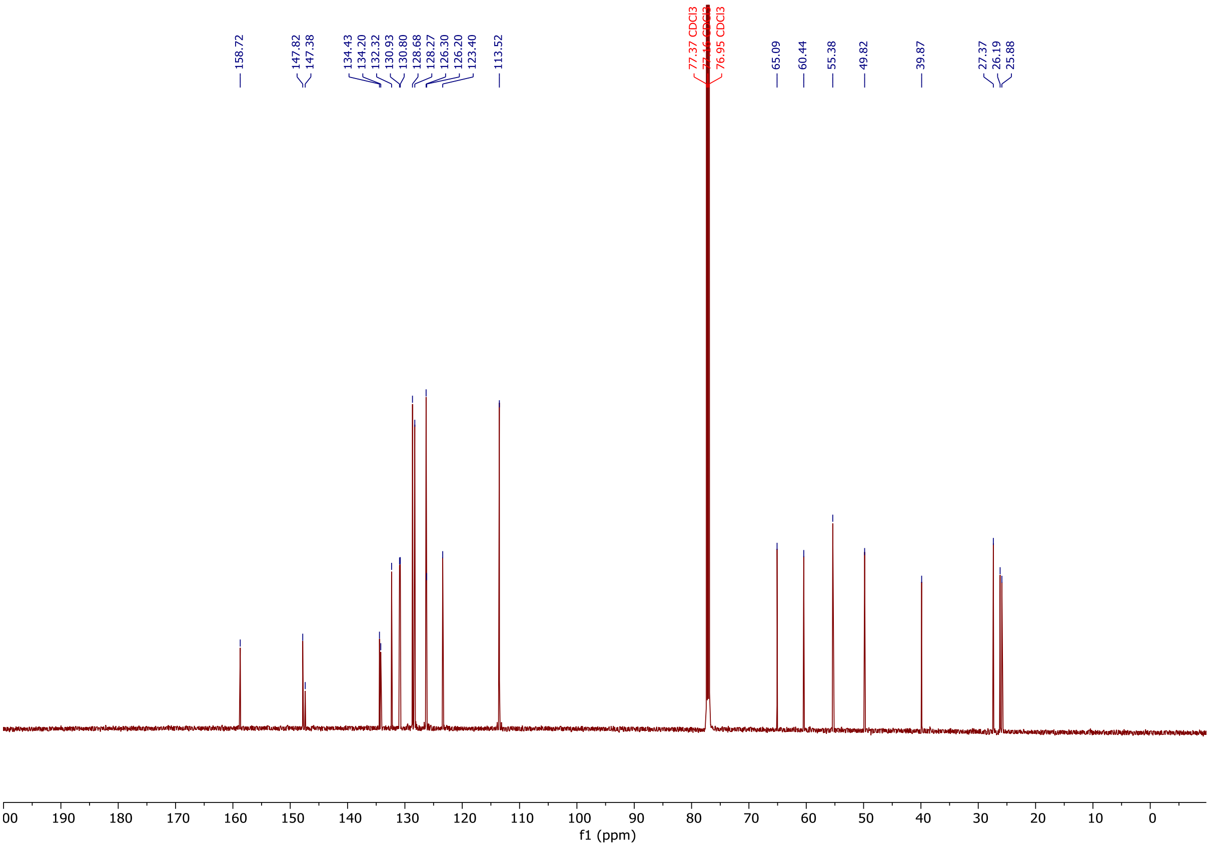

References

1. B. E. Maryanoff, A. B. Reitz, M. S. Mutter, R. R. Inners, H. R. Almond, R. R. Whittle, R. A. Olofson, *J. Am. Chem. Soc.* **1986**, *108*, 7664–7678.
2. P. R. Leger, R. A. Murphy, E. Pushkarskaya, R. Sarpong, *Chemistry – A European Journal*, **2015**, *11*, 4377–4383.
3. R. Yousefi, T. J. Struble, J. L. Payne, M. Vishe, N. D. Schley, J. N. Johnston, *J. Am. Chem. Soc.* **2019**, *1*, 618–625.
4. B. Karimi, J. Maleki, *J. Org. Chem.* **2003**, *68*, 4951-4954.
5. F. Chromans, I. H. Alder, *Chemistry – A European Journal,* **2010**, *10210*, 1445-1448.
6. T. Okamura, S. Fujiki, Y. Iwabuchi, N. Kanoh, *Organic and Biomolecular Chemisrty,* **2019**, *37,* 8522-8526.
7. M. Shi, L. Liu, J. Tang, *Org. Lett*. **2006**, *18,* 4043-4046.
8. D. Morton, S. Leach, C. Cordier, S. Warriner, A. Nelson, *Angew. Chemie Int. Ed.* **2009**, *48*, 104-109.
9. J. Dörfler, T. Preuß, A. Schischko, M. Schmidtmann, S. Doye, *Angew. Chemie Int. Ed.* **2014**, *53,* 7918–7922.
10. R. J. Lee, M. R. Lindley, G. J. Pritchard, M. C. Kimber, *Chem. Comm.* **2017**, *53*, 6327-6330.
11. M. Sugawara, J. Yoshida, *J. Org. Chem.* **200**, *65,* 3135-3142.
12. H. Knoelker, N. Foitzik, H. Goesmann, R. Graf, P. G. Jones, G. Wanzl, *Org. Lett.* **2014**, *16*, 6468-6471.
13. B. Chen, P. Cao, X. Yin, Y. Liao. L. Jiang, J. Ye, M. Wang, J. Liao, *ACS Catal.* **2017**, *7*, 2425-2429.
14. J. J. Farndon, T. A. Young, J. F. Bower, *J. Am. Chem. Soc.* **2018**, *140*, 17846-17850.
15. A. W. J. Logan, J. S. Parker, M. S. Hallside, J. W. Burton, *Org. Lett.* **2012**, *14,* 2940-2943.
16. S. E. Denmark, H. M. Chi, *J. Am. Chem. Soc.* **2014**, *136,* 8915–8918.
17. J. Chen, L. Zhou, Y. Y. Yeung, *Org. Biomol. Chem.* **2012**, *10,* 3808–3811.
18. Z. Wang, Z. He, L. Zhang, Y. Huang, *J. Am. Chem. Soc.* **2018**, *140*, 735–740.
19. I. Yalavac, S. E. Lyons, W. Webb, D. J. Procter, *Chem. Commun.* **2014**, *50*, 12863–12866.
20. S. Fu, H. Yang, G. Li, Y. Deng, H. Jiang, W. Zeng, *Org. Lett.* **2015**, *17*, 1018–1021.
21. J. Davies, L. Angelini, M. A. Alkhalifah, L. M. Sanz, N. S. Sheikh, D. Leonori, *Synth.* **2018**, *50,* 821–830.
22. X. Huo, R. He, J. Fu, J. Zhang, G. Yang, W. Zhang. *J. Am. Chem. Soc.* **2017**, *139,* 9819-9822.
23. J. J. Farnon, X. Ma, J. F. Bower, *J. Am. Chem. Soc.* **2017**, *139*, 14005–14008.
24. K. Dong, X.-L. Jin, S. Chen, L.-Z. Wu, Q. Lin, *Chem.Commun.* **2020**, *56*, 7941-7944.
25. Y. Zhu, I. Colomer, A. Thompson, T. J. Donohoe, *J. Am. Chem. Soc.* **2019**, *141*, 6489–6493.
